# Multi-locus models of deleterious epimutation-selection balance and the evolution of recombination modifiers

**DOI:** 10.1093/g3journal/jkag147

**Published:** 2026-06-11

**Authors:** Gregory Chernomas, Cortland K Griswold

**Affiliations:** Department of Integrative Biology, University of Guelph, Guelph, Ontario, Canada N1G 2W1; Department of Integrative Biology, University of Guelph, Guelph, Ontario, Canada N1G 2W1

**Keywords:** epigenetics, epialleles, transposons, selfing, recombination

## Abstract

Epigenetic inheritance has the potential to act as an additional source of phenotypic variation in natural populations of organisms. Previous theoretical works have investigated the deleterious potential of epigenetic variation at single loci. Nevertheless, epialleles are often associated with a genetic background, including the presence or absence of nearby transposons. The presence of transposons may indirectly generate epialleles at nearby loci as a byproduct of transposon regulation through methylation. Like genetic mutation, the epialleles at loci near transposons are expected to be deleterious, on average. Transposons are also known to be potentially deleterious upon insertion near genes, such that transposon insertion and subsequent epigenetic modification of nearby loci have been termed a “double edged” sword. In order to gain a deeper biological understanding of this process, this paper investigates theoretically deleterious epimutation-transposition balance. In contrast to previous theoretical works, this model is multi-locus, which gives rise to additional fundamental biological processes in comparison to single-locus models. In particular, recombination rates are a major element of multi-locus models. Our analytical and numerical results suggest the evolution of reduced recombination as a result of the balance of transposition and epimutation; however, we also find conditions where recombination may evolve to increase, such as due to a high background epiallele reversion rate, as found in mammals. Additionally, since selfing reduces the effective recombination rate, there may be a potentially critical interaction between mating systems and transposition-epimutation deleterious effects.

## Introduction

There is greater recognition that epigenetic effects at one locus can affect other loci, such as when an epigenetically tagged transposon causes secondary epigenetic gene expression changes at neighboring genetic loci (Colonna Romano and Fanti 2022). Transposons tend to be highly methylated to prevent transposition (Lyons et al. 2023). This is a key regulatory process as transposition is generally deleterious, particularly when insertions affect genetic regulatory or protein sequences (Fouché et al. 2022). For genomic regions with transposons, it has been shown that the ratio of forward to reverse spontaneous methylation events can be significantly greater than in other regions of the genome (van der Graaf et al. 2015), presumably as a secondary effect of selection to regulate transposons by methylation. Following this, there is also evidence of phenotypically non-neutral epialleles segregating in populations caused by the presence of transposons (Shah 2022). For example, transposon-associated segregating non-neutral epialleles have been identified in a few species, including melon (Martin et al. 2009), tomato (Quadrana et al. 2014), and mice (Morgan et al. 1999). Overall, transposons may be a significant associated genomic source of heritable, and non-neutral epigenetic variation in populations, which allows for evolutionary implications of such variation (Bonduriansky and Day 2018).

Spontaneous epimutation events, such as those caused indirectly by transposon regulation, are expected to be deleterious on average following the classic understanding of spontaneous mutation (Charlesworth et al. 2017). There is empirical evidence that epigenetic effects caused by transposons are usually deleterious (for a review, see Choi and Lee 2020). Some have termed the deleterious effects of transposition and the secondary deleterious epigenetic effects due to regulation of transposition the double-edged sword of transposons (Choi and Lee 2020). We note that more evidence and more direct measurements of the distribution of fitness effects for natural epialleles associated with transposons are needed to help support these expectations and determine the broader evolutionary significance. There is also a need for theoretical predictions of the evolutionary implications of transposon-associated spontaneous epimutations to support empirical investigations and for a deeper biological understanding of these processes.

Previous works have studied theoretical (epi)mutation-selection balance at a single locus, with consideration of both adaptive and/or deleterious epialleles (Stenøien and Pedersen 2005; Geoghegan and Spencer 2012, 2013a, 2013b; Furrow 2014; Chernomas and Griswold 2024; Webster 2024). These studies have considered key processes such as environmental shifts (Geoghegan and Spencer 2013a), ploidy (Stenøien and Pedersen 2005), and inbreeding (Chernomas and Griswold 2024) and have shown how they impact frequencies of alleles and epialleles at equilibria. Some models allowed for coexisting genetic and epigenetic variation at a locus, such that both a mutational and epimutational load can be present simultaneously (Stenøien and Pedersen 2005; Geoghegan and Spencer 2012; Chernomas and Griswold 2024; Webster 2024). Epialleles can behave similarly to genetic alleles in these models, but a relatively high reversion rate of epialleles and other types of epigenetic processes, such as paramutation (Geoghegan and Spencer 2013b), can lead to departures from classic expectations of adaptive and/or deleterious variation at equilibrium.

When multiple loci are considered, recombination and indirect selection are major processes that can potentially impact the degree of segregating deleterious variation. Most relevant to the current study are multi-locus models where a transposon is variable at a genetic locus and where it can have secondary epigenetic effects on a neighboring locus. One previous theoretical study demonstrated results pertaining to the probability of fixation and time to fixation of a transposon under conditions where it could adaptively (or neutrally) modify the expression of a neighboring gene (Branciamore et al. 2015). These authors show that (adaptive) epigenetic effects caused by the presence of a transposon increase the rate and probability of fixation of a transposable element. They also note these effects correlate with increases in the effective population size. Another study used an ODE-based model to analyze detailed interactions of host TE silencing mechanisms and TE transposition/deletion events (Roessler et al. 2018). Based on the balance of transposition and a transposon-deleting/silencing mechanism, they identified parameter conditions where transposons will be purged or maintained at equilibrium, and interestingly, they also found conditions where oscillations of transposon frequencies occur (Roessler et al. 2018), although they did not include major evolutionary forces such as natural selection in their model. We note there is a gap in the understanding of interactions between the deleterious epigenetic effects of transposons and major evolutionary processes such as natural selection, recombination, and selfing.

In this study, we seek to address this gap. Using classic population genetics approaches, we derive a set of theoretical models to gain insights into deleterious transposon epigenetic effects, assuming that spontaneous epialleles generated by transposons are detrimental to fitness. To our knowledge, this paper is the first to study 2 and 3 locus models of deleterious-epimutation selection balance theoretically. Specifically, for 2 loci, one locus is a transposon locus and the second locus is the epiallele locus, and for 3 loci, a third locus is a modifier of recombination. We incorporate selfing to allow for inferences in relation to mating systems, which are quite variable in nature and have critical interactions with deleterious variation (Charlesworth 2006; Barrett 2013). Additionally, a major research area relevant to these models is the evolution of recombination rates. There is a rich set of theoretical predictions on the evolution of recombination, yet transposon-associated epigenetic effects are an unexplored factor.

In particular, theoretical works have identified specific mechanisms for when recombination is an adaptive evolutionary process. Heterogenous selection (Lenormand and Otto 2000), epistasis (Otto and Feldman 1997), genetic drift, and deleterious (Kondrashov 1984; Keightley and Otto 2006) and adaptive mutation (Hartfield et al. 2010) rates can predict the evolution of increased recombination. However, there are also conditions that favor reduced recombination because it can indirectly act as a deleterious process, such as at equilibria where adaptive haplotypes are already at high frequencies (Altenberg et al. 2017).

Epimutation has unique properties relative to genetic mutation. In particular, the reversion rate of epialleles can be very high (Charlesworth et al. 2017), which is not known to be possible for genetic alleles. Also, genetic background can induce strong asymmetry in rates (van der Graaf et al. 2015). These distinct epimutational processes may lead to unique interactions with recombination and lead to novel biological conditions for when recombination is anticipated to evolve. For example, in relation to this study, transposons induce deleterious epimutation, yet recombination may decouple a transposon from the deleterious epiallele that it generates, weakening selection against the transposon and allowing the transposon to generate a new epiallele.

The paper studies (epi)genetic mutation-selection balance and the evolution of a recombination modifier with both haploid and diploid selection. The main text of the paper focuses on the haploid case and the diploid case is presented in Supplementary material because its results generally follow the haploid case.

### Overview of models

Models assume an infinite population size and have 2 or 3 loci. For both the 2 and 3 locus models, there is a transposon locus (T/t) and an epigenetic locus (A/B). For the transposon locus, a transposon is either present (T) or absent (t), with frequencies p_T_ and p_t,_ respectively. We study 2 models. Model 1 considers a haploid population with 2 loci: a transposon locus and an epigenetic locus. For Model 1, 2 subcases are analyzed. In Case A, the transposon is neutral, whereas in Case B, the transposon is deleterious; otherwise, the 2 cases share identical assumptions. Model 2 considers a haploid population with 3 loci: a transposon locus, an epigenetic locus, and a modifier of recombination locus, in order to investigate the evolution of recombination rates.

For all models, transposon insertion at the locus occurs at a rate *z* and transposon deletion occurs at rate *y*.


$$t\begin{array}{c} z\\ ⇄\\ y \end{array}T$$

Following this, we assume the deletion rate at the insertion site is greater than the insertion rate. This assumption is justified because the insertion occupies a small region, such that the probability of insertion is low. Although the probability of insertion is low because the region is small, this does not affect the deletion rate per transposon. To more concretely define the limits of this assumption, we next apply the Markov theory of a random birth-death process, with a reflecting boundary at zero. In particular, the probability ($\pi_{i}$) of i transposons being present at a site is (see Supplementary File 7 for details)


$$\pi_{i}=\left(\frac{y-z}{y}\right){\left(\frac{z}{y}\right)}^{i},$$

such that the probability of more than one transposon being present is


$$P(i>1)=\frac{z^{2}}{y^{2}}.$$

Our paper assumes this probability is small. Accordingly, if y = *a*z, then for a choice of *a*, $P(i>1)=1/a^{2}$. In our analysis, we choose an *a*, such that *P*(*i* > 1) < 0.001. Overall, by choosing a sufficiently large value for a, our modeling approach follows other works that assumed the presence or absence of a transposon (Branciamore et al. 2015; Roessler et al. 2018).

A second adjacent (epi)genetic locus has 2 possible states: a wild-type state (A) with frequency p_A_ and an epiallele variant state (B) with frequency p_B_. When a transposon is not present in cis, rates of forward and reverse epimutation are *α*_1_ and *β*_1_, respectively.


$$\mathrm{tA}\begin{array}{c} \alpha_{1}\\ ⇄\\ \beta_{1} \end{array}\mathrm{tB}$$

When a transposon is present, rates of forward and reverse epimutation are *α*_2_ and *β*_2,_ respectively.


$$\mathrm{TA}\begin{array}{c} \alpha_{2}\\ ⇄\\ \beta_{2} \end{array}\mathrm{TB}$$

Equilibrium frequencies of the epiallele and transposon are derived analytically in a haploid 2-locus setting for Model 1 (see results section below), when the epiallele is deleterious, but the transposon is neutral (Case A), and when both are deleterious (Case B).

We also study a three-locus model in which the third locus acts as a modifier of recombination rates between loci. The arrangement of loci along a chromosome is the modifier, then the transposon locus, and then the epigenetic locus. Adjacency of the transposon to the epigenetic locus reflects cis effects of the transposon (Choi and Lee 2020). Recombination can occur between all adjacent loci. The modifier locus has 2 alleles (J and j) with distinct sets of recombination rates, one higher and one lower. We allow for incomplete dominance of the modifier on recombination (see Table 1). A numerical analysis for the invasion conditions of a modifier of recombination is presented for the haploid three-locus case (Model 2) in the results section.

**Table 1.** The gene order assumed for the haploid and diploid selection models with a modifier of recombination (Models 3 and 4).

*Recombination modifier genotype*	*Intergenic recombination rate 1*	*Transposon locus*	*Intergenic recombination rate 2*	*(Epi)genetic locus*
J/J	r_1_	t or T	r_2_	A or B
J/j	r_3_	t or T	r_4_	A or B
j/j	r_5_	t or T	r_6_	A or B

Intergenic regions are highlighted in gray. Recombination rates can take on distinct values given the modifier genotype and location of recombination. States at the transposon and epigenetic locus were assumed not to impact the rate of recombination.

All models allow for natural selection, whereby the transposon is neutral or deleterious and the epiallele is deleterious. Natural selection acts at either the haploid level (main text) or diploid level (Supplementary File 3). Table 2 summarizes variables and parameters for all models. Table 3 summarizes genotype fitnesses across all models. Supplementary Files 1 and 2 provide an overview of the derivation for the recursive haploid (main text) and diploid models, respectively. Supplementary File 3 presents a summary of the diploid results. Supplementary Files 4 and 5 provide detailed derivations and calculations related to the equilibria and local stability results for the haploid and diploid settings, respectively, and which employ either Taylor approximations or numerical analysis. A systematic evaluation of the accuracy of the analytical approximations compared to numerical solutions for biologically valid parameter ranges is provided in Supplementary File 1 for Model 1, Section C (Case A Supplementary Figs. 1–7, Case B Supplementary Figs. 8–15, Supplementary File 1). Supplementary File 6 provides the Python code used to perform the numerical simulations for Model 2.

**Table 2.** Summary of variables and parameters for all models.

	Symbol
**Variables**	
Frequency of wild-type allele	p_A_
Frequency of epiallele	p_B_
Frequency of no transposon present	p_t_
Frequency of transposon	p_T_
**Parameters**	
Forward epimutation rate without transposon	*α*_1_
Reverse epimutation rate without transposon	*β*_1_
Forward epimutation rate with transposon (in cis)	*α*_2_
Reverse epimutation rate with transposon (in cis)	*β*_2_
Relative fitness of genotype “i’	$w_{i}$
Probability of selfing	S
Transposition rate	z
Transposon deletion rate	y
Recombination rate	r
Selection coefficient of the epiallele	k
Selection coefficient of the transposon	L

**Table 3.** Selection on haploids fitness assumptions (Models 1 and 2).

Genotype	Case A	Case B
tA	1	1
tB	1–k	1–k
TA	1	1–L
TB	1–k	1–k–L

#### Overview of the recursive system for Model 1

Here, we track the frequency of the tA haplotype throughout the life cycle, as an example. The first event that occurs during the life cycle is the mating of haploid organisms. There are 4 transient diploid genotypes that contribute to the frequency of the tA haplotype:


$$p_{tA}^{\left(1\right)}=p_{tA,tA}^{\left(1\right)}+\frac{1}{2}p_{tA,tB}^{\left(1\right)}+\frac{1}{2}p_{tA,TA}^{\left(1\right)}+\frac{1}{2}p_{tA,TB}^{\left(1\right)}.$$

After mating and accounting for selfing, the frequency of the transient diploid tA, tA genotype is


$$\begin{array}{c} \;p_{tA,tA}^{\left(1\right)}=(1-S)(\;p_{tA}^{2}{\left(1-\alpha_{1}\right)}^{2}+2p_{tA}p_{tB}(1-\alpha_{1})\beta_{1}+p_{tB}^{2}\beta_{1}^{2})\\ +S(\;p_{tA}{\left(1-\alpha_{1}\right)}^{2}+p_{tB}\beta_{1}^{2}) \end{array}$$

When expanded, the term $(1-S)p_{tA}^{2}(1-\alpha_{1}{)}^{2}$ represents 2 haploid individuals (with genotype tA) pairing at random with probability (1−S) and neither undergoes forward epimutation. Other terms correspond to other parental haploid genotypes that, when mated, form the tA,tA genotype and account for epimutation as well as selfing.

The next step in the life cycle is recombination during the diploid phase. After recombination, the frequency of the tA haplotype is


$$p_{tA}^{\left(2\right)}=p_{tA,tA}^{\left(1\right)}+\frac{1}{2}p_{tA,tB}^{\left(1\right)}+\frac{1}{2}p_{tA,TA}^{\left(1\right)}+(1-r)\frac{1}{2}p_{tA,TB}^{\left(1\right)}+r\frac{1}{2}p_{tB,TA}^{\left(1\right)}$$

Here, the term on the far right (i.e. $r\frac{1}{2}p_{tB,TA}^{\left(1\right)}$) refers to when the transient tB,TA genotype undergoes recombination to generate the tA haplotype with probability *r*, recognizing that one-half of recombinants are tA.

Natural selection occurs next in the life cycle and acts on adult haploid organisms, where $w_{i}$ is the symbol for relative fitness and $\bar{w}$ is the mean fitness of the population. The relative fitness of the tA haplotype is $w_{1}$ and after natural selection, its frequency is


$$p_{tA}^{\left(3\right)}=p_{tA}^{\left(2\right)}w_{1}\left(\frac{1}{\bar{w}}\right)$$

where $\bar{w}=p_{tA}^{\left(2\right)}w_{1}+p_{tB}^{\left(2\right)}w_{2}+p_{TA}^{\left(2\right)}w_{3}+p_{TB}^{\left(2\right)}w_{4}$.

As the final event in the life cycle, transposition and deletion occur such that


$$p_{tA}^{\left(4\right)}=p_{tA}^{\left(3\right)}(1-z)+p_{TA}^{\left(3\right)}y.$$

After plugging each event into the consecutive event and then simplifying, the final set of recursion equations for haplotype frequencies is derived. The full derivation and detailed calculations for the analysis are provided in Supplementary File 4. Model 2 follows the same life cycle, and an overview of the derivation is provided in Supplementary File 1, with the full derivation provided in Supplementary File 4.

### Approximation assumptions and analysis of equilibria

Due to the complexity of the equations, a subset of parameters was assumed to be small in magnitude (*O*(*ζ*)), based on empirical evidence, and Taylor approximations of equilibria were calculated. Specifically, the epiallele reversion rate adjacent to the transposon (*β*_2_), the forward epimutation rate adjacent to a site without a transposon (*α*_1_), the transposition rate (*z*), and the transposon deletion rate (*y*) were assumed to be small in magnitude for all models. These are reasonable biological assumptions as forward rates of spontaneous epimutation near transposons can be orders of magnitude greater than reverse rates (∼ 5 × 10^−4^ (*α*_2_) and 8 × 10^−6^ (*β*_2_)) whereas the opposite is expected when a transposon is not present (∼ 3 × 10^−5^ (*α*_1_) and 1.4 × 10^−3^ (*β*_1_), van der Graaf et al. 2015). As the assumptions for epimutation rates are based on studies in plants, the results are most relevant to the kingdom Plantae. Based on empirical evidence, it is also reasonable to assume the transposition/deletion rates are relatively small in magnitude (Tenaillon et al. 2010). The rate of selfing (*S*), the recombination rate (*r*), selection against the epiallele (*k*), selection against the transposon (L, Model 1, Case B), the background reversion rate (*β*_1_), and the forward epimutation rate adjacent to a transposon (*α*_2_) were not assumed to be constrained in magnitude.

After calculating equilibria, those that were always expected to have frequencies below zero and/or above one for small values of *β*_2_, *α*_1_, *z*, *y*, and across the range of other parameters were omitted from further analysis since they are biologically invalid. The remaining equilibria were then assessed for stability. We used both numerical calculations of approximate eigenvalues and numerical simulations of the recursion equations to determine the stability of a given equilibrium. For the eigenvalue approach, a given parameter was varied across an empirically reasonable range and all other parameters were held constant (see Supplementary File 4). Eigenvalues with absolute values less than one indicate local stability. For the simulations, local stability is indicated by reaching the numerical equilibrium values of the frequencies after starting above and below the equilibrium. Using this approach, only one biologically relevant equilibrium arises for Cases A and B. For more details on the technical approaches, see Supplementary File 1, and for the detailed calculations of the equilibria and eigenvalues see Supplementary File 4.

All mathematical analysis and simulations were performed using Wolfram Mathematica version 12.3.1 and Python version 3.8.5.

## Results

### Model 1: two-loci with haploid selection ((epi)mutation selection balance)

For this model, we assume the organism is haploid throughout most of its life cycle, other than a transient diploid phase during which recombination can occur. This context allows for obtaining baseline expectations of epi-mutation selection balance and, in particular, the effect of recombination on this balance. We consider two cases, one in which the transposon itself has a neutral direct effect on fitness (Case A) and one where it is directly deleterious (Case B). The assumed sequence of events for this model's life cycle is spontaneous epimutation (1), mating (2), recombination (3), selection on haploid genotypes (4), and transposition/deletion (5).

#### Case A: the epiallele is deleterious and the transposon is neutral

This case considers deleterious epimutation-selection balance where there is a fitness-neutral transposon that increases the forward rate of spontaneous epimutation of an adjacent genetic locus. For analytical tractability, we assume reverse epimutation near a transposon (*β*_2_), forward epimutation without a transposon (*α*_1_), and the transposition (z) and transposon deletion rate (*y*) are all small in magnitude (i.e. ∼ O[ *ζ* ]).

One locally stable and biologically valid equilibrium occurs (eq. 1) whereby the epiallele and transposon are at a low frequency. The transposon is at low frequency due to it being disproportionately associated with the epiallele and indirect selection.


(1) $$\begin{array}{r} \begin{array}{c} {\hat{\;p}}_{B}\approx \;(1-k)\left(\frac{r(1-S)(z-k\alpha_{1})+\alpha_{1}k}{k(1-r(1-S))(k+\beta_{1}(1-k))}+\frac{z}{k}\right)+O[\zeta^{2}]\\ {\hat{\;p}}_{T}\approx \frac{z}{k}\left(1-k+\frac{k+r(1-S)(1-k)}{\alpha_{2}(1-r(1-S))}\right)+O[\zeta^{2}] \end{array} \end{array}$$

**Fig. 1.**
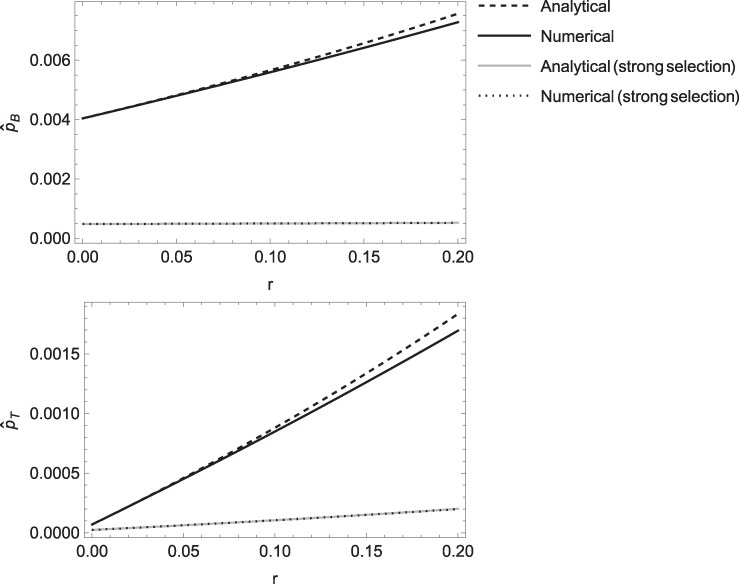
The equilibrium frequencies of the epiallele (top) and transposon (bottom) as the recombination rate varies and with different magnitudes of selection against the epiallele (*k* = 0.002 and *k* = 0.02, strong). The other parameter values are: *α*_1_ = 10^−5^, *α*_2_ = 0.005, *β*_1_ = 0.0005, *β*_2_ = 10^−5^, *z* = 10^−7^, *y* = 5 × 10^−6^, *S* = 0.25. “Analytical” numerical values above are based on inputting numerical values into eq. 1 and the numerical results are generated by simulations of the recursion equations. Note: as significant physical linkage between the transposon site and epigenetic site is anticipated, the recombination rate is likely to be smaller such that values over 0.1 where the approximation begins to break down (when selection is weaker) is unlikely.

A key result is that recombination will increase the epiallele frequency at this equilibrium (see Fig. 1, top panel for a numerical example). Recombination increases the effective rate of forward epimutation by breaking up the linkage between transposons and the epialleles they are associated with, allowing for more wild-type alleles to undergo epimutation. Selection against the epiallele (k) reduces epiallele, as well as transposon frequencies at equilibrium (see Supplementary File 1, Model 1, Case A, Supplementary Fig. 1 for a numerical example). Also, the forward epimutation when the transposon is absent (i.e. *α*_1_) always increases the epiallele frequency at equilibrium (see Supplementary File 1, Model 1, Case A, Supplementary Fig. 2, for a numerical example). Separately, selection (k) decreases the contribution of the epiallele reversion rate (see term $(1-k)\beta_{1}$) suggesting an interaction between these processes, such that they are not fully additive in terms of decreasing the epiallele equilibrium frequency. Unexpectedly, the epiallele itself was not impacted by *α*_2_ at equilibrium, potentially due to *α*_2_ having the same magnitude of effect in the numerator and denominator (not shown).

Next, considering the transposon, recombination causes an increase in its frequency at equilibrium (in eq. 1 and also see Fig. 1, bottom panel). This is due to recombination decoupling transposons from the deleterious epialleles that they are more often associated with, allowing them to pair more often with the fitter wild-type alleles. As the rate of epimutation adjacent to the transposon increases (*α*_2_), the transposon reaches a lower frequency due to more pairings with the deleterious epiallele.

With regards to selfing, it reduces the effective recombination rate (i.e. r(1−S)). At a high level, these results are in line with established effects of inbreeding (within classic frameworks), causing decreased deleterious variation at equilibria (Crow and Kimura 1970), but through recombination suppression (with the haploid setting), whereas in the classic framework (diploid setting) deleterious variation is reduced by inbreeding increasing the efficiency of selection (Crow and Kimura 1970).

#### Case B: the epiallele and transposon are deleterious

We next consider the possibility that the transposon is directly deleterious. Similar to the previous case, for analytical tractability, we assume the forward epimutation rate (*α*_1_), the reverse rate (*β*_2_), and the transposition/deletion rates (*z* and *y*) are small in magnitude (i.e. ∼ O[ *ζ* ]). We identify one locally stable and biologically valid equilibrium (eq. 2).


$$\begin{array}{r} {\hat{\;p}}_{B}\approx \frac{\alpha_{1}w_{t,B}(1-(1-\alpha_{2})w_{T,A})(1-w_{T,B})}{X_{2}}+\frac{z\alpha_{2}w_{T,B}}{X_{1}}+\frac{r(1-S)(\alpha_{1}w_{t,B}(\left(1-w_{T,A})w_{T,B}-\alpha_{2}w_{T,A}(1-w_{T,B}\right))+z\alpha_{2}(w_{t,B}(1+(1-\beta_{1})w_{T,B})-w_{T,B}))}{X_{2}}\;+\;O\left[\zeta^{2}\right] \end{array}$$

$${\hat{\;p}}_{T}\approx \frac{z(1-(1-r(1-S))(1-\alpha_{2})w_{T,B})}{X_{1}}+O[\zeta^{2}]$$

where


(2) $$\begin{array}{rl} & X_{1}=(1-(1-\alpha_{2})w_{T,A})(1-w_{T,B})+r(1-S)((1-w_{T,A})w_{T,B}\\ & \;-\;\alpha_{2}w_{T,A}(1-w_{T,B}))\\ & X_{2}=(1-(1-\beta_{1})w_{t,B})X_{1} \end{array}$$

Relative fitness symbols are used for clarity of presentation, where $w_{t,B}=1-k$, $w_{T,A}=1-L$, and $w_{T,B}=1-k-L$.

Considering ${\hat{\;p}}_{B}$, the first term indicates an increase in ${\hat{\;p}}_{B}$ through the background epimutation rate (*α*_1_), which is balanced by a combination of selection, recombination, and the background reversion rate. The second term indicates an increase in ${\hat{\;p}}_{B}$ through transposition and the cis epimutation rate due to the presence of the transposon (*α*_2_), which is balanced by selection, recombination, and the background reversion rate. The third term indicates interactions between recombination, epimutation, and transposition. Here, *α*_2_ has opposing effects. In particular, as *α*_2_ increases, this also reduces the increase in the background effective epimutation rate (*α*_1_).

Overall, a key result is that recombination can increase or decrease the epiallele frequency at equilibrium, in contrast to the previous case (when the transposon was neutral). To better understand the parameter interactions that cause recombination to have a different qualitative effect on the epiallele equilibrium frequency, a Taylor approximation of eq. 2 was calculated (around the point *r* = 0). This resulted in a zeroth-order term analogous to single locus (epi)mutation-selection balance and a first-order term representing a perturbation due to recombination. Then, setting each parameter (*k*, L, *α*_2_, and *β*_1_) to be proportional to *ζ* in this first order term for *r*, and using a Taylor series around the point (ζ=0) and including up to second order terms resulted in the condition


$$\alpha_{2}>\frac{L(\beta_{1}-L)}{k+L}$$

for when an increase in *r* increases ${\hat{\;p}}_{B}$. This relationship indicates that there is a tradeoff between indirect selection due to the transposon and the background reversion rate without a transposon with respect to the net effect of recombination on the epiallele frequency. For example, if $L>\beta_{1}$, recombination always increases the epiallele because indirect selection is larger in magnitude than the background reversion rate (i.e. there is a benefit for the epiallele by decoupling it from the transposon). If $L=\beta_{1}$, any non-zero *α*_2_ value also causes recombination to increase the epiallele frequency because indirect selection and the background reversion rate “cancel” out. If $L<\beta_{1}$, then specific parameter magnitudes determine the net effect of recombination. Of note, for Case A, by assumption L=0 (i.e. no capacity for indirect selection against the epiallele), suggesting why in Case A, recombination only increases the epiallele frequency based on the above relationship, since *α*_2_ > 0 always holds. More generally, whether the effective reversion rate of the epiallele is larger than the combined effects of the forward epimutation rate and selection is a key relationship.

**Fig. 2.**
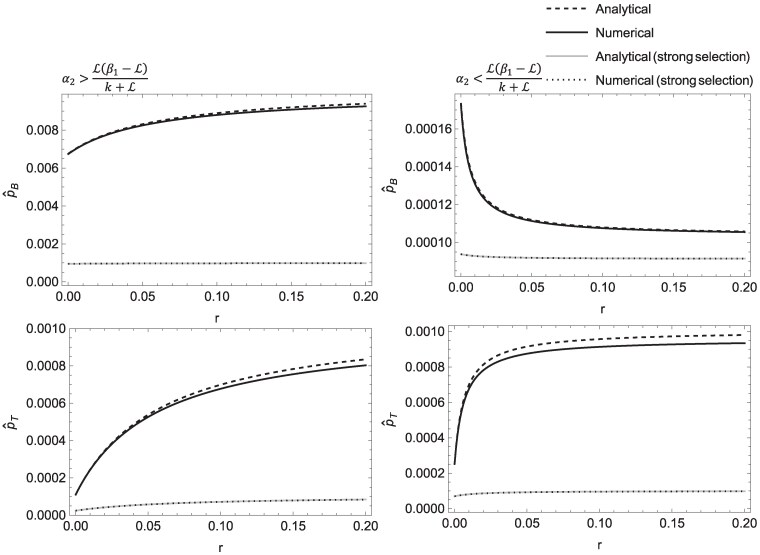
The equilibrium frequencies of the epiallele (top) and transposon (bottom) as the recombination rate varies and with different magnitudes of selection (*k* = 0.001, L=0.0001 and *k* = 0.01, L=0.001 strong). For the left panels *α*_2_ = 0.005 > *β*_1_ = 0.0005 and for the right panels *α*_2_ = 0.0005 < *β*_1_ = 0.1. The other parameter values are: *α*_1_ = 10^-5^, *β*_2_ = 10^-5^, z = 10^−7^, y = 5 × 10^−6^, S = 0.01. “Analytical” numerical values above are based on inputting numerical values into eq. 2 and the numerical results are generated by simulations of the recursion equations.

Figure 2 presents numerical results for this parameter relationship where the left panels are when $\alpha_{2}>\frac{L(\beta_{1}-L)}{k+L}$ such that recombination increases the epiallele equilibrium frequency and the right panels are when $\alpha_{2}<\frac{L(\beta_{1}-L)}{k+L}$ and recombination decreases the epiallele equilibrium frequency. Since *β*_1_ plays a key role in changing the qualitative effects of recombination, this suggests the degree of epigenetic resetting in the absence of a modifier (such as a transposon) for a given population/species (which can be quite variable) would have important implications on how recombination would impact the degree of segregating deleterious epigenetic variation (see Discussion for details). Separately, variation in mating systems may effect levels of selfing, which may increase or decrease deleterious epigenetic variation for a given population through its indirect effects on recombination.

Considering ${\hat{\;p}}_{T}$, it is determined by a balance of recombination, selection, transposition, and epimutation associated with the transposon (*α*_2_). Specifically, in the numerator, the effect of recombination is to increase ${\hat{\;p}}_{T}$, likely due to decoupling of the deleterious epiallele adjacent to the transposon, increasing its marginal fitness. As w_TB_ increases, this likely reduces the benefit of recombination for the transposon because the decoupling is less beneficial for the transposon. Whereas when w_TA_ increases (see denominator), the benefit of recombination increases, as TA haplotypes are generated after recombination, raising the marginal fitness of the transposon and simultaneously decreasing the marginal fitness of the absence of transposon (‘*t*’) site by generating tB haplotypes at the expense of tA haplotypes (where w_tA_ > w_tB_ by assumption). After further analysis, a first-order Taylor approximation (around the point *r* = 0) on ${\hat{\;p}}_{T}$ (from eq. 2) suggests the net effect of recombination is to always increase ${\hat{\;p}}_{T}$ (analytical approximation not shown; for a numerical example, see Fig. 2 bottom panels). As selfing reduces the effective recombination rate, it is anticipated to decrease ${\hat{\;p}}_{T}$, suggesting a potential benefit to populations with higher selfing rates.

### Model 2: three loci with haploid selection (a modifier of recombination)

**Fig. 3.**
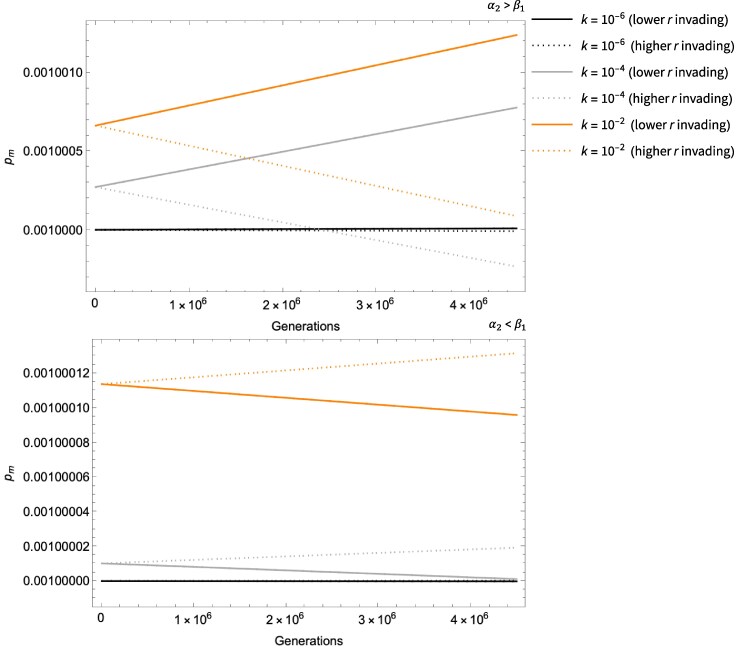
Frequency of a modifier of recombination (*p*_m_) is plotted as a function of time in generations. Solid lines are when a low recombination modifier is being introduced, whereas dotted lines are when a higher recombination rate modifier is being introduced. The low recombination modifier causes a recombination rate (between the transposon and epigenetic locus) of 0.00099 when homozygous and 0.000995 when heterozygous compared to the higher recombination modifier, which causes a rate of 0.001 when homozygous. The recombination rate between the modifier of recombination and the transposon locus is 0.05. For the top panel, *α*_2_ = 0.005 and *β*_1_ = 0.001 and for the bottom panel *α*_2_ = 0.001 and *β*_1_ = 0.005. Other parameter values are *α*_1_ = 10^−5^, *β*_2_ = 10^−5^, z = 10^−6^, y = 10^−4^.

This model is identical to Model 1 in terms of the life cycle but now includes a third locus, which modifies the recombination rate. The results from Model 1 indicate that recombination can increase deleterious epigenetic variation. This implies a third locus with a modifier of recombination should select for a lower recombination rate to help attenuate this deleterious process. We investigated numerically whether a modifier with a lower recombination rate can invade at an equilibrium where a higher recombination rate is fixed. In line with expectations, a modifier for recombination that lowers the recombination rate is able to invade. We plot the frequency of the invading modifier (*p*_m_) as a function of time (Fig. 3). A key relationship impacting whether the modifier increases or decreases in frequency is the relative magnitudes of the forward epimutation rate (*α*_2_) and the background epiallele reversion rate (*β*_1_). Roughly, when *α*_2_ > *β*_1_ (Fig. 3, top panel), invasion of a low recombination modifier occurs (solid lines), whereas a higher recombination rate invades for the reverse scenario (i.e. *α*_2_ < *β*_1_, Fig. 3, bottom panel). This suggests the importance of the indirect effects of recombination on the effective epimutation rate to allow for invasion of a lower recombination rate. The strength of selection against the epiallele did not have a significant effect on the invasion conditions; however, it does increase the speed of invasion or purging of the modifier (comparing slopes of gray to orange lines to black lines). Overall, since the invading modifier is of weak effect (1% decreased recombination rate when homozygous and 0.5% when heterozygous), it is anticipated any larger reduction in the recombination rate will invade, as well. A modifier of a larger effect was evaluated and invasion occurs at a faster rate (data not shown).

A more extensive evaluation of the parameter conditions that allow for invasion of a recombination modifier is presented in Supplementary File 1, Model 2, section C, Supplementary Figs. 16–21. Of note, stronger linkage between the modifier and transposon locus increases the speed of invasion (Supplementary File 1, Model 2, Supplementary Fig. 16 compared to Supplementary Fig. 17) likely through more efficient indirect selection for (or against) the modifier. Whereas weaker linkage between the transposon and epigenetic locus increases the speed of invasion of the modifier (Supplementary File 1, Model 2, Supplementary Fig. 20). This is likely due to a stronger indirect effect of recombination on the effective epimutation rate, leading to larger differences in marginal fitness between the invading vs wild-type recombination modifier. Generally, we find the interaction between recombination, *α*_2_, and *β*_1_ play a key role in the invasion conditions for the parameter conditions investigated. Additionally, selfing did not appear to qualitatively affect the conditions for invasion and had marginal effects on the speed of invasion (data not shown).

Overall, we find key findings from the haploid models appear to hold in the diploid setting, suggesting broader applicability of the results (see Supplementary File 3).

## Discussion

### Comparison of the results to previous theoretical work on the evolution of recombination

Recombination is a ubiquitous and fundamental evolutionary process, and much theory has sought to identify the conditions that favor its maintenance. Increased recombination is predicted to be favored under several scenarios: (1) when there is synergistic epistasis for the loci under selection (Altenberg et al. 2017), (2) when there is negative epistasis and tight linkage (Otto and Feldman 1997), (3) when it reduces selective interference when there are multiple loci and deleterious mutations (Keightley and Otto 2006; “Hill Robertson effect”, Hill and Robertson 1966), (4) during adaptation to a new environment, particularly with continual background deleterious mutations (Hartfield et al. 2010), and (5) when distinct individuals in a population carry adaptive mutations and recombination incorporates them together, accelerating adaptation (Felsenstein 1974). In contrast, an increased recombination rate is expected to be maladaptive when there are positive associations between favorable alleles (Barton 2009) and a reduction in the recombination rate is expected to be favored when a population is close to an equilibrium where there is a balance of selection and a variation generating process with a constant environment (Altenberg et al. 2017).

Further, a recent study that accounts for stochasticity and selective interference (Stetsenko and Roze 2022) indicates intermediate degrees of selfing can lead to the evolution of increased or decreased recombination. Separately, the authors indicate that in a deterministic setting (such as in our study), without epistasis and with partial selfing, recombination is predicted to be selected against due to breaking up positive associations of deleterious alleles, which interferes with purging (Stetsenko and Roze 2022). Overall, in the context of transposons, theory indicates recombination may be favored or disfavored based on the number of TEs per genome, the cost of recombination (such as due to ectopic recombination) and the strength of drift (Roze 2023). Also, of note, multiple recombination rates may be favored genomically, as suggested by Parée et al. (2025), who considered recombination as a more local process and perhaps leads to conflicting selection (Parée et al. 2025).

Our study examines the evolution of recombination in the context of deleterious epimutation and transposons, which lead to potentially novel mechanisms that may shape the evolution of recombination. When transposons are neutral and increase the cis epimutation rate, recombination always increases the effective epimutation rate at equilibrium. This increases deleterious epiallele and transposon frequencies (eq. 1), and selection is then predicted to lower recombination rates. The mechanism is distinct from other mechanisms that are predicted to lower recombination rates, such as interference during purging of deleterious alleles or the generation of haplotype variation when a population is near an equilibrium. Selfing reduces the effective recombination rate, such that selection for increased selfing would reduce deleterious variation in our setting, suggesting the importance of breeding systems.

When a transposon is deleterious and increases the cis epimutation rate, the implications for recombination evolution were found to qualitatively depend on key parameter interactions, making it distinct from the neutral setting. The interaction of these parameters could be a novel factor to consider in terms of transposon impacts on the *direction* of recombination rate evolution in addition to established factors (ex. TE copy number, genetic drift, ectopic recombination, Roze 2023). Specifically, the strength of selection, the cis epimutation rate due to the transposon, and the background epiallele reversion rate were key parameters qualitatively determining the indirect fitness effects of recombination (eq. 2). Roughly, when the background epiallele reversion rate is greater in magnitude than selection and the forward rate due to the presence of a transposon, recombination is beneficial because it decreases deleterious epiallele frequencies (and vice versa). Importantly, this relates to the unique biology of epialleles vs genetic alleles because epialleles tend to revert at much higher rates and there is more clade variation in the reversion rate (ex. plants vs mammals, Charlesworth et al. 2017). Selection then may increase or decrease recombination rates depending on the net stability of epialleles, which could cause divergence in recombination rate evolution between clades.

As a further consideration (relevant to both neutral and deleterious transposons), transposon heterogeneity exists between clades and within populations (Dubin et al. 2018). This could result in more epigenomic variation across individuals and populations, potentially influencing local recombination rate evolution in addition to more global impacts.

### Comparison of results to empirical data and predictions on the evolution of recombination rates

Empirical work indicates transposons are often present in low-recombining genomic regions. However, at a more local scale, there are examples where transposons can be associated with higher or lower recombination rates (Kent et al. 2017). In the context of epigenetics, some evidence indicates the degree of silencing of TEs is a factor that interacts with recombination rates and fitness effects at a local scale (Hollister and Gaut 2009; Lee and Chwen 2015; Kent et al. 2017).

Our finding that recombination can be indirectly deleterious due to cis epigenetic effects of transposons aligns with the general pattern of transposons being present in low recombining regions that also tend to be gene poor (Kent et al. 2017). However, the location of transposons could also be due to selection against the direct negative effects of transposons, causing deleterious mutations when they insert near or in genes.

In terms of predictions that may motivate empirical research, our work suggests that when transposon insertion is neutral, deleterious epialleles and nearby transposons should be at higher frequencies in more outbred populations vs more inbred/selfing populations and further increased when epigenetic resetting rates are low (provided modifiers have not significantly decreased recombination rates). The reason is with outbreeding, there is a higher effective recombination rate resulting in a higher effective epimutation rate. In contrast, when a transposon insertion near a gene is deleterious, predictions of equilibrium epiallele frequencies are more nuanced and depend on the relative balance of the forward rate caused by a transposon, the background resetting rate, and the strength of selection for that given locus. For example, when epigenetic resetting rates are high, as in mammals (Charlesworth et al. 2017), deleterious epialleles may be at higher frequencies in more inbred populations. This is due to a lower effective recombination rate, lowering the effective epiallele reversion rate.

Ideally, to evaluate the predictions, comparison of transgenerational methylome data sets of sister species with distinct breeding systems and/or distinct epigenetic resetting rates would be performed. Roughly, the results are expected to correspond to transposon families that actively insert near genetic loci (within a few kbs) and could be used to predict, for example, methylated nucleotide frequencies and their fitness consequences on populations. As large methylome data sets are increasing, this should be feasible.

### Implications of the results on the host population/transposon relationship

A further consideration is the impact of deleterious epigenetic effects on the host–transposon relationship. Transposons are often considered molecular parasites that are largely deleterious (when non-neutral), although they can be co-opted for adaptive uses (Colonna Romano and Fanti 2022) or possibly even act as a general source of adaptive variation (for a review see Baduel and Quadrana 2021). From the perspective that transposons are “selfish agents”, they may have deleterious impacts on host fitness either directly through mutation or indirectly through secondary epimutation due to their regulation (Choi and Lee 2020). However, since transposons mainly transmit vertically, they may evolve to be less deleterious (Hosaka and Kakutani 2018), which can be mutually beneficial for host and parasite. One major strategy to reduce deleterious effects is for transposons to evolve an insertional bias to avoid deleterious mutations in genes and regulatory sequences. With consideration of the potential additional deleterious epigenetic effects, this could cause further selection for insertional bias even further away from genes and regulator sequences to reduce transposon-induced epimutation, and some evidence of this exists (Choi and Lee 2020).

Some of the results presented in this paper suggest an additional evolutionary conflict between host and transposon. Essentially, selection on the host population can favor reduced recombination because this reduces deleterious epigenetic variation. In contrast, transposons benefit from increased recombination because they are often associated with a deleterious epiallele, such that recombination decouples them from the epiallele, which increases their frequency. This could indicate the type of population or species mating system that a particular transposon strain could invade more easily by giving it an advantage based on the effective recombination rate. For example, a transposon would likely more easily invade a more outbreeding population relative to a more selfing population when its direct fitness effects are neutral (or near neutral). At a high level, this could be viewed as a deleterious byproduct of an outbreeding strategy (despite the established benefits of such a strategy) due to the existence of transposons and their secondary epigenetic impacts.

### Future directions and conclusion

The current model allows for distinct backward and forward epimutation rates, with and without the transposon; however, one limitation is that transposon copy number or dosage effects in relation to the probability of epimutation are not considered. This might be particularly interesting to consider in models that allow for some possibility of adaptive epialleles or epigenetically affected mutation rates leading to adaptive alleles. This may imply that transposons (or similar genetic elements) could be utilized by selection to generate distinct local (epi)mutation rates, with increased precision based on copy number variation, for example.

As a separate consideration, genetic drift was not included in the models. Although the mechanism of recombination breaking up linkage between epialleles and transposons is still expected to occur, drift may reduce the effective recombination rate as newly formed transposon and epiallele haplotypes may often be lost before recombination events. This would be particularly important when considering the net effect of transposon-associated epialleles at the genomic scale (likely reducing their impacts on fitness and recombination evolution), along with the co-evolution of mating systems with these dynamics. Importantly, however, drift could also increase the chance of fixation of transposons (Kent et al. 2017) and associated epialleles.

We did not consider paramutation in our models. Paramutation relates to the conversion of the gene expression pattern of one gene by another through an in trans interaction (Geoghegan and Spencer 2013b). Paramutation is currently a rare phenomenon, but some studies suggest it may be more common than currently thought (Springer and McGinnis 2015). Paramutation could have interesting interactions since it can depend on transposons in order to occur and selfing/inbreeding is expected to reduce the effective paramutation rate (Springer and McGinnis 2015). Paramutation could exacerbate the deleterious effects of transposon-associated epialleles in populations and further lead to selection for selfing to mitigate such effects, for example.

Overall, this study characterizes multi-locus epimutation-selection balance and has implications for microevolutionary interactions between genetic background and segregating deleterious epigenetic variation and the evolution of recombination. Our models focus on transposons specifically, due to their ubiquitous presence in nature and their genomic consequences; however, they are not the only known genetic element that can act as a modifier of epimutation. For example, repetitive elements are another type of element that are associated with epimutations (Silveira et al. 2013). Therefore, this study's results could offer a baseline understanding and predictions for epialleles associated with transposons, repetitive elements, and possibly future modifiers of epimutation that have not been discovered.

## Supplementary Material

jkag147_Supplementary_Data

## Data Availability

The authors affirm that all data necessary for confirming the conclusions of the article are present within the article, figures, tables, and Supplementary material. Supplemental material available at G3 online.

## References

[jkag147-B1] Altenberg L, Liberman U, Feldman MW. 2017. Unified reduction principle for the evolution of mutation, migration, and recombination. Proc Natl Acad Sci U S A. 114:E2392–E2400. 10.1073/pnas.1619655114. 28265103 PMC5373362

[jkag147-B2] Baduel P, Quadrana L. 2021. Jumpstarting evolution: How transposition can facilitate adaptation to rapid environmental changes. Curr Opin Plant Biol. 61:102043. 10.1016/j.pbi.2021.102043. 33932785

[jkag147-B3] Barrett SCH . 2013. IV.8. Evolution of mating systems: outcrossing versus selfing. In: Losos JB et al., editors. The Princeton guide to evolution. Princeton University Press. pp. 356–362. 10.1515/9781400848065-050.

[jkag147-B4] Barton NH . 2009. Why sex and recombination? Cold Spring Harb Symp Quant Biol. 74:187–195. 10.1101/sqb.2009.74.030. 19903748

[jkag147-B5] Bonduriansky R, Day T. 2018. Extended heredity: a new understanding of inheritance and evolution. Princeton University Press.

[jkag147-B6] Branciamore S, Rodin AS, Gogoshin G, Riggs AD. 2015. Epigenetics and evolution: transposons and the stochastic epigenetic modification model. AIMS Genet. 02:148–162. 10.3934/genet.2015.2.148.

[jkag147-B7] Charlesworth D . 2006. Evolution of plant breeding systems. Curr Biol. 16:R726–R735. 10.1016/j.cub.2006.07.068. 16950099

[jkag147-B8] Charlesworth D, Barton NH, Charlesworth B. 2017. The sources of adaptive variation. Proc Biol Sci. 284:20162864. 10.1098/rspb.2016.2864. 28566483 PMC5454256

[jkag147-B9] Chernomas G, Griswold CK. 2024. Deleterious mutation/epimutation–selection balance with and without inbreeding: a population (epi)genetics model. Genetics. 227:iyae080. 10.1093/genetics/iyae080. 38733620 PMC11228854

[jkag147-B10] Choi JY, Lee YCG. 2020. Double-edged sword: the evolutionary consequences of the epigenetic silencing of transposable elements. PLoS Genet. 16:e1008872. 10.1371/journal.pgen.1008872. 32673310 PMC7365398

[jkag147-B11] Colonna Romano N, Fanti L. 2022. Transposable elements: major players in shaping genomic and evolutionary patterns. Cells. 11:1048. 10.3390/cells11061048. 35326499 PMC8947103

[jkag147-B12] Crow JF, Kimura M. 1970. An Introduction to population genetics theory. Indian ed. Scientific Publisher (India); The Blackburn Press.

[jkag147-B13] Dubin MJ, Scheid OM, Becker C. 2018. Transposons: a blessing curse. Curr Opin Plant Biol. 42:23–29. 10.1016/j.pbi.2018.01.003. 29453028

[jkag147-B14] Felsenstein J . 1974. The evolutionary advantage of recombination. Genetics. 78:737–756. 10.1093/genetics/78.2.737. 4448362 PMC1213231

[jkag147-B15] Fouché S, Oggenfuss U, Chanclud E, Croll D. 2022. A Devil's bargain with transposable elements in plant pathogens. Trends Genet. 38:222–230. 10.1016/j.tig.2021.08.005. 34489138

[jkag147-B16] Furrow RE . 2014. Epigenetic inheritance, epimutation, and the response to selection. PLoS One. 9:e101559. 10.1371/journal.pone.0101559. 25019291 PMC4096402

[jkag147-B17] Geoghegan JL, Spencer HG. 2012. Population-epigenetic models of selection. Theor Popul Biol. 81:232–242. 10.1016/j.tpb.2011.08.001. 21855559

[jkag147-B18] Geoghegan JL, Spencer HG. 2013a. Exploring epiallele stability in a population-epigenetic model. Theor Popul Biol. 83:136–144. 10.1016/j.tpb.2012.09.001. 23044385

[jkag147-B19] Geoghegan JL, Spencer HG. 2013b. The evolutionary potential of paramutation: a population-epigenetic model. Theor Popul Biol. 88:9–19. 10.1016/j.tpb.2013.05.003. 23751219

[jkag147-B20] Hartfield M, Otto SP, Keightley PD. 2010. The role of advantageous mutations in enhancing the evolution of a recombination modifier. Genetics. 184:1153–1164. 10.1534/genetics.109.112920. 20139345 PMC2865915

[jkag147-B21] Hill WG, Robertson A. 1966. The effect of linkage on limits to artificial selection. Genet Res. 8:269–294. 10.1017/S0016672300010156. 5980116

[jkag147-B22] Hollister JD, Gaut BS. 2009. Epigenetic silencing of transposable elements: a trade-off between reduced transposition and deleterious effects on neighboring gene expression. Genome Res. 19:1419–1428. 10.1101/gr.091678.109. 19478138 PMC2720190

[jkag147-B23] Hosaka A, Kakutani T. 2018. Transposable elements, genome evolution and transgenerational epigenetic variation. Curr Opin Genet Dev. 49:43–48. 10.1016/j.gde.2018.02.012. 29525544

[jkag147-B24] Keightley PD, Otto SP. 2006. Interference among deleterious mutations favours sex and recombination in finite populations. Nature. 443:89–92. 10.1038/nature05049. 16957730

[jkag147-B25] Kent TV, Uzunović J, Wright SI. 2017. Coevolution between transposable elements and recombination. Philos Trans R Soc Lond B Biol Sci. 372:20160458. 10.1098/rstb.2016.0458. 29109221 PMC5698620

[jkag147-B26] Kondrashov AS . 1984. Deleterious mutations as an evolutionary factor: 1. The advantage of recombination. Genet Res. 44:199–217. 10.1017/S0016672300026392. 6510714

[jkag147-B27] Lee Y, Chwen G. 2015. The role of piRNA-mediated epigenetic silencing in the population dynamics of transposable elements in Drosophila melanogaster. PLoS Genet. 11:e1005269. 10.1371/journal.pgen.1005269. 26042931 PMC4456100

[jkag147-B28] Lenormand T, Otto SP. 2000. The evolution of recombination in a heterogeneous environment. Genetics. 156:423–438. 10.1093/genetics/156.1.423. 10978305 PMC1461255

[jkag147-B29] Lyons DB et al 2023. Extensive de novo activity stabilizes epigenetic inheritance of cg methylation in arabidopsis transposons. Cell Rep. 42:112132. 10.1016/j.celrep.2023.112132. 36827183

[jkag147-B30] Martin A et al 2009. A transposon-induced epigenetic change leads to sex determination in melon. Nature. 461:1135–1138. 10.1038/nature08498. 19847267

[jkag147-B31] Morgan HD, Sutherland HGE, Martin DIK, Whitelaw E. 1999. Epigenetic inheritance at the agouti locus in the mouse. Nat Genet. 23:314–318. 10.1038/15490. 10545949

[jkag147-B32] Otto SP, Feldman MW. 1997. Deleterious mutations, variable epistatic interactions, and the evolution of recombination. Theor Popul Biol. 51:134–147. 10.1006/tpbi.1997.1301. 9169238

[jkag147-B33] Parée T, Noble L, Roze D, Teotónio H. 2025. Selection can favor a recombination landscape that limits polygenic adaptation. Mol Biol Evol. 42:msae273. 10.1093/molbev/msae273. 39776196 PMC11739800

[jkag147-B34] Quadrana L et al 2014. Natural occurring epialleles determine vitamin e accumulation in tomato fruits. Nat Commun. 5:3027. 10.1038/ncomms5027. 24967512

[jkag147-B35] Roessler K, Bousios A, Meca E, Gaut BS. 2018. Modeling interactions between transposable elements and the plant epigenetic response: a surprising reliance on element retention. Genome Biol Evol. 10:803–815. 10.1093/gbe/evy043. 29608716 PMC5841382

[jkag147-B36] Roze D . 2023. Causes and consequences of linkage disequilibrium among transposable elements within eukaryotic genomes. Genetics. 224:iyad058. 10.1093/genetics/iyad058. 37019818

[jkag147-B37] Shah JM . 2022. Epimutations and mutations, nurturing phenotypic diversity. Genetica. 150:171–181. 10.1007/s10709-021-00124-8. 34114171

[jkag147-B38] Silveira AB et al 2013. Extensive natural epigenetic variation at a de novo originated gene. PLoS Genet. 9:e1003437. 10.1371/journal.pgen.1003437. 23593031 PMC3623765

[jkag147-B39] Springer NM, McGinnis KM. 2015. Paramutation in evolution, population genetics and breeding. Semin Cell Dev Biol. 44:33–38. 10.1016/j.semcdb.2015.08.010. 26325077

[jkag147-B40] Stenøien HK, Pedersen B. 2005. Mutation and epimutation load in haploid and diploid life forms. J Theor Biol. 233:119–126. 10.1016/j.jtbi.2004.09.012. 15615625

[jkag147-B41] Stetsenko R, Roze D. 2022. The evolution of recombination in self-fertilizing organisms. Genetics. 222:iyac114. 10.1093/genetics/iyac114. 35929790 PMC9434187

[jkag147-B42] Tenaillon MI, Hollister JD, Gaut BS. 2010. A triptych of the evolution of plant transposable elements. Trends Plant Sci. 15:471–478. 10.1016/j.tplants.2010.05.003. 20541961

[jkag147-B43] van der Graaf A et al 2015. Rate, spectrum, and evolutionary dynamics of spontaneous epimutations. Proc Natl Acad Sci U S A. 112:6676–6681. 10.1073/pnas.1424254112. 25964364 PMC4450394

[jkag147-B44] Webster AK . 2024. Heritable epigenetic variation facilitates long-term maintenance of epigenetic and genetic variation. G3 (Bethesda). 14:jkad287. 10.1093/g3journal/jkad287. 38113034 PMC10849368

