# Supplementary material for: Multi-locus models of deleterious epimutation-selection balance and the evolution of recombination modifiers
Source: G3 (Bethesda). 2026 Jun 11;16(8):jkag147. doi: 10.1093/g3journal/jkag147 (PMC13439953; doi:10.1093/g3journal/jkag147)
Supplement: Supplementary file 1 [file jkag147_supplementary_data.zip › File_S1_G3-2026-406777.pdf]

Overview of analytical methods

The analytical methods used (for models 1 and 3) are very similar to that of Chernomas and Griswold (2024). Two distinctions in comparison to the previous study are that there are three dimensions in the system of equations instead of two, and the variables that were explicitly tracked were haplotypes instead of (epi)allele frequencies. The haplotype frequencies that are explicitly tracked are p_{tB}, p_{TA}, and p_{TB}, (where ptA = 1 - p_{tB} - p_{TA} - p_{TB}) with a given recursion for each haplotype which is function of the variables and parameters (ex. ptB' = $f_1(p_{tB}, p_{TA}, p_{TB}; n_{1,1}, n_{1,2}, \ldots n_{1,x})$) when n_i is a general symbol for a parameter and 'x' is the number of parameters for the function (f_1, f_2, or f_3).

After setting the equilibrium conditions for the haplotype frequencies (ex. $\hat{p}_{tB} = f_1(\hat{p}_{tB}, \hat{p}_{TA}, \hat{p}_{TB}; n_{1,1}, n_{1,2}, \ldots n_{1,x})$), and assuming certain parameters were relatively small in magnitude, allowed for Taylor's approximated equilibrium solutions across all the cases for models 1 and 2 (see eq. 1 and 2 of the main text and eq. 1 and 2 in File S3). The methods for calculating the equilibria directly follow Chernomas and Griswold, 2024, with detailed calculations provided here in File S4 and S5. The local stability analysis also directly followed that of the previous study, with the only consideration being the added complexity of a third dimension to the system, which gives three eigenvalues instead of two for each equilibrium. Due to the complicated nature of the eigenvalues, they were assessed numerically, varying key parameters within empirically reasonable magnitudes (see File S4 and S5).

Note: All analysis was performed using Wolfram Mathematica, version 12.3.1.0 and numerical simulations for model 3 was performed using Python 3 within a Jupyter Notebook.

Model 1: Two locus haploid mating and haploid selection

A - Introduction

This model assumes organisms are haploid through most of the life cycle, and are only transiently diploid during the mating phase. Specifically, the life cycle consists of four events:

1 - 'Mating' of haploid organisms occurs, conditioned on epimutation occurring on both gametes (regardless of whether they pair randomly or through selfing).

2 - Recombination occurs prior to the next generation of haploid organisms being generated

3 - Selection occurs on haploid organisms

4 - Transposition/deletion occurs

Cycle repeats

Of note, for models 1, 2, and 4 , selfing is used to model inbreeding. Classic models of inbreeding often do not account for the possibility that there can be a change in state of both gametes that are identical by descent prior to mating. This is a biologically reasonable assumption as mutation rates tend to be very small. Some models have allowed for forward mutation to occur to both gametes prior to mating/selfing, (for example, see Uyenoyama and Waller, 1991) but ignore back mutation. As epiallele reversion rates can be high, these simplifying assumptions are likely not biological reasonable for epimutations. By using selfing instead of modelling inbreeding (using an inbreeding coefficient), we make explicit that the gametes are from the same individual prior to mating (with selfing) and we allow for both gametes to separately undergo a possible epimutational change prior to forming a zygote.

B - Overview of Model

To give more clarity for the derivation, we will track the frequency of the tA haplotype throughout the life cycle, as an example. The first event that occurs during the life cycle is the mating of haploid organisms, conditioned on epimutation. There are four transient diploid genotypes that contribute to the frequency of the tA haplotype:

$$p_{t,A}{}^{(1)} = p_{t,A,t,A}{}^{(1)} + \frac{1}{2}\, p_{t,A,t,B}{}^{(1)} + \frac{1}{2}\, p_{t,A,T,A}{}^{(1)} + \frac{1}{2}\, p_{t,A,T,B}{}^{(1)}$$

where

$$p_{t,A,t,A}{}^{(1)} = (1-S)\left(p_{t,A}\,(1-\alpha_1)\,p_{t,A}\,(1-\alpha_1) + p_{t,A}\,(1-\alpha_1)\,p_{t,B}\,\beta_1 + p_{t,B}\,\beta_1\,p_{t,A}\,(1-\alpha_1) + p_{t,B}\,\beta_1\,p_{t,B}\,\beta_1\right) + S\left(p_{t,A}\,(1-\alpha_1)\,(1-\alpha_1) + p_{t,B}\,\beta_1\,\beta_1\right)$$

$$p_{t,A,t,B}{}^{(1)} = (1-S)\left(p_{t,A}\,\alpha_1\,p_{t,A}\,(1-\alpha_1) + p_{t,A}\,\alpha_1\,p_{t,B}\,\beta_1 + p_{t,A}\,(1-\alpha_1)\,p_{t,A}\,\alpha_1 + p_{t,A}\,(1-\alpha_1)\,p_{t,B}\,(1-\beta_1) + p_{t,B}\,\beta_1\,p_{t,A}\,\alpha_1 + p_{t,B}\,\beta_1\,p_{t,B}\,(1-\beta_1) + p_{t,B}\,(1-\beta_1)\,p_{t,B}\,\beta_1 + p_{t,B}\,(1-\beta_1)\,p_{t,A}\,(1-\alpha_1)\right) + S\left(2\,p_{t,A}\,(1-\alpha_1)\,\alpha_1 + 2\,p_{t,B}\,(1-\beta_1)\,\beta_1\right)$$

$$p_{t,A,T,A}{}^{(1)} = (1-S)\,2\left(p_{t,A}\,(1-\alpha_1)\,p_{T,A}\,(1-\alpha_2) + p_{t,A}\,(1-\alpha_1)\,p_{T,B}\,\beta_2 + p_{t,B}\,\beta_1\,p_{T,A}\,(1-\alpha_2) + p_{t,B}\,\beta_1\,p_{T,B}\,\beta_2\right)$$

$$p_{t,A,T,B}{}^{(1)} = (1-S)\,2\left(p_{t,A}\,(1-\alpha_1)\,p_{T,A}\,\alpha_2 + p_{t,A}\,(1-\alpha_1)\,p_{T,B}\,(1-\beta_2) + p_{t,B}\,\beta_1\,p_{T,A}\,\alpha_2 + p_{t,B}\,\beta_1\,p_{T,B}\,(1-\beta_2)\right)$$

Specifically, for example, the first term at the top left in the first equation (i.e. $(1-S)\left(p_{t,A}\,(1-\alpha_1)\,p_{t,A}\,(1-\alpha_1)\right)$) represents two haploid individuals (with genotype tA) pairing at random with probability (1 - S) and neither undergoes forward epimutation $(1-\alpha_1)$.

The next step in the life cycle is recombination during the temporary diploid phase. After recombination, the frequency of the t-A haplotype is

$$p_{t,A}{}^{(2)} = p_{t,A,t,A}{}^{(1)} + \frac{1}{2} p_{t,A,t,B}{}^{(1)} + \frac{1}{2} p_{t,A,T,A}{}^{(1)} + (1-r) \frac{1}{2} p_{t,A,T,B}{}^{(1)} + r \frac{1}{2} p_{t,B,T,A}{}^{(1)}$$

For example, the term on the far right (i.e. $r \frac{1}{2} p_{t,B,T,A}{}^{(1)}$) refers to the tB and TA haplotypes being paired together, and recombination occurs with probability r. Half of this term gives tA haplotypes contributing to the overall frequency (and one half TB haplotypes).

As the next step in the life cycle, natural selection occurs on adult haploid organisms where w_i is the symbol for relative fitness and $\overline{w}$ is the mean fitness of the population. Following the main text, the relative fitness of the tA haplotype is w_1.

$$p_{t,A}{}^{(3)} = p_{t,A}{}^{(2)} w_1 \left(\frac{1}{\overline{w}}\right)$$

where $\overline{w} = p_{t,A}{}^{(2)} w_1 + p_{t,B}{}^{(2)} w_2 + p_{T,A}{}^{(2)} w_3 + p_{T,B}{}^{(2)} w_4$

As the final event in the life cycle, transposition and deletion occur within the haploid organisms.

$$p_{t,A}{}^{(4)} = p_{t,A}{}^{(3)} (1-z) + p_{T,A}{}^{(3)} y$$

There are only two possibilities that impact the frequency of the tA haplotype. The current frequency of the haplotype is not impacted by transposition with probability (1 - z) and the tA haplotype frequency is increased by the TA haplotype undergoing transposon deletions with probability y.

After plugging each event into the consecutive event and then simplifying, the final recursion equations for the haplotype frequencies are derived. The full derivation and equations are given in Supplement 2, under Model 1. Supplement 2, Model 1, case A and B, gives the detailed calculations for the analysis.

C - Figures

The set of figures for cases A and B below evaluate the accuracy of the analytical approximations (main text eq. 1 for case A and eq. 2 for case B) in comparison to numerical equilibrium solutions which were calculated by simulating the full recursion equations for each case. Parameters are evaluates across empirically reasonable ranges. Additionally, for both cases the approximated eigenvalues were evaluated over the same parameter ranges and always indicated local stability (see File S4 for the eigenvalues calculations).

Case A: The epiallele is deleterious and the transposon is neutral

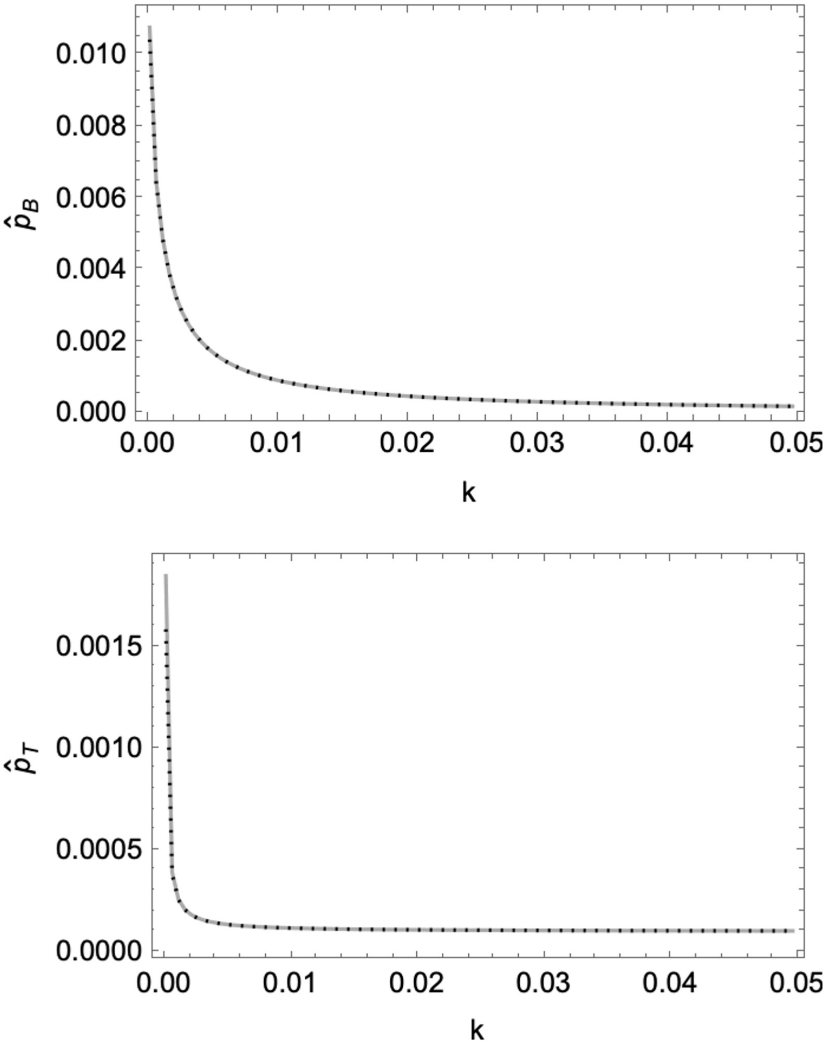

Figure S1: The equilibrium frequencies of the epiallele (top) and transposon (bottom) as selection against the epiallele varies. The gray line represents the analytical approximation and the black dotted line represents numerical simulations of the recursion equations. The other parameter values are: $\alpha_1 = 10^{-5}$, $\alpha_2 = 0.001$, $\beta_1 = 0.001$, $\beta_2 = 10^{-5}$, $z = 10^{-7}$, $y = 10^{-5}$, $S = 0.25$.

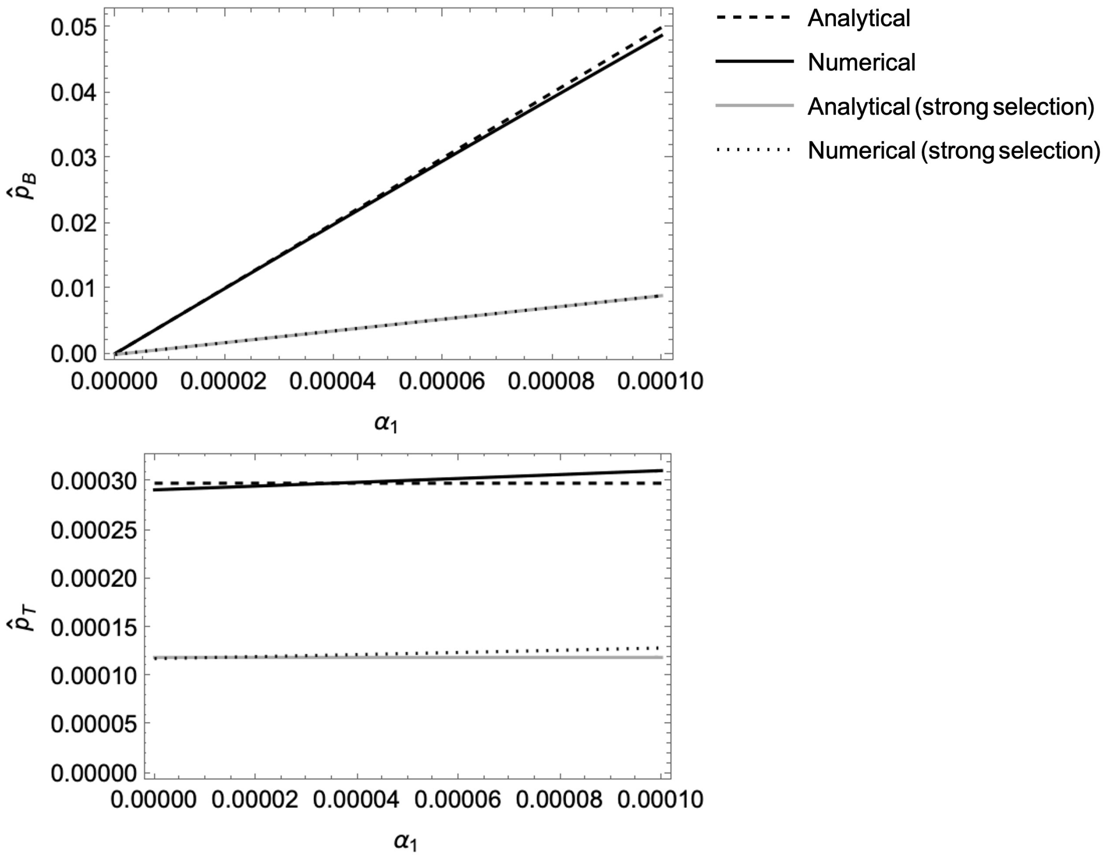

Figure S2: The equilibrium frequencies of the epiallele (top) and transposon (bottom) as the epimutation rate varies and with different magnitudes of selection against the epiallele (k = 0.001 and k = 0.01, strong). The other parameter values are: α_2= 0.001, β_1= 0.001 , $\beta_2 = 10^{-5}$,
z = 10^{-7}, y = 10^{-5}, S = 0.01.

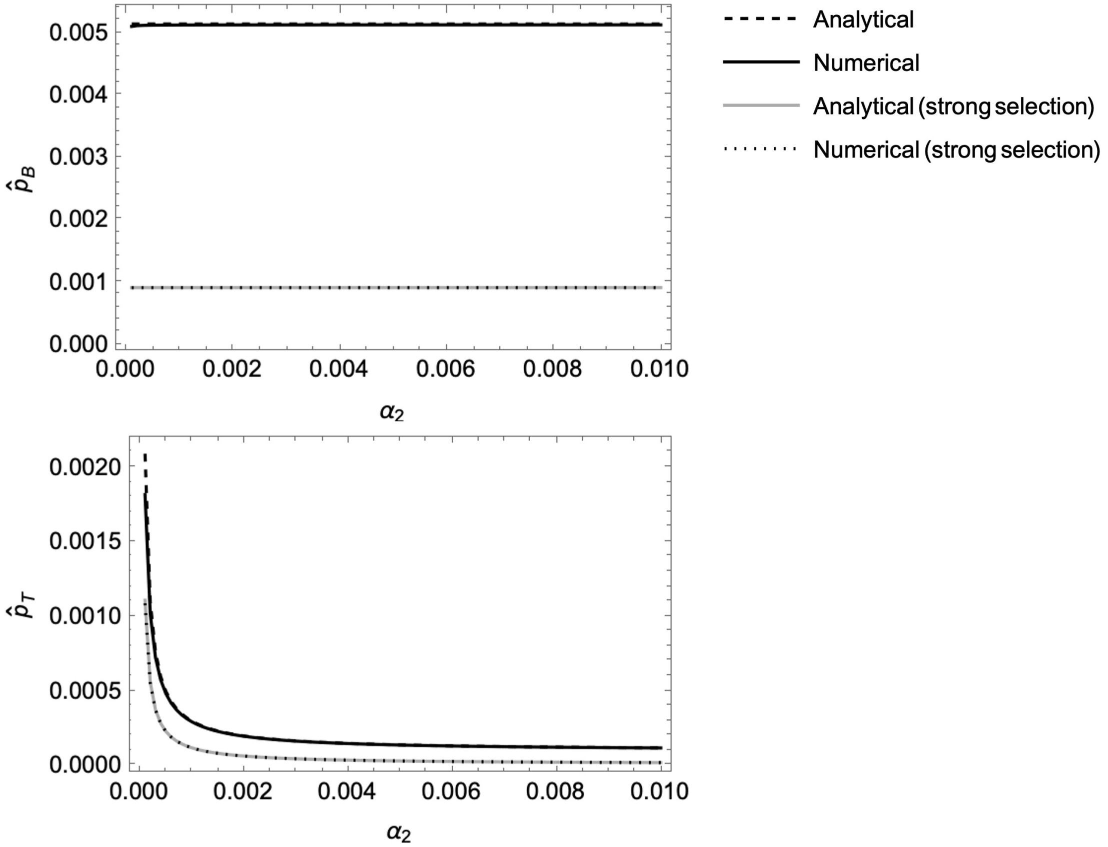

Figure S3: The equilibrium frequencies of the epiallele (top) and transposon (bottom) as the epimutation rate varies and with different magnitudes of selection against the epiallele (k = 0.001 and k = 0.01, strong). The other parameter values are: $\alpha_1 = 10^{-5}$, $\beta_1 = 0.001$, $\beta_2 = 10^{-5}$, $z = 10^{-7}$, $y = 10^{-5}$, S = 0.01.

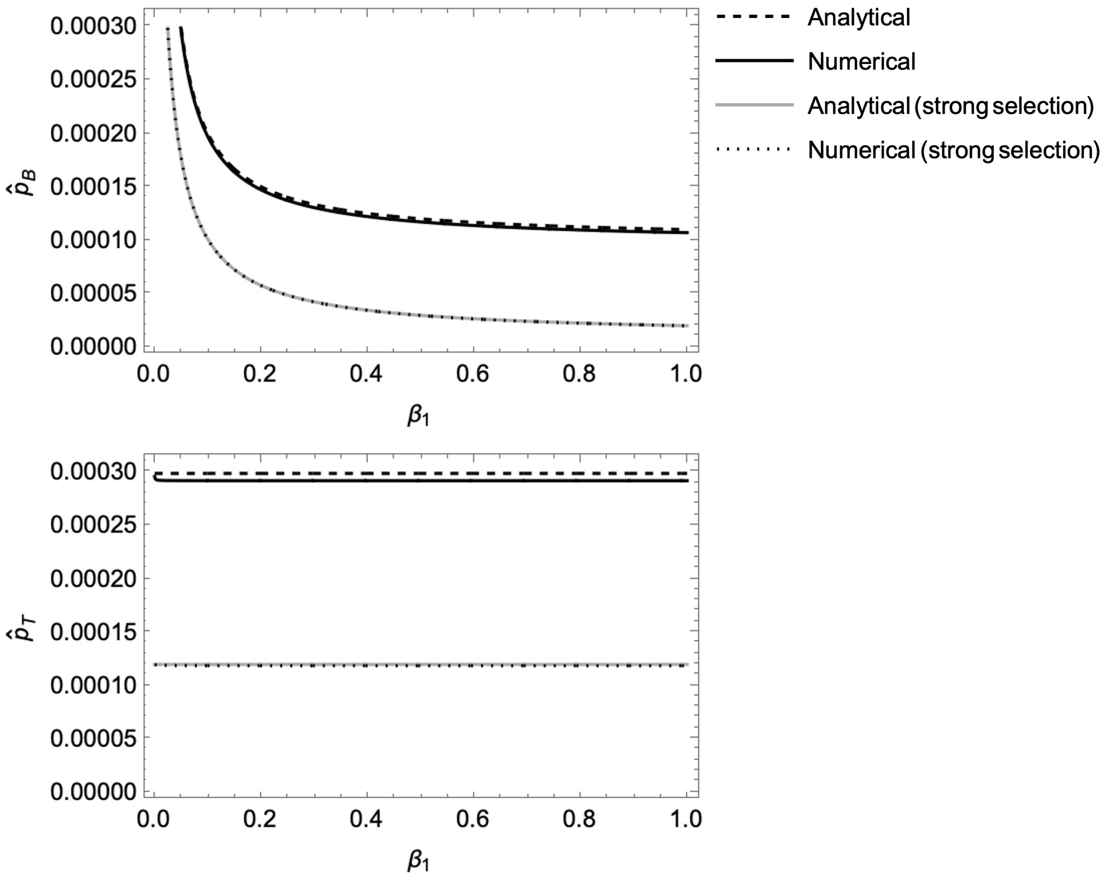

Figure S4: The equilibrium frequencies of the epiallele (top and transposon (bottom) as the background epiallele reversion rate varies and with different magnitudes of selection against the epiallele (k = 0.001 and k = 0.01, strong). The other parameter values are: $\alpha_1 = 10^{-5}$, $\alpha_2 = 0.001$, $\beta_2 = 10^{-5}$, $z = 10^{-7}$, $y = 10^{-5}$, S = 0.01.

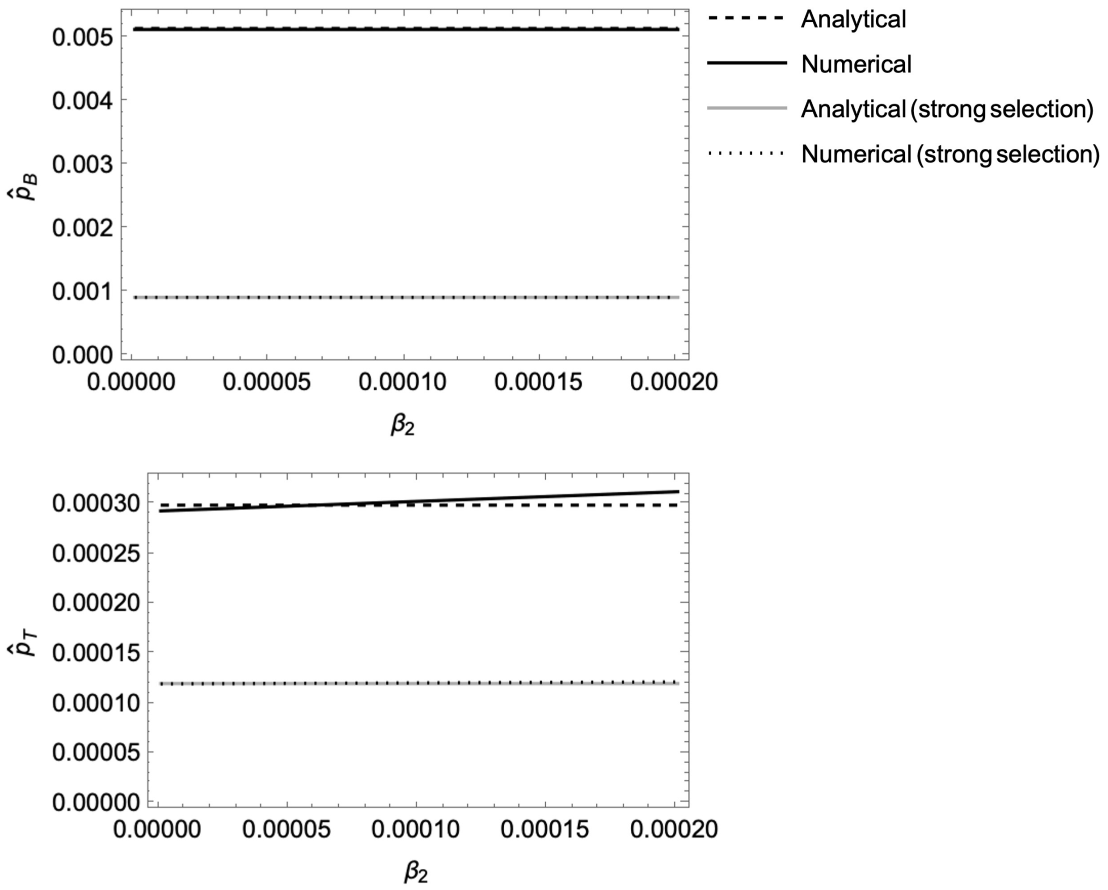

Figure S5: The equilibrium frequencies of the epiallele (top) and transposon (bottom) as the epiallele reversion rate adjacent to the transposon varies and with different magnitudes of selection against the epiallele (k = 0.001 and k = 0.01, strong). The other parameter values are: $\alpha_1 = 10^{-5}$, $\alpha_2 = 0.001$, $\beta_1 = 0.001$, $z = 10^{-7}$, $y = 10^{-5}$, S = 0.01.

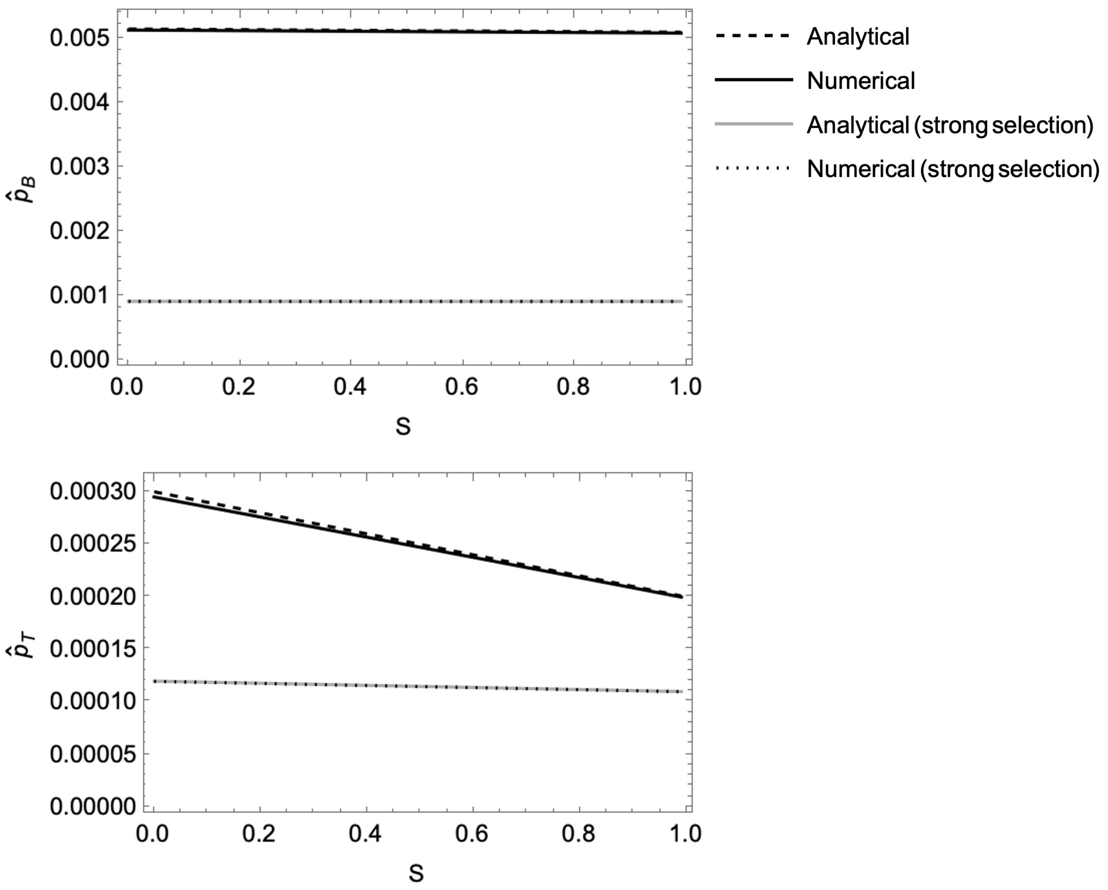

Figure S6: The equilibrium frequencies of the epiallele (top) and transposon (bottom) as the selfing rate varies and with different magnitudes of selection against the epiallele (k = 0.001 and k = 0.01, strong). The other parameter values are: α_1= 10^{-5}, α_2= 0.001, β_1= 0.001 , β_2 = 10^{-5},
z = 10^{-7}, y = 10^{-5}.

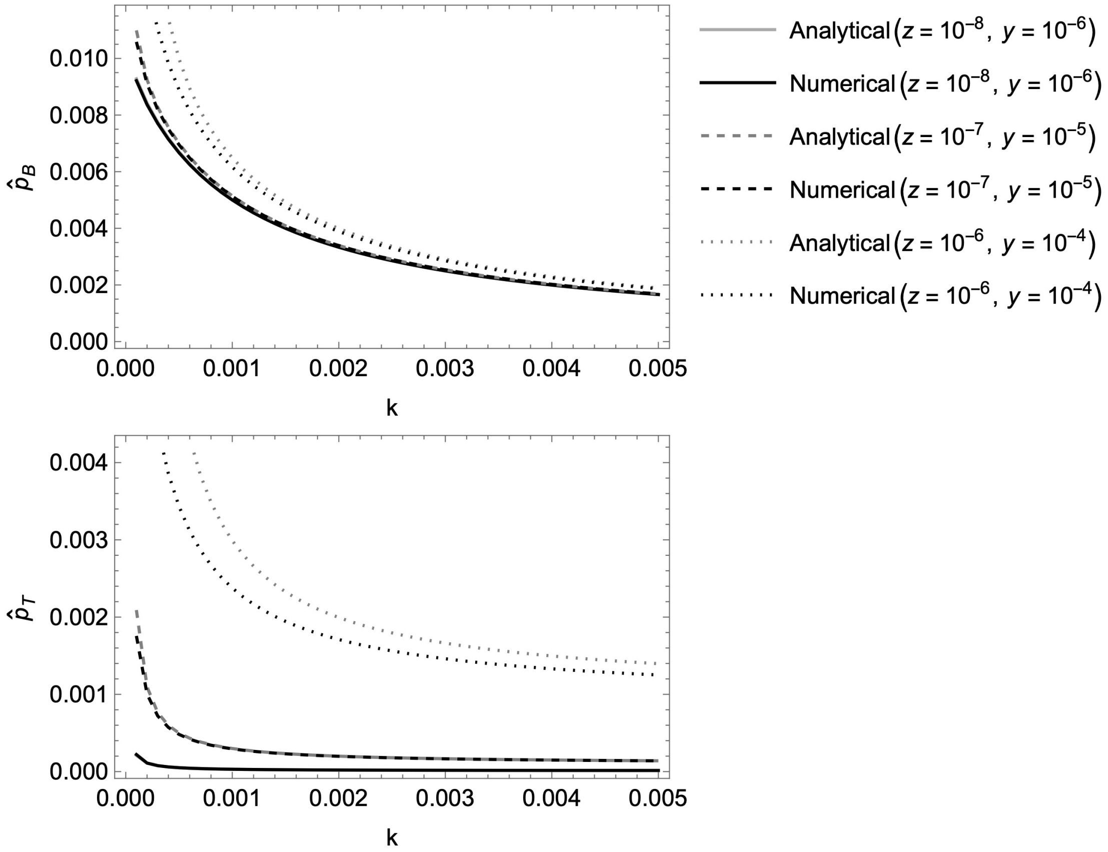

Figure S7: The equilibrium frequencies of the epiallele (top and transposon (bottom) as the background epimutation rate varies and with different magnitudes of selection against the epiallele (k = 0.001 and k = 0.01, strong).. The other parameter values are: $\alpha_1 = 10^{-5}$, $\alpha_2 = 0.001$, $\beta_1 = 0.001$, $\beta_2 = 10^{-5}$, $z = 10^{-7}$, $y = 10^{-5}$, S = 0.01.

Case B: The transposon and epiallele are deleterious

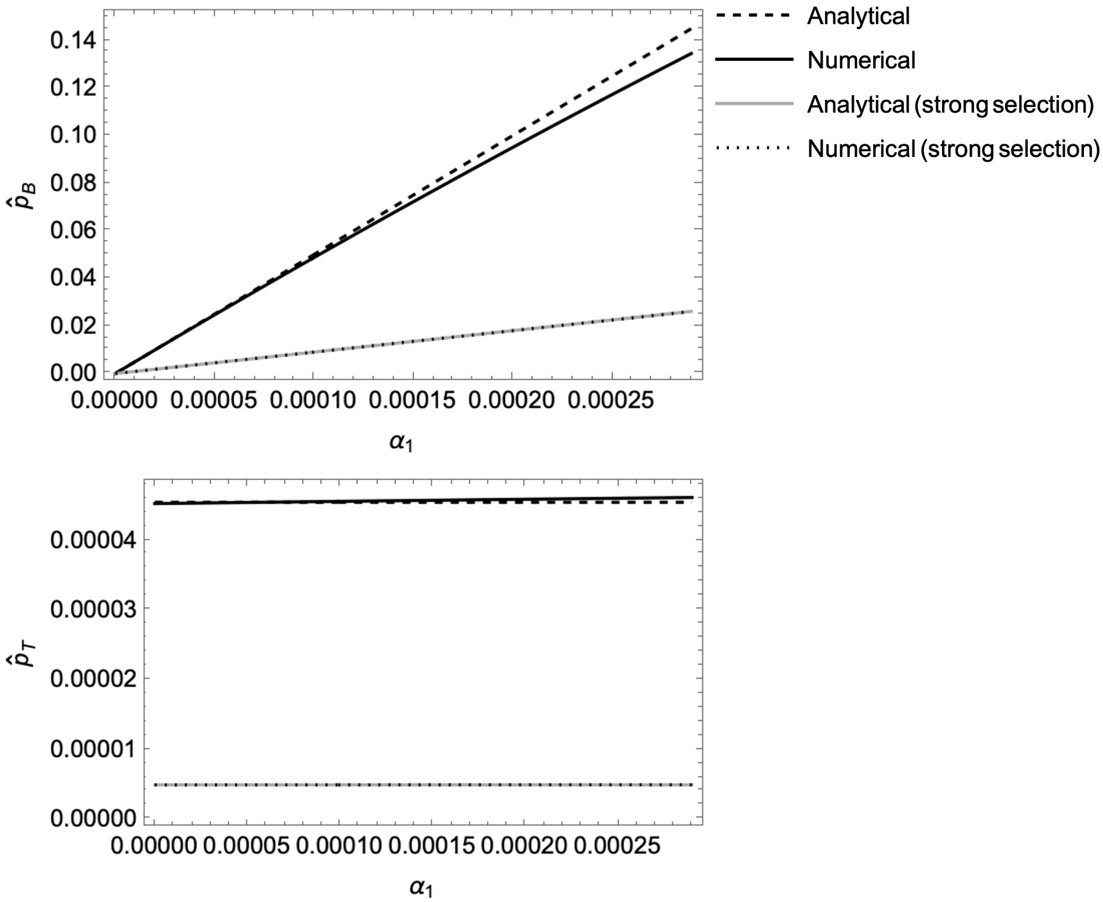

Figure S8: The equilibrium frequencies of the epiallele (top) and transposon (bottom) as the background epimutation rate varies and with different magnitudes of selection against the epiallele (k = 0.001, $\mathcal{L}$ = 0.002 and k = 0.01, $\mathcal{L}$ = 0.02 strong). The other parameter values are: $\alpha_2 = 10^{-3}$, $\beta_1 = 10^{-3}$, $\beta_2 = 10^{-5}$, $z = 10^{-7}$, $y = 10^{-5}$, S = 0.01.

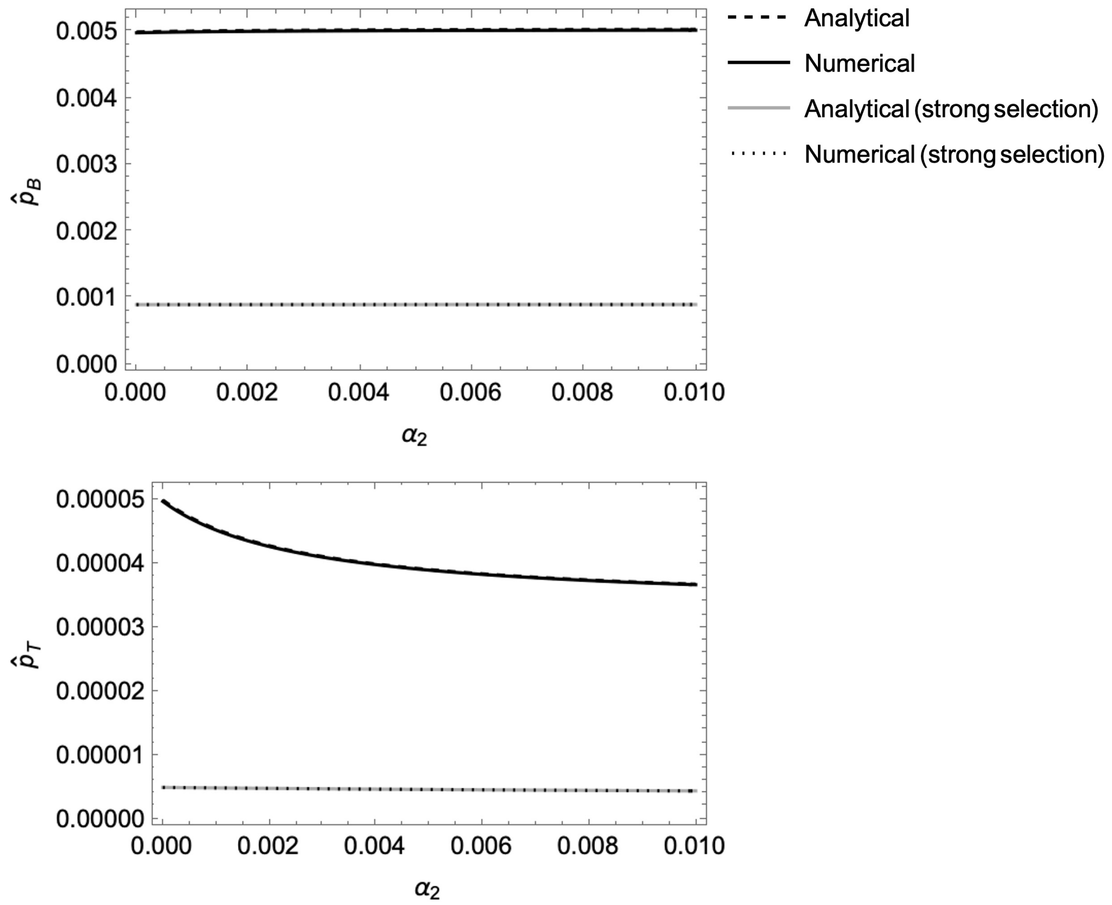

Figure S9: The equilibrium frequencies of the epiallele (top) and transposon (bottom) as the epimutation rate varies and with different magnitudes of selection against the epiallele (k = 0.001, $\mathcal{L}$ = 0.002 and k = 0.01, $\mathcal{L}$ = 0.02 strong). The other parameter values are: $\alpha_1 = 10^{-5}$, $\beta_1 = 10^{-3}$, $\beta_2 = 10^{-5}$, $z = 10^{-7}$, $y = 10^{-5}$, S = 0.01.

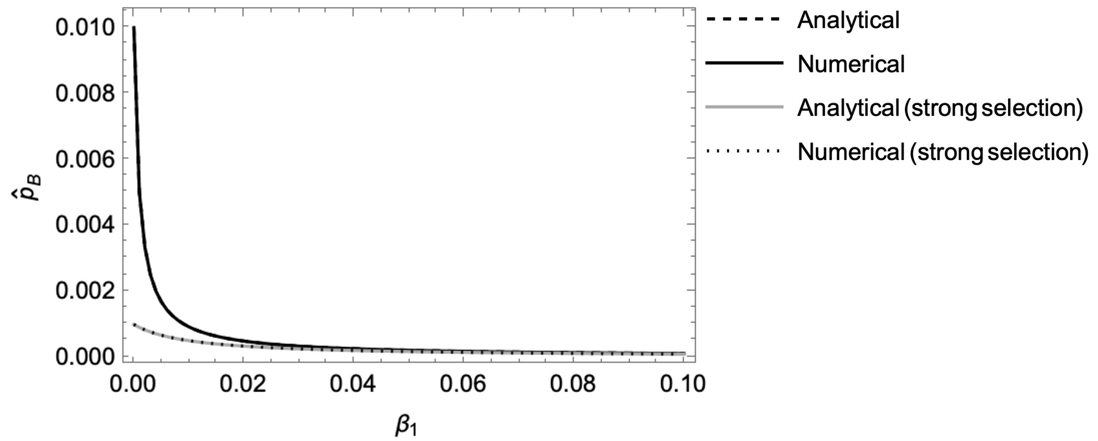

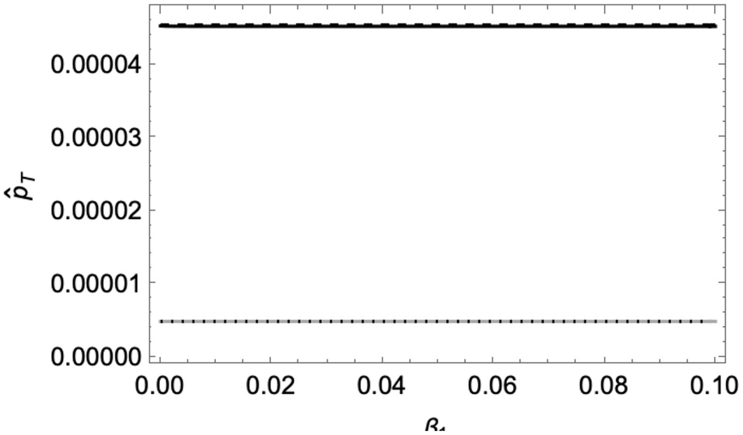

Figure S10: The equilibrium frequencies of the epiallele (top) and transposon (bottom) as the background epiallele reversion rate varies and with different magnitudes of selection against the epiallele (k = 0.001, $\mathcal{L}$ = 0.002 and k = 0.01, $\mathcal{L}$ = 0.02 strong). The other parameter values are: α_1 = 10^{-5}, α_2= 10^{-3}, β_2 = 10^{-5}, z = 10^{-7}, y = 10^{-5}, S = 0.01.

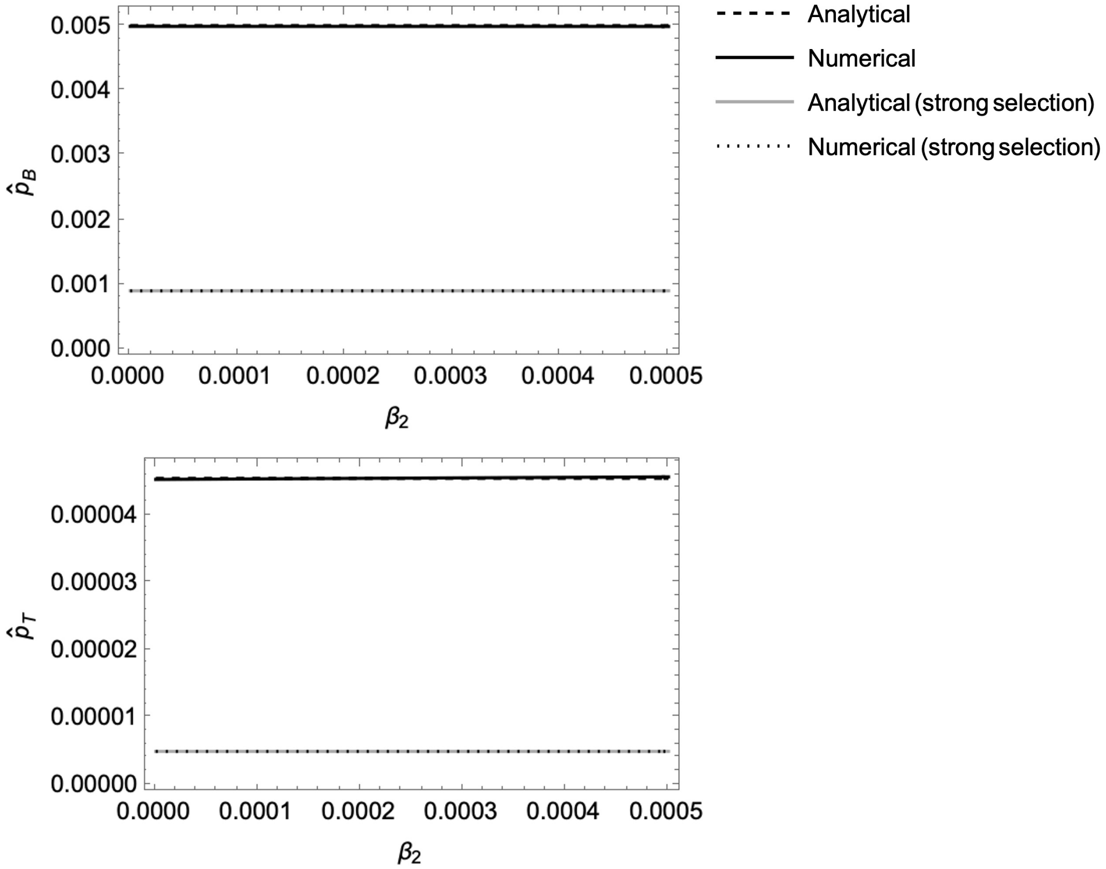

Figure S11: The equilibrium frequencies of the epiallele (top) and transposon (bottom) as the background epiallele reversion rate adjacent to the transposon varies and with different magnitudes of selection against the epiallele (k = 0.001, $\mathcal{L}$ = 0.002 and k = 0.01, $\mathcal{L}$ = 0.02 strong). The other parameter values are: α_1 = 10^{-5}, α_2= 10^{-3}, β_1 = 10^{-3}, z = 10^{-7}, y = 10^{-5}, S = 0.01.

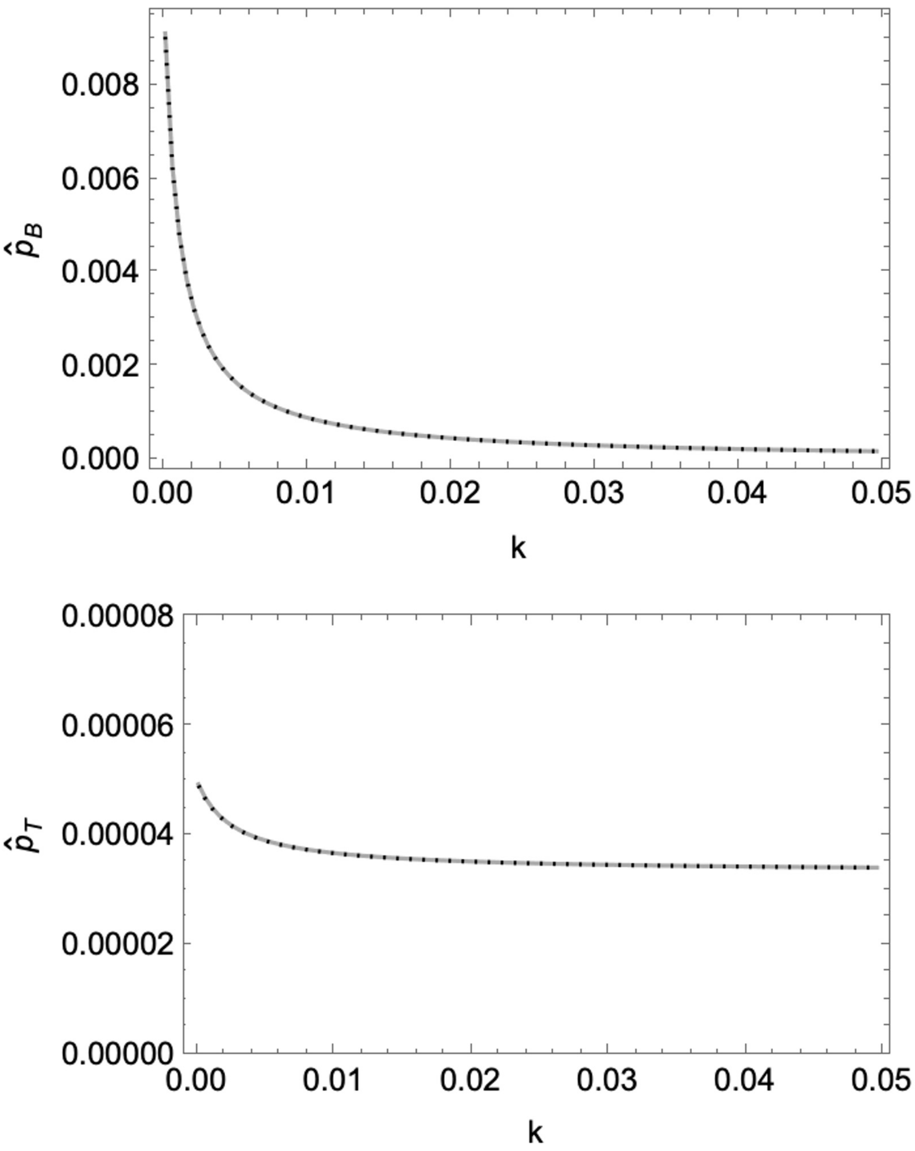

Figure S12: The equilibrium frequencies of the epiallele (top) and transposon (bottom) as the the strength of selection against the epiallele varies. The gray line represents the analytical approximation and the black dotted line represents numerical simulations of the recursion equations. The other parameter values are: $\alpha_1 = 10^{-5}$, $\alpha_2 = 10^{-3}$, $\beta_1 = 10^{-3}$, $\beta_2 = 10^{-5}$, $z = 10^{-7}$, $y = 10^{-5}$, $S = 0.01$, $\mathcal{L} = 0.002$.

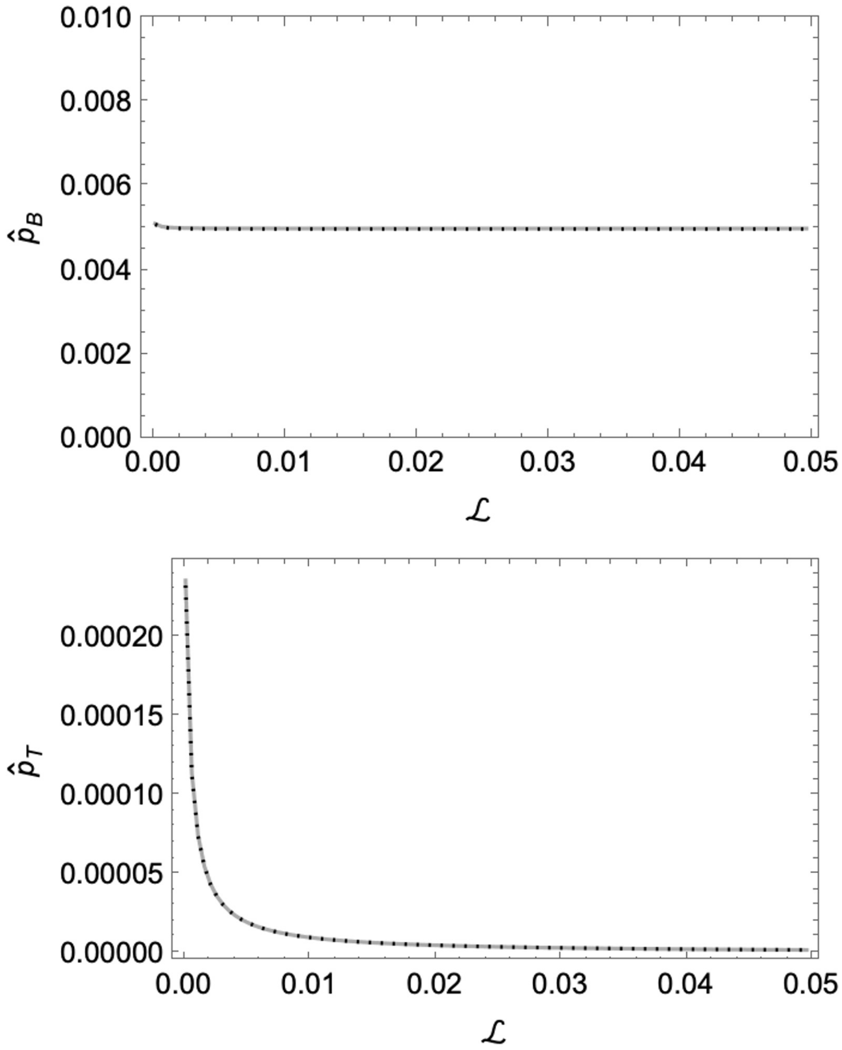

Figure S13: The equilibrium frequencies of the epiallele (top) and transposon (bottom) as the the strength of selection against the transposon varies. The gray line represents the analytical approximation and the black dotted line represents numerical simulations of the recursion equations. The other parameter values are: $\alpha_1 = 10^{-5}$, $\alpha_2 = 10^{-3}$, $\beta_1 = 10^{-3}$, $\beta_2 = 10^{-5}$, $z = 10^{-7}$, $y = 10^{-5}$, S = 0.01, k = 0.001.

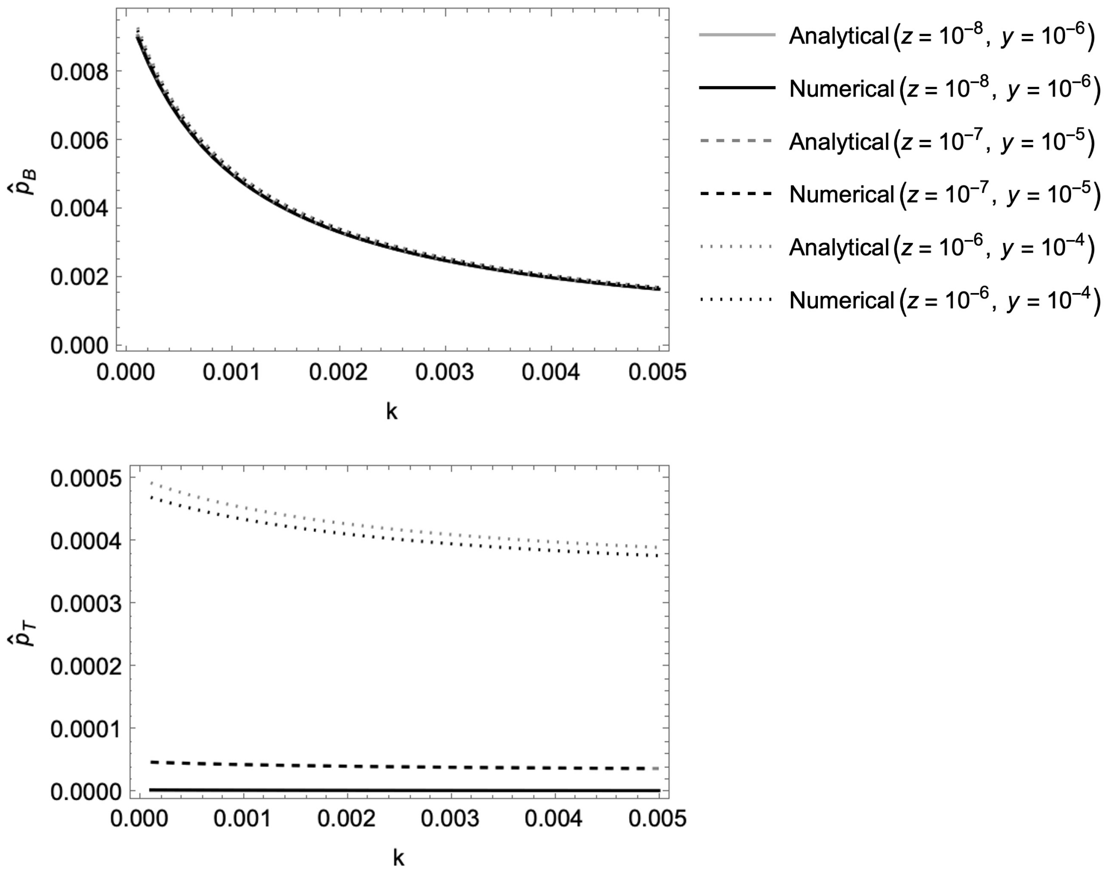

Figure S14: The equilibrium frequencies of the epiallele (top) and transposon (bottom) as the the strength of selection against the epiallele varies and for different magnitudes of the transposition and deletion rates. The other parameter values are: $\alpha_1 = 10^{-5}$, $\alpha_2 = 10^{-3}$, $\beta_1 = 10^{-3}$, $\beta_2 = 10^{-5}$, $z = 10^{-7}$, $y = 10^{-5}$, $S = 0.01$, $\mathcal{L} = 0.002$.

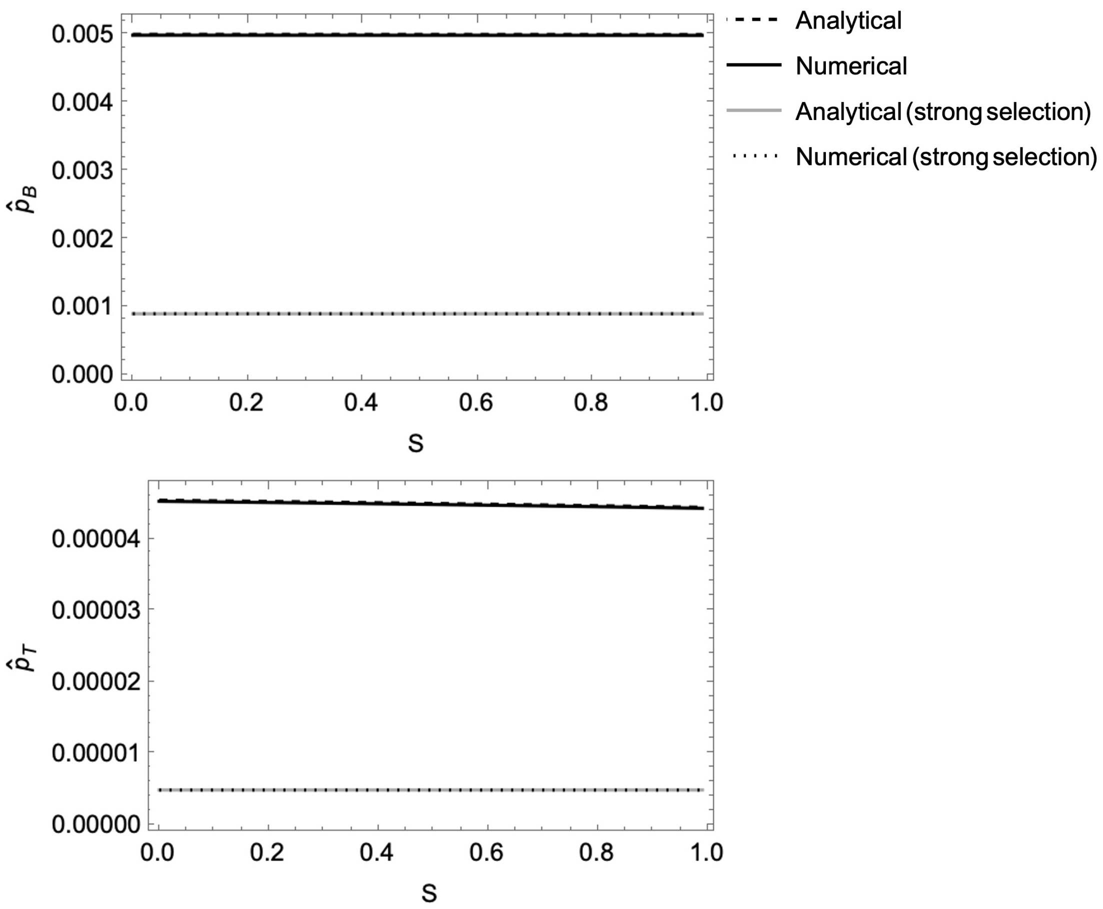

Figure S15: The equilibrium frequencies of the epiallele (top) and transposon (bottom) as the the selfing rate varies and with different magnitudes of selection (k = 0.001, $\mathcal{L}$ = 0.002 and k = 0.01, $\mathcal{L}$ = 0.02 strong). The other parameter values are: $\alpha_1 = 10^{-5}$, $\alpha_2 = 10^{-3}$, $\beta_1 = 10^{-3}$, $\beta_2 = 10^{-5}$, $z = 10^{-7}$, $y = 10^{-5}$, S = 0.01.

Model 2: Three locus haploid mating and selection

A - Introduction

Here we follow the same life cycle as model 1. The only distinction is that we incorporate a third locus that acts as a modifier of the recombination rate (see table below for a summary of the assumptions).

Recombination Modifier Genotype	Intergenic recombination rate 1	Transposon Locus	Intergenic recombination rate 2	(Epi) genetic Locus
J / J	r1	t or T	r2	A or B
J / j	r3	t or T	r4	A or B
j / j	r5	t or T	r6	A or B

Noting that a distinct recombination rate is allowed for both intergenic regions, and we allow for incomplete dominance for the modifier of recombination locus.

The sequence of events in the life cycle follows model 1 directly, such that organisms are only transiently diploid during a mating phase and are haploid throughout the rest of the life cycle. The four events in the life cycle are

1- Mating of haploid organisms occurs, conditioned on epimutation occurring on both gametes (regardless of whether they pair randomly or with selfing).

2- Recombination occurs prior to next generation of haploid organisms being generated

3- Selection occurs on haploid organisms

4- Transposition/deletion occurs

Cycle repeats

B - Overview of Model

The first event that occurs during the life cycle is the mating of haploid organisms, conditioned on epimutation. For a demonstration, we track the frequency of the JtA haplotype throughout the life cycle. There are eight possible transient (epi)genotypes that contribute to the frequency of the JtA haplotype:

$$p_{J,t,A,J,t,A}{}^{(1)} = S\left((1-\alpha_1)^2\, p_{J,t,A} + \beta_1^2\, p_{J,t,B}\right) + (1-S)\left((1-\alpha_1)^2\, p_{J,t,A}^2 + 2\,(1-\alpha_1)\,\beta_1\, p_{J,t,A}\, p_{J,t,B} + \beta_1^2\, p_{J,t,B}^2\right)$$

$$p_{J,t,A,J,t,B}{}^{(1)} = S\left(2\,(1-\alpha_1)\,\alpha_1\, p_{J,t,A} + 2\,(1-\beta_1)\,\beta_1\, p_{J,t,B}\right) + (1-S)\left(2\,(1-\alpha_1)\,\alpha_1\, p_{J,t,A}^2 + 2\,(1-\alpha_1)\,(1-\beta_1)\, p_{J,t,A}\, p_{J,t,B} + 2\,\alpha_1\,\beta_1\, p_{J,t,A}\, p_{J,t,B} + 2\,(1-\beta_1)\,\beta_1\, p_{J,t,B}^2\right)$$

$$p_{J,t,A,J,T,A}{}^{(1)} = 2\,(1-S)\left((1-\alpha_1)\,(1-\alpha_2)\, p_{J,t,A}\, p_{J,T,A} + (1-\alpha_2)\,\beta_1\, p_{J,t,B}\, p_{J,T,A} + (1-\alpha_1)\,\beta_2\, p_{J,t,A}\, p_{J,T,B} + \beta_1\,\beta_2\, p_{J,t,B}\, p_{J,T,B}\right)$$

$$p_{J,t,A,J,T,B}{}^{(1)} = 2\,(1-S)\left((1-\alpha_1)\,\alpha_2\, p_{J,t,A}\, p_{J,T,A} + \alpha_2\,\beta_1\, p_{J,t,B}\, p_{J,T,A} + (1-\alpha_1)\,(1-\beta_2)\, p_{J,t,A}\, p_{J,T,B} + \beta_1\,(1-\beta_2)\, p_{J,t,B}\, p_{J,T,B}\right)$$

$$p_{J,t,A,j,t,A}{}^{(1)} = (1-S)\left((1-\alpha_1)\,(1-\alpha_1)\, 2\, p_{J,t,A}\, p_{j,t,A} + (1-\alpha_1)\,\beta_1\, 2\, p_{J,t,A}\, p_{j,t,B} + \beta_1\,(1-\alpha_1)\, 2\, p_{J,t,B}\, p_{j,t,A} + \beta_1\,\beta_1\, 2\, p_{J,t,B}\, p_{j,t,B}\right)$$

$$p_{J,t,A,j,t,B}{}^{(1)} = (1-S)\left((1-\alpha_1)\,\alpha_1\, 2\, p_{J,t,A}\, p_{j,t,A} + (1-\alpha_1)\,(1-\beta_1)\, 2\, p_{J,t,A}\, p_{j,t,B} + \beta_1\,\alpha_1\, 2\, p_{J,t,B}\, p_{j,t,A} + \beta_1\,(1-\beta_1)\, 2\, p_{J,t,B}\, p_{j,t,B}\right)$$

$$p_{J,t,A,j,T,A}{}^{(1)} = (1-S)\left((1-\alpha_1)\,(1-\alpha_2)\, 2\, p_{J,t,A}\, p_{j,T,A} + (1-\alpha_1)\,\beta_2\, 2\, p_{J,t,A}\, p_{j,T,B} + \beta_1\,(1-\alpha_2)\, 2\, p_{J,t,B}\, p_{j,T,A} + \beta_1\,\beta_2\, 2\, p_{J,t,B}\, p_{j,T,B}\right)$$

$$p_{J,t,A,j,T,B}{}^{(1)} = (1-S)\left((1-\alpha_1)\,\alpha_2\, 2\, p_{J,t,A}\, p_{j,T,A} + (1-\alpha_1)\,(1-\beta_2)\, 2\, p_{J,t,A}\, p_{j,T,B} + \beta_1\,\alpha_2\, 2\, p_{J,t,B}\, p_{j,T,A} + \beta_1\,(1-\beta_2)\, 2\, p_{J,t,B}\, p_{j,T,B}\right)$$

$$p_{J,t,A}{}^{(1)} = p_{J,t,A,J,t,A}{}^{(1)} + \frac{1}{2}\, p_{J,t,A,J,t,B}{}^{(1)} + \frac{1}{2}\, p_{J,t,A,J,T,A}{}^{(1)} + \frac{1}{2}\, p_{J,t,A,J,T,B}{}^{(1)} + \frac{1}{2}\, p_{J,t,A,j,t,A}{}^{(1)} + \frac{1}{2}\, p_{J,t,A,j,t,B}{}^{(1)} + \frac{1}{2}\, p_{J,t,A,j,T,B}{}^{(1)} + \frac{1}{2}\, p_{J,t,A,j,T,A}{}^{(1)}$$

The next step in the life cycle is recombination of this temporary diploid phase. After recombination, the frequency of the JtA haplotype is

$$p_{J,t,A}{}^{(2)} = p_{J,t,A,J,t,A}{}^{(1)} + 1/2\, p_{J,t,A,J,t,B}{}^{(1)} + 1/2\, p_{J,t,A,J,T,A}{}^{(1)} + \left((1-r_1)(1-r_2)\,1/2 + r_1(1-r_2)\,1/2\right) p_{J,t,A,J,T,B}{}^{(1)} + \left((1-r_1)\, r_2\, 1/2 + r_1\, r_2\, 1/2\right) p_{J,T,A,J,t,B}{}^{(1)} + 1/2\, p_{J,t,A,j,t,A}{}^{(1)} + \left((1-r_3)(1-r_4)\,1/2 + r_3\, r_4\, 1/2\right) p_{J,t,A,j,t,B}{}^{(1)} + \left(r_3(1-r_4)\,1/2 + (1-r_3)\, r_4\, 1/2\right) p_{J,t,B,j,t,A}{}^{(1)} + \left((1-r_3)(1-r_4)\,1/2 + (1-r_3)\, r_4\, 1/2\right) p_{J,t,A,j,T,A}{}^{(1)} + (1-r_3)(1-r_4)\,1/2\, p_{J,t,A,j,T,B}{}^{(1)} + (1-r_3)\, r_4\, 1/2\, p_{J,t,B,j,T,A}{}^{(1)} + \left(r_3(1-r_4)\,1/2 + r_3\, r_4\, 1/2\right) p_{J,T,A,j,t,A}{}^{(1)} + r_3\, r_4\, 1/2\, p_{J,T,A,j,t,B}{}^{(1)} + r_3(1-r_4)\,1/2\, p_{J,T,B,j,t,A}{}^{(1)}$$

For example, the last term in the equation (i.e. $r_3 (1-r_4)\, 1/2\, p_{J,T,B,j,t,A}{}^{(1)}$) corresponds to recombination occurring at the first intergenic region (r_3) and not occurring at the second intergenic region $(1-r_4)$. Noting that the subscripts are 3 and 4 for the recombination rates because the modifier is heterozygous (J/j). After recombination, only half of the resulting haploid organism will be JtA, which is why the 1/2 is present in the term.

As the next step in the life cycle, natural selection occurs on adult haploid organisms where $w_i$is the symbol for relative fitness and $\overline{w}$ is the mean fitness of the population. The frequency of the JtA haplotype after natural selection is

$$p_{J,t,A}{}^{(3)} = p_{J,t,A}{}^{(2)}\, w_1 \left(\frac{1}{\overline{w}}\right)$$

where $\overline{w} = p_{J,t,A}{}^{(2)} w_1 + p_{J,t,B}{}^{(2)} w_2 + p_{J,T,A}{}^{(2)} w_3 + p_{J,T,B}{}^{(2)} w_4 + p_{j,t,A}{}^{(2)} w_5 + p_{j,t,B}{}^{(2)} w_6 + p_{j,T,A}{}^{(2)} w_7 + p_{j,T,B}{}^{(2)} w_8$

As the final event in the life cycle, transposition and deletion occur to the haploid organisms. The final frequency of the JtA haplotype is

$$p_{J,t,A}{}^{(4)} = (1-z)\, p_{J,t,A}{}^{(3)} + y p_{J,T,A}{}^{(3)}$$

After plugging each event into the consecutive event and then simplifying, the final recursion equations for the haplotype frequencies are derived. These full equation are given in Supplement 2, under Model 3, section A. Analytical results were not obtainable because of the complicated nature of the system. In turn, the Mathematica code used to run numerical simulations for the system of recursion equations is given in Supplement 2, Model 3, section A.

C - Figures

Summary

Figure S16 (below) assumes stronger linkage between the modifier and transposon locus relative to the main text (r = 0.005 versus r = 0.05, respectively). Invasion occurs at a faster rate with the increased linkage allowing for more efficient indirect selection for the modifier and stronger selection against the epiallele when $\alpha_2 > \beta_1$ (top panel), but the relationship is more nuanced when $\alpha_2 < \beta_1$ (bottom panel). Figure S17 has weaker linkage between between the modifier and transposon locus relative to the main text (r = 0.5 versus r = 0.05, respectively). Invasion of the modifier occurs but at a slower rate due to weaker indirect selection for the modifier due to the high recombination rate.

Figure S18 shows the invasion conditions for the modifier as the forward (α_2) and backward (β_1) rates of epimutation vary. As α_2 gets stronger invasion (or purging) occurs at a faster rate whereas the relationship is more nuanced as $\beta_1$increases. Of note, when $\alpha_2 = \beta_1$, no invasion (or purging) occurs likely because there is no indirect effect of recombination on the effective epimutation rate, and in turn no effect on the marginal fitness of the wild-type and incoming modifier. Therefore, at equilibrium there can be polymorphism at the modifier locus, albeit this is exact parameter situation is unlikely to occur. With reduced epimutation rates (figure S19), the speed of invasion of a recombination modifier becomes slower. Figure S21 shows that higher rates of transposition/deletion increase the speed of invasion.

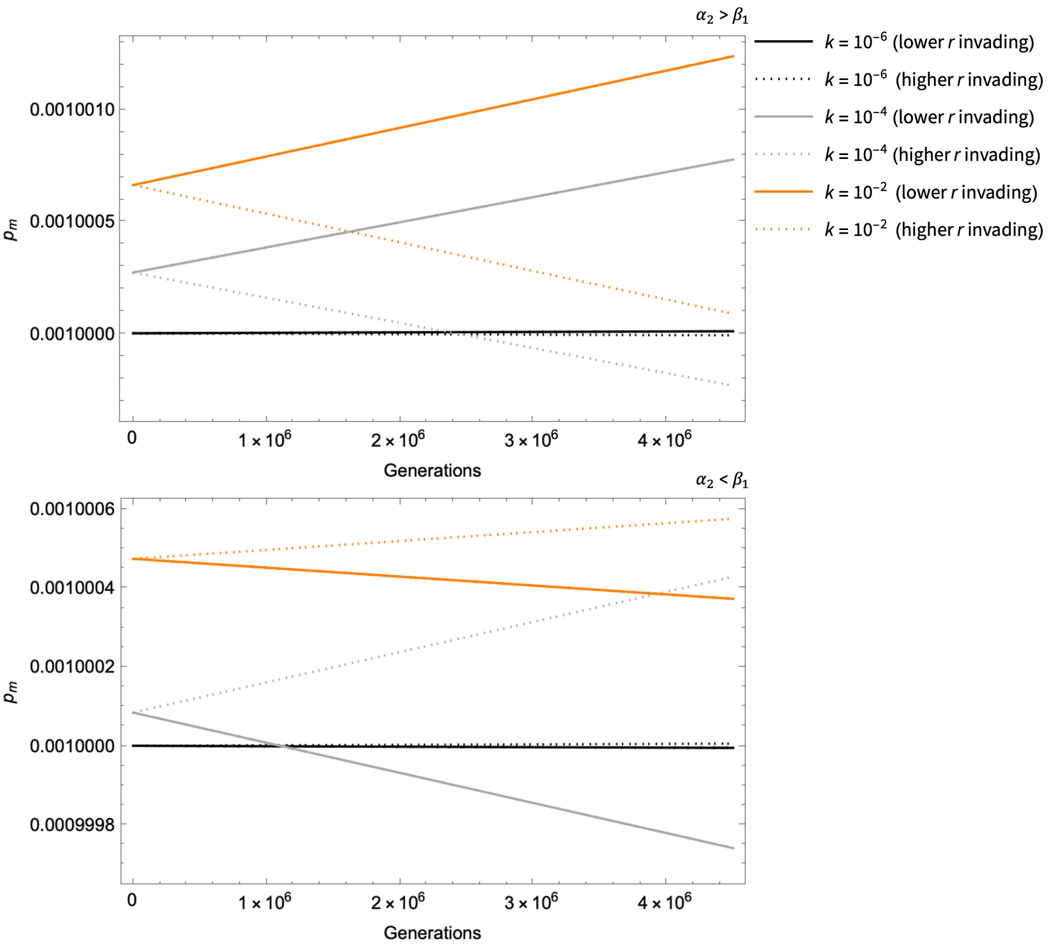

Figure S16: Numerical simulations for invasion of a modifier of recombination with varying strength of selection against the epiallele and stronger linkage between the modifier and transposon locus (r = 0.005) compared to the main text figure 4. Solid lines are when the introduced modifier has a lower recombination rate than the wild-type rate (1% lower when homozygous) and the dashed lines are when the modifier has a higher rate (1% higher when homozygous). Top panel shows invasion conditions when $\alpha_2 = 0.005 > \beta_1 = 0.001$ and bottom panel is the reverse $\alpha_2 = 0.001 < \beta_1 = 0.005$. Other

parameter values are $\alpha_1 = 10^{-5}$, $\beta_2 = 10^{-5}$, $z = 10^{-6}$, $y = 10^{-4}$.

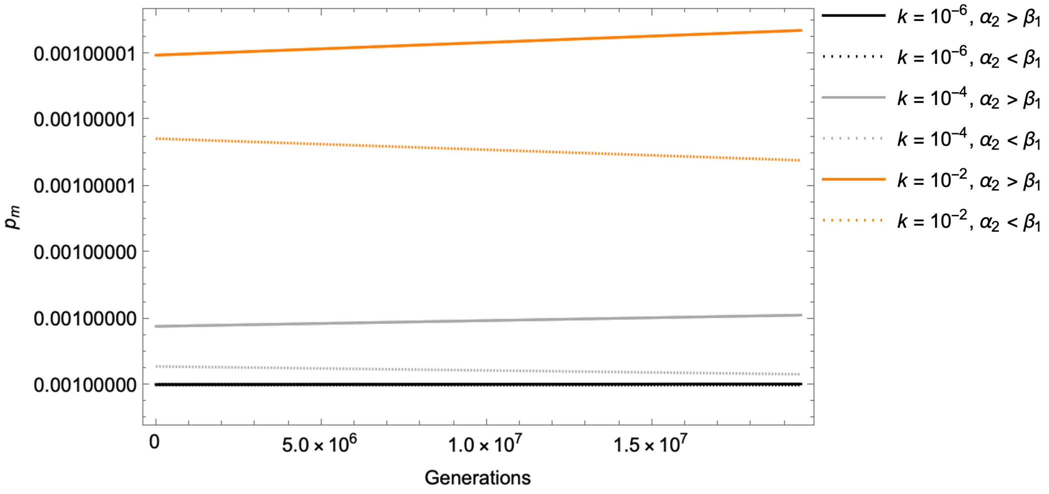

Figure S17: Numerical simulations for invasion of a modifier of recombination with varying strength of selection against the epiallele and no linkage between the modifier and transposon locus (r = 0.5) compared to the main text figure 4. Solid lines are when the introduced modifier has a lower recombination rate than the wild-type rate (1% lower when homozygous) and the dashed lines are when the modifier has a higher rate (1% higher when homozygous). There are two scenarios: $\alpha_2 = 0.005 > \beta_1 = 0.001$ and $\alpha_2 = 0.001 < \beta_1 = 0.005$. Other parameter values are $\alpha_1 = 10^{-5}$, $\beta_2 = 10^{-5}$, $z = 10^{-6}$, $y = 10^{-4}$.

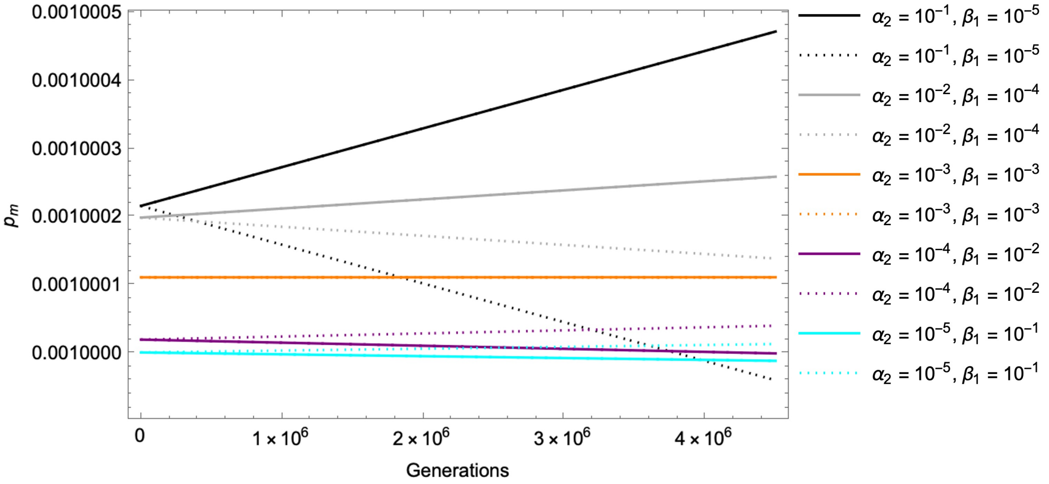

Figure S18: Numerical simulations for invasion of a modifier of recombination with varying forward and backward epimutation rates. Solid lines are when the introduced modifier has a lower recombination rate than the wild-type rate (1% lower when homozygous) and the dashed lines are when the modifier has a higher rate (1% higher when homozygous). Other parameter values are $\alpha_1 = 10^{-5}$, $\beta_2 = 10^{-5}$, $z = 10^{-6}$, $y = 10^{-4}$, recombination rate between modifier and transposon = 0.05.

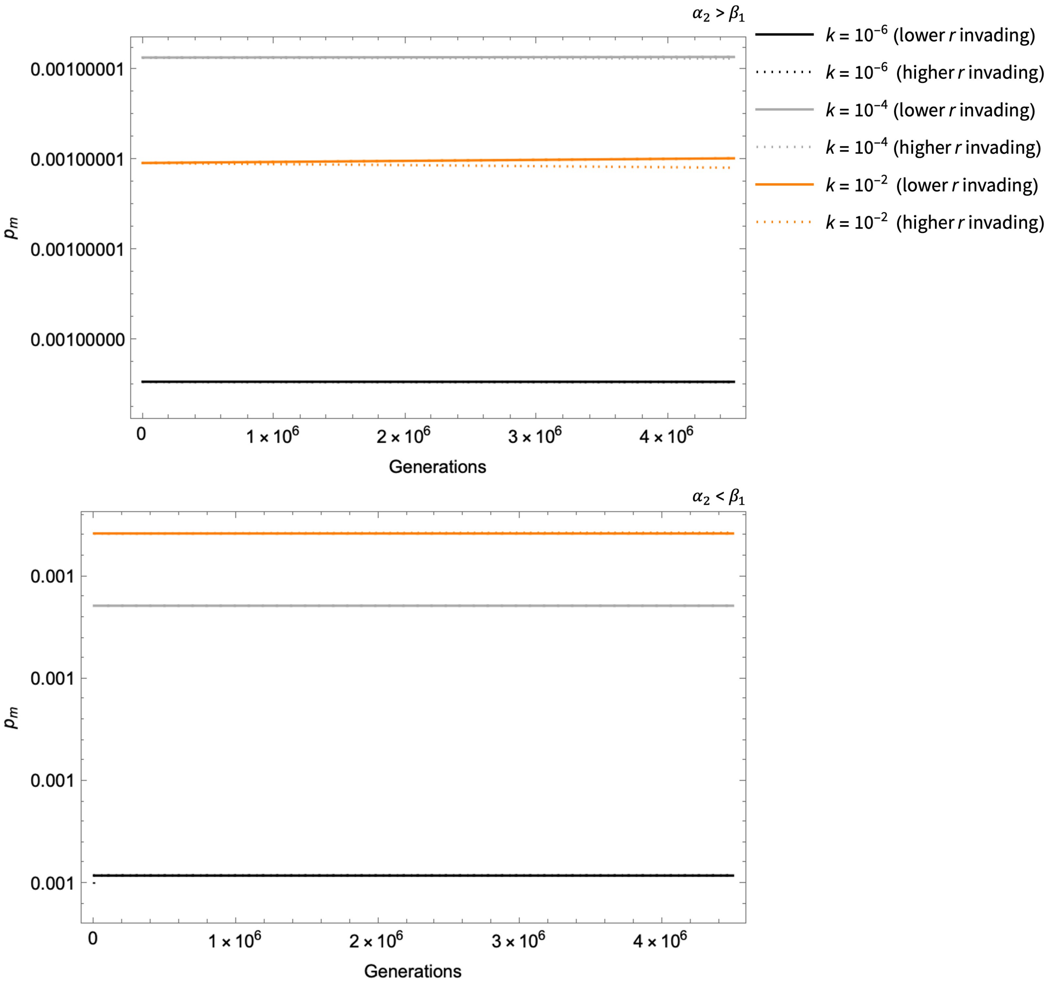

Figure S19: Numerical simulations for invasion of a modifier of recombination with varying strength of selection against the epiallele and lower epimutation rates. Solid lines are when the introduced modifier has a lower recombination rate than the wild-type rate (1% lower when homozygous) and the dashed lines are when the modifier has a higher rate (1% higher when homozygous). Top panel shows invasion conditions when $\alpha_2 = 5 \text{ x} 10^{-5} > \beta_1 = 1 \text{ x} 10^{-6}$ and bottom panel is the reverse $\alpha_2 = 1 \text{ x} 10^{-6} < \beta_1 = 5 \text{ x} 10^{-5}$. Other parameter values are $\alpha_1 = 10^{-7}$, $\beta_2 = 10^{-7}$, $z = 10^{-6}$, $y = 10^{-4}$, the recombination rate between the modifier and transposon = 0.05.

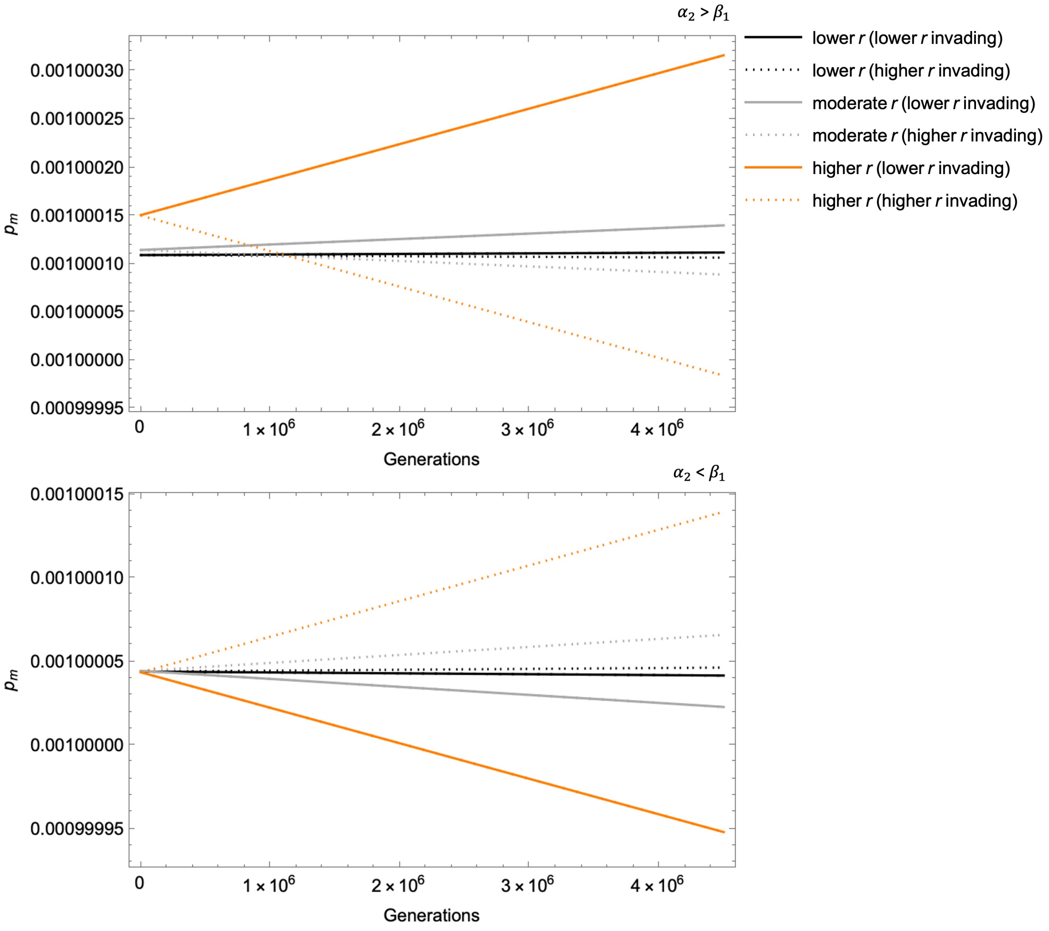

Figure S20: Numerical simulations for invasion of a modifier of recombination with varying recombination rates in between the transposon and epigenetic locus (lower r ~ 10^{-4}, moderate r ~ 10^{-3}, and higher r ~10^{-2}) rates. Solid lines are when the introduced modifier has a lower recombination rate than the wild-type rate (1% lower when homozygous) and the dashed lines are when the modifier has a higher rate (1% higher when homozygous). Top panel shows invasion conditions when α_2 = 0.005 > β_1 = 0.001 and bottom panel is the reverse α_2 = 0.001 < β_1 = 0.005. Other parameter values are $\alpha_1 = 10^{-5}$, $\beta_2 = 10^{-5}$, z = 10^{-6}, y = 10^{-4}, the recombination rate between the modifier and transposon = 0.05.

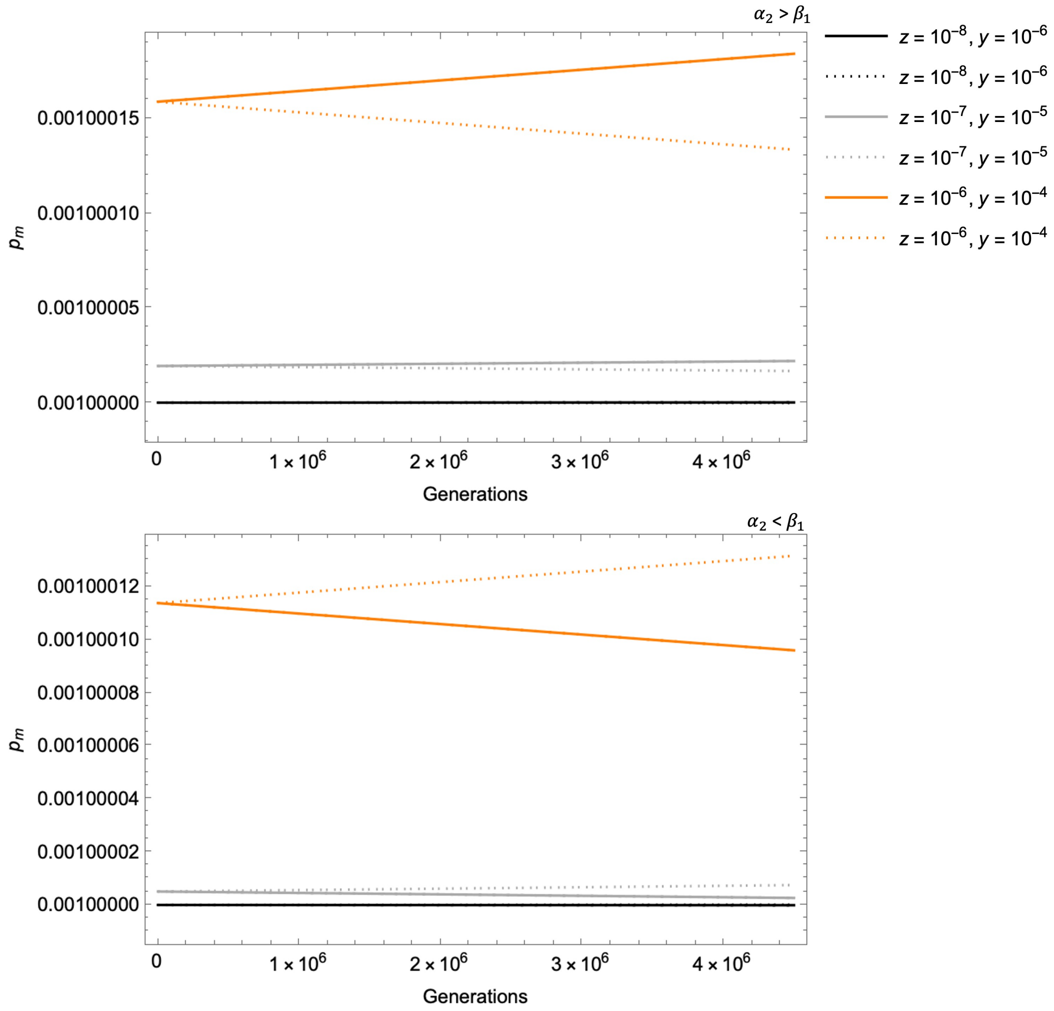

Figure S21: Numerical simulations for invasion of a modifier of recombination with varying transposition and deletion rates. Solid lines are when the introduced modifier has a lower recombination rate than the wild-type rate (1% lower when homozygous) and the dashed lines are when the modifier has a higher rate (1% higher when homozygous). Top panel shows invasion conditions when $\alpha_2 = 0.005 > \beta_1 = 0.001$ and bottom panel is the reverse $\alpha_2 = 0.001 < \beta_1 = 0.005$. Other parameter values are $\alpha_1 = 10^{-5}$, $\beta_2 = 10^{-5}$, the recombination rate between the modifier and transposon is 0.05.
