# Supplementary material for: Multi-locus models of deleterious epimutation-selection balance and the evolution of recombination modifiers
Source: G3 (Bethesda). 2026 Jun 11;16(8):jkag147. doi: 10.1093/g3journal/jkag147 (PMC13439953; doi:10.1093/g3journal/jkag147)
Supplement: Supplementary file 1 [file jkag147_supplementary_data.zip › File_S2_G3-2026-406777.pdf]

Model 3: Two locus haploid mating and diploid selection, overview of derivation

A - Introduction

This model assumes selection occurs at the diploid stage, in contrast to model 1 with haploid selection. This allows for the effects of dominance to be considered. The life cycle has three events:

1- Selection occurs on diploid organisms conditioned on epimutation and random pairing of haploid gametes.

2- Recombination generates the next generation of haploid gametes

3- Transposition/deletion occurs to haploid gametes

Cycle repeats

B - Overview of Model

Random Mating with Epimutation (1)

There are ten possible pairings of haploid gametes (see below) to generate diploid individuals.

Possible genotypes with random mating
tA X tA
tA X tB
tB X tB
TA X TA
TA X TB
TB X TB
tA X TA
tA X T B
TA X t B
tB X TB

The first step in the life cycle is random mating (model 4 allows for selfing). As there is no inbreeding, genotype frequencies can be assumed to be products of the haplotype frequencies (i.e. diploid parents can be assumed to be composed of haplotypes with given frequencies: $p_{i,j}$). As an explicit example to illustrate the derivation, we track the frequency of the tA haplotype throughout the life cycle. There are four (out of the ten) diploid genotypes that contribute to the frequency of the tA haplotype after epimutation:

$p_{t,A,t,A}{}^{(1)} = p_{t,A} (1-\alpha_1) p_{t,A} (1-\alpha_1) + p_{t,A} (1-\alpha_1) p_{t,B} \beta_1 + p_{t,B} \beta_1 p_{t,A} (1-\alpha_1) + p_{t,B} \beta_1 p_{t,B} \beta_1$

$p_{t,A,t,B}{}^{(1)} = \big(p_{t,A} \alpha_1 p_{t,A} (1-\alpha_1) + p_{t,A} \alpha_1 p_{t,B} \beta_1 + p_{t,A} (1-\alpha_1) p_{t,A} \alpha_1 +$

$$p_{t,A}(1-\alpha_1)\,p_{t,B}(1-\beta_1)+p_{t,B}\,\beta_1\,p_{t,A}\,\alpha_1+p_{t,B}\,\beta_1\,p_{t,B}(1-\beta_1)+p_{t,B}(1-\beta_1)\,p_{t,B}\,\beta_1+p_{t,B}(1-\beta_1)\,p_{t,A}(1-\alpha_1)\Big)$$

$$p_{t,A,T,A}{}^{(1)}=2\left(p_{t,A}(1-\alpha_1)\,p_{T,A}(1-\alpha_2)+p_{t,A}(1-\alpha_1)\,p_{T,B}\,\beta_2+p_{t,B}\,\beta_1\,p_{T,A}(1-\alpha_2)+p_{t,B}\,\beta_1\,p_{T,B}\,\beta_2\right)$$

$$p_{t,A,T,B}{}^{(1)}=2\left(p_{t,A}(1-\alpha_1)\,p_{T,A}\,\alpha_2+p_{t,A}(1-\alpha_1)\,p_{T,B}(1-\beta_2)+p_{t,B}\,\beta_1\,p_{T,A}\,\alpha_2+p_{t,B}\,\beta_1\,p_{T,B}(1-\beta_2)\right)$$

Specifically, for example, the term in the first line on the far right (i.e. $p_{t,B}\,\beta_1\,p_{t,B}\,\beta_1$) represents a case where there is a tA/tA genotype formed from two tB gametes that both underwent reverse epimutation (at rate β_1) prior to joining during mating. Noting that the current frequency of the tA haplotype is

$$p_{t,A}{}^{(1)}=p_{t,A,t,A}{}^{(1)}+\frac{1}{2}\,p_{t,A,t,B}{}^{(1)}+\frac{1}{2}\,p_{t,A,T,A}{}^{(1)}+\frac{1}{2}\,p_{t,A,T,B}{}^{(1)}$$

Natural selection occurs (2) for the next step in the life cycle, acting on diploid organisms. w_i represents the relative fitness for a given genotype and $\overline{w}$ is the symbol for mean fitness. Noting that only the bolded genotypes contribute to the frequency of the tA haplotype.

$$\mathbf{p_{t,A,t,A}{}^{(2)}=w_1\left(\frac{1}{\overline{w}}\right)p_{t,A,t,A}{}^{(1)}}$$

$$\mathbf{p_{t,A,t,B}{}^{(2)}=w_2\left(\frac{1}{\overline{w}}\right)p_{t,A,t,B}{}^{(1)}}$$

$$p_{t,B,t,B}{}^{(2)}=w_3\left(\frac{1}{\overline{w}}\right)p_{t,B,t,B}{}^{(1)}$$

$$p_{T,A,T,A}{}^{(2)}=w_4\left(\frac{1}{\overline{w}}\right)p_{T,A,T,A}{}^{(1\,A)}$$

$$p_{T,A,T,B}{}^{(2)}=w_5\left(\frac{1}{\overline{w}}\right)p_{T,A,T,B}{}^{(1)}$$

$$p_{T,B,T,B}{}^{(2)}=w_6\left(\frac{1}{\overline{w}}\right)p_{T,B,T,B}{}^{(1)}$$

$$\mathbf{p_{t,A,T,A}{}^{(2)}=w_7\left(\frac{1}{\overline{w}}\right)p_{t,A,T,A}{}^{(1)}}$$

$$p_{t,B,T,A}{}^{(2)}=w_8\left(\frac{1}{\overline{w}}\right)p_{t,B,T,A}{}^{(1)}$$

$$\mathbf{p_{t,A,T,B}{}^{(2)} = w_9 \left(\frac{1}{\overline{w}}\right) p_{t,A,T,B}{}^{(1)}}$$

$$p_{t,B,T,B}{}^{(2)} = w_{10} \left(\frac{1}{\overline{w}}\right) p_{t,B,T,B}{}^{(1)}$$

where $\overline{w}$ =

$$w_1\, p_{t,A,t,A}{}^{(2)} + w_2\, p_{t,A,t,B}{}^{(2)} + w_3\, p_{t,B,t,B}{}^{(2)} + w_4\, p_{T,A,T,A}{}^{(2)} + w_5\, p_{T,A,T,B}{}^{(2)} + w_6\, p_{T,B,T,B}{}^{(2)} + w_7\, p_{t,A,T,A}{}^{(2)} + w_8\, p_{t,B,T,A}{}^{(2)} + w_9\, p_{t,A,T,B}{}^{(2)} + w_{10}\, p_{t,B,T,B}{}^{(2)}$$

For the next event, recombination occurs (3) generating haploid gametes. The frequency of the tA haplotype after recombination is

$$p_{t,A}{}^{(3)} = p_{t,A,t,A}{}^{(2)} + p_{t,A,t,B}{}^{(2)} + p_{t,A,T,A}{}^{(2)} + \frac{1}{2}\, r\, p_{t,B,T,A}{}^{(2)} + \frac{1}{2}\,(1-r)\, p_{t,A,T,B}{}^{(2)}$$

Specifically, for example, the term on the far right (i.e. $\frac{1}{2}(1-r)\, p_{t,A,T,B}{}^{(2)}$) refers to the tA/TB genotype not undergoing recombination (with probability 1 - r), where half the gametes generated are tA (and the other half are TB).

As the final event in the life cycle, the haploid gametes undergo transposition/deletion. The frequency of the tA haplotype after transposition/deletion is

$$p_{t,A}{}^{(4)} = p_{t,A}{}^{(3)}\,(1-z) + p_{T,A}{}^{(3)}\, y$$

Haplotypes that are already tA do not undergo transposition event with probability (1 - z) or they are generated through a deletion event (with probability y) in the TA haplotypes.

Each event in the life cycle can be substituted into the consecutive event until the final recursion equations are derived. The full derivation and recursion equations for all haplotype frequencies is given in Supplement 2, under model 2. With regards to the analysis of the system of recursion equations, an overview of the methods are given in the Appendix of the main text and detailed calculation are provided in Supplement 2, Model 2, for case A and B.

Model 4: Three locus diploid mating and selection, overview of derivation

A - Introduction

The model assumes organisms are diploid throughout the life cycle and allows for random mating and selfing. This acts as an extension of model 2 with two loci, diploid selection, and random mating. There are two major events in the life cycle:

1- A particular genotype produces a given gamete conditioned on epimutation, recombination and transposition occurring.

Gametes can pair at random or through selfing.

2- Natural selection occurs on diploid organisms.

Cycle repeats

B - Gamete Probabilities

Here we give an overview of the derivation of the equations related to the probability of gametes being passed on. There is a probability of generating a given gamete for each of the thirty six possible genotypes. For convenience, we label each of the 8 possible gametes 1-8 and the genotypes 1-36. The 8 possible gametes are JtA (1), JtB (2), JTA (3), JTB (4), jtA (5), jtB (6), jTA (7), and jTB (8). In terms of general notation, $p_{X,b,c,d,w,m,n}$ is the probability of generating gamete X and having initial genotype bcd/wmn. Importantly, in the section on mating (i.e. see sections C1 “Selfing” and C2 “Random Mating”) the same notation (i.e. $p_{X,b,c,d,w,m,n}$) now represents the probability of generating gamete X given the initial genotype bcd/wmn. The reason for this was due to technical convenience when using the Mathematica software. The thirty six possible genotypes are:

Possible Genotypes	Number
JtA/JtA	1
JtA/JtB	2
JtA/JTA	3
JtA/JTB	4
JtB/JtB	5
JtB/JTA	6
JtB/JTB	7
JTA/JTA	8
JTA/JTB	9
JTB/JTB	10
JtA/jta	11
JtA/jtB	12
jtA/JtB	13
JtA/jTA	14
jtA/JTA	15
JtA/jTB	16
jtA/JTB	17
JtB/jtB	18
jtB/JTA	19
JtB/jTA	20
JtB/jTB	21
jtB/JTB	22
JTA/jTA	23
JTA/jTB	24
jTA/JTB	25
JTB/jTB	26
jtA/jtA	27

jtA / jtB	28
jtA / jTA	29
jtA / jTB	30
jtB / jtB	31
jtB / jTA	32
jtB / jTB	33
jTA / jTA	34
jTA / jTB	35
jTB / jTB	36

Using genotype 1 as an example, the first event is epimutation. Two chromosomes are paired with each other and undergo epimutation (independently) just prior to recombination occurring between them. Noting that genotype 1 is JtA/JtA (with frequency $p_{J,t,A,J,t,A}$).This event generates each of the gametes with a given probability through epimutation, with the total sum equaling the initial frequency of the genotype.

$$p_{1,J,t,A,J,t,A}{}^{(1)} = p_{J,t,A,J,t,A}\,(1-\alpha_1)\,(1-\alpha_1) + p_{J,t,A,J,t,A}\,(1-\alpha_1)\,\alpha_1$$

$$p_{2,J,t,A,J,t,A}{}^{(1)} = p_{J,t,A,J,t,A}\,\alpha_1\,\alpha_1 + p_{J,t,A,J,t,A}\,(1-\alpha_1)\,\alpha_1$$

$$p_{3,J,t,A,J,t,A}{}^{(1)} = 0$$

$$p_{4,J,t,A,J,t,A}{}^{(1)} = 0$$

$$p_{5,J,t,A,J,t,A}{}^{(1)} = 0$$

$$p_{6,J,t,A,J,t,A}{}^{(2)} = 0$$

$$p_{7,J,t,A,J,t,A}{}^{(1)} = 0$$

$$p_{8,J,t,A,J,t,A}{}^{(1)} = 0$$

Where

$$p_{J,t,A,J,t,A} = p_{1,J,t,A,J,t,A}{}^{(1)} + p_{2,J,t,A,J,t,A}{}^{(1)} + p_{3,J,t,A,J,t,A}{}^{(1)} + p_{4,J,t,A,J,t,A}{}^{(1)} + p_{5,J,t,A,J,t,A}{}^{(1)} + p_{6,J,t,A,J,t,A}{}^{(1)} + p_{7,J,t,A,J,t,A}{}^{(1)} + p_{8,J,t,A,J,t,A}{}^{(1)}$$

Specifically, for example, the first term on the second line (i.e. $p_{J,t,A,J,t,A}\,\alpha_1\,\alpha_1$) refers to both haplotypes undergoing epimutation prior to being paired with each other. Therefore this term contributes to the probability of generating the JtB haplotype (which corresponds to the subscript ‘2’ in the term $p_{2,J,t,A,J,t,A}{}^{(1)}$).

Next, recombination occurs after epimutation but in this case recombination does not impact the probability of a gamete being generated given the initial genotype (JtA/JtA).

$$p_{1,J,t,A,J,t,A}{}^{(2)} = p_{J,t,A,J,t,A}\,(1-\alpha_1)\,(1-\alpha_1) + p_{J,t,A,J,t,A}\,(1-\alpha_1)\,\alpha_1$$

$p_{2,J,t,A,J,t,A}{}^{(2)} = p_{J,t,A,J,t,A}\,\alpha_1\,\alpha_1 + p_{J,t,A,J,t,A}\,(1-\alpha_1)\,\alpha_1$

$p_{3,J,t,A,J,t,A}{}^{(2)} = 0$

$p_{4,J,t,A,J,t,A}{}^{(2)} = 0$

$p_{5,J,t,A,J,t,A}{}^{(2)} = 0$

$p_{6,J,t,A,J,t,A}{}^{(2)} = 0$

$p_{7,J,t,A,J,t,A}{}^{(2)} = 0$

$p_{8,J,t,A,J,t,A}{}^{(2)} = 0$

Transposition/deletion occurs on the gametes post recombination

$p_{1,J,t,A,J,t,A}{}^{(3)} = (1-z)\,(p_{1\,|\,J,t,A,J,t,A}{}^{(2)})$

$p_{2,J,t,A,J,t,A}{}^{(3)} = (1-z)\,(p_{2\,|\,J,t,A,J,t,A}{}^{(2)})$

$p_{3,J,t,A,J,t,A}{}^{(3)} = z\,(p_{1\,|\,J,t,A,J,t,A}{}^{(2)})$

$p_{4,J,t,A,J,t,A}{}^{(3)} = z\,(p_{2\,|\,J,t,A,J,t,A}{}^{(2)})$

$p_{5,J,t,A,J,t,A}{}^{(3)} = 0$

$p_{6,J,t,A,J,t,A}{}^{(3)} = 0$

$p_{7,J,t,A,J,t,A}{}^{(3)} = 0$

$p_{8,J,t,A,J,t,A}{}^{(3)} = 0$

For example, observing the term on the third line (i.e. $z\,(p_{1,J,t,A,J,t,A}{}^{(2)})$), this is the probability that what is currently a JtA haplotype becomes a JTA haplotype through transposition. Noting the probability remains zero for generating any of the haplotypes with a ‘j’ allele because we do not allow mutation at the modifier locus, and the initial genotype (JtA/JtA) does not have a ‘j’ allele.

After substituting each term into the consecutive term, we obtain the final probabilities of generating each of the gametes given the initial JtA/JtA genotype.

$p_{1,J,t,A,J,t,A}{}^{(3)} = (1-z)\left(p_{J,t,A,J,t,A}\,(1-\alpha_1)\,(1-\alpha_1) + p_{J,t,A,J,t,A}\,(1-\alpha_1)\,\alpha_1\right)$

$$p_{2,J,t,A,J,t,A}{}^{(3)} = (1-z)\left(p_{J,t,A,J,t,A}\,\alpha_1\,\alpha_1 + p_{J,t,A,J,t,A}\,(1-\alpha_1)\,\alpha_1\right)$$

$$p_{3,J,t,A,J,t,A}{}^{(3)} = z\left(p_{J,t,A,J,t,A}\,(1-\alpha_1)\,(1-\alpha_1) + p_{J,t,A,J,t,A}\,(1-\alpha_1)\,\alpha_1\right)$$

$$p_{4,J,t,A,J,t,A}{}^{(3)} = z\left(p_{J,t,A,J,t,A}\,\alpha_1\,\alpha_1 + p_{J,t,A,J,t,A}\,(1-\alpha_1)\,\alpha_1\right)$$

$$p_{5,J,t,A,J,t,A}{}^{(3)} = 0$$

$$p_{6,J,t,A,J,t,A}{}^{(3)} = 0$$

$$p_{7,J,t,A,J,t,A}{}^{(3)} = 0$$

$$p_{8,J,t,A,J,t,A}{}^{(3)} = 0$$

As a second example to better illustrate the effects of recombination for this model, we use genotype 16 (JtA/jTB, with frequency $p_{J,t,A,j,T,B}$) where recombination between the two chromosomes does have an effect on the probability of generating a particular gamete after epimutation.

The initial event is again epimutation, whereby two chromosomes are paired with each other and undergo epimutation (independently) just prior to recombination occurring between them. After epimutation, transient (epi)genotypes are generated prior to recombination occurring and generating individual gametes.

$$p_{1,J,t,A,j,T,B}{}^{(1)} = \frac{1}{2}\,p_{J,t,A,j,T,B}\,(1-\alpha_1)\,(1-\beta_2) + \frac{1}{2}\,p_{J,t,A,j,T,B}\,(1-\alpha_1)\,\beta_2$$

$$p_{2,J,t,A,j,T,B}{}^{(1)} = \frac{1}{2}\,p_{J,t,A,j,T,B}\,\alpha_1\,(1-\beta_2) + \frac{1}{2}\,p_{J,t,A,j,T,B}\,\alpha_1\,\beta_2$$

$$p_{3,J,t,A,j,T,B}{}^{(1)} = 0$$

$$p_{4,J,t,A,j,T,B}{}^{(1)} = 0$$

$$p_{5,J,t,A,j,T,B}{}^{(1)} = 0$$

$$p_{6,J,t,A,j,T,B}{}^{(1)} = 0$$

$$p_{7,J,t,A,j,T,B}{}^{(1)} = \frac{1}{2}\,p_{J,t,A,j,T,B}\,(1-\alpha_1)\,\beta_2 + \frac{1}{2}\,p_{J,t,A,j,T,B}\,\alpha_1\,\beta_2$$

$$p_{8,J,t,A,j,T,B}{}^{(1)} = \frac{1}{2}\,p_{J,t,A,j,T,B}\,(1-\alpha_1)\,(1-\beta_2) + \frac{1}{2}\,p_{J,t,A,j,T,B}\,\alpha_1\,(1-\beta_2)$$

For example, going through the probability of generating gamete ‘JtA’ given this genotype which is the first equation in

above set. I.e.

$$p_{1,J,t,A,j,T,B}{}^{(1)} = \frac{1}{2}\, p_{J,t,A,j,T,B}\,(1-\alpha_1)\,(1-\beta_2) + \frac{1}{2}\, p_{J,t,A,j,T,B}\,(1-\alpha_1)\,\beta_2$$

After epimutation, two genotypes are generated that can generate the JtA gamete. The first term on the right ($\frac{1}{2}\, p_{J,t,A,j,T,B}\,(1-\alpha_1)\,(1-\beta_2)$) indicates the initial 'genotype' did not change (i.e. it remained JtA/jTB), where the 1/2 indicates the number of chromosomes that carry the JtA gamete/haplotype. The second term ($\frac{1}{2}\, p_{J,t,A,j,T,B}\,(1-\alpha_1)\,\beta_2$) represents the genotype JtA/jTA since the epiallele has been reverted, where again the 1/2 indicates the number of chromosomes that carry the JtA haplotype just prior to recombination.

The next event is recombination, which occurs between the two paired chromosomes where the probability of recombination between the 'J/j' and 't/T' locus is r3 and the probability of recombination in between the 't/T' and 'A/B' locus is r4, since J/j is the genotype at the modifier locus. The gamete probabilities after recombination are

$$p_{1,J,t,A,j,T,B}{}^{(2)} = (1-r3)\,(1-r4)\left(\frac{1}{2}\, p_{J,t,A,j,T,B}\,(1-\alpha_1)\,(1-\beta_2)\right) + (1-r3)\,\frac{1}{2}\, p_{J,t,A,j,T,B}\,(1-\alpha_1)\,\beta_2 + (1-r3)\,r4\,\frac{1}{2}\, p_{J,t,A,j,T,B}\,\alpha_1\,\beta_2$$

$$p_{2,J,t,A,j,T,B}{}^{(2)} = (1-r3)\,\frac{1}{2}\, p_{J,t,A,j,T,B}\,\alpha_1\,(1-\beta_2) + (1-r3)\,(1-r4)\,\frac{1}{2}\, p_{J,t,A,j,T,B}\,\alpha_1\,\beta_2 + (1-r3)\,r4\left(\frac{1}{2}\, p_{J,t,A,j,T,B}\,(1-\alpha_1)\,(1-\beta_2)\right)$$

$$p_{3,J,t,A,j,T,B}{}^{(2)} = r3\,r4\left(\frac{1}{2}\, p_{J,t,A,j,T,B}\,(1-\alpha_1)\,(1-\beta_2)\right) + r3\,(1-r4)\,\frac{1}{2}\, p_{J,t,A,j,T,B}\,\alpha_1\,\beta_2 + r3\,\frac{1}{2}\, p_{J,t,A,j,T,B}\,(1-\alpha_1)\,\beta_2$$

$$p_{4,J,t,A,j,T,B}{}^{(2)} = r3\,(1-r4)\left(\frac{1}{2}\, p_{J,t,A,j,T,B}\,(1-\alpha_1)\,(1-\beta_2)\right) + r3\,\frac{1}{2}\, p_{J,t,A,j,T,B}\,\alpha_1\,(1-\beta_2) + r3\,r4\,\frac{1}{2}\, p_{J,t,A,j,T,B}\,\alpha_1\,\beta_2$$

$$p_{5,J,t,A,j,T,B}{}^{(2)} = r3\,\frac{1}{2}\, p_{J,t,A,j,T,B}\,(1-\alpha_1)\,\beta_2 + r3\,r4\,\frac{1}{2}\, p_{J,t,A,j,T,B}\,\alpha_1\,\beta_2 + r3\,(1-r4)\,\frac{1}{2}\, p_{J,t,A,j,T,B}\,(1-\alpha_1)\,(1-\beta_2)$$

$$p_{6,J,t,A,j,T,B}{}^{(2)} = r3\,(1-r4)\,\frac{1}{2}\, p_{J,t,A,j,T,B}\,\alpha_1\,\beta_2 + r3\,r4\,\frac{1}{2}\, p_{J,t,A,j,T,B}\,(1-\alpha_1)\,(1-\beta_2) + r3\,\frac{1}{2}\, p_{J,t,A,j,T,B}\,\alpha_1\,(1-\beta_2)$$

$$p_{7,J,t,A,j,T,B}{}^{(2)} = (1-r3)\,\frac{1}{2}\, p_{J,t,A,j,T,B}\,(1-\alpha_1)\,\beta_2 + (1-r3)\,(1-r4)\,\frac{1}{2}\, p_{J,t,A,j,T,B}\,\alpha_1\,\beta_2 + (1-r3)\,r4\,\frac{1}{2}\, p_{J,t,A,j,T,B}\,(1-\alpha_1)\,(1-\beta_2)$$

$$p_{8,J,t,A,j,T,B}{}^{(2)} = (1-r3)\,(1-r4)\,\frac{1}{2}\, p_{J,t,A,j,T,B}\,(1-\alpha_1)\,(1-\beta_2) + (1-r3)\,\frac{1}{2}\, p_{J,t,A,j,T,B}\,\alpha_1\,(1-\beta_2) + (1-r3)\,r4\,\frac{1}{2}\, p_{J,t,A,j,T,B}\,\alpha_1\,\beta_2$$

Focusing on the probability of generating gamete JtA after recombination as an example:

$$p_{1,J,t,A,j,T,B}{}^{(2)} = (1-r3)\,(1-r4)\left(\frac{1}{2}\, p_{J,t,A,j,T,B}\,(1-\alpha_1)\,(1-\beta_2)\right) + (1-r3)\,\frac{1}{2}\, p_{J,t,A,j,T,B}\,(1-\alpha_1)\,\beta_2 + (1-r3)\,r4\,\frac{1}{2}\, p_{J,t,A,j,T,B}\,\alpha_1\,\beta_2$$

Beginning with the first term $\left((1-r3)(1-r4)\left(\frac{1}{2}\, p_{J,t,A,j,T,B}\,(1-\alpha_1)(1-\beta_2)\right)\right)$, this is the chance that neither epimutation nor any recombination took place, such that the initial genotype (did not change after the recombination step). The second term $(1-r3)\,\frac{1}{2}\, p_{J,t,A,j,T,B}\,(1-\alpha_1)\,\beta_2$ represents a case where recombination does not change the genotype that arose after epimutation (i.e. JtA/jTA) since recombination did not happen in between the 'J/j' and 't/T' locus (ie. 1 - r3). For the third term $((1-r3)\, r4\, \frac{1}{2}\, p_{J,t,A,j,T,B}\,\alpha_1\,\beta_2)$ this indicates that after epimutation the genotype is JtB/jTA and then if recombination does not occur at the first site (1 - r3) but does occur at the second site (r4) then this generates the gamete (JtA) at one chromosome.

The next event is transposition/deletion of the gametes post recombination.

$$p_{1,J,t,A,j,T,B}{}^{(3)} = (1-z)\, p_{1,J,t,A,j,T,B}{}^{(2)} + y\, p_{3,J,t,A,j,T,B}{}^{(2)}$$

$$p_{2,J,t,A,j,T,B}{}^{(3)} = (1-z)\, p_{2,J,t,A,j,T,B}{}^{(2)} + y\, p_{4,J,t,A,j,T,B}{}^{(2)}$$

$$p_{3,J,t,A,j,T,B}{}^{(3)} = z\, p_{1,J,t,A,j,T,B}{}^{(2)} + (1-y)\, p_{3,J,t,A,j,T,B}{}^{(2)}$$

$$p_{4,J,t,A,j,T,B}{}^{(3)} = z\, p_{2,J,t,A,j,T,B}{}^{(2)} + (1-y)\, p_{4,J,t,A,j,T,B}{}^{(2)}$$

$$p_{5,J,t,A,j,T,B}{}^{(3)} = (1-z)\, p_{5,J,t,A,j,T,B}{}^{(2)} + y\, p_{7,J,t,A,j,T,B}{}^{(2)}$$

$$p_{6,J,t,A,j,T,B}{}^{(3)} = (1-z)\, p_{6,J,t,A,j,T,B}{}^{(2)} + y\, p_{8,J,t,A,j,T,B}{}^{(2)}$$

$$p_{7,J,t,A,j,T,B}{}^{(3)} = z\, p_{5,J,t,A,j,T,B}{}^{(2)} + (1-y)\, p_{7,J,t,A,j,T,B}{}^{(2)}$$

$$p_{8,J,t,A,j,T,B}{}^{(3)} = z\, p_{6,J,t,A,j,T,B}{}^{(2)} + (1-y)\, p_{8,J,t,A,j,T,B}{}^{(2)}$$

The final gamete probabilities generated by the initial genotype are derived by substituting each event into the consecutive event. The equations are

$$p_{1,J,t,A,j,T,B}{}^{(3)} = (1-z)\left((1-r3)(1-r4)\left(\frac{1}{2}\, p_{J,t,A,j,T,B}\,(1-\alpha_1)(1-\beta_2)\right) + (1-r3)\,\frac{1}{2}\, p_{J,t,A,j,T,B}\,(1-\alpha_1)\,\beta_2 + (1-r3)\, r4\,\frac{1}{2}\, p_{J,t,A,j,T,B}\,\alpha_1\,\beta_2\right) + y\left(r3\, r4\left(\frac{1}{2}\, p_{J,t,A,j,T,B}\,(1-\alpha_1)(1-\beta_2)\right) + r3\,(1-r4)\,\frac{1}{2}\, p_{J,t,A,j,T,B}\,\alpha_1\,\beta_2 + r3\,\frac{1}{2}\, p_{J,t,A,j,T,B}\,(1-\alpha_1)\,\beta_2\right)$$

$$p_{2,J,t,A,j,T,B}{}^{(3)} = (1-z)\left((1-r3)\,\frac{1}{2}\, p_{J,t,A,j,T,B}\,\alpha_1\,(1-\beta_2) + (1-r3)(1-r4)\,\frac{1}{2}\, p_{J,t,A,j,T,B}\,\alpha_1\,\beta_2 + (1-r3)\, r4\left(\frac{1}{2}\, p_{J,t,A,j,T,B}\,(1-\alpha_1)(1-\beta_2)\right)\right) + y\left(r3\,(1-r4)\left(\frac{1}{2}\, p_{J,t,A,j,T,B}\,(1-\alpha_1)(1-\beta_2)\right) + r3\,\frac{1}{2}\, p_{J,t,A,j,T,B}\,\alpha_1\,(1-\beta_2) + r3\, r4\,\frac{1}{2}\, p_{J,t,A,j,T,B}\,\alpha_1\,\beta_2\right)$$

$$p_{3,J,t,A,j,T,B}{}^{(3)} = z\left((1-r3)(1-r4)\left(\frac{1}{2}\,p_{J,t,A,j,T,B}(1-\alpha_1)(1-\beta_2)\right)+(1-r3)\,\frac{1}{2}\,p_{J,t,A,j,T,B}(1-\alpha_1)\,\beta_2+(1-r3)\,r4\,\frac{1}{2}\,p_{J,t,A,j,T,B}\,\alpha_1\,\beta_2\right)+ (1-y)\left(r3\,r4\left(\frac{1}{2}\,p_{J,t,A,j,T,B}(1-\alpha_1)(1-\beta_2)\right)+r3\,(1-r4)\,\frac{1}{2}\,p_{J,t,A,j,T,B}\,\alpha_1\,\beta_2+r3\,\frac{1}{2}\,p_{J,t,A,j,T,B}(1-\alpha_1)\,\beta_2\right)$$

$$p_{4,J,t,A,j,T,B}{}^{(3)} = z\left((1-r3)\,\frac{1}{2}\,p_{J,t,A,j,T,B}\,\alpha_1\,(1-\beta_2)+(1-r3)(1-r4)\,\frac{1}{2}\,p_{J,t,A,j,T,B}\,\alpha_1\,\beta_2+(1-r3)\,r4\left(\frac{1}{2}\,p_{J,t,A,j,T,B}(1-\alpha_1)(1-\beta_2)\right)\right)+ (1-y)\left(r3\,(1-r4)\left(\frac{1}{2}\,p_{J,t,A,j,T,B}(1-\alpha_1)(1-\beta_2)\right)+r3\,\frac{1}{2}\,p_{J,t,A,j,T,B}\,\alpha_1\,(1-\beta_2)+r3\,r4\,\frac{1}{2}\,p_{J,t,A,j,T,B}\,\alpha_1\,\beta_2\right)$$

$$p_{5,J,t,A,j,T,B}{}^{(3)} = (1-z)\left(r3\,\frac{1}{2}\,p_{J,t,A,j,T,B}(1-\alpha_1)\,\beta_2+r3\,r4\,\frac{1}{2}\,p_{J,t,A,j,T,B}\,\alpha_1\,\beta_2+r3\,(1-r4)\,\frac{1}{2}\,p_{J,t,A,j,T,B}(1-\alpha_1)(1-\beta_2)\right)+ y\left((1-r3)\,\frac{1}{2}\,p_{J,t,A,j,T,B}(1-\alpha_1)\,\beta_2+(1-r3)(1-r4)\,\frac{1}{2}\,p_{J,t,A,j,T,B}\,\alpha_1\,\beta_2+(1-r3)\,r4\,\frac{1}{2}\,p_{J,t,A,j,T,B}(1-\alpha_1)(1-\beta_2)\right)$$

$$p_{6,J,t,A,j,T,B}{}^{(3)} = (1-z)\left(r3\,(1-r4)\,\frac{1}{2}\,p_{J,t,A,j,T,B}\,\alpha_1\,\beta_2+r3\,r4\,\frac{1}{2}\,p_{J,t,A,j,T,B}(1-\alpha_1)(1-\beta_2)+r3\,\frac{1}{2}\,p_{J,t,A,j,T,B}\,\alpha_1\,(1-\beta_2)\right)+ y\left((1-r3)(1-r4)\,\frac{1}{2}\,p_{J,t,A,j,T,B}(1-\alpha_1)(1-\beta_2)+(1-r3)\,\frac{1}{2}\,p_{J,t,A,j,T,B}\,\alpha_1\,(1-\beta_2)+(1-r3)\,r4\,\frac{1}{2}\,p_{J,t,A,j,T,B}\,\alpha_1\,\beta_2\right)$$

$$p_{7,J,t,A,j,T,B}{}^{(3)} = z\left(r3\,\frac{1}{2}\,p_{J,t,A,j,T,B}(1-\alpha_1)\,\beta_2+r3\,r4\,\frac{1}{2}\,p_{J,t,A,j,T,B}\,\alpha_1\,\beta_2+r3\,(1-r4)\,\frac{1}{2}\,p_{J,t,A,j,T,B}(1-\alpha_1)(1-\beta_2)\right)+ (1-y)\left((1-r3)\,\frac{1}{2}\,p_{J,t,A,j,T,B}(1-\alpha_1)\,\beta_2+(1-r3)(1-r4)\,\frac{1}{2}\,p_{J,t,A,j,T,B}\,\alpha_1\,\beta_2+(1-r3)\,r4\,\frac{1}{2}\,p_{J,t,A,j,T,B}(1-\alpha_1)(1-\beta_2)\right)$$

$$p_{8,J,t,A,j,T,B}{}^{(3)} = z\left(r3\,(1-r4)\,\frac{1}{2}\,p_{J,t,A,j,T,B}\,\alpha_1\,\beta_2+r3\,r4\,\frac{1}{2}\,p_{J,t,A,j,T,B}(1-\alpha_1)(1-\beta_2)+r3\,\frac{1}{2}\,p_{J,t,A,j,T,B}\,\alpha_1\,(1-\beta_2)\right)+ (1-y)\left((1-r3)(1-r4)\,\frac{1}{2}\,p_{J,t,A,j,T,B}(1-\alpha_1)(1-\beta_2)+(1-r3)\,\frac{1}{2}\,p_{J,t,A,j,T,B}\,\alpha_1\,(1-\beta_2)+(1-r3)\,r4\,\frac{1}{2}\,p_{J,t,A,j,T,B}\,\alpha_1\,\beta_2\right)$$

The gamete probabilities for each of the genotypes were similarly derived. Supplement 2, Model 4, section A, gives the gamete probabilities for each of the thirty six genotypes.

C - Derivation of Recursion Equations

C1 - Selfing

Diploid individuals undergo selfing, such that all thirty six unique epigenotypes mate with themselves to generate diploid offspring. As a general example, given a genotype undergoes selfing with probability S, the probability of choosing a specific genotype that then undergoes selfing is $Sp_{b,c,d,w,m,n}$ where the subscripts represent a three locus diploid genotype. Both identical genotypes produce a haploid gamete to form a new diploid offspring, but undergo epimutation independently. So the probability any given genotype is formed after a selfing event is $Sp_{b,c,d,w,m,n}p_j p_k$, where p_j is the gamete produced by the first (diploid) genotype and the p_k is the gamete produced by the second (diploid) genotype. Noting that these gamete

probabilities (i.e. p_j and p_k) were derived in the previous section (“Gamete Probabilities”).

As a more concrete example the table below demonstrates the frequency of all possible genotypes generated through the selfing of the JtA/JtA genotype. For a specific case, focusing on the top left of the table, the probability of the JtA/JtA genotype generating the JtA/JtA genotype through selfing is $S\, p_{J,t,A,J,t,A}\, (p_{1,J,t,A,J,t,A})\, (p_{1,J,t,A,J,t,A})$. As a reminder, the subscript ‘1’ corresponds to the JtA gamete such that both gametes produced by the initial genotype is this gamete, which generates a JtA/JtA genotype. Also, as a reminder, the notation $p_{X,b,c,d,w,m,n}$ now represents the probability of generation gamete X given initial genotype bcd/wmn for the mating phase (both selfing and random mating).

Genotype 1 : JtA/JtA

Gametes	JtA	JtB	JTA	JTB	jtA	jtB	jTA	jTB
JtA	$S\, p_{J,t,A,J,t,A}\, (p_{1,J,t,A,J,t,A})\, (p_{1,J,t,A,J,t,A})$	$S\, p_{J,t,A,J,t,A}\, 2\, (p_{1,J,t,A,J,t,A})\, (p_{2,J,t,A,J,t,A})$	$S\, p_{J,t,A,J,t,A}\, 2\, (p_{1,J,t,A,J,t,A})\, (p_{3,J,t,A,J,t,A})$	$S\, p_{J,t,A,J,t,A}\, 2\, (p_{1,J,t,A,J,t,A})\, (p_{4,J,t,A,J,t,A})$	0	0	0	0
JtB	0	$S\, p_{J,t,A,J,t,A}\, (p_{2,J,t,A,J,t,A})\, (p_{2,J,t,A,J,t,A})$	$S\, p_{J,t,A,J,t,A}\, 2\, (p_{2,J,t,A,J,t,A})\, (p_{3,J,t,A,J,t,A})$	$S\, p_{J,t,A,J,t,A}\, 2\, (p_{2,J,t,A,J,t,A})\, (p_{4,J,t,A,J,t,A})$	0	0	0	0
JTA	0	0	$S\, p_{J,t,A,J,t,A}\, (p_{3,J,t,A,J,t,A})\, (p_{3,J,t,A,J,t,A})$	$S\, p_{J,t,A,J,t,A}\, 2\, (p_{3,J,t,A,J,t,A})\, (p_{4,J,t,A,J,t,A})$	0	0	0	0
JTB	0	0	0	$S\, p_{J,t,A,J,t,A}\, (p_{4,J,t,A,J,t,A})\, (p_{4,J,t,A,J,t,A})$	0	0	0	0
jtA	0	0	0	0	0	0	0	0
jtB	0	0	0	0	0	0	0	0
jTA	0	0	0	0	0	0	0	0
jTB	0	0	0	0	0	0	0	0

Similar tables are provided for all thirty six genotypes in Supplement 2, Model 4, section B1.

C2 - Random mating

Random mating of diploid parents is derived by taking a 36 X 36 matrix containing all the unique epigenotypes repeated along the rows and multiplying (the direct product, not the dot product) by the transpose of the same matrix. The gamete probabilities (derived separately in section B, “Gamete Probabilities”) are embedded within the tables accounting for all the possible epigenotypes that can be generated by the gametes produced by both diploid parents during a mating event. To illustrate this, below shows a portion of the matrix calculation

$$\left(\begin{array}{|c|c|c|} p_{J,t,A,J,t,A} \sum_{k=1}^{8} p_{k,J,t,A,J,t,A}\, m_k & p_{J,t,A,J,t,A} \sum_{k=1}^{8} p_{k,J,t,A,J,t,A}\, m_k & \ldots \\ p_{J,t,A,J,t,B} \sum_{k=1}^{8} p_{k,J,t,A,J,t,B}\, m_k & p_{J,t,A,J,t,B} \sum_{k=1}^{8} p_{k,J,t,A,J,t,B}\, m_k & \ldots \\ \ldots & \ldots & \ldots \end{array}\right) \cdot \left(\begin{array}{|c|c|c|} p_{J,t,A,J,t,A} \sum_{l=1}^{8} p_{l,J,t,A,J,t,A}\, m_l & p_{J,t,A,J,t,B} \sum_{l=1}^{8} p_{l,J,t,A,J,t,B}\, m_l & \ldots \\ p_{J,t,A,J,t,A} \sum_{l=1}^{8} p_{l,J,t,A,J,t,A}\, m_l & p_{J,t,A,J,t,B} \sum_{l=1}^{8} p_{l,J,t,A,J,t,B}\, m_l & \ldots \\ \ldots & \ldots & \ldots \end{array}\right)$$

For example multiplying the first position of both matrices together gives all the possible epigenotypes generated by pairing these two parents. The expanded calculation is

In[∘]:=

$$\left(p_{J,t,A,J,t,A} \sum_{k=1}^{8} p_{k,J,t,A,J,t,A}\, m_k\right)\left(p_{J,t,A,J,t,A} \sum_{l=1}^{8} p_{l,J,t,A,J,t,A}\, m_l\right)$$

Out[∘]=

$$p_{J,t,A,J,t,A}^2 \left(m_1\, p_{1,J,t,A,J,t,A} + m_2\, p_{2,J,t,A,J,t,A} + m_3\, p_{3,J,t,A,J,t,A} + m_4\, p_{4,J,t,A,J,t,A} + m_5\, p_{5,J,t,A,J,t,A} + m_6\, p_{6,J,t,A,J,t,A} + m_7\, p_{7,J,t,A,J,t,A} + m_8\, p_{8,J,t,A,J,t,A}\right)^2$$

The probability of a particular diploid parent generating a particular gamete (i.e. $p_{X|b,c,d,w,m,n}$) was then substituted into the table for all such terms (see previous section on gamete probabilities for details). m_i is an indicator variable used to derive the total frequency of each unique diploid offspring after mating . For example, m_1^2 indicates the offspring (epi)genotype

JtA/JtA. Setting $m_1^2 = 1$ after taking the direct product of the above matrices, then setting all other m_i terms to zero, and then summing the total gives the frequency of the JtA/JtA (epi)genotype after random mating (i.e. $p_{J,t,A,J,t,A}$ '). The entire calculation and derivation of the recursion equations for all (epi)genotypes after mating is provided in Supplement 2, Model 4, section B.

C3 -Natural Selection

After mating, diploid individuals undergo natural selection as the final event. Each genotype was assumed to have a unique relative fitness w_i and mean fitness is $\overline{w}$. This is shown below for all genotypes:

$p_{J,t,A,J,t,A}'' = \frac{w_1}{\overline{w}} p_{J,t,A,J,t,A}'$
$p_{J,t,A,J,t,B}'' = \frac{w_2}{\overline{w}} p_{J,t,A,J,t,B}'$
$p_{J,t,A,J,T,A}'' = \frac{w_3}{\overline{w}} p_{J,t,A,J,T,A}'$
$p_{J,t,A,J,T,B}'' = \frac{w_4}{\overline{w}} p_{J,t,A,J,T,B}'$
$p_{J,t,B,J,t,B}'' = \frac{w_5}{\overline{w}} p_{J,t,B,J,t,B}'$
$p_{J,t,B,J,T,A}'' = \frac{w_6}{\overline{w}} p_{J,t,B,J,T,A}'$
$p_{J,t,B,J,T,B}'' = \frac{w_7}{\overline{w}} p_{J,t,B,J,T,B}'$
$p_{J,T,A,J,T,A}'' = \frac{w_8}{\overline{w}} p_{J,T,A,J,T,A}'$
$p_{J,T,A,J,T,B}'' = \frac{w_9}{\overline{w}} p_{J,T,A,J,T,B}'$
$p_{J,T,B,J,T,B}'' = \frac{w_{10}}{\overline{w}} p_{J,T,B,J,T,B}'$
$p_{J,t,A,j,t,A}'' = \frac{w_{11}}{\overline{w}} p_{J,t,A,j,t,A}'$
$p_{J,t,A,j,t,B}'' = \frac{w_{12}}{\overline{w}} p_{J,t,A,j,t,B}'$
$p_{j,t,A,J,t,B}'' = \frac{w_{13}}{\overline{w}} p_{j,t,A,J,t,B}'$
$p_{J,t,A,j,T,A}'' = \frac{w_{14}}{\overline{w}} p_{J,t,A,j,T,A}'$
$p_{j,t,A,J,T,A}'' = \frac{w_{15}}{\overline{w}} p_{j,t,A,J,T,A}'$
$p_{J,t,A,j,T,B}'' = \frac{w_{16}}{\overline{w}} p_{J,t,A,j,T,B}'$
$p_{j,t,A,J,T,B}'' = \frac{w_{17}}{\overline{w}} p_{j,t,A,J,T,B}'$
$p_{J,t,B,j,t,B}'' = \frac{w_{18}}{\overline{w}} p_{J,t,B,j,t,B}'$
$p_{J,t,B,j,T,A}'' = \frac{w_{19}}{\overline{w}} p_{J,t,B,j,T,A}'$
$p_{j,t,B,J,T,A}'' = \frac{w_{20}}{\overline{w}} p_{j,t,B,J,T,A}'$
$p_{j,t,B,J,T,A}'' = \frac{w_{21}}{\overline{w}} p_{j,t,B,J,T,A}'$
$p_{J,t,B,j,T,B}'' = \frac{w_{22}}{\overline{w}} p_{J,t,B,j,T,B}'$
$p_{j,t,B,J,T,B}'' = \frac{w_{23}}{\overline{w}} p_{j,t,B,J,T,B}'$
$p_{J,T,A,j,T,A}'' = \frac{w_{24}}{\overline{w}} p_{J,T,A,j,T,A}'$
$p_{J,T,A,j,T,B}'' = \frac{w_{25}}{\overline{w}} p_{J,T,A,j,T,B}'$
$p_{j,T,A,J,T,B}'' = \frac{w_{26}}{\overline{w}} p_{j,T,A,J,T,B}'$
$p_{J,T,B,j,T,B}'' = \frac{w_{27}}{\overline{w}} p_{J,T,B,j,T,B}'$
$p_{j,t,A,j,t,A}'' = \frac{w_{28}}{\overline{w}} p_{j,t,A,j,t,A}'$

$p_{j,t,A,j,t,B}{}' \frac{w_{29}}{\overline{w}} p_{j,t,A,j,t,B}{}'$
$p_{j,t,A,j,T,A}{}'' = \frac{w_{30}}{\overline{w}} p_{j,t,A,j,T,A}{}'$
$p_{j,t,A,j,T,B}{}'' = \frac{w_{31}}{\overline{w}} p_{j,t,A,j,T,B}{}'$
$p_{j,t,B,j,t,B}{}'' = \frac{w_{32}}{\overline{w}} p_{j,t,B,j,t,B}{}'$
$p_{j,t,B,j,T,A}{}'' = \frac{w_{33}}{\overline{w}} p_{j,t,B,j,T,A}{}'$
$p_{j,t,B,j,T,B}{}'' = \frac{w_{34}}{\overline{w}} p_{j,t,B,j,T,B}{}'$
$p_{j,T,A,j,T,A}{}'' = \frac{w_{35}}{\overline{w}} p_{j,T,A,j,T,A}{}'$
$p_{j,T,A,j,T,B}{}'' = \frac{w_{36}}{\overline{w}} p_{j,T,A,j,T,B}{}'$

where $p_{b,c,d,w,m,n}{}' = \left(p_{b,c,d,w,m,n}{}' \left(\text{from random mating}\right) + p_{b,c,d,w,m,n}{}' \left(\text{from selfing}\right)\right)$

C4 -Final Recursion Equations

The final equation were derived by combining the equations from the selfing and random mating (see previous sections) followed by the natural selection event. The equations were too large to show in their entirety, however the code used to obtain the final equations is provided in File S5, Model 4.
