# Supplementary material for: Multi-locus models of deleterious epimutation-selection balance and the evolution of recombination modifiers
Source: G3 (Bethesda). 2026 Jun 11;16(8):jkag147. doi: 10.1093/g3journal/jkag147 (PMC13439953; doi:10.1093/g3journal/jkag147)
Supplement: Supplementary file 1 [file jkag147_supplementary_data.zip › File_S3_G3-2026-406777.pdf]

Model 3: Two locus haploid mating and diploid selection results

This model assumes selection occurs on diploids, allowing for analysis of the effect of dominance on epimutation-selection balance, with random mating. Specifically, with regards to the life cycle, we assume selection occurs prior to recombination, and acts on diploid (epi)genotypes, otherwise the same order of events and assumptions is identical to model 1 (see figure 3). Mirroring model 1 (from the main text) we consider two cases, one in which the transposon is neutral (case A) and one in which it is directly deleterious (case B).

Case A: The epiallele is deleterious and the transposon is neutral

When selection occurs on diploids, the key additional consideration is the dominance relationship between the epiallele and wild-type allele. We follow the same approximation assumptions of the previous model to obtain interpretable analytical results which also allows for more direct comparisons between the two models. We assume reverse epimutation near a transposon (β_2), forward epimutation without a transposon (α_1), the transposition (z) and transposon deletion rate (y) are all small in magnitude (~ O[ζ]). Similar to results from the previous model, an equilibrium arises where the epiallele and transposons are at a relatively low frequency (eq. 1).

$$\hat{p}_B \approx \frac{(1-h\,k)}{h\,k}\left(r\,z\left(\frac{1}{h\,k+\beta_1(1-h\,k)}-1\right)\right)+(1-h\,k)\left(\frac{z}{h\,k}+\frac{\alpha_1}{h\,k+(1-h\,k)\,\beta_1}\right)+O[\zeta^2]$$

$$\hat{p}_T \approx z\left(\frac{1}{h\,k}+\frac{1}{\alpha_2}-1+\frac{r(1-h\,k)(1-\alpha_2)}{h\,k\,\alpha_2}\right)+O[\zeta^2]$$

(1)

Here the net effect of recombination is to increase the epiallele equilibrium frequency, similar to model 1 (case A, from the main text). As the reversion rate (β_1) increases, the positive effect of recombination on the frequency of the epiallele is reduced and with complete resetting when the transposon is not adjacent (β_1 = 1), recombination has no effect on the epiallele frequency at equilibrium. Mechanistically, when a recombination event occurs between a tA and a TB haplotype, the epiallele will be immediately lost (when β_1 = 1) and although the newly positioned wild-type is also now more likely to epimutate, this is cancelled out by the loss of the epiallele and the net effect is to prevent any impact of recombination on the epiallele. Separately, generally as the degree of dominance of the epiallele increases, this decreases the effect of recombination on the epiallele. This may be simply due to epialleles being more visible to selection, where the positive effect of recombination on the epiallele through increasing the effective forward spontaneous epimutation rate is partly

mitigated by less heterozygous masking of the epialleles generated by this process.

Next, focusing on the transposon equilibrium frequency, and observing the numerator (for the last term in the brackets), recombination always has an increasing effect on the transposon here by decoupling it from the deleterious epiallele. As dominance (h) or increases, recombination has a reduced effect on the transposon equilibrium frequency. Despite recombination decoupling the transposon from the deleterious epiallele, increased dominance means that the newly generated TA haplotypes would be more negatively affected by genotypic pairing with epialleles, partly mitigating the increasing effect of recombination on the transposon at equilibrium. As increases, the decoupling of the transposon and epiallele through recombination is partly mitigated by the higher probability of the newly position wild-type adjacent to a transposon undergoing epimutation.

Case B: The transposon is deleterious and the epiallele is weakly deleterious

Next, considering the context where the transposon is deleterious and the epiallele is weakly deleterious (k ~ O[ζ])and otherwise the same approximation assumptions were used as previous cases. One equilibrium arises with very similar properties to eq. 2 (model 1, Case B), with the key additional factor being the dominance relationship of the transposon when paired with an empty transposon site.

$$\hat{p}_B \approx \frac{\alpha_1}{\beta_1} + \frac{r\, z\, \alpha_2 (1 - h\, \mathcal{L})(1 - \beta_1) + z(1 - h\, \mathcal{L})\, \alpha_2\, \beta_1}{h\, \mathcal{L}\, \beta_1 \left(h\, \mathcal{L} + (1 - h\, \mathcal{L})\, \alpha_2 + r(1 - h\, \mathcal{L})(1 - \alpha_2)\right)} + O[\zeta^2]$$

$$\hat{p}_T \approx \frac{z}{h\, \mathcal{L}} + O[\zeta^2]$$

(2)

Similar to eq. 2 from the main text, the net effect of recombination on the epiallele depends on the balance of selection against the transposon ($\mathcal{L}$), the forward rate adjacent the transposon (α_2), and the reverse rate in the absence of the transposon (β_1). For a transparent example, β_1 as approaches 1 (which is feasible in nature) recombination always decreases the epiallele at equilibrium. With regards to the additional factor of dominance, the epiallele increases with less dominance of the transposon. This is likely a result of its deleterious association with the transposon being masked when paired with a fitter ‘t’ site in a (epi)genotype.

The transposon frequency at equilibrium is identical in state to classic mutation selection balance with incomplete domi-

nance (i.e. z/hs -> u/hs).

Model 4: Two/three locus diploid mating and selection (with selfing) results

This model was derived in order to evaluate the effects of diploid selection with the incorporation of selfing (see File S2, for an overview of the derivation and File S5 for the detailed derivation), to compare and contrast with the haploid and diploid (with random mating) results from earlier models.

In a two locus setting with selfing, we find key parameter interactions were similar in comparison to the analytical results presented in model 1 (main text) and 3 (this file). These models indicated that with a strong asymmetry in epimutation rates (particularly when $\alpha_2 > \beta_1$), whereby transposons cause a relatively high forward rate of a neighbouring genetic locus, leads to recombination being a deleterious process (i.e. a higher frequency of the epiallele at equilibrium). A subset of these results is presented in table 1 below, which demonstrates that a higher recombination rate increases the equilibrium frequency of the deleterious epiallele (for the parameter range examined). This mirrors key analytical findings of model 1 and 3 (see eq. 1 from main text and eq. 1 in this file). However, the reverse occurs and recombination decreases the epiallele when $\alpha_2 < \beta_1$ (see table 3 below), similar to results from the eq. 2 from main text and eq. 2 this file.

The analytical results from model 2 (diploid selection and random mating) gave basic insight into the effect of dominance of the epiallele, such that it is expected to decrease the epiallele as it increases. We find this holds in the two locus setting with selfing (see table 2 and 4 below).

Finally, we sought to evaluate whether a recombination rate modifier could invade in a population, but now in a diploid three locus setting. For the parameter range examined, whereby recombination is expected to be deleterious (mirroring chapter 2, Model 2, main text), we find a relatively low recombination rate modifier can invade a population when $\alpha_2 > \beta_1$ and relatively high recombination rate modifier can invade when $\alpha_2 < \beta_1$ (see table 5 below). Selfing did not have a qualitative effect on invasion conditions (data not shown).

Table 1: Equilibrium frequencies as function of the recombination rate ($\alpha_2 > \beta_1$, two locus diploid selection)

r	$\hat{p}_B$	$\hat{p}_T$
0.00001	0.020644	0.00226813
0.0001	0.0209665	0.00228395
0.001	0.023864	0.00244085

0.01	0.0399277	0.0036754
0.1	0.0731032	0.00739396

Parameter values are: k = 0.001, $\alpha_1 = 10^{-5}$, $\alpha_2 = 0.0005$, $\beta_1 = 0.005$, $\beta_2 = 10^{-5}$, $z = 10^{-7}$, $y = 10^{-5}$, $h = 0.01$, S = 0.01.

Table 2: Equilibrium frequencies as function of the dominance coefficient ($\alpha_2 > \beta_1$, two locus diploid selection)

h	$\hat{p}_B$	$\hat{p}_T$
0	0.0252587	0.00290306
0.25	0.0138786	0.00046507
0.5	0.010311	0.00025514
0.75	0.00822625	0.00017903
0.99	0.00689014	0.00014112

Parameter values are: k = 0.001, $\alpha_1 = 10^{-5}$, $\alpha_2 = 0.0005$, $\beta_1 = 0.005$, $\beta_2 = 10^{-5}$, $z = 10^{-7}$, $y = 10^{-5}$, $r = 0.001$, S = 0.01.

Table 3: Equilibrium frequencies as function of the recombination rate ($\alpha_2 < \beta_1$, two locus diploid selection)

r	$\hat{p}_B$	$\hat{p}_T$
0.00001	0.00520958	0.00353634
0.0001	0.00509826	0.00389836
0.001	0.00438177	0.0061145
0.01	0.00328882	0.00912197
0.1	0.00301132	0.00981449

Parameter values are: k = 0.001, $\alpha_1 = 10^{-5}$, $\alpha_2 = 0.0005$, $\beta_1 = 0.005$, $\beta_2 = 10^{-5}$, $z = 10^{-7}$, $y = 10^{-5}$, h = 0.01, S = 0.01.

Table 4: Equilibrium frequencies as function of the dominance coefficient ($\alpha_2 < \beta_1$, two locus diploid selection)

h	$\hat{p}_B$	$\hat{p}_T$
0	0.00494374	0.00748653
0.25	0.00230948	0.0012213
0.5	0.00203194	0.0007451
0.75	0.00188375	0.00057114
0.99	0.00177966	0.00048382

Parameter values are: k = 0.001, $\alpha_1 = 10^{-5}$, $\alpha_2 = 0.0005$, $\beta_1 = 0.005$, $\beta_2 = 10^{-5}$, $z = 10^{-7}$, $y = 10^{-5}$, $r = 0.001$, S = 0.01.

Table 5: Evaluating invasion of a modifier of recombination (three locus diploid selection)

Low recombination rate modifier	Invades (yes or no)
$\alpha_2 = 0.005 > \beta_1 = 0.0005$	Yes
$\alpha_2 = 0.0005 < \beta_1 = 0.005$	No
High recombination rate modifier	–
$\alpha_2 = 0.005 > \beta_1 = 0.0005$	No
$\alpha_2 = 0.0005 < \beta_1 = 0.005$	Yes

The modifier starting frequency was 0.001. When a low recombination modifier is invading: $r_1 = r_3 = r_5 = 0.05$, $r_2 = 0.05$, $r_4 =$

0.005, r_6 = 0.0005. When a high recombination rate modifier is invading: r_1= r_3= r_5 = 0.05, r_2 = 0.0005, r_4 = 0.005, r_6 = 0.05. Other parameter values are: k = 0.001, $\alpha_1 = 10^{-5}$, $\beta_2 = 10^{-5}$, z = 10^{-7}, y = 10^{-5}, h = 0.01.
