# Supplementary material for: Multi-locus models of deleterious epimutation-selection balance and the evolution of recombination modifiers
Source: G3 (Bethesda). 2026 Jun 11;16(8):jkag147. doi: 10.1093/g3journal/jkag147 (PMC13439953; doi:10.1093/g3journal/jkag147)
Supplement: Supplementary file 1 [file jkag147_supplementary_data.zip › File_S4_G3-2026-406777.pdf]

Model 1: Two locus haploid mating and haploid selection, full derivation and systematic analysis

Derivation of the Recursion Equations

Mating of haploid organisms occurs, conditioned on epimutation (1):

$$p_{t,A}{}^{(1)} = p_{t,A,t,A}{}^{(1)} + \frac{1}{2}\, p_{t,A,t,B}{}^{(1)} + \frac{1}{2}\, p_{t,A,T,A}{}^{(1)} + \frac{1}{2}\, p_{t,A,T,B}{}^{(1)}$$

$$p_{t,B}{}^{(1)} = p_{t,B,t,B}{}^{(1)} + \frac{1}{2}\, p_{t,A,t,B}{}^{(1)} + \frac{1}{2}\, p_{t,B,T,A}{}^{(1)} + \frac{1}{2}\, p_{t,B,T,B}{}^{(1)}$$

$$p_{T,A}{}^{(1)} = p_{T,A,T,A}{}^{(1)} + \frac{1}{2}\, p_{t,A,T,A}{}^{(1)} + \frac{1}{2}\, p_{t,B,T,A}{}^{(1)} + \frac{1}{2}\, p_{T,A,T,B}{}^{(1)}$$

$$p_{T,B}{}^{(1)} = p_{T,B,T,B}{}^{(1)} + \frac{1}{2}\, p_{t,A,T,B}{}^{(1)} + \frac{1}{2}\, p_{t,B,T,B}{}^{(1)} + \frac{1}{2}\, p_{T,A,T,B}{}^{(1)}$$

where

$$p_{t,A,t,A}{}^{(1)} = \Big((1-S)\left(p_{t,A}\,(1-\alpha_1)\,p_{t,A}\,(1-\alpha_1) + p_{t,A}\,(1-\alpha_1)\,p_{t,B}\,\beta_1 + p_{t,B}\,\beta_1\,p_{t,A}\,(1-\alpha_1) + p_{t,B}\,\beta_1\,p_{t,B}\,\beta_1\right) + S\left(p_{t,A}\,(1-\alpha_1)\,(1-\alpha_1) + p_{t,B}\,\beta_1\,\beta_1\right)\Big)$$

$$p_{t,A,t,B}{}^{(1)} = \Big(\Big((1-S)\big(p_{t,A}\,\alpha_1\,p_{t,A}\,(1-\alpha_1) + p_{t,A}\,\alpha_1\,p_{t,B}\,\beta_1 + p_{t,A}\,(1-\alpha_1)\,p_{t,A}\,\alpha_1 + p_{t,A}\,(1-\alpha_1)\,p_{t,B}\,(1-\beta_1) + p_{t,B}\,\beta_1\,p_{t,A}\,\alpha_1 + p_{t,B}\,\beta_1\,p_{t,B}\,(1-\beta_1) + p_{t,B}\,(1-\beta_1)\,p_{t,B}\,\beta_1 + p_{t,B}\,(1-\beta_1)\,p_{t,A}\,(1-\alpha_1)\big) + S\left(2\,p_{t,A}\,(1-\alpha_1)\,\alpha_1 + 2\,p_{t,B}\,(1-\beta_1)\,\beta_1\right)\Big)\Big)$$

$$p_{t,A,T,A}{}^{(1)} = (1-S)\,2\left(p_{t,A}\,(1-\alpha_1)\,p_{T,A}\,(1-\alpha_2) + p_{t,A}\,(1-\alpha_1)\,p_{T,B}\,\beta_2 + p_{t,B}\,\beta_1\,p_{T,A}\,(1-\alpha_2) + p_{t,B}\,\beta_1\,p_{T,B}\,\beta_2\right)$$

$$p_{t,A,T,B}{}^{(1)} = (1-S)\,2\left(p_{t,A}\,(1-\alpha_1)\,p_{T,A}\,\alpha_2 + p_{t,A}\,(1-\alpha_1)\,p_{T,B}\,(1-\beta_2) + p_{t,B}\,\beta_1\,p_{T,A}\,\alpha_2 + p_{t,B}\,\beta_1\,p_{T,B}\,(1-\beta_2)\right)$$

$$p_{t,B,t,B}{}^{(1)} = \Big((1-S)\left(p_{t,A}\,\alpha_1\,p_{t,A}\,\alpha_1 + p_{t,A}\,\alpha_1\,p_{t,B}\,(1-\beta_1) + p_{t,B}\,(1-\beta_1)\,p_{t,B}\,(1-\beta_1) + p_{t,B}\,(1-\beta_1)\,p_{t,A}\,\alpha_1\right) + S\left(p_{t,B}\,(1-\beta_1)\,(1-\beta_1) + p_{t,A}\,\alpha_1\,\alpha_1\right)\Big)$$

$$p_{t,B,T,A}{}^{(1)} = (1-S)\,2\left(p_{t,A}\,\alpha_1\,p_{T,A}\,(1-\alpha_2) + p_{t,A}\,\alpha_1\,p_{T,B}\,\beta_2 + p_{t,B}\,(1-\beta_1)\,p_{T,A}\,(1-\alpha_2) + p_{t,B}\,(1-\beta_1)\,p_{T,B}\,\beta_2\right)$$

$$p_{t,B,T,B}{}^{(1)} = (1-S)\,2\left(p_{t,A}\,\alpha_1\,p_{T,A}\,\alpha_2 + p_{t,A}\,\alpha_1\,p_{T,B}\,(1-\beta_2) + p_{t,B}\,(1-\beta_1)\,p_{T,A}\,\alpha_2 + p_{t,B}\,(1-\beta_1)\,p_{T,B}\,(1-\beta_2)\right)$$

$$p_{T,A,T,A}{}^{(1)} = \Big((1-S)\left((1-\alpha_2)^2\,p_{T,A}^2 + 2\,(1-\alpha_2)\,\beta_2\,p_{T,A}\,p_{T,B} + \beta_2^2\,p_{T,B}^2\right) + S\left(p_{T,A}\,(1-\alpha_2)\,(1-\alpha_2) + p_{T,B}\,\beta_2\,\beta_2\right)\Big)$$

$$p_{T,A,T,B}{}^{(1)} = \Big((1-S)\left(2\,(1-\alpha_2)\,\alpha_2\,p_{T,A}^2 + 2\,(1-\alpha_2)\,(1-\beta_2)\,p_{T,A}\,p_{T,B} + 2\,\alpha_2\,\beta_2\,p_{T,A}\,p_{T,B} + 2\,(1-\beta_2)\,\beta_2\,p_{T,B}^2\right) + S\left(2\,p_{T,A}\,(1-\alpha_2)\,\alpha_2 + 2\,p_{T,B}\,(1-\beta_2)\,\beta_2\right)\Big)$$

$$p_{T,B,T,B}{}^{(1)} = \left((1-S)\left(\alpha_2^2\, p_{T,A}^2 + 2\,\alpha_2\,(1-\beta_2)\,p_{T,A}\,p_{T,B} + (1-\beta_2)^2\,p_{T,B}^2\right) + S\left(p_{T,B}\,(1-\beta_2)\,(1-\beta_2) + p_{T,A}\,\alpha_2\,\alpha_2\right)\right)$$

Recombination occurs as the next event during the transient diploid phase (2).

$$p_{t,A}{}^{(2)} = p_{t,A,t,A}{}^{(1)} + \frac{1}{2}\,p_{t,A,t,B}{}^{(1)} + \frac{1}{2}\,p_{t,A,T,A}{}^{(1)} + (1-r)\,\frac{1}{2}\,p_{t,A,T,B}{}^{(1)} + r\,\frac{1}{2}\,p_{t,B,T,A}{}^{(1)}$$

$$p_{t,B}{}^{(2)} = p_{t,B,t,B}{}^{(1)} + \frac{1}{2}\,p_{t,A,t,B}{}^{(1)} + (1-r)\,\frac{1}{2}\,p_{t,B,T,A}{}^{(1)} + \frac{1}{2}\,p_{t,B,T,B}{}^{(1)} + r\,\frac{1}{2}\,p_{t,A,T,B}{}^{(1)}$$

$$p_{T,A}{}^{(2)} = p_{T,A,T,A}{}^{(1)} + \frac{1}{2}\,p_{t,A,T,A}{}^{(1)} + (1-r)\,\frac{1}{2}\,p_{t,B,T,A}{}^{(1)} + \frac{1}{2}\,p_{T,A,T,B}{}^{(1)} + r\,\frac{1}{2}\,p_{t,A,T,B}{}^{(1)}$$

$$p_{T,B}{}^{(2)} = p_{T,B,T,B}{}^{(1)} + (1-r)\,\frac{1}{2}\,p_{t,A,T,B}{}^{(1)} + \frac{1}{2}\,p_{t,B,T,B}{}^{(1)} + \frac{1}{2}\,p_{T,A,T,B}{}^{(1)} + r\,\frac{1}{2}\,p_{t,B,T,A}{}^{(1)}$$

Natural selection occurs on adult haploid organisms developed from gametes post recombination (3).

$$p_{t,A}{}^{(3)} = p_{t,A}{}^{(2)}\,w_1\left(\frac{1}{\overline{w}}\right)$$

$$p_{t,B}{}^{(3)} = p_{t,B}{}^{(2)}\,w_2\left(\frac{1}{\overline{w}}\right)$$

$$p_{T,A}{}^{(3)} = p_{T,A}{}^{(2)}\,w_3\left(\frac{1}{\overline{w}}\right)$$

$$p_{T,B}{}^{(3)} = p_{T,B}{}^{(2)}\,w_4\left(\frac{1}{\overline{w}}\right)$$

where

$$\overline{w} = p_{t,A}{}^{(2)}\,w_1 + p_{t,B}{}^{(2)}\,w_2 + p_{T,A}{}^{(2)}\,w_3 + p_{T,B}{}^{(2)}\,w_4$$

Transposition and deletion occur as the final step in the life cycle (4).

$$p_{t,A}{}^{(4)} = p_{t,A}{}^{(3)}\,(1-z) + p_{T,A}{}^{(3)}\,y$$

$$p_{t,B}{}^{(4)} = p_{t,B}{}^{(3)}\,(1-z) + p_{T,B}{}^{(3)}\,y$$

$$p_{T,A}{}^{(4)} = p_{T,A}{}^{(3)}\,(1-y) + p_{t,A}{}^{(3)}\,z$$

$$p_{T,B}{}^{(4)} = p_{T,B}{}^{(3)}\,(1-y) + p_{t,B}{}^{(3)}\,z$$

After plugging each event into the consecutive event and then simplifying, the final recursion equations for the haplotype

frequencies are show below:

$$p_{t,A}{}' =$$

$$\Big(y\, w_3 \big((-1+S)\,(-1+\alpha_2)\, p_{T,A}^2 + p_{T,B}\,\big(r\,(-1+S)\,\big((-1+\alpha_1)\, p_{t,A} - \beta_1\, p_{t,B}\big) + \beta_2\,\big(S + (-1+r)\,(-1+S)\, p_{t,A} + (-1+r)\,(-1+S)\, p_{t,B} - (-1+S)\, p_{T,B}\big)\big) + p_{T,A}$$

$$\big(S + p_{t,A} - S\, p_{t,A} - r\,\alpha_1\, p_{t,A} + r\, S\,\alpha_1\, p_{t,A} + p_{t,B} - r\, p_{t,B} - S\, p_{t,B} + r\, S\, p_{t,B} + r\,\beta_1\, p_{t,B} - r\, S\,\beta_1\, p_{t,B} + p_{T,B} -$$

$$S\, p_{T,B} + \beta_2\, p_{T,B} - S\,\beta_2\, p_{T,B} + \alpha_2\,\big(-S - (-1+r)\,(-1+S)\, p_{t,A} - (-1+r)\,(-1+S)\, p_{t,B} - p_{T,B} + S\, p_{T,B}\big)\big)\big) -$$

$$(-1+z)\, w_1\,\big((-1+S)\,(-1+\alpha_1)\, p_{t,A}^2 + p_{t,A}\,\big(S + p_{t,B} - S\, p_{t,B} + \beta_1\, p_{t,B} - S\,\beta_1\, p_{t,B} + p_{T,A} - S\, p_{T,A} - r\,\alpha_2\, p_{T,A} + r\, S\,\alpha_2\, p_{T,A} + p_{T,B} - r\, p_{T,B} -$$

$$S\, p_{T,B} + r\, S\, p_{T,B} + r\,\beta_2\, p_{T,B} - r\, S\,\beta_2\, p_{T,B} + \alpha_1\,\big(-S + (-1+S)\, p_{t,B} - (-1+r)\,(-1+S)\, p_{T,A} - p_{T,B} + r\, p_{T,B} + S\, p_{T,B} - r\, S\, p_{T,B}\big)\big) +$$

$$p_{t,B}\,\big(\beta_1\,\big(S - (-1+S)\, p_{t,B} + (-1+r)\,(-1+S)\, p_{T,A} + p_{T,B} - r\, p_{T,B} - S\, p_{T,B} + r\, S\, p_{T,B}\big) + r\,(-1+S)\,\big((-1+\alpha_2)\, p_{T,A} - \beta_2\, p_{T,B}\big)\big)\big)\Big) \Big/$$

$$\Big(S\, w_3\, p_{T,A} - S\, w_3\,\alpha_2\, p_{T,A} + S\, w_4\,\alpha_2\, p_{T,A} + w_3\, p_{t,A}\, p_{T,A} - S\, w_3\, p_{t,A}\, p_{T,A} - r\, w_3\,\alpha_1\, p_{t,A}\, p_{T,A} + r\, S\, w_3\,\alpha_1\, p_{t,A}\, p_{T,A} +$$

$$r\, w_4\,\alpha_1\, p_{t,A}\, p_{T,A} - r\, S\, w_4\,\alpha_1\, p_{t,A}\, p_{T,A} - w_3\,\alpha_2\, p_{t,A}\, p_{T,A} + r\, w_3\,\alpha_2\, p_{t,A}\, p_{T,A} + S\, w_3\,\alpha_2\, p_{t,A}\, p_{T,A} -$$

$$r\, S\, w_3\,\alpha_2\, p_{t,A}\, p_{T,A} + w_4\,\alpha_2\, p_{t,A}\, p_{T,A} - r\, w_4\,\alpha_2\, p_{t,A}\, p_{T,A} - S\, w_4\,\alpha_2\, p_{t,A}\, p_{T,A} + r\, S\, w_4\,\alpha_2\, p_{t,A}\, p_{T,A} + w_3\, p_{t,B}\, p_{T,A} -$$

$$r\, w_3\, p_{t,B}\, p_{T,A} - S\, w_3\, p_{t,B}\, p_{T,A} + r\, S\, w_3\, p_{t,B}\, p_{T,A} + r\, w_4\, p_{t,B}\, p_{T,A} - r\, S\, w_4\, p_{t,B}\, p_{T,A} - w_3\,\alpha_2\, p_{t,B}\, p_{T,A} +$$

$$r\, w_3\,\alpha_2\, p_{t,B}\, p_{T,A} + S\, w_3\,\alpha_2\, p_{t,B}\, p_{T,A} - r\, S\, w_3\,\alpha_2\, p_{t,B}\, p_{T,A} + w_4\,\alpha_2\, p_{t,B}\, p_{T,A} - r\, w_4\,\alpha_2\, p_{t,B}\, p_{T,A} -$$

$$S\, w_4\,\alpha_2\, p_{t,B}\, p_{T,A} + r\, S\, w_4\,\alpha_2\, p_{t,B}\, p_{T,A} + r\, w_3\,\beta_1\, p_{t,B}\, p_{T,A} - r\, S\, w_3\,\beta_1\, p_{t,B}\, p_{T,A} - r\, w_4\,\beta_1\, p_{t,B}\, p_{T,A} +$$

$$r\, S\, w_4\,\beta_1\, p_{t,B}\, p_{T,A} + w_3\, p_{T,A}^2 - S\, w_3\, p_{T,A}^2 - w_3\,\alpha_2\, p_{T,A}^2 + S\, w_3\,\alpha_2\, p_{T,A}^2 + w_4\,\alpha_2\, p_{T,A}^2 - S\, w_4\,\alpha_2\, p_{T,A}^2 + S\, w_4\, p_{T,B} +$$

$$S\, w_3\,\beta_2\, p_{T,B} - S\, w_4\,\beta_2\, p_{T,B} + r\, w_3\, p_{t,A}\, p_{T,B} - r\, S\, w_3\, p_{t,A}\, p_{T,B} + w_4\, p_{t,A}\, p_{T,B} - r\, w_4\, p_{t,A}\, p_{T,B} - S\, w_4\, p_{t,A}\, p_{T,B} +$$

$$r\, S\, w_4\, p_{t,A}\, p_{T,B} - r\, w_3\,\alpha_1\, p_{t,A}\, p_{T,B} + r\, S\, w_3\,\alpha_1\, p_{t,A}\, p_{T,B} + r\, w_4\,\alpha_1\, p_{t,A}\, p_{T,B} - r\, S\, w_4\,\alpha_1\, p_{t,A}\, p_{T,B} + w_3\,\beta_2\, p_{t,A}\, p_{T,B} -$$

$$r\, w_3\,\beta_2\, p_{t,A}\, p_{T,B} - S\, w_3\,\beta_2\, p_{t,A}\, p_{T,B} + r\, S\, w_3\,\beta_2\, p_{t,A}\, p_{T,B} - w_4\,\beta_2\, p_{t,A}\, p_{T,B} + r\, w_4\,\beta_2\, p_{t,A}\, p_{T,B} + S\, w_4\,\beta_2\, p_{t,A}\, p_{T,B} -$$

$$r\, S\, w_4\,\beta_2\, p_{t,A}\, p_{T,B} + w_4\, p_{t,B}\, p_{T,B} - S\, w_4\, p_{t,B}\, p_{T,B} + r\, w_3\,\beta_1\, p_{t,B}\, p_{T,B} - r\, S\, w_3\,\beta_1\, p_{t,B}\, p_{T,B} - r\, w_4\,\beta_1\, p_{t,B}\, p_{T,B} +$$

$$r\, S\, w_4\,\beta_1\, p_{t,B}\, p_{T,B} + w_3\,\beta_2\, p_{t,B}\, p_{T,B} - r\, w_3\,\beta_2\, p_{t,B}\, p_{T,B} - S\, w_3\,\beta_2\, p_{t,B}\, p_{T,B} + r\, S\, w_3\,\beta_2\, p_{t,B}\, p_{T,B} - w_4\,\beta_2\, p_{t,B}\, p_{T,B} +$$

$$r\, w_4\,\beta_2\, p_{t,B}\, p_{T,B} + S\, w_4\,\beta_2\, p_{t,B}\, p_{T,B} - r\, S\, w_4\,\beta_2\, p_{t,B}\, p_{T,B} + w_3\, p_{T,A}\, p_{T,B} - S\, w_3\, p_{T,A}\, p_{T,B} + w_4\, p_{T,A}\, p_{T,B} - S\, w_4\, p_{T,A}\, p_{T,B} -$$

$$w_3\,\alpha_2\, p_{T,A}\, p_{T,B} + S\, w_3\,\alpha_2\, p_{T,A}\, p_{T,B} + w_4\,\alpha_2\, p_{T,A}\, p_{T,B} - S\, w_4\,\alpha_2\, p_{T,A}\, p_{T,B} + w_3\,\beta_2\, p_{T,A}\, p_{T,B} - S\, w_3\,\beta_2\, p_{T,A}\, p_{T,B} -$$

$$w_4\,\beta_2\, p_{T,A}\, p_{T,B} + S\, w_4\,\beta_2\, p_{T,A}\, p_{T,B} + w_4\, p_{T,B}^2 - S\, w_4\, p_{T,B}^2 + w_3\,\beta_2\, p_{T,B}^2 - S\, w_3\,\beta_2\, p_{T,B}^2 - w_4\,\beta_2\, p_{T,B}^2 + S\, w_4\,\beta_2\, p_{T,B}^2 +$$

$$w_2\,\big((-1+S)\,(-1+\beta_1)\, p_{t,B}^2 + \alpha_1\, p_{t,A}\,\big(S - (-1+S)\, p_{t,A} - (-1+S)\, p_{t,B} + p_{T,A} - r\, p_{T,A} - S\, p_{T,A} + r\, S\, p_{T,A} + p_{T,B} - r\, p_{T,B} - S\, p_{T,B} + r\, S\, p_{T,B}\big) -$$

$$r\,(-1+S)\, p_{t,A}\,\big(\alpha_2\, p_{T,A} - (-1+\beta_2)\, p_{T,B}\big) + p_{t,B}\,\big(S + p_{t,A} - S\, p_{t,A} + p_{T,A} - r\, p_{T,A} - S\, p_{T,A} + r\, S\, p_{T,A} + r\,\alpha_2\, p_{T,A} - r\, S\,\alpha_2\, p_{T,A} + p_{T,B} -$$

$$S\, p_{T,B} - r\,\beta_2\, p_{T,B} + r\, S\,\beta_2\, p_{T,B} + \beta_1\,\big(-S + (-1+S)\, p_{t,A} - (-1+r)\,(-1+S)\, p_{T,A} - p_{T,B} + r\, p_{T,B} + S\, p_{T,B} - r\, S\, p_{T,B}\big)\big)\big) +$$

$$w_1\,\big((-1+S)\,(-1+\alpha_1)\, p_{t,A}^2 + p_{t,A}\,\big(S + p_{t,B} - S\, p_{t,B} + \beta_1\, p_{t,B} - S\,\beta_1\, p_{t,B} + p_{T,A} - S\, p_{T,A} - r\,\alpha_2\, p_{T,A} + r\, S\,\alpha_2\, p_{T,A} + p_{T,B} - r\, p_{T,B} -$$

$$S\, p_{T,B} + r\, S\, p_{T,B} + r\,\beta_2\, p_{T,B} - r\, S\,\beta_2\, p_{T,B} + \alpha_1\,\big(-S + (-1+S)\, p_{t,B} - (-1+r)\,(-1+S)\, p_{T,A} - p_{T,B} + r\, p_{T,B} + S\, p_{T,B} - r\, S\, p_{T,B}\big)\big) +$$

$$p_{t,B}\,\big(\beta_1\,\big(S - (-1+S)\, p_{t,B} + (-1+r)\,(-1+S)\, p_{T,A} + p_{T,B} - r\, p_{T,B} - S\, p_{T,B} + r\, S\, p_{T,B}\big) + r\,(-1+S)\,\big((-1+\alpha_2)\, p_{T,A} - \beta_2\, p_{T,B}\big)\big)\big)\Big)$$

$$p_{t,B}{}' = \Big(y\, w_4\,\big(r\, p_{t,B}\, p_{T,A} - r\, S\, p_{t,B}\, p_{T,A} - r\,\beta_1\, p_{t,B}\, p_{T,A} + r\, S\,\beta_1\, p_{t,B}\, p_{T,A} + S\, p_{T,B} - S\,\beta_2\, p_{T,B} + p_{t,A}\, p_{T,B} - r\, p_{t,A}\, p_{T,B} -$$

$$S\, p_{t,A}\, p_{T,B} + r\, S\, p_{t,A}\, p_{T,B} - \beta_2\, p_{t,A}\, p_{T,B} + r\,\beta_2\, p_{t,A}\, p_{T,B} + S\,\beta_2\, p_{t,A}\, p_{T,B} - r\, S\,\beta_2\, p_{t,A}\, p_{T,B} + p_{t,B}\, p_{T,B} - S\, p_{t,B}\, p_{T,B} -$$

$$r\,\beta_1\, p_{t,B}\, p_{T,B} + r\, S\,\beta_1\, p_{t,B}\, p_{T,B} - \beta_2\, p_{t,B}\, p_{T,B} + r\,\beta_2\, p_{t,B}\, p_{T,B} + S\,\beta_2\, p_{t,B}\, p_{T,B} - r\, S\,\beta_2\, p_{t,B}\, p_{T,B} + p_{T,A}\, p_{T,B} -$$

$$S\, p_{T,A}\, p_{T,B} - \beta_2\, p_{T,A}\, p_{T,B} + S\,\beta_2\, p_{T,A}\, p_{T,B} + p_{T,B}^2 - S\, p_{T,B}^2 - \beta_2\, p_{T,B}^2 + S\,\beta_2\, p_{T,B}^2 + (-1+S)\,\alpha_1\, p_{t,A}\,\big(-r\, p_{T,A} - r\, p_{T,B}\big) +$$

$$\alpha_2\, p_{T,A}\,\big(S + (-1+r)\,(-1+S)\, p_{t,A} + (-1+r)\,(-1+S)\, p_{t,B} + p_{T,A} - S\, p_{T,A} + p_{T,B} - S\, p_{T,B}\big)\big) + (-1+z)\, w_2$$

$$\big(-\big((-1+S)\,(-1+\beta_1)\, p_{t,B}^2\big) + \alpha_1\, p_{t,A}\,\big(-S + (-1+S)\, p_{t,A} + (-1+S)\, p_{t,B} - p_{T,A} + r\, p_{T,A} + S\, p_{T,A} - r\, S\, p_{T,A} - p_{T,B} + r\, p_{T,B} + S\, p_{T,B} - r\, S\, p_{T,B}\big) +$$

$$r\,(-1+S)\, p_{t,A}\,\big(\alpha_2\, p_{T,A} - (-1+\beta_2)\, p_{T,B}\big) - p_{t,B}\,\big(S + p_{t,A} - S\, p_{t,A} + p_{T,A} - r\, p_{T,A} - S\, p_{T,A} + r\, S\, p_{T,A} + r\,\alpha_2\, p_{T,A} - r\, S\,\alpha_2\, p_{T,A} + p_{T,B} -$$

$$S\, p_{T,B} - r\,\beta_2\, p_{T,B} + r\, S\,\beta_2\, p_{T,B} + \beta_1\,\big(-S + (-1+S)\, p_{t,A} - (-1+r)\,(-1+S)\, p_{T,A} - p_{T,B} + r\, p_{T,B} + S\, p_{T,B} - r\, S\, p_{T,B}\big)\big)\big)\Big) \Big/$$

$$\Big(S\, w_3\, p_{T,A} - S\, w_3\,\alpha_2\, p_{T,A} + S\, w_4\,\alpha_2\, p_{T,A} + w_3\, p_{t,A}\, p_{T,A} - S\, w_3\, p_{t,A}\, p_{T,A} - r\, w_3\,\alpha_1\, p_{t,A}\, p_{T,A} + r\, S\, w_3\,\alpha_1\, p_{t,A}\, p_{T,A} +$$

$$r\, w_4\,\alpha_1\, p_{t,A}\, p_{T,A} - r\, S\, w_4\,\alpha_1\, p_{t,A}\, p_{T,A} - w_3\,\alpha_2\, p_{t,A}\, p_{T,A} + r\, w_3\,\alpha_2\, p_{t,A}\, p_{T,A} + S\, w_3\,\alpha_2\, p_{t,A}\, p_{T,A} -$$

$$r\, S\, w_3\,\alpha_2\, p_{t,A}\, p_{T,A} + w_4\,\alpha_2\, p_{t,A}\, p_{T,A} - r\, w_4\,\alpha_2\, p_{t,A}\, p_{T,A} - S\, w_4\,\alpha_2\, p_{t,A}\, p_{T,A} + r\, S\, w_4\,\alpha_2\, p_{t,A}\, p_{T,A} +$$

$$w_3\,p_{t,B}\,p_{T,A}-r\,w_3\,p_{t,B}\,p_{T,A}-S\,w_3\,p_{t,B}\,p_{T,A}+r\,S\,w_3\,p_{t,B}\,p_{T,A}+r\,w_4\,p_{t,B}\,p_{T,A}-$$
$$r\,S\,w_4\,p_{t,B}\,p_{T,A}-w_3\,\alpha_2\,p_{t,B}\,p_{T,A}+r\,w_3\,\alpha_2\,p_{t,B}\,p_{T,A}+S\,w_3\,\alpha_2\,p_{t,B}\,p_{T,A}-r\,S\,w_3\,\alpha_2\,p_{t,B}\,p_{T,A}+$$
$$w_4\,\alpha_2\,p_{t,B}\,p_{T,A}-r\,w_4\,\alpha_2\,p_{t,B}\,p_{T,A}-S\,w_4\,\alpha_2\,p_{t,B}\,p_{T,A}+r\,S\,w_4\,\alpha_2\,p_{t,B}\,p_{T,A}+r\,w_3\,\beta_1\,p_{t,B}\,p_{T,A}-$$
$$r\,S\,w_3\,\beta_1\,p_{t,B}\,p_{T,A}-r\,w_4\,\beta_1\,p_{t,B}\,p_{T,A}+r\,S\,w_4\,\beta_1\,p_{t,B}\,p_{T,A}+w_3\,p_{T,A}^2-S\,w_3\,p_{T,A}^2-w_3\,\alpha_2\,p_{T,A}^2+S\,w_3\,\alpha_2\,p_{T,A}^2+$$
$$w_4\,\alpha_2\,p_{T,A}^2-S\,w_4\,\alpha_2\,p_{T,A}^2+S\,w_4\,p_{T,B}+S\,w_3\,\beta_2\,p_{T,B}-S\,w_4\,\beta_2\,p_{T,B}+r\,w_3\,p_{t,A}\,p_{T,B}-r\,S\,w_3\,p_{t,A}\,p_{T,B}+$$
$$w_4\,p_{t,A}\,p_{T,B}-r\,w_4\,p_{t,A}\,p_{T,B}-S\,w_4\,p_{t,A}\,p_{T,B}+r\,S\,w_4\,p_{t,A}\,p_{T,B}-r\,w_3\,\alpha_1\,p_{t,A}\,p_{T,B}+r\,S\,w_3\,\alpha_1\,p_{t,A}\,p_{T,B}+$$
$$r\,w_4\,\alpha_1\,p_{t,A}\,p_{T,B}-r\,S\,w_4\,\alpha_1\,p_{t,A}\,p_{T,B}+w_3\,\beta_2\,p_{t,A}\,p_{T,B}-r\,w_3\,\beta_2\,p_{t,A}\,p_{T,B}-S\,w_3\,\beta_2\,p_{t,A}\,p_{T,B}+$$
$$r\,S\,w_3\,\beta_2\,p_{t,A}\,p_{T,B}-w_4\,\beta_2\,p_{t,A}\,p_{T,B}+r\,w_4\,\beta_2\,p_{t,A}\,p_{T,B}+S\,w_4\,\beta_2\,p_{t,A}\,p_{T,B}-r\,S\,w_4\,\beta_2\,p_{t,A}\,p_{T,B}+$$
$$w_4\,p_{t,B}\,p_{T,B}-S\,w_4\,p_{t,B}\,p_{T,B}+r\,w_3\,\beta_1\,p_{t,B}\,p_{T,B}-r\,S\,w_3\,\beta_1\,p_{t,B}\,p_{T,B}-r\,w_4\,\beta_1\,p_{t,B}\,p_{T,B}+r\,S\,w_4\,\beta_1\,p_{t,B}\,p_{T,B}+$$
$$w_3\,\beta_2\,p_{t,B}\,p_{T,B}-r\,w_3\,\beta_2\,p_{t,B}\,p_{T,B}-S\,w_3\,\beta_2\,p_{t,B}\,p_{T,B}+r\,S\,w_3\,\beta_2\,p_{t,B}\,p_{T,B}-w_4\,\beta_2\,p_{t,B}\,p_{T,B}+r\,w_4\,\beta_2\,p_{t,B}\,p_{T,B}+$$
$$S\,w_4\,\beta_2\,p_{t,B}\,p_{T,B}-r\,S\,w_4\,\beta_2\,p_{t,B}\,p_{T,B}+w_3\,p_{T,A}\,p_{T,B}-S\,w_3\,p_{T,A}\,p_{T,B}+w_4\,p_{T,A}\,p_{T,B}-S\,w_4\,p_{T,A}\,p_{T,B}-$$
$$w_3\,\alpha_2\,p_{T,A}\,p_{T,B}+S\,w_3\,\alpha_2\,p_{T,A}\,p_{T,B}+w_4\,\alpha_2\,p_{T,A}\,p_{T,B}-S\,w_4\,\alpha_2\,p_{T,A}\,p_{T,B}+w_3\,\beta_2\,p_{T,A}\,p_{T,B}-S\,w_3\,\beta_2\,p_{T,A}\,p_{T,B}-$$
$$w_4\,\beta_2\,p_{T,A}\,p_{T,B}+S\,w_4\,\beta_2\,p_{T,A}\,p_{T,B}+w_4\,p_{T,B}^2-S\,w_4\,p_{T,B}^2+w_3\,\beta_2\,p_{T,B}^2-S\,w_3\,\beta_2\,p_{T,B}^2-w_4\,\beta_2\,p_{T,B}^2+S\,w_4\,\beta_2\,p_{T,B}^2+$$
$$w_2\left((-1+S)\,(-1+\beta_1)\,p_{t,B}^2+\alpha_1\,p_{t,A}\left(S-(-1+S)\,p_{t,A}-(-1+S)\,p_{t,B}+p_{T,A}-r\,p_{T,A}-S\,p_{T,A}+r\,S\,p_{T,A}+p_{T,B}-r\,p_{T,B}-S\,p_{T,B}+r\,S\,p_{T,B}\right)-\right.$$
$$r\,(-1+S)\,p_{t,A}\left(\alpha_2\,p_{T,A}-(-1+\beta_2)\,p_{T,B}\right)+p_{t,B}\left(S+p_{t,A}-S\,p_{t,A}+p_{T,A}-r\,p_{T,A}-S\,p_{T,A}+r\,S\,p_{T,A}+r\,\alpha_2\,p_{T,A}-r\,S\,\alpha_2\,p_{T,A}+p_{T,B}-\right.$$
$$\left.\left.S\,p_{T,B}-r\,\beta_2\,p_{T,B}+r\,S\,\beta_2\,p_{T,B}+\beta_1\left(-S+(-1+S)\,p_{t,A}-(-1+r)\,(-1+S)\,p_{T,A}-p_{T,B}+r\,p_{T,B}+S\,p_{T,B}-r\,S\,p_{T,B}\right)\right)\right)+$$
$$w_1\left((-1+S)\,(-1+\alpha_1)\,p_{t,A}^2+p_{t,A}\left(S+p_{t,B}-S\,p_{t,B}+\beta_1\,p_{t,B}-S\,\beta_1\,p_{t,B}+p_{T,A}-S\,p_{T,A}-r\,\alpha_2\,p_{T,A}+r\,S\,\alpha_2\,p_{T,A}+p_{T,B}-r\,p_{T,B}-\right.\right.$$
$$\left.S\,p_{T,B}+r\,S\,p_{T,B}+r\,\beta_2\,p_{T,B}-r\,S\,\beta_2\,p_{T,B}+\alpha_1\left(-S+(-1+S)\,p_{t,B}-(-1+r)\,(-1+S)\,p_{T,A}-p_{T,B}+r\,p_{T,B}+S\,p_{T,B}-r\,S\,p_{T,B}\right)\right)+$$
$$\left.p_{t,B}\left(\beta_1\left(S-(-1+S)\,p_{t,B}+(-1+r)\,(-1+S)\,p_{T,A}+p_{T,B}-r\,p_{T,B}-S\,p_{T,B}+r\,S\,p_{T,B}\right)+r\,(-1+S)\left((-1+\alpha_2)\,p_{T,A}-\beta_2\,p_{T,B}\right)\right)\right)\Big)\Big)$$

$$p_{T,A}{}'=\left((-1+y)\,w_3\left(-\left((-1+S)\,(-1+\alpha_2)\,p_{T,A}^2\right)+\right.\right.$$
$$p_{T,B}\left(-r\,(-1+S)\left((-1+\alpha_1)\,p_{t,A}-\beta_1\,p_{t,B}\right)+\beta_2\left(-S-(-1+r)\,(-1+S)\,p_{t,A}-(-1+r)\,(-1+S)\,p_{t,B}+(-1+S)\,p_{T,B}\right)\right)-$$
$$p_{T,A}\left(S+p_{t,A}-S\,p_{t,A}-r\,\alpha_1\,p_{t,A}+r\,S\,\alpha_1\,p_{t,A}+p_{t,B}-r\,p_{t,B}-S\,p_{t,B}+r\,S\,p_{t,B}+r\,\beta_1\,p_{t,B}-r\,S\,\beta_1\,p_{t,B}+p_{T,B}-\right.$$
$$\left.\left.S\,p_{T,B}+\beta_2\,p_{T,B}-S\,\beta_2\,p_{T,B}+\alpha_2\left(-S-(-1+r)\,(-1+S)\,p_{t,A}-(-1+r)\,(-1+S)\,p_{t,B}-p_{T,B}+S\,p_{T,B}\right)\right)\right)+$$
$$z\,w_1\left((-1+S)\,(-1+\alpha_1)\,p_{t,A}^2+p_{t,A}\left(S+p_{t,B}-S\,p_{t,B}+\beta_1\,p_{t,B}-S\,\beta_1\,p_{t,B}+p_{T,A}-S\,p_{T,A}-r\,\alpha_2\,p_{T,A}+r\,S\,\alpha_2\,p_{T,A}+p_{T,B}-r\,p_{T,B}-\right.\right.$$
$$\left.S\,p_{T,B}+r\,S\,p_{T,B}+r\,\beta_2\,p_{T,B}-r\,S\,\beta_2\,p_{T,B}+\alpha_1\left(-S+(-1+S)\,p_{t,B}-(-1+r)\,(-1+S)\,p_{T,A}-p_{T,B}+r\,p_{T,B}+S\,p_{T,B}-r\,S\,p_{T,B}\right)\right)+$$
$$\left.\left.p_{t,B}\left(\beta_1\left(S-(-1+S)\,p_{t,B}+(-1+r)\,(-1+S)\,p_{T,A}+p_{T,B}-r\,p_{T,B}-S\,p_{T,B}+r\,S\,p_{T,B}\right)+r\,(-1+S)\left((-1+\alpha_2)\,p_{T,A}-\beta_2\,p_{T,B}\right)\right)\right)\right)\Big/$$
$$\Big(S\,w_3\,p_{T,A}-S\,w_3\,\alpha_2\,p_{T,A}+S\,w_4\,\alpha_2\,p_{T,A}+w_3\,p_{t,A}\,p_{T,A}-S\,w_3\,p_{t,A}\,p_{T,A}-r\,w_3\,\alpha_1\,p_{t,A}\,p_{T,A}+r\,S\,w_3\,\alpha_1\,p_{t,A}\,p_{T,A}+$$
$$r\,w_4\,\alpha_1\,p_{t,A}\,p_{T,A}-r\,S\,w_4\,\alpha_1\,p_{t,A}\,p_{T,A}-w_3\,\alpha_2\,p_{t,A}\,p_{T,A}+r\,w_3\,\alpha_2\,p_{t,A}\,p_{T,A}+S\,w_3\,\alpha_2\,p_{t,A}\,p_{T,A}-$$
$$r\,S\,w_3\,\alpha_2\,p_{t,A}\,p_{T,A}+w_4\,\alpha_2\,p_{t,A}\,p_{T,A}-r\,w_4\,\alpha_2\,p_{t,A}\,p_{T,A}-S\,w_4\,\alpha_2\,p_{t,A}\,p_{T,A}+$$
$$r\,S\,w_4\,\alpha_2\,p_{t,A}\,p_{T,A}+w_3\,p_{t,B}\,p_{T,A}-r\,w_3\,p_{t,B}\,p_{T,A}-S\,w_3\,p_{t,B}\,p_{T,A}+r\,S\,w_3\,p_{t,B}\,p_{T,A}+r\,w_4\,p_{t,B}\,p_{T,A}-$$
$$r\,S\,w_4\,p_{t,B}\,p_{T,A}-w_3\,\alpha_2\,p_{t,B}\,p_{T,A}+r\,w_3\,\alpha_2\,p_{t,B}\,p_{T,A}+S\,w_3\,\alpha_2\,p_{t,B}\,p_{T,A}-r\,S\,w_3\,\alpha_2\,p_{t,B}\,p_{T,A}+$$
$$w_4\,\alpha_2\,p_{t,B}\,p_{T,A}-r\,w_4\,\alpha_2\,p_{t,B}\,p_{T,A}-S\,w_4\,\alpha_2\,p_{t,B}\,p_{T,A}+r\,S\,w_4\,\alpha_2\,p_{t,B}\,p_{T,A}+r\,w_3\,\beta_1\,p_{t,B}\,p_{T,A}-$$
$$r\,S\,w_3\,\beta_1\,p_{t,B}\,p_{T,A}-r\,w_4\,\beta_1\,p_{t,B}\,p_{T,A}+r\,S\,w_4\,\beta_1\,p_{t,B}\,p_{T,A}+w_3\,p_{T,A}^2-S\,w_3\,p_{T,A}^2-w_3\,\alpha_2\,p_{T,A}^2+S\,w_3\,\alpha_2\,p_{T,A}^2+$$
$$w_4\,\alpha_2\,p_{T,A}^2-S\,w_4\,\alpha_2\,p_{T,A}^2+S\,w_4\,p_{T,B}+S\,w_3\,\beta_2\,p_{T,B}-S\,w_4\,\beta_2\,p_{T,B}+r\,w_3\,p_{t,A}\,p_{T,B}-r\,S\,w_3\,p_{t,A}\,p_{T,B}+$$
$$w_4\,p_{t,A}\,p_{T,B}-r\,w_4\,p_{t,A}\,p_{T,B}-S\,w_4\,p_{t,A}\,p_{T,B}+r\,S\,w_4\,p_{t,A}\,p_{T,B}-r\,w_3\,\alpha_1\,p_{t,A}\,p_{T,B}+r\,S\,w_3\,\alpha_1\,p_{t,A}\,p_{T,B}+$$
$$r\,w_4\,\alpha_1\,p_{t,A}\,p_{T,B}-r\,S\,w_4\,\alpha_1\,p_{t,A}\,p_{T,B}+w_3\,\beta_2\,p_{t,A}\,p_{T,B}-r\,w_3\,\beta_2\,p_{t,A}\,p_{T,B}-S\,w_3\,\beta_2\,p_{t,A}\,p_{T,B}+$$
$$r\,S\,w_3\,\beta_2\,p_{t,A}\,p_{T,B}-w_4\,\beta_2\,p_{t,A}\,p_{T,B}+r\,w_4\,\beta_2\,p_{t,A}\,p_{T,B}+S\,w_4\,\beta_2\,p_{t,A}\,p_{T,B}-r\,S\,w_4\,\beta_2\,p_{t,A}\,p_{T,B}+$$
$$w_4\,p_{t,B}\,p_{T,B}-S\,w_4\,p_{t,B}\,p_{T,B}+r\,w_3\,\beta_1\,p_{t,B}\,p_{T,B}-r\,S\,w_3\,\beta_1\,p_{t,B}\,p_{T,B}-r\,w_4\,\beta_1\,p_{t,B}\,p_{T,B}+r\,S\,w_4\,\beta_1\,p_{t,B}\,p_{T,B}+$$
$$w_3\,\beta_2\,p_{t,B}\,p_{T,B}-r\,w_3\,\beta_2\,p_{t,B}\,p_{T,B}-S\,w_3\,\beta_2\,p_{t,B}\,p_{T,B}+r\,S\,w_3\,\beta_2\,p_{t,B}\,p_{T,B}-w_4\,\beta_2\,p_{t,B}\,p_{T,B}+r\,w_4\,\beta_2\,p_{t,B}\,p_{T,B}+$$
$$S\,w_4\,\beta_2\,p_{t,B}\,p_{T,B}-r\,S\,w_4\,\beta_2\,p_{t,B}\,p_{T,B}+w_3\,p_{T,A}\,p_{T,B}-S\,w_3\,p_{T,A}\,p_{T,B}+w_4\,p_{T,A}\,p_{T,B}-S\,w_4\,p_{T,A}\,p_{T,B}-$$
$$w_3\,\alpha_2\,p_{T,A}\,p_{T,B}+S\,w_3\,\alpha_2\,p_{T,A}\,p_{T,B}+w_4\,\alpha_2\,p_{T,A}\,p_{T,B}-S\,w_4\,\alpha_2\,p_{T,A}\,p_{T,B}+w_3\,\beta_2\,p_{T,A}\,p_{T,B}-S\,w_3\,\beta_2\,p_{T,A}\,p_{T,B}-$$

$$w_4\,\beta_2\,p_{T,A}\,p_{T,B}+S\,w_4\,\beta_2\,p_{T,A}\,p_{T,B}+w_4\,p_{T,B}^2-S\,w_4\,p_{T,B}^2+w_3\,\beta_2\,p_{T,B}^2-S\,w_3\,\beta_2\,p_{T,B}^2-w_4\,\beta_2\,p_{T,B}^2+S\,w_4\,\beta_2\,p_{T,B}^2+$$
$$w_2\,\Big((-1+S)\,(-1+\beta_1)\,p_{t,B}^2+\alpha_1\,p_{t,A}\,\big(S-(-1+S)\,p_{t,A}-(-1+S)\,p_{t,B}+p_{T,A}-r\,p_{T,A}-S\,p_{T,A}+r\,S\,p_{T,A}+p_{T,B}-r\,p_{T,B}-S\,p_{T,B}+r\,S\,p_{T,B}\big)-$$
$$r\,(-1+S)\,p_{t,A}\,\big(\alpha_2\,p_{T,A}-(-1+\beta_2)\,p_{T,B}\big)+p_{t,B}\,\big(S+p_{t,A}-S\,p_{t,A}+p_{T,A}-r\,p_{T,A}-S\,p_{T,A}+r\,S\,p_{T,A}+r\,\alpha_2\,p_{T,A}-r\,S\,\alpha_2\,p_{T,A}+p_{T,B}-$$
$$S\,p_{T,B}-r\,\beta_2\,p_{T,B}+r\,S\,\beta_2\,p_{T,B}+\beta_1\,\big(-S+(-1+S)\,p_{t,A}-(-1+r)\,(-1+S)\,p_{T,A}-p_{T,B}+r\,p_{T,B}+S\,p_{T,B}-r\,S\,p_{T,B}\big)\big)\Big)+$$
$$w_1\,\Big((-1+S)\,(-1+\alpha_1)\,p_{t,A}^2+p_{t,A}\,\big(S+p_{t,B}-S\,p_{t,B}+\beta_1\,p_{t,B}-S\,\beta_1\,p_{t,B}+p_{T,A}-S\,p_{T,A}-r\,\alpha_2\,p_{T,A}+r\,S\,\alpha_2\,p_{T,A}+p_{T,B}-r\,p_{T,B}-$$
$$S\,p_{T,B}+r\,S\,p_{T,B}+r\,\beta_2\,p_{T,B}-r\,S\,\beta_2\,p_{T,B}+\alpha_1\,\big(-S+(-1+S)\,p_{t,B}-(-1+r)\,(-1+S)\,p_{T,A}-p_{T,B}+r\,p_{T,B}+S\,p_{T,B}-r\,S\,p_{T,B}\big)\big)+$$
$$p_{t,B}\,\big(\beta_1\,\big(S-(-1+S)\,p_{t,B}+(-1+r)\,(-1+S)\,p_{T,A}+p_{T,B}-r\,p_{T,B}-S\,p_{T,B}+r\,S\,p_{T,B}\big)+r\,(-1+S)\,\big((-1+\alpha_2)\,p_{T,A}-\beta_2\,p_{T,B}\big)\big)\Big)\Big)$$

$$p_{T,B}{}' =$$
$$\Big(-\Big((-1+y)\,w_4\,\big(r\,p_{t,B}\,p_{T,A}-r\,S\,p_{t,B}\,p_{T,A}-r\,\beta_1\,p_{t,B}\,p_{T,A}+r\,S\,\beta_1\,p_{t,B}\,p_{T,A}+S\,p_{T,B}-S\,\beta_2\,p_{T,B}+p_{t,A}\,p_{T,B}-r\,p_{t,A}\,p_{T,B}-S\,p_{t,A}\,p_{T,B}+r\,S\,p_{t,A}\,p_{T,B}-$$
$$\beta_2\,p_{t,A}\,p_{T,B}+r\,\beta_2\,p_{t,A}\,p_{T,B}+S\,\beta_2\,p_{t,A}\,p_{T,B}-r\,S\,\beta_2\,p_{t,A}\,p_{T,B}+p_{t,B}\,p_{T,B}-S\,p_{t,B}\,p_{T,B}-r\,\beta_1\,p_{t,B}\,p_{T,B}+$$
$$r\,S\,\beta_1\,p_{t,B}\,p_{T,B}-\beta_2\,p_{t,B}\,p_{T,B}+r\,\beta_2\,p_{t,B}\,p_{T,B}+S\,\beta_2\,p_{t,B}\,p_{T,B}-r\,S\,\beta_2\,p_{t,B}\,p_{T,B}+p_{T,A}\,p_{T,B}-S\,p_{T,A}\,p_{T,B}-$$
$$\beta_2\,p_{T,A}\,p_{T,B}+S\,\beta_2\,p_{T,A}\,p_{T,B}+p_{T,B}^2-S\,p_{T,B}^2-\beta_2\,p_{T,B}^2+S\,\beta_2\,p_{T,B}^2+(-1+S)\,\alpha_1\,p_{t,A}\,\big(-r\,p_{T,A}-r\,p_{T,B}\big)+$$
$$\alpha_2\,p_{T,A}\,\big(S+(-1+r)\,(-1+S)\,p_{t,A}+(-1+r)\,(-1+S)\,p_{t,B}+p_{T,A}-S\,p_{T,A}+p_{T,B}-S\,p_{T,B}\big)\big)\Big)+$$
$$z\,w_2\,\Big((-1+S)\,(-1+\beta_1)\,p_{t,B}^2+\alpha_1\,p_{t,A}\,\big(S-(-1+S)\,p_{t,A}-(-1+S)\,p_{t,B}+p_{T,A}-r\,p_{T,A}-S\,p_{T,A}+r\,S\,p_{T,A}+p_{T,B}-r\,p_{T,B}-S\,p_{T,B}+r\,S\,p_{T,B}\big)-$$
$$r\,(-1+S)\,p_{t,A}\,\big(\alpha_2\,p_{T,A}-(-1+\beta_2)\,p_{T,B}\big)+p_{t,B}\,\big(S+p_{t,A}-S\,p_{t,A}+p_{T,A}-r\,p_{T,A}-S\,p_{T,A}+r\,S\,p_{T,A}+r\,\alpha_2\,p_{T,A}-r\,S\,\alpha_2\,p_{T,A}+p_{T,B}-$$
$$S\,p_{T,B}-r\,\beta_2\,p_{T,B}+r\,S\,\beta_2\,p_{T,B}+\beta_1\,\big(-S+(-1+S)\,p_{t,A}-(-1+r)\,(-1+S)\,p_{T,A}-p_{T,B}+r\,p_{T,B}+S\,p_{T,B}-r\,S\,p_{T,B}\big)\big)\Big)\Big)\Big)\Big/$$
$$\Big(S\,w_3\,p_{T,A}-S\,w_3\,\alpha_2\,p_{T,A}+S\,w_4\,\alpha_2\,p_{T,A}+w_3\,p_{t,A}\,p_{T,A}-S\,w_3\,p_{t,A}\,p_{T,A}-r\,w_3\,\alpha_1\,p_{t,A}\,p_{T,A}+r\,S\,w_3\,\alpha_1\,p_{t,A}\,p_{T,A}+$$
$$r\,w_4\,\alpha_1\,p_{t,A}\,p_{T,A}-r\,S\,w_4\,\alpha_1\,p_{t,A}\,p_{T,A}-w_3\,\alpha_2\,p_{t,A}\,p_{T,A}+r\,w_3\,\alpha_2\,p_{t,A}\,p_{T,A}+$$
$$S\,w_3\,\alpha_2\,p_{t,A}\,p_{T,A}-r\,S\,w_3\,\alpha_2\,p_{t,A}\,p_{T,A}+w_4\,\alpha_2\,p_{t,A}\,p_{T,A}-r\,w_4\,\alpha_2\,p_{t,A}\,p_{T,A}-$$
$$S\,w_4\,\alpha_2\,p_{t,A}\,p_{T,A}+r\,S\,w_4\,\alpha_2\,p_{t,A}\,p_{T,A}+w_3\,p_{t,B}\,p_{T,A}-r\,w_3\,p_{t,B}\,p_{T,A}-S\,w_3\,p_{t,B}\,p_{T,A}+$$
$$r\,S\,w_3\,p_{t,B}\,p_{T,A}+r\,w_4\,p_{t,B}\,p_{T,A}-r\,S\,w_4\,p_{t,B}\,p_{T,A}-w_3\,\alpha_2\,p_{t,B}\,p_{T,A}+r\,w_3\,\alpha_2\,p_{t,B}\,p_{T,A}+$$
$$S\,w_3\,\alpha_2\,p_{t,B}\,p_{T,A}-r\,S\,w_3\,\alpha_2\,p_{t,B}\,p_{T,A}+w_4\,\alpha_2\,p_{t,B}\,p_{T,A}-r\,w_4\,\alpha_2\,p_{t,B}\,p_{T,A}-S\,w_4\,\alpha_2\,p_{t,B}\,p_{T,A}+$$
$$r\,S\,w_4\,\alpha_2\,p_{t,B}\,p_{T,A}+r\,w_3\,\beta_1\,p_{t,B}\,p_{T,A}-r\,S\,w_3\,\beta_1\,p_{t,B}\,p_{T,A}-r\,w_4\,\beta_1\,p_{t,B}\,p_{T,A}+r\,S\,w_4\,\beta_1\,p_{t,B}\,p_{T,A}+$$
$$w_3\,p_{T,A}^2-S\,w_3\,p_{T,A}^2-w_3\,\alpha_2\,p_{T,A}^2+S\,w_3\,\alpha_2\,p_{T,A}^2+w_4\,\alpha_2\,p_{T,A}^2-S\,w_4\,\alpha_2\,p_{T,A}^2+S\,w_4\,p_{T,B}+$$
$$S\,w_3\,\beta_2\,p_{T,B}-S\,w_4\,\beta_2\,p_{T,B}+r\,w_3\,p_{t,A}\,p_{T,B}-r\,S\,w_3\,p_{t,A}\,p_{T,B}+w_4\,p_{t,A}\,p_{T,B}-r\,w_4\,p_{t,A}\,p_{T,B}-$$
$$S\,w_4\,p_{t,A}\,p_{T,B}+r\,S\,w_4\,p_{t,A}\,p_{T,B}-r\,w_3\,\alpha_1\,p_{t,A}\,p_{T,B}+r\,S\,w_3\,\alpha_1\,p_{t,A}\,p_{T,B}+r\,w_4\,\alpha_1\,p_{t,A}\,p_{T,B}-$$
$$r\,S\,w_4\,\alpha_1\,p_{t,A}\,p_{T,B}+w_3\,\beta_2\,p_{t,A}\,p_{T,B}-r\,w_3\,\beta_2\,p_{t,A}\,p_{T,B}-S\,w_3\,\beta_2\,p_{t,A}\,p_{T,B}+r\,S\,w_3\,\beta_2\,p_{t,A}\,p_{T,B}-$$
$$w_4\,\beta_2\,p_{t,A}\,p_{T,B}+r\,w_4\,\beta_2\,p_{t,A}\,p_{T,B}+S\,w_4\,\beta_2\,p_{t,A}\,p_{T,B}-r\,S\,w_4\,\beta_2\,p_{t,A}\,p_{T,B}+w_4\,p_{t,B}\,p_{T,B}-S\,w_4\,p_{t,B}\,p_{T,B}+$$
$$r\,w_3\,\beta_1\,p_{t,B}\,p_{T,B}-r\,S\,w_3\,\beta_1\,p_{t,B}\,p_{T,B}-r\,w_4\,\beta_1\,p_{t,B}\,p_{T,B}+r\,S\,w_4\,\beta_1\,p_{t,B}\,p_{T,B}+w_3\,\beta_2\,p_{t,B}\,p_{T,B}-$$
$$r\,w_3\,\beta_2\,p_{t,B}\,p_{T,B}-S\,w_3\,\beta_2\,p_{t,B}\,p_{T,B}+r\,S\,w_3\,\beta_2\,p_{t,B}\,p_{T,B}-w_4\,\beta_2\,p_{t,B}\,p_{T,B}+r\,w_4\,\beta_2\,p_{t,B}\,p_{T,B}+S\,w_4\,\beta_2\,p_{t,B}\,p_{T,B}-$$
$$r\,S\,w_4\,\beta_2\,p_{t,B}\,p_{T,B}+w_3\,p_{T,A}\,p_{T,B}-S\,w_3\,p_{T,A}\,p_{T,B}+w_4\,p_{T,A}\,p_{T,B}-S\,w_4\,p_{T,A}\,p_{T,B}-w_3\,\alpha_2\,p_{T,A}\,p_{T,B}+$$
$$S\,w_3\,\alpha_2\,p_{T,A}\,p_{T,B}+w_4\,\alpha_2\,p_{T,A}\,p_{T,B}-S\,w_4\,\alpha_2\,p_{T,A}\,p_{T,B}+w_3\,\beta_2\,p_{T,A}\,p_{T,B}-S\,w_3\,\beta_2\,p_{T,A}\,p_{T,B}-w_4\,\beta_2\,p_{T,A}\,p_{T,B}+$$
$$S\,w_4\,\beta_2\,p_{T,A}\,p_{T,B}+w_4\,p_{T,B}^2-S\,w_4\,p_{T,B}^2+w_3\,\beta_2\,p_{T,B}^2-S\,w_3\,\beta_2\,p_{T,B}^2-w_4\,\beta_2\,p_{T,B}^2+S\,w_4\,\beta_2\,p_{T,B}^2+$$
$$w_2\,\Big((-1+S)\,(-1+\beta_1)\,p_{t,B}^2+\alpha_1\,p_{t,A}\,\big(S-(-1+S)\,p_{t,A}-(-1+S)\,p_{t,B}+p_{T,A}-r\,p_{T,A}-S\,p_{T,A}+r\,S\,p_{T,A}+p_{T,B}-r\,p_{T,B}-S\,p_{T,B}+r\,S\,p_{T,B}\big)-$$
$$r\,(-1+S)\,p_{t,A}\,\big(\alpha_2\,p_{T,A}-(-1+\beta_2)\,p_{T,B}\big)+p_{t,B}\,\big(S+p_{t,A}-S\,p_{t,A}+p_{T,A}-r\,p_{T,A}-S\,p_{T,A}+r\,S\,p_{T,A}+r\,\alpha_2\,p_{T,A}-r\,S\,\alpha_2\,p_{T,A}+p_{T,B}-$$
$$S\,p_{T,B}-r\,\beta_2\,p_{T,B}+r\,S\,\beta_2\,p_{T,B}+\beta_1\,\big(-S+(-1+S)\,p_{t,A}-(-1+r)\,(-1+S)\,p_{T,A}-p_{T,B}+r\,p_{T,B}+S\,p_{T,B}-r\,S\,p_{T,B}\big)\big)\Big)+$$
$$w_1\,\Big((-1+S)\,(-1+\alpha_1)\,p_{t,A}^2+p_{t,A}\,\big(S+p_{t,B}-S\,p_{t,B}+\beta_1\,p_{t,B}-S\,\beta_1\,p_{t,B}+p_{T,A}-S\,p_{T,A}-r\,\alpha_2\,p_{T,A}+r\,S\,\alpha_2\,p_{T,A}+p_{T,B}-r\,p_{T,B}-$$
$$S\,p_{T,B}+r\,S\,p_{T,B}+r\,\beta_2\,p_{T,B}-r\,S\,\beta_2\,p_{T,B}+\alpha_1\,\big(-S+(-1+S)\,p_{t,B}-(-1+r)\,(-1+S)\,p_{T,A}-p_{T,B}+r\,p_{T,B}+S\,p_{T,B}-r\,S\,p_{T,B}\big)\big)+$$
$$p_{t,B}\,\big(\beta_1\,\big(S-(-1+S)\,p_{t,B}+(-1+r)\,(-1+S)\,p_{T,A}+p_{T,B}-r\,p_{T,B}-S\,p_{T,B}+r\,S\,p_{T,B}\big)+r\,(-1+S)\,\big((-1+\alpha_2)\,p_{T,A}-\beta_2\,p_{T,B}\big)\big)\Big)\Big)$$

Case A: The epiallele is deleterious and the transposon is neutral

A1 - Simplified Recursion Equations

Fitness assumptions:

Haploid Genotype	Fitness
tA	$w_1 = 1$
tB	$w_2 = 1-k$
TA	$w_3 = 1$
TB	$w_4 = 1-k$

After inputting the fitness assumptions above, reducing dimensions, and simplifying, the recursion equation are

$$
\begin{aligned}
p_{T,A}' = \Big(& z + p_{T,A} - y\, p_{T,A} - z\, p_{T,A} - \alpha_2\, p_{T,A} + r\, \alpha_2\, p_{T,A} - r\, S\, \alpha_2\, p_{T,A} + y\, \alpha_2\, p_{T,A} - r\, y\, \alpha_2\, p_{T,A} + r\, S\, y\, \alpha_2\, p_{T,A} - r\, z\, \alpha_2\, p_{T,A} + r\, S\, z\, \alpha_2\, p_{T,A} - \\
& r\, \alpha_2\, p_{T,A}^2 + r\, S\, \alpha_2\, p_{T,A}^2 + r\, y\, \alpha_2\, p_{T,A}^2 - r\, S\, y\, \alpha_2\, p_{T,A}^2 + r\, z\, \alpha_2\, p_{T,A}^2 - r\, S\, z\, \alpha_2\, p_{T,A}^2 + r\, p_{T,B} - r\, S\, p_{T,B} - r\, y\, p_{T,B} + \\
& r\, S\, y\, p_{T,B} - z\, p_{T,B} - r\, z\, p_{T,B} + r\, S\, z\, p_{T,B} + \beta_2\, p_{T,B} - r\, \beta_2\, p_{T,B} + r\, S\, \beta_2\, p_{T,B} - y\, \beta_2\, p_{T,B} + r\, y\, \beta_2\, p_{T,B} - r\, S\, y\, \beta_2\, p_{T,B} + \\
& r\, z\, \beta_2\, p_{T,B} - r\, S\, z\, \beta_2\, p_{T,B} - r\, p_{T,A}\, p_{T,B} + r\, S\, p_{T,A}\, p_{T,B} + r\, y\, p_{T,A}\, p_{T,B} - r\, S\, y\, p_{T,A}\, p_{T,B} + r\, z\, p_{T,A}\, p_{T,B} - r\, S\, z\, p_{T,A}\, p_{T,B} - \\
& r\, \alpha_2\, p_{T,A}\, p_{T,B} + r\, S\, \alpha_2\, p_{T,A}\, p_{T,B} + r\, y\, \alpha_2\, p_{T,A}\, p_{T,B} - r\, S\, y\, \alpha_2\, p_{T,A}\, p_{T,B} + r\, z\, \alpha_2\, p_{T,A}\, p_{T,B} - r\, S\, z\, \alpha_2\, p_{T,A}\, p_{T,B} + \\
& r\, \beta_2\, p_{T,A}\, p_{T,B} - r\, S\, \beta_2\, p_{T,A}\, p_{T,B} - r\, y\, \beta_2\, p_{T,A}\, p_{T,B} + r\, S\, y\, \beta_2\, p_{T,A}\, p_{T,B} - r\, z\, \beta_2\, p_{T,A}\, p_{T,B} + r\, S\, z\, \beta_2\, p_{T,A}\, p_{T,B} - r\, p_{T,B}^2 + \\
& r\, S\, p_{T,B}^2 + r\, y\, p_{T,B}^2 - r\, S\, y\, p_{T,B}^2 + r\, z\, p_{T,B}^2 - r\, S\, z\, p_{T,B}^2 + r\, \beta_2\, p_{T,B}^2 - r\, S\, \beta_2\, p_{T,B}^2 - r\, y\, \beta_2\, p_{T,B}^2 + r\, S\, y\, \beta_2\, p_{T,B}^2 - \\
& r\, z\, \beta_2\, p_{T,B}^2 + r\, S\, z\, \beta_2\, p_{T,B}^2 + (-1+\beta_1)\, p_{t,B} \big(z + r\,(-1+S)\,(-1+y+z)\, p_{T,A} + r\,(-1+S)\,(-1+y+z)\, p_{T,B}\big) + \\
& \alpha_1\,(-1+p_{t,B}+p_{T,A}+p_{T,B}) \big(z + r\,(-1+S)\,(-1+y+z)\, p_{T,A} + r\,(-1+S)\,(-1+y+z)\, p_{T,B}\big)\Big) \Big/ \\
& \big(1 + k\,(-1+\beta_1)\, p_{t,B} - k\, \alpha_2\, p_{T,A} - k\, p_{T,B} + k\, \beta_2\, p_{T,B} + k\, \alpha_1\,(-1+p_{t,B}+p_{T,A}+p_{T,B})\big)
\end{aligned}
$$

$$
\begin{aligned}
p_{t,B}' = & \\
-\Big(\Big(&(-1+k) \big((-1+\beta_1)\, p_{t,B} \big(-1 + z + r\,(-1+S)\,(-1+y+z)\, p_{T,A} + r\,(-1+S)\,(-1+y+z)\, p_{T,B}\big) + \alpha_1\,(-1+p_{t,B}+p_{T,A}+p_{T,B}) \big(-1 + \\
& z + r\,(-1+S)\,(-1+y+z)\, p_{T,A} + r\,(-1+S)\,(-1+y+z)\, p_{T,B}\big) - \big(-r + r\, S - y + r\, y - r\, S\, y + r\, z - \\
& r\, S\, z + r\,(-1+S)\,(-1+y+z)\, p_{T,A} + r\,(-1+S)\,(-1+y+z)\, p_{T,B}\big) \big(\alpha_2\, p_{T,A} - (-1+\beta_2)\, p_{T,B}\big)\big)\Big) \Big/ \\
& \big(1 + k\,(-1+\beta_1)\, p_{t,B} - k\, \alpha_2\, p_{T,A} - k\, p_{T,B} + k\, \beta_2\, p_{T,B} + k\, \alpha_1\,(-1+p_{t,B}+p_{T,A}+p_{T,B})\big)\Big)
\end{aligned}
$$

$$
\begin{aligned}
p_{T,B}' = \Big(&(-1+k) \big((-1+\beta_1)\, p_{t,B} \big(z + r\,(-1+S)\,(-1+y+z)\, p_{T,A} + r\,(-1+S)\,(-1+y+z)\, p_{T,B}\big) + \\
& \alpha_1\,(-1+p_{t,B}+p_{T,A}+p_{T,B}) \big(z + r\,(-1+S)\,(-1+y+z)\, p_{T,A} + r\,(-1+S)\,(-1+y+z)\, p_{T,B}\big) - \big(1 - r + r\, S - y + r\, y - \\
& r\, S\, y + r\, z - r\, S\, z + r\,(-1+S)\,(-1+y+z)\, p_{T,A} + r\,(-1+S)\,(-1+y+z)\, p_{T,B}\big) \big(\alpha_2\, p_{T,A} - (-1+\beta_2)\, p_{T,B}\big)\big)\Big) \Big/ \\
& \big(1 + k\,(-1+\beta_1)\, p_{t,B} - k\, \alpha_2\, p_{T,A} - k\, p_{T,B} + k\, \beta_2\, p_{T,B} + k\, \alpha_1\,(-1+p_{t,B}+p_{T,A}+p_{T,B})\big)
\end{aligned}
$$

A2 - Calculating Equilibria

$$
\begin{aligned}
&\{0 == \big(z + p_{T,A} - y\, p_{T,A} - z\, p_{T,A} - \alpha_2\, p_{T,A} + r\, \alpha_2\, p_{T,A} - r\, S\, \alpha_2\, p_{T,A} + y\, \alpha_2\, p_{T,A} - r\, y\, \alpha_2\, p_{T,A} + r\, S\, y\, \alpha_2\, p_{T,A} - \\
&\quad r\, z\, \alpha_2\, p_{T,A} + r\, S\, z\, \alpha_2\, p_{T,A} - r\, \alpha_2\, p_{T,A}^2 + r\, S\, \alpha_2\, p_{T,A}^2 + r\, y\, \alpha_2\, p_{T,A}^2 - r\, S\, y\, \alpha_2\, p_{T,A}^2 + r\, z\, \alpha_2\, p_{T,A}^2 - \\
&\quad r\, S\, z\, \alpha_2\, p_{T,A}^2 + r\, p_{T,B} - r\, S\, p_{T,B} - r\, y\, p_{T,B} + r\, S\, y\, p_{T,B} - z\, p_{T,B} - r\, z\, p_{T,B} + r\, S\, z\, p_{T,B} + \\
&\quad \beta_2\, p_{T,B} - r\, \beta_2\, p_{T,B} + r\, S\, \beta_2\, p_{T,B} - y\, \beta_2\, p_{T,B} + r\, y\, \beta_2\, p_{T,B} - r\, S\, y\, \beta_2\, p_{T,B} + r\, z\, \beta_2\, p_{T,B} - \\
&\quad r\, S\, z\, \beta_2\, p_{T,B} - r\, p_{T,A}\, p_{T,B} + r\, S\, p_{T,A}\, p_{T,B} + r\, y\, p_{T,A}\, p_{T,B} - r\, S\, y\, p_{T,A}\, p_{T,B} + r\, z\, p_{T,A}\, p_{T,B} - \\
&\quad r\, S\, z\, p_{T,A}\, p_{T,B} - r\, \alpha_2\, p_{T,A}\, p_{T,B} + r\, S\, \alpha_2\, p_{T,A}\, p_{T,B} + r\, y\, \alpha_2\, p_{T,A}\, p_{T,B} - r\, S\, y\, \alpha_2\, p_{T,A}\, p_{T,B} + \\
&\quad r\, z\, \alpha_2\, p_{T,A}\, p_{T,B} - r\, S\, z\, \alpha_2\, p_{T,A}\, p_{T,B} + r\, \beta_2\, p_{T,A}\, p_{T,B} - r\, S\, \beta_2\, p_{T,A}\, p_{T,B} - r\, y\, \beta_2\, p_{T,A}\, p_{T,B} + \\
&\quad r\, S\, y\, \beta_2\, p_{T,A}\, p_{T,B} - r\, z\, \beta_2\, p_{T,A}\, p_{T,B} + r\, S\, z\, \beta_2\, p_{T,A}\, p_{T,B} - r\, p_{T,B}^2 + r\, S\, p_{T,B}^2 + r\, y\, p_{T,B}^2 - r\, S\, y\, p_{T,B}^2 + \\
&\quad r\, z\, p_{T,B}^2 - r\, S\, z\, p_{T,B}^2 + r\, \beta_2\, p_{T,B}^2 - r\, S\, \beta_2\, p_{T,B}^2 - r\, y\, \beta_2\, p_{T,B}^2 + r\, S\, y\, \beta_2\, p_{T,B}^2 - r\, z\, \beta_2\, p_{T,B}^2 + \\
&\quad r\, S\, z\, \beta_2\, p_{T,B}^2 + (-1 + \beta_1)\, p_{t,B} \big(z + r\, (-1 + S)\, (-1 + y + z)\, p_{T,A} + r\, (-1 + S)\, (-1 + y + z)\, p_{T,B}\big) + \\
&\quad \alpha_1\, (-1 + p_{t,B} + p_{T,A} + p_{T,B}) \big(z + r\, (-1 + S)\, (-1 + y + z)\, p_{T,A} + r\, (-1 + S)\, (-1 + y + z)\, p_{T,B}\big)\big) \,/ \\
&\quad \big(1 + k\, (-1 + \beta_1)\, p_{t,B} - k\, \alpha_2\, p_{T,A} - k\, p_{T,B} + k\, \beta_2\, p_{T,B} + k\, \alpha_1\, (-1 + p_{t,B} + p_{T,A} + p_{T,B})\big) - p_{T,A}, \\
&0 == -\Big(\Big(\big((-1 + k) \big((-1 + \beta_1)\, p_{t,B} \big(-1 + z + r\, (-1 + S)\, (-1 + y + z)\, p_{T,A} + r\, (-1 + S)\, (-1 + y + z)\, p_{T,B}\big) + \\
&\quad \alpha_1\, (-1 + p_{t,B} + p_{T,A} + p_{T,B}) \big(-1 + z + r\, (-1 + S)\, (-1 + y + z)\, p_{T,A} + r\, (-1 + S)\, (-1 + y + z)\, p_{T,B}\big) - \\
&\quad \big(-r + r\, S - y + r\, y - r\, S\, y + r\, z - r\, S\, z + r\, (-1 + S)\, (-1 + y + z)\, p_{T,A} + r\, (-1 + S)\, (-1 + y + z)\, p_{T,B}\big) \\
&\quad \big(\alpha_2\, p_{T,A} - (-1 + \beta_2)\, p_{T,B}\big)\big)\Big) \,/ \\
&\quad \big(1 + k\, (-1 + \beta_1)\, p_{t,B} - k\, \alpha_2\, p_{T,A} - k\, p_{T,B} + k\, \beta_2\, p_{T,B} + k\, \alpha_1\, (-1 + p_{t,B} + p_{T,A} + p_{T,B})\big)\Big) - p_{t,B}, \\
&0 == \big((-1 + k) \big((-1 + \beta_1)\, p_{t,B} \big(z + r\, (-1 + S)\, (-1 + y + z)\, p_{T,A} + r\, (-1 + S)\, (-1 + y + z)\, p_{T,B}\big) + \\
&\quad \alpha_1\, (-1 + p_{t,B} + p_{T,A} + p_{T,B}) \big(z + r\, (-1 + S)\, (-1 + y + z)\, p_{T,A} + r\, (-1 + S)\, (-1 + y + z)\, p_{T,B}\big) - \\
&\quad \big(1 - r + r\, S - y + r\, y - r\, S\, y + r\, z - r\, S\, z + r\, (-1 + S)\, (-1 + y + z)\, p_{T,A} + r\, (-1 + S)\, (-1 + y + z)\, p_{T,B}\big) \\
&\quad \big(\alpha_2\, p_{T,A} - (-1 + \beta_2)\, p_{T,B}\big)\big)\big) \,/ \\
&\quad \big(1 + k\, (-1 + \beta_1)\, p_{t,B} - k\, \alpha_2\, p_{T,A} - k\, p_{T,B} + k\, \beta_2\, p_{T,B} + k\, \alpha_1\, (-1 + p_{t,B} + p_{T,A} + p_{T,B})\big) - p_{T,B}\} \;/. \\
&\{z \to z\, \zeta,\; y \to y\, \zeta,\; \beta_2 \to \beta_2\, \zeta,\; \alpha_1 \to \alpha_1\, \zeta,\; p_{T,A} \to (p_{T,A,0} + p_{T,A,1}\, \zeta), \\
&\quad p_{t,B} \to \\
&\quad (p_{t,B,0} + p_{t,B,1}\, \zeta),\; p_{T,B} \to \\
&\quad (p_{T,B,0} + p_{T,B,1}\, \zeta)\};
\end{aligned}
$$

In[◦]:= `Series[%, {ζ, 0, 1}];`

In[∘]:=

$$\text{Solve}\Big[\Big\{\Big(-p_{T,A,0}+\big(p_{T,A,0}-\alpha_2\,p_{T,A,0}+r\,\alpha_2\,p_{T,A,0}-r\,S\,\alpha_2\,p_{T,A,0}-r\,\alpha_2\,p_{T,A,0}^2+r\,S\,\alpha_2\,p_{T,A,0}^2+r\,p_{T,B,0}-r\,S\,p_{T,B,0}-r\,p_{T,A,0}\,p_{T,B,0}+r\,S\,p_{T,A,0}\,p_{T,B,0}-r\,\alpha_2\,p_{T,A,0}\,p_{T,B,0}+r\,S\,\alpha_2\,p_{T,A,0}\,p_{T,B,0}-r\,p_{T,B,0}^2+r\,S\,p_{T,B,0}^2+(-1+\beta_1)\,p_{t,B,0}\,\big(-r\,(-1+S)\,p_{T,A,0}-r\,(-1+S)\,p_{T,B,0}\big)\big)\Big/\big(1+k\,(-1+\beta_1)\,p_{t,B,0}-k\,\alpha_2\,p_{T,A,0}-k\,p_{T,B,0}\big)\Big)==0,$$

$$\Big(-p_{t,B,0}-\Big((-1+k)\,\big((-1+\beta_1)\,p_{t,B,0}\,\big(-1-r\,(-1+S)\,p_{T,A,0}-r\,(-1+S)\,p_{T,B,0}\big)-\big(\alpha_2\,p_{T,A,0}+p_{T,B,0}\big)\,\big(-r+r\,S-r\,(-1+S)\,p_{T,A,0}-r\,(-1+S)\,p_{T,B,0}\big)\big)\Big)\Big/\big(1+k\,(-1+\beta_1)\,p_{t,B,0}-k\,\alpha_2\,p_{T,A,0}-k\,p_{T,B,0}\big)\Big)==0,$$

$$\Big(-p_{T,B,0}+\Big((-1+k)\,\big((-1+\beta_1)\,p_{t,B,0}\,\big(-r\,(-1+S)\,p_{T,A,0}-r\,(-1+S)\,p_{T,B,0}\big)-\big(\alpha_2\,p_{T,A,0}+p_{T,B,0}\big)\,\big(1-r+r\,S-r\,(-1+S)\,p_{T,A,0}-r\,(-1+S)\,p_{T,B,0}\big)\big)\Big)\Big/\big(1+k\,(-1+\beta_1)\,p_{t,B,0}-k\,\alpha_2\,p_{T,A,0}-k\,p_{T,B,0}\big)\Big)==0\Big\},\ \{p_{T,A,0},\ p_{t,B,0},\ p_{T,B,0}\}\Big];$$

Only the zeroth order terms $\{p_{T,A,0}\to 0,\ p_{t,B,0}\to 0,\ p_{T,B,0}\to 0\}$, lead to a biologically valid and locally stable equilibrium.

Plugging these into the first order terms and solving for the first order terms

In[∘]:=

$$\Big\{\Big(-p_{T,A,1}-\Big(\big(p_{T,A,0}-\alpha_2\,p_{T,A,0}+r\,\alpha_2\,p_{T,A,0}-r\,S\,\alpha_2\,p_{T,A,0}-r\,\alpha_2\,p_{T,A,0}^2+r\,S\,\alpha_2\,p_{T,A,0}^2+r\,p_{T,B,0}-r\,S\,p_{T,B,0}-r\,p_{T,A,0}\,p_{T,B,0}+r\,S\,p_{T,A,0}\,p_{T,B,0}-r\,\alpha_2\,p_{T,A,0}\,p_{T,B,0}+r\,S\,\alpha_2\,p_{T,A,0}\,p_{T,B,0}-r\,p_{T,B,0}^2+r\,S\,p_{T,B,0}^2+(-1+\beta_1)\,p_{t,B,0}\,\big(-r\,(-1+S)\,p_{T,A,0}-r\,(-1+S)\,p_{T,B,0}\big)\big)\,\big(k\,(-1+\beta_1)\,p_{t,B,1}-k\,\alpha_2\,p_{T,A,1}+k\,\beta_2\,p_{T,B,0}+k\,\alpha_1\,(-1+p_{t,B,0}+p_{T,A,0}+p_{T,B,0})-k\,p_{T,B,1}\big)\Big)\Big/\big(1+k\,(-1+\beta_1)\,p_{t,B,0}-k\,\alpha_2\,p_{T,A,0}-k\,p_{T,B,0}\big)^2+$$

$$\Big(z-y\,p_{T,A,0}-z\,p_{T,A,0}+y\,\alpha_2\,p_{T,A,0}-r\,y\,\alpha_2\,p_{T,A,0}+r\,S\,y\,\alpha_2\,p_{T,A,0}-r\,z\,\alpha_2\,p_{T,A,0}+r\,S\,z\,\alpha_2\,p_{T,A,0}+r\,y\,\alpha_2\,p_{T,A,0}^2-r\,S\,y\,\alpha_2\,p_{T,A,0}^2+r\,z\,\alpha_2\,p_{T,A,0}^2-r\,S\,z\,\alpha_2\,p_{T,A,0}^2+p_{T,A,1}-\alpha_2\,p_{T,A,1}+r\,\alpha_2\,p_{T,A,1}-r\,S\,\alpha_2\,p_{T,A,1}-2\,r\,\alpha_2\,p_{T,A,0}\,p_{T,A,1}+2\,r\,S\,\alpha_2\,p_{T,A,0}\,p_{T,A,1}-r\,y\,p_{T,B,0}+r\,S\,y\,p_{T,B,0}-z\,p_{T,B,0}-r\,z\,p_{T,B,0}+r\,S\,z\,p_{T,B,0}+\beta_2\,p_{T,B,0}-r\,\beta_2\,p_{T,B,0}+r\,S\,\beta_2\,p_{T,B,0}+r\,y\,p_{T,A,0}\,p_{T,B,0}-r\,S\,y\,p_{T,A,0}\,p_{T,B,0}+r\,z\,p_{T,A,0}\,p_{T,B,0}-r\,S\,z\,p_{T,A,0}\,p_{T,B,0}+r\,y\,\alpha_2\,p_{T,A,0}\,p_{T,B,0}-r\,S\,y\,\alpha_2\,p_{T,A,0}\,p_{T,B,0}+r\,z\,\alpha_2\,p_{T,A,0}\,p_{T,B,0}-r\,S\,z\,\alpha_2\,p_{T,A,0}\,p_{T,B,0}+r\,\beta_2\,p_{T,A,0}\,p_{T,B,0}-r\,S\,\beta_2\,p_{T,A,0}\,p_{T,B,0}+r\,y\,p_{T,B,0}^2-r\,S\,y\,p_{T,B,0}^2+r\,z\,p_{T,B,0}^2-r\,S\,z\,p_{T,B,0}^2+r\,\beta_2\,p_{T,B,0}^2-r\,S\,\beta_2\,p_{T,B,0}^2+\alpha_1\,(-1+p_{t,B,0}+p_{T,A,0}+p_{T,B,0})\,\big(-r\,(-1+S)\,p_{T,A,0}-r\,(-1+S)\,p_{T,B,0}\big)+(-1+\beta_1)\,\Big(p_{t,B,1}\,\big(-r\,(-1+S)\,p_{T,A,0}-r\,(-1+S)\,p_{T,B,0}\big)+p_{t,B,0}\,\Big(z+r\,(-1+S)\,\big((y+z)\,p_{T,A,0}-p_{T,A,1}\big)+r\,(-1+S)\,\big((y+z)\,p_{T,B,0}-p_{T,B,1}\big)\Big)\Big)+r\,p_{T,B,1}-r\,S\,p_{T,B,1}-2\,r\,p_{T,B,0}\,p_{T,B,1}+2\,r\,S\,p_{T,B,0}\,p_{T,B,1}-r\,\big(p_{T,A,1}\,p_{T,B,0}+p_{T,A,0}\,p_{T,B,1}\big)+r\,S\,\big(p_{T,A,1}\,p_{T,B,0}+p_{T,A,0}\,p_{T,B,1}\big)-r\,\alpha_2\,\big(p_{T,A,1}\,p_{T,B,0}+p_{T,A,0}\,p_{T,B,1}\big)+r\,S\,\alpha_2\,\big(p_{T,A,1}\,p_{T,B,0}+p_{T,A,0}\,p_{T,B,1}\big)\Big)\Big/\big(1+k\,(-1+\beta_1)\,p_{t,B,0}-k\,\alpha_2\,p_{T,A,0}-k\,p_{T,B,0}\big)\Big)==0,$$

$$\Big(-p_{t,B,1}+(-1+k)\,\Big(\Big(\Big(\big((-1+\beta_1)\,p_{t,B,0}\,\big(-1-r\,(-1+S)\,p_{T,A,0}-r\,(-1+S)\,p_{T,B,0}\big)-\big(\alpha_2\,p_{T,A,0}+p_{T,B,0}\big)\,\big(-r+r\,S-r\,(-1+S)\,p_{T,A,0}-r\,(-1+S)\,p_{T,B,0}\big)\big)\,\big(k\,(-1+\beta_1)\,p_{t,B,1}-k\,\alpha_2\,p_{T,A,1}+k\,\beta_2\,p_{T,B,0}+k\,\alpha_1\,(-1+p_{t,B,0}+p_{T,A,0}+p_{T,B,0})-k\,p_{T,B,1}\big)\Big)\Big/\big(1+k\,(-1+\beta_1)\,p_{t,B,0}-k\,\alpha_2\,p_{T,A,0}-k\,p_{T,B,0}\big)^2-$$

$$\Big(\alpha_1\,(-1+p_{t,B,0}+p_{T,A,0}+p_{T,B,0})\,\big(-1-r\,(-1+S)\,p_{T,A,0}-r\,(-1+S)\,p_{T,B,0}\big)+(-1+\beta_1)\,\Big(p_{t,B,1}\,\big(-1-r\,(-1+S)\,p_{T,A,0}-r\,(-1+S)\,p_{T,B,0}\big)+p_{t,B,0}$$

$$(z + r (-1 + S) ((y + z) p_{T,A,0} - p_{T,A,1}) + r (-1 + S) ((y + z) p_{T,B,0} - p_{T,B,1}))) -$$
$$(\alpha_2 p_{T,A,0} + p_{T,B,0}) (-y + r y - r S y + r z - r S z + r (-1 + S) ((y + z) p_{T,A,0} - p_{T,A,1}) +$$
$$r (-1 + S) ((y + z) p_{T,B,0} - p_{T,B,1})) - (-r + r S - r (-1 + S) p_{T,A,0} - r (-1 + S) p_{T,B,0})$$
$$(\alpha_2 p_{T,A,1} - \beta_2 p_{T,B,0} + p_{T,B,1})) / (1 + k (-1 + \beta_1) p_{t,B,0} - k \alpha_2 p_{T,A,0} - k p_{T,B,0}))) == 0,$$
$$(-p_{T,B,1} + (-1 + k) (-((((-1 + \beta_1) p_{t,B,0} (-r (-1 + S) p_{T,A,0} - r (-1 + S) p_{T,B,0}) - (\alpha_2 p_{T,A,0} + p_{T,B,0})$$
$$(1 - r + r S - r (-1 + S) p_{T,A,0} - r (-1 + S) p_{T,B,0}))$$
$$(k (-1 + \beta_1) p_{t,B,1} - k \alpha_2 p_{T,A,1} + k \beta_2 p_{T,B,0} + k \alpha_1 (-1 + p_{t,B,0} + p_{T,A,0} + p_{T,B,0}) -$$
$$k p_{T,B,1})) / (1 + k (-1 + \beta_1) p_{t,B,0} - k \alpha_2 p_{T,A,0} - k p_{T,B,0})^2) +$$
$$(\alpha_1 (-1 + p_{t,B,0} + p_{T,A,0} + p_{T,B,0}) (-r (-1 + S) p_{T,A,0} - r (-1 + S) p_{T,B,0}) + (-1 + \beta_1)$$
$$(p_{t,B,1} (-r (-1 + S) p_{T,A,0} - r (-1 + S) p_{T,B,0}) + p_{t,B,0} (z + r (-1 + S) ((y + z) p_{T,A,0} - p_{T,A,1}) +$$
$$r (-1 + S) ((y + z) p_{T,B,0} - p_{T,B,1}))) - (\alpha_2 p_{T,A,0} + p_{T,B,0}) (-y + r y - r S y + r z - r$$
$$S z + r (-1 + S) ((y + z) p_{T,A,0} - p_{T,A,1}) + r (-1 + S) ((y + z) p_{T,B,0} - p_{T,B,1})) -$$
$$(1 - r + r S - r (-1 + S) p_{T,A,0} - r (-1 + S) p_{T,B,0}) (\alpha_2 p_{T,A,1} - \beta_2 p_{T,B,0} + p_{T,B,1})) /$$
$$(1 + k (-1 + \beta_1) p_{t,B,0} - k \alpha_2 p_{T,A,0} - k p_{T,B,0}))) == 0\} \;\text{/.}\; \{p_{T,A,0} \to 0,\ p_{t,B,0} \to 0,\ p_{T,B,0} \to 0\};$$

In[ ]:= Solve[%, $\{p_{T,A,1}, p_{t,B,1}, p_{T,B,1}\}$]

Out[ ]= $$\left\{\left\{p_{T,A,1} \to \frac{(k + r - k r - r S + k r S) z}{k (1 - r + r S) \alpha_2},\ p_{t,B,1} \to \frac{(-1 + k) (r z - r S z + k \alpha_1 - k r \alpha_1 + k r S \alpha_1)}{k (1 - r + r S) (-k - \beta_1 + k \beta_1)},\ p_{T,B,1} \to -\frac{(-1 + k) z}{k}\right\}\right\}$$

Combining these first order terms with the zero order terms and simplifying results in eq. 1 from the main text.

A3 - Calculating Eigenvalues

```
ResourceFunction["JacobianMatrix"][
  {(z + p_{T,A} - y p_{T,A} - z p_{T,A} - α2 p_{T,A} + r α2 p_{T,A} - r S α2 p_{T,A} + y α2 p_{T,A} - r y α2 p_{T,A} + r S y α2 p_{T,A} -
       r z α2 p_{T,A} + r S z α2 p_{T,A} - r α2 p_{T,A}^2 + r S α2 p_{T,A}^2 + r y α2 p_{T,A}^2 - r S y α2 p_{T,A}^2 + r z α2 p_{T,A}^2 -
       r S z α2 p_{T,A}^2 + r p_{T,B} - r S p_{T,B} - r y p_{T,B} + r S y p_{T,B} - z p_{T,B} - r z p_{T,B} + r S z p_{T,B} +
       β2 p_{T,B} - r β2 p_{T,B} + r S β2 p_{T,B} - y β2 p_{T,B} + r y β2 p_{T,B} - r S y β2 p_{T,B} + r z β2 p_{T,B} -
       r S z β2 p_{T,B} - r p_{T,A} p_{T,B} + r S p_{T,A} p_{T,B} + r y p_{T,A} p_{T,B} - r S y p_{T,A} p_{T,B} + r z p_{T,A} p_{T,B} -
       r S z p_{T,A} p_{T,B} - r α2 p_{T,A} p_{T,B} + r S α2 p_{T,A} p_{T,B} + r y α2 p_{T,A} p_{T,B} - r S y α2 p_{T,A} p_{T,B} +
       r z α2 p_{T,A} p_{T,B} - r S z α2 p_{T,A} p_{T,B} + r β2 p_{T,A} p_{T,B} - r S β2 p_{T,A} p_{T,B} - r y β2 p_{T,A} p_{T,B} +
       r S y β2 p_{T,A} p_{T,B} - r z β2 p_{T,A} p_{T,B} + r S z β2 p_{T,A} p_{T,B} - r p_{T,B}^2 + r S p_{T,B}^2 + r y p_{T,B}^2 - r S y p_{T,B}^2 +
       r z p_{T,B}^2 - r S z p_{T,B}^2 + r β2 p_{T,B}^2 - r S β2 p_{T,B}^2 - r y β2 p_{T,B}^2 + r S y β2 p_{T,B}^2 - r z β2 p_{T,B}^2 +
       r S z β2 p_{T,B}^2 + (-1 + β1) p_{t,B} (z + r (-1 + S) (-1 + y + z) p_{T,A} + r (-1 + S) (-1 + y + z) p_{T,B}) +
       α1 (-1 + p_{t,B} + p_{T,A} + p_{T,B}) (z + r (-1 + S) (-1 + y + z) p_{T,A} + r (-1 + S) (-1 + y + z) p_{T,B})) /
     (1 + k (-1 + β1) p_{t,B} - k α2 p_{T,A} - k p_{T,B} + k β2 p_{T,B} + k α1 (-1 + p_{t,B} + p_{T,A} + p_{T,B})),
   -(((-1 + k) ((-1 + β1) p_{t,B} (-1 + z + r (-1 + S) (-1 + y + z) p_{T,A} + r (-1 + S) (-1 + y + z) p_{T,B}) +
           α1 (-1 + p_{t,B} + p_{T,A} + p_{T,B}) (-1 + z + r (-1 + S) (-1 + y + z) p_{T,A} + r (-1 + S) (-1 + y + z) p_{T,B}) -
           (-r + r S - y + r y - r S y + r z - r S z + r (-1 + S) (-1 + y + z) p_{T,A} + r (-1 + S) (-1 + y + z) p_{T,B})
            (α2 p_{T,A} - (-1 + β2) p_{T,B}))) /
      (1 + k (-1 + β1) p_{t,B} - k α2 p_{T,A} - k p_{T,B} + k β2 p_{T,B} + k α1 (-1 + p_{t,B} + p_{T,A} + p_{T,B}))),
   ((-1 + k) ((-1 + β1) p_{t,B} (z + r (-1 + S) (-1 + y + z) p_{T,A} + r (-1 + S) (-1 + y + z) p_{T,B}) + α1 (-1 + p_{t,B} + p_{T,A} + p_{T,B})
          (z + r (-1 + S) (-1 + y + z) p_{T,A} + r (-1 + S) (-1 + y + z) p_{T,B}) - (1 - r + r S - y + r y - r S y + r z -
             r S z + r (-1 + S) (-1 + y + z) p_{T,A} + r (-1 + S) (-1 + y + z) p_{T,B}) (α2 p_{T,A} - (-1 + β2) p_{T,B}))) /
    (1 + k (-1 + β1) p_{t,B} - k α2 p_{T,A} - k p_{T,B} + k β2 p_{T,B} + k α1 (-1 + p_{t,B} + p_{T,A} + p_{T,B}))},
  {p_{T,A}, p_{t,B}, p_{T,B}}] // MatrixForm;

% /. {z → z ζ, y → y ζ, β2 → β2 ζ, α1 → α1 ζ, p_{T,A} → (p_{T,A,0} + p_{T,A,1} ζ),
    p_{t,B} → (p_{t,B,0} + p_{t,B,1} ζ), p_{T,B} → (p_{T,B,0} + p_{T,B,1} ζ)} // MatrixForm;

Det[% - ( λ0 + λ1 ζ    0            0
          0            λ0 + λ1 ζ    0
          0            0            λ0 + λ1 ζ )];

Series[% == 0, {ζ, 0, 1}];

% /. {p_{T,A,0} → 0, p_{t,B,0} → 0, p_{T,B,0} → 0, p_{T,A,1} → ((k + r - k r - r S + k r S) z) / (k (1 - r + r S) α2),
    p_{t,B,1} → ((-1 + k) (r z - r S z + k α1 - k r α1 + k r S α1)) / (k (1 - r + r S) (-k - β1 + k β1)), p_{T,B,1} → -((-1 + k) z) / k};
```

Solving for zeroth order eigenvalues:

```
Solve[(-((-1 + k) (-1 + r - r S) (r - r S) α2 ((-1 + k) (-1 + β1) - λ0)) +
    ((-1 + k) (-1 + r - r S) - λ0) (1 - α2 + r α2 - r S α2 - λ0) ((-1 + k) (-1 + β1) - λ0)) == 0, λ0];

Simplify[%]
```

Out[∘]=
$$\left\{\left\{\lambda_0 \to \frac{1}{2}\left(2 - k - r + k\,r + r\,S - k\,r\,S - \alpha_2 + r\,\alpha_2 - r\,S\,\alpha_2 - \sqrt{\left(-4\,(-1+k)\left(1 + r\,(-1+S)\right)(-1+\alpha_2) + \left(-2 + k + r - k\,r - r\,S + k\,r\,S + \left(1 + r\,(-1+S)\right)\alpha_2\right)^2\right)}\right)\right\},\right.$$
$$\left\{\lambda_0 \to \frac{1}{2}\left(2 - k - r + k\,r + r\,S - k\,r\,S - \alpha_2 + r\,\alpha_2 - r\,S\,\alpha_2 + \sqrt{\left(-4\,(-1+k)\left(1 + r\,(-1+S)\right)(-1+\alpha_2) + \left(-2 + k + r - k\,r - r\,S + k\,r\,S + \left(1 + r\,(-1+S)\right)\alpha_2\right)^2\right)}\right)\right\},\ \left.\left\{\lambda_0 \to (-1+k)(-1+\beta_1)\right\}\right\}$$

Combining the zeroth and first order terms for each eigenvalue give the full first order approximation. For numerical parameter values evaluated in the main text and File S1 for case A, the eigenvalues suggested local stability of the presented equilibria such that $|\lambda_i| < 1$.

Calculations of the first order terms of the eigenvalues

Plugging the first zeroth order eigenvalue term and solving for the first order eigenvalue term:

In[∘]:=
$$\Bigg(-\Bigg(\Bigg((-1+k)\,k\,(1-r+r\,S)\left(-\frac{(-1+k)\,z}{k} + \frac{(k+r-k\,r-r\,S+k\,r\,S)\,z}{k\,(1-r+r\,S)}\right)(-1+\beta_1) +$$
$$(-1+k)\left(z + \frac{(-1+k)\,r\,(-1+S)\,z}{k} - \frac{r\,(-1+S)\,(k+r-k\,r-r\,S+k\,r\,S)\,z}{k\,(1-r+r\,S)\,\alpha_2}\right)(-1+\beta_1)\Bigg)$$
$$\left((-1+k)\,(r-r\,S)^2\,\alpha_2 - (-1+k)\,(r-r\,S)\,(1-\alpha_2+r\,\alpha_2-r\,S\,\alpha_2-\lambda_0)\right)\Bigg) -$$
$$(r-r\,S)\Bigg(-\Bigg((-1+k)\,k\,(1-r+r\,S)\left(-\frac{(-1+k)\,z}{k} + \frac{(k+r-k\,r-r\,S+k\,r\,S)\,z}{k\,(1-r+r\,S)}\right)\alpha_2\Bigg) +$$
$$(-1+k)\Bigg(r\,(-1+S)\left(-\frac{(-1+k)\,z}{k} + \frac{(k+r-k\,r-r\,S+k\,r\,S)\,z}{k\,(1-r+r\,S)}\right) + r\,(-1+S)\,\alpha_1 +$$
$$\left(y - r\,y + r\,S\,y - r\,z - \frac{(-1+k)\,r\,(-1+S)\,z}{k} + r\,S\,z + \frac{r\,(-1+S)\,(k+r-k\,r-r\,S+k\,r\,S)\,z}{k\,(1-r+r\,S)\,\alpha_2}\right)\alpha_2 -$$
$$\frac{(-1+k)\,r\,(-1+S)\,(r\,z-r\,S\,z+k\,\alpha_1-k\,r\,\alpha_1+k\,r\,S\,\alpha_1)\,(-1+\beta_1)}{k\,(1-r+r\,S)\,(-k-\beta_1+k\,\beta_1)} -$$
$$(-1+r-r\,S)\,\alpha_2\Bigg((-1+k)\,z - \frac{(k+r-k\,r-r\,S+k\,r\,S)\,z}{1-r+r\,S} - k\,\alpha_1 +$$
$$\frac{(-1+k)\,(r\,z-r\,S\,z+k\,\alpha_1-k\,r\,\alpha_1+k\,r\,S\,\alpha_1)\,(-1+\beta_1)}{(1-r+r\,S)\,(-k-\beta_1+k\,\beta_1)}\Bigg)\Bigg)\Bigg)\left((-1+k)\,(-1+\beta_1)-\lambda_0\right) +$$

$$(-1+k)\,(-1+r-r\,S)\,\alpha_2\,\Bigg(-\Bigg((-1+k)\,(r-r\,S)\,\Bigg(-k\,\Bigg(z-\frac{(-1+k)\,r\,z}{k}+\frac{(-1+k)\,r\,S\,z}{k}-\frac{(k+r-k\,r-r\,S+k\,r\,S)\,z}{k\,(1-r+r\,S)}+$$

$$\frac{r\,(k+r-k\,r-r\,S+k\,r\,S)\,z}{k\,(1-r+r\,S)}-\frac{r\,S\,(k+r-k\,r-r\,S+k\,r\,S)\,z}{k\,(1-r+r\,S)}+\frac{(k+r-k\,r-r\,S+k\,r\,S)\,z}{k\,(1-r+r\,S)\,\alpha_2}\Bigg)$$

$$(-1+\beta_1)+\left(z+\frac{(-1+k)\,r\,(-1+S)\,z}{k}-\frac{r\,(-1+S)\,(k+r-k\,r-r\,S+k\,r\,S)\,z}{k\,(1-r+r\,S)\,\alpha_2}\right)(-1+\beta_1)\Bigg)\Bigg)-$$

$$\Bigg(-r\,y+r\,S\,y-z-r\,z+\frac{2\,(-1+k)\,r\,z}{k}+r\,S\,z-\frac{2\,(-1+k)\,r\,S\,z}{k}-\frac{r\,(k+r-k\,r-r\,S+k\,r\,S)\,z}{k\,(1-r+r\,S)}+$$

$$\frac{r\,S\,(k+r-k\,r-r\,S+k\,r\,S)\,z}{k\,(1-r+r\,S)}+r\,(-1+S)\,\alpha_1+$$

$$k\,\Bigg(z-\frac{(-1+k)\,r\,z}{k}+\frac{(-1+k)\,r\,S\,z}{k}-\frac{(k+r-k\,r-r\,S+k\,r\,S)\,z}{k\,(1-r+r\,S)}+\frac{r\,(k+r-k\,r-r\,S+k\,r\,S)\,z}{k\,(1-r+r\,S)}-$$

$$\frac{r\,S\,(k+r-k\,r-r\,S+k\,r\,S)\,z}{k\,(1-r+r\,S)}+\frac{(k+r-k\,r-r\,S+k\,r\,S)\,z}{k\,(1-r+r\,S)\,\alpha_2}\Bigg)-\frac{r\,(k+r-k\,r-r\,S+k\,r\,S)\,z}{k\,(1-r+r\,S)\,\alpha_2}+$$

$$\frac{r\,S\,(k+r-k\,r-r\,S+k\,r\,S)\,z}{k\,(1-r+r\,S)\,\alpha_2}-\frac{(-1+k)\,r\,(-1+S)\,(r\,z-r\,S\,z+k\,\alpha_1-k\,r\,\alpha_1+k\,r\,S\,\alpha_1)\,(-1+\beta_1)}{k\,(1-r+r\,S)\,(-k-\beta_1+k\,\beta_1)}-$$

$$(r-r\,S)\,\Bigg((-1+k)\,z-\frac{(k+r-k\,r-r\,S+k\,r\,S)\,z}{1-r+r\,S}-k\,\alpha_1+$$

$$\frac{(-1+k)\,(r\,z-r\,S\,z+k\,\alpha_1-k\,r\,\alpha_1+k\,r\,S\,\alpha_1)\,(-1+\beta_1)}{(1-r+r\,S)\,(-k-\beta_1+k\,\beta_1)}\Bigg)+\beta_2-r\,\beta_2+r\,S\,\beta_2\Bigg)\,((-1+k)\,(-1+\beta_1)-\lambda_0)-$$

$$(r-r\,S)\,\Bigg((-1+k)\,k\,(-1+\beta_1)\,\Bigg(-\Bigg((-r+r\,S)\,\Bigg(-\frac{(-1+k)\,z}{k}+\frac{(k+r-k\,r-r\,S+k\,r\,S)\,z}{k\,(1-r+r\,S)}\Bigg)\Bigg)+$$

$$\alpha_1-\frac{(-1+k)\,(r\,z-r\,S\,z+k\,\alpha_1-k\,r\,\alpha_1+k\,r\,S\,\alpha_1)\,(-1+\beta_1)}{k\,(1-r+r\,S)\,(-k-\beta_1+k\,\beta_1)}\Bigg)+$$

$$(-1+k)\,\Bigg(\alpha_1-\left(z+\frac{(-1+k)\,r\,(-1+S)\,z}{k}-\frac{r\,(-1+S)\,(k+r-k\,r-r\,S+k\,r\,S)\,z}{k\,(1-r+r\,S)\,\alpha_2}\right)(-1+\beta_1)-$$

$$(-1+\beta_1)\,\Bigg((-1+k)\,z-\frac{(k+r-k\,r-r\,S+k\,r\,S)\,z}{1-r+r\,S}-k\,\alpha_1+$$

$$\frac{(-1+k)\,(r\,z-r\,S\,z+k\,\alpha_1-k\,r\,\alpha_1+k\,r\,S\,\alpha_1)\,(-1+\beta_1)}{(1-r+r\,S)\,(-k-\beta_1+k\,\beta_1)}\Bigg)\Bigg)-\lambda_1\Bigg)\Bigg)+((-1+k)\,(-1+r-r\,S)-\lambda_0)$$

$$\Bigg((-1+k)\,(r-r\,S)\,\alpha_2\,\Bigg(-k\,\Bigg(z-\frac{(-1+k)\,r\,z}{k}+\frac{(-1+k)\,r\,S\,z}{k}-\frac{(k+r-k\,r-r\,S+k\,r\,S)\,z}{k\,(1-r+r\,S)}+$$

$$\frac{r\,(k+r-k\,r-r\,S+k\,r\,S)\,z}{k\,(1-r+r\,S)}-\frac{r\,S\,(k+r-k\,r-r\,S+k\,r\,S)\,z}{k\,(1-r+r\,S)}+\frac{(k+r-k\,r-r\,S+k\,r\,S)\,z}{k\,(1-r+r\,S)\,\alpha_2}\Bigg)$$

$$(-1+\beta_1)+\Bigg(z+\frac{(-1+k)\,r\,(-1+S)\,z}{k}-\frac{r\,(-1+S)\,(k+r-k\,r-r\,S+k\,r\,S)\,z}{k\,(1-r+r\,S)\,\alpha_2}\Bigg)\,(-1+\beta_1)\Bigg)+$$

$$((-1+k)\,(-1+\beta_1)-\lambda_0)\,\Bigg(-y-z+\frac{(-1+k)\,r\,z}{k}-\frac{(-1+k)\,r\,S\,z}{k}-\frac{2\,r\,(k+r-k\,r-r\,S+k\,r\,S)\,z}{k\,(1-r+r\,S)}+$$

$$\frac{2\,r\,S\,(k+r-k\,r-r\,S+k\,r\,S)\,z}{k\,(1-r+r\,S)}+r\,(-1+S)\,\alpha_1+y\,\alpha_2-r\,y\,\alpha_2+r\,S\,y\,\alpha_2-r\,z\,\alpha_2+\frac{(-1+k)\,r\,z\,\alpha_2}{k}+$$

$$r\,S\,z\,\alpha_2-\frac{(-1+k)\,r\,S\,z\,\alpha_2}{k}+k\,\Bigg(z-\frac{(-1+k)\,r\,z}{k}+\frac{(-1+k)\,r\,S\,z}{k}-\frac{(k+r-k\,r-r\,S+k\,r\,S)\,z}{k\,(1-r+r\,S)}+$$

$$\frac{r\,(k+r-k\,r-r\,S+k\,r\,S)\,z}{k\,(1-r+r\,S)}-\frac{r\,S\,(k+r-k\,r-r\,S+k\,r\,S)\,z}{k\,(1-r+r\,S)}+\frac{(k+r-k\,r-r\,S+k\,r\,S)\,z}{k\,(1-r+r\,S)\,\alpha_2}\Bigg)\,\alpha_2-$$

$$\frac{(-1+k)\,r\,(-1+S)\,(r\,z-r\,S\,z+k\,\alpha_1-k\,r\,\alpha_1+k\,r\,S\,\alpha_1)\,(-1+\beta_1)}{k\,(1-r+r\,S)\,(-k-\beta_1+k\,\beta_1)}-(1-\alpha_2+r\,\alpha_2-r\,S\,\alpha_2)\,\Bigg((-1+k)\,z-$$

$$\frac{(k+r-k\,r-r\,S+k\,r\,S)\,z}{1-r+r\,S}-k\,\alpha_1+\frac{(-1+k)\,(r\,z-r\,S\,z+k\,\alpha_1-k\,r\,\alpha_1+k\,r\,S\,\alpha_1)\,(-1+\beta_1)}{(1-r+r\,S)\,(-k-\beta_1+k\,\beta_1)}\Bigg)-\lambda_1\Bigg)+$$

$$(1-\alpha_2+r\,\alpha_2-r\,S\,\alpha_2-\lambda_0)\,\Bigg((-1+k)\,k\,(-1+\beta_1)\,\Bigg(-\Bigg((-r+r\,S)\,\Bigg(-\frac{(-1+k)\,z}{k}+\frac{(k+r-k\,r-r\,S+k\,r\,S)\,z}{k\,(1-r+r\,S)}\Bigg)\Bigg)+$$

$$\alpha_1-\frac{(-1+k)\,(r\,z-r\,S\,z+k\,\alpha_1-k\,r\,\alpha_1+k\,r\,S\,\alpha_1)\,(-1+\beta_1)}{k\,(1-r+r\,S)\,(-k-\beta_1+k\,\beta_1)}\Bigg)+$$

$$(-1+k)\,\Bigg(\alpha_1-\Bigg(z+\frac{(-1+k)\,r\,(-1+S)\,z}{k}-\frac{r\,(-1+S)\,(k+r-k\,r-r\,S+k\,r\,S)\,z}{k\,(1-r+r\,S)\,\alpha_2}\Bigg)\,(-1+\beta_1)-$$

$$(-1+\beta_1)\,\Bigg((-1+k)\,z-\frac{(k+r-k\,r-r\,S+k\,r\,S)\,z}{1-r+r\,S}-k\,\alpha_1+$$

$$\frac{(-1+k)\,(r\,z-r\,S\,z+k\,\alpha_1-k\,r\,\alpha_1+k\,r\,S\,\alpha_1)\,(-1+\beta_1)}{(1-r+r\,S)\,(-k-\beta_1+k\,\beta_1)}\Bigg)\Bigg)-\lambda_1\Bigg)\Bigg)+(1-\alpha_2+r\,\alpha_2-r\,S\,\alpha_2-\lambda_0)$$

$$((-1+k)\,(-1+\beta_1)-\lambda_0)\,\Bigg(-\Bigg((-1+k)\,k\,(1-r+r\,S)\,\Bigg(-\frac{(-1+k)\,z}{k}+\frac{(k+r-k\,r-r\,S+k\,r\,S)\,z}{k\,(1-r+r\,S)}\Bigg)\Bigg)+$$

$$(-1+k)\left(y-r\,y+r\,S\,y-r\,z-\frac{(-1+k)\,r\,(-1+S)\,z}{k}+r\,S\,z+r\,(-1+S)\right.$$

$$\left(-\frac{(-1+k)\,z}{k}+\frac{\left(k+r-k\,r-r\,S+k\,r\,S\right)z}{k\left(1-r+r\,S\right)}\right)+r\,(-1+S)\,\alpha_1+\frac{r\,(-1+S)\left(k+r-k\,r-r\,S+k\,r\,S\right)z}{k\left(1-r+r\,S\right)\alpha_2}-$$

$$\frac{(-1+k)\,r\,(-1+S)\left(r\,z-r\,S\,z+k\,\alpha_1-k\,r\,\alpha_1+k\,r\,S\,\alpha_1\right)(-1+\beta_1)}{k\left(1-r+r\,S\right)\left(-k-\beta_1+k\,\beta_1\right)}-$$

$$\left(-1+r-r\,S\right)\left((-1+k)\,z-\frac{\left(k+r-k\,r-r\,S+k\,r\,S\right)z}{1-r+r\,S}-k\,\alpha_1+\right.$$

$$\left.\left.\left.\frac{(-1+k)\left(r\,z-r\,S\,z+k\,\alpha_1-k\,r\,\alpha_1+k\,r\,S\,\alpha_1\right)(-1+\beta_1)}{\left(1-r+r\,S\right)\left(-k-\beta_1+k\,\beta_1\right)}\right)+\left(1-r+r\,S\right)\beta_2\right)-\lambda_1\right)\right)\ /.$$

$$\lambda_0\to\frac{1}{2}\left(2-k-r+k\,r+r\,S-k\,r\,S-\alpha_2+r\,\alpha_2-r\,S\,\alpha_2-\sqrt{\left(-4\,(-1+k)\left(1+r\,(-1+S)\right)(-1+\alpha_2)+\right.}\right.$$

$$\left.\left.\left(-2+k+r-k\,r-r\,S+k\,r\,S+\left(1+r\,(-1+S)\right)\alpha_2\right)^2\right)\right);$$

```
Solve[% == 0, λ1];
```

```
Simplify[%];
```

Plugging in the second zeroth order eigenvalue term and solving for the first order eigenvalue term:

$$\left(-\left(\left((-1+k)\,k\left(1-r+r\,S\right)\left(-\frac{(-1+k)\,z}{k}+\frac{\left(k+r-k\,r-r\,S+k\,r\,S\right)z}{k\left(1-r+r\,S\right)}\right)(-1+\beta_1)+\right.\right.\right.$$

$$\left.(-1+k)\left(z+\frac{(-1+k)\,r\,(-1+S)\,z}{k}-\frac{r\,(-1+S)\left(k+r-k\,r-r\,S+k\,r\,S\right)z}{k\left(1-r+r\,S\right)\alpha_2}\right)(-1+\beta_1)\right)$$

$$\left.\left((-1+k)\left(r-r\,S\right)^2\alpha_2-(-1+k)\left(r-r\,S\right)\left(1-\alpha_2+r\,\alpha_2-r\,S\,\alpha_2-\lambda_0\right)\right)\right)-$$

$$\left(r-r\,S\right)\left(-\left((-1+k)\,k\left(1-r+r\,S\right)\left(-\frac{(-1+k)\,z}{k}+\frac{\left(k+r-k\,r-r\,S+k\,r\,S\right)z}{k\left(1-r+r\,S\right)}\right)\alpha_2\right)+\right.$$

$$(-1+k)\left(r\,(-1+S)\left(-\frac{(-1+k)\,z}{k}+\frac{\left(k+r-k\,r-r\,S+k\,r\,S\right)z}{k\left(1-r+r\,S\right)}\right)+r\,(-1+S)\,\alpha_1+\right.$$

$$\left(y-r\,y+r\,S\,y-r\,z-\frac{(-1+k)\,r\,(-1+S)\,z}{k}+r\,S\,z+\frac{r\,(-1+S)\left(k+r-k\,r-r\,S+k\,r\,S\right)z}{k\left(1-r+r\,S\right)\alpha_2}\right)\alpha_2-$$

$$\frac{(-1+k)\,r\,(-1+S)\left(r\,z-r\,S\,z+k\,\alpha_1-k\,r\,\alpha_1+k\,r\,S\,\alpha_1\right)(-1+\beta_1)}{k\left(1-r+r\,S\right)\left(-k-\beta_1+k\,\beta_1\right)}-$$

$$(-1 + r - r\,S)\,\alpha_2 \Bigg((-1 + k)\,z - \frac{(k + r - k\,r - r\,S + k\,r\,S)\,z}{1 - r + r\,S} - k\,\alpha_1 +$$

$$\frac{(-1 + k)\,(r\,z - r\,S\,z + k\,\alpha_1 - k\,r\,\alpha_1 + k\,r\,S\,\alpha_1)\,(-1 + \beta_1)}{(1 - r + r\,S)\,(-k - \beta_1 + k\,\beta_1)}\Bigg)\Bigg)\Bigg)\,((-1 + k)\,(-1 + \beta_1) - \lambda_0) +$$

$$(-1 + k)\,(-1 + r - r\,S)\,\alpha_2 \Bigg(-\Bigg((-1 + k)\,(r - r\,S)\Bigg(-k\Bigg(z - \frac{(-1 + k)\,r\,z}{k} + \frac{(-1 + k)\,r\,S\,z}{k} - \frac{(k + r - k\,r - r\,S + k\,r\,S)\,z}{k\,(1 - r + r\,S)} +$$

$$\frac{r\,(k + r - k\,r - r\,S + k\,r\,S)\,z}{k\,(1 - r + r\,S)} - \frac{r\,S\,(k + r - k\,r - r\,S + k\,r\,S)\,z}{k\,(1 - r + r\,S)} + \frac{(k + r - k\,r - r\,S + k\,r\,S)\,z}{k\,(1 - r + r\,S)\,\alpha_2}\Bigg)$$

$$(-1 + \beta_1) + \Bigg(z + \frac{(-1 + k)\,r\,(-1 + S)\,z}{k} - \frac{r\,(-1 + S)\,(k + r - k\,r - r\,S + k\,r\,S)\,z}{k\,(1 - r + r\,S)\,\alpha_2}\Bigg)\,(-1 + \beta_1)\Bigg)\Bigg) -$$

$$\Bigg(-r\,y + r\,S\,y - z - r\,z + \frac{2\,(-1 + k)\,r\,z}{k} + r\,S\,z - \frac{2\,(-1 + k)\,r\,S\,z}{k} - \frac{r\,(k + r - k\,r - r\,S + k\,r\,S)\,z}{k\,(1 - r + r\,S)} +$$

$$\frac{r\,S\,(k + r - k\,r - r\,S + k\,r\,S)\,z}{k\,(1 - r + r\,S)} + r\,(-1 + S)\,\alpha_1 +$$

$$k\Bigg(z - \frac{(-1 + k)\,r\,z}{k} + \frac{(-1 + k)\,r\,S\,z}{k} - \frac{(k + r - k\,r - r\,S + k\,r\,S)\,z}{k\,(1 - r + r\,S)} + \frac{r\,(k + r - k\,r - r\,S + k\,r\,S)\,z}{k\,(1 - r + r\,S)} -$$

$$\frac{r\,S\,(k + r - k\,r - r\,S + k\,r\,S)\,z}{k\,(1 - r + r\,S)} + \frac{(k + r - k\,r - r\,S + k\,r\,S)\,z}{k\,(1 - r + r\,S)\,\alpha_2}\Bigg) - \frac{r\,(k + r - k\,r - r\,S + k\,r\,S)\,z}{k\,(1 - r + r\,S)\,\alpha_2} +$$

$$\frac{r\,S\,(k + r - k\,r - r\,S + k\,r\,S)\,z}{k\,(1 - r + r\,S)\,\alpha_2} - \frac{(-1 + k)\,r\,(-1 + S)\,(r\,z - r\,S\,z + k\,\alpha_1 - k\,r\,\alpha_1 + k\,r\,S\,\alpha_1)\,(-1 + \beta_1)}{k\,(1 - r + r\,S)\,(-k - \beta_1 + k\,\beta_1)} -$$

$$(r - r\,S)\Bigg((-1 + k)\,z - \frac{(k + r - k\,r - r\,S + k\,r\,S)\,z}{1 - r + r\,S} - k\,\alpha_1 +$$

$$\frac{(-1 + k)\,(r\,z - r\,S\,z + k\,\alpha_1 - k\,r\,\alpha_1 + k\,r\,S\,\alpha_1)\,(-1 + \beta_1)}{(1 - r + r\,S)\,(-k - \beta_1 + k\,\beta_1)}\Bigg) + \beta_2 - r\,\beta_2 + r\,S\,\beta_2\Bigg)\,((-1 + k)\,(-1 + \beta_1) - \lambda_0) -$$

$$(r - r\,S)\Bigg((-1 + k)\,k\,(-1 + \beta_1)\Bigg(-\Bigg((-r + r\,S)\Bigg(-\frac{(-1 + k)\,z}{k} + \frac{(k + r - k\,r - r\,S + k\,r\,S)\,z}{k\,(1 - r + r\,S)}\Bigg)\Bigg) +$$

$$\alpha_1 - \frac{(-1 + k)\,(r\,z - r\,S\,z + k\,\alpha_1 - k\,r\,\alpha_1 + k\,r\,S\,\alpha_1)\,(-1 + \beta_1)}{k\,(1 - r + r\,S)\,(-k - \beta_1 + k\,\beta_1)}\Bigg) +$$

$$(-1 + k)\Bigg(\alpha_1 - \Bigg(z + \frac{(-1 + k)\,r\,(-1 + S)\,z}{k} - \frac{r\,(-1 + S)\,(k + r - k\,r - r\,S + k\,r\,S)\,z}{k\,(1 - r + r\,S)\,\alpha_2}\Bigg)\,(-1 + \beta_1) -$$

$$(-1+\beta_1)\Bigg((-1+k)\,z-\frac{(k+r-k\,r-r\,S+k\,r\,S)\,z}{1-r+r\,S}-k\,\alpha_1+$$

$$\frac{(-1+k)\,(r\,z-r\,S\,z+k\,\alpha_1-k\,r\,\alpha_1+k\,r\,S\,\alpha_1)\,(-1+\beta_1)}{(1-r+r\,S)\,(-k-\beta_1+k\,\beta_1)}\Bigg)\Bigg)-\lambda_1\Bigg)\Bigg)+\big((-1+k)\,(-1+r-r\,S)-\lambda_0\big)$$

$$\Bigg((-1+k)\,(r-r\,S)\,\alpha_2\Bigg(-k\Bigg(z-\frac{(-1+k)\,r\,z}{k}+\frac{(-1+k)\,r\,S\,z}{k}-\frac{(k+r-k\,r-r\,S+k\,r\,S)\,z}{k\,(1-r+r\,S)}+$$

$$\frac{r\,(k+r-k\,r-r\,S+k\,r\,S)\,z}{k\,(1-r+r\,S)}-\frac{r\,S\,(k+r-k\,r-r\,S+k\,r\,S)\,z}{k\,(1-r+r\,S)}+\frac{(k+r-k\,r-r\,S+k\,r\,S)\,z}{k\,(1-r+r\,S)\,\alpha_2}\Bigg)$$

$$(-1+\beta_1)+\Bigg(z+\frac{(-1+k)\,r\,(-1+S)\,z}{k}-\frac{r\,(-1+S)\,(k+r-k\,r-r\,S+k\,r\,S)\,z}{k\,(1-r+r\,S)\,\alpha_2}\Bigg)(-1+\beta_1)\Bigg)+$$

$$\big((-1+k)\,(-1+\beta_1)-\lambda_0\big)\Bigg(-y-z+\frac{(-1+k)\,r\,z}{k}-\frac{(-1+k)\,r\,S\,z}{k}-\frac{2\,r\,(k+r-k\,r-r\,S+k\,r\,S)\,z}{k\,(1-r+r\,S)}+$$

$$\frac{2\,r\,S\,(k+r-k\,r-r\,S+k\,r\,S)\,z}{k\,(1-r+r\,S)}+r\,(-1+S)\,\alpha_1+y\,\alpha_2-r\,y\,\alpha_2+r\,S\,y\,\alpha_2-r\,z\,\alpha_2+\frac{(-1+k)\,r\,z\,\alpha_2}{k}+$$

$$r\,S\,z\,\alpha_2-\frac{(-1+k)\,r\,S\,z\,\alpha_2}{k}+k\Bigg(z-\frac{(-1+k)\,r\,z}{k}+\frac{(-1+k)\,r\,S\,z}{k}-\frac{(k+r-k\,r-r\,S+k\,r\,S)\,z}{k\,(1-r+r\,S)}+$$

$$\frac{r\,(k+r-k\,r-r\,S+k\,r\,S)\,z}{k\,(1-r+r\,S)}-\frac{r\,S\,(k+r-k\,r-r\,S+k\,r\,S)\,z}{k\,(1-r+r\,S)}+\frac{(k+r-k\,r-r\,S+k\,r\,S)\,z}{k\,(1-r+r\,S)\,\alpha_2}\Bigg)\alpha_2-$$

$$\frac{(-1+k)\,r\,(-1+S)\,(r\,z-r\,S\,z+k\,\alpha_1-k\,r\,\alpha_1+k\,r\,S\,\alpha_1)\,(-1+\beta_1)}{k\,(1-r+r\,S)\,(-k-\beta_1+k\,\beta_1)}-(1-\alpha_2+r\,\alpha_2-r\,S\,\alpha_2)\Bigg((-1+k)\,z-$$

$$\frac{(k+r-k\,r-r\,S+k\,r\,S)\,z}{1-r+r\,S}-k\,\alpha_1+\frac{(-1+k)\,(r\,z-r\,S\,z+k\,\alpha_1-k\,r\,\alpha_1+k\,r\,S\,\alpha_1)\,(-1+\beta_1)}{(1-r+r\,S)\,(-k-\beta_1+k\,\beta_1)}\Bigg)-\lambda_1\Bigg)+$$

$$(1-\alpha_2+r\,\alpha_2-r\,S\,\alpha_2-\lambda_0)\Bigg((-1+k)\,k\,(-1+\beta_1)\Bigg(-\Bigg((-r+r\,S)\Bigg(-\frac{(-1+k)\,z}{k}+\frac{(k+r-k\,r-r\,S+k\,r\,S)\,z}{k\,(1-r+r\,S)}\Bigg)\Bigg)+$$

$$\alpha_1-\frac{(-1+k)\,(r\,z-r\,S\,z+k\,\alpha_1-k\,r\,\alpha_1+k\,r\,S\,\alpha_1)\,(-1+\beta_1)}{k\,(1-r+r\,S)\,(-k-\beta_1+k\,\beta_1)}\Bigg)+$$

$$(-1+k)\Bigg(\alpha_1-\Bigg(z+\frac{(-1+k)\,r\,(-1+S)\,z}{k}-\frac{r\,(-1+S)\,(k+r-k\,r-r\,S+k\,r\,S)\,z}{k\,(1-r+r\,S)\,\alpha_2}\Bigg)(-1+\beta_1)-$$

$$(-1+\beta_1)\Bigg((-1+k)\,z-\frac{(k+r-k\,r-r\,S+k\,r\,S)\,z}{1-r+r\,S}-k\,\alpha_1+$$

$$\frac{(-1+k)\,(r\,z-r\,S\,z+k\,\alpha_1-k\,r\,\alpha_1+k\,r\,S\,\alpha_1)\,(-1+\beta_1)}{(1-r+r\,S)\,(-k-\beta_1+k\,\beta_1)}\Bigg)\Bigg)-\lambda_1\Bigg)\Bigg)+(1-\alpha_2+r\,\alpha_2-r\,S\,\alpha_2-\lambda_0)$$

$$\big((-1+k)\,(-1+\beta_1)-\lambda_0\big)\Bigg(-\Bigg((-1+k)\,k\,(1-r+r\,S)\Bigg(-\frac{(-1+k)\,z}{k}+\frac{(k+r-k\,r-r\,S+k\,r\,S)\,z}{k\,(1-r+r\,S)}\Bigg)\Bigg)+$$

$$(-1+k)\Bigg(y-r\,y+r\,S\,y-r\,z-\frac{(-1+k)\,r\,(-1+S)\,z}{k}+r\,S\,z+r\,(-1+S)$$

$$\Bigg(-\frac{(-1+k)\,z}{k}+\frac{(k+r-k\,r-r\,S+k\,r\,S)\,z}{k\,(1-r+r\,S)}\Bigg)+r\,(-1+S)\,\alpha_1+\frac{r\,(-1+S)\,(k+r-k\,r-r\,S+k\,r\,S)\,z}{k\,(1-r+r\,S)\,\alpha_2}-$$

$$\frac{(-1+k)\,r\,(-1+S)\,(r\,z-r\,S\,z+k\,\alpha_1-k\,r\,\alpha_1+k\,r\,S\,\alpha_1)\,(-1+\beta_1)}{k\,(1-r+r\,S)\,(-k-\beta_1+k\,\beta_1)}-$$

$$(-1+r-r\,S)\Bigg((-1+k)\,z-\frac{(k+r-k\,r-r\,S+k\,r\,S)\,z}{1-r+r\,S}-k\,\alpha_1+$$

$$\frac{(-1+k)\,(r\,z-r\,S\,z+k\,\alpha_1-k\,r\,\alpha_1+k\,r\,S\,\alpha_1)\,(-1+\beta_1)}{(1-r+r\,S)\,(-k-\beta_1+k\,\beta_1)}\Bigg)+(1-r+r\,S)\,\beta_2\Bigg)-\lambda_1\Bigg)\Bigg)\ /.$$

$$\lambda_0\to\frac{1}{2}\Big(2-k-r+k\,r+r\,S-k\,r\,S-\alpha_2+r\,\alpha_2-r\,S\,\alpha_2+\sqrt{\big(-4\,(-1+k)\,(1+r\,(-1+S))\,(-1+\alpha_2)+}$$

$$\big(-2+k+r-k\,r-r\,S+k\,r\,S+(1+r\,(-1+S))\,\alpha_2\big)^2\big)\Big);$$

```
Solve[% == 0, λ1];
```

```
Simplify[%];
```

Plugging in the third zeroth order eigenvalue term and solving for the first order eigenvalue term:

$$\Bigg(-\Bigg(\Bigg((-1+k)\,k\,(1-r+r\,S)\Bigg(-\frac{(-1+k)\,z}{k}+\frac{(k+r-k\,r-r\,S+k\,r\,S)\,z}{k\,(1-r+r\,S)}\Bigg)(-1+\beta_1)+$$

$$(-1+k)\Bigg(z+\frac{(-1+k)\,r\,(-1+S)\,z}{k}-\frac{r\,(-1+S)\,(k+r-k\,r-r\,S+k\,r\,S)\,z}{k\,(1-r+r\,S)\,\alpha_2}\Bigg)(-1+\beta_1)\Bigg)$$

$$\big((-1+k)\,(r-r\,S)^2\,\alpha_2-(-1+k)\,(r-r\,S)\,(1-\alpha_2+r\,\alpha_2-r\,S\,\alpha_2-\lambda_0)\big)\Bigg)-$$

$$(r-r\,S)\Bigg(-\Bigg((-1+k)\,k\,(1-r+r\,S)\Bigg(-\frac{(-1+k)\,z}{k}+\frac{(k+r-k\,r-r\,S+k\,r\,S)\,z}{k\,(1-r+r\,S)}\Bigg)\alpha_2\Bigg)+$$

$$(-1+k)\Bigg(r\,(-1+S)\Bigg(-\frac{(-1+k)\,z}{k}+\frac{(k+r-k\,r-r\,S+k\,r\,S)\,z}{k\,(1-r+r\,S)}\Bigg)+r\,(-1+S)\,\alpha_1+$$

$$\Bigg(y - r\,y + r\,S\,y - r\,z - \frac{(-1+k)\,r\,(-1+S)\,z}{k} + r\,S\,z + \frac{r\,(-1+S)\,(k+r-k\,r-r\,S+k\,r\,S)\,z}{k\,(1-r+r\,S)\,\alpha_2}\Bigg)\,\alpha_2 -$$

$$\frac{(-1+k)\,r\,(-1+S)\,(r\,z - r\,S\,z + k\,\alpha_1 - k\,r\,\alpha_1 + k\,r\,S\,\alpha_1)\,(-1+\beta_1)}{k\,(1-r+r\,S)\,(-k-\beta_1+k\,\beta_1)} -$$

$$(-1+r-r\,S)\,\alpha_2\,\Bigg((-1+k)\,z - \frac{(k+r-k\,r-r\,S+k\,r\,S)\,z}{1-r+r\,S} - k\,\alpha_1 +$$

$$\frac{(-1+k)\,(r\,z - r\,S\,z + k\,\alpha_1 - k\,r\,\alpha_1 + k\,r\,S\,\alpha_1)\,(-1+\beta_1)}{(1-r+r\,S)\,(-k-\beta_1+k\,\beta_1)}\Bigg)\Bigg)\Bigg)\,\big((-1+k)\,(-1+\beta_1) - \lambda_0\big) +$$

$$(-1+k)\,(-1+r-r\,S)\,\alpha_2\,\Bigg(-\Bigg((-1+k)\,(r-r\,S)\,\Bigg(-k\,\Bigg(z - \frac{(-1+k)\,r\,z}{k} + \frac{(-1+k)\,r\,S\,z}{k} - \frac{(k+r-k\,r-r\,S+k\,r\,S)\,z}{k\,(1-r+r\,S)} +$$

$$\frac{r\,(k+r-k\,r-r\,S+k\,r\,S)\,z}{k\,(1-r+r\,S)} - \frac{r\,S\,(k+r-k\,r-r\,S+k\,r\,S)\,z}{k\,(1-r+r\,S)} + \frac{(k+r-k\,r-r\,S+k\,r\,S)\,z}{k\,(1-r+r\,S)\,\alpha_2}\Bigg)$$

$$(-1+\beta_1) + \Bigg(z + \frac{(-1+k)\,r\,(-1+S)\,z}{k} - \frac{r\,(-1+S)\,(k+r-k\,r-r\,S+k\,r\,S)\,z}{k\,(1-r+r\,S)\,\alpha_2}\Bigg)\,(-1+\beta_1)\Bigg)\Bigg) -$$

$$\Bigg(-r\,y + r\,S\,y - z - r\,z + \frac{2\,(-1+k)\,r\,z}{k} + r\,S\,z - \frac{2\,(-1+k)\,r\,S\,z}{k} - \frac{r\,(k+r-k\,r-r\,S+k\,r\,S)\,z}{k\,(1-r+r\,S)} +$$

$$\frac{r\,S\,(k+r-k\,r-r\,S+k\,r\,S)\,z}{k\,(1-r+r\,S)} + r\,(-1+S)\,\alpha_1 +$$

$$k\,\Bigg(z - \frac{(-1+k)\,r\,z}{k} + \frac{(-1+k)\,r\,S\,z}{k} - \frac{(k+r-k\,r-r\,S+k\,r\,S)\,z}{k\,(1-r+r\,S)} + \frac{r\,(k+r-k\,r-r\,S+k\,r\,S)\,z}{k\,(1-r+r\,S)} -$$

$$\frac{r\,S\,(k+r-k\,r-r\,S+k\,r\,S)\,z}{k\,(1-r+r\,S)} + \frac{(k+r-k\,r-r\,S+k\,r\,S)\,z}{k\,(1-r+r\,S)\,\alpha_2}\Bigg) - \frac{r\,(k+r-k\,r-r\,S+k\,r\,S)\,z}{k\,(1-r+r\,S)\,\alpha_2} +$$

$$\frac{r\,S\,(k+r-k\,r-r\,S+k\,r\,S)\,z}{k\,(1-r+r\,S)\,\alpha_2} - \frac{(-1+k)\,r\,(-1+S)\,(r\,z - r\,S\,z + k\,\alpha_1 - k\,r\,\alpha_1 + k\,r\,S\,\alpha_1)\,(-1+\beta_1)}{k\,(1-r+r\,S)\,(-k-\beta_1+k\,\beta_1)} -$$

$$(r-r\,S)\,\Bigg((-1+k)\,z - \frac{(k+r-k\,r-r\,S+k\,r\,S)\,z}{1-r+r\,S} - k\,\alpha_1 +$$

$$\frac{(-1+k)\,(r\,z - r\,S\,z + k\,\alpha_1 - k\,r\,\alpha_1 + k\,r\,S\,\alpha_1)\,(-1+\beta_1)}{(1-r+r\,S)\,(-k-\beta_1+k\,\beta_1)}\Bigg) + \beta_2 - r\,\beta_2 + r\,S\,\beta_2\Bigg)\,\big((-1+k)\,(-1+\beta_1) - \lambda_0\big) -$$

$$(r-r\,S)\,\Bigg((-1+k)\,k\,(-1+\beta_1)\,\Bigg(-\Bigg((-r+r\,S)\,\Bigg(-\frac{(-1+k)\,z}{k} + \frac{(k+r-k\,r-r\,S+k\,r\,S)\,z}{k\,(1-r+r\,S)}\Bigg)\Bigg) +$$

$$\alpha_1 - \frac{(-1+k)\big(r z - r S z + k \alpha_1 - k r \alpha_1 + k r S \alpha_1\big)(-1+\beta_1)}{k\big(1-r+r S\big)\big(-k-\beta_1+k \beta_1\big)}\Bigg) +$$

$$(-1+k)\Bigg(\alpha_1 - \Bigg(z + \frac{(-1+k) r (-1+S) z}{k} - \frac{r(-1+S)\big(k+r-k r-r S+k r S\big) z}{k\big(1-r+r S\big)\alpha_2}\Bigg)(-1+\beta_1) -$$

$$(-1+\beta_1)\Bigg((-1+k) z - \frac{\big(k+r-k r-r S+k r S\big) z}{1-r+r S} - k \alpha_1 +$$

$$\frac{(-1+k)\big(r z - r S z + k \alpha_1 - k r \alpha_1 + k r S \alpha_1\big)(-1+\beta_1)}{\big(1-r+r S\big)\big(-k-\beta_1+k \beta_1\big)}\Bigg)\Bigg) - \lambda_1\Bigg)\Bigg) + \Big((-1+k)\big(-1+r-r S\big) - \lambda_0\Big)$$

$$\Bigg((-1+k)\big(r - r S\big)\alpha_2\Bigg(-k\Bigg(z - \frac{(-1+k) r z}{k} + \frac{(-1+k) r S z}{k} - \frac{\big(k+r-k r-r S+k r S\big) z}{k\big(1-r+r S\big)} +$$

$$\frac{r\big(k+r-k r-r S+k r S\big) z}{k\big(1-r+r S\big)} - \frac{r S\big(k+r-k r-r S+k r S\big) z}{k\big(1-r+r S\big)} + \frac{\big(k+r-k r-r S+k r S\big) z}{k\big(1-r+r S\big)\alpha_2}\Bigg)$$

$$(-1+\beta_1) + \Bigg(z + \frac{(-1+k) r (-1+S) z}{k} - \frac{r(-1+S)\big(k+r-k r-r S+k r S\big) z}{k\big(1-r+r S\big)\alpha_2}\Bigg)(-1+\beta_1)\Bigg) +$$

$$\Big((-1+k)(-1+\beta_1) - \lambda_0\Big)\Bigg(-y - z + \frac{(-1+k) r z}{k} - \frac{(-1+k) r S z}{k} - \frac{2 r\big(k+r-k r-r S+k r S\big) z}{k\big(1-r+r S\big)} +$$

$$\frac{2 r S\big(k+r-k r-r S+k r S\big) z}{k\big(1-r+r S\big)} + r(-1+S)\alpha_1 + y \alpha_2 - r y \alpha_2 + r S y \alpha_2 - r z \alpha_2 + \frac{(-1+k) r z \alpha_2}{k} +$$

$$r S z \alpha_2 - \frac{(-1+k) r S z \alpha_2}{k} + k\Bigg(z - \frac{(-1+k) r z}{k} + \frac{(-1+k) r S z}{k} - \frac{\big(k+r-k r-r S+k r S\big) z}{k\big(1-r+r S\big)} +$$

$$\frac{r\big(k+r-k r-r S+k r S\big) z}{k\big(1-r+r S\big)} - \frac{r S\big(k+r-k r-r S+k r S\big) z}{k\big(1-r+r S\big)} + \frac{\big(k+r-k r-r S+k r S\big) z}{k\big(1-r+r S\big)\alpha_2}\Bigg)\alpha_2 -$$

$$\frac{(-1+k) r (-1+S)\big(r z - r S z + k \alpha_1 - k r \alpha_1 + k r S \alpha_1\big)(-1+\beta_1)}{k\big(1-r+r S\big)\big(-k-\beta_1+k \beta_1\big)} - \big(1 - \alpha_2 + r \alpha_2 - r S \alpha_2\big)\Bigg(\Bigg((-1+k) z -$$

$$\frac{\big(k+r-k r-r S+k r S\big) z}{1-r+r S} - k \alpha_1 + \frac{(-1+k)\big(r z - r S z + k \alpha_1 - k r \alpha_1 + k r S \alpha_1\big)(-1+\beta_1)}{\big(1-r+r S\big)\big(-k-\beta_1+k \beta_1\big)}\Bigg) - \lambda_1\Bigg) +$$

$$\big(1 - \alpha_2 + r \alpha_2 - r S \alpha_2 - \lambda_0\big)\Bigg((-1+k) k (-1+\beta_1)\Bigg(-\Bigg(\big(-r + r S\big)\Bigg(-\frac{(-1+k) z}{k} + \frac{\big(k+r-k r-r S+k r S\big) z}{k\big(1-r+r S\big)}\Bigg)\Bigg) +$$

$$\alpha_1 - \frac{(-1+k)\big(r z - r S z + k \alpha_1 - k r \alpha_1 + k r S \alpha_1\big)(-1+\beta_1)}{k\big(1-r+r S\big)\big(-k-\beta_1+k \beta_1\big)}\Bigg) +$$

$$(-1+k)\left(\alpha_1-\left(z+\frac{(-1+k)\,r\,(-1+S)\,z}{k}-\frac{r\,(-1+S)\left(k+r-k\,r-r\,S+k\,r\,S\right)z}{k\left(1-r+r\,S\right)\alpha_2}\right)(-1+\beta_1)-\right.$$

$$(-1+\beta_1)\left((-1+k)\,z-\frac{\left(k+r-k\,r-r\,S+k\,r\,S\right)z}{1-r+r\,S}-k\,\alpha_1+\right.$$

$$\left.\left.\frac{(-1+k)\left(r\,z-r\,S\,z+k\,\alpha_1-k\,r\,\alpha_1+k\,r\,S\,\alpha_1\right)(-1+\beta_1)}{\left(1-r+r\,S\right)\left(-k-\beta_1+k\,\beta_1\right)}\right)\right)-\lambda_1\Bigg)\Bigg)+\left(1-\alpha_2+r\,\alpha_2-r\,S\,\alpha_2-\lambda_0\right)$$

$$\left((-1+k)\,(-1+\beta_1)-\lambda_0\right)\left(-\left((-1+k)\,k\left(1-r+r\,S\right)\left(-\frac{(-1+k)\,z}{k}+\frac{\left(k+r-k\,r-r\,S+k\,r\,S\right)z}{k\left(1-r+r\,S\right)}\right)\right)+\right.$$

$$(-1+k)\left(y-r\,y+r\,S\,y-r\,z-\frac{(-1+k)\,r\,(-1+S)\,z}{k}+r\,S\,z+r\,(-1+S)\right.$$

$$\left(-\frac{(-1+k)\,z}{k}+\frac{\left(k+r-k\,r-r\,S+k\,r\,S\right)z}{k\left(1-r+r\,S\right)}\right)+r\,(-1+S)\,\alpha_1+\frac{r\,(-1+S)\left(k+r-k\,r-r\,S+k\,r\,S\right)z}{k\left(1-r+r\,S\right)\alpha_2}-$$

$$\frac{(-1+k)\,r\,(-1+S)\left(r\,z-r\,S\,z+k\,\alpha_1-k\,r\,\alpha_1+k\,r\,S\,\alpha_1\right)(-1+\beta_1)}{k\left(1-r+r\,S\right)\left(-k-\beta_1+k\,\beta_1\right)}-\left(-1+r-r\,S\right)\left((-1+k)\,z-\right.$$

$$\left.\frac{\left(k+r-k\,r-r\,S+k\,r\,S\right)z}{1-r+r\,S}-k\,\alpha_1+\frac{(-1+k)\left(r\,z-r\,S\,z+k\,\alpha_1-k\,r\,\alpha_1+k\,r\,S\,\alpha_1\right)(-1+\beta_1)}{\left(1-r+r\,S\right)\left(-k-\beta_1+k\,\beta_1\right)}\right)+$$

$$\left.\left.\left(1-r+r\,S\right)\beta_2\right)-\lambda_1\right)\Bigg)\ \text{/. }\lambda_0\to(-1+k)\,(-1+\beta_1);$$

```
Solve[%39 == 0, λ1];
```

In[∘]:= `Simplify[%]`

Out[∘]=

$$\Big\{\Big\{\lambda_1\to$$

$$-\Big(\Big((-1+k)\left(-r\,(-1+S)\left(k+r+k\,r\,(-1+S)-r\,S\right)z\,(-1+\beta_1)\,\beta_1\left(k-(-1+k)\,\beta_1\right)^2-\left(1+r\,(-1+S)\right)\alpha_2^2\left(k^2\,r\,(-1+S)\,z+\right.\right.$$

$$k^2\left(-2\,r\,(-1+S)\,z+\left(1+r\,(-1+S)\right)\alpha_1\right)\beta_1+\left(\left(1-k+k^2\right)r\,(-1+S)\,z-k\,(1+k)\left(1+r\,(-1+S)\right)\alpha_1\right)\beta_1^2+$$

$$\left.(-1+k)\,r\,(-1+S)\,z\,\beta_1^3\right)+\alpha_2\left(-k+(-1+k)\,\beta_1\right)$$

$$\left(k\left(1+r\,(-1+S)\right)\alpha_1\left(r\,(-1+S)+\beta_1\right)\left(-k+(1+k)\,\beta_1\right)+r\,(-1+S)\,z\,(-1+\beta_1)\left(k\left(k+r+k\,r\,(-1+S)-r\,S\right)-\right.\right.$$

$$\left.\left.\left.\left(r+2\,k^2\left(1+r\,(-1+S)\right)-r\,S+k\left(r-r\,S\right)\right)\beta_1+(-1+k)^2\left(1+r\,(-1+S)\right)\beta_1^2\right)\right)\right)\Big)\Big/\Big(k\left(1+r\,(-1+S)\right)$$

$$\alpha_2\left(-k+(-1+k)\,\beta_1\right)\left(-k\,r\,(-1+S)+\left(r+k\left(-1+r\,(-1+S)\right)-r\,S+\left(1+r\,(-1+S)\right)\alpha_2\right)\beta_1+(-1+k)\,\beta_1^2\right)\Big)\Big\}\Big\}$$

A4 - Numerically evaluating accuracy of equilibria approximations

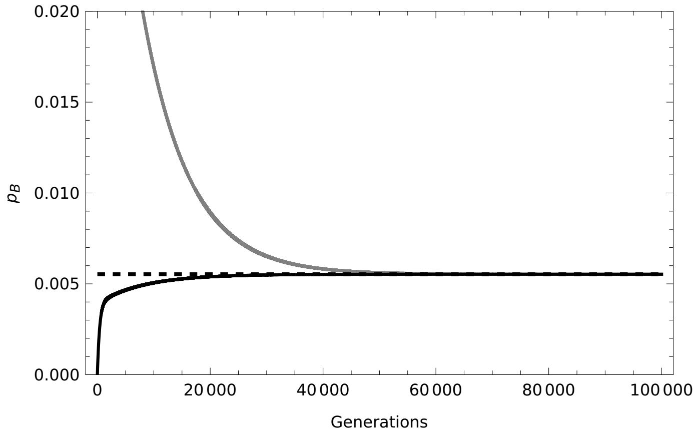

Figure S22: Numerical simulations of the recursion equations to assess equilibrium stability. The dashed line is the equilibrium point, the gray line starts above the equilibrium (p_B= 0.02), and the black line starts below the equilibrium (p_B= 2x10^{-6}). Other parameter values are: $\alpha_1 = 10^{-5}$, $\alpha_2 = 0.005$, $\beta_1 = 0.0005$, $\beta_2 = 10^{-5}$, $z = 10^{-7}$, $y = 10^{-5}$, S = 0.25, k = 0.002, r = 0.1.

Case B: The transposon and epiallele are deleterious

B1 - Simplified Recursion Equations

Fitness assumptions:

Haploid Genotype	Fitness
tA	$w_{tA} = 1$
tB	$w_{tB} = 1 - k$
TA	$w_{TA} = 1 - \mathcal{L}$
TB	$w_{TB} = 1 - \mathcal{L} - k$

After inputting the fitness assumptions above, reducing dimensions and simplifying, the recursion equation are

$$p_{T,A}' = (z((-1+S)(-1+\alpha_1)(1-p_{t,B}-p_{T,A}-p_{T,B})^2 +$$
$$(1-p_{t,B}-p_{T,A}-p_{T,B})(S+p_{t,B}-S\,p_{t,B}+\beta_1\,p_{t,B}-S\,\beta_1\,p_{t,B}+p_{T,A}-S\,p_{T,A}-r\,\alpha_2\,p_{T,A}+r\,S\,\alpha_2\,p_{T,A}+p_{T,B}-r\,p_{T,B}-S\,p_{T,B}+$$
$$r\,S\,p_{T,B}+r\,\beta_2\,p_{T,B}-r\,S\,\beta_2\,p_{T,B}+\alpha_1(-S+(-1+S)\,p_{t,B}-(-1+r)(-1+S)\,p_{T,A}-p_{T,B}+r\,p_{T,B}+S\,p_{T,B}-r\,S\,p_{T,B}))+$$
$$p_{t,B}(\beta_1(S-(-1+S)\,p_{t,B}+(-1+r)(-1+S)\,p_{T,A}+p_{T,B}-r\,p_{T,B}-S\,p_{T,B}+r\,S\,p_{T,B})+r(-1+S)((-1+\alpha_2)\,p_{T,A}-\beta_2\,p_{T,B})))+$$
$$(-1+y)(-((-1+S)(-1+\alpha_2)\,p_{T,A}^2)+p_{T,B}(-r(-1+S)(-\beta_1\,p_{t,B}+(-1+\alpha_1)(1-p_{t,B}-p_{T,A}-p_{T,B}))+$$
$$\beta_2(-S-(-1+r)(-1+S)\,p_{t,B}-(-1+r)(-1+S)(1-p_{t,B}-p_{T,A}-p_{T,B})+(-1+S)\,p_{T,B}))-$$
$$p_{T,A}(1+S-r\,p_{t,B}-S\,p_{t,B}+r\,S\,p_{t,B}+r\,\beta_1\,p_{t,B}-r\,S\,\beta_1\,p_{t,B}-p_{T,A}-S(1-p_{t,B}-p_{T,A}-p_{T,B})-$$
$$r\,\alpha_1(1-p_{t,B}-p_{T,A}-p_{T,B})+r\,S\,\alpha_1(1-p_{t,B}-p_{T,A}-p_{T,B})-S\,p_{T,B}+\beta_2\,p_{T,B}-S\,\beta_2\,p_{T,B}+$$
$$\alpha_2(-S-(-1+r)(-1+S)\,p_{t,B}-(-1+r)(-1+S)(1-p_{t,B}-p_{T,A}-p_{T,B})-p_{T,B}+S\,p_{T,B})))\,w_{T,A})/$$
$$((-1+S)(-1+\alpha_1)(1-p_{t,B}-p_{T,A}-p_{T,B})^2+(1-p_{t,B}-p_{T,A}-p_{T,B})(S+p_{t,B}-S\,p_{t,B}+\beta_1\,p_{t,B}-S\,\beta_1\,p_{t,B}+p_{T,A}-S\,p_{T,A}-$$
$$r\,\alpha_2\,p_{T,A}+r\,S\,\alpha_2\,p_{T,A}+p_{T,B}-r\,p_{T,B}-S\,p_{T,B}+r\,S\,p_{T,B}+r\,\beta_2\,p_{T,B}-r\,S\,\beta_2\,p_{T,B}+$$
$$\alpha_1(-S+(-1+S)\,p_{t,B}-(-1+r)(-1+S)\,p_{T,A}-p_{T,B}+r\,p_{T,B}+S\,p_{T,B}-r\,S\,p_{T,B}))+$$
$$p_{t,B}(\beta_1(S-(-1+S)\,p_{t,B}+(-1+r)(-1+S)\,p_{T,A}+p_{T,B}-r\,p_{T,B}-S\,p_{T,B}+r\,S\,p_{T,B})+r(-1+S)((-1+\alpha_2)\,p_{T,A}-\beta_2\,p_{T,B}))+$$

$$\left((-1+S)\,(-1+\beta_1)\,p_{t,B}^2+\alpha_1\,(1-p_{t,B}-p_{T,A}-p_{T,B})\,\Big(S-(-1+S)\,p_{t,B}+p_{T,A}-r\,p_{T,A}-S\,p_{T,A}+r\,S\,p_{T,A}-(-1+S)\,(1-p_{t,B}-p_{T,A}-p_{T,B})+\right.$$

$$p_{T,B}-r\,p_{T,B}-S\,p_{T,B}+r\,S\,p_{T,B}\Big)-r\,(-1+S)\,(1-p_{t,B}-p_{T,A}-p_{T,B})\,\big(\alpha_2\,p_{T,A}-(-1+\beta_2)\,p_{T,B}\big)+$$

$$p_{t,B}\,\Big(1+S-p_{t,B}-r\,p_{T,A}-S\,p_{T,A}+r\,S\,p_{T,A}+r\,\alpha_2\,p_{T,A}-r\,S\,\alpha_2\,p_{T,A}-S\,(1-p_{t,B}-p_{T,A}-p_{T,B})-S\,p_{T,B}-r\,\beta_2\,p_{T,B}+$$

$$r\,S\,\beta_2\,p_{T,B}+\beta_1\,\big(-S-(-1+r)\,(-1+S)\,p_{T,A}+(-1+S)\,(1-p_{t,B}-p_{T,A}-p_{T,B})-p_{T,B}+r\,p_{T,B}+S\,p_{T,B}-r\,S\,p_{T,B}\big)\Big)\Big)\,w_{t,B}+$$

$$S\,p_{T,A}\,w_{T,A}-S\,\alpha_2\,p_{T,A}\,w_{T,A}+p_{t,B}\,p_{T,A}\,w_{T,A}-r\,p_{t,B}\,p_{T,A}\,w_{T,A}-S\,p_{t,B}\,p_{T,A}\,w_{T,A}+r\,S\,p_{t,B}\,p_{T,A}\,w_{T,A}-$$

$$\alpha_2\,p_{t,B}\,p_{T,A}\,w_{T,A}+r\,\alpha_2\,p_{t,B}\,p_{T,A}\,w_{T,A}+S\,\alpha_2\,p_{t,B}\,p_{T,A}\,w_{T,A}-$$

$$r\,S\,\alpha_2\,p_{t,B}\,p_{T,A}\,w_{T,A}+r\,\beta_1\,p_{t,B}\,p_{T,A}\,w_{T,A}-$$

$$r\,S\,\beta_1\,p_{t,B}\,p_{T,A}\,w_{T,A}+p_{T,A}^2\,w_{T,A}-S\,p_{T,A}^2\,w_{T,A}-\alpha_2\,p_{T,A}^2\,w_{T,A}+$$

$$S\,\alpha_2\,p_{T,A}^2\,w_{T,A}+p_{T,A}\,(1-p_{t,B}-p_{T,A}-p_{T,B})\,w_{T,A}-$$

$$S\,p_{T,A}\,(1-p_{t,B}-p_{T,A}-p_{T,B})\,w_{T,A}-r\,\alpha_1\,p_{T,A}\,(1-p_{t,B}-p_{T,A}-p_{T,B})\,w_{T,A}+$$

$$r\,S\,\alpha_1\,p_{T,A}\,(1-p_{t,B}-p_{T,A}-p_{T,B})\,w_{T,A}-\alpha_2\,p_{T,A}\,(1-p_{t,B}-p_{T,A}-p_{T,B})\,w_{T,A}+$$

$$r\,\alpha_2\,p_{T,A}\,(1-p_{t,B}-p_{T,A}-p_{T,B})\,w_{T,A}+S\,\alpha_2\,p_{T,A}\,(1-p_{t,B}-p_{T,A}-p_{T,B})\,w_{T,A}-$$

$$r\,S\,\alpha_2\,p_{T,A}\,(1-p_{t,B}-p_{T,A}-p_{T,B})\,w_{T,A}+S\,\beta_2\,p_{T,B}\,w_{T,A}+r\,\beta_1\,p_{t,B}\,p_{T,B}\,w_{T,A}-$$

$$r\,S\,\beta_1\,p_{t,B}\,p_{T,B}\,w_{T,A}+\beta_2\,p_{t,B}\,p_{T,B}\,w_{T,A}-r\,\beta_2\,p_{t,B}\,p_{T,B}\,w_{T,A}-S\,\beta_2\,p_{t,B}\,p_{T,B}\,w_{T,A}+$$

$$r\,S\,\beta_2\,p_{t,B}\,p_{T,B}\,w_{T,A}+p_{T,A}\,p_{T,B}\,w_{T,A}-S\,p_{T,A}\,p_{T,B}\,w_{T,A}-\alpha_2\,p_{T,A}\,p_{T,B}\,w_{T,A}+$$

$$S\,\alpha_2\,p_{T,A}\,p_{T,B}\,w_{T,A}+\beta_2\,p_{T,A}\,p_{T,B}\,w_{T,A}-S\,\beta_2\,p_{T,A}\,p_{T,B}\,w_{T,A}+r\,(1-p_{t,B}-p_{T,A}-p_{T,B})\,p_{T,B}\,w_{T,A}-$$

$$r\,S\,(1-p_{t,B}-p_{T,A}-p_{T,B})\,p_{T,B}\,w_{T,A}-r\,\alpha_1\,(1-p_{t,B}-p_{T,A}-p_{T,B})\,p_{T,B}\,w_{T,A}+$$

$$r\,S\,\alpha_1\,(1-p_{t,B}-p_{T,A}-p_{T,B})\,p_{T,B}\,w_{T,A}+\beta_2\,(1-p_{t,B}-p_{T,A}-p_{T,B})\,p_{T,B}\,w_{T,A}-$$

$$r\,\beta_2\,(1-p_{t,B}-p_{T,A}-p_{T,B})\,p_{T,B}\,w_{T,A}-S\,\beta_2\,(1-p_{t,B}-p_{T,A}-p_{T,B})\,p_{T,B}\,w_{T,A}+$$

$$r\,S\,\beta_2\,(1-p_{t,B}-p_{T,A}-p_{T,B})\,p_{T,B}\,w_{T,A}+\beta_2\,p_{T,B}^2\,w_{T,A}-S\,\beta_2\,p_{T,B}^2\,w_{T,A}+S\,\alpha_2\,p_{T,A}\,w_{T,B}+$$

$$r\,p_{t,B}\,p_{T,A}\,w_{T,B}-r\,S\,p_{t,B}\,p_{T,A}\,w_{T,B}+\alpha_2\,p_{t,B}\,p_{T,A}\,w_{T,B}-r\,\alpha_2\,p_{t,B}\,p_{T,A}\,w_{T,B}-S\,\alpha_2\,p_{t,B}\,p_{T,A}\,w_{T,B}+$$

$$r\,S\,\alpha_2\,p_{t,B}\,p_{T,A}\,w_{T,B}-r\,\beta_1\,p_{t,B}\,p_{T,A}\,w_{T,B}+r\,S\,\beta_1\,p_{t,B}\,p_{T,A}\,w_{T,B}+\alpha_2\,p_{T,A}^2\,w_{T,B}-$$

$$S\,\alpha_2\,p_{T,A}^2\,w_{T,B}+r\,\alpha_1\,p_{T,A}\,(1-p_{t,B}-p_{T,A}-p_{T,B})\,w_{T,B}-r\,S\,\alpha_1\,p_{T,A}\,(1-p_{t,B}-p_{T,A}-p_{T,B})\,w_{T,B}+$$

$$\alpha_2\,p_{T,A}\,(1-p_{t,B}-p_{T,A}-p_{T,B})\,w_{T,B}-r\,\alpha_2\,p_{T,A}\,(1-p_{t,B}-p_{T,A}-p_{T,B})\,w_{T,B}-S\,\alpha_2\,p_{T,A}\,(1-p_{t,B}-p_{T,A}-p_{T,B})\,w_{T,B}+$$

$$r\,S\,\alpha_2\,p_{T,A}\,(1-p_{t,B}-p_{T,A}-p_{T,B})\,w_{T,B}+S\,p_{T,B}\,w_{T,B}-S\,\beta_2\,p_{T,B}\,w_{T,B}+p_{t,B}\,p_{T,B}\,w_{T,B}-$$

$$S\,p_{t,B}\,p_{T,B}\,w_{T,B}-r\,\beta_1\,p_{t,B}\,p_{T,B}\,w_{T,B}+r\,S\,\beta_1\,p_{t,B}\,p_{T,B}\,w_{T,B}-\beta_2\,p_{t,B}\,p_{T,B}\,w_{T,B}+r\,\beta_2\,p_{t,B}\,p_{T,B}\,w_{T,B}+$$

$$S\,\beta_2\,p_{t,B}\,p_{T,B}\,w_{T,B}-r\,S\,\beta_2\,p_{t,B}\,p_{T,B}\,w_{T,B}+p_{T,A}\,p_{T,B}\,w_{T,B}-S\,p_{T,A}\,p_{T,B}\,w_{T,B}+\alpha_2\,p_{T,A}\,p_{T,B}\,w_{T,B}-$$

$$S\,\alpha_2\,p_{T,A}\,p_{T,B}\,w_{T,B}-\beta_2\,p_{T,A}\,p_{T,B}\,w_{T,B}+S\,\beta_2\,p_{T,A}\,p_{T,B}\,w_{T,B}+(1-p_{t,B}-p_{T,A}-p_{T,B})\,p_{T,B}\,w_{T,B}-$$

$$r\,(1-p_{t,B}-p_{T,A}-p_{T,B})\,p_{T,B}\,w_{T,B}-S\,(1-p_{t,B}-p_{T,A}-p_{T,B})\,p_{T,B}\,w_{T,B}+r\,S\,(1-p_{t,B}-p_{T,A}-p_{T,B})\,p_{T,B}\,w_{T,B}+$$

$$r\,\alpha_1\,(1-p_{t,B}-p_{T,A}-p_{T,B})\,p_{T,B}\,w_{T,B}-r\,S\,\alpha_1\,(1-p_{t,B}-p_{T,A}-p_{T,B})\,p_{T,B}\,w_{T,B}-$$

$$\beta_2\,(1-p_{t,B}-p_{T,A}-p_{T,B})\,p_{T,B}\,w_{T,B}+r\,\beta_2\,(1-p_{t,B}-p_{T,A}-p_{T,B})\,p_{T,B}\,w_{T,B}+S\,\beta_2\,(1-p_{t,B}-p_{T,A}-p_{T,B})\,p_{T,B}\,w_{T,B}-$$

$$\left.r\,S\,\beta_2\,(1-p_{t,B}-p_{T,A}-p_{T,B})\,p_{T,B}\,w_{T,B}+p_{T,B}^2\,w_{T,B}-S\,p_{T,B}^2\,w_{T,B}-\beta_2\,p_{T,B}^2\,w_{T,B}+S\,\beta_2\,p_{T,B}^2\,w_{T,B}\right),$$

$$p_{t,B}{}' =$$

$$\Big((-1+z)\,\Big(-\big((-1+S)\,(-1+\beta_1)\,p_{t,B}^2\big)+\alpha_1\,(1-p_{t,B}-p_{T,A}-p_{T,B})\,\Big(-S+(-1+S)\,p_{t,B}-p_{T,A}+r\,p_{T,A}+S\,p_{T,A}-r\,S\,p_{T,A}+(-1+S)\,(1-p_{t,B}-p_{T,A}-p_{T,B})-$$

$$p_{T,B}+r\,p_{T,B}+S\,p_{T,B}-r\,S\,p_{T,B}\Big)+r\,(-1+S)\,(1-p_{t,B}-p_{T,A}-p_{T,B})\,\big(\alpha_2\,p_{T,A}-(-1+\beta_2)\,p_{T,B}\big)-$$

$$p_{t,B}\,\Big(1+S-p_{t,B}-r\,p_{T,A}-S\,p_{T,A}+r\,S\,p_{T,A}+r\,\alpha_2\,p_{T,A}-r\,S\,\alpha_2\,p_{T,A}-S\,(1-p_{t,B}-p_{T,A}-p_{T,B})-S\,p_{T,B}-r\,\beta_2\,p_{T,B}+$$

$$r\,S\,\beta_2\,p_{T,B}+\beta_1\,\big(-S-(-1+r)\,(-1+S)\,p_{T,A}+(-1+S)\,(1-p_{t,B}-p_{T,A}-p_{T,B})-p_{T,B}+r\,p_{T,B}+S\,p_{T,B}-r\,S\,p_{T,B}\big)\Big)\Big)\,w_{t,B}+$$

$$y\,\Big(r\,p_{t,B}\,p_{T,A}-r\,S\,p_{t,B}\,p_{T,A}-r\,\beta_1\,p_{t,B}\,p_{T,A}+r\,S\,\beta_1\,p_{t,B}\,p_{T,A}+S\,p_{T,B}-S\,\beta_2\,p_{T,B}+p_{t,B}\,p_{T,B}-S\,p_{t,B}\,p_{T,B}-r\,\beta_1\,p_{t,B}\,p_{T,B}+$$

$$r\,S\,\beta_1\,p_{t,B}\,p_{T,B}-\beta_2\,p_{t,B}\,p_{T,B}+r\,\beta_2\,p_{t,B}\,p_{T,B}+S\,\beta_2\,p_{t,B}\,p_{T,B}-r\,S\,\beta_2\,p_{t,B}\,p_{T,B}+p_{T,A}\,p_{T,B}-S\,p_{T,A}\,p_{T,B}-\beta_2\,p_{T,A}\,p_{T,B}+$$

$$S\,\beta_2\,p_{T,A}\,p_{T,B}+(1-p_{t,B}-p_{T,A}-p_{T,B})\,p_{T,B}-r\,(1-p_{t,B}-p_{T,A}-p_{T,B})\,p_{T,B}-S\,(1-p_{t,B}-p_{T,A}-p_{T,B})\,p_{T,B}+$$

$$r\,S\,(1-p_{t,B}-p_{T,A}-p_{T,B})\,p_{T,B}-\beta_2\,(1-p_{t,B}-p_{T,A}-p_{T,B})\,p_{T,B}+r\,\beta_2\,(1-p_{t,B}-p_{T,A}-p_{T,B})\,p_{T,B}+S\,\beta_2\,(1-p_{t,B}-p_{T,A}-p_{T,B})\,p_{T,B}-$$

$$r\,S\,\beta_2\,(1-p_{t,B}-p_{T,A}-p_{T,B})\,p_{T,B}+p_{T,B}^2-S\,p_{T,B}^2-\beta_2\,p_{T,B}^2+S\,\beta_2\,p_{T,B}^2+(-1+S)\,\alpha_1\,(1-p_{t,B}-p_{T,A}-p_{T,B})\,\big(-r\,p_{T,A}-r\,p_{T,B}\big)+$$

$$\alpha_2\,p_{T,A}\,\big(S+(-1+r)\,(-1+S)\,p_{t,B}+p_{T,A}-S\,p_{T,A}+(-1+r)\,(-1+S)\,(1-p_{t,B}-p_{T,A}-p_{T,B})+p_{T,B}-S\,p_{T,B}\big)\Big)\,w_{T,B}\Big)\Big/$$

$$\Big((-1+S)\,(-1+\alpha_1)\,(1-p_{t,B}-p_{T,A}-p_{T,B})^2+(1-p_{t,B}-p_{T,A}-p_{T,B})\,\Big(S+p_{t,B}-S\,p_{t,B}+\beta_1\,p_{t,B}-S\,\beta_1\,p_{t,B}+p_{T,A}-S\,p_{T,A}-$$

$$r \alpha_2 p_{T,A} + r S \alpha_2 p_{T,A} + p_{T,B} - r p_{T,B} - S p_{T,B} + r S p_{T,B} + r \beta_2 p_{T,B} - r S \beta_2 p_{T,B} +$$
$$\alpha_1 (-S + (-1+S) p_{t,B} - (-1+r)(-1+S) p_{T,A} - p_{T,B} + r p_{T,B} + S p_{T,B} - r S p_{T,B})) +$$
$$p_{t,B} (\beta_1 (S - (-1+S) p_{t,B} + (-1+r)(-1+S) p_{T,A} + p_{T,B} - r p_{T,B} - S p_{T,B} + r S p_{T,B}) + r (-1+S) ((-1+\alpha_2) p_{T,A} - \beta_2 p_{T,B})) +$$
$$((-1+S)(-1+\beta_1) p_{t,B}^2 + \alpha_1 (1 - p_{t,B} - p_{T,A} - p_{T,B}) (S - (-1+S) p_{t,B} + p_{T,A} - r p_{T,A} - S p_{T,A} + r S p_{T,A} - (-1+S)(1 - p_{t,B} - p_{T,A} - p_{T,B}) +$$
$$p_{T,B} - r p_{T,B} - S p_{T,B} + r S p_{T,B}) - r (-1+S)(1 - p_{t,B} - p_{T,A} - p_{T,B}) (\alpha_2 p_{T,A} - (-1+\beta_2) p_{T,B}) +$$
$$p_{t,B} (1 + S - p_{t,B} - r p_{T,A} - S p_{T,A} + r S p_{T,A} + r \alpha_2 p_{T,A} - r S \alpha_2 p_{T,A} - S (1 - p_{t,B} - p_{T,A} - p_{T,B}) - S p_{T,B} - r \beta_2 p_{T,B} +$$
$$r S \beta_2 p_{T,B} + \beta_1 (-S - (-1+r)(-1+S) p_{T,A} + (-1+S)(1 - p_{t,B} - p_{T,A} - p_{T,B}) - p_{T,B} + r p_{T,B} + S p_{T,B} - r S p_{T,B}))) w_{t,B} +$$
$$S p_{T,A} w_{T,A} - S \alpha_2 p_{T,A} w_{T,A} + p_{t,B} p_{T,A} w_{T,A} - r p_{t,B} p_{T,A} w_{T,A} - S p_{t,B} p_{T,A} w_{T,A} + r S p_{t,B} p_{T,A} w_{T,A} - \alpha_2 p_{t,B} p_{T,A} w_{T,A} +$$
$$r \alpha_2 p_{t,B} p_{T,A} w_{T,A} + S \alpha_2 p_{t,B} p_{T,A} w_{T,A} - r S \alpha_2 p_{t,B} p_{T,A} w_{T,A} +$$
$$r \beta_1 p_{t,B} p_{T,A} w_{T,A} - r S \beta_1 p_{t,B} p_{T,A} w_{T,A} + p_{T,A}^2 w_{T,A} - S p_{T,A}^2 w_{T,A} - \alpha_2 p_{T,A}^2 w_{T,A} +$$
$$S \alpha_2 p_{T,A}^2 w_{T,A} + p_{T,A} (1 - p_{t,B} - p_{T,A} - p_{T,B}) w_{T,A} - S p_{T,A} (1 - p_{t,B} - p_{T,A} - p_{T,B}) w_{T,A} -$$
$$r \alpha_1 p_{T,A} (1 - p_{t,B} - p_{T,A} - p_{T,B}) w_{T,A} + r S \alpha_1 p_{T,A} (1 - p_{t,B} - p_{T,A} - p_{T,B}) w_{T,A} -$$
$$\alpha_2 p_{T,A} (1 - p_{t,B} - p_{T,A} - p_{T,B}) w_{T,A} + r \alpha_2 p_{T,A} (1 - p_{t,B} - p_{T,A} - p_{T,B}) w_{T,A} +$$
$$S \alpha_2 p_{T,A} (1 - p_{t,B} - p_{T,A} - p_{T,B}) w_{T,A} - r S \alpha_2 p_{T,A} (1 - p_{t,B} - p_{T,A} - p_{T,B}) w_{T,A} + S \beta_2 p_{T,B} w_{T,A} +$$
$$r \beta_1 p_{t,B} p_{T,B} w_{T,A} - r S \beta_1 p_{t,B} p_{T,B} w_{T,A} + \beta_2 p_{t,B} p_{T,B} w_{T,A} - r \beta_2 p_{t,B} p_{T,B} w_{T,A} -$$
$$S \beta_2 p_{t,B} p_{T,B} w_{T,A} + r S \beta_2 p_{t,B} p_{T,B} w_{T,A} + p_{T,A} p_{T,B} w_{T,A} - S p_{T,A} p_{T,B} w_{T,A} - \alpha_2 p_{T,A} p_{T,B} w_{T,A} +$$
$$S \alpha_2 p_{T,A} p_{T,B} w_{T,A} + \beta_2 p_{T,A} p_{T,B} w_{T,A} - S \beta_2 p_{T,A} p_{T,B} w_{T,A} + r (1 - p_{t,B} - p_{T,A} - p_{T,B}) p_{T,B} w_{T,A} -$$
$$r S (1 - p_{t,B} - p_{T,A} - p_{T,B}) p_{T,B} w_{T,A} - r \alpha_1 (1 - p_{t,B} - p_{T,A} - p_{T,B}) p_{T,B} w_{T,A} +$$
$$r S \alpha_1 (1 - p_{t,B} - p_{T,A} - p_{T,B}) p_{T,B} w_{T,A} + \beta_2 (1 - p_{t,B} - p_{T,A} - p_{T,B}) p_{T,B} w_{T,A} -$$
$$r \beta_2 (1 - p_{t,B} - p_{T,A} - p_{T,B}) p_{T,B} w_{T,A} - S \beta_2 (1 - p_{t,B} - p_{T,A} - p_{T,B}) p_{T,B} w_{T,A} +$$
$$r S \beta_2 (1 - p_{t,B} - p_{T,A} - p_{T,B}) p_{T,B} w_{T,A} + \beta_2 p_{T,B}^2 w_{T,A} - S \beta_2 p_{T,B}^2 w_{T,A} + S \alpha_2 p_{T,A} w_{T,B} +$$
$$r p_{t,B} p_{T,A} w_{T,B} - r S p_{t,B} p_{T,A} w_{T,B} + \alpha_2 p_{t,B} p_{T,A} w_{T,B} - r \alpha_2 p_{t,B} p_{T,A} w_{T,B} - S \alpha_2 p_{t,B} p_{T,A} w_{T,B} +$$
$$r S \alpha_2 p_{t,B} p_{T,A} w_{T,B} - r \beta_1 p_{t,B} p_{T,A} w_{T,B} + r S \beta_1 p_{t,B} p_{T,A} w_{T,B} + \alpha_2 p_{T,A}^2 w_{T,B} -$$
$$S \alpha_2 p_{T,A}^2 w_{T,B} + r \alpha_1 p_{T,A} (1 - p_{t,B} - p_{T,A} - p_{T,B}) w_{T,B} - r S \alpha_1 p_{T,A} (1 - p_{t,B} - p_{T,A} - p_{T,B}) w_{T,B} +$$
$$\alpha_2 p_{T,A} (1 - p_{t,B} - p_{T,A} - p_{T,B}) w_{T,B} - r \alpha_2 p_{T,A} (1 - p_{t,B} - p_{T,A} - p_{T,B}) w_{T,B} - S \alpha_2 p_{T,A} (1 - p_{t,B} - p_{T,A} - p_{T,B}) w_{T,B} +$$
$$r S \alpha_2 p_{T,A} (1 - p_{t,B} - p_{T,A} - p_{T,B}) w_{T,B} + S p_{T,B} w_{T,B} - S \beta_2 p_{T,B} w_{T,B} + p_{t,B} p_{T,B} w_{T,B} -$$
$$S p_{t,B} p_{T,B} w_{T,B} - r \beta_1 p_{t,B} p_{T,B} w_{T,B} + r S \beta_1 p_{t,B} p_{T,B} w_{T,B} - \beta_2 p_{t,B} p_{T,B} w_{T,B} + r \beta_2 p_{t,B} p_{T,B} w_{T,B} +$$
$$S \beta_2 p_{t,B} p_{T,B} w_{T,B} - r S \beta_2 p_{t,B} p_{T,B} w_{T,B} + p_{T,A} p_{T,B} w_{T,B} - S p_{T,A} p_{T,B} w_{T,B} + \alpha_2 p_{T,A} p_{T,B} w_{T,B} -$$
$$S \alpha_2 p_{T,A} p_{T,B} w_{T,B} - \beta_2 p_{T,A} p_{T,B} w_{T,B} + S \beta_2 p_{T,A} p_{T,B} w_{T,B} + (1 - p_{t,B} - p_{T,A} - p_{T,B}) p_{T,B} w_{T,B} -$$
$$r (1 - p_{t,B} - p_{T,A} - p_{T,B}) p_{T,B} w_{T,B} - S (1 - p_{t,B} - p_{T,A} - p_{T,B}) p_{T,B} w_{T,B} + r S (1 - p_{t,B} - p_{T,A} - p_{T,B}) p_{T,B} w_{T,B} +$$
$$r \alpha_1 (1 - p_{t,B} - p_{T,A} - p_{T,B}) p_{T,B} w_{T,B} - r S \alpha_1 (1 - p_{t,B} - p_{T,A} - p_{T,B}) p_{T,B} w_{T,B} -$$
$$\beta_2 (1 - p_{t,B} - p_{T,A} - p_{T,B}) p_{T,B} w_{T,B} + r \beta_2 (1 - p_{t,B} - p_{T,A} - p_{T,B}) p_{T,B} w_{T,B} + S \beta_2 (1 - p_{t,B} - p_{T,A} - p_{T,B}) p_{T,B} w_{T,B} -$$
$$r S \beta_2 (1 - p_{t,B} - p_{T,A} - p_{T,B}) p_{T,B} w_{T,B} + p_{T,B}^2 w_{T,B} - S p_{T,B}^2 w_{T,B} - \beta_2 p_{T,B}^2 w_{T,B} + S \beta_2 p_{T,B}^2 w_{T,B}),$$

$$p_{T,B}' =$$
$$(z ((-1+S)(-1+\beta_1) p_{t,B}^2 + \alpha_1 (1 - p_{t,B} - p_{T,A} - p_{T,B}) (S - (-1+S) p_{t,B} + p_{T,A} - r p_{T,A} - S p_{T,A} + r S p_{T,A} - (-1+S)(1 - p_{t,B} - p_{T,A} - p_{T,B}) + p_{T,B} - r$$
$$p_{T,B} - S p_{T,B} + r S p_{T,B}) - r (-1+S)(1 - p_{t,B} - p_{T,A} - p_{T,B}) (\alpha_2 p_{T,A} - (-1+\beta_2) p_{T,B}) +$$
$$p_{t,B} (1 + S - p_{t,B} - r p_{T,A} - S p_{T,A} + r S p_{T,A} + r \alpha_2 p_{T,A} - r S \alpha_2 p_{T,A} - S (1 - p_{t,B} - p_{T,A} - p_{T,B}) - S p_{T,B} - r \beta_2 p_{T,B} +$$
$$r S \beta_2 p_{T,B} + \beta_1 (-S - (-1+r)(-1+S) p_{T,A} + (-1+S)(1 - p_{t,B} - p_{T,A} - p_{T,B}) - p_{T,B} + r p_{T,B} + S p_{T,B} - r S p_{T,B}))) w_{t,B} -$$
$$(-1+y) (r p_{t,B} p_{T,A} - r S p_{t,B} p_{T,A} - r \beta_1 p_{t,B} p_{T,A} + r S \beta_1 p_{t,B} p_{T,A} + S p_{T,B} - S \beta_2 p_{T,B} + p_{t,B} p_{T,B} - S p_{t,B} p_{T,B} -$$
$$r \beta_1 p_{t,B} p_{T,B} + r S \beta_1 p_{t,B} p_{T,B} - \beta_2 p_{t,B} p_{T,B} + r \beta_2 p_{t,B} p_{T,B} + S \beta_2 p_{t,B} p_{T,B} - r S \beta_2 p_{t,B} p_{T,B} + p_{T,A} p_{T,B} - S p_{T,A} p_{T,B} -$$
$$\beta_2 p_{T,A} p_{T,B} + S \beta_2 p_{T,A} p_{T,B} + (1 - p_{t,B} - p_{T,A} - p_{T,B}) p_{T,B} - r (1 - p_{t,B} - p_{T,A} - p_{T,B}) p_{T,B} - S (1 - p_{t,B} - p_{T,A} - p_{T,B}) p_{T,B} +$$
$$r S (1 - p_{t,B} - p_{T,A} - p_{T,B}) p_{T,B} - \beta_2 (1 - p_{t,B} - p_{T,A} - p_{T,B}) p_{T,B} + r \beta_2 (1 - p_{t,B} - p_{T,A} - p_{T,B}) p_{T,B} + S \beta_2 (1 - p_{t,B} - p_{T,A} - p_{T,B}) p_{T,B} -$$
$$r S \beta_2 (1 - p_{t,B} - p_{T,A} - p_{T,B}) p_{T,B} + p_{T,B}^2 - S p_{T,B}^2 - \beta_2 p_{T,B}^2 + S \beta_2 p_{T,B}^2 + (-1+S) \alpha_1 (1 - p_{t,B} - p_{T,A} - p_{T,B}) (-r p_{T,A} - r p_{T,B}) +$$
$$\alpha_2 p_{T,A} (S + (-1+r)(-1+S) p_{t,B} + p_{T,A} - S p_{T,A} + (-1+r)(-1+S)(1 - p_{t,B} - p_{T,A} - p_{T,B}) + p_{T,B} - S p_{T,B})) w_{T,B}) /$$

$$\Big((-1+S)\,(-1+\alpha_1)\,(1-p_{t,B}-p_{T,A}-p_{T,B})^2+(1-p_{t,B}-p_{T,A}-p_{T,B})\,\big(S+p_{t,B}-S\,p_{t,B}+\beta_1\,p_{t,B}-S\,\beta_1\,p_{t,B}+p_{T,A}-S\,p_{T,A}-$$
$$r\,\alpha_2\,p_{T,A}+r\,S\,\alpha_2\,p_{T,A}+p_{T,B}-r\,p_{T,B}-S\,p_{T,B}+r\,S\,p_{T,B}+r\,\beta_2\,p_{T,B}-r\,S\,\beta_2\,p_{T,B}+$$
$$\alpha_1\,\big(-S+(-1+S)\,p_{t,B}-(-1+r)\,(-1+S)\,p_{T,A}-p_{T,B}+r\,p_{T,B}+S\,p_{T,B}-r\,S\,p_{T,B}\big)\big)+$$
$$p_{t,B}\,\big(\beta_1\,\big(S-(-1+S)\,p_{t,B}+(-1+r)\,(-1+S)\,p_{T,A}+p_{T,B}-r\,p_{T,B}-S\,p_{T,B}+r\,S\,p_{T,B}\big)+r\,(-1+S)\,\big((-1+\alpha_2)\,p_{T,A}-\beta_2\,p_{T,B}\big)\big)+$$
$$\Big((-1+S)\,(-1+\beta_1)\,p_{t,B}^2+\alpha_1\,(1-p_{t,B}-p_{T,A}-p_{T,B})\,\big(S-(-1+S)\,p_{t,B}+p_{T,A}-r\,p_{T,A}-S\,p_{T,A}+r\,S\,p_{T,A}-(-1+S)\,(1-p_{t,B}-p_{T,A}-p_{T,B})+$$
$$p_{T,B}-r\,p_{T,B}-S\,p_{T,B}+r\,S\,p_{T,B}\big)-r\,(-1+S)\,(1-p_{t,B}-p_{T,A}-p_{T,B})\,\big(\alpha_2\,p_{T,A}-(-1+\beta_2)\,p_{T,B}\big)+$$
$$p_{t,B}\,\big(1+S-p_{t,B}-r\,p_{T,A}-S\,p_{T,A}+r\,S\,p_{T,A}+r\,\alpha_2\,p_{T,A}-r\,S\,\alpha_2\,p_{T,A}-S\,(1-p_{t,B}-p_{T,A}-p_{T,B})-S\,p_{T,B}-r\,\beta_2\,p_{T,B}+$$
$$r\,S\,\beta_2\,p_{T,B}+\beta_1\,\big(-S-(-1+r)\,(-1+S)\,p_{T,A}+(-1+S)\,(1-p_{t,B}-p_{T,A}-p_{T,B})-p_{T,B}+r\,p_{T,B}+S\,p_{T,B}-r\,S\,p_{T,B}\big)\big)\Big)\,w_{t,B}+$$
$$S\,p_{T,A}\,w_{T,A}-S\,\alpha_2\,p_{T,A}\,w_{T,A}+p_{t,B}\,p_{T,A}\,w_{T,A}-r\,p_{t,B}\,p_{T,A}\,w_{T,A}-S\,p_{t,B}\,p_{T,A}\,w_{T,A}+r\,S\,p_{t,B}\,p_{T,A}\,w_{T,A}-\alpha_2\,p_{t,B}\,p_{T,A}\,w_{T,A}+$$
$$r\,\alpha_2\,p_{t,B}\,p_{T,A}\,w_{T,A}+S\,\alpha_2\,p_{t,B}\,p_{T,A}\,w_{T,A}-r\,S\,\alpha_2\,p_{t,B}\,p_{T,A}\,w_{T,A}+$$
$$r\,\beta_1\,p_{t,B}\,p_{T,A}\,w_{T,A}-r\,S\,\beta_1\,p_{t,B}\,p_{T,A}\,w_{T,A}+p_{T,A}^2\,w_{T,A}-S\,p_{T,A}^2\,w_{T,A}-$$
$$\alpha_2\,p_{T,A}^2\,w_{T,A}+S\,\alpha_2\,p_{T,A}^2\,w_{T,A}+p_{T,A}\,(1-p_{t,B}-p_{T,A}-p_{T,B})\,w_{T,A}-$$
$$S\,p_{T,A}\,(1-p_{t,B}-p_{T,A}-p_{T,B})\,w_{T,A}-r\,\alpha_1\,p_{T,A}\,(1-p_{t,B}-p_{T,A}-p_{T,B})\,w_{T,A}+$$
$$r\,S\,\alpha_1\,p_{T,A}\,(1-p_{t,B}-p_{T,A}-p_{T,B})\,w_{T,A}-\alpha_2\,p_{T,A}\,(1-p_{t,B}-p_{T,A}-p_{T,B})\,w_{T,A}+$$
$$r\,\alpha_2\,p_{T,A}\,(1-p_{t,B}-p_{T,A}-p_{T,B})\,w_{T,A}+S\,\alpha_2\,p_{T,A}\,(1-p_{t,B}-p_{T,A}-p_{T,B})\,w_{T,A}-$$
$$r\,S\,\alpha_2\,p_{T,A}\,(1-p_{t,B}-p_{T,A}-p_{T,B})\,w_{T,A}+S\,\beta_2\,p_{T,B}\,w_{T,A}+r\,\beta_1\,p_{t,B}\,p_{T,B}\,w_{T,A}-r\,S\,\beta_1\,p_{t,B}\,p_{T,B}\,w_{T,A}+$$
$$\beta_2\,p_{t,B}\,p_{T,B}\,w_{T,A}-r\,\beta_2\,p_{t,B}\,p_{T,B}\,w_{T,A}-S\,\beta_2\,p_{t,B}\,p_{T,B}\,w_{T,A}+r\,S\,\beta_2\,p_{t,B}\,p_{T,B}\,w_{T,A}+$$
$$p_{T,A}\,p_{T,B}\,w_{T,A}-S\,p_{T,A}\,p_{T,B}\,w_{T,A}-\alpha_2\,p_{T,A}\,p_{T,B}\,w_{T,A}+S\,\alpha_2\,p_{T,A}\,p_{T,B}\,w_{T,A}+\beta_2\,p_{T,A}\,p_{T,B}\,w_{T,A}-$$
$$S\,\beta_2\,p_{T,A}\,p_{T,B}\,w_{T,A}+r\,(1-p_{t,B}-p_{T,A}-p_{T,B})\,p_{T,B}\,w_{T,A}-r\,S\,(1-p_{t,B}-p_{T,A}-p_{T,B})\,p_{T,B}\,w_{T,A}-$$
$$r\,\alpha_1\,(1-p_{t,B}-p_{T,A}-p_{T,B})\,p_{T,B}\,w_{T,A}+r\,S\,\alpha_1\,(1-p_{t,B}-p_{T,A}-p_{T,B})\,p_{T,B}\,w_{T,A}+$$
$$\beta_2\,(1-p_{t,B}-p_{T,A}-p_{T,B})\,p_{T,B}\,w_{T,A}-r\,\beta_2\,(1-p_{t,B}-p_{T,A}-p_{T,B})\,p_{T,B}\,w_{T,A}-$$
$$S\,\beta_2\,(1-p_{t,B}-p_{T,A}-p_{T,B})\,p_{T,B}\,w_{T,A}+r\,S\,\beta_2\,(1-p_{t,B}-p_{T,A}-p_{T,B})\,p_{T,B}\,w_{T,A}+\beta_2\,p_{T,B}^2\,w_{T,A}-$$
$$S\,\beta_2\,p_{T,B}^2\,w_{T,A}+S\,\alpha_2\,p_{T,A}\,w_{T,B}+r\,p_{t,B}\,p_{T,A}\,w_{T,B}-r\,S\,p_{t,B}\,p_{T,A}\,w_{T,B}+\alpha_2\,p_{t,B}\,p_{T,A}\,w_{T,B}-$$
$$r\,\alpha_2\,p_{t,B}\,p_{T,A}\,w_{T,B}-S\,\alpha_2\,p_{t,B}\,p_{T,A}\,w_{T,B}+r\,S\,\alpha_2\,p_{t,B}\,p_{T,A}\,w_{T,B}-r\,\beta_1\,p_{t,B}\,p_{T,A}\,w_{T,B}+$$
$$r\,S\,\beta_1\,p_{t,B}\,p_{T,A}\,w_{T,B}+\alpha_2\,p_{T,A}^2\,w_{T,B}-S\,\alpha_2\,p_{T,A}^2\,w_{T,B}+r\,\alpha_1\,p_{T,A}\,(1-p_{t,B}-p_{T,A}-p_{T,B})\,w_{T,B}-$$
$$r\,S\,\alpha_1\,p_{T,A}\,(1-p_{t,B}-p_{T,A}-p_{T,B})\,w_{T,B}+\alpha_2\,p_{T,A}\,(1-p_{t,B}-p_{T,A}-p_{T,B})\,w_{T,B}-r\,\alpha_2\,p_{T,A}\,(1-p_{t,B}-p_{T,A}-p_{T,B})\,w_{T,B}-$$
$$S\,\alpha_2\,p_{T,A}\,(1-p_{t,B}-p_{T,A}-p_{T,B})\,w_{T,B}+r\,S\,\alpha_2\,p_{T,A}\,(1-p_{t,B}-p_{T,A}-p_{T,B})\,w_{T,B}+S\,p_{T,B}\,w_{T,B}-$$
$$S\,\beta_2\,p_{T,B}\,w_{T,B}+p_{t,B}\,p_{T,B}\,w_{T,B}-S\,p_{t,B}\,p_{T,B}\,w_{T,B}-r\,\beta_1\,p_{t,B}\,p_{T,B}\,w_{T,B}+r\,S\,\beta_1\,p_{t,B}\,p_{T,B}\,w_{T,B}-$$
$$\beta_2\,p_{t,B}\,p_{T,B}\,w_{T,B}+r\,\beta_2\,p_{t,B}\,p_{T,B}\,w_{T,B}+S\,\beta_2\,p_{t,B}\,p_{T,B}\,w_{T,B}-r\,S\,\beta_2\,p_{t,B}\,p_{T,B}\,w_{T,B}+p_{T,A}\,p_{T,B}\,w_{T,B}-$$
$$S\,p_{T,A}\,p_{T,B}\,w_{T,B}+\alpha_2\,p_{T,A}\,p_{T,B}\,w_{T,B}-S\,\alpha_2\,p_{T,A}\,p_{T,B}\,w_{T,B}-\beta_2\,p_{T,A}\,p_{T,B}\,w_{T,B}+S\,\beta_2\,p_{T,A}\,p_{T,B}\,w_{T,B}+$$
$$(1-p_{t,B}-p_{T,A}-p_{T,B})\,p_{T,B}\,w_{T,B}-r\,(1-p_{t,B}-p_{T,A}-p_{T,B})\,p_{T,B}\,w_{T,B}-S\,(1-p_{t,B}-p_{T,A}-p_{T,B})\,p_{T,B}\,w_{T,B}+$$
$$r\,S\,(1-p_{t,B}-p_{T,A}-p_{T,B})\,p_{T,B}\,w_{T,B}+r\,\alpha_1\,(1-p_{t,B}-p_{T,A}-p_{T,B})\,p_{T,B}\,w_{T,B}-r\,S\,\alpha_1\,(1-p_{t,B}-p_{T,A}-p_{T,B})\,p_{T,B}\,w_{T,B}-$$
$$\beta_2\,(1-p_{t,B}-p_{T,A}-p_{T,B})\,p_{T,B}\,w_{T,B}+r\,\beta_2\,(1-p_{t,B}-p_{T,A}-p_{T,B})\,p_{T,B}\,w_{T,B}+S\,\beta_2\,(1-p_{t,B}-p_{T,A}-p_{T,B})\,p_{T,B}\,w_{T,B}-$$
$$r\,S\,\beta_2\,(1-p_{t,B}-p_{T,A}-p_{T,B})\,p_{T,B}\,w_{T,B}+p_{T,B}^2\,w_{T,B}-S\,p_{T,B}^2\,w_{T,B}-\beta_2\,p_{T,B}^2\,w_{T,B}+S\,\beta_2\,p_{T,B}^2\,w_{T,B}\Big)$$

B2 - Calculating Equilibria

In[∘]:=

$$\{0 == -p_{t,B}+$$
$$\Big((-1+z)\,\Big(-\big((-1+S)\,(-1+\beta_1)\,p_{t,B}^2\big)+\alpha_1\,(1-p_{t,B}-p_{T,A}-p_{T,B})\,\big(-S+(-1+S)\,p_{t,B}-p_{T,A}+r\,p_{T,A}+S\,p_{T,A}-$$
$$r\,S\,p_{T,A}+(-1+S)\,(1-p_{t,B}-p_{T,A}-p_{T,B})-p_{T,B}+r\,p_{T,B}+S\,p_{T,B}-r\,S\,p_{T,B}\big)+$$
$$r\,(-1+S)\,(1-p_{t,B}-p_{T,A}-p_{T,B})\,\big(\alpha_2\,p_{T,A}-(-1+\beta_2)\,p_{T,B}\big)-$$
$$p_{t,B}\,\big(1+S-p_{t,B}-r\,p_{T,A}-S\,p_{T,A}+r\,S\,p_{T,A}+r\,\alpha_2\,p_{T,A}-r\,S\,\alpha_2\,p_{T,A}-$$
$$S\,(1-p_{t,B}-p_{T,A}-p_{T,B})-S\,p_{T,B}-r\,\beta_2\,p_{T,B}+r\,S\,\beta_2\,p_{T,B}+\beta_1\,\big(-S-(-1+r)\,(-1+S)\,p_{T,A}+$$
$$(-1+S)\,(1-p_{t,B}-p_{T,A}-p_{T,B})-p_{T,B}+r\,p_{T,B}+S\,p_{T,B}-r\,S\,p_{T,B}\big)\big)\Big)\,w_{t,B}+$$

$$y\left(r\,p_{t,B}\,p_{T,A}-r\,S\,p_{t,B}\,p_{T,A}-r\,\beta_1\,p_{t,B}\,p_{T,A}+r\,S\,\beta_1\,p_{t,B}\,p_{T,A}+S\,p_{T,B}-S\,\beta_2\,p_{T,B}+\right.$$
$$p_{t,B}\,p_{T,B}-S\,p_{t,B}\,p_{T,B}-r\,\beta_1\,p_{t,B}\,p_{T,B}+r\,S\,\beta_1\,p_{t,B}\,p_{T,B}-\beta_2\,p_{t,B}\,p_{T,B}+r\,\beta_2\,p_{t,B}\,p_{T,B}+$$
$$S\,\beta_2\,p_{t,B}\,p_{T,B}-r\,S\,\beta_2\,p_{t,B}\,p_{T,B}+p_{T,A}\,p_{T,B}-S\,p_{T,A}\,p_{T,B}-\beta_2\,p_{T,A}\,p_{T,B}+S\,\beta_2\,p_{T,A}\,p_{T,B}+$$
$$(1-p_{t,B}-p_{T,A}-p_{T,B})\,p_{T,B}-r\,(1-p_{t,B}-p_{T,A}-p_{T,B})\,p_{T,B}-S\,(1-p_{t,B}-p_{T,A}-p_{T,B})\,p_{T,B}+$$
$$r\,S\,(1-p_{t,B}-p_{T,A}-p_{T,B})\,p_{T,B}-\beta_2\,(1-p_{t,B}-p_{T,A}-p_{T,B})\,p_{T,B}+r\,\beta_2\,(1-p_{t,B}-p_{T,A}-p_{T,B})\,p_{T,B}+$$
$$S\,\beta_2\,(1-p_{t,B}-p_{T,A}-p_{T,B})\,p_{T,B}-r\,S\,\beta_2\,(1-p_{t,B}-p_{T,A}-p_{T,B})\,p_{T,B}+p_{T,B}^2-S\,p_{T,B}^2-\beta_2\,p_{T,B}^2+$$
$$S\,\beta_2\,p_{T,B}^2+(-1+S)\,\alpha_1\,(1-p_{t,B}-p_{T,A}-p_{T,B})\,(-r\,p_{T,A}-r\,p_{T,B})+\alpha_2\,p_{T,A}\,\big(S+(-1+r)\,(-1+S)\,p_{t,B}+$$
$$\left.p_{T,A}-S\,p_{T,A}+(-1+r)\,(-1+S)\,(1-p_{t,B}-p_{T,A}-p_{T,B})+p_{T,B}-S\,p_{T,B}\big)\big)\,w_{T,B}\right)\Big/$$
$$\Big((-1+S)\,(-1+\alpha_1)\,(1-p_{t,B}-p_{T,A}-p_{T,B})^2+(1-p_{t,B}-p_{T,A}-p_{T,B})\,\big(S+p_{t,B}-S\,p_{t,B}+\beta_1\,p_{t,B}-$$
$$S\,\beta_1\,p_{t,B}+p_{T,A}-S\,p_{T,A}-r\,\alpha_2\,p_{T,A}+r\,S\,\alpha_2\,p_{T,A}+p_{T,B}-r\,p_{T,B}-S\,p_{T,B}+r\,S\,p_{T,B}+r\,\beta_2\,p_{T,B}-$$
$$r\,S\,\beta_2\,p_{T,B}+\alpha_1\,(-S+(-1+S)\,p_{t,B}-(-1+r)\,(-1+S)\,p_{T,A}-p_{T,B}+r\,p_{T,B}+S\,p_{T,B}-r\,S\,p_{T,B})\big)+$$
$$p_{t,B}\,\big(\beta_1\,(S-(-1+S)\,p_{t,B}+(-1+r)\,(-1+S)\,p_{T,A}+p_{T,B}-r\,p_{T,B}-S\,p_{T,B}+r\,S\,p_{T,B})+$$
$$r\,(-1+S)\,((-1+\alpha_2)\,p_{T,A}-\beta_2\,p_{T,B})\big)+$$
$$\big((-1+S)\,(-1+\beta_1)\,p_{t,B}^2+\alpha_1\,(1-p_{t,B}-p_{T,A}-p_{T,B})\,(S-(-1+S)\,p_{t,B}+p_{T,A}-r\,p_{T,A}-S\,p_{T,A}+$$
$$r\,S\,p_{T,A}-(-1+S)\,(1-p_{t,B}-p_{T,A}-p_{T,B})+p_{T,B}-r\,p_{T,B}-S\,p_{T,B}+r\,S\,p_{T,B})-$$
$$r\,(-1+S)\,(1-p_{t,B}-p_{T,A}-p_{T,B})\,(\alpha_2\,p_{T,A}-(-1+\beta_2)\,p_{T,B})+$$
$$p_{t,B}\,\big(1+S-p_{t,B}-r\,p_{T,A}-S\,p_{T,A}+r\,S\,p_{T,A}+r\,\alpha_2\,p_{T,A}-r\,S\,\alpha_2\,p_{T,A}-$$
$$S\,(1-p_{t,B}-p_{T,A}-p_{T,B})-S\,p_{T,B}-r\,\beta_2\,p_{T,B}+r\,S\,\beta_2\,p_{T,B}+\beta_1\,(-S-(-1+r)\,(-1+S)\,p_{T,A}+$$
$$(-1+S)\,(1-p_{t,B}-p_{T,A}-p_{T,B})-p_{T,B}+r\,p_{T,B}+S\,p_{T,B}-r\,S\,p_{T,B})\big)\big)\,w_{t,B}+$$
$$S\,p_{T,A}\,w_{T,A}-S\,\alpha_2\,p_{T,A}\,w_{T,A}+p_{t,B}\,p_{T,A}\,w_{T,A}-r\,p_{t,B}\,p_{T,A}\,w_{T,A}-S\,p_{t,B}\,p_{T,A}\,w_{T,A}+$$
$$r\,S\,p_{t,B}\,p_{T,A}\,w_{T,A}-\alpha_2\,p_{t,B}\,p_{T,A}\,w_{T,A}+r\,\alpha_2\,p_{t,B}\,p_{T,A}\,w_{T,A}+$$
$$S\,\alpha_2\,p_{t,B}\,p_{T,A}\,w_{T,A}-r\,S\,\alpha_2\,p_{t,B}\,p_{T,A}\,w_{T,A}+r\,\beta_1\,p_{t,B}\,p_{T,A}\,w_{T,A}-$$
$$r\,S\,\beta_1\,p_{t,B}\,p_{T,A}\,w_{T,A}+p_{T,A}^2\,w_{T,A}-S\,p_{T,A}^2\,w_{T,A}-\alpha_2\,p_{T,A}^2\,w_{T,A}+S\,\alpha_2\,p_{T,A}^2\,w_{T,A}+$$
$$p_{T,A}\,(1-p_{t,B}-p_{T,A}-p_{T,B})\,w_{T,A}-S\,p_{T,A}\,(1-p_{t,B}-p_{T,A}-p_{T,B})\,w_{T,A}-$$
$$r\,\alpha_1\,p_{T,A}\,(1-p_{t,B}-p_{T,A}-p_{T,B})\,w_{T,A}+r\,S\,\alpha_1\,p_{T,A}\,(1-p_{t,B}-p_{T,A}-p_{T,B})\,w_{T,A}-$$
$$\alpha_2\,p_{T,A}\,(1-p_{t,B}-p_{T,A}-p_{T,B})\,w_{T,A}+r\,\alpha_2\,p_{T,A}\,(1-p_{t,B}-p_{T,A}-p_{T,B})\,w_{T,A}+$$
$$S\,\alpha_2\,p_{T,A}\,(1-p_{t,B}-p_{T,A}-p_{T,B})\,w_{T,A}-r\,S\,\alpha_2\,p_{T,A}\,(1-p_{t,B}-p_{T,A}-p_{T,B})\,w_{T,A}+$$
$$S\,\beta_2\,p_{T,B}\,w_{T,A}+r\,\beta_1\,p_{t,B}\,p_{T,B}\,w_{T,A}-r\,S\,\beta_1\,p_{t,B}\,p_{T,B}\,w_{T,A}+\beta_2\,p_{t,B}\,p_{T,B}\,w_{T,A}-$$
$$r\,\beta_2\,p_{t,B}\,p_{T,B}\,w_{T,A}-S\,\beta_2\,p_{t,B}\,p_{T,B}\,w_{T,A}+r\,S\,\beta_2\,p_{t,B}\,p_{T,B}\,w_{T,A}+$$
$$p_{T,A}\,p_{T,B}\,w_{T,A}-S\,p_{T,A}\,p_{T,B}\,w_{T,A}-\alpha_2\,p_{T,A}\,p_{T,B}\,w_{T,A}+S\,\alpha_2\,p_{T,A}\,p_{T,B}\,w_{T,A}+$$
$$\beta_2\,p_{T,A}\,p_{T,B}\,w_{T,A}-S\,\beta_2\,p_{T,A}\,p_{T,B}\,w_{T,A}+r\,(1-p_{t,B}-p_{T,A}-p_{T,B})\,p_{T,B}\,w_{T,A}-$$
$$r\,S\,(1-p_{t,B}-p_{T,A}-p_{T,B})\,p_{T,B}\,w_{T,A}-r\,\alpha_1\,(1-p_{t,B}-p_{T,A}-p_{T,B})\,p_{T,B}\,w_{T,A}+$$
$$r\,S\,\alpha_1\,(1-p_{t,B}-p_{T,A}-p_{T,B})\,p_{T,B}\,w_{T,A}+\beta_2\,(1-p_{t,B}-p_{T,A}-p_{T,B})\,p_{T,B}\,w_{T,A}-$$
$$r\,\beta_2\,(1-p_{t,B}-p_{T,A}-p_{T,B})\,p_{T,B}\,w_{T,A}-S\,\beta_2\,(1-p_{t,B}-p_{T,A}-p_{T,B})\,p_{T,B}\,w_{T,A}+$$
$$r\,S\,\beta_2\,(1-p_{t,B}-p_{T,A}-p_{T,B})\,p_{T,B}\,w_{T,A}+\beta_2\,p_{T,B}^2\,w_{T,A}-S\,\beta_2\,p_{T,B}^2\,w_{T,A}+S\,\alpha_2\,p_{T,A}\,w_{T,B}+$$
$$r\,p_{t,B}\,p_{T,A}\,w_{T,B}-r\,S\,p_{t,B}\,p_{T,A}\,w_{T,B}+\alpha_2\,p_{t,B}\,p_{T,A}\,w_{T,B}-r\,\alpha_2\,p_{t,B}\,p_{T,A}\,w_{T,B}-$$
$$S\,\alpha_2\,p_{t,B}\,p_{T,A}\,w_{T,B}+r\,S\,\alpha_2\,p_{t,B}\,p_{T,A}\,w_{T,B}-r\,\beta_1\,p_{t,B}\,p_{T,A}\,w_{T,B}+r\,S\,\beta_1\,p_{t,B}\,p_{T,A}\,w_{T,B}+$$
$$\alpha_2\,p_{T,A}^2\,w_{T,B}-S\,\alpha_2\,p_{T,A}^2\,w_{T,B}+r\,\alpha_1\,p_{T,A}\,(1-p_{t,B}-p_{T,A}-p_{T,B})\,w_{T,B}-$$
$$r\,S\,\alpha_1\,p_{T,A}\,(1-p_{t,B}-p_{T,A}-p_{T,B})\,w_{T,B}+\alpha_2\,p_{T,A}\,(1-p_{t,B}-p_{T,A}-p_{T,B})\,w_{T,B}-$$
$$r\,\alpha_2\,p_{T,A}\,(1-p_{t,B}-p_{T,A}-p_{T,B})\,w_{T,B}-S\,\alpha_2\,p_{T,A}\,(1-p_{t,B}-p_{T,A}-p_{T,B})\,w_{T,B}+$$
$$r\,S\,\alpha_2\,p_{T,A}\,(1-p_{t,B}-p_{T,A}-p_{T,B})\,w_{T,B}+S\,p_{T,B}\,w_{T,B}-S\,\beta_2\,p_{T,B}\,w_{T,B}+$$

$$p_{t,B}\,p_{T,B}\,w_{T,B}-S\,p_{t,B}\,p_{T,B}\,w_{T,B}-r\,\beta_1\,p_{t,B}\,p_{T,B}\,w_{T,B}+r\,S\,\beta_1\,p_{t,B}\,p_{T,B}\,w_{T,B}-$$
$$\beta_2\,p_{t,B}\,p_{T,B}\,w_{T,B}+r\,\beta_2\,p_{t,B}\,p_{T,B}\,w_{T,B}+S\,\beta_2\,p_{t,B}\,p_{T,B}\,w_{T,B}-r\,S\,\beta_2\,p_{t,B}\,p_{T,B}\,w_{T,B}+$$
$$p_{T,A}\,p_{T,B}\,w_{T,B}-S\,p_{T,A}\,p_{T,B}\,w_{T,B}+\alpha_2\,p_{T,A}\,p_{T,B}\,w_{T,B}-S\,\alpha_2\,p_{T,A}\,p_{T,B}\,w_{T,B}-$$
$$\beta_2\,p_{T,A}\,p_{T,B}\,w_{T,B}+S\,\beta_2\,p_{T,A}\,p_{T,B}\,w_{T,B}+(1-p_{t,B}-p_{T,A}-p_{T,B})\,p_{T,B}\,w_{T,B}-$$
$$r\,(1-p_{t,B}-p_{T,A}-p_{T,B})\,p_{T,B}\,w_{T,B}-S\,(1-p_{t,B}-p_{T,A}-p_{T,B})\,p_{T,B}\,w_{T,B}+$$
$$r\,S\,(1-p_{t,B}-p_{T,A}-p_{T,B})\,p_{T,B}\,w_{T,B}+r\,\alpha_1\,(1-p_{t,B}-p_{T,A}-p_{T,B})\,p_{T,B}\,w_{T,B}-$$
$$r\,S\,\alpha_1\,(1-p_{t,B}-p_{T,A}-p_{T,B})\,p_{T,B}\,w_{T,B}-\beta_2\,(1-p_{t,B}-p_{T,A}-p_{T,B})\,p_{T,B}\,w_{T,B}+$$
$$r\,\beta_2\,(1-p_{t,B}-p_{T,A}-p_{T,B})\,p_{T,B}\,w_{T,B}+S\,\beta_2\,(1-p_{t,B}-p_{T,A}-p_{T,B})\,p_{T,B}\,w_{T,B}-$$
$$r\,S\,\beta_2\,(1-p_{t,B}-p_{T,A}-p_{T,B})\,p_{T,B}\,w_{T,B}+p_{T,B}^2\,w_{T,B}-S\,p_{T,B}^2\,w_{T,B}-\beta_2\,p_{T,B}^2\,w_{T,B}+S\,\beta_2\,p_{T,B}^2\,w_{T,B}\big),$$
$$0==-p_{T,A}+\Big(z\,\big((-1+S)\,(-1+\alpha_1)\,(1-p_{t,B}-p_{T,A}-p_{T,B})^2+(1-p_{t,B}-p_{T,A}-p_{T,B})$$
$$\big(S+p_{t,B}-S\,p_{t,B}+\beta_1\,p_{t,B}-S\,\beta_1\,p_{t,B}+p_{T,A}-S\,p_{T,A}-r\,\alpha_2\,p_{T,A}+r\,S\,\alpha_2\,p_{T,A}+p_{T,B}-r\,p_{T,B}-$$
$$S\,p_{T,B}+r\,S\,p_{T,B}+r\,\beta_2\,p_{T,B}-r\,S\,\beta_2\,p_{T,B}+\alpha_1\,\big(-S+(-1+S)\,p_{t,B}-(-1+r)\,(-1+S)\,p_{T,A}-$$
$$p_{T,B}+r\,p_{T,B}+S\,p_{T,B}-r\,S\,p_{T,B}\big)\big)+p_{t,B}\,\big(\beta_1\,\big(S-(-1+S)\,p_{t,B}+(-1+r)\,(-1+S)\,p_{T,A}+$$
$$p_{T,B}-r\,p_{T,B}-S\,p_{T,B}+r\,S\,p_{T,B}\big)+r\,(-1+S)\,\big((-1+\alpha_2)\,p_{T,A}-\beta_2\,p_{T,B}\big)\big)\big)+$$
$$(-1+y)\,\big(-\big((-1+S)\,(-1+\alpha_2)\,p_{T,A}^2\big)+p_{T,B}\,\big(-r\,(-1+S)\,\big(-\beta_1\,p_{t,B}+(-1+\alpha_1)\,(1-p_{t,B}-p_{T,A}-p_{T,B})\big)+$$
$$\beta_2\,\big(-S-(-1+r)\,(-1+S)\,p_{t,B}-(-1+r)\,(-1+S)\,(1-p_{t,B}-p_{T,A}-p_{T,B})+(-1+S)\,p_{T,B}\big)\big)-$$
$$p_{T,A}\,\big(1+S-r\,p_{t,B}-S\,p_{t,B}+r\,S\,p_{t,B}+r\,\beta_1\,p_{t,B}-r\,S\,\beta_1\,p_{t,B}-p_{T,A}-S\,(1-p_{t,B}-p_{T,A}-p_{T,B})-$$
$$r\,\alpha_1\,(1-p_{t,B}-p_{T,A}-p_{T,B})+r\,S\,\alpha_1\,(1-p_{t,B}-p_{T,A}-p_{T,B})-S\,p_{T,B}+\beta_2\,p_{T,B}-S\,\beta_2\,p_{T,B}+$$
$$\alpha_2\,\big(-S-(-1+r)\,(-1+S)\,p_{t,B}-(-1+r)\,(-1+S)\,(1-p_{t,B}-p_{T,A}-p_{T,B})-p_{T,B}+S\,p_{T,B}\big)\big)\big)\,w_{T,A}\Big)\,/$$
$$\big((-1+S)\,(-1+\alpha_1)\,(1-p_{t,B}-p_{T,A}-p_{T,B})^2+(1-p_{t,B}-p_{T,A}-p_{T,B})\,\big(S+p_{t,B}-S\,p_{t,B}+\beta_1\,p_{t,B}-$$
$$S\,\beta_1\,p_{t,B}+p_{T,A}-S\,p_{T,A}-r\,\alpha_2\,p_{T,A}+r\,S\,\alpha_2\,p_{T,A}+p_{T,B}-r\,p_{T,B}-S\,p_{T,B}+r\,S\,p_{T,B}+r\,\beta_2\,p_{T,B}-$$
$$r\,S\,\beta_2\,p_{T,B}+\alpha_1\,\big(-S+(-1+S)\,p_{t,B}-(-1+r)\,(-1+S)\,p_{T,A}-p_{T,B}+r\,p_{T,B}+S\,p_{T,B}-r\,S\,p_{T,B}\big)\big)+$$
$$p_{t,B}\,\big(\beta_1\,\big(S-(-1+S)\,p_{t,B}+(-1+r)\,(-1+S)\,p_{T,A}+p_{T,B}-r\,p_{T,B}-S\,p_{T,B}+r\,S\,p_{T,B}\big)+$$
$$r\,(-1+S)\,\big((-1+\alpha_2)\,p_{T,A}-\beta_2\,p_{T,B}\big)\big)+$$
$$\big((-1+S)\,(-1+\beta_1)\,p_{t,B}^2+\alpha_1\,(1-p_{t,B}-p_{T,A}-p_{T,B})\,\big(S-(-1+S)\,p_{t,B}+p_{T,A}-r\,p_{T,A}-S\,p_{T,A}+$$
$$r\,S\,p_{T,A}-(-1+S)\,(1-p_{t,B}-p_{T,A}-p_{T,B})+p_{T,B}-r\,p_{T,B}-S\,p_{T,B}+r\,S\,p_{T,B}\big)-r\,(-1+S)$$
$$(1-p_{t,B}-p_{T,A}-p_{T,B})\,\big(\alpha_2\,p_{T,A}-(-1+\beta_2)\,p_{T,B}\big)+p_{t,B}\,\big(1+S-p_{t,B}-r\,p_{T,A}-S\,p_{T,A}+r\,S\,p_{T,A}+$$
$$r\,\alpha_2\,p_{T,A}-r\,S\,\alpha_2\,p_{T,A}-S\,(1-p_{t,B}-p_{T,A}-p_{T,B})-S\,p_{T,B}-r\,\beta_2\,p_{T,B}+r\,S\,\beta_2\,p_{T,B}+\beta_1\,\big(-S-$$
$$(-1+r)\,(-1+S)\,p_{T,A}+(-1+S)\,(1-p_{t,B}-p_{T,A}-p_{T,B})-p_{T,B}+r\,p_{T,B}+S\,p_{T,B}-r\,S\,p_{T,B}\big)\big)\big)$$
$$w_{t,B}+S\,p_{T,A}\,w_{T,A}-S\,\alpha_2\,p_{T,A}\,w_{T,A}+p_{t,B}\,p_{T,A}\,w_{T,A}-r\,p_{t,B}\,p_{T,A}\,w_{T,A}-S\,p_{t,B}\,p_{T,A}\,w_{T,A}+$$
$$r\,S\,p_{t,B}\,p_{T,A}\,w_{T,A}-\alpha_2\,p_{t,B}\,p_{T,A}\,w_{T,A}+r\,\alpha_2\,p_{t,B}\,p_{T,A}\,w_{T,A}+S\,\alpha_2\,p_{t,B}\,p_{T,A}\,w_{T,A}-$$
$$r\,S\,\alpha_2\,p_{t,B}\,p_{T,A}\,w_{T,A}+r\,\beta_1\,p_{t,B}\,p_{T,A}\,w_{T,A}-r\,S\,\beta_1\,p_{t,B}\,p_{T,A}\,w_{T,A}+p_{T,A}^2\,w_{T,A}-$$
$$S\,p_{T,A}^2\,w_{T,A}-\alpha_2\,p_{T,A}^2\,w_{T,A}+S\,\alpha_2\,p_{T,A}^2\,w_{T,A}+p_{T,A}\,(1-p_{t,B}-p_{T,A}-p_{T,B})\,w_{T,A}-$$
$$S\,p_{T,A}\,(1-p_{t,B}-p_{T,A}-p_{T,B})\,w_{T,A}-r\,\alpha_1\,p_{T,A}\,(1-p_{t,B}-p_{T,A}-p_{T,B})\,w_{T,A}+$$
$$r\,S\,\alpha_1\,p_{T,A}\,(1-p_{t,B}-p_{T,A}-p_{T,B})\,w_{T,A}-\alpha_2\,p_{T,A}\,(1-p_{t,B}-p_{T,A}-p_{T,B})\,w_{T,A}+$$
$$r\,\alpha_2\,p_{T,A}\,(1-p_{t,B}-p_{T,A}-p_{T,B})\,w_{T,A}+S\,\alpha_2\,p_{T,A}\,(1-p_{t,B}-p_{T,A}-p_{T,B})\,w_{T,A}-$$
$$r\,S\,\alpha_2\,p_{T,A}\,(1-p_{t,B}-p_{T,A}-p_{T,B})\,w_{T,A}+S\,\beta_2\,p_{T,B}\,w_{T,A}+r\,\beta_1\,p_{t,B}\,p_{T,B}\,w_{T,A}-$$
$$r\,S\,\beta_1\,p_{t,B}\,p_{T,B}\,w_{T,A}+\beta_2\,p_{t,B}\,p_{T,B}\,w_{T,A}-r\,\beta_2\,p_{t,B}\,p_{T,B}\,w_{T,A}-$$
$$S\,\beta_2\,p_{t,B}\,p_{T,B}\,w_{T,A}+r\,S\,\beta_2\,p_{t,B}\,p_{T,B}\,w_{T,A}+p_{T,A}\,p_{T,B}\,w_{T,A}-S\,p_{T,A}\,p_{T,B}\,w_{T,A}-$$
$$\alpha_2\,p_{T,A}\,p_{T,B}\,w_{T,A}+S\,\alpha_2\,p_{T,A}\,p_{T,B}\,w_{T,A}+\beta_2\,p_{T,A}\,p_{T,B}\,w_{T,A}-S\,\beta_2\,p_{T,A}\,p_{T,B}\,w_{T,A}+$$

$$r\,(1-p_{t,B}-p_{T,A}-p_{T,B})\,p_{T,B}\,w_{T,A}-r\,S\,(1-p_{t,B}-p_{T,A}-p_{T,B})\,p_{T,B}\,w_{T,A}-$$
$$r\,\alpha_1\,(1-p_{t,B}-p_{T,A}-p_{T,B})\,p_{T,B}\,w_{T,A}+r\,S\,\alpha_1\,(1-p_{t,B}-p_{T,A}-p_{T,B})\,p_{T,B}\,w_{T,A}+$$
$$\beta_2\,(1-p_{t,B}-p_{T,A}-p_{T,B})\,p_{T,B}\,w_{T,A}-r\,\beta_2\,(1-p_{t,B}-p_{T,A}-p_{T,B})\,p_{T,B}\,w_{T,A}-$$
$$S\,\beta_2\,(1-p_{t,B}-p_{T,A}-p_{T,B})\,p_{T,B}\,w_{T,A}+r\,S\,\beta_2\,(1-p_{t,B}-p_{T,A}-p_{T,B})\,p_{T,B}\,w_{T,A}+$$
$$\beta_2\,p_{T,B}^2\,w_{T,A}-S\,\beta_2\,p_{T,B}^2\,w_{T,A}+S\,\alpha_2\,p_{T,A}\,w_{T,B}+r\,p_{t,B}\,p_{T,A}\,w_{T,B}-$$
$$r\,S\,p_{t,B}\,p_{T,A}\,w_{T,B}+\alpha_2\,p_{t,B}\,p_{T,A}\,w_{T,B}-r\,\alpha_2\,p_{t,B}\,p_{T,A}\,w_{T,B}-S\,\alpha_2\,p_{t,B}\,p_{T,A}\,w_{T,B}+$$
$$r\,S\,\alpha_2\,p_{t,B}\,p_{T,A}\,w_{T,B}-r\,\beta_1\,p_{t,B}\,p_{T,A}\,w_{T,B}+r\,S\,\beta_1\,p_{t,B}\,p_{T,A}\,w_{T,B}+$$
$$\alpha_2\,p_{T,A}^2\,w_{T,B}-S\,\alpha_2\,p_{T,A}^2\,w_{T,B}+r\,\alpha_1\,p_{T,A}\,(1-p_{t,B}-p_{T,A}-p_{T,B})\,w_{T,B}-$$
$$r\,S\,\alpha_1\,p_{T,A}\,(1-p_{t,B}-p_{T,A}-p_{T,B})\,w_{T,B}+\alpha_2\,p_{T,A}\,(1-p_{t,B}-p_{T,A}-p_{T,B})\,w_{T,B}-$$
$$r\,\alpha_2\,p_{T,A}\,(1-p_{t,B}-p_{T,A}-p_{T,B})\,w_{T,B}-S\,\alpha_2\,p_{T,A}\,(1-p_{t,B}-p_{T,A}-p_{T,B})\,w_{T,B}+$$
$$r\,S\,\alpha_2\,p_{T,A}\,(1-p_{t,B}-p_{T,A}-p_{T,B})\,w_{T,B}+S\,p_{T,B}\,w_{T,B}-S\,\beta_2\,p_{T,B}\,w_{T,B}+$$
$$p_{t,B}\,p_{T,B}\,w_{T,B}-S\,p_{t,B}\,p_{T,B}\,w_{T,B}-r\,\beta_1\,p_{t,B}\,p_{T,B}\,w_{T,B}+r\,S\,\beta_1\,p_{t,B}\,p_{T,B}\,w_{T,B}-$$
$$\beta_2\,p_{t,B}\,p_{T,B}\,w_{T,B}+r\,\beta_2\,p_{t,B}\,p_{T,B}\,w_{T,B}+S\,\beta_2\,p_{t,B}\,p_{T,B}\,w_{T,B}-r\,S\,\beta_2\,p_{t,B}\,p_{T,B}\,w_{T,B}+$$
$$p_{T,A}\,p_{T,B}\,w_{T,B}-S\,p_{T,A}\,p_{T,B}\,w_{T,B}+\alpha_2\,p_{T,A}\,p_{T,B}\,w_{T,B}-S\,\alpha_2\,p_{T,A}\,p_{T,B}\,w_{T,B}-$$
$$\beta_2\,p_{T,A}\,p_{T,B}\,w_{T,B}+S\,\beta_2\,p_{T,A}\,p_{T,B}\,w_{T,B}+(1-p_{t,B}-p_{T,A}-p_{T,B})\,p_{T,B}\,w_{T,B}-$$
$$r\,(1-p_{t,B}-p_{T,A}-p_{T,B})\,p_{T,B}\,w_{T,B}-S\,(1-p_{t,B}-p_{T,A}-p_{T,B})\,p_{T,B}\,w_{T,B}+$$
$$r\,S\,(1-p_{t,B}-p_{T,A}-p_{T,B})\,p_{T,B}\,w_{T,B}+r\,\alpha_1\,(1-p_{t,B}-p_{T,A}-p_{T,B})\,p_{T,B}\,w_{T,B}-$$
$$r\,S\,\alpha_1\,(1-p_{t,B}-p_{T,A}-p_{T,B})\,p_{T,B}\,w_{T,B}-\beta_2\,(1-p_{t,B}-p_{T,A}-p_{T,B})\,p_{T,B}\,w_{T,B}+$$
$$r\,\beta_2\,(1-p_{t,B}-p_{T,A}-p_{T,B})\,p_{T,B}\,w_{T,B}+S\,\beta_2\,(1-p_{t,B}-p_{T,A}-p_{T,B})\,p_{T,B}\,w_{T,B}-$$
$$r\,S\,\beta_2\,(1-p_{t,B}-p_{T,A}-p_{T,B})\,p_{T,B}\,w_{T,B}+p_{T,B}^2\,w_{T,B}-S\,p_{T,B}^2\,w_{T,B}-\beta_2\,p_{T,B}^2\,w_{T,B}+S\,\beta_2\,p_{T,B}^2\,w_{T,B}\Big),$$

$$0==-p_{T,B}+\Big(z\,\Big((-1+S)\,(-1+\beta_1)\,p_{t,B}^2+\alpha_1\,(1-p_{t,B}-p_{T,A}-p_{T,B})\,\Big(S-(-1+S)\,p_{t,B}+p_{T,A}-r\,p_{T,A}-S\,p_{T,A}+$$
$$r\,S\,p_{T,A}-(-1+S)\,(1-p_{t,B}-p_{T,A}-p_{T,B})+p_{T,B}-r\,p_{T,B}-S\,p_{T,B}+r\,S\,p_{T,B}\Big)-r\,(-1+S)$$
$$(1-p_{t,B}-p_{T,A}-p_{T,B})\,\big(\alpha_2\,p_{T,A}-(-1+\beta_2)\,p_{T,B}\big)+p_{t,B}\,\Big(1+S-p_{t,B}-r\,p_{T,A}-S\,p_{T,A}+r\,S\,p_{T,A}+$$
$$r\,\alpha_2\,p_{T,A}-r\,S\,\alpha_2\,p_{T,A}-S\,(1-p_{t,B}-p_{T,A}-p_{T,B})-S\,p_{T,B}-r\,\beta_2\,p_{T,B}+r\,S\,\beta_2\,p_{T,B}+\beta_1\,\Big(-S-$$
$$(-1+r)\,(-1+S)\,p_{T,A}+(-1+S)\,(1-p_{t,B}-p_{T,A}-p_{T,B})-p_{T,B}+r\,p_{T,B}+S\,p_{T,B}-r\,S\,p_{T,B}\Big)\Big)\Big)$$
$$w_{t,B}-(-1+y)\,\Big(r\,p_{t,B}\,p_{T,A}-r\,S\,p_{t,B}\,p_{T,A}-r\,\beta_1\,p_{t,B}\,p_{T,A}+r\,S\,\beta_1\,p_{t,B}\,p_{T,A}+S\,p_{T,B}-S\,\beta_2\,p_{T,B}+$$
$$p_{t,B}\,p_{T,B}-S\,p_{t,B}\,p_{T,B}-r\,\beta_1\,p_{t,B}\,p_{T,B}+r\,S\,\beta_1\,p_{t,B}\,p_{T,B}-\beta_2\,p_{t,B}\,p_{T,B}+r\,\beta_2\,p_{t,B}\,p_{T,B}+$$
$$S\,\beta_2\,p_{t,B}\,p_{T,B}-r\,S\,\beta_2\,p_{t,B}\,p_{T,B}+p_{T,A}\,p_{T,B}-S\,p_{T,A}\,p_{T,B}-\beta_2\,p_{T,A}\,p_{T,B}+S\,\beta_2\,p_{T,A}\,p_{T,B}+$$
$$(1-p_{t,B}-p_{T,A}-p_{T,B})\,p_{T,B}-r\,(1-p_{t,B}-p_{T,A}-p_{T,B})\,p_{T,B}-S\,(1-p_{t,B}-p_{T,A}-p_{T,B})\,p_{T,B}+$$
$$r\,S\,(1-p_{t,B}-p_{T,A}-p_{T,B})\,p_{T,B}-\beta_2\,(1-p_{t,B}-p_{T,A}-p_{T,B})\,p_{T,B}+r\,\beta_2\,(1-p_{t,B}-p_{T,A}-p_{T,B})\,p_{T,B}+$$
$$S\,\beta_2\,(1-p_{t,B}-p_{T,A}-p_{T,B})\,p_{T,B}-r\,S\,\beta_2\,(1-p_{t,B}-p_{T,A}-p_{T,B})\,p_{T,B}+p_{T,B}^2-S\,p_{T,B}^2-\beta_2\,p_{T,B}^2+$$
$$S\,\beta_2\,p_{T,B}^2+(-1+S)\,\alpha_1\,(1-p_{t,B}-p_{T,A}-p_{T,B})\,\big(-r\,p_{T,A}-r\,p_{T,B}\big)+\alpha_2\,p_{T,A}\,\Big(S+(-1+r)\,(-1+S)\,p_{t,B}+$$
$$p_{T,A}-S\,p_{T,A}+(-1+r)\,(-1+S)\,(1-p_{t,B}-p_{T,A}-p_{T,B})+p_{T,B}-S\,p_{T,B}\Big)\Big)\,w_{T,B}\Big)\Big/$$
$$\Big((-1+S)\,(-1+\alpha_1)\,(1-p_{t,B}-p_{T,A}-p_{T,B})^2+(1-p_{t,B}-p_{T,A}-p_{T,B})\,\Big(S+p_{t,B}-S\,p_{t,B}+\beta_1\,p_{t,B}-$$
$$S\,\beta_1\,p_{t,B}+p_{T,A}-S\,p_{T,A}-r\,\alpha_2\,p_{T,A}+r\,S\,\alpha_2\,p_{T,A}+p_{T,B}-r\,p_{T,B}-S\,p_{T,B}+r\,S\,p_{T,B}+r\,\beta_2\,p_{T,B}-$$
$$r\,S\,\beta_2\,p_{T,B}+\alpha_1\,\Big(-S+(-1+S)\,p_{t,B}-(-1+r)\,(-1+S)\,p_{T,A}-p_{T,B}+r\,p_{T,B}+S\,p_{T,B}-r\,S\,p_{T,B}\Big)\Big)+$$
$$p_{t,B}\,\Big(\beta_1\,\Big(S-(-1+S)\,p_{t,B}+(-1+r)\,(-1+S)\,p_{T,A}+p_{T,B}-r\,p_{T,B}-S\,p_{T,B}+r\,S\,p_{T,B}\Big)+$$
$$r\,(-1+S)\,\big((-1+\alpha_2)\,p_{T,A}-\beta_2\,p_{T,B}\big)\Big)+$$
$$\Big((-1+S)\,(-1+\beta_1)\,p_{t,B}^2+\alpha_1\,(1-p_{t,B}-p_{T,A}-p_{T,B})\,\Big(S-(-1+S)\,p_{t,B}+p_{T,A}-r\,p_{T,A}-S\,p_{T,A}+$$
$$r\,S\,p_{T,A}-(-1+S)\,(1-p_{t,B}-p_{T,A}-p_{T,B})+p_{T,B}-r\,p_{T,B}-S\,p_{T,B}+r\,S\,p_{T,B}\Big)-r\,(-1+S)$$
$$(1-p_{t,B}-p_{T,A}-p_{T,B})\,\big(\alpha_2\,p_{T,A}-(-1+\beta_2)\,p_{T,B}\big)+p_{t,B}\,\Big(1+S-p_{t,B}-r\,p_{T,A}-S\,p_{T,A}+r\,S\,p_{T,A}+$$

$$r\,\alpha_2\,p_{T,A} - r\,S\,\alpha_2\,p_{T,A} - S\,(1 - p_{t,B} - p_{T,A} - p_{T,B}) - S\,p_{T,B} - r\,\beta_2\,p_{T,B} + r\,S\,\beta_2\,p_{T,B} + \beta_1\,\Big(-S -$$
$$(-1 + r)\,(-1 + S)\,p_{T,A} + (-1 + S)\,(1 - p_{t,B} - p_{T,A} - p_{T,B}) - p_{T,B} + r\,p_{T,B} + S\,p_{T,B} - r\,S\,p_{T,B}\Big)\Big)\Big)$$
$$w_{t,B} + S\,p_{T,A}\,w_{T,A} - S\,\alpha_2\,p_{T,A}\,w_{T,A} + p_{t,B}\,p_{T,A}\,w_{T,A} - r\,p_{t,B}\,p_{T,A}\,w_{T,A} - S\,p_{t,B}\,p_{T,A}\,w_{T,A} +$$
$$r\,S\,p_{t,B}\,p_{T,A}\,w_{T,A} - \alpha_2\,p_{t,B}\,p_{T,A}\,w_{T,A} + r\,\alpha_2\,p_{t,B}\,p_{T,A}\,w_{T,A} + S\,\alpha_2\,p_{t,B}\,p_{T,A}\,w_{T,A} -$$
$$r\,S\,\alpha_2\,p_{t,B}\,p_{T,A}\,w_{T,A} + r\,\beta_1\,p_{t,B}\,p_{T,A}\,w_{T,A} - r\,S\,\beta_1\,p_{t,B}\,p_{T,A}\,w_{T,A} + p_{T,A}^2\,w_{T,A} -$$
$$S\,p_{T,A}^2\,w_{T,A} - \alpha_2\,p_{T,A}^2\,w_{T,A} + S\,\alpha_2\,p_{T,A}^2\,w_{T,A} + p_{T,A}\,(1 - p_{t,B} - p_{T,A} - p_{T,B})\,w_{T,A} -$$
$$S\,p_{T,A}\,(1 - p_{t,B} - p_{T,A} - p_{T,B})\,w_{T,A} - r\,\alpha_1\,p_{T,A}\,(1 - p_{t,B} - p_{T,A} - p_{T,B})\,w_{T,A} +$$
$$r\,S\,\alpha_1\,p_{T,A}\,(1 - p_{t,B} - p_{T,A} - p_{T,B})\,w_{T,A} - \alpha_2\,p_{T,A}\,(1 - p_{t,B} - p_{T,A} - p_{T,B})\,w_{T,A} +$$
$$r\,\alpha_2\,p_{T,A}\,(1 - p_{t,B} - p_{T,A} - p_{T,B})\,w_{T,A} + S\,\alpha_2\,p_{T,A}\,(1 - p_{t,B} - p_{T,A} - p_{T,B})\,w_{T,A} -$$
$$r\,S\,\alpha_2\,p_{T,A}\,(1 - p_{t,B} - p_{T,A} - p_{T,B})\,w_{T,A} + S\,\beta_2\,p_{T,B}\,w_{T,A} + r\,\beta_1\,p_{t,B}\,p_{T,B}\,w_{T,A} -$$
$$r\,S\,\beta_1\,p_{t,B}\,p_{T,B}\,w_{T,A} + \beta_2\,p_{t,B}\,p_{T,B}\,w_{T,A} - r\,\beta_2\,p_{t,B}\,p_{T,B}\,w_{T,A} -$$
$$S\,\beta_2\,p_{t,B}\,p_{T,B}\,w_{T,A} + r\,S\,\beta_2\,p_{t,B}\,p_{T,B}\,w_{T,A} + p_{T,A}\,p_{T,B}\,w_{T,A} - S\,p_{T,A}\,p_{T,B}\,w_{T,A} -$$
$$\alpha_2\,p_{T,A}\,p_{T,B}\,w_{T,A} + S\,\alpha_2\,p_{T,A}\,p_{T,B}\,w_{T,A} + \beta_2\,p_{T,A}\,p_{T,B}\,w_{T,A} - S\,\beta_2\,p_{T,A}\,p_{T,B}\,w_{T,A} +$$
$$r\,(1 - p_{t,B} - p_{T,A} - p_{T,B})\,p_{T,B}\,w_{T,A} - r\,S\,(1 - p_{t,B} - p_{T,A} - p_{T,B})\,p_{T,B}\,w_{T,A} -$$
$$r\,\alpha_1\,(1 - p_{t,B} - p_{T,A} - p_{T,B})\,p_{T,B}\,w_{T,A} + r\,S\,\alpha_1\,(1 - p_{t,B} - p_{T,A} - p_{T,B})\,p_{T,B}\,w_{T,A} +$$
$$\beta_2\,(1 - p_{t,B} - p_{T,A} - p_{T,B})\,p_{T,B}\,w_{T,A} - r\,\beta_2\,(1 - p_{t,B} - p_{T,A} - p_{T,B})\,p_{T,B}\,w_{T,A} -$$
$$S\,\beta_2\,(1 - p_{t,B} - p_{T,A} - p_{T,B})\,p_{T,B}\,w_{T,A} + r\,S\,\beta_2\,(1 - p_{t,B} - p_{T,A} - p_{T,B})\,p_{T,B}\,w_{T,A} +$$
$$\beta_2\,p_{T,B}^2\,w_{T,A} - S\,\beta_2\,p_{T,B}^2\,w_{T,A} + S\,\alpha_2\,p_{T,A}\,w_{T,B} + r\,p_{t,B}\,p_{T,A}\,w_{T,B} - r\,S\,p_{t,B}\,p_{T,A}\,w_{T,B} +$$
$$\alpha_2\,p_{t,B}\,p_{T,A}\,w_{T,B} - r\,\alpha_2\,p_{t,B}\,p_{T,A}\,w_{T,B} - S\,\alpha_2\,p_{t,B}\,p_{T,A}\,w_{T,B} + r\,S\,\alpha_2\,p_{t,B}\,p_{T,A}\,w_{T,B} -$$
$$r\,\beta_1\,p_{t,B}\,p_{T,A}\,w_{T,B} + r\,S\,\beta_1\,p_{t,B}\,p_{T,A}\,w_{T,B} + \alpha_2\,p_{T,A}^2\,w_{T,B} - S\,\alpha_2\,p_{T,A}^2\,w_{T,B} +$$
$$r\,\alpha_1\,p_{T,A}\,(1 - p_{t,B} - p_{T,A} - p_{T,B})\,w_{T,B} - r\,S\,\alpha_1\,p_{T,A}\,(1 - p_{t,B} - p_{T,A} - p_{T,B})\,w_{T,B} +$$
$$\alpha_2\,p_{T,A}\,(1 - p_{t,B} - p_{T,A} - p_{T,B})\,w_{T,B} - r\,\alpha_2\,p_{T,A}\,(1 - p_{t,B} - p_{T,A} - p_{T,B})\,w_{T,B} -$$
$$S\,\alpha_2\,p_{T,A}\,(1 - p_{t,B} - p_{T,A} - p_{T,B})\,w_{T,B} + r\,S\,\alpha_2\,p_{T,A}\,(1 - p_{t,B} - p_{T,A} - p_{T,B})\,w_{T,B} +$$
$$S\,p_{T,B}\,w_{T,B} - S\,\beta_2\,p_{T,B}\,w_{T,B} + p_{t,B}\,p_{T,B}\,w_{T,B} - S\,p_{t,B}\,p_{T,B}\,w_{T,B} -$$
$$r\,\beta_1\,p_{t,B}\,p_{T,B}\,w_{T,B} + r\,S\,\beta_1\,p_{t,B}\,p_{T,B}\,w_{T,B} - \beta_2\,p_{t,B}\,p_{T,B}\,w_{T,B} + r\,\beta_2\,p_{t,B}\,p_{T,B}\,w_{T,B} +$$
$$S\,\beta_2\,p_{t,B}\,p_{T,B}\,w_{T,B} - r\,S\,\beta_2\,p_{t,B}\,p_{T,B}\,w_{T,B} + p_{T,A}\,p_{T,B}\,w_{T,B} - S\,p_{T,A}\,p_{T,B}\,w_{T,B} +$$
$$\alpha_2\,p_{T,A}\,p_{T,B}\,w_{T,B} - S\,\alpha_2\,p_{T,A}\,p_{T,B}\,w_{T,B} - \beta_2\,p_{T,A}\,p_{T,B}\,w_{T,B} + S\,\beta_2\,p_{T,A}\,p_{T,B}\,w_{T,B} +$$
$$(1 - p_{t,B} - p_{T,A} - p_{T,B})\,p_{T,B}\,w_{T,B} - r\,(1 - p_{t,B} - p_{T,A} - p_{T,B})\,p_{T,B}\,w_{T,B} -$$
$$S\,(1 - p_{t,B} - p_{T,A} - p_{T,B})\,p_{T,B}\,w_{T,B} + r\,S\,(1 - p_{t,B} - p_{T,A} - p_{T,B})\,p_{T,B}\,w_{T,B} +$$
$$r\,\alpha_1\,(1 - p_{t,B} - p_{T,A} - p_{T,B})\,p_{T,B}\,w_{T,B} - r\,S\,\alpha_1\,(1 - p_{t,B} - p_{T,A} - p_{T,B})\,p_{T,B}\,w_{T,B} -$$
$$\beta_2\,(1 - p_{t,B} - p_{T,A} - p_{T,B})\,p_{T,B}\,w_{T,B} + r\,\beta_2\,(1 - p_{t,B} - p_{T,A} - p_{T,B})\,p_{T,B}\,w_{T,B} +$$
$$S\,\beta_2\,(1 - p_{t,B} - p_{T,A} - p_{T,B})\,p_{T,B}\,w_{T,B} - r\,S\,\beta_2\,(1 - p_{t,B} - p_{T,A} - p_{T,B})\,p_{T,B}\,w_{T,B} +$$
$$p_{T,B}^2\,w_{T,B} - S\,p_{T,B}^2\,w_{T,B} - \beta_2\,p_{T,B}^2\,w_{T,B} + S\,\beta_2\,p_{T,B}^2\,w_{T,B})\Big)\Big\}\;/.$$
$$\Big\{z \to z\,\zeta,\; y \to y\,\zeta,\; \beta_2 \to \beta_2\,\zeta,\; \alpha_1 \to \alpha_1\,\zeta,\; p_{T,A} \to (p_{T,A,0} + p_{T,A,1}\,\zeta),$$
$$p_{t,B} \to$$
$$(p_{t,B,0} + p_{t,B,1}\,\zeta),\; p_{T,B} \to$$
$$(p_{T,B,0} + p_{T,B,1}\,\zeta)\Big\};$$

In[∘]:=
```
Series[%, {ζ, 0, 1}];
```

Evaluating the first zeroth order terms : $\{p_{T,A,0} \to 0,\ p_{t,B,0} \to 0,\ p_{T,B,0} \to 0\}$

In[∘]:=

$$\Big\{\Big(-p_{t,B,1} - \Big(w_{t,B}\,\Big((-1 + S)\,(-1 + \beta_1)\,p_{t,B,0}^2 + r\,(-1 + S)\,(-1 + p_{t,B,0} + p_{T,A,0} + p_{T,B,0})\,(\alpha_2\,p_{T,A,0} + p_{T,B,0}) -$$

$$p_{t,B,0} (-1 + \beta_1 + p_{t,B,0} - S\, p_{t,B,0} - \beta_1\, p_{t,B,0} + S\, \beta_1\, p_{t,B,0} + r\, p_{T,A,0} - r\, S\, p_{T,A,0} -$$

$$r\, \alpha_2\, p_{T,A,0} + r\, S\, \alpha_2\, p_{T,A,0} - r\, \beta_1\, p_{T,A,0} + r\, S\, \beta_1\, p_{T,A,0} - r\, \beta_1\, p_{T,B,0} + r\, S\, \beta_1\, p_{T,B,0}))$$

$$(S\, w_{T,A}\, p_{T,A,1} - S\, \alpha_2\, w_{T,A}\, p_{T,A,1} + S\, \alpha_2\, w_{T,B}\, p_{T,A,1} + 2\, w_{T,A}\, p_{T,A,0}\, p_{T,A,1} -$$

$$2\, S\, w_{T,A}\, p_{T,A,0}\, p_{T,A,1} - 2\, \alpha_2\, w_{T,A}\, p_{T,A,0}\, p_{T,A,1} + 2\, S\, \alpha_2\, w_{T,A}\, p_{T,A,0}\, p_{T,A,1} +$$

$$2\, \alpha_2\, w_{T,B}\, p_{T,A,0}\, p_{T,A,1} - 2\, S\, \alpha_2\, w_{T,B}\, p_{T,A,0}\, p_{T,A,1} + w_{T,A} (p_{t,B,1}\, p_{T,A,0} + p_{t,B,0}\, p_{T,A,1}) -$$

$$r\, w_{T,A} (p_{t,B,1}\, p_{T,A,0} + p_{t,B,0}\, p_{T,A,1}) - S\, w_{T,A} (p_{t,B,1}\, p_{T,A,0} + p_{t,B,0}\, p_{T,A,1}) +$$

$$r\, S\, w_{T,A} (p_{t,B,1}\, p_{T,A,0} + p_{t,B,0}\, p_{T,A,1}) - \alpha_2\, w_{T,A} (p_{t,B,1}\, p_{T,A,0} + p_{t,B,0}\, p_{T,A,1}) +$$

$$r\, \alpha_2\, w_{T,A} (p_{t,B,1}\, p_{T,A,0} + p_{t,B,0}\, p_{T,A,1}) + S\, \alpha_2\, w_{T,A} (p_{t,B,1}\, p_{T,A,0} + p_{t,B,0}\, p_{T,A,1}) -$$

$$r\, S\, \alpha_2\, w_{T,A} (p_{t,B,1}\, p_{T,A,0} + p_{t,B,0}\, p_{T,A,1}) + r\, \beta_1\, w_{T,A} (p_{t,B,1}\, p_{T,A,0} + p_{t,B,0}\, p_{T,A,1}) -$$

$$r\, S\, \beta_1\, w_{T,A} (p_{t,B,1}\, p_{T,A,0} + p_{t,B,0}\, p_{T,A,1}) + r\, w_{T,B} (p_{t,B,1}\, p_{T,A,0} + p_{t,B,0}\, p_{T,A,1}) -$$

$$r\, S\, w_{T,B} (p_{t,B,1}\, p_{T,A,0} + p_{t,B,0}\, p_{T,A,1}) + \alpha_2\, w_{T,B} (p_{t,B,1}\, p_{T,A,0} + p_{t,B,0}\, p_{T,A,1}) -$$

$$r\, \alpha_2\, w_{T,B} (p_{t,B,1}\, p_{T,A,0} + p_{t,B,0}\, p_{T,A,1}) - S\, \alpha_2\, w_{T,B} (p_{t,B,1}\, p_{T,A,0} + p_{t,B,0}\, p_{T,A,1}) +$$

$$r\, S\, \alpha_2\, w_{T,B} (p_{t,B,1}\, p_{T,A,0} + p_{t,B,0}\, p_{T,A,1}) - r\, \beta_1\, w_{T,B} (p_{t,B,1}\, p_{T,A,0} + p_{t,B,0}\, p_{T,A,1}) +$$

$$r\, S\, \beta_1\, w_{T,B} (p_{t,B,1}\, p_{T,A,0} + p_{t,B,0}\, p_{T,A,1}) + S\, \beta_2\, w_{T,A}\, p_{T,B,0} - S\, \beta_2\, w_{T,B}\, p_{T,B,0} +$$

$$\beta_2\, w_{T,A}\, p_{t,B,0}\, p_{T,B,0} - r\, \beta_2\, w_{T,A}\, p_{t,B,0}\, p_{T,B,0} - S\, \beta_2\, w_{T,A}\, p_{t,B,0}\, p_{T,B,0} +$$

$$r\, S\, \beta_2\, w_{T,A}\, p_{t,B,0}\, p_{T,B,0} - \beta_2\, w_{T,B}\, p_{t,B,0}\, p_{T,B,0} + r\, \beta_2\, w_{T,B}\, p_{t,B,0}\, p_{T,B,0} +$$

$$S\, \beta_2\, w_{T,B}\, p_{t,B,0}\, p_{T,B,0} - r\, S\, \beta_2\, w_{T,B}\, p_{t,B,0}\, p_{T,B,0} + \beta_2\, w_{T,A}\, p_{T,A,0}\, p_{T,B,0} -$$

$$S\, \beta_2\, w_{T,A}\, p_{T,A,0}\, p_{T,B,0} - \beta_2\, w_{T,B}\, p_{T,A,0}\, p_{T,B,0} + S\, \beta_2\, w_{T,B}\, p_{T,A,0}\, p_{T,B,0} +$$

$$\beta_2\, w_{T,A}\, p_{T,B,0}^2 - S\, \beta_2\, w_{T,A}\, p_{T,B,0}^2 - \beta_2\, w_{T,B}\, p_{T,B,0}^2 + S\, \beta_2\, w_{T,B}\, p_{T,B,0}^2 + r\, \alpha_1\, w_{T,A}\, p_{T,A,0}$$

$$(-1 + p_{t,B,0} + p_{T,A,0} + p_{T,B,0}) - r\, S\, \alpha_1\, w_{T,A}\, p_{T,A,0} (-1 + p_{t,B,0} + p_{T,A,0} + p_{T,B,0}) -$$

$$r\, \alpha_1\, w_{T,B}\, p_{T,A,0} (-1 + p_{t,B,0} + p_{T,A,0} + p_{T,B,0}) + r\, S\, \alpha_1\, w_{T,B}\, p_{T,A,0}$$

$$(-1 + p_{t,B,0} + p_{T,A,0} + p_{T,B,0}) + r\, \alpha_1\, w_{T,A}\, p_{T,B,0} (-1 + p_{t,B,0} + p_{T,A,0} + p_{T,B,0}) -$$

$$r\, S\, \alpha_1\, w_{T,A}\, p_{T,B,0} (-1 + p_{t,B,0} + p_{T,A,0} + p_{T,B,0}) - \beta_2\, w_{T,A}\, p_{T,B,0} (-1 + p_{t,B,0} + p_{T,A,0} + p_{T,B,0}) +$$

$$r\, \beta_2\, w_{T,A}\, p_{T,B,0} (-1 + p_{t,B,0} + p_{T,A,0} + p_{T,B,0}) + S\, \beta_2\, w_{T,A}\, p_{T,B,0} (-1 + p_{t,B,0} + p_{T,A,0} + p_{T,B,0}) -$$

$$r\, S\, \beta_2\, w_{T,A}\, p_{T,B,0} (-1 + p_{t,B,0} + p_{T,A,0} + p_{T,B,0}) - r\, \alpha_1\, w_{T,B}\, p_{T,B,0} (-1 + p_{t,B,0} + p_{T,A,0} + p_{T,B,0}) +$$

$$r\, S\, \alpha_1\, w_{T,B}\, p_{T,B,0} (-1 + p_{t,B,0} + p_{T,A,0} + p_{T,B,0}) + \beta_2\, w_{T,B}\, p_{T,B,0} (-1 + p_{t,B,0} + p_{T,A,0} + p_{T,B,0}) -$$

$$r\, \beta_2\, w_{T,B}\, p_{T,B,0} (-1 + p_{t,B,0} + p_{T,A,0} + p_{T,B,0}) - S\, \beta_2\, w_{T,B}\, p_{T,B,0} (-1 + p_{t,B,0} + p_{T,A,0} + p_{T,B,0}) +$$

$$r\, S\, \beta_2\, w_{T,B}\, p_{T,B,0} (-1 + p_{t,B,0} + p_{T,A,0} + p_{T,B,0}) + p_{t,B,1} (r (-1 + S) (-1 + \alpha_2)\, p_{T,A,0} +$$

$$\beta_1 (S - (-1 + S)\, p_{t,B,0} + (-1 + r) (-1 + S)\, p_{T,A,0} + p_{T,B,0} - r\, p_{T,B,0} - S\, p_{T,B,0} + r\, S\, p_{T,B,0})) +$$

$$S\, w_{T,A} (-p_{T,A,1} (1 - p_{t,B,0} - p_{T,A,0} - p_{T,B,0}) - p_{T,A,0} (-p_{t,B,1} - p_{T,A,1} - p_{T,B,1})) +$$

$$\alpha_2\, w_{T,A} (-p_{T,A,1} (1 - p_{t,B,0} - p_{T,A,0} - p_{T,B,0}) - p_{T,A,0} (-p_{t,B,1} - p_{T,A,1} - p_{T,B,1})) +$$

$$r\, S\, \alpha_2\, w_{T,A} (-p_{T,A,1} (1 - p_{t,B,0} - p_{T,A,0} - p_{T,B,0}) - p_{T,A,0} (-p_{t,B,1} - p_{T,A,1} - p_{T,B,1})) +$$

$$r\, \alpha_2\, w_{T,B} (-p_{T,A,1} (1 - p_{t,B,0} - p_{T,A,0} - p_{T,B,0}) - p_{T,A,0} (-p_{t,B,1} - p_{T,A,1} - p_{T,B,1})) +$$

$$S\, \alpha_2\, w_{T,B} (-p_{T,A,1} (1 - p_{t,B,0} - p_{T,A,0} - p_{T,B,0}) - p_{T,A,0} (-p_{t,B,1} - p_{T,A,1} - p_{T,B,1})) +$$

$$w_{T,A} (p_{T,A,1} (1 - p_{t,B,0} - p_{T,A,0} - p_{T,B,0}) + p_{T,A,0} (-p_{t,B,1} - p_{T,A,1} - p_{T,B,1})) +$$

$$r\, \alpha_2\, w_{T,A} (p_{T,A,1} (1 - p_{t,B,0} - p_{T,A,0} - p_{T,B,0}) + p_{T,A,0} (-p_{t,B,1} - p_{T,A,1} - p_{T,B,1})) +$$

$$S\, \alpha_2\, w_{T,A} (p_{T,A,1} (1 - p_{t,B,0} - p_{T,A,0} - p_{T,B,0}) + p_{T,A,0} (-p_{t,B,1} - p_{T,A,1} - p_{T,B,1})) +$$

$$\alpha_2\, w_{T,B} (p_{T,A,1} (1 - p_{t,B,0} - p_{T,A,0} - p_{T,B,0}) + p_{T,A,0} (-p_{t,B,1} - p_{T,A,1} - p_{T,B,1})) +$$

$$r\, S\, \alpha_2\, w_{T,B} (p_{T,A,1} (1 - p_{t,B,0} - p_{T,A,0} - p_{T,B,0}) + p_{T,A,0} (-p_{t,B,1} - p_{T,A,1} - p_{T,B,1})) +$$

$$(S + p_{t,B,0} - S\, p_{t,B,0} + \beta_1\, p_{t,B,0} - S\, \beta_1\, p_{t,B,0} + p_{T,A,0} - S\, p_{T,A,0} - r\, \alpha_2\, p_{T,A,0} + r\, S\, \alpha_2\, p_{T,A,0} +$$

$$p_{T,B,0} - r\, p_{T,B,0} - S\, p_{T,B,0} + r\, S\, p_{T,B,0}) (-p_{t,B,1} - p_{T,A,1} - p_{T,B,1}) + S\, w_{T,B}\, p_{T,B,1} +$$

$$2\, w_{T,B}\, p_{T,B,0}\, p_{T,B,1} - 2\, S\, w_{T,B}\, p_{T,B,0}\, p_{T,B,1} + (1 - p_{t,B,0} - p_{T,A,0} - p_{T,B,0})$$

$$(p_{t,B,1} - S\, p_{t,B,1} + \beta_1\, p_{t,B,1} - S\, \beta_1\, p_{t,B,1} + p_{T,A,1} - S\, p_{T,A,1} - r\, \alpha_2\, p_{T,A,1} + r\, S\, \alpha_2\, p_{T,A,1} +$$

$$r\, \beta_2\, p_{T,B,0} - r\, S\, \beta_2\, p_{T,B,0} + \alpha_1\, (-S + (-1 + S)\, p_{t,B,0} - (-1 + r)\, (-1 + S)\, p_{T,A,0} - p_{T,B,0} +$$

$$r\, p_{T,B,0} + S\, p_{T,B,0} - r\, S\, p_{T,B,0}) + p_{T,B,1} - r\, p_{T,B,1} - S\, p_{T,B,1} + r\, S\, p_{T,B,1}) +$$

$$r\, \beta_1\, w_{T,A}\, (p_{t,B,1}\, p_{T,B,0} + p_{t,B,0}\, p_{T,B,1}) - r\, S\, \beta_1\, w_{T,A}\, (p_{t,B,1}\, p_{T,B,0} + p_{t,B,0}\, p_{T,B,1}) +$$

$$w_{T,B}\, (p_{t,B,1}\, p_{T,B,0} + p_{t,B,0}\, p_{T,B,1}) - S\, w_{T,B}\, (p_{t,B,1}\, p_{T,B,0} + p_{t,B,0}\, p_{T,B,1}) -$$

$$r\, \beta_1\, w_{T,B}\, (p_{t,B,1}\, p_{T,B,0} + p_{t,B,0}\, p_{T,B,1}) + r\, S\, \beta_1\, w_{T,B}\, (p_{t,B,1}\, p_{T,B,0} + p_{t,B,0}\, p_{T,B,1}) +$$

$$w_{T,A}\, (p_{T,A,1}\, p_{T,B,0} + p_{T,A,0}\, p_{T,B,1}) - S\, w_{T,A}\, (p_{T,A,1}\, p_{T,B,0} + p_{T,A,0}\, p_{T,B,1}) -$$

$$\alpha_2\, w_{T,A}\, (p_{T,A,1}\, p_{T,B,0} + p_{T,A,0}\, p_{T,B,1}) + S\, \alpha_2\, w_{T,A}\, (p_{T,A,1}\, p_{T,B,0} + p_{T,A,0}\, p_{T,B,1}) +$$

$$w_{T,B}\, (p_{T,A,1}\, p_{T,B,0} + p_{T,A,0}\, p_{T,B,1}) - S\, w_{T,B}\, (p_{T,A,1}\, p_{T,B,0} + p_{T,A,0}\, p_{T,B,1}) +$$

$$\alpha_2\, w_{T,B}\, (p_{T,A,1}\, p_{T,B,0} + p_{T,A,0}\, p_{T,B,1}) - S\, \alpha_2\, w_{T,B}\, (p_{T,A,1}\, p_{T,B,0} + p_{T,A,0}\, p_{T,B,1}) +$$

$$r\, S\, w_{T,A}\, (-p_{T,B,0}\, (-p_{t,B,1} - p_{T,A,1} - p_{T,B,1}) - (1 - p_{t,B,0} - p_{T,A,0} - p_{T,B,0})\, p_{T,B,1}) +$$

$$r\, w_{T,B}\, (-p_{T,B,0}\, (-p_{t,B,1} - p_{T,A,1} - p_{T,B,1}) - (1 - p_{t,B,0} - p_{T,A,0} - p_{T,B,0})\, p_{T,B,1}) +$$

$$S\, w_{T,B}\, (-p_{T,B,0}\, (-p_{t,B,1} - p_{T,A,1} - p_{T,B,1}) - (1 - p_{t,B,0} - p_{T,A,0} - p_{T,B,0})\, p_{T,B,1}) +$$

$$r\, w_{T,A}\, (p_{T,B,0}\, (-p_{t,B,1} - p_{T,A,1} - p_{T,B,1}) + (1 - p_{t,B,0} - p_{T,A,0} - p_{T,B,0})\, p_{T,B,1}) +$$

$$w_{T,B}\, (p_{T,B,0}\, (-p_{t,B,1} - p_{T,A,1} - p_{T,B,1}) + (1 - p_{t,B,0} - p_{T,A,0} - p_{T,B,0})\, p_{T,B,1}) +$$

$$r\, S\, w_{T,B}\, (p_{T,B,0}\, (-p_{t,B,1} - p_{T,A,1} - p_{T,B,1}) + (1 - p_{t,B,0} - p_{T,A,0} - p_{T,B,0})\, p_{T,B,1}) + (-1 + S)$$

$$(\alpha_1\, (1 - p_{t,B,0} - p_{T,A,0} - p_{T,B,0})^2 - 2\, (-1 + p_{t,B,0} + p_{T,A,0} + p_{T,B,0})\, (p_{t,B,1} + p_{T,A,1} + p_{T,B,1})) +$$

$$p_{t,B,0}\, (r\, (-1 + S)\, ((-1 + \alpha_2)\, p_{T,A,1} - \beta_2\, p_{T,B,0}) + \beta_1\, (-((-1 + S)\, p_{t,B,1}) + (-1 + r)\, (-1 + S)\, p_{T,A,1} +$$

$$p_{T,B,1} - r\, p_{T,B,1} - S\, p_{T,B,1} + r\, S\, p_{T,B,1})) + w_{t,B}\, (2\, (-1 + S)\, (-1 + \beta_1)\, p_{t,B,0}\, p_{t,B,1} -$$

$$\alpha_1\, (-1 + p_{t,B,0} + p_{T,A,0} + p_{T,B,0})\, (1 - r\, p_{T,A,0} + r\, S\, p_{T,A,0} - r\, p_{T,B,0} + r\, S\, p_{T,B,0}) +$$

$$p_{t,B,1}\, (1 - \beta_1 - p_{t,B,0} + S\, p_{t,B,0} + \beta_1\, p_{t,B,0} - S\, \beta_1\, p_{t,B,0} - r\, p_{T,A,0} + r\, S\, p_{T,A,0} +$$

$$r\, \alpha_2\, p_{T,A,0} - r\, S\, \alpha_2\, p_{T,A,0} + r\, \beta_1\, p_{T,A,0} - r\, S\, \beta_1\, p_{T,A,0} + r\, \beta_1\, p_{T,B,0} - r\, S\, \beta_1\, p_{T,B,0}) +$$

$$p_{t,B,0}\, (-p_{t,B,1} + S\, p_{t,B,1} + \beta_1\, p_{t,B,1} - S\, \beta_1\, p_{t,B,1} - r\, p_{T,A,1} + r\, S\, p_{T,A,1} +$$

$$r\, \alpha_2\, p_{T,A,1} - r\, S\, \alpha_2\, p_{T,A,1} + r\, \beta_1\, p_{T,A,1} - r\, S\, \beta_1\, p_{T,A,1} - r\, \beta_2\, p_{T,B,0} + r\, S\, \beta_2\, p_{T,B,0} +$$

$$r\, \beta_1\, p_{T,B,1} - r\, S\, \beta_1\, p_{T,B,1}) + r\, (-1 + S)\, (-((\alpha_2\, p_{T,A,0} + p_{T,B,0})\, (-p_{t,B,1} - p_{T,A,1} - p_{T,B,1})) -$$

$$(1 - p_{t,B,0} - p_{T,A,0} - p_{T,B,0})\, (\alpha_2\, p_{T,A,1} - \beta_2\, p_{T,B,0} + p_{T,B,1}))))) \Big/$$

$$(1 - p_{t,B,0} + \beta_1\, p_{t,B,0} + w_{t,B}\, p_{t,B,0} - \beta_1\, w_{t,B}\, p_{t,B,0} - p_{T,A,0} - r\, \alpha_2\, p_{T,A,0} + r\, S\, \alpha_2\, p_{T,A,0} +$$

$$r\, \alpha_2\, w_{t,B}\, p_{T,A,0} - r\, S\, \alpha_2\, w_{t,B}\, p_{T,A,0} + w_{T,A}\, p_{T,A,0} - \alpha_2\, w_{T,A}\, p_{T,A,0} + r\, \alpha_2\, w_{T,A}\, p_{T,A,0} -$$

$$r\, S\, \alpha_2\, w_{T,A}\, p_{T,A,0} + \alpha_2\, w_{T,B}\, p_{T,A,0} - r\, \alpha_2\, w_{T,B}\, p_{T,A,0} + r\, S\, \alpha_2\, w_{T,B}\, p_{T,A,0} + r\, p_{t,B,0}\, p_{T,A,0} -$$

$$r\, S\, p_{t,B,0}\, p_{T,A,0} - r\, \beta_1\, p_{t,B,0}\, p_{T,A,0} + r\, S\, \beta_1\, p_{t,B,0}\, p_{T,A,0} - r\, w_{t,B}\, p_{t,B,0}\, p_{T,A,0} +$$

$$r\, S\, w_{t,B}\, p_{t,B,0}\, p_{T,A,0} + r\, \beta_1\, w_{t,B}\, p_{t,B,0}\, p_{T,A,0} - r\, S\, \beta_1\, w_{t,B}\, p_{t,B,0}\, p_{T,A,0} -$$

$$r\, w_{T,A}\, p_{t,B,0}\, p_{T,A,0} + r\, S\, w_{T,A}\, p_{t,B,0}\, p_{T,A,0} + r\, \beta_1\, w_{T,A}\, p_{t,B,0}\, p_{T,A,0} -$$

$$r\, S\, \beta_1\, w_{T,A}\, p_{t,B,0}\, p_{T,A,0} + r\, w_{T,B}\, p_{t,B,0}\, p_{T,A,0} - r\, S\, w_{T,B}\, p_{t,B,0}\, p_{T,A,0} -$$

$$r\, \beta_1\, w_{T,B}\, p_{t,B,0}\, p_{T,A,0} + r\, S\, \beta_1\, w_{T,B}\, p_{t,B,0}\, p_{T,A,0} + r\, \alpha_2\, p_{T,A,0}^2 - r\, S\, \alpha_2\, p_{T,A,0}^2 -$$

$$r\, \alpha_2\, w_{t,B}\, p_{T,A,0}^2 + r\, S\, \alpha_2\, w_{t,B}\, p_{T,A,0}^2 - r\, \alpha_2\, w_{T,A}\, p_{T,A,0}^2 + r\, S\, \alpha_2\, w_{T,A}\, p_{T,A,0}^2 +$$

$$r\, \alpha_2\, w_{T,B}\, p_{T,A,0}^2 - r\, S\, \alpha_2\, w_{T,B}\, p_{T,A,0}^2 - p_{T,B,0} - r\, p_{T,B,0} + r\, S\, p_{T,B,0} + r\, w_{t,B}\, p_{T,B,0} -$$

$$r\, S\, w_{t,B}\, p_{T,B,0} + r\, w_{T,A}\, p_{T,B,0} - r\, S\, w_{T,A}\, p_{T,B,0} + w_{T,B}\, p_{T,B,0} - r\, w_{T,B}\, p_{T,B,0} +$$

$$r S w_{T,B} p_{T,B,0} + r p_{t,B,0} p_{T,B,0} - r S p_{t,B,0} p_{T,B,0} - r \beta_1 p_{t,B,0} p_{T,B,0} +$$
$$r S \beta_1 p_{t,B,0} p_{T,B,0} - r w_{t,B} p_{t,B,0} p_{T,B,0} + r S w_{t,B} p_{t,B,0} p_{T,B,0} + r \beta_1 w_{t,B} p_{t,B,0} p_{T,B,0} -$$
$$r S \beta_1 w_{t,B} p_{t,B,0} p_{T,B,0} - r w_{T,A} p_{t,B,0} p_{T,B,0} + r S w_{T,A} p_{t,B,0} p_{T,B,0} +$$
$$r \beta_1 w_{T,A} p_{t,B,0} p_{T,B,0} - r S \beta_1 w_{T,A} p_{t,B,0} p_{T,B,0} + r w_{T,B} p_{t,B,0} p_{T,B,0} -$$
$$r S w_{T,B} p_{t,B,0} p_{T,B,0} - r \beta_1 w_{T,B} p_{t,B,0} p_{T,B,0} + r S \beta_1 w_{T,B} p_{t,B,0} p_{T,B,0} + r p_{T,A,0} p_{T,B,0} -$$
$$r S p_{T,A,0} p_{T,B,0} + r \alpha_2 p_{T,A,0} p_{T,B,0} - r S \alpha_2 p_{T,A,0} p_{T,B,0} - r w_{t,B} p_{T,A,0} p_{T,B,0} +$$
$$r S w_{t,B} p_{T,A,0} p_{T,B,0} - r \alpha_2 w_{t,B} p_{T,A,0} p_{T,B,0} + r S \alpha_2 w_{t,B} p_{T,A,0} p_{T,B,0} -$$
$$r w_{T,A} p_{T,A,0} p_{T,B,0} + r S w_{T,A} p_{T,A,0} p_{T,B,0} - r \alpha_2 w_{T,A} p_{T,A,0} p_{T,B,0} +$$
$$r S \alpha_2 w_{T,A} p_{T,A,0} p_{T,B,0} + r w_{T,B} p_{T,A,0} p_{T,B,0} - r S w_{T,B} p_{T,A,0} p_{T,B,0} +$$
$$r \alpha_2 w_{T,B} p_{T,A,0} p_{T,B,0} - r S \alpha_2 w_{T,B} p_{T,A,0} p_{T,B,0} + r p_{T,B,0}^2 - r S p_{T,B,0}^2 - r w_{t,B} p_{T,B,0}^2 +$$
$$r S w_{t,B} p_{T,B,0}^2 - r w_{T,A} p_{T,B,0}^2 + r S w_{T,A} p_{T,B,0}^2 + r w_{T,B} p_{T,B,0}^2 - r S w_{T,B} p_{T,B,0}^2)^2 +$$
$$(y w_{T,B} (\alpha_2 p_{T,A,0} - r \alpha_2 p_{T,A,0} + r S \alpha_2 p_{T,A,0} + r p_{t,B,0} p_{T,A,0} - r S p_{t,B,0} p_{T,A,0} - r \beta_1 p_{t,B,0} p_{T,A,0} +$$
$$r S \beta_1 p_{t,B,0} p_{T,A,0} + r \alpha_2 p_{T,A,0}^2 - r S \alpha_2 p_{T,A,0}^2 + p_{T,B,0} - r p_{T,B,0} + r S p_{T,B,0} +$$
$$r p_{t,B,0} p_{T,B,0} - r S p_{t,B,0} p_{T,B,0} - r \beta_1 p_{t,B,0} p_{T,B,0} + r S \beta_1 p_{t,B,0} p_{T,B,0} + r p_{T,A,0} p_{T,B,0} -$$
$$r S p_{T,A,0} p_{T,B,0} + r \alpha_2 p_{T,A,0} p_{T,B,0} - r S \alpha_2 p_{T,A,0} p_{T,B,0} + r p_{T,B,0}^2 - r S p_{T,B,0}^2) +$$
$$w_{t,B} (2 (-1 + S) (-1 + \beta_1) p_{t,B,0} p_{t,B,1} - \alpha_1 (-1 + p_{t,B,0} + p_{T,A,0} + p_{T,B,0})$$
$$(1 - r p_{T,A,0} + r S p_{T,A,0} - r p_{T,B,0} + r S p_{T,B,0}) +$$
$$p_{t,B,1} (1 - \beta_1 - p_{t,B,0} + S p_{t,B,0} + \beta_1 p_{t,B,0} - S \beta_1 p_{t,B,0} - r p_{T,A,0} + r S p_{T,A,0} + r$$
$$\alpha_2 p_{T,A,0} - r S \alpha_2 p_{T,A,0} + r \beta_1 p_{T,A,0} - r S \beta_1 p_{T,A,0} + r \beta_1 p_{T,B,0} - r S \beta_1 p_{T,B,0}) +$$
$$z (-((-1 + S) (-1 + \beta_1) p_{t,B,0}^2) - r (-1 + S) (-1 + p_{t,B,0} + p_{T,A,0} + p_{T,B,0}) (\alpha_2 p_{T,A,0} + p_{T,B,0}) +$$
$$p_{t,B,0} (-1 + \beta_1 + p_{t,B,0} - S p_{t,B,0} - \beta_1 p_{t,B,0} + S \beta_1 p_{t,B,0} + r p_{T,A,0} - r S p_{T,A,0} -$$
$$r \alpha_2 p_{T,A,0} + r S \alpha_2 p_{T,A,0} - r \beta_1 p_{T,A,0} + r S \beta_1 p_{T,A,0} - r \beta_1 p_{T,B,0} + r S \beta_1 p_{T,B,0})) +$$
$$p_{t,B,0} (-p_{t,B,1} + S p_{t,B,1} + \beta_1 p_{t,B,1} - S \beta_1 p_{t,B,1} - r p_{T,A,1} + r S p_{T,A,1} + r \alpha_2$$
$$p_{T,A,1} - r S \alpha_2 p_{T,A,1} + r \beta_1 p_{T,A,1} - r S \beta_1 p_{T,A,1} - r \beta_2 p_{T,B,0} + r S \beta_2 p_{T,B,0} + r \beta_1$$
$$p_{T,B,1} - r S \beta_1 p_{T,B,1}) - r (-1 + S) ((\alpha_2 p_{T,A,0} + p_{T,B,0}) (-p_{t,B,1} - p_{T,A,1} - p_{T,B,1}) +$$
$$(1 - p_{t,B,0} - p_{T,A,0} - p_{T,B,0}) (\alpha_2 p_{T,A,1} - \beta_2 p_{T,B,0} + p_{T,B,1})))) /$$
$$(1 - p_{t,B,0} + \beta_1 p_{t,B,0} + w_{t,B} p_{t,B,0} - \beta_1 w_{t,B} p_{t,B,0} - p_{T,A,0} - r \alpha_2 p_{T,A,0} + r S \alpha_2 p_{T,A,0} +$$
$$r \alpha_2 w_{t,B} p_{T,A,0} - r S \alpha_2 w_{t,B} p_{T,A,0} + w_{T,A} p_{T,A,0} - \alpha_2 w_{T,A} p_{T,A,0} +$$
$$r \alpha_2 w_{T,A} p_{T,A,0} - r S \alpha_2 w_{T,A} p_{T,A,0} + \alpha_2 w_{T,B} p_{T,A,0} - r \alpha_2 w_{T,B} p_{T,A,0} +$$
$$r S \alpha_2 w_{T,B} p_{T,A,0} + r p_{t,B,0} p_{T,A,0} - r S p_{t,B,0} p_{T,A,0} -$$
$$r \beta_1 p_{t,B,0} p_{T,A,0} + r S \beta_1 p_{t,B,0} p_{T,A,0} - r w_{t,B} p_{t,B,0} p_{T,A,0} +$$
$$r S w_{t,B} p_{t,B,0} p_{T,A,0} + r \beta_1 w_{t,B} p_{t,B,0} p_{T,A,0} - r S \beta_1 w_{t,B} p_{t,B,0} p_{T,A,0} -$$
$$r w_{T,A} p_{t,B,0} p_{T,A,0} + r S w_{T,A} p_{t,B,0} p_{T,A,0} + r \beta_1 w_{T,A} p_{t,B,0} p_{T,A,0} -$$
$$r S \beta_1 w_{T,A} p_{t,B,0} p_{T,A,0} + r w_{T,B} p_{t,B,0} p_{T,A,0} - r S w_{T,B} p_{t,B,0} p_{T,A,0} -$$
$$r \beta_1 w_{T,B} p_{t,B,0} p_{T,A,0} + r S \beta_1 w_{T,B} p_{t,B,0} p_{T,A,0} + r \alpha_2 p_{T,A,0}^2 -$$
$$r S \alpha_2 p_{T,A,0}^2 - r \alpha_2 w_{t,B} p_{T,A,0}^2 + r S \alpha_2 w_{t,B} p_{T,A,0}^2 - r \alpha_2 w_{T,A} p_{T,A,0}^2 +$$
$$r S \alpha_2 w_{T,A} p_{T,A,0}^2 + r \alpha_2 w_{T,B} p_{T,A,0}^2 - r S \alpha_2 w_{T,B} p_{T,A,0}^2 - p_{T,B,0} -$$
$$r p_{T,B,0} + r S p_{T,B,0} + r w_{t,B} p_{T,B,0} - r S w_{t,B} p_{T,B,0} + r w_{T,A} p_{T,B,0} -$$
$$r S w_{T,A} p_{T,B,0} + w_{T,B} p_{T,B,0} - r w_{T,B} p_{T,B,0} + r S w_{T,B} p_{T,B,0} +$$
$$r p_{t,B,0} p_{T,B,0} - r S p_{t,B,0} p_{T,B,0} - r \beta_1 p_{t,B,0} p_{T,B,0} +$$
$$r S \beta_1 p_{t,B,0} p_{T,B,0} - r w_{t,B} p_{t,B,0} p_{T,B,0} + r S w_{t,B} p_{t,B,0} p_{T,B,0} +$$

$$r \beta_1 w_{t,B} p_{t,B,0} p_{T,B,0} - r S \beta_1 w_{t,B} p_{t,B,0} p_{T,B,0} - r w_{T,A} p_{t,B,0} p_{T,B,0} +$$
$$r S w_{T,A} p_{t,B,0} p_{T,B,0} + r \beta_1 w_{T,A} p_{t,B,0} p_{T,B,0} - r S \beta_1 w_{T,A} p_{t,B,0} p_{T,B,0} +$$
$$r w_{T,B} p_{t,B,0} p_{T,B,0} - r S w_{T,B} p_{t,B,0} p_{T,B,0} - r \beta_1 w_{T,B} p_{t,B,0} p_{T,B,0} +$$
$$r S \beta_1 w_{T,B} p_{t,B,0} p_{T,B,0} + r p_{T,A,0} p_{T,B,0} - r S p_{T,A,0} p_{T,B,0} +$$
$$r \alpha_2 p_{T,A,0} p_{T,B,0} - r S \alpha_2 p_{T,A,0} p_{T,B,0} - r w_{t,B} p_{T,A,0} p_{T,B,0} +$$
$$r S w_{t,B} p_{T,A,0} p_{T,B,0} - r \alpha_2 w_{t,B} p_{T,A,0} p_{T,B,0} + r S \alpha_2 w_{t,B} p_{T,A,0} p_{T,B,0} -$$
$$r w_{T,A} p_{T,A,0} p_{T,B,0} + r S w_{T,A} p_{T,A,0} p_{T,B,0} - r \alpha_2 w_{T,A} p_{T,A,0} p_{T,B,0} +$$
$$r S \alpha_2 w_{T,A} p_{T,A,0} p_{T,B,0} + r w_{T,B} p_{T,A,0} p_{T,B,0} - r S w_{T,B} p_{T,A,0} p_{T,B,0} +$$
$$r \alpha_2 w_{T,B} p_{T,A,0} p_{T,B,0} - r S \alpha_2 w_{T,B} p_{T,A,0} p_{T,B,0} + r p^2_{T,B,0} -$$
$$r S p^2_{T,B,0} - r w_{t,B} p^2_{T,B,0} + r S w_{t,B} p^2_{T,B,0} - r w_{T,A} p^2_{T,B,0} +$$
$$r S w_{T,A} p^2_{T,B,0} + r w_{T,B} p^2_{T,B,0} - r S w_{T,B} p^2_{T,B,0})) == 0,$$
$$(-p_{T,A,1} - (w_{T,A} ((-1 + S) (-1 + \alpha_2) p^2_{T,A,0} - r (-1 + S) (1 - p_{t,B,0} + \beta_1 p_{t,B,0} - p_{T,A,0} - p_{T,B,0}) p_{T,B,0} +$$
$$p_{T,A,0} (1 - \alpha_2 + r \alpha_2 - r S \alpha_2 - r p_{t,B,0} + r S p_{t,B,0} + r \beta_1 p_{t,B,0} - r S \beta_1 p_{t,B,0} - p_{T,A,0} +$$
$$S p_{T,A,0} + \alpha_2 p_{T,A,0} - r \alpha_2 p_{T,A,0} - S \alpha_2 p_{T,A,0} + r S \alpha_2 p_{T,A,0} - r \alpha_2 p_{T,B,0} + r S \alpha_2 p_{T,B,0}))$$
$$(S w_{T,A} p_{T,A,1} - S \alpha_2 w_{T,A} p_{T,A,1} + S \alpha_2 w_{T,B} p_{T,A,1} + 2 w_{T,A} p_{T,A,0} p_{T,A,1} -$$
$$2 S w_{T,A} p_{T,A,0} p_{T,A,1} - 2 \alpha_2 w_{T,A} p_{T,A,0} p_{T,A,1} + 2 S \alpha_2 w_{T,A} p_{T,A,0} p_{T,A,1} +$$
$$2 \alpha_2 w_{T,B} p_{T,A,0} p_{T,A,1} - 2 S \alpha_2 w_{T,B} p_{T,A,0} p_{T,A,1} + w_{T,A} (p_{t,B,1} p_{T,A,0} + p_{t,B,0} p_{T,A,1}) -$$
$$r w_{T,A} (p_{t,B,1} p_{T,A,0} + p_{t,B,0} p_{T,A,1}) - S w_{T,A} (p_{t,B,1} p_{T,A,0} + p_{t,B,0} p_{T,A,1}) +$$
$$r S w_{T,A} (p_{t,B,1} p_{T,A,0} + p_{t,B,0} p_{T,A,1}) - \alpha_2 w_{T,A} (p_{t,B,1} p_{T,A,0} + p_{t,B,0} p_{T,A,1}) +$$
$$r \alpha_2 w_{T,A} (p_{t,B,1} p_{T,A,0} + p_{t,B,0} p_{T,A,1}) + S \alpha_2 w_{T,A} (p_{t,B,1} p_{T,A,0} + p_{t,B,0} p_{T,A,1}) -$$
$$r S \alpha_2 w_{T,A} (p_{t,B,1} p_{T,A,0} + p_{t,B,0} p_{T,A,1}) + r \beta_1 w_{T,A} (p_{t,B,1} p_{T,A,0} + p_{t,B,0} p_{T,A,1}) -$$
$$r S \beta_1 w_{T,A} (p_{t,B,1} p_{T,A,0} + p_{t,B,0} p_{T,A,1}) + r w_{T,B} (p_{t,B,1} p_{T,A,0} + p_{t,B,0} p_{T,A,1}) -$$
$$r S w_{T,B} (p_{t,B,1} p_{T,A,0} + p_{t,B,0} p_{T,A,1}) + \alpha_2 w_{T,B} (p_{t,B,1} p_{T,A,0} + p_{t,B,0} p_{T,A,1}) -$$
$$r \alpha_2 w_{T,B} (p_{t,B,1} p_{T,A,0} + p_{t,B,0} p_{T,A,1}) - S \alpha_2 w_{T,B} (p_{t,B,1} p_{T,A,0} + p_{t,B,0} p_{T,A,1}) +$$
$$r S \alpha_2 w_{T,B} (p_{t,B,1} p_{T,A,0} + p_{t,B,0} p_{T,A,1}) - r \beta_1 w_{T,B} (p_{t,B,1} p_{T,A,0} + p_{t,B,0} p_{T,A,1}) +$$
$$r S \beta_1 w_{T,B} (p_{t,B,1} p_{T,A,0} + p_{t,B,0} p_{T,A,1}) + S \beta_2 w_{T,A} p_{T,B,0} - S \beta_2 w_{T,B} p_{T,B,0} +$$
$$\beta_2 w_{T,A} p_{t,B,0} p_{T,B,0} - r \beta_2 w_{T,A} p_{t,B,0} p_{T,B,0} - S \beta_2 w_{T,A} p_{t,B,0} p_{T,B,0} +$$
$$r S \beta_2 w_{T,A} p_{t,B,0} p_{T,B,0} - \beta_2 w_{T,B} p_{t,B,0} p_{T,B,0} + r \beta_2 w_{T,B} p_{t,B,0} p_{T,B,0} +$$
$$S \beta_2 w_{T,B} p_{t,B,0} p_{T,B,0} - r S \beta_2 w_{T,B} p_{t,B,0} p_{T,B,0} + \beta_2 w_{T,A} p_{T,A,0} p_{T,B,0} -$$
$$S \beta_2 w_{T,A} p_{T,A,0} p_{T,B,0} - \beta_2 w_{T,B} p_{T,A,0} p_{T,B,0} + S \beta_2 w_{T,B} p_{T,A,0} p_{T,B,0} +$$
$$\beta_2 w_{T,A} p^2_{T,B,0} - S \beta_2 w_{T,A} p^2_{T,B,0} - \beta_2 w_{T,B} p^2_{T,B,0} + S \beta_2 w_{T,B} p^2_{T,B,0} + r \alpha_1 w_{T,A} p_{T,A,0}$$
$$(-1 + p_{t,B,0} + p_{T,A,0} + p_{T,B,0}) - r S \alpha_1 w_{T,A} p_{T,A,0} (-1 + p_{t,B,0} + p_{T,A,0} + p_{T,B,0}) -$$
$$r \alpha_1 w_{T,B} p_{T,A,0} (-1 + p_{t,B,0} + p_{T,A,0} + p_{T,B,0}) + r S \alpha_1 w_{T,B} p_{T,A,0}$$
$$(-1 + p_{t,B,0} + p_{T,A,0} + p_{T,B,0}) + r \alpha_1 w_{T,A} p_{T,B,0} (-1 + p_{t,B,0} + p_{T,A,0} + p_{T,B,0}) -$$
$$r S \alpha_1 w_{T,A} p_{T,B,0} (-1 + p_{t,B,0} + p_{T,A,0} + p_{T,B,0}) - \beta_2 w_{T,A} p_{T,B,0} (-1 + p_{t,B,0} + p_{T,A,0} + p_{T,B,0}) +$$
$$r \beta_2 w_{T,A} p_{T,B,0} (-1 + p_{t,B,0} + p_{T,A,0} + p_{T,B,0}) + S \beta_2 w_{T,A} p_{T,B,0} (-1 + p_{t,B,0} + p_{T,A,0} + p_{T,B,0}) -$$
$$r S \beta_2 w_{T,A} p_{T,B,0} (-1 + p_{t,B,0} + p_{T,A,0} + p_{T,B,0}) - r \alpha_1 w_{T,B} p_{T,B,0} (-1 + p_{t,B,0} + p_{T,A,0} + p_{T,B,0}) +$$
$$r S \alpha_1 w_{T,B} p_{T,B,0} (-1 + p_{t,B,0} + p_{T,A,0} + p_{T,B,0}) + \beta_2 w_{T,B} p_{T,B,0} (-1 + p_{t,B,0} + p_{T,A,0} + p_{T,B,0}) -$$
$$r \beta_2 w_{T,B} p_{T,B,0} (-1 + p_{t,B,0} + p_{T,A,0} + p_{T,B,0}) - S \beta_2 w_{T,B} p_{T,B,0} (-1 + p_{t,B,0} + p_{T,A,0} + p_{T,B,0}) +$$
$$r S \beta_2 w_{T,B} p_{T,B,0} (-1 + p_{t,B,0} + p_{T,A,0} + p_{T,B,0}) + p_{t,B,1} (r (-1 + S) (-1 + \alpha_2) p_{T,A,0} +$$

$$\beta_1 \left(S-(-1+S)\, p_{t,B,0}+(-1+r)(-1+S)\, p_{T,A,0}+p_{T,B,0}-r\, p_{T,B,0}-S\, p_{T,B,0}+r\, S\, p_{T,B,0}\right)\Big)+$$
$$S\, w_{T,A} \left(-p_{T,A,1}\left(1-p_{t,B,0}-p_{T,A,0}-p_{T,B,0}\right)-p_{T,A,0}\left(-p_{t,B,1}-p_{T,A,1}-p_{T,B,1}\right)\right)+$$
$$\alpha_2\, w_{T,A} \left(-p_{T,A,1}\left(1-p_{t,B,0}-p_{T,A,0}-p_{T,B,0}\right)-p_{T,A,0}\left(-p_{t,B,1}-p_{T,A,1}-p_{T,B,1}\right)\right)+$$
$$r\, S\, \alpha_2\, w_{T,A} \left(-p_{T,A,1}\left(1-p_{t,B,0}-p_{T,A,0}-p_{T,B,0}\right)-p_{T,A,0}\left(-p_{t,B,1}-p_{T,A,1}-p_{T,B,1}\right)\right)+$$
$$r\, \alpha_2\, w_{T,B} \left(-p_{T,A,1}\left(1-p_{t,B,0}-p_{T,A,0}-p_{T,B,0}\right)-p_{T,A,0}\left(-p_{t,B,1}-p_{T,A,1}-p_{T,B,1}\right)\right)+$$
$$S\, \alpha_2\, w_{T,B} \left(-p_{T,A,1}\left(1-p_{t,B,0}-p_{T,A,0}-p_{T,B,0}\right)-p_{T,A,0}\left(-p_{t,B,1}-p_{T,A,1}-p_{T,B,1}\right)\right)+$$
$$w_{T,A} \left(p_{T,A,1}\left(1-p_{t,B,0}-p_{T,A,0}-p_{T,B,0}\right)+p_{T,A,0}\left(-p_{t,B,1}-p_{T,A,1}-p_{T,B,1}\right)\right)+$$
$$r\, \alpha_2\, w_{T,A} \left(p_{T,A,1}\left(1-p_{t,B,0}-p_{T,A,0}-p_{T,B,0}\right)+p_{T,A,0}\left(-p_{t,B,1}-p_{T,A,1}-p_{T,B,1}\right)\right)+$$
$$S\, \alpha_2\, w_{T,A} \left(p_{T,A,1}\left(1-p_{t,B,0}-p_{T,A,0}-p_{T,B,0}\right)+p_{T,A,0}\left(-p_{t,B,1}-p_{T,A,1}-p_{T,B,1}\right)\right)+$$
$$\alpha_2\, w_{T,B} \left(p_{T,A,1}\left(1-p_{t,B,0}-p_{T,A,0}-p_{T,B,0}\right)+p_{T,A,0}\left(-p_{t,B,1}-p_{T,A,1}-p_{T,B,1}\right)\right)+$$
$$r\, S\, \alpha_2\, w_{T,B} \left(p_{T,A,1}\left(1-p_{t,B,0}-p_{T,A,0}-p_{T,B,0}\right)+p_{T,A,0}\left(-p_{t,B,1}-p_{T,A,1}-p_{T,B,1}\right)\right)+$$
$$\Big(S+p_{t,B,0}-S\, p_{t,B,0}+\beta_1\, p_{t,B,0}-S\, \beta_1\, p_{t,B,0}+p_{T,A,0}-S\, p_{T,A,0}-r\, \alpha_2\, p_{T,A,0}+r\, S\, \alpha_2\, p_{T,A,0}+$$
$$p_{T,B,0}-r\, p_{T,B,0}-S\, p_{T,B,0}+r\, S\, p_{T,B,0}\Big)\left(-p_{t,B,1}-p_{T,A,1}-p_{T,B,1}\right)+S\, w_{T,B}\, p_{T,B,1}+$$
$$2\, w_{T,B}\, p_{T,B,0}\, p_{T,B,1}-2\, S\, w_{T,B}\, p_{T,B,0}\, p_{T,B,1}+\left(1-p_{t,B,0}-p_{T,A,0}-p_{T,B,0}\right)$$
$$\Big(p_{t,B,1}-S\, p_{t,B,1}+\beta_1\, p_{t,B,1}-S\, \beta_1\, p_{t,B,1}+p_{T,A,1}-S\, p_{T,A,1}-r\, \alpha_2\, p_{T,A,1}+r\, S\, \alpha_2\, p_{T,A,1}+$$
$$r\, \beta_2\, p_{T,B,0}-r\, S\, \beta_2\, p_{T,B,0}+\alpha_1 \Big(-S+(-1+S)\, p_{t,B,0}-(-1+r)(-1+S)\, p_{T,A,0}-p_{T,B,0}+$$
$$r\, p_{T,B,0}+S\, p_{T,B,0}-r\, S\, p_{T,B,0}\Big)+p_{T,B,1}-r\, p_{T,B,1}-S\, p_{T,B,1}+r\, S\, p_{T,B,1}\Big)+$$
$$r\, \beta_1\, w_{T,A}\left(p_{t,B,1}\, p_{T,B,0}+p_{t,B,0}\, p_{T,B,1}\right)-r\, S\, \beta_1\, w_{T,A}\left(p_{t,B,1}\, p_{T,B,0}+p_{t,B,0}\, p_{T,B,1}\right)+$$
$$w_{T,B}\left(p_{t,B,1}\, p_{T,B,0}+p_{t,B,0}\, p_{T,B,1}\right)-S\, w_{T,B}\left(p_{t,B,1}\, p_{T,B,0}+p_{t,B,0}\, p_{T,B,1}\right)-$$
$$r\, \beta_1\, w_{T,B}\left(p_{t,B,1}\, p_{T,B,0}+p_{t,B,0}\, p_{T,B,1}\right)+r\, S\, \beta_1\, w_{T,B}\left(p_{t,B,1}\, p_{T,B,0}+p_{t,B,0}\, p_{T,B,1}\right)+$$
$$w_{T,A}\left(p_{T,A,1}\, p_{T,B,0}+p_{T,A,0}\, p_{T,B,1}\right)-S\, w_{T,A}\left(p_{T,A,1}\, p_{T,B,0}+p_{T,A,0}\, p_{T,B,1}\right)-$$
$$\alpha_2\, w_{T,A}\left(p_{T,A,1}\, p_{T,B,0}+p_{T,A,0}\, p_{T,B,1}\right)+S\, \alpha_2\, w_{T,A}\left(p_{T,A,1}\, p_{T,B,0}+p_{T,A,0}\, p_{T,B,1}\right)+$$
$$w_{T,B}\left(p_{T,A,1}\, p_{T,B,0}+p_{T,A,0}\, p_{T,B,1}\right)-S\, w_{T,B}\left(p_{T,A,1}\, p_{T,B,0}+p_{T,A,0}\, p_{T,B,1}\right)+$$
$$\alpha_2\, w_{T,B}\left(p_{T,A,1}\, p_{T,B,0}+p_{T,A,0}\, p_{T,B,1}\right)-S\, \alpha_2\, w_{T,B}\left(p_{T,A,1}\, p_{T,B,0}+p_{T,A,0}\, p_{T,B,1}\right)+$$
$$r\, S\, w_{T,A}\left(-p_{T,B,0}\left(-p_{t,B,1}-p_{T,A,1}-p_{T,B,1}\right)-\left(1-p_{t,B,0}-p_{T,A,0}-p_{T,B,0}\right)p_{T,B,1}\right)+$$
$$r\, w_{T,B}\left(-p_{T,B,0}\left(-p_{t,B,1}-p_{T,A,1}-p_{T,B,1}\right)-\left(1-p_{t,B,0}-p_{T,A,0}-p_{T,B,0}\right)p_{T,B,1}\right)+$$
$$S\, w_{T,B}\left(-p_{T,B,0}\left(-p_{t,B,1}-p_{T,A,1}-p_{T,B,1}\right)-\left(1-p_{t,B,0}-p_{T,A,0}-p_{T,B,0}\right)p_{T,B,1}\right)+$$
$$r\, w_{T,A}\left(p_{T,B,0}\left(-p_{t,B,1}-p_{T,A,1}-p_{T,B,1}\right)+\left(1-p_{t,B,0}-p_{T,A,0}-p_{T,B,0}\right)p_{T,B,1}\right)+$$
$$w_{T,B}\left(p_{T,B,0}\left(-p_{t,B,1}-p_{T,A,1}-p_{T,B,1}\right)+\left(1-p_{t,B,0}-p_{T,A,0}-p_{T,B,0}\right)p_{T,B,1}\right)+$$
$$r\, S\, w_{T,B}\left(p_{T,B,0}\left(-p_{t,B,1}-p_{T,A,1}-p_{T,B,1}\right)+\left(1-p_{t,B,0}-p_{T,A,0}-p_{T,B,0}\right)p_{T,B,1}\right)+(-1+S)$$
$$\left(\alpha_1\left(1-p_{t,B,0}-p_{T,A,0}-p_{T,B,0}\right)^2-2\left(-1+p_{t,B,0}+p_{T,A,0}+p_{T,B,0}\right)\left(p_{t,B,1}+p_{T,A,1}+p_{T,B,1}\right)\right)+$$
$$p_{t,B,0}\Big(r\,(-1+S)\left((-1+\alpha_2)\, p_{T,A,1}-\beta_2\, p_{T,B,0}\right)+\beta_1\Big(-\left((-1+S)\, p_{t,B,1}\right)+(-1+r)(-1+S)\, p_{T,A,1}+$$
$$p_{T,B,1}-r\, p_{T,B,1}-S\, p_{T,B,1}+r\, S\, p_{T,B,1}\Big)\Big)+w_{t,B}\Big(2\,(-1+S)(-1+\beta_1)\, p_{t,B,0}\, p_{t,B,1}-$$
$$\alpha_1\left(-1+p_{t,B,0}+p_{T,A,0}+p_{T,B,0}\right)\left(1-r\, p_{T,A,0}+r\, S\, p_{T,A,0}-r\, p_{T,B,0}+r\, S\, p_{T,B,0}\right)+$$
$$p_{t,B,1}\Big(1-\beta_1-p_{t,B,0}+S\, p_{t,B,0}+\beta_1\, p_{t,B,0}-S\, \beta_1\, p_{t,B,0}-r\, p_{T,A,0}+r\, S\, p_{T,A,0}+$$
$$r\, \alpha_2\, p_{T,A,0}-r\, S\, \alpha_2\, p_{T,A,0}+r\, \beta_1\, p_{T,A,0}-r\, S\, \beta_1\, p_{T,A,0}+r\, \beta_1\, p_{T,B,0}-r\, S\, \beta_1\, p_{T,B,0}\Big)+$$
$$p_{t,B,0}\Big(-p_{t,B,1}+S\, p_{t,B,1}+\beta_1\, p_{t,B,1}-S\, \beta_1\, p_{t,B,1}-r\, p_{T,A,1}+r\, S\, p_{T,A,1}+$$
$$r\, \alpha_2\, p_{T,A,1}-r\, S\, \alpha_2\, p_{T,A,1}+r\, \beta_1\, p_{T,A,1}-r\, S\, \beta_1\, p_{T,A,1}-r\, \beta_2\, p_{T,B,0}+r\, S\, \beta_2\, p_{T,B,0}+$$
$$r\, \beta_1\, p_{T,B,1}-r\, S\, \beta_1\, p_{T,B,1}\Big)+r\,(-1+S)\Big(-\Big(\left(\alpha_2\, p_{T,A,0}+p_{T,B,0}\right)\left(-p_{t,B,1}-p_{T,A,1}-p_{T,B,1}\right)\Big)-$$

$$(1 - p_{t,B,0} - p_{T,A,0} - p_{T,B,0}) (\alpha_2 p_{T,A,1} - \beta_2 p_{T,B,0} + p_{T,B,1}))))) /$$

$$(1 - p_{t,B,0} + \beta_1 p_{t,B,0} + w_{t,B} p_{t,B,0} - \beta_1 w_{t,B} p_{t,B,0} - p_{T,A,0} - r \alpha_2 p_{T,A,0} + r S \alpha_2 p_{T,A,0} +$$

$$r \alpha_2 w_{t,B} p_{T,A,0} - r S \alpha_2 w_{t,B} p_{T,A,0} + w_{T,A} p_{T,A,0} - \alpha_2 w_{T,A} p_{T,A,0} + r \alpha_2 w_{T,A} p_{T,A,0} -$$

$$r S \alpha_2 w_{T,A} p_{T,A,0} + \alpha_2 w_{T,B} p_{T,A,0} - r \alpha_2 w_{T,B} p_{T,A,0} + r S \alpha_2 w_{T,B} p_{T,A,0} + r p_{t,B,0} p_{T,A,0} -$$

$$r S p_{t,B,0} p_{T,A,0} - r \beta_1 p_{t,B,0} p_{T,A,0} + r S \beta_1 p_{t,B,0} p_{T,A,0} - r w_{t,B} p_{t,B,0} p_{T,A,0} +$$

$$r S w_{t,B} p_{t,B,0} p_{T,A,0} + r \beta_1 w_{t,B} p_{t,B,0} p_{T,A,0} - r S \beta_1 w_{t,B} p_{t,B,0} p_{T,A,0} -$$

$$r w_{T,A} p_{t,B,0} p_{T,A,0} + r S w_{T,A} p_{t,B,0} p_{T,A,0} + r \beta_1 w_{T,A} p_{t,B,0} p_{T,A,0} -$$

$$r S \beta_1 w_{T,A} p_{t,B,0} p_{T,A,0} + r w_{T,B} p_{t,B,0} p_{T,A,0} - r S w_{T,B} p_{t,B,0} p_{T,A,0} -$$

$$r \beta_1 w_{T,B} p_{t,B,0} p_{T,A,0} + r S \beta_1 w_{T,B} p_{t,B,0} p_{T,A,0} + r \alpha_2 p_{T,A,0}^2 - r S \alpha_2 p_{T,A,0}^2 -$$

$$r \alpha_2 w_{t,B} p_{T,A,0}^2 + r S \alpha_2 w_{t,B} p_{T,A,0}^2 - r \alpha_2 w_{T,A} p_{T,A,0}^2 + r S \alpha_2 w_{T,A} p_{T,A,0}^2 +$$

$$r \alpha_2 w_{T,B} p_{T,A,0}^2 - r S \alpha_2 w_{T,B} p_{T,A,0}^2 - p_{T,B,0} - r p_{T,B,0} + r S p_{T,B,0} + r w_{t,B} p_{T,B,0} -$$

$$r S w_{t,B} p_{T,B,0} + r w_{T,A} p_{T,B,0} - r S w_{T,A} p_{T,B,0} + w_{T,B} p_{T,B,0} - r w_{T,B} p_{T,B,0} +$$

$$r S w_{T,B} p_{T,B,0} + r p_{t,B,0} p_{T,B,0} - r S p_{t,B,0} p_{T,B,0} - r \beta_1 p_{t,B,0} p_{T,B,0} +$$

$$r S \beta_1 p_{t,B,0} p_{T,B,0} - r w_{t,B} p_{t,B,0} p_{T,B,0} + r S w_{t,B} p_{t,B,0} p_{T,B,0} + r \beta_1 w_{t,B} p_{t,B,0} p_{T,B,0} -$$

$$r S \beta_1 w_{t,B} p_{t,B,0} p_{T,B,0} - r w_{T,A} p_{t,B,0} p_{T,B,0} + r S w_{T,A} p_{t,B,0} p_{T,B,0} +$$

$$r \beta_1 w_{T,A} p_{t,B,0} p_{T,B,0} - r S \beta_1 w_{T,A} p_{t,B,0} p_{T,B,0} + r w_{T,B} p_{t,B,0} p_{T,B,0} -$$

$$r S w_{T,B} p_{t,B,0} p_{T,B,0} - r \beta_1 w_{T,B} p_{t,B,0} p_{T,B,0} + r S \beta_1 w_{T,B} p_{t,B,0} p_{T,B,0} + r p_{T,A,0} p_{T,B,0} -$$

$$r S p_{T,A,0} p_{T,B,0} + r \alpha_2 p_{T,A,0} p_{T,B,0} - r S \alpha_2 p_{T,A,0} p_{T,B,0} - r w_{t,B} p_{T,A,0} p_{T,B,0} +$$

$$r S w_{t,B} p_{T,A,0} p_{T,B,0} - r \alpha_2 w_{t,B} p_{T,A,0} p_{T,B,0} + r S \alpha_2 w_{t,B} p_{T,A,0} p_{T,B,0} -$$

$$r w_{T,A} p_{T,A,0} p_{T,B,0} + r S w_{T,A} p_{T,A,0} p_{T,B,0} - r \alpha_2 w_{T,A} p_{T,A,0} p_{T,B,0} +$$

$$r S \alpha_2 w_{T,A} p_{T,A,0} p_{T,B,0} + r w_{T,B} p_{T,A,0} p_{T,B,0} - r S w_{T,B} p_{T,A,0} p_{T,B,0} +$$

$$r \alpha_2 w_{T,B} p_{T,A,0} p_{T,B,0} - r S \alpha_2 w_{T,B} p_{T,A,0} p_{T,B,0} + r p_{T,B,0}^2 - r S p_{T,B,0}^2 - r w_{t,B} p_{T,B,0}^2 +$$

$$r S w_{t,B} p_{T,B,0}^2 - r w_{T,A} p_{T,B,0}^2 + r S w_{T,A} p_{T,B,0}^2 + r w_{T,B} p_{T,B,0}^2 - r S w_{T,B} p_{T,B,0}^2)^2 +$$

$$(z (-((-1 + S) (-1 + p_{t,B,0} + p_{T,A,0} + p_{T,B,0})^2) + (-1 + p_{t,B,0} + p_{T,A,0} + p_{T,B,0})$$

$$(-S - p_{t,B,0} + S p_{t,B,0} - \beta_1 p_{t,B,0} + S \beta_1 p_{t,B,0} - p_{T,A,0} + S p_{T,A,0} + r \alpha_2 p_{T,A,0} - r S \alpha_2$$

$$p_{T,A,0} - p_{T,B,0} + r p_{T,B,0} + S p_{T,B,0} - r S p_{T,B,0}) + p_{t,B,0} (r (-1 + S) (-1 + \alpha_2) p_{T,A,0} + \beta_1$$

$$(S - (-1 + S) p_{t,B,0} + (-1 + r) (-1 + S) p_{T,A,0} + p_{T,B,0} - r p_{T,B,0} - S p_{T,B,0} + r S p_{T,B,0}))) +$$

$$w_{T,A} (2 (-1 + S) (-1 + \alpha_2) p_{T,A,0} p_{T,A,1} + p_{T,A,1} (1 - \alpha_2 + r \alpha_2 - r S \alpha_2 - r p_{t,B,0} + r S$$

$$p_{t,B,0} + r \beta_1 p_{t,B,0} - r S \beta_1 p_{t,B,0} - p_{T,A,0} + S p_{T,A,0} + \alpha_2 p_{T,A,0} - r \alpha_2 p_{T,A,0} - S \alpha_2$$

$$p_{T,A,0} + r S \alpha_2 p_{T,A,0} - r \alpha_2 p_{T,B,0} + r S \alpha_2 p_{T,B,0}) + y (-((-1 + S) (-1 + \alpha_2) p_{T,A,0}^2) + r$$

$$(-1 + S) (1 - p_{t,B,0} + \beta_1 p_{t,B,0} - p_{T,A,0} - p_{T,B,0}) p_{T,B,0} - p_{T,A,0} (1 - \alpha_2 + r \alpha_2 - r S \alpha_2 -$$

$$r p_{t,B,0} + r S p_{t,B,0} + r \beta_1 p_{t,B,0} - r S \beta_1 p_{t,B,0} - p_{T,A,0} + S p_{T,A,0} + \alpha_2 p_{T,A,0} -$$

$$r \alpha_2 p_{T,A,0} - S \alpha_2 p_{T,A,0} + r S \alpha_2 p_{T,A,0} - r \alpha_2 p_{T,B,0} + r S \alpha_2 p_{T,B,0})) - p_{T,B,0} (\beta_2 (-S -$$

$$(-1 + r) (-1 + S) p_{t,B,0} + (-1 + S) p_{T,B,0} + (-1 + r) (-1 + S) (-1 + p_{t,B,0} + p_{T,A,0} + p_{T,B,0})) +$$

$$r (-1 + S) (-p_{t,B,1} + \beta_1 p_{t,B,1} - p_{T,A,1} - \alpha_1 (1 - p_{t,B,0} - p_{T,A,0} - p_{T,B,0}) - p_{T,B,1})) -$$

$$r (-1 + S) (1 - p_{t,B,0} + \beta_1 p_{t,B,0} - p_{T,A,0} - p_{T,B,0}) p_{T,B,1} +$$

$$p_{T,A,0} (-r p_{t,B,1} - S p_{t,B,1} + r S p_{t,B,1} + r \beta_1 p_{t,B,1} - r S \beta_1 p_{t,B,1} - p_{T,A,1} + \beta_2$$

$$p_{T,B,0} - S \beta_2 p_{T,B,0} + r \alpha_1 (-1 + p_{t,B,0} + p_{T,A,0} + p_{T,B,0}) - r S \alpha_1$$

$$(-1 + p_{t,B,0} + p_{T,A,0} + p_{T,B,0}) - S p_{T,B,1} + S (p_{t,B,1} + p_{T,A,1} + p_{T,B,1}) + \alpha_2$$

$$(p_{T,A,1} - r p_{T,A,1} - S p_{T,A,1} + r S p_{T,A,1} - r p_{T,B,1} + r S p_{T,B,1})))) /$$

$$(1 - p_{t,B,0} + \beta_1 p_{t,B,0} + w_{t,B} p_{t,B,0} - \beta_1 w_{t,B} p_{t,B,0} - p_{T,A,0} - r \alpha_2 p_{T,A,0} + r S \alpha_2 p_{T,A,0} +$$

$$r \alpha_2 w_{t,B} p_{T,A,0} - r S \alpha_2 w_{t,B} p_{T,A,0} + w_{T,A} p_{T,A,0} - \alpha_2 w_{T,A} p_{T,A,0} +$$
$$r \alpha_2 w_{T,A} p_{T,A,0} - r S \alpha_2 w_{T,A} p_{T,A,0} + \alpha_2 w_{T,B} p_{T,A,0} -$$
$$r \alpha_2 w_{T,B} p_{T,A,0} + r S \alpha_2 w_{T,B} p_{T,A,0} + r p_{t,B,0} p_{T,A,0} -$$
$$r S p_{t,B,0} p_{T,A,0} - r \beta_1 p_{t,B,0} p_{T,A,0} + r S \beta_1 p_{t,B,0} p_{T,A,0} -$$
$$r w_{t,B} p_{t,B,0} p_{T,A,0} + r S w_{t,B} p_{t,B,0} p_{T,A,0} + r \beta_1 w_{t,B} p_{t,B,0} p_{T,A,0} -$$
$$r S \beta_1 w_{t,B} p_{t,B,0} p_{T,A,0} - r w_{T,A} p_{t,B,0} p_{T,A,0} + r S w_{T,A} p_{t,B,0} p_{T,A,0} +$$
$$r \beta_1 w_{T,A} p_{t,B,0} p_{T,A,0} - r S \beta_1 w_{T,A} p_{t,B,0} p_{T,A,0} +$$
$$r w_{T,B} p_{t,B,0} p_{T,A,0} - r S w_{T,B} p_{t,B,0} p_{T,A,0} - r \beta_1 w_{T,B} p_{t,B,0} p_{T,A,0} +$$
$$r S \beta_1 w_{T,B} p_{t,B,0} p_{T,A,0} + r \alpha_2 p_{T,A,0}^2 - r S \alpha_2 p_{T,A,0}^2 - r \alpha_2 w_{t,B} p_{T,A,0}^2 +$$
$$r S \alpha_2 w_{t,B} p_{T,A,0}^2 - r \alpha_2 w_{T,A} p_{T,A,0}^2 + r S \alpha_2 w_{T,A} p_{T,A,0}^2 +$$
$$r \alpha_2 w_{T,B} p_{T,A,0}^2 - r S \alpha_2 w_{T,B} p_{T,A,0}^2 - p_{T,B,0} - r p_{T,B,0} + r S p_{T,B,0} +$$
$$r w_{t,B} p_{T,B,0} - r S w_{t,B} p_{T,B,0} + r w_{T,A} p_{T,B,0} - r S w_{T,A} p_{T,B,0} +$$
$$w_{T,B} p_{T,B,0} - r w_{T,B} p_{T,B,0} + r S w_{T,B} p_{T,B,0} + r p_{t,B,0} p_{T,B,0} -$$
$$r S p_{t,B,0} p_{T,B,0} - r \beta_1 p_{t,B,0} p_{T,B,0} + r S \beta_1 p_{t,B,0} p_{T,B,0} -$$
$$r w_{t,B} p_{t,B,0} p_{T,B,0} + r S w_{t,B} p_{t,B,0} p_{T,B,0} + r \beta_1 w_{t,B} p_{t,B,0} p_{T,B,0} -$$
$$r S \beta_1 w_{t,B} p_{t,B,0} p_{T,B,0} - r w_{T,A} p_{t,B,0} p_{T,B,0} + r S w_{T,A} p_{t,B,0} p_{T,B,0} +$$
$$r \beta_1 w_{T,A} p_{t,B,0} p_{T,B,0} - r S \beta_1 w_{T,A} p_{t,B,0} p_{T,B,0} +$$
$$r w_{T,B} p_{t,B,0} p_{T,B,0} - r S w_{T,B} p_{t,B,0} p_{T,B,0} - r \beta_1 w_{T,B} p_{t,B,0} p_{T,B,0} +$$
$$r S \beta_1 w_{T,B} p_{t,B,0} p_{T,B,0} + r p_{T,A,0} p_{T,B,0} - r S p_{T,A,0} p_{T,B,0} +$$
$$r \alpha_2 p_{T,A,0} p_{T,B,0} - r S \alpha_2 p_{T,A,0} p_{T,B,0} - r w_{t,B} p_{T,A,0} p_{T,B,0} +$$
$$r S w_{t,B} p_{T,A,0} p_{T,B,0} - r \alpha_2 w_{t,B} p_{T,A,0} p_{T,B,0} + r S \alpha_2 w_{t,B} p_{T,A,0} p_{T,B,0} -$$
$$r w_{T,A} p_{T,A,0} p_{T,B,0} + r S w_{T,A} p_{T,A,0} p_{T,B,0} - r \alpha_2 w_{T,A} p_{T,A,0} p_{T,B,0} +$$
$$r S \alpha_2 w_{T,A} p_{T,A,0} p_{T,B,0} + r w_{T,B} p_{T,A,0} p_{T,B,0} - r S w_{T,B} p_{T,A,0} p_{T,B,0} +$$
$$r \alpha_2 w_{T,B} p_{T,A,0} p_{T,B,0} - r S \alpha_2 w_{T,B} p_{T,A,0} p_{T,B,0} + r p_{T,B,0}^2 -$$
$$r S p_{T,B,0}^2 - r w_{t,B} p_{T,B,0}^2 + r S w_{t,B} p_{T,B,0}^2 - r w_{T,A} p_{T,B,0}^2 +$$
$$r S w_{T,A} p_{T,B,0}^2 + r w_{T,B} p_{T,B,0}^2 - r S w_{T,B} p_{T,B,0}^2 \Big)\Big) == 0,$$

$$\Big(-p_{T,B,1} + \Big(z\, w_{t,B} \Big((-1 + S)(-1 + \beta_1) p_{t,B,0}^2 + r(-1 + S)(-1 + p_{t,B,0} + p_{T,A,0} + p_{T,B,0})\,(\alpha_2 p_{T,A,0} + p_{T,B,0}) -$$
$$p_{t,B,0} \Big(-1 + \beta_1 + p_{t,B,0} - S p_{t,B,0} - \beta_1 p_{t,B,0} + S \beta_1 p_{t,B,0} + r p_{T,A,0} - r S p_{T,A,0} - r \alpha_2$$
$$p_{T,A,0} + r S \alpha_2 p_{T,A,0} - r \beta_1 p_{T,A,0} + r S \beta_1 p_{T,A,0} - r \beta_1 p_{T,B,0} + r S \beta_1 p_{T,B,0}\Big)\Big) +$$
$$w_{T,B} \Big(r \alpha_1 p_{T,A,0} - r S \alpha_1 p_{T,A,0} - r \alpha_1 p_{t,B,0} p_{T,A,0} + r S \alpha_1 p_{t,B,0} p_{T,A,0} +$$
$$r p_{t,B,1} p_{T,A,0} - r S p_{t,B,1} p_{T,A,0} - r \beta_1 p_{t,B,1} p_{T,A,0} + r S \beta_1 p_{t,B,1} p_{T,A,0} - r \alpha_1 p_{T,A,0}^2 +$$
$$r S \alpha_1 p_{T,A,0}^2 + \alpha_2 p_{T,A,1} - r \alpha_2 p_{T,A,1} + r S \alpha_2 p_{T,A,1} + r p_{t,B,0} p_{T,A,1} - r S p_{t,B,0} p_{T,A,1} -$$
$$r \beta_1 p_{t,B,0} p_{T,A,1} + r S \beta_1 p_{t,B,0} p_{T,A,1} + 2 r \alpha_2 p_{T,A,0} p_{T,A,1} - 2 r S \alpha_2 p_{T,A,0} p_{T,A,1} +$$
$$r \alpha_1 p_{T,B,0} - r S \alpha_1 p_{T,B,0} - \beta_2 p_{T,B,0} + r \beta_2 p_{T,B,0} - r S \beta_2 p_{T,B,0} - r \alpha_1 p_{t,B,0} p_{T,B,0} +$$
$$r S \alpha_1 p_{t,B,0} p_{T,B,0} + r p_{t,B,1} p_{T,B,0} - r S p_{t,B,1} p_{T,B,0} - r \beta_1 p_{t,B,1} p_{T,B,0} +$$
$$r S \beta_1 p_{t,B,1} p_{T,B,0} - 2 r \alpha_1 p_{T,A,0} p_{T,B,0} + 2 r S \alpha_1 p_{T,A,0} p_{T,B,0} - r \beta_2 p_{T,A,0} p_{T,B,0} +$$
$$r S \beta_2 p_{T,A,0} p_{T,B,0} + r p_{T,A,1} p_{T,B,0} - r S p_{T,A,1} p_{T,B,0} + r \alpha_2 p_{T,A,1} p_{T,B,0} -$$
$$r S \alpha_2 p_{T,A,1} p_{T,B,0} - r \alpha_1 p_{T,B,0}^2 + r S \alpha_1 p_{T,B,0}^2 - r \beta_2 p_{T,B,0}^2 + r S \beta_2 p_{T,B,0}^2 -$$
$$y \Big(\alpha_2 p_{T,A,0} - r \alpha_2 p_{T,A,0} + r S \alpha_2 p_{T,A,0} + r p_{t,B,0} p_{T,A,0} - r S p_{t,B,0} p_{T,A,0} - r \beta_1 p_{t,B,0}$$
$$p_{T,A,0} + r S \beta_1 p_{t,B,0} p_{T,A,0} + r \alpha_2 p_{T,A,0}^2 - r S \alpha_2 p_{T,A,0}^2 + p_{T,B,0} - r p_{T,B,0} + r S$$
$$p_{T,B,0} + r p_{t,B,0} p_{T,B,0} - r S p_{t,B,0} p_{T,B,0} - r \beta_1 p_{t,B,0} p_{T,B,0} + r S \beta_1 p_{t,B,0} p_{T,B,0} + r$$

$$p_{T,A,0}\, p_{T,B,0} - r\, S\, p_{T,A,0}\, p_{T,B,0} + r\, \alpha_2\, p_{T,A,0}\, p_{T,B,0} - r\, S\, \alpha_2\, p_{T,A,0}\, p_{T,B,0} + r$$
$$p_{T,B,0}^2 - r\, S\, p_{T,B,0}^2\Big) + p_{T,B,1} - r\, p_{T,B,1} + r\, S\, p_{T,B,1} + r\, p_{t,B,0}\, p_{T,B,1} - r\, S\, p_{t,B,0}\, p_{T,B,1} -$$
$$r\, \beta_1\, p_{t,B,0}\, p_{T,B,1} + r\, S\, \beta_1\, p_{t,B,0}\, p_{T,B,1} + r\, p_{T,A,0}\, p_{T,B,1} - r\, S\, p_{T,A,0}\, p_{T,B,1} +$$
$$r\, \alpha_2\, p_{T,A,0}\, p_{T,B,1} - r\, S\, \alpha_2\, p_{T,A,0}\, p_{T,B,1} + 2\, r\, p_{T,B,0}\, p_{T,B,1} - 2\, r\, S\, p_{T,B,0}\, p_{T,B,1}\Big)\Big) \Big/$$
$$\Big(1 - p_{t,B,0} + \beta_1\, p_{t,B,0} + w_{t,B}\, p_{t,B,0} - \beta_1\, w_{t,B}\, p_{t,B,0} - p_{T,A,0} - r\, \alpha_2\, p_{T,A,0} + r\, S\, \alpha_2\, p_{T,A,0} +$$
$$r\, \alpha_2\, w_{t,B}\, p_{T,A,0} - r\, S\, \alpha_2\, w_{t,B}\, p_{T,A,0} + w_{T,A}\, p_{T,A,0} - \alpha_2\, w_{T,A}\, p_{T,A,0} +$$
$$r\, \alpha_2\, w_{T,A}\, p_{T,A,0} - r\, S\, \alpha_2\, w_{T,A}\, p_{T,A,0} + \alpha_2\, w_{T,B}\, p_{T,A,0} - r\, \alpha_2\, w_{T,B}\, p_{T,A,0} +$$
$$r\, S\, \alpha_2\, w_{T,B}\, p_{T,A,0} + r\, p_{t,B,0}\, p_{T,A,0} - r\, S\, p_{t,B,0}\, p_{T,A,0} -$$
$$r\, \beta_1\, p_{t,B,0}\, p_{T,A,0} + r\, S\, \beta_1\, p_{t,B,0}\, p_{T,A,0} - r\, w_{t,B}\, p_{t,B,0}\, p_{T,A,0} +$$
$$r\, S\, w_{t,B}\, p_{t,B,0}\, p_{T,A,0} + r\, \beta_1\, w_{t,B}\, p_{t,B,0}\, p_{T,A,0} - r\, S\, \beta_1\, w_{t,B}\, p_{t,B,0}\, p_{T,A,0} -$$
$$r\, w_{T,A}\, p_{t,B,0}\, p_{T,A,0} + r\, S\, w_{T,A}\, p_{t,B,0}\, p_{T,A,0} + r\, \beta_1\, w_{T,A}\, p_{t,B,0}\, p_{T,A,0} -$$
$$r\, S\, \beta_1\, w_{T,A}\, p_{t,B,0}\, p_{T,A,0} + r\, w_{T,B}\, p_{t,B,0}\, p_{T,A,0} - r\, S\, w_{T,B}\, p_{t,B,0}\, p_{T,A,0} -$$
$$r\, \beta_1\, w_{T,B}\, p_{t,B,0}\, p_{T,A,0} + r\, S\, \beta_1\, w_{T,B}\, p_{t,B,0}\, p_{T,A,0} + r\, \alpha_2\, p_{T,A,0}^2 -$$
$$r\, S\, \alpha_2\, p_{T,A,0}^2 - r\, \alpha_2\, w_{t,B}\, p_{T,A,0}^2 + r\, S\, \alpha_2\, w_{t,B}\, p_{T,A,0}^2 - r\, \alpha_2\, w_{T,A}\, p_{T,A,0}^2 +$$
$$r\, S\, \alpha_2\, w_{T,A}\, p_{T,A,0}^2 + r\, \alpha_2\, w_{T,B}\, p_{T,A,0}^2 - r\, S\, \alpha_2\, w_{T,B}\, p_{T,A,0}^2 - p_{T,B,0} -$$
$$r\, p_{T,B,0} + r\, S\, p_{T,B,0} + r\, w_{t,B}\, p_{T,B,0} - r\, S\, w_{t,B}\, p_{T,B,0} + r\, w_{T,A}\, p_{T,B,0} -$$
$$r\, S\, w_{T,A}\, p_{T,B,0} + w_{T,B}\, p_{T,B,0} - r\, w_{T,B}\, p_{T,B,0} + r\, S\, w_{T,B}\, p_{T,B,0} +$$
$$r\, p_{t,B,0}\, p_{T,B,0} - r\, S\, p_{t,B,0}\, p_{T,B,0} - r\, \beta_1\, p_{t,B,0}\, p_{T,B,0} +$$
$$r\, S\, \beta_1\, p_{t,B,0}\, p_{T,B,0} - r\, w_{t,B}\, p_{t,B,0}\, p_{T,B,0} + r\, S\, w_{t,B}\, p_{t,B,0}\, p_{T,B,0} +$$
$$r\, \beta_1\, w_{t,B}\, p_{t,B,0}\, p_{T,B,0} - r\, S\, \beta_1\, w_{t,B}\, p_{t,B,0}\, p_{T,B,0} - r\, w_{T,A}\, p_{t,B,0}\, p_{T,B,0} +$$
$$r\, S\, w_{T,A}\, p_{t,B,0}\, p_{T,B,0} + r\, \beta_1\, w_{T,A}\, p_{t,B,0}\, p_{T,B,0} - r\, S\, \beta_1\, w_{T,A}\, p_{t,B,0}\, p_{T,B,0} +$$
$$r\, w_{T,B}\, p_{t,B,0}\, p_{T,B,0} - r\, S\, w_{T,B}\, p_{t,B,0}\, p_{T,B,0} - r\, \beta_1\, w_{T,B}\, p_{t,B,0}\, p_{T,B,0} +$$
$$r\, S\, \beta_1\, w_{T,B}\, p_{t,B,0}\, p_{T,B,0} + r\, p_{T,A,0}\, p_{T,B,0} - r\, S\, p_{T,A,0}\, p_{T,B,0} +$$
$$r\, \alpha_2\, p_{T,A,0}\, p_{T,B,0} - r\, S\, \alpha_2\, p_{T,A,0}\, p_{T,B,0} - r\, w_{t,B}\, p_{T,A,0}\, p_{T,B,0} +$$
$$r\, S\, w_{t,B}\, p_{T,A,0}\, p_{T,B,0} - r\, \alpha_2\, w_{t,B}\, p_{T,A,0}\, p_{T,B,0} + r\, S\, \alpha_2\, w_{t,B}\, p_{T,A,0}\, p_{T,B,0} -$$
$$r\, w_{T,A}\, p_{T,A,0}\, p_{T,B,0} + r\, S\, w_{T,A}\, p_{T,A,0}\, p_{T,B,0} - r\, \alpha_2\, w_{T,A}\, p_{T,A,0}\, p_{T,B,0} +$$
$$r\, S\, \alpha_2\, w_{T,A}\, p_{T,A,0}\, p_{T,B,0} + r\, w_{T,B}\, p_{T,A,0}\, p_{T,B,0} - r\, S\, w_{T,B}\, p_{T,A,0}\, p_{T,B,0} +$$
$$r\, \alpha_2\, w_{T,B}\, p_{T,A,0}\, p_{T,B,0} - r\, S\, \alpha_2\, w_{T,B}\, p_{T,A,0}\, p_{T,B,0} + r\, p_{T,B,0}^2 -$$
$$r\, S\, p_{T,B,0}^2 - r\, w_{t,B}\, p_{T,B,0}^2 + r\, S\, w_{t,B}\, p_{T,B,0}^2 - r\, w_{T,A}\, p_{T,B,0}^2 +$$
$$r\, S\, w_{T,A}\, p_{T,B,0}^2 + r\, w_{T,B}\, p_{T,B,0}^2 - r\, S\, w_{T,B}\, p_{T,B,0}^2\Big) -$$
$$\Big(w_{T,B}\, \Big(\alpha_2\, p_{T,A,0} - r\, \alpha_2\, p_{T,A,0} + r\, S\, \alpha_2\, p_{T,A,0} + r\, p_{t,B,0}\, p_{T,A,0} - r\, S\, p_{t,B,0}\, p_{T,A,0} - r\, \beta_1\, p_{t,B,0}\, p_{T,A,0} +$$
$$r\, S\, \beta_1\, p_{t,B,0}\, p_{T,A,0} + r\, \alpha_2\, p_{T,A,0}^2 - r\, S\, \alpha_2\, p_{T,A,0}^2 + p_{T,B,0} - r\, p_{T,B,0} + r\, S\, p_{T,B,0} +$$
$$r\, p_{t,B,0}\, p_{T,B,0} - r\, S\, p_{t,B,0}\, p_{T,B,0} - r\, \beta_1\, p_{t,B,0}\, p_{T,B,0} + r\, S\, \beta_1\, p_{t,B,0}\, p_{T,B,0} + r\, p_{T,A,0}\, p_{T,B,0} -$$
$$r\, S\, p_{T,A,0}\, p_{T,B,0} + r\, \alpha_2\, p_{T,A,0}\, p_{T,B,0} - r\, S\, \alpha_2\, p_{T,A,0}\, p_{T,B,0} + r\, p_{T,B,0}^2 - r\, S\, p_{T,B,0}^2\Big)$$
$$\Big(S\, w_{T,A}\, p_{T,A,1} - S\, \alpha_2\, w_{T,A}\, p_{T,A,1} + S\, \alpha_2\, w_{T,B}\, p_{T,A,1} + 2\, w_{T,A}\, p_{T,A,0}\, p_{T,A,1} -$$
$$2\, S\, w_{T,A}\, p_{T,A,0}\, p_{T,A,1} - 2\, \alpha_2\, w_{T,A}\, p_{T,A,0}\, p_{T,A,1} + 2\, S\, \alpha_2\, w_{T,A}\, p_{T,A,0}\, p_{T,A,1} +$$
$$2\, \alpha_2\, w_{T,B}\, p_{T,A,0}\, p_{T,A,1} - 2\, S\, \alpha_2\, w_{T,B}\, p_{T,A,0}\, p_{T,A,1} + w_{T,A}\, \left(p_{t,B,1}\, p_{T,A,0} + p_{t,B,0}\, p_{T,A,1}\right) -$$
$$r\, w_{T,A}\, \left(p_{t,B,1}\, p_{T,A,0} + p_{t,B,0}\, p_{T,A,1}\right) - S\, w_{T,A}\, \left(p_{t,B,1}\, p_{T,A,0} + p_{t,B,0}\, p_{T,A,1}\right) +$$
$$r\, S\, w_{T,A}\, \left(p_{t,B,1}\, p_{T,A,0} + p_{t,B,0}\, p_{T,A,1}\right) - \alpha_2\, w_{T,A}\, \left(p_{t,B,1}\, p_{T,A,0} + p_{t,B,0}\, p_{T,A,1}\right) +$$
$$r\, \alpha_2\, w_{T,A}\, \left(p_{t,B,1}\, p_{T,A,0} + p_{t,B,0}\, p_{T,A,1}\right) + S\, \alpha_2\, w_{T,A}\, \left(p_{t,B,1}\, p_{T,A,0} + p_{t,B,0}\, p_{T,A,1}\right) -$$
$$r\, S\, \alpha_2\, w_{T,A}\, \left(p_{t,B,1}\, p_{T,A,0} + p_{t,B,0}\, p_{T,A,1}\right) + r\, \beta_1\, w_{T,A}\, \left(p_{t,B,1}\, p_{T,A,0} + p_{t,B,0}\, p_{T,A,1}\right) -$$

$$r\,S\,\beta_1\,w_{T,A}\left(p_{t,B,1}\,p_{T,A,0}+p_{t,B,0}\,p_{T,A,1}\right)+r\,w_{T,B}\left(p_{t,B,1}\,p_{T,A,0}+p_{t,B,0}\,p_{T,A,1}\right)-$$
$$r\,S\,w_{T,B}\left(p_{t,B,1}\,p_{T,A,0}+p_{t,B,0}\,p_{T,A,1}\right)+\alpha_2\,w_{T,B}\left(p_{t,B,1}\,p_{T,A,0}+p_{t,B,0}\,p_{T,A,1}\right)-$$
$$r\,\alpha_2\,w_{T,B}\left(p_{t,B,1}\,p_{T,A,0}+p_{t,B,0}\,p_{T,A,1}\right)-S\,\alpha_2\,w_{T,B}\left(p_{t,B,1}\,p_{T,A,0}+p_{t,B,0}\,p_{T,A,1}\right)+$$
$$r\,S\,\alpha_2\,w_{T,B}\left(p_{t,B,1}\,p_{T,A,0}+p_{t,B,0}\,p_{T,A,1}\right)-r\,\beta_1\,w_{T,B}\left(p_{t,B,1}\,p_{T,A,0}+p_{t,B,0}\,p_{T,A,1}\right)+$$
$$r\,S\,\beta_1\,w_{T,B}\left(p_{t,B,1}\,p_{T,A,0}+p_{t,B,0}\,p_{T,A,1}\right)+S\,\beta_2\,w_{T,A}\,p_{T,B,0}-S\,\beta_2\,w_{T,B}\,p_{T,B,0}+$$
$$\beta_2\,w_{T,A}\,p_{t,B,0}\,p_{T,B,0}-r\,\beta_2\,w_{T,A}\,p_{t,B,0}\,p_{T,B,0}-S\,\beta_2\,w_{T,A}\,p_{t,B,0}\,p_{T,B,0}+$$
$$r\,S\,\beta_2\,w_{T,A}\,p_{t,B,0}\,p_{T,B,0}-\beta_2\,w_{T,B}\,p_{t,B,0}\,p_{T,B,0}+r\,\beta_2\,w_{T,B}\,p_{t,B,0}\,p_{T,B,0}+$$
$$S\,\beta_2\,w_{T,B}\,p_{t,B,0}\,p_{T,B,0}-r\,S\,\beta_2\,w_{T,B}\,p_{t,B,0}\,p_{T,B,0}+\beta_2\,w_{T,A}\,p_{T,A,0}\,p_{T,B,0}-$$
$$S\,\beta_2\,w_{T,A}\,p_{T,A,0}\,p_{T,B,0}-\beta_2\,w_{T,B}\,p_{T,A,0}\,p_{T,B,0}+S\,\beta_2\,w_{T,B}\,p_{T,A,0}\,p_{T,B,0}+$$
$$\beta_2\,w_{T,A}\,p_{T,B,0}^2-S\,\beta_2\,w_{T,A}\,p_{T,B,0}^2-\beta_2\,w_{T,B}\,p_{T,B,0}^2+S\,\beta_2\,w_{T,B}\,p_{T,B,0}^2+r\,\alpha_1\,w_{T,A}\,p_{T,A,0}$$
$$\left(-1+p_{t,B,0}+p_{T,A,0}+p_{T,B,0}\right)-r\,S\,\alpha_1\,w_{T,A}\,p_{T,A,0}\left(-1+p_{t,B,0}+p_{T,A,0}+p_{T,B,0}\right)-$$
$$r\,\alpha_1\,w_{T,B}\,p_{T,A,0}\left(-1+p_{t,B,0}+p_{T,A,0}+p_{T,B,0}\right)+r\,S\,\alpha_1\,w_{T,B}\,p_{T,A,0}$$
$$\left(-1+p_{t,B,0}+p_{T,A,0}+p_{T,B,0}\right)+r\,\alpha_1\,w_{T,A}\,p_{T,B,0}\left(-1+p_{t,B,0}+p_{T,A,0}+p_{T,B,0}\right)-$$
$$r\,S\,\alpha_1\,w_{T,A}\,p_{T,B,0}\left(-1+p_{t,B,0}+p_{T,A,0}+p_{T,B,0}\right)-\beta_2\,w_{T,A}\,p_{T,B,0}\left(-1+p_{t,B,0}+p_{T,A,0}+p_{T,B,0}\right)+$$
$$r\,\beta_2\,w_{T,A}\,p_{T,B,0}\left(-1+p_{t,B,0}+p_{T,A,0}+p_{T,B,0}\right)+S\,\beta_2\,w_{T,A}\,p_{T,B,0}\left(-1+p_{t,B,0}+p_{T,A,0}+p_{T,B,0}\right)-$$
$$r\,S\,\beta_2\,w_{T,A}\,p_{T,B,0}\left(-1+p_{t,B,0}+p_{T,A,0}+p_{T,B,0}\right)-r\,\alpha_1\,w_{T,B}\,p_{T,B,0}\left(-1+p_{t,B,0}+p_{T,A,0}+p_{T,B,0}\right)+$$
$$r\,S\,\alpha_1\,w_{T,B}\,p_{T,B,0}\left(-1+p_{t,B,0}+p_{T,A,0}+p_{T,B,0}\right)+\beta_2\,w_{T,B}\,p_{T,B,0}\left(-1+p_{t,B,0}+p_{T,A,0}+p_{T,B,0}\right)-$$
$$r\,\beta_2\,w_{T,B}\,p_{T,B,0}\left(-1+p_{t,B,0}+p_{T,A,0}+p_{T,B,0}\right)-S\,\beta_2\,w_{T,B}\,p_{T,B,0}\left(-1+p_{t,B,0}+p_{T,A,0}+p_{T,B,0}\right)+$$
$$r\,S\,\beta_2\,w_{T,B}\,p_{T,B,0}\left(-1+p_{t,B,0}+p_{T,A,0}+p_{T,B,0}\right)+p_{t,B,1}\Big(r\,(-1+S)\,(-1+\alpha_2)\,p_{T,A,0}+$$
$$\beta_1\left(S-(-1+S)\,p_{t,B,0}+(-1+r)\,(-1+S)\,p_{T,A,0}+p_{T,B,0}-r\,p_{T,B,0}-S\,p_{T,B,0}+r\,S\,p_{T,B,0}\right)\Big)+$$
$$S\,w_{T,A}\left(-p_{T,A,1}\left(1-p_{t,B,0}-p_{T,A,0}-p_{T,B,0}\right)-p_{T,A,0}\left(-p_{t,B,1}-p_{T,A,1}-p_{T,B,1}\right)\right)+$$
$$\alpha_2\,w_{T,A}\left(-p_{T,A,1}\left(1-p_{t,B,0}-p_{T,A,0}-p_{T,B,0}\right)-p_{T,A,0}\left(-p_{t,B,1}-p_{T,A,1}-p_{T,B,1}\right)\right)+$$
$$r\,S\,\alpha_2\,w_{T,A}\left(-p_{T,A,1}\left(1-p_{t,B,0}-p_{T,A,0}-p_{T,B,0}\right)-p_{T,A,0}\left(-p_{t,B,1}-p_{T,A,1}-p_{T,B,1}\right)\right)+$$
$$r\,\alpha_2\,w_{T,B}\left(-p_{T,A,1}\left(1-p_{t,B,0}-p_{T,A,0}-p_{T,B,0}\right)-p_{T,A,0}\left(-p_{t,B,1}-p_{T,A,1}-p_{T,B,1}\right)\right)+$$
$$S\,\alpha_2\,w_{T,B}\left(-p_{T,A,1}\left(1-p_{t,B,0}-p_{T,A,0}-p_{T,B,0}\right)-p_{T,A,0}\left(-p_{t,B,1}-p_{T,A,1}-p_{T,B,1}\right)\right)+$$
$$w_{T,A}\left(p_{T,A,1}\left(1-p_{t,B,0}-p_{T,A,0}-p_{T,B,0}\right)+p_{T,A,0}\left(-p_{t,B,1}-p_{T,A,1}-p_{T,B,1}\right)\right)+$$
$$r\,\alpha_2\,w_{T,A}\left(p_{T,A,1}\left(1-p_{t,B,0}-p_{T,A,0}-p_{T,B,0}\right)+p_{T,A,0}\left(-p_{t,B,1}-p_{T,A,1}-p_{T,B,1}\right)\right)+$$
$$S\,\alpha_2\,w_{T,A}\left(p_{T,A,1}\left(1-p_{t,B,0}-p_{T,A,0}-p_{T,B,0}\right)+p_{T,A,0}\left(-p_{t,B,1}-p_{T,A,1}-p_{T,B,1}\right)\right)+$$
$$\alpha_2\,w_{T,B}\left(p_{T,A,1}\left(1-p_{t,B,0}-p_{T,A,0}-p_{T,B,0}\right)+p_{T,A,0}\left(-p_{t,B,1}-p_{T,A,1}-p_{T,B,1}\right)\right)+$$
$$r\,S\,\alpha_2\,w_{T,B}\left(p_{T,A,1}\left(1-p_{t,B,0}-p_{T,A,0}-p_{T,B,0}\right)+p_{T,A,0}\left(-p_{t,B,1}-p_{T,A,1}-p_{T,B,1}\right)\right)+$$
$$\Big(S+p_{t,B,0}-S\,p_{t,B,0}+\beta_1\,p_{t,B,0}-S\,\beta_1\,p_{t,B,0}+p_{T,A,0}-S\,p_{T,A,0}-r\,\alpha_2\,p_{T,A,0}+r\,S\,\alpha_2\,p_{T,A,0}+$$
$$p_{T,B,0}-r\,p_{T,B,0}-S\,p_{T,B,0}+r\,S\,p_{T,B,0}\Big)\left(-p_{t,B,1}-p_{T,A,1}-p_{T,B,1}\right)+S\,w_{T,B}\,p_{T,B,1}+$$
$$2\,w_{T,B}\,p_{T,B,0}\,p_{T,B,1}-2\,S\,w_{T,B}\,p_{T,B,0}\,p_{T,B,1}+\left(1-p_{t,B,0}-p_{T,A,0}-p_{T,B,0}\right)$$
$$\Big(p_{t,B,1}-S\,p_{t,B,1}+\beta_1\,p_{t,B,1}-S\,\beta_1\,p_{t,B,1}+p_{T,A,1}-S\,p_{T,A,1}-r\,\alpha_2\,p_{T,A,1}+r\,S\,\alpha_2\,p_{T,A,1}+$$
$$r\,\beta_2\,p_{T,B,0}-r\,S\,\beta_2\,p_{T,B,0}+\alpha_1\Big(-S+(-1+S)\,p_{t,B,0}-(-1+r)\,(-1+S)\,p_{T,A,0}-p_{T,B,0}+$$
$$r\,p_{T,B,0}+S\,p_{T,B,0}-r\,S\,p_{T,B,0}\Big)+p_{T,B,1}-r\,p_{T,B,1}-S\,p_{T,B,1}+r\,S\,p_{T,B,1}\Big)+$$
$$r\,\beta_1\,w_{T,A}\left(p_{t,B,1}\,p_{T,B,0}+p_{t,B,0}\,p_{T,B,1}\right)-r\,S\,\beta_1\,w_{T,A}\left(p_{t,B,1}\,p_{T,B,0}+p_{t,B,0}\,p_{T,B,1}\right)+$$
$$w_{T,B}\left(p_{t,B,1}\,p_{T,B,0}+p_{t,B,0}\,p_{T,B,1}\right)-S\,w_{T,B}\left(p_{t,B,1}\,p_{T,B,0}+p_{t,B,0}\,p_{T,B,1}\right)-$$
$$r\,\beta_1\,w_{T,B}\left(p_{t,B,1}\,p_{T,B,0}+p_{t,B,0}\,p_{T,B,1}\right)+r\,S\,\beta_1\,w_{T,B}\left(p_{t,B,1}\,p_{T,B,0}+p_{t,B,0}\,p_{T,B,1}\right)+$$
$$w_{T,A}\left(p_{T,A,1}\,p_{T,B,0}+p_{T,A,0}\,p_{T,B,1}\right)-S\,w_{T,A}\left(p_{T,A,1}\,p_{T,B,0}+p_{T,A,0}\,p_{T,B,1}\right)-$$

$$\alpha_2 w_{T,A} (p_{T,A,1} p_{T,B,0} + p_{T,A,0} p_{T,B,1}) + S \alpha_2 w_{T,A} (p_{T,A,1} p_{T,B,0} + p_{T,A,0} p_{T,B,1}) +$$
$$w_{T,B} (p_{T,A,1} p_{T,B,0} + p_{T,A,0} p_{T,B,1}) - S w_{T,B} (p_{T,A,1} p_{T,B,0} + p_{T,A,0} p_{T,B,1}) +$$
$$\alpha_2 w_{T,B} (p_{T,A,1} p_{T,B,0} + p_{T,A,0} p_{T,B,1}) - S \alpha_2 w_{T,B} (p_{T,A,1} p_{T,B,0} + p_{T,A,0} p_{T,B,1}) +$$
$$r S w_{T,A} (-p_{T,B,0} (-p_{t,B,1} - p_{T,A,1} - p_{T,B,1}) - (1 - p_{t,B,0} - p_{T,A,0} - p_{T,B,0}) p_{T,B,1}) +$$
$$r w_{T,B} (-p_{T,B,0} (-p_{t,B,1} - p_{T,A,1} - p_{T,B,1}) - (1 - p_{t,B,0} - p_{T,A,0} - p_{T,B,0}) p_{T,B,1}) +$$
$$S w_{T,B} (-p_{T,B,0} (-p_{t,B,1} - p_{T,A,1} - p_{T,B,1}) - (1 - p_{t,B,0} - p_{T,A,0} - p_{T,B,0}) p_{T,B,1}) +$$
$$r w_{T,A} (p_{T,B,0} (-p_{t,B,1} - p_{T,A,1} - p_{T,B,1}) + (1 - p_{t,B,0} - p_{T,A,0} - p_{T,B,0}) p_{T,B,1}) +$$
$$w_{T,B} (p_{T,B,0} (-p_{t,B,1} - p_{T,A,1} - p_{T,B,1}) + (1 - p_{t,B,0} - p_{T,A,0} - p_{T,B,0}) p_{T,B,1}) +$$
$$r S w_{T,B} (p_{T,B,0} (-p_{t,B,1} - p_{T,A,1} - p_{T,B,1}) + (1 - p_{t,B,0} - p_{T,A,0} - p_{T,B,0}) p_{T,B,1}) + (-1 + S)$$
$$(\alpha_1 (1 - p_{t,B,0} - p_{T,A,0} - p_{T,B,0})^2 - 2 (-1 + p_{t,B,0} + p_{T,A,0} + p_{T,B,0}) (p_{t,B,1} + p_{T,A,1} + p_{T,B,1})) +$$
$$p_{t,B,0} (r (-1 + S) ((-1 + \alpha_2) p_{T,A,1} - \beta_2 p_{T,B,0}) + \beta_1 (-((-1 + S) p_{t,B,1}) + (-1 + r) (-1 + S) p_{T,A,1} +$$
$$p_{T,B,1} - r p_{T,B,1} - S p_{T,B,1} + r S p_{T,B,1})) + w_{t,B} (2 (-1 + S) (-1 + \beta_1) p_{t,B,0} p_{t,B,1} -$$
$$\alpha_1 (-1 + p_{t,B,0} + p_{T,A,0} + p_{T,B,0}) (1 - r p_{T,A,0} + r S p_{T,A,0} - r p_{T,B,0} + r S p_{T,B,0}) +$$
$$p_{t,B,1} (1 - \beta_1 - p_{t,B,0} + S p_{t,B,0} + \beta_1 p_{t,B,0} - S \beta_1 p_{t,B,0} - r p_{T,A,0} + r S p_{T,A,0} +$$
$$r \alpha_2 p_{T,A,0} - r S \alpha_2 p_{T,A,0} + r \beta_1 p_{T,A,0} - r S \beta_1 p_{T,A,0} + r \beta_1 p_{T,B,0} - r S \beta_1 p_{T,B,0}) +$$
$$p_{t,B,0} (-p_{t,B,1} + S p_{t,B,1} + \beta_1 p_{t,B,1} - S \beta_1 p_{t,B,1} - r p_{T,A,1} + r S p_{T,A,1} +$$
$$r \alpha_2 p_{T,A,1} - r S \alpha_2 p_{T,A,1} + r \beta_1 p_{T,A,1} - r S \beta_1 p_{T,A,1} - r \beta_2 p_{T,B,0} + r S \beta_2 p_{T,B,0} +$$
$$r \beta_1 p_{T,B,1} - r S \beta_1 p_{T,B,1}) + r (-1 + S) (-((\alpha_2 p_{T,A,0} + p_{T,B,0}) (-p_{t,B,1} - p_{T,A,1} - p_{T,B,1})) -$$
$$(1 - p_{t,B,0} - p_{T,A,0} - p_{T,B,0}) (\alpha_2 p_{T,A,1} - \beta_2 p_{T,B,0} + p_{T,B,1}))))) /$$
$$(1 - p_{t,B,0} + \beta_1 p_{t,B,0} + w_{t,B} p_{t,B,0} - \beta_1 w_{t,B} p_{t,B,0} - p_{T,A,0} - r \alpha_2 p_{T,A,0} + r S \alpha_2 p_{T,A,0} +$$
$$r \alpha_2 w_{t,B} p_{T,A,0} - r S \alpha_2 w_{t,B} p_{T,A,0} + w_{T,A} p_{T,A,0} - \alpha_2 w_{T,A} p_{T,A,0} + r \alpha_2 w_{T,A} p_{T,A,0} -$$
$$r S \alpha_2 w_{T,A} p_{T,A,0} + \alpha_2 w_{T,B} p_{T,A,0} - r \alpha_2 w_{T,B} p_{T,A,0} + r S \alpha_2 w_{T,B} p_{T,A,0} +$$
$$r p_{t,B,0} p_{T,A,0} - r S p_{t,B,0} p_{T,A,0} - r \beta_1 p_{t,B,0} p_{T,A,0} + r S \beta_1 p_{t,B,0} p_{T,A,0} -$$
$$r w_{t,B} p_{t,B,0} p_{T,A,0} + r S w_{t,B} p_{t,B,0} p_{T,A,0} + r \beta_1 w_{t,B} p_{t,B,0} p_{T,A,0} -$$
$$r S \beta_1 w_{t,B} p_{t,B,0} p_{T,A,0} - r w_{T,A} p_{t,B,0} p_{T,A,0} + r S w_{T,A} p_{t,B,0} p_{T,A,0} +$$
$$r \beta_1 w_{T,A} p_{t,B,0} p_{T,A,0} - r S \beta_1 w_{T,A} p_{t,B,0} p_{T,A,0} + r w_{T,B} p_{t,B,0} p_{T,A,0} -$$
$$r S w_{T,B} p_{t,B,0} p_{T,A,0} - r \beta_1 w_{T,B} p_{t,B,0} p_{T,A,0} + r S \beta_1 w_{T,B} p_{t,B,0} p_{T,A,0} +$$
$$r \alpha_2 p_{T,A,0}^2 - r S \alpha_2 p_{T,A,0}^2 - r \alpha_2 w_{t,B} p_{T,A,0}^2 + r S \alpha_2 w_{t,B} p_{T,A,0}^2 - r \alpha_2 w_{T,A} p_{T,A,0}^2 +$$
$$r S \alpha_2 w_{T,A} p_{T,A,0}^2 + r \alpha_2 w_{T,B} p_{T,A,0}^2 - r S \alpha_2 w_{T,B} p_{T,A,0}^2 - p_{T,B,0} - r p_{T,B,0} +$$
$$r S p_{T,B,0} + r w_{t,B} p_{T,B,0} - r S w_{t,B} p_{T,B,0} + r w_{T,A} p_{T,B,0} - r S w_{T,A} p_{T,B,0} +$$
$$w_{T,B} p_{T,B,0} - r w_{T,B} p_{T,B,0} + r S w_{T,B} p_{T,B,0} + r p_{t,B,0} p_{T,B,0} - r S p_{t,B,0} p_{T,B,0} -$$
$$r \beta_1 p_{t,B,0} p_{T,B,0} + r S \beta_1 p_{t,B,0} p_{T,B,0} - r w_{t,B} p_{t,B,0} p_{T,B,0} + r S w_{t,B} p_{t,B,0} p_{T,B,0} +$$
$$r \beta_1 w_{t,B} p_{t,B,0} p_{T,B,0} - r S \beta_1 w_{t,B} p_{t,B,0} p_{T,B,0} - r w_{T,A} p_{t,B,0} p_{T,B,0} +$$
$$r S w_{T,A} p_{t,B,0} p_{T,B,0} + r \beta_1 w_{T,A} p_{t,B,0} p_{T,B,0} - r S \beta_1 w_{T,A} p_{t,B,0} p_{T,B,0} +$$
$$r w_{T,B} p_{t,B,0} p_{T,B,0} - r S w_{T,B} p_{t,B,0} p_{T,B,0} - r \beta_1 w_{T,B} p_{t,B,0} p_{T,B,0} +$$
$$r S \beta_1 w_{T,B} p_{t,B,0} p_{T,B,0} + r p_{T,A,0} p_{T,B,0} - r S p_{T,A,0} p_{T,B,0} + r \alpha_2 p_{T,A,0} p_{T,B,0} -$$
$$r S \alpha_2 p_{T,A,0} p_{T,B,0} - r w_{t,B} p_{T,A,0} p_{T,B,0} + r S w_{t,B} p_{T,A,0} p_{T,B,0} - r \alpha_2 w_{t,B} p_{T,A,0} p_{T,B,0} +$$
$$r S \alpha_2 w_{t,B} p_{T,A,0} p_{T,B,0} - r w_{T,A} p_{T,A,0} p_{T,B,0} + r S w_{T,A} p_{T,A,0} p_{T,B,0} -$$
$$r \alpha_2 w_{T,A} p_{T,A,0} p_{T,B,0} + r S \alpha_2 w_{T,A} p_{T,A,0} p_{T,B,0} + r w_{T,B} p_{T,A,0} p_{T,B,0} -$$
$$r S w_{T,B} p_{T,A,0} p_{T,B,0} + r \alpha_2 w_{T,B} p_{T,A,0} p_{T,B,0} - r S \alpha_2 w_{T,B} p_{T,A,0} p_{T,B,0} + r p_{T,B,0}^2 -$$

```
r S p²T,B,0 - r wt,B p²T,B,0 + r S wt,B p²T,B,0 - r wT,A p²T,B,0 + r S wT,A p²T,B,0 +
r wT,B p²T,B,0 - r S wT,B p²T,B,0)²) == 0} /. {pT,A,0 → 0, pt,B,0 → 0, pT,B,0 → 0};
```

In[]:=
```
Solve[%, {pT,A,1, pt,B,1, pT,B,1}];
```

In[]:=
```
Simplify[%]
```

Out[]=

$$\left\{\left\{p_{T,A,1} \to \frac{z \left(-1 + \left(1 + r \left(-1 + S\right)\right) w_{T,B}\right)}{-1 + \left(1 + r \left(-1 + S\right)\right) w_{T,B} + w_{T,A} \left(1 + \left(1 + r \left(-1 + S\right)\right) \alpha_2 \left(-1 + w_{T,B}\right) + \left(-1 + r - r\, S\right) w_{T,B}\right)},\right.\right.$$

$$p_{t,B,1} \to \left(w_{t,B} \left(r \left(-1 + S\right) z\, \alpha_2 + \alpha_1 \left(-1 + w_{T,B} - r\, w_{T,B} + r\, S\, w_{T,B} + w_{T,A} \left(1 + \left(1 + r \left(-1 + S\right)\right) \alpha_2 \left(-1 + w_{T,B}\right) + \left(-1 + r - r\, S\right) w_{T,B}\right)\right)\right)\right) \Big/ \left(\left(1 + \left(-1 + \beta_1\right) w_{t,B}\right) \left(-1 + \left(1 + r \left(-1 + S\right)\right) w_{T,B} + w_{T,A} \left(1 + \left(1 + r \left(-1 + S\right)\right) \alpha_2 \left(-1 + w_{T,B}\right) + \left(-1 + r - r\, S\right) w_{T,B}\right)\right)\right),$$

$$\left.\left. p_{T,B,1} \to -\frac{\left(1 + r \left(-1 + S\right)\right) z\, \alpha_2\, w_{T,B}}{-1 + \left(1 + r \left(-1 + S\right)\right) w_{T,B} + w_{T,A} \left(1 + \left(1 + r \left(-1 + S\right)\right) \alpha_2 \left(-1 + w_{T,B}\right) + \left(-1 + r - r\, S\right) w_{T,B}\right)}\right\}\right\}$$

After simplification, the haplotype frequencies lead to eq. 2 of the main text.

B3 - Calculating Eigenvalues

```
ResourceFunction["JacobianMatrix"][
  {(z + p_{T,A} - y p_{T,A} - z p_{T,A} - ℒ p_{T,A} + y ℒ p_{T,A} - α2 p_{T,A} + r α2 p_{T,A} + y α2 p_{T,A} - r y α2 p_{T,A} - r z α2 p_{T,A} +
      ℒ α2 p_{T,A} - r ℒ α2 p_{T,A} - y ℒ α2 p_{T,A} + r y ℒ α2 p_{T,A} - r α2 p_{T,A}^2 + r y α2 p_{T,A}^2 + r z α2 p_{T,A}^2 +
      r ℒ α2 p_{T,A}^2 - r y ℒ α2 p_{T,A}^2 + r p_{T,B} - r y p_{T,B} - z p_{T,B} - r z p_{T,B} - r ℒ p_{T,B} + r y ℒ p_{T,B} +
      β2 p_{T,B} - r β2 p_{T,B} - y β2 p_{T,B} + r y β2 p_{T,B} + r z β2 p_{T,B} - ℒ β2 p_{T,B} + r ℒ β2 p_{T,B} + y ℒ β2 p_{T,B} -
      r y ℒ β2 p_{T,B} - r p_{T,A} p_{T,B} + r y p_{T,A} p_{T,B} + r z p_{T,A} p_{T,B} + r ℒ p_{T,A} p_{T,B} - r y ℒ p_{T,A} p_{T,B} -
      r α2 p_{T,A} p_{T,B} + r y α2 p_{T,A} p_{T,B} + r z α2 p_{T,A} p_{T,B} + r ℒ α2 p_{T,A} p_{T,B} - r y ℒ α2 p_{T,A} p_{T,B} +
      r β2 p_{T,A} p_{T,B} - r y β2 p_{T,A} p_{T,B} - r z β2 p_{T,A} p_{T,B} - r ℒ β2 p_{T,A} p_{T,B} + r y ℒ β2 p_{T,A} p_{T,B} - r p_{T,B}^2 +
      r y p_{T,B}^2 + r z p_{T,B}^2 + r ℒ p_{T,B}^2 - r y ℒ p_{T,B}^2 + r β2 p_{T,B}^2 - r y β2 p_{T,B}^2 - r z β2 p_{T,B}^2 - r ℒ β2 p_{T,B}^2 +
      r y ℒ β2 p_{T,B}^2 - (-1 + β1) p_{t,B} (-z + r (-1 + y + z + ℒ - y ℒ) p_{T,A} + r (-1 + y + z + ℒ - y ℒ) p_{T,B}) -
      α1 (-1 + p_{t,B} + p_{T,A} + p_{T,B}) (-z + r (-1 + y + z + ℒ - y ℒ) p_{T,A} + r (-1 + y + z + ℒ - y ℒ) p_{T,B})) /
    (1 + k (-1 + β1) p_{t,B} - ℒ p_{T,A} - k α2 p_{T,A} - k p_{T,B} - ℒ p_{T,B} + k β2 p_{T,B} + k α1 (-1 + p_{t,B} + p_{T,A} + p_{T,B})),
   ((-1 + β1) p_{t,B} (-1 + k + z - k z + r (1 - z + k (-1 + y + z) + y (-1 + ℒ)) p_{T,A} +
          r (1 - z + k (-1 + y + z) + y (-1 + ℒ)) p_{T,B}) + α1 (-1 + p_{t,B} + p_{T,A} + p_{T,B})
        (-1 + k + z - k z + r (1 - z + k (-1 + y + z) + y (-1 + ℒ)) p_{T,A} + r (1 - z + k (-1 + y + z) + y (-1 + ℒ)) p_{T,B}) -
      (-r + k r - y + k y + r y - k r y + r z - k r z + y ℒ - r y ℒ + r (1 - z + k (-1 + y + z) + y (-1 + ℒ)) p_{T,A} +
          r (1 - z + k (-1 + y + z) + y (-1 + ℒ)) p_{T,B}) (α2 p_{T,A} - (-1 + β2) p_{T,B})) /
    (1 + k (-1 + β1) p_{t,B} - ℒ p_{T,A} - k α2 p_{T,A} - k p_{T,B} - ℒ p_{T,B} + k β2 p_{T,B} + k α1 (-1 + p_{t,B} + p_{T,A} + p_{T,B})),
   ((-1 + β1) p_{t,B} (z - k z + r (1 - z + k (-1 + y + z) + y (-1 + ℒ) - ℒ) p_{T,A} +
          r (1 - z + k (-1 + y + z) + y (-1 + ℒ) - ℒ) p_{T,B}) + α1 (-1 + p_{t,B} + p_{T,A} + p_{T,B})
        (z - k z + r (1 - z + k (-1 + y + z) + y (-1 + ℒ) - ℒ) p_{T,A} + r (1 - z + k (-1 + y + z) + y (-1 + ℒ) - ℒ) p_{T,B}) -
      (1 - k - r + k r - y + k y + r y - k r y + r z - k r z - ℒ + r ℒ + y ℒ - r y ℒ + r (1 - z + k (-1 + y + z) +
              y (-1 + ℒ) - ℒ) p_{T,A} + r (1 - z + k (-1 + y + z) + y (-1 + ℒ) - ℒ) p_{T,B}) (α2 p_{T,A} - (-1 + β2) p_{T,B})) /
    (-1 - k (-1 + β1) p_{t,B} + ℒ p_{T,A} + k α2 p_{T,A} + k p_{T,B} + ℒ p_{T,B} - k β2 p_{T,B} - k α1 (-1 + p_{t,B} + p_{T,A} + p_{T,B}))},
  {p_{T,A}, p_{t,B}, p_{T,B}}] // MatrixForm;

% /. {z → z ζ, y → y ζ, β2 → β2 ζ, α1 → α1 ζ, p_{T,A} → (p_{T,A,0} + p_{T,A,1} ζ),
    p_{t,B} → (p_{t,B,0} + p_{t,B,1} ζ), p_{T,B} → (p_{T,B,0} + p_{T,B,1} ζ)} // MatrixForm;

Det[% - ( λ0 + λ1 ζ      0            0
          0            λ0 + λ1 ζ      0
          0            0            λ0 + λ1 ζ )];

Series[% == 0, {ζ, 0, 1}];
```

```
% /. {p_{T,A,0} → 0, p_{t,B,0} → 0, p_{T,B,0} → 0, p_{T,A,1} → (z (-1 + (1 + r (-1 + S)) (1 - k - ℒ))) /
    (-1 + (1 + r (-1 + S)) (1 - k - ℒ) + (1 - ℒ) (1 + (-1 + r - r S) (1 - k - ℒ) + (1 + r (-1 + S)) (-k - ℒ) α2)),
   p_{t,B,1} → ((1 - k) (r (-1 + S) z α2 + α1 (-k - r (1 - k - ℒ) + r S (1 - k - ℒ) - ℒ +
              (1 - ℒ) (1 + (-1 + r - r S) (1 - k - ℒ) + (1 + r (-1 + S)) (-k - ℒ) α2)))) /
     ((-1 + (1 + r (-1 + S)) (1 - k - ℒ) + (1 - ℒ) (1 + (-1 + r - r S) (1 - k - ℒ) + (1 + r (-1 + S)) (-k - ℒ) α2))
       (1 + (1 - k) (-1 + β1))), p_{T,B,1} → -(((1 + r (-1 + S)) z (1 - k - ℒ) α2) /
       (-1 + (1 + r (-1 + S)) (1 - k - ℒ) + (1 - ℒ) (1 + (-1 + r - r S) (1 - k - ℒ) + (1 + r (-1 + S)) (-k - ℒ) α2)))};

Solve[((r - r ℒ) (-1 + k + r - k r + ℒ - r ℒ) α2 ((-1 + k) (-1 + β1) - λ0) +
    (1 - k - r + k r - ℒ + r ℒ - λ0) (1 - ℒ - α2 + r α2 + ℒ α2 - r ℒ α2 - λ0) ((-1 + k) (-1 + β1) - λ0)) == 0, λ0];
```

In[•]:= `Simplify[%]`

Out[•]=

$$\left\{\left\{\lambda_0 \to \frac{1}{2}\left(2 - k - r + k r - 2\mathcal{L} + r\mathcal{L} - \alpha_2 + r\alpha_2 + \mathcal{L}\alpha_2 - r\mathcal{L}\alpha_2 - \sqrt{-4(-1+r)(-1+\mathcal{L})(-1+k+\mathcal{L})(-1+\alpha_2) + \left(k(-1+r) + (-2+r)(-1+\mathcal{L}) + (-1+r+\mathcal{L}-r\mathcal{L})\alpha_2\right)^2}\right)\right\},\right.$$

$$\left\{\lambda_0 \to \frac{1}{2}\left(2 - k - r + k r - 2\mathcal{L} + r\mathcal{L} - \alpha_2 + r\alpha_2 + \mathcal{L}\alpha_2 - r\mathcal{L}\alpha_2 + \sqrt{-4(-1+r)(-1+\mathcal{L})(-1+k+\mathcal{L})(-1+\alpha_2) + \left(k(-1+r) + (-2+r)(-1+\mathcal{L}) + (-1+r+\mathcal{L}-r\mathcal{L})\alpha_2\right)^2}\right)\right\},$$

$$\left.\left\{\lambda_0 \to (-1+k)(-1+\beta_1)\right\}\right\}$$

Plugging each of the zeroth order eigenvalues into the first order term and solving for the corresponding first order term for each eigenvalue give the full first order approximation. For numerical parameter values evaluated in the main text and File S1 for case B, the eigenvalues suggested local stability of the presented equilibria such that $|\lambda_i| < 1$.

Calculations of the first order terms of the eigenvalues

Solving for first eigenvalue approximated to first order:

```
{(-((-k (1 - k - r + k r - ℒ + r ℒ) ((z (-1 + (1 + r (-1 + S)) (1 - k - ℒ)) α2) / (-1 + (1 + r (-1 + S)) (1 - k - ℒ) + (1 - ℒ)
                (1 + (-1 + r - r S) (1 - k - ℒ) + (1 + r (-1 + S)) (-k - ℒ) α2)) - ((1 + r (-1 + S)) z (1 - k - ℒ) α2) /
              (-1 + (1 + r (-1 + S)) (1 - k - ℒ) + (1 - ℒ) (1 + (-1 + r - r S) (1 - k - ℒ) + (1 + r (-1 + S)) (-k - ℒ) α2)))
           (-1 + β1) - (z - k z + (r z (-1 + (1 + r (-1 + S)) (1 - k - ℒ)) (1 - k - ℒ)) /
              (-1 + (1 + r (-1 + S)) (1 - k - ℒ) + (1 - ℒ) (1 + (-1 + r - r S) (1 - k - ℒ) + (1 + r (-1 + S)) (-k - ℒ) α2)) -
             (r (1 + r (-1 + S)) z (1 - k - ℒ)^2 α2) / (-1 + (1 + r (-1 + S)) (1 - k - ℒ) +
                (1 - ℒ) (1 + (-1 + r - r S) (1 - k - ℒ) + (1 + r (-1 + S)) (-k - ℒ) α2))) (-1 + β1))
       (-((r - k r) (r - r ℒ) α2) + (r - k r) (1 - ℒ - α2 + r α2 + ℒ α2 - r ℒ α2 - λ0))) - (r - r ℒ)
     (-r (-1 + k + ℒ) α1 + r (1 - k - ℒ) ((z (-1 + (1 + r (-1 + S)) (1 - k - ℒ)) α2) / (-1 + (1 + r (-1 + S)) (1 - k - ℒ) +
              (1 - ℒ) (1 + (-1 + r - r S) (1 - k - ℒ) + (1 + r (-1 + S)) (-k - ℒ) α2)) - ((1 + r (-1 + S)) z (1 - k - ℒ) α2) /
           (-1 + (1 + r (-1 + S)) (1 - k - ℒ) + (1 - ℒ) (1 + (-1 + r - r S) (1 - k - ℒ) + (1 + r (-1 + S)) (-k - ℒ) α2))) +
       (1 - k - r + k r - ℒ + r ℒ) (ℒ + k α2) ((z (-1 + (1 + r (-1 + S)) (1 - k - ℒ)) α2) / (-1 + (1 + r (-1 + S)) (1 - k - ℒ) +
              (1 - ℒ) (1 + (-1 + r - r S) (1 - k - ℒ) + (1 + r (-1 + S)) (-k - ℒ) α2)) - ((1 + r (-1 + S)) z (1 - k - ℒ) α2) /
           (-1 + (1 + r (-1 + S)) (1 - k - ℒ) + (1 - ℒ) (1 + (-1 + r - r S) (1 - k - ℒ) + (1 + r (-1 + S)) (-k - ℒ) α2))) -
```

$$\alpha_2 (y - k y - r y + k r y - r z + k r z - y \mathcal{L} + r y \mathcal{L} - (r z (-1 + (1 + r (-1 + S)) (1 - k - \mathcal{L})) (1 - k - \mathcal{L})) /$$
$$(-1 + (1 + r (-1 + S)) (1 - k - \mathcal{L}) + (1 - \mathcal{L}) (1 + (-1 + r - r S) (1 - k - \mathcal{L}) + (1 + r (-1 + S)) (-k - \mathcal{L}) \alpha_2)) +$$
$$(r (1 + r (-1 + S)) z (1 - k - \mathcal{L})^2 \alpha_2) / (-1 + (1 + r (-1 + S)) (1 - k - \mathcal{L}) +$$
$$(1 - \mathcal{L}) (1 + (-1 + r - r S) (1 - k - \mathcal{L}) + (1 + r (-1 + S)) (-k - \mathcal{L}) \alpha_2))) -$$
$$(-1 + k + r - k r + \mathcal{L} - r \mathcal{L}) \alpha_2 (k \alpha_1 + (z (-1 + (1 + r (-1 + S)) (1 - k - \mathcal{L})) \mathcal{L}) /$$
$$(-1 + (1 + r (-1 + S)) (1 - k - \mathcal{L}) + (1 - \mathcal{L}) (1 + (-1 + r - r S) (1 - k - \mathcal{L}) + (1 + r (-1 + S)) (-k - \mathcal{L}) \alpha_2)) +$$
$$(k z (-1 + (1 + r (-1 + S)) (1 - k - \mathcal{L})) \alpha_2) / (-1 + (1 + r (-1 + S)) (1 - k - \mathcal{L}) + (1 - \mathcal{L})$$
$$(1 + (-1 + r - r S) (1 - k - \mathcal{L}) + (1 + r (-1 + S)) (-k - \mathcal{L}) \alpha_2)) - (k (1 + r (-1 + S)) z (1 - k - \mathcal{L}) \alpha_2) /$$
$$(-1 + (1 + r (-1 + S)) (1 - k - \mathcal{L}) + (1 - \mathcal{L}) (1 + (-1 + r - r S) (1 - k - \mathcal{L}) + (1 + r (-1 + S)) (-k - \mathcal{L}) \alpha_2)) -$$
$$((1 + r (-1 + S)) z (1 - k - \mathcal{L}) \mathcal{L} \alpha_2) / (-1 + (1 + r (-1 + S)) (1 - k - \mathcal{L}) +$$
$$(1 - \mathcal{L}) (1 + (-1 + r - r S) (1 - k - \mathcal{L}) + (1 + r (-1 + S)) (-k - \mathcal{L}) \alpha_2)) -$$
$$((1 - k) k (r (-1 + S) z \alpha_2 + \alpha_1 (-k - r (1 - k - \mathcal{L}) + r S (1 - k - \mathcal{L}) - \mathcal{L} + (1 - \mathcal{L}) (1 + (-1 + r - r S)$$
$$(1 - k - \mathcal{L}) + (1 + r (-1 + S)) (-k - \mathcal{L}) \alpha_2))) (-1 + \beta_1)) / ((-1 + (1 + r (-1 + S)) (1 - k - \mathcal{L}) +$$
$$(1 - \mathcal{L}) (1 + (-1 + r - r S) (1 - k - \mathcal{L}) + (1 + r (-1 + S)) (-k - \mathcal{L}) \alpha_2)) (1 + (1 - k) (-1 + \beta_1)))) -$$
$$((1 - k) r (1 - k - \mathcal{L}) (r (-1 + S) z \alpha_2 + \alpha_1 (-k - r (1 - k - \mathcal{L}) + r S (1 - k - \mathcal{L}) - \mathcal{L} +$$
$$(1 - \mathcal{L}) (1 + (-1 + r - r S) (1 - k - \mathcal{L}) + (1 + r (-1 + S)) (-k - \mathcal{L}) \alpha_2))) (-1 + \beta_1)) /$$
$$((-1 + (1 + r (-1 + S)) (1 - k - \mathcal{L}) + (1 - \mathcal{L}) (1 + (-1 + r - r S) (1 - k - \mathcal{L}) + (1 + r (-1 + S)) (-k - \mathcal{L}) \alpha_2))$$
$$(1 + (1 - k) (-1 + \beta_1)))) ((-1 + k) (-1 + \beta_1) - \lambda_0) -$$
$$(-1 + k + r - k r + \mathcal{L} - r \mathcal{L}) \alpha_2 ((r - k r) ((z - (r z (-1 + (1 + r (-1 + S)) (1 - k - \mathcal{L})) (-1 + \mathcal{L})) / (-1 +$$
$$(1 + r (-1 + S)) (1 - k - \mathcal{L}) + (1 - \mathcal{L}) (1 + (-1 + r - r S) (1 - k - \mathcal{L}) + (1 + r (-1 + S)) (-k - \mathcal{L}) \alpha_2)) +$$
$$(r (1 + r (-1 + S)) z (1 - k - \mathcal{L}) (-1 + \mathcal{L}) \alpha_2) / (-1 + (1 + r (-1 + S)) (1 - k - \mathcal{L}) +$$
$$(1 - \mathcal{L}) (1 + (-1 + r - r S) (1 - k - \mathcal{L}) + (1 + r (-1 + S)) (-k - \mathcal{L}) \alpha_2))) (-1 + \beta_1) -$$
$$k (z + (z (-1 + (1 + r (-1 + S)) (1 - k - \mathcal{L}))) / (-1 + (1 + r (-1 + S)) (1 - k - \mathcal{L}) + (1 - \mathcal{L}) (1 + (-1 + r - r S)$$
$$(1 - k - \mathcal{L}) + (1 + r (-1 + S)) (-k - \mathcal{L}) \alpha_2)) - (z (-1 + (1 + r (-1 + S)) (1 - k - \mathcal{L})) \mathcal{L}) / (-1 +$$
$$(1 + r (-1 + S)) (1 - k - \mathcal{L}) + (1 - \mathcal{L}) (1 + (-1 + r - r S) (1 - k - \mathcal{L}) + (1 + r (-1 + S)) (-k - \mathcal{L}) \alpha_2)) -$$
$$(z (-1 + (1 + r (-1 + S)) (1 - k - \mathcal{L})) \alpha_2) / (-1 + (1 + r (-1 + S)) (1 - k - \mathcal{L}) +$$
$$(1 - \mathcal{L}) (1 + (-1 + r - r S) (1 - k - \mathcal{L}) + (1 + r (-1 + S)) (-k - \mathcal{L}) \alpha_2)) +$$
$$(r z (-1 + (1 + r (-1 + S)) (1 - k - \mathcal{L})) \alpha_2) / (-1 + (1 + r (-1 + S)) (1 - k - \mathcal{L}) +$$
$$(1 - \mathcal{L}) (1 + (-1 + r - r S) (1 - k - \mathcal{L}) + (1 + r (-1 + S)) (-k - \mathcal{L}) \alpha_2)) -$$
$$(r (1 + r (-1 + S)) z (1 - k - \mathcal{L}) \alpha_2) / (-1 + (1 + r (-1 + S)) (1 - k - \mathcal{L}) +$$
$$(1 - \mathcal{L}) (1 + (-1 + r - r S) (1 - k - \mathcal{L}) + (1 + r (-1 + S)) (-k - \mathcal{L}) \alpha_2)) +$$
$$(z (-1 + (1 + r (-1 + S)) (1 - k - \mathcal{L})) \mathcal{L} \alpha_2) / (-1 + (1 + r (-1 + S)) (1 - k - \mathcal{L}) +$$
$$(1 - \mathcal{L}) (1 + (-1 + r - r S) (1 - k - \mathcal{L}) + (1 + r (-1 + S)) (-k - \mathcal{L}) \alpha_2)) -$$
$$(r z (-1 + (1 + r (-1 + S)) (1 - k - \mathcal{L})) \mathcal{L} \alpha_2) / (-1 + (1 + r (-1 + S)) (1 - k - \mathcal{L}) +$$
$$(1 - \mathcal{L}) (1 + (-1 + r - r S) (1 - k - \mathcal{L}) + (1 + r (-1 + S)) (-k - \mathcal{L}) \alpha_2)) +$$
$$(r (1 + r (-1 + S)) z (1 - k - \mathcal{L}) \mathcal{L} \alpha_2) / (-1 + (1 + r (-1 + S)) (1 - k - \mathcal{L}) +$$
$$(1 - \mathcal{L}) (1 + (-1 + r - r S) (1 - k - \mathcal{L}) + (1 + r (-1 + S)) (-k - \mathcal{L}) \alpha_2))) (-1 + \beta_1)) -$$
$$(-r y - z - r z + r y \mathcal{L} + r (-1 + \mathcal{L}) \alpha_1 - (r z (-1 + (1 + r (-1 + S)) (1 - k - \mathcal{L}))) /$$

$$\left(-1+\left(1+r\,(-1+S)\right)(1-k-\mathcal{L})+(1-\mathcal{L})\left(1+\left(-1+r-r\,S\right)(1-k-\mathcal{L})+\left(1+r\,(-1+S)\right)(-k-\mathcal{L})\,\alpha_2\right)\right)+$$

$$\left(r\,z\left(-1+\left(1+r\,(-1+S)\right)(1-k-\mathcal{L})\right)\mathcal{L}\right)\Big/\left(-1+\left(1+r\,(-1+S)\right)(1-k-\mathcal{L})+\right.$$

$$\left.(1-\mathcal{L})\left(1+\left(-1+r-r\,S\right)(1-k-\mathcal{L})+\left(1+r\,(-1+S)\right)(-k-\mathcal{L})\,\alpha_2\right)\right)-$$

$$\left(r\,z\left(-1+\left(1+r\,(-1+S)\right)(1-k-\mathcal{L})\right)\alpha_2\right)\Big/\left(-1+\left(1+r\,(-1+S)\right)(1-k-\mathcal{L})+(1-\mathcal{L})\right.$$

$$\left.\left(1+\left(-1+r-r\,S\right)(1-k-\mathcal{L})+\left(1+r\,(-1+S)\right)(-k-\mathcal{L})\,\alpha_2\right)\right)+\left(2\,r\left(1+r\,(-1+S)\right)z\,(1-k-\mathcal{L})\,\alpha_2\right)\Big/$$

$$\left(-1+\left(1+r\,(-1+S)\right)(1-k-\mathcal{L})+(1-\mathcal{L})\left(1+\left(-1+r-r\,S\right)(1-k-\mathcal{L})+\left(1+r\,(-1+S)\right)(-k-\mathcal{L})\,\alpha_2\right)\right)+$$

$$\left(r\,z\left(-1+\left(1+r\,(-1+S)\right)(1-k-\mathcal{L})\right)\mathcal{L}\,\alpha_2\right)\Big/\left(-1+\left(1+r\,(-1+S)\right)(1-k-\mathcal{L})+\right.$$

$$\left.(1-\mathcal{L})\left(1+\left(-1+r-r\,S\right)(1-k-\mathcal{L})+\left(1+r\,(-1+S)\right)(-k-\mathcal{L})\,\alpha_2\right)\right)-$$

$$\left(2\,r\left(1+r\,(-1+S)\right)z\,(1-k-\mathcal{L})\,\mathcal{L}\,\alpha_2\right)\Big/\left(-1+\left(1+r\,(-1+S)\right)(1-k-\mathcal{L})+\right.$$

$$\left.(1-\mathcal{L})\left(1+\left(-1+r-r\,S\right)(1-k-\mathcal{L})+\left(1+r\,(-1+S)\right)(-k-\mathcal{L})\,\alpha_2\right)\right)-$$

$$(-k-\mathcal{L})\left(z+\left(z\left(-1+\left(1+r\,(-1+S)\right)(1-k-\mathcal{L})\right)\right)\Big/\left(-1+\left(1+r\,(-1+S)\right)(1-k-\mathcal{L})+\right.\right.$$

$$\left.(1-\mathcal{L})\left(1+\left(-1+r-r\,S\right)(1-k-\mathcal{L})+\left(1+r\,(-1+S)\right)(-k-\mathcal{L})\,\alpha_2\right)\right)-$$

$$\left(z\left(-1+\left(1+r\,(-1+S)\right)(1-k-\mathcal{L})\right)\mathcal{L}\right)\Big/\left(-1+\left(1+r\,(-1+S)\right)(1-k-\mathcal{L})+\right.$$

$$\left.(1-\mathcal{L})\left(1+\left(-1+r-r\,S\right)(1-k-\mathcal{L})+\left(1+r\,(-1+S)\right)(-k-\mathcal{L})\,\alpha_2\right)\right)-$$

$$\left(z\left(-1+\left(1+r\,(-1+S)\right)(1-k-\mathcal{L})\right)\alpha_2\right)\Big/\left(-1+\left(1+r\,(-1+S)\right)(1-k-\mathcal{L})+\right.$$

$$\left.(1-\mathcal{L})\left(1+\left(-1+r-r\,S\right)(1-k-\mathcal{L})+\left(1+r\,(-1+S)\right)(-k-\mathcal{L})\,\alpha_2\right)\right)+$$

$$\left(r\,z\left(-1+\left(1+r\,(-1+S)\right)(1-k-\mathcal{L})\right)\alpha_2\right)\Big/\left(-1+\left(1+r\,(-1+S)\right)(1-k-\mathcal{L})+\right.$$

$$\left.(1-\mathcal{L})\left(1+\left(-1+r-r\,S\right)(1-k-\mathcal{L})+\left(1+r\,(-1+S)\right)(-k-\mathcal{L})\,\alpha_2\right)\right)-$$

$$\left(r\left(1+r\,(-1+S)\right)z\,(1-k-\mathcal{L})\,\alpha_2\right)\Big/\left(-1+\left(1+r\,(-1+S)\right)(1-k-\mathcal{L})+\right.$$

$$\left.(1-\mathcal{L})\left(1+\left(-1+r-r\,S\right)(1-k-\mathcal{L})+\left(1+r\,(-1+S)\right)(-k-\mathcal{L})\,\alpha_2\right)\right)+$$

$$\left(z\left(-1+\left(1+r\,(-1+S)\right)(1-k-\mathcal{L})\right)\mathcal{L}\,\alpha_2\right)\Big/\left(-1+\left(1+r\,(-1+S)\right)(1-k-\mathcal{L})+\right.$$

$$\left.(1-\mathcal{L})\left(1+\left(-1+r-r\,S\right)(1-k-\mathcal{L})+\left(1+r\,(-1+S)\right)(-k-\mathcal{L})\,\alpha_2\right)\right)-$$

$$\left(r\,z\left(-1+\left(1+r\,(-1+S)\right)(1-k-\mathcal{L})\right)\mathcal{L}\,\alpha_2\right)\Big/\left(-1+\left(1+r\,(-1+S)\right)(1-k-\mathcal{L})+\right.$$

$$\left.(1-\mathcal{L})\left(1+\left(-1+r-r\,S\right)(1-k-\mathcal{L})+\left(1+r\,(-1+S)\right)(-k-\mathcal{L})\,\alpha_2\right)\right)+$$

$$\left(r\left(1+r\,(-1+S)\right)z\,(1-k-\mathcal{L})\,\mathcal{L}\,\alpha_2\right)\Big/\left(-1+\left(1+r\,(-1+S)\right)(1-k-\mathcal{L})+\right.$$

$$\left.\left.(1-\mathcal{L})\left(1+\left(-1+r-r\,S\right)(1-k-\mathcal{L})+\left(1+r\,(-1+S)\right)(-k-\mathcal{L})\,\alpha_2\right)\right)\right)-$$

$$\left(r-r\,\mathcal{L}\right)\left(-k\,\alpha_1-\left(z\left(-1+\left(1+r\,(-1+S)\right)(1-k-\mathcal{L})\right)\mathcal{L}\right)\Big/\left(-1+\left(1+r\,(-1+S)\right)(1-k-\mathcal{L})+\right.\right.$$

$$\left.(1-\mathcal{L})\left(1+\left(-1+r-r\,S\right)(1-k-\mathcal{L})+\left(1+r\,(-1+S)\right)(-k-\mathcal{L})\,\alpha_2\right)\right)-$$

$$\left(k\,z\left(-1+\left(1+r\,(-1+S)\right)(1-k-\mathcal{L})\right)\alpha_2\right)\Big/\left(-1+\left(1+r\,(-1+S)\right)(1-k-\mathcal{L})+\right.$$

$$\left.(1-\mathcal{L})\left(1+\left(-1+r-r\,S\right)(1-k-\mathcal{L})+\left(1+r\,(-1+S)\right)(-k-\mathcal{L})\,\alpha_2\right)\right)+$$

$$\left(k\left(1+r\,(-1+S)\right)z\,(1-k-\mathcal{L})\,\alpha_2\right)\Big/\left(-1+\left(1+r\,(-1+S)\right)(1-k-\mathcal{L})+\right.$$

$$\left.(1-\mathcal{L})\left(1+\left(-1+r-r\,S\right)(1-k-\mathcal{L})+\left(1+r\,(-1+S)\right)(-k-\mathcal{L})\,\alpha_2\right)\right)+$$

$$\left(\left(1+r\,(-1+S)\right)z\,(1-k-\mathcal{L})\,\mathcal{L}\,\alpha_2\right)\Big/\left(-1+\left(1+r\,(-1+S)\right)(1-k-\mathcal{L})+(1-\mathcal{L})\right.$$

$$\left.\left(1+\left(-1+r-r\,S\right)(1-k-\mathcal{L})+\left(1+r\,(-1+S)\right)(-k-\mathcal{L})\,\alpha_2\right)\right)+\left((1-k)\,k\left(r\,(-1+S)\,z\,\alpha_2+\right.\right.$$

$$\alpha_1\left(-k-r\,(1-k-\mathcal{L})+r\,S\,(1-k-\mathcal{L})-\mathcal{L}+(1-\mathcal{L})\left(1+\left(-1+r-r\,S\right)(1-k-\mathcal{L})+\right.\right.$$

$$\left.\left.\left.\left.\left(1+r\,(-1+S)\right)(-k-\mathcal{L})\,\alpha_2\right)\right)\right)(-1+\beta_1)\right)\Big/\left(\left(-1+\left(1+r\,(-1+S)\right)(1-k-\mathcal{L})+(1-\mathcal{L})\right.\right.$$

$$\left.\left.\left.\left(1+\left(-1+r-r\,S\right)(1-k-\mathcal{L})+\left(1+r\,(-1+S)\right)(-k-\mathcal{L})\,\alpha_2\right)\right)\left(1+(1-k)\,(-1+\beta_1)\right)\right)\right)-$$

$$\left((1-k)\,r\,(-1+\mathcal{L})\left(r\,(-1+S)\,z\,\alpha_2+\alpha_1\left(-k-r\,(1-k-\mathcal{L})+r\,S\,(1-k-\mathcal{L})-\mathcal{L}+(1-\mathcal{L})\right.\right.\right.$$

$$\left(1+\left(-1+r-r S\right) (1-k-\mathcal{L})+\left(1+r (-1+S)\right) (-k-\mathcal{L}) \alpha_2\right)\right)\right) (-1+\beta_1)\right) /$$
$$\left(\left(-1+\left(1+r (-1+S)\right) (1-k-\mathcal{L})+(1-\mathcal{L}) \left(1+\left(-1+r-r S\right) (1-k-\mathcal{L})+\left(1+r (-1+S)\right) (-k-\mathcal{L}) \alpha_2\right)\right)\right.$$
$$\left(1+(1-k) (-1+\beta_1)\right)\right)+\beta_2-r \beta_2-\mathcal{L} \beta_2+r \mathcal{L} \beta_2\right) \left((-1+k) (-1+\beta_1)-\lambda_0\right)-$$
$$\left(r-r \mathcal{L}\right) \left((-1+k) \alpha_1+\left(z-k z+\left((1-k) r z \left(-1+\left(1+r (-1+S)\right) (1-k-\mathcal{L})\right)\right) / \left(-1+\right.\right.\right.$$
$$\left(1+r (-1+S)\right) (1-k-\mathcal{L})+(1-\mathcal{L}) \left(1+\left(-1+r-r S\right) (1-k-\mathcal{L})+\left(1+r (-1+S)\right) (-k-\mathcal{L}) \alpha_2\right)\right)-$$
$$\left((1-k) r \left(1+r (-1+S)\right) z (1-k-\mathcal{L}) \alpha_2\right) / \left(-1+\left(1+r (-1+S)\right) (1-k-\mathcal{L})+\right.$$
$$(1-\mathcal{L}) \left(1+\left(-1+r-r S\right) (1-k-\mathcal{L})+\left(1+r (-1+S)\right) (-k-\mathcal{L}) \alpha_2\right)\right)\right) (-1+\beta_1)-$$
$$k \left(-\left((-1+k) \alpha_1\right)-\left(-r+k r\right) \left(\left(z \left(-1+\left(1+r (-1+S)\right) (1-k-\mathcal{L})\right) \alpha_2\right) / \left(-1+\left(1+r (-1+S)\right)\right.\right.\right.$$
$$(1-k-\mathcal{L})+(1-\mathcal{L}) \left(1+\left(-1+r-r S\right) (1-k-\mathcal{L})+\left(1+r (-1+S)\right) (-k-\mathcal{L}) \alpha_2\right)\right)-$$
$$\left(\left(1+r (-1+S)\right) z (1-k-\mathcal{L}) \alpha_2\right) / \left(-1+\left(1+r (-1+S)\right) (1-k-\mathcal{L})+\right.$$
$$(1-\mathcal{L}) \left(1+\left(-1+r-r S\right) (1-k-\mathcal{L})+\left(1+r (-1+S)\right) (-k-\mathcal{L}) \alpha_2\right)\right)\right)+$$
$$\left((1-k) (-1+k) \left(r (-1+S) z \alpha_2+\alpha_1 \left(-k-r (1-k-\mathcal{L})+r S (1-k-\mathcal{L})-\mathcal{L}+\right.\right.\right.$$
$$(1-\mathcal{L}) \left(1+\left(-1+r-r S\right) (1-k-\mathcal{L})+\left(1+r (-1+S)\right) (-k-\mathcal{L}) \alpha_2\right)\right)\right)$$
$$(-1+\beta_1)\right) / \left(\left(-1+\left(1+r (-1+S)\right) (1-k-\mathcal{L})+(1-\mathcal{L}) \left(1+\left(-1+r-r S\right) (1-k-\mathcal{L})+\right.\right.\right.$$
$$\left(1+r (-1+S)\right) (-k-\mathcal{L}) \alpha_2\right)\right) \left(1+(1-k) (-1+\beta_1)\right)\right)\right)\right) (-1+\beta_1)-$$
$$(-1+k) \left(-k \alpha_1-\left(z \left(-1+\left(1+r (-1+S)\right) (1-k-\mathcal{L})\right) \mathcal{L}\right) / \left(-1+\left(1+r (-1+S)\right) (1-k-\mathcal{L})+\right.\right.$$
$$(1-\mathcal{L}) \left(1+\left(-1+r-r S\right) (1-k-\mathcal{L})+\left(1+r (-1+S)\right) (-k-\mathcal{L}) \alpha_2\right)\right)-$$
$$\left(k z \left(-1+\left(1+r (-1+S)\right) (1-k-\mathcal{L})\right) \alpha_2\right) / \left(-1+\left(1+r (-1+S)\right) (1-k-\mathcal{L})+\right.$$
$$(1-\mathcal{L}) \left(1+\left(-1+r-r S\right) (1-k-\mathcal{L})+\left(1+r (-1+S)\right) (-k-\mathcal{L}) \alpha_2\right)\right)+$$
$$\left(k \left(1+r (-1+S)\right) z (1-k-\mathcal{L}) \alpha_2\right) / \left(-1+\left(1+r (-1+S)\right) (1-k-\mathcal{L})+\right.$$
$$(1-\mathcal{L}) \left(1+\left(-1+r-r S\right) (1-k-\mathcal{L})+\left(1+r (-1+S)\right) (-k-\mathcal{L}) \alpha_2\right)\right)+$$
$$\left(\left(1+r (-1+S)\right) z (1-k-\mathcal{L}) \mathcal{L} \alpha_2\right) / \left(-1+\left(1+r (-1+S)\right) (1-k-\mathcal{L})+\right.$$
$$(1-\mathcal{L}) \left(1+\left(-1+r-r S\right) (1-k-\mathcal{L})+\left(1+r (-1+S)\right) (-k-\mathcal{L}) \alpha_2\right)\right)+$$
$$\left((1-k) k \left(r (-1+S) z \alpha_2+\alpha_1 \left(-k-r (1-k-\mathcal{L})+r S (1-k-\mathcal{L})-\mathcal{L}+(1-\mathcal{L})\right.\right.\right.$$
$$\left(1+\left(-1+r-r S\right) (1-k-\mathcal{L})+\left(1+r (-1+S)\right) (-k-\mathcal{L}) \alpha_2\right)\right)\right) (-1+\beta_1)\right) /$$
$$\left(\left(-1+\left(1+r (-1+S)\right) (1-k-\mathcal{L})+(1-\mathcal{L}) \left(1+\left(-1+r-r S\right) (1-k-\mathcal{L})+\left(1+r (-1+S)\right)\right.\right.\right.$$
$$(-k-\mathcal{L}) \alpha_2\right)\right) \left(1+(1-k) (-1+\beta_1)\right)\right)\right)\right) (-1+\beta_1)-\lambda_1\right)\right)+\left(1-k-r+k r-\mathcal{L}+r \mathcal{L}-\lambda_0\right)$$
$$\left(-\left(\left(r-k r\right) \alpha_2 \left(\left(z-\left(r z \left(-1+\left(1+r (-1+S)\right) (1-k-\mathcal{L})\right) (-1+\mathcal{L})\right) / \left(-1+\left(1+r (-1+S)\right) (1-k-\mathcal{L})+\right.\right.\right.\right.\right.$$
$$(1-\mathcal{L}) \left(1+\left(-1+r-r S\right) (1-k-\mathcal{L})+\left(1+r (-1+S)\right) (-k-\mathcal{L}) \alpha_2\right)\right)+$$
$$\left(r \left(1+r (-1+S)\right) z (1-k-\mathcal{L}) (-1+\mathcal{L}) \alpha_2\right) / \left(-1+\left(1+r (-1+S)\right) (1-k-\mathcal{L})+\right.$$
$$(1-\mathcal{L}) \left(1+\left(-1+r-r S\right) (1-k-\mathcal{L})+\left(1+r (-1+S)\right) (-k-\mathcal{L}) \alpha_2\right)\right)\right) (-1+\beta_1)-$$
$$k \left(z+\left(z \left(-1+\left(1+r (-1+S)\right) (1-k-\mathcal{L})\right)\right) / \left(-1+\left(1+r (-1+S)\right) (1-k-\mathcal{L})+\right.\right.$$
$$(1-\mathcal{L}) \left(1+\left(-1+r-r S\right) (1-k-\mathcal{L})+\left(1+r (-1+S)\right) (-k-\mathcal{L}) \alpha_2\right)\right)-$$
$$\left(z \left(-1+\left(1+r (-1+S)\right) (1-k-\mathcal{L})\right) \mathcal{L}\right) / \left(-1+\left(1+r (-1+S)\right) (1-k-\mathcal{L})+\right.$$
$$(1-\mathcal{L}) \left(1+\left(-1+r-r S\right) (1-k-\mathcal{L})+\left(1+r (-1+S)\right) (-k-\mathcal{L}) \alpha_2\right)\right)-$$
$$\left(z \left(-1+\left(1+r (-1+S)\right) (1-k-\mathcal{L})\right) \alpha_2\right) / \left(-1+\left(1+r (-1+S)\right) (1-k-\mathcal{L})+\right.$$
$$(1-\mathcal{L}) \left(1+\left(-1+r-r S\right) (1-k-\mathcal{L})+\left(1+r (-1+S)\right) (-k-\mathcal{L}) \alpha_2\right)\right)+$$
$$\left(r z \left(-1+\left(1+r (-1+S)\right) (1-k-\mathcal{L})\right) \alpha_2\right) / \left(-1+\left(1+r (-1+S)\right) (1-k-\mathcal{L})+\right.$$

$$(1-\mathcal{L})\,(1+(-1+r-r\,S)\,(1-k-\mathcal{L})+(1+r\,(-1+S))\,(-k-\mathcal{L})\,\alpha_2))-$$
$$(r\,(1+r\,(-1+S))\,z\,(1-k-\mathcal{L})\,\alpha_2)\,/\,(-1+(1+r\,(-1+S))\,(1-k-\mathcal{L})+$$
$$(1-\mathcal{L})\,(1+(-1+r-r\,S)\,(1-k-\mathcal{L})+(1+r\,(-1+S))\,(-k-\mathcal{L})\,\alpha_2))+$$
$$(z\,(-1+(1+r\,(-1+S))\,(1-k-\mathcal{L}))\,\mathcal{L}\,\alpha_2)\,/\,(-1+(1+r\,(-1+S))\,(1-k-\mathcal{L})+$$
$$(1-\mathcal{L})\,(1+(-1+r-r\,S)\,(1-k-\mathcal{L})+(1+r\,(-1+S))\,(-k-\mathcal{L})\,\alpha_2))-$$
$$(r\,z\,(-1+(1+r\,(-1+S))\,(1-k-\mathcal{L}))\,\mathcal{L}\,\alpha_2)\,/\,(-1+(1+r\,(-1+S))\,(1-k-\mathcal{L})+$$
$$(1-\mathcal{L})\,(1+(-1+r-r\,S)\,(1-k-\mathcal{L})+(1+r\,(-1+S))\,(-k-\mathcal{L})\,\alpha_2))+$$
$$(r\,(1+r\,(-1+S))\,z\,(1-k-\mathcal{L})\,\mathcal{L}\,\alpha_2)\,/\,(-1+(1+r\,(-1+S))\,(1-k-\mathcal{L})+$$
$$(1-\mathcal{L})\,(1+(-1+r-r\,S)\,(1-k-\mathcal{L})+(1+r\,(-1+S))\,(-k-\mathcal{L})\,\alpha_2)))\,(-1+\beta_1)))+$$
$$((-1+k)\,(-1+\beta_1)-\lambda_0)\,(-y-z+y\,\mathcal{L}+r\,(-1+\mathcal{L})\,\alpha_1+y\,\alpha_2-r\,y\,\alpha_2-r\,z\,\alpha_2-y\,\mathcal{L}\,\alpha_2+$$
$$r\,y\,\mathcal{L}\,\alpha_2-(2\,r\,z\,(-1+(1+r\,(-1+S))\,(1-k-\mathcal{L}))\,\alpha_2)\,/$$
$$(-1+(1+r\,(-1+S))\,(1-k-\mathcal{L})+(1-\mathcal{L})\,(1+(-1+r-r\,S)\,(1-k-\mathcal{L})+(1+r\,(-1+S))\,(-k-\mathcal{L})\,\alpha_2))+$$
$$(r\,(1+r\,(-1+S))\,z\,(1-k-\mathcal{L})\,\alpha_2)\,/\,(-1+(1+r\,(-1+S))\,(1-k-\mathcal{L})+$$
$$(1-\mathcal{L})\,(1+(-1+r-r\,S)\,(1-k-\mathcal{L})+(1+r\,(-1+S))\,(-k-\mathcal{L})\,\alpha_2))+$$
$$(2\,r\,z\,(-1+(1+r\,(-1+S))\,(1-k-\mathcal{L}))\,\mathcal{L}\,\alpha_2)\,/\,(-1+(1+r\,(-1+S))\,(1-k-\mathcal{L})+$$
$$(1-\mathcal{L})\,(1+(-1+r-r\,S)\,(1-k-\mathcal{L})+(1+r\,(-1+S))\,(-k-\mathcal{L})\,\alpha_2))-$$
$$(r\,(1+r\,(-1+S))\,z\,(1-k-\mathcal{L})\,\mathcal{L}\,\alpha_2)\,/\,(-1+(1+r\,(-1+S))\,(1-k-\mathcal{L})+(1-\mathcal{L})$$
$$(1+(-1+r-r\,S)\,(1-k-\mathcal{L})+(1+r\,(-1+S))\,(-k-\mathcal{L})\,\alpha_2))+(r\,(1+r\,(-1+S))\,z\,(1-k-\mathcal{L})\,\alpha_2^2)\,/$$
$$(-1+(1+r\,(-1+S))\,(1-k-\mathcal{L})+(1-\mathcal{L})\,(1+(-1+r-r\,S)\,(1-k-\mathcal{L})+(1+r\,(-1+S))\,(-k-\mathcal{L})\,\alpha_2))-$$
$$(r\,(1+r\,(-1+S))\,z\,(1-k-\mathcal{L})\,\mathcal{L}\,\alpha_2^2)\,/\,(-1+(1+r\,(-1+S))\,(1-k-\mathcal{L})+$$
$$(1-\mathcal{L})\,(1+(-1+r-r\,S)\,(1-k-\mathcal{L})+(1+r\,(-1+S))\,(-k-\mathcal{L})\,\alpha_2))-$$
$$(-\mathcal{L}-k\,\alpha_2)\,(z+(z\,(-1+(1+r\,(-1+S))\,(1-k-\mathcal{L})))\,/\,(-1+(1+r\,(-1+S))\,(1-k-\mathcal{L})+$$
$$(1-\mathcal{L})\,(1+(-1+r-r\,S)\,(1-k-\mathcal{L})+(1+r\,(-1+S))\,(-k-\mathcal{L})\,\alpha_2))-$$
$$(z\,(-1+(1+r\,(-1+S))\,(1-k-\mathcal{L}))\,\mathcal{L})\,/\,(-1+(1+r\,(-1+S))\,(1-k-\mathcal{L})+$$
$$(1-\mathcal{L})\,(1+(-1+r-r\,S)\,(1-k-\mathcal{L})+(1+r\,(-1+S))\,(-k-\mathcal{L})\,\alpha_2))-$$
$$(z\,(-1+(1+r\,(-1+S))\,(1-k-\mathcal{L}))\,\alpha_2)\,/\,(-1+(1+r\,(-1+S))\,(1-k-\mathcal{L})+$$
$$(1-\mathcal{L})\,(1+(-1+r-r\,S)\,(1-k-\mathcal{L})+(1+r\,(-1+S))\,(-k-\mathcal{L})\,\alpha_2))+$$
$$(r\,z\,(-1+(1+r\,(-1+S))\,(1-k-\mathcal{L}))\,\alpha_2)\,/\,(-1+(1+r\,(-1+S))\,(1-k-\mathcal{L})+$$
$$(1-\mathcal{L})\,(1+(-1+r-r\,S)\,(1-k-\mathcal{L})+(1+r\,(-1+S))\,(-k-\mathcal{L})\,\alpha_2))-$$
$$(r\,(1+r\,(-1+S))\,z\,(1-k-\mathcal{L})\,\alpha_2)\,/\,(-1+(1+r\,(-1+S))\,(1-k-\mathcal{L})+$$
$$(1-\mathcal{L})\,(1+(-1+r-r\,S)\,(1-k-\mathcal{L})+(1+r\,(-1+S))\,(-k-\mathcal{L})\,\alpha_2))+$$
$$(z\,(-1+(1+r\,(-1+S))\,(1-k-\mathcal{L}))\,\mathcal{L}\,\alpha_2)\,/\,(-1+(1+r\,(-1+S))\,(1-k-\mathcal{L})+$$
$$(1-\mathcal{L})\,(1+(-1+r-r\,S)\,(1-k-\mathcal{L})+(1+r\,(-1+S))\,(-k-\mathcal{L})\,\alpha_2))-$$
$$(r\,z\,(-1+(1+r\,(-1+S))\,(1-k-\mathcal{L}))\,\mathcal{L}\,\alpha_2)\,/\,(-1+(1+r\,(-1+S))\,(1-k-\mathcal{L})+$$
$$(1-\mathcal{L})\,(1+(-1+r-r\,S)\,(1-k-\mathcal{L})+(1+r\,(-1+S))\,(-k-\mathcal{L})\,\alpha_2))+$$
$$(r\,(1+r\,(-1+S))\,z\,(1-k-\mathcal{L})\,\mathcal{L}\,\alpha_2)\,/\,(-1+(1+r\,(-1+S))\,(1-k-\mathcal{L})+$$
$$(1-\mathcal{L})\,(1+(-1+r-r\,S)\,(1-k-\mathcal{L})+(1+r\,(-1+S))\,(-k-\mathcal{L})\,\alpha_2)))-$$
$$(1-\mathcal{L}-\alpha_2+r\,\alpha_2+\mathcal{L}\,\alpha_2-r\,\mathcal{L}\,\alpha_2)\,(-k\,\alpha_1-(z\,(-1+(1+r\,(-1+S))\,(1-k-\mathcal{L}))\,\mathcal{L})\,/\,(-1+$$

$$(1 + r (-1 + S)) (1 - k - \mathcal{L}) + (1 - \mathcal{L}) (1 + (-1 + r - r S) (1 - k - \mathcal{L}) + (1 + r (-1 + S)) (-k - \mathcal{L}) \alpha_2)) -$$
$$(k z (-1 + (1 + r (-1 + S)) (1 - k - \mathcal{L})) \alpha_2) / (-1 + (1 + r (-1 + S)) (1 - k - \mathcal{L}) +$$
$$(1 - \mathcal{L}) (1 + (-1 + r - r S) (1 - k - \mathcal{L}) + (1 + r (-1 + S)) (-k - \mathcal{L}) \alpha_2)) +$$
$$(k (1 + r (-1 + S)) z (1 - k - \mathcal{L}) \alpha_2) / (-1 + (1 + r (-1 + S)) (1 - k - \mathcal{L}) +$$
$$(1 - \mathcal{L}) (1 + (-1 + r - r S) (1 - k - \mathcal{L}) + (1 + r (-1 + S)) (-k - \mathcal{L}) \alpha_2)) +$$
$$((1 + r (-1 + S)) z (1 - k - \mathcal{L}) \mathcal{L} \alpha_2) / (-1 + (1 + r (-1 + S)) (1 - k - \mathcal{L}) +$$
$$(1 - \mathcal{L}) (1 + (-1 + r - r S) (1 - k - \mathcal{L}) + (1 + r (-1 + S)) (-k - \mathcal{L}) \alpha_2)) +$$
$$((1 - k) k (r (-1 + S) z \alpha_2 + \alpha_1 (-k - r (1 - k - \mathcal{L}) + r S (1 - k - \mathcal{L}) - \mathcal{L} + (1 - \mathcal{L})$$
$$(1 + (-1 + r - r S) (1 - k - \mathcal{L}) + (1 + r (-1 + S)) (-k - \mathcal{L}) \alpha_2)))$$
$$(-1 + \beta_1)) / ((-1 + (1 + r (-1 + S)) (1 - k - \mathcal{L}) + (1 - \mathcal{L}) (1 + (-1 + r - r S) (1 - k - \mathcal{L}) +$$
$$(1 + r (-1 + S)) (-k - \mathcal{L}) \alpha_2)) (1 + (1 - k) (-1 + \beta_1)))) -$$
$$((1 - k) r (-1 + \mathcal{L}) (r (-1 + S) z \alpha_2 + \alpha_1 (-k - r (1 - k - \mathcal{L}) + r S (1 - k - \mathcal{L}) - \mathcal{L} + (1 - \mathcal{L})$$
$$(1 + (-1 + r - r S) (1 - k - \mathcal{L}) + (1 + r (-1 + S)) (-k - \mathcal{L}) \alpha_2))) (-1 + \beta_1)) /$$
$$((-1 + (1 + r (-1 + S)) (1 - k - \mathcal{L}) + (1 - \mathcal{L}) (1 + (-1 + r - r S) (1 - k - \mathcal{L}) + (1 + r (-1 + S)) (-k - \mathcal{L}) \alpha_2))$$
$$(1 + (1 - k) (-1 + \beta_1))) - \lambda_1) + (1 - \mathcal{L} - \alpha_2 + r \alpha_2 + \mathcal{L} \alpha_2 - r \mathcal{L} \alpha_2 - \lambda_0)$$
$$((-1 + k) \alpha_1 + (z - k z + ((1 - k) r z (-1 + (1 + r (-1 + S)) (1 - k - \mathcal{L}))) / (-1 + (1 + r (-1 + S))$$
$$(1 - k - \mathcal{L}) + (1 - \mathcal{L}) (1 + (-1 + r - r S) (1 - k - \mathcal{L}) + (1 + r (-1 + S)) (-k - \mathcal{L}) \alpha_2)) -$$
$$((1 - k) r (1 + r (-1 + S)) z (1 - k - \mathcal{L}) \alpha_2) / (-1 + (1 + r (-1 + S)) (1 - k - \mathcal{L}) +$$
$$(1 - \mathcal{L}) (1 + (-1 + r - r S) (1 - k - \mathcal{L}) + (1 + r (-1 + S)) (-k - \mathcal{L}) \alpha_2))) (-1 + \beta_1) -$$
$$k (-((-1 + k) \alpha_1) - (-r + k r) ((z (-1 + (1 + r (-1 + S)) (1 - k - \mathcal{L})) \alpha_2) / (-1 + (1 + r (-1 + S))$$
$$(1 - k - \mathcal{L}) + (1 - \mathcal{L}) (1 + (-1 + r - r S) (1 - k - \mathcal{L}) + (1 + r (-1 + S)) (-k - \mathcal{L}) \alpha_2)) -$$
$$((1 + r (-1 + S)) z (1 - k - \mathcal{L}) \alpha_2) / (-1 + (1 + r (-1 + S)) (1 - k - \mathcal{L}) +$$
$$(1 - \mathcal{L}) (1 + (-1 + r - r S) (1 - k - \mathcal{L}) + (1 + r (-1 + S)) (-k - \mathcal{L}) \alpha_2))) +$$
$$((1 - k) (-1 + k) (r (-1 + S) z \alpha_2 + \alpha_1 (-k - r (1 - k - \mathcal{L}) + r S (1 - k - \mathcal{L}) - \mathcal{L} +$$
$$(1 - \mathcal{L}) (1 + (-1 + r - r S) (1 - k - \mathcal{L}) + (1 + r (-1 + S)) (-k - \mathcal{L}) \alpha_2)))$$
$$(-1 + \beta_1)) / ((-1 + (1 + r (-1 + S)) (1 - k - \mathcal{L}) + (1 - \mathcal{L}) (1 + (-1 + r - r S) (1 - k - \mathcal{L}) +$$
$$(1 + r (-1 + S)) (-k - \mathcal{L}) \alpha_2)) (1 + (1 - k) (-1 + \beta_1)))) (-1 + \beta_1) -$$
$$(-1 + k) (-k \alpha_1 - (z (-1 + (1 + r (-1 + S)) (1 - k - \mathcal{L})) \mathcal{L}) / (-1 + (1 + r (-1 + S)) (1 - k - \mathcal{L}) +$$
$$(1 - \mathcal{L}) (1 + (-1 + r - r S) (1 - k - \mathcal{L}) + (1 + r (-1 + S)) (-k - \mathcal{L}) \alpha_2)) -$$
$$(k z (-1 + (1 + r (-1 + S)) (1 - k - \mathcal{L})) \alpha_2) / (-1 + (1 + r (-1 + S)) (1 - k - \mathcal{L}) +$$
$$(1 - \mathcal{L}) (1 + (-1 + r - r S) (1 - k - \mathcal{L}) + (1 + r (-1 + S)) (-k - \mathcal{L}) \alpha_2)) +$$
$$(k (1 + r (-1 + S)) z (1 - k - \mathcal{L}) \alpha_2) / (-1 + (1 + r (-1 + S)) (1 - k - \mathcal{L}) +$$
$$(1 - \mathcal{L}) (1 + (-1 + r - r S) (1 - k - \mathcal{L}) + (1 + r (-1 + S)) (-k - \mathcal{L}) \alpha_2)) +$$
$$((1 + r (-1 + S)) z (1 - k - \mathcal{L}) \mathcal{L} \alpha_2) / (-1 + (1 + r (-1 + S)) (1 - k - \mathcal{L}) +$$
$$(1 - \mathcal{L}) (1 + (-1 + r - r S) (1 - k - \mathcal{L}) + (1 + r (-1 + S)) (-k - \mathcal{L}) \alpha_2)) +$$
$$((1 - k) k (r (-1 + S) z \alpha_2 + \alpha_1 (-k - r (1 - k - \mathcal{L}) + r S (1 - k - \mathcal{L}) - \mathcal{L} + (1 - \mathcal{L})$$
$$(1 + (-1 + r - r S) (1 - k - \mathcal{L}) + (1 + r (-1 + S)) (-k - \mathcal{L}) \alpha_2)))$$
$$(-1 + \beta_1)) / ((-1 + (1 + r (-1 + S)) (1 - k - \mathcal{L}) + (1 - \mathcal{L}) (1 + (-1 + r - r S) (1 - k - \mathcal{L}) +$$

$$\left(1 + r\,(-1 + S)\right)(-k - \mathcal{L})\,\alpha_2\big)\big)\left(1 + (1 - k)\,(-1 + \beta_1)\right)\big)\big)\,(-1 + \beta_1) - \lambda_1\big)\big) +$$

$$\left(1 - \mathcal{L} - \alpha_2 + r\,\alpha_2 + \mathcal{L}\,\alpha_2 - r\,\mathcal{L}\,\alpha_2 - \lambda_0\right)\left((-1 + k)\,(-1 + \beta_1) - \lambda_0\right)\big(-y + k\,y + r\,y -$$

$k\,r\,y +$

$r\,z -$

$k\,r\,z +$

$y\,\mathcal{L} -$

$r\,y\,\mathcal{L} -$

$r\,(-1 + k + \mathcal{L})\,\alpha_1 +$

$$\left(r\,z\left(-1 + \left(1 + r\,(-1 + S)\right)(1 - k - \mathcal{L})\right)(1 - k - \mathcal{L})\right)\Big/$$
$$\big(-1 + \left(1 + r\,(-1 + S)\right)(1 - k - \mathcal{L}) +$$
$$(1 - \mathcal{L})\left(1 + \left(-1 + r - r\,S\right)(1 - k - \mathcal{L}) + \left(1 + r\,(-1 + S)\right)(-k - \mathcal{L})\,\alpha_2\right)\big) -$$
$$\left(r\left(1 + r\,(-1 + S)\right)z\,(1 - k - \mathcal{L})^2\,\alpha_2\right)\Big/\big(-1 + \left(1 + r\,(-1 + S)\right)(1 - k - \mathcal{L}) +$$
$$(1 - \mathcal{L})\left(1 + \left(-1 + r - r\,S\right)(1 - k - \mathcal{L}) + \left(1 + r\,(-1 + S)\right)(-k - \mathcal{L})\,\alpha_2\right)\big) +$$
$$r\,(1 - k - \mathcal{L})\Big(\Big(z\left(-1 + \left(1 + r\,(-1 + S)\right)(1 - k - \mathcal{L})\right)\alpha_2\Big)\Big/\big(-1 + \left(1 + r\,(-1 + S)\right)(1 - k - \mathcal{L}) +$$
$$(1 - \mathcal{L})\left(1 + \left(-1 + r - r\,S\right)(1 - k - \mathcal{L}) + \left(1 + r\,(-1 + S)\right)(-k - \mathcal{L})\,\alpha_2\right)\big) - \Big(\left(1 + r\,(-1 + S)\right)z\,(1 - k - \mathcal{L})\,\alpha_2\Big)\Big/$$
$$\big(-1 + \left(1 + r\,(-1 + S)\right)(1 - k - \mathcal{L}) + (1 - \mathcal{L})\left(1 + \left(-1 + r - r\,S\right)(1 - k - \mathcal{L}) + \left(1 + r\,(-1 + S)\right)(-k - \mathcal{L})\,\alpha_2\right)\big)\Big) +$$
$$(k + \mathcal{L})\left(1 - k - r + k\,r - \mathcal{L} + r\,\mathcal{L}\right)\Big(\Big(z\left(-1 + \left(1 + r\,(-1 + S)\right)(1 - k - \mathcal{L})\right)\alpha_2\Big)\Big/$$
$$\big(-1 + \left(1 + r\,(-1 + S)\right)(1 - k - \mathcal{L}) + (1 - \mathcal{L})\left(1 + \left(-1 + r - r\,S\right)(1 - k - \mathcal{L}) + \left(1 + r\,(-1 + S)\right)(-k - \mathcal{L})\,\alpha_2\right)\big) -$$
$$\Big(\left(1 + r\,(-1 + S)\right)z\,(1 - k - \mathcal{L})\,\alpha_2\Big)\Big/\big(-1 + \left(1 + r\,(-1 + S)\right)(1 - k - \mathcal{L}) +$$
$$(1 - \mathcal{L})\left(1 + \left(-1 + r - r\,S\right)(1 - k - \mathcal{L}) + \left(1 + r\,(-1 + S)\right)(-k - \mathcal{L})\,\alpha_2\right)\big)\Big) -$$
$$\left(-1 + k + r - k\,r + \mathcal{L} - r\,\mathcal{L}\right)\Big(k\,\alpha_1 + \Big(z\left(-1 + \left(1 + r\,(-1 + S)\right)(1 - k - \mathcal{L})\right)\mathcal{L}\Big)\Big/$$
$$\big(-1 + \left(1 + r\,(-1 + S)\right)(1 - k - \mathcal{L}) + (1 - \mathcal{L})\left(1 + \left(-1 + r - r\,S\right)(1 - k - \mathcal{L}) + \left(1 + r\,(-1 + S)\right)(-k - \mathcal{L})\,\alpha_2\right)\big) +$$
$$\Big(k\,z\left(-1 + \left(1 + r\,(-1 + S)\right)(1 - k - \mathcal{L})\right)\alpha_2\Big)\Big/\big(-1 + \left(1 + r\,(-1 + S)\right)(1 - k - \mathcal{L}) +$$
$$(1 - \mathcal{L})\left(1 + \left(-1 + r - r\,S\right)(1 - k - \mathcal{L}) + \left(1 + r\,(-1 + S)\right)(-k - \mathcal{L})\,\alpha_2\right)\big) -$$
$$\Big(k\left(1 + r\,(-1 + S)\right)z\,(1 - k - \mathcal{L})\,\alpha_2\Big)\Big/\big(-1 + \left(1 + r\,(-1 + S)\right)(1 - k - \mathcal{L}) +$$
$$(1 - \mathcal{L})\left(1 + \left(-1 + r - r\,S\right)(1 - k - \mathcal{L}) + \left(1 + r\,(-1 + S)\right)(-k - \mathcal{L})\,\alpha_2\right)\big) -$$
$$\Big(\left(1 + r\,(-1 + S)\right)z\,(1 - k - \mathcal{L})\,\mathcal{L}\,\alpha_2\Big)\Big/\big(-1 + \left(1 + r\,(-1 + S)\right)(1 - k - \mathcal{L}) +$$
$$(1 - \mathcal{L})\left(1 + \left(-1 + r - r\,S\right)(1 - k - \mathcal{L}) + \left(1 + r\,(-1 + S)\right)(-k - \mathcal{L})\,\alpha_2\right)\big) -$$
$$\Big((1 - k)\,k\,\big(r\,(-1 + S)\,z\,\alpha_2 + \alpha_1\big(-k - r\,(1 - k - \mathcal{L}) + r\,S\,(1 - k - \mathcal{L}) - \mathcal{L} + (1 - \mathcal{L})$$
$$\left(1 + \left(-1 + r - r\,S\right)(1 - k - \mathcal{L}) + \left(1 + r\,(-1 + S)\right)(-k - \mathcal{L})\,\alpha_2\right)\big)\big)\,(-1 + \beta_1)\Big)\Big/$$
$$\Big(\big(-1 + \left(1 + r\,(-1 + S)\right)(1 - k - \mathcal{L}) + (1 - \mathcal{L})\left(1 + \left(-1 + r - r\,S\right)(1 - k - \mathcal{L}) + \left(1 + r\,(-1 + S)\right)(-k - \mathcal{L})\,\alpha_2\right)\big)$$
$$\left(1 + (1 - k)\,(-1 + \beta_1)\right)\Big)\Big) -$$
$$\Big((1 - k)\,r\,(1 - k - \mathcal{L})\,\big(r\,(-1 + S)\,z\,\alpha_2 + \alpha_1\big(-k - r\,(1 - k - \mathcal{L}) + r\,S\,(1 - k - \mathcal{L}) - \mathcal{L} +$$
$$(1 - \mathcal{L})\left(1 + \left(-1 + r - r\,S\right)(1 - k - \mathcal{L}) + \left(1 + r\,(-1 + S)\right)(-k - \mathcal{L})\,\alpha_2\right)\big)\big)\,(-1 + \beta_1)\Big)\Big/$$
$$\Big(\big(-1 + \left(1 + r\,(-1 + S)\right)(1 - k - \mathcal{L}) + (1 - \mathcal{L})\left(1 + \left(-1 + r - r\,S\right)(1 - k - \mathcal{L}) + \left(1 + r\,(-1 + S)\right)(-k - \mathcal{L})\,\alpha_2\right)\big)$$
$$\left(1 + (1 - k)\,(-1 + \beta_1)\right)\Big) -$$
$$\left(1 - k - r + k\,r - \mathcal{L} + r\,\mathcal{L}\right)\beta_2 - \lambda_1\big)\big)\big\}\ /.\ \lambda_0 \to \frac{1}{2}\big(2 - k - r + k$$

$r - 2\,\mathcal{L} + r\,\mathcal{L} - \alpha_2 + r\,\alpha_2 + \mathcal{L}$

$\alpha_2 - r$

```
ℒ
α2 -
√(-4 (-1 + r) (-1 + ℒ) (-1 + k + ℒ) (-1 + α2) +
    (k (-1 + r) + (-2 + r) (-1 + ℒ) + (-1 + r + ℒ - r ℒ) α2)^2));
```

```
Solve[% == 0, λ1];
```

```
Simplify[%];
```

Solving for second eigenvalue approximated to first order:

```
{(-((-k (1 - k - r + k r - ℒ + r ℒ) ((z (-1 + (1 + r (-1 + S)) (1 - k - ℒ)) α2) / (-1 + (1 + r (-1 + S)) (1 - k - ℒ) + (1 - ℒ)
                (1 + (-1 + r - r S) (1 - k - ℒ) + (1 + r (-1 + S)) (-k - ℒ) α2)) - ((1 + r (-1 + S)) z (1 - k - ℒ) α2) /
              (-1 + (1 + r (-1 + S)) (1 - k - ℒ) + (1 - ℒ) (1 + (-1 + r - r S) (1 - k - ℒ) + (1 + r (-1 + S)) (-k - ℒ) α2)))
           (-1 + β1) - (z - k z + (r z (-1 + (1 + r (-1 + S)) (1 - k - ℒ)) (1 - k - ℒ)) /
              (-1 + (1 + r (-1 + S)) (1 - k - ℒ) + (1 - ℒ) (1 + (-1 + r - r S) (1 - k - ℒ) + (1 + r (-1 + S)) (-k - ℒ) α2)) -
            (r (1 + r (-1 + S)) z (1 - k - ℒ)^2 α2) / (-1 + (1 + r (-1 + S)) (1 - k - ℒ) +
               (1 - ℒ) (1 + (-1 + r - r S) (1 - k - ℒ) + (1 + r (-1 + S)) (-k - ℒ) α2))) (-1 + β1))
       (-((r - k r) (r - r ℒ) α2) + (r - k r) (1 - ℒ - α2 + r α2 + ℒ α2 - r ℒ α2 - λ0))) - (r - r ℒ)
     (-r (-1 + k + ℒ) α1 + r (1 - k - ℒ) ((z (-1 + (1 + r (-1 + S)) (1 - k - ℒ)) α2) / (-1 + (1 + r (-1 + S)) (1 - k - ℒ) +
              (1 - ℒ) (1 + (-1 + r - r S) (1 - k - ℒ) + (1 + r (-1 + S)) (-k - ℒ) α2)) - ((1 + r (-1 + S)) z (1 - k - ℒ) α2) /
           (-1 + (1 + r (-1 + S)) (1 - k - ℒ) + (1 - ℒ) (1 + (-1 + r - r S) (1 - k - ℒ) + (1 + r (-1 + S)) (-k - ℒ) α2))) +
       (1 - k - r + k r - ℒ + r ℒ) (ℒ + k α2) ((z (-1 + (1 + r (-1 + S)) (1 - k - ℒ)) α2) / (-1 + (1 + r (-1 + S)) (1 - k - ℒ) +
              (1 - ℒ) (1 + (-1 + r - r S) (1 - k - ℒ) + (1 + r (-1 + S)) (-k - ℒ) α2)) - ((1 + r (-1 + S)) z (1 - k - ℒ) α2) /
           (-1 + (1 + r (-1 + S)) (1 - k - ℒ) + (1 - ℒ) (1 + (-1 + r - r S) (1 - k - ℒ) + (1 + r (-1 + S)) (-k - ℒ) α2))) -
       α2 (y - k y - r y + k r y - r z + k r z - y ℒ + r y ℒ - (r z (-1 + (1 + r (-1 + S)) (1 - k - ℒ)) (1 - k - ℒ)) /
           (-1 + (1 + r (-1 + S)) (1 - k - ℒ) + (1 - ℒ) (1 + (-1 + r - r S) (1 - k - ℒ) + (1 + r (-1 + S)) (-k - ℒ) α2)) +
         (r (1 + r (-1 + S)) z (1 - k - ℒ)^2 α2) / (-1 + (1 + r (-1 + S)) (1 - k - ℒ) +
            (1 - ℒ) (1 + (-1 + r - r S) (1 - k - ℒ) + (1 + r (-1 + S)) (-k - ℒ) α2))) -
       (-1 + k + r - k r + ℒ - r ℒ) α2 (k α1 + (z (-1 + (1 + r (-1 + S)) (1 - k - ℒ)) ℒ) /
           (-1 + (1 + r (-1 + S)) (1 - k - ℒ) + (1 - ℒ) (1 + (-1 + r - r S) (1 - k - ℒ) + (1 + r (-1 + S)) (-k - ℒ) α2)) +
         (k z (-1 + (1 + r (-1 + S)) (1 - k - ℒ)) α2) / (-1 + (1 + r (-1 + S)) (1 - k - ℒ) + (1 - ℒ)
              (1 + (-1 + r - r S) (1 - k - ℒ) + (1 + r (-1 + S)) (-k - ℒ) α2)) - (k (1 + r (-1 + S)) z (1 - k - ℒ) α2) /
           (-1 + (1 + r (-1 + S)) (1 - k - ℒ) + (1 - ℒ) (1 + (-1 + r - r S) (1 - k - ℒ) + (1 + r (-1 + S)) (-k - ℒ) α2)) -
         ((1 + r (-1 + S)) z (1 - k - ℒ) ℒ α2) / (-1 + (1 + r (-1 + S)) (1 - k - ℒ) +
            (1 - ℒ) (1 + (-1 + r - r S) (1 - k - ℒ) + (1 + r (-1 + S)) (-k - ℒ) α2)) -
         ((1 - k) k (r (-1 + S) z α2 + α1 (-k - r (1 - k - ℒ) + r S (1 - k - ℒ) - ℒ + (1 - ℒ) (1 + (-1 + r - r S)
                     (1 - k - ℒ) + (1 + r (-1 + S)) (-k - ℒ) α2))) (-1 + β1)) / ((-1 + (1 + r (-1 + S)) (1 - k - ℒ) +
              (1 - ℒ) (1 + (-1 + r - r S) (1 - k - ℒ) + (1 + r (-1 + S)) (-k - ℒ) α2)) (1 + (1 - k) (-1 + β1)))) -
       ((1 - k) r (1 - k - ℒ) (r (-1 + S) z α2 + α1 (-k - r (1 - k - ℒ) + r S (1 - k - ℒ) - ℒ +
                (1 - ℒ) (1 + (-1 + r - r S) (1 - k - ℒ) + (1 + r (-1 + S)) (-k - ℒ) α2))) (-1 + β1)) /
```

$$((-1+(1+r(-1+S))(1-k-\mathcal{L})+(1-\mathcal{L})(1+(-1+r-rS)(1-k-\mathcal{L})+(1+r(-1+S))(-k-\mathcal{L})\alpha_2))$$
$$(1+(1-k)(-1+\beta_1))))((-1+k)(-1+\beta_1)-\lambda_0)-$$
$$(-1+k+r-kr+\mathcal{L}-r\mathcal{L})\alpha_2((r-kr)((z-(rz(-1+(1+r(-1+S))(1-k-\mathcal{L}))(-1+\mathcal{L}))/(-1+$$
$$(1+r(-1+S))(1-k-\mathcal{L})+(1-\mathcal{L})(1+(-1+r-rS)(1-k-\mathcal{L})+(1+r(-1+S))(-k-\mathcal{L})\alpha_2))+$$
$$(r(1+r(-1+S))z(1-k-\mathcal{L})(-1+\mathcal{L})\alpha_2)/(-1+(1+r(-1+S))(1-k-\mathcal{L})+$$
$$(1-\mathcal{L})(1+(-1+r-rS)(1-k-\mathcal{L})+(1+r(-1+S))(-k-\mathcal{L})\alpha_2)))(-1+\beta_1)-$$
$$k(z+(z(-1+(1+r(-1+S))(1-k-\mathcal{L})))/(-1+(1+r(-1+S))(1-k-\mathcal{L})+(1-\mathcal{L})(1+(-1+r-rS)$$
$$(1-k-\mathcal{L})+(1+r(-1+S))(-k-\mathcal{L})\alpha_2))-(z(-1+(1+r(-1+S))(1-k-\mathcal{L}))\mathcal{L})/(-1+$$
$$(1+r(-1+S))(1-k-\mathcal{L})+(1-\mathcal{L})(1+(-1+r-rS)(1-k-\mathcal{L})+(1+r(-1+S))(-k-\mathcal{L})\alpha_2))-$$
$$(z(-1+(1+r(-1+S))(1-k-\mathcal{L}))\alpha_2)/(-1+(1+r(-1+S))(1-k-\mathcal{L})+$$
$$(1-\mathcal{L})(1+(-1+r-rS)(1-k-\mathcal{L})+(1+r(-1+S))(-k-\mathcal{L})\alpha_2))+$$
$$(rz(-1+(1+r(-1+S))(1-k-\mathcal{L}))\alpha_2)/(-1+(1+r(-1+S))(1-k-\mathcal{L})+$$
$$(1-\mathcal{L})(1+(-1+r-rS)(1-k-\mathcal{L})+(1+r(-1+S))(-k-\mathcal{L})\alpha_2))-$$
$$(r(1+r(-1+S))z(1-k-\mathcal{L})\alpha_2)/(-1+(1+r(-1+S))(1-k-\mathcal{L})+$$
$$(1-\mathcal{L})(1+(-1+r-rS)(1-k-\mathcal{L})+(1+r(-1+S))(-k-\mathcal{L})\alpha_2))+$$
$$(z(-1+(1+r(-1+S))(1-k-\mathcal{L}))\mathcal{L}\alpha_2)/(-1+(1+r(-1+S))(1-k-\mathcal{L})+$$
$$(1-\mathcal{L})(1+(-1+r-rS)(1-k-\mathcal{L})+(1+r(-1+S))(-k-\mathcal{L})\alpha_2))-$$
$$(rz(-1+(1+r(-1+S))(1-k-\mathcal{L}))\mathcal{L}\alpha_2)/(-1+(1+r(-1+S))(1-k-\mathcal{L})+$$
$$(1-\mathcal{L})(1+(-1+r-rS)(1-k-\mathcal{L})+(1+r(-1+S))(-k-\mathcal{L})\alpha_2))+$$
$$(r(1+r(-1+S))z(1-k-\mathcal{L})\mathcal{L}\alpha_2)/(-1+(1+r(-1+S))(1-k-\mathcal{L})+$$
$$(1-\mathcal{L})(1+(-1+r-rS)(1-k-\mathcal{L})+(1+r(-1+S))(-k-\mathcal{L})\alpha_2)))(-1+\beta_1)-$$
$$(-ry-z-rz+ry\mathcal{L}+r(-1+\mathcal{L})\alpha_1-(rz(-1+(1+r(-1+S))(1-k-\mathcal{L})))/$$
$$(-1+(1+r(-1+S))(1-k-\mathcal{L})+(1-\mathcal{L})(1+(-1+r-rS)(1-k-\mathcal{L})+(1+r(-1+S))(-k-\mathcal{L})\alpha_2))+$$
$$(rz(-1+(1+r(-1+S))(1-k-\mathcal{L}))\mathcal{L})/(-1+(1+r(-1+S))(1-k-\mathcal{L})+$$
$$(1-\mathcal{L})(1+(-1+r-rS)(1-k-\mathcal{L})+(1+r(-1+S))(-k-\mathcal{L})\alpha_2))-$$
$$(rz(-1+(1+r(-1+S))(1-k-\mathcal{L}))\alpha_2)/(-1+(1+r(-1+S))(1-k-\mathcal{L})+(1-\mathcal{L})$$
$$(1+(-1+r-rS)(1-k-\mathcal{L})+(1+r(-1+S))(-k-\mathcal{L})\alpha_2))+(2r(1+r(-1+S))z(1-k-\mathcal{L})\alpha_2)/$$
$$(-1+(1+r(-1+S))(1-k-\mathcal{L})+(1-\mathcal{L})(1+(-1+r-rS)(1-k-\mathcal{L})+(1+r(-1+S))(-k-\mathcal{L})\alpha_2))+$$
$$(rz(-1+(1+r(-1+S))(1-k-\mathcal{L}))\mathcal{L}\alpha_2)/(-1+(1+r(-1+S))(1-k-\mathcal{L})+$$
$$(1-\mathcal{L})(1+(-1+r-rS)(1-k-\mathcal{L})+(1+r(-1+S))(-k-\mathcal{L})\alpha_2))-$$
$$(2r(1+r(-1+S))z(1-k-\mathcal{L})\mathcal{L}\alpha_2)/(-1+(1+r(-1+S))(1-k-\mathcal{L})+$$
$$(1-\mathcal{L})(1+(-1+r-rS)(1-k-\mathcal{L})+(1+r(-1+S))(-k-\mathcal{L})\alpha_2))-$$
$$(-k-\mathcal{L})(z+(z(-1+(1+r(-1+S))(1-k-\mathcal{L})))/(-1+(1+r(-1+S))(1-k-\mathcal{L})+$$
$$(1-\mathcal{L})(1+(-1+r-rS)(1-k-\mathcal{L})+(1+r(-1+S))(-k-\mathcal{L})\alpha_2))-$$
$$(z(-1+(1+r(-1+S))(1-k-\mathcal{L}))\mathcal{L})/(-1+(1+r(-1+S))(1-k-\mathcal{L})+$$
$$(1-\mathcal{L})(1+(-1+r-rS)(1-k-\mathcal{L})+(1+r(-1+S))(-k-\mathcal{L})\alpha_2))-$$
$$(z(-1+(1+r(-1+S))(1-k-\mathcal{L}))\alpha_2)/(-1+(1+r(-1+S))(1-k-\mathcal{L})+$$
$$(1-\mathcal{L})(1+(-1+r-rS)(1-k-\mathcal{L})+(1+r(-1+S))(-k-\mathcal{L})\alpha_2))+$$

$$(r\,z\,(-1+(1+r\,(-1+S))\,(1-k-\mathcal{L}))\,\alpha_2)\,/\,(-1+(1+r\,(-1+S))\,(1-k-\mathcal{L})+$$
$$(1-\mathcal{L})\,(1+(-1+r-r\,S)\,(1-k-\mathcal{L})+(1+r\,(-1+S))\,(-k-\mathcal{L})\,\alpha_2))-$$
$$(r\,(1+r\,(-1+S))\,z\,(1-k-\mathcal{L})\,\alpha_2)\,/\,(-1+(1+r\,(-1+S))\,(1-k-\mathcal{L})+$$
$$(1-\mathcal{L})\,(1+(-1+r-r\,S)\,(1-k-\mathcal{L})+(1+r\,(-1+S))\,(-k-\mathcal{L})\,\alpha_2))+$$
$$(z\,(-1+(1+r\,(-1+S))\,(1-k-\mathcal{L}))\,\mathcal{L}\,\alpha_2)\,/\,(-1+(1+r\,(-1+S))\,(1-k-\mathcal{L})+$$
$$(1-\mathcal{L})\,(1+(-1+r-r\,S)\,(1-k-\mathcal{L})+(1+r\,(-1+S))\,(-k-\mathcal{L})\,\alpha_2))-$$
$$(r\,z\,(-1+(1+r\,(-1+S))\,(1-k-\mathcal{L}))\,\mathcal{L}\,\alpha_2)\,/\,(-1+(1+r\,(-1+S))\,(1-k-\mathcal{L})+$$
$$(1-\mathcal{L})\,(1+(-1+r-r\,S)\,(1-k-\mathcal{L})+(1+r\,(-1+S))\,(-k-\mathcal{L})\,\alpha_2))+$$
$$(r\,(1+r\,(-1+S))\,z\,(1-k-\mathcal{L})\,\mathcal{L}\,\alpha_2)\,/\,(-1+(1+r\,(-1+S))\,(1-k-\mathcal{L})+$$
$$(1-\mathcal{L})\,(1+(-1+r-r\,S)\,(1-k-\mathcal{L})+(1+r\,(-1+S))\,(-k-\mathcal{L})\,\alpha_2)))-$$
$$(r-r\,\mathcal{L})\,(-k\,\alpha_1-(z\,(-1+(1+r\,(-1+S))\,(1-k-\mathcal{L}))\,\mathcal{L})\,/\,(-1+(1+r\,(-1+S))\,(1-k-\mathcal{L})+$$
$$(1-\mathcal{L})\,(1+(-1+r-r\,S)\,(1-k-\mathcal{L})+(1+r\,(-1+S))\,(-k-\mathcal{L})\,\alpha_2))-$$
$$(k\,z\,(-1+(1+r\,(-1+S))\,(1-k-\mathcal{L}))\,\alpha_2)\,/\,(-1+(1+r\,(-1+S))\,(1-k-\mathcal{L})+$$
$$(1-\mathcal{L})\,(1+(-1+r-r\,S)\,(1-k-\mathcal{L})+(1+r\,(-1+S))\,(-k-\mathcal{L})\,\alpha_2))+$$
$$(k\,(1+r\,(-1+S))\,z\,(1-k-\mathcal{L})\,\alpha_2)\,/\,(-1+(1+r\,(-1+S))\,(1-k-\mathcal{L})+$$
$$(1-\mathcal{L})\,(1+(-1+r-r\,S)\,(1-k-\mathcal{L})+(1+r\,(-1+S))\,(-k-\mathcal{L})\,\alpha_2))+$$
$$((1+r\,(-1+S))\,z\,(1-k-\mathcal{L})\,\mathcal{L}\,\alpha_2)\,/\,(-1+(1+r\,(-1+S))\,(1-k-\mathcal{L})+(1-\mathcal{L})$$
$$(1+(-1+r-r\,S)\,(1-k-\mathcal{L})+(1+r\,(-1+S))\,(-k-\mathcal{L})\,\alpha_2))+((1-k)\,k\,(r\,(-1+S)\,z\,\alpha_2+$$
$$\alpha_1\,(-k-r\,(1-k-\mathcal{L})+r\,S\,(1-k-\mathcal{L})-\mathcal{L}+(1-\mathcal{L})\,(1+(-1+r-r\,S)\,(1-k-\mathcal{L})+$$
$$(1+r\,(-1+S))\,(-k-\mathcal{L})\,\alpha_2)))\,(-1+\beta_1))\,/\,((-1+(1+r\,(-1+S))\,(1-k-\mathcal{L})+(1-\mathcal{L})$$
$$(1+(-1+r-r\,S)\,(1-k-\mathcal{L})+(1+r\,(-1+S))\,(-k-\mathcal{L})\,\alpha_2))\,(1+(1-k)\,(-1+\beta_1))))-$$
$$((1-k)\,r\,(-1+\mathcal{L})\,(r\,(-1+S)\,z\,\alpha_2+\alpha_1\,(-k-r\,(1-k-\mathcal{L})+r\,S\,(1-k-\mathcal{L})-\mathcal{L}+(1-\mathcal{L})$$
$$(1+(-1+r-r\,S)\,(1-k-\mathcal{L})+(1+r\,(-1+S))\,(-k-\mathcal{L})\,\alpha_2)))\,(-1+\beta_1))\,/$$
$$((-1+(1+r\,(-1+S))\,(1-k-\mathcal{L})+(1-\mathcal{L})\,(1+(-1+r-r\,S)\,(1-k-\mathcal{L})+(1+r\,(-1+S))\,(-k-\mathcal{L})\,\alpha_2))$$
$$(1+(1-k)\,(-1+\beta_1)))+\beta_2-r\,\beta_2-\mathcal{L}\,\beta_2+r\,\mathcal{L}\,\beta_2)\,((-1+k)\,(-1+\beta_1)-\lambda_0)-$$
$$(r-r\,\mathcal{L})\,((-1+k)\,\alpha_1+(z-k\,z+((1-k)\,r\,z\,(-1+(1+r\,(-1+S))\,(1-k-\mathcal{L})))\,/\,(-1+$$
$$(1+r\,(-1+S))\,(1-k-\mathcal{L})+(1-\mathcal{L})\,(1+(-1+r-r\,S)\,(1-k-\mathcal{L})+(1+r\,(-1+S))\,(-k-\mathcal{L})\,\alpha_2))-$$
$$((1-k)\,r\,(1+r\,(-1+S))\,z\,(1-k-\mathcal{L})\,\alpha_2)\,/\,(-1+(1+r\,(-1+S))\,(1-k-\mathcal{L})+$$
$$(1-\mathcal{L})\,(1+(-1+r-r\,S)\,(1-k-\mathcal{L})+(1+r\,(-1+S))\,(-k-\mathcal{L})\,\alpha_2)))\,(-1+\beta_1)-$$
$$k\,(-((-1+k)\,\alpha_1)-(-r+k\,r)\,((z\,(-1+(1+r\,(-1+S))\,(1-k-\mathcal{L}))\,\alpha_2)\,/\,(-1+(1+r\,(-1+S))$$
$$(1-k-\mathcal{L})+(1-\mathcal{L})\,(1+(-1+r-r\,S)\,(1-k-\mathcal{L})+(1+r\,(-1+S))\,(-k-\mathcal{L})\,\alpha_2))-$$
$$((1+r\,(-1+S))\,z\,(1-k-\mathcal{L})\,\alpha_2)\,/\,(-1+(1+r\,(-1+S))\,(1-k-\mathcal{L})+$$
$$(1-\mathcal{L})\,(1+(-1+r-r\,S)\,(1-k-\mathcal{L})+(1+r\,(-1+S))\,(-k-\mathcal{L})\,\alpha_2)))+$$
$$((1-k)\,(-1+k)\,(r\,(-1+S)\,z\,\alpha_2+\alpha_1\,(-k-r\,(1-k-\mathcal{L})+r\,S\,(1-k-\mathcal{L})-\mathcal{L}+$$
$$(1-\mathcal{L})\,(1+(-1+r-r\,S)\,(1-k-\mathcal{L})+(1+r\,(-1+S))\,(-k-\mathcal{L})\,\alpha_2)))$$
$$(-1+\beta_1))\,/\,((-1+(1+r\,(-1+S))\,(1-k-\mathcal{L})+(1-\mathcal{L})\,(1+(-1+r-r\,S)\,(1-k-\mathcal{L})+$$
$$(1+r\,(-1+S))\,(-k-\mathcal{L})\,\alpha_2))\,(1+(1-k)\,(-1+\beta_1))))\,(-1+\beta_1)-$$
$$(-1+k)\,(-k\,\alpha_1-(z\,(-1+(1+r\,(-1+S))\,(1-k-\mathcal{L}))\,\mathcal{L})\,/\,(-1+(1+r\,(-1+S))\,(1-k-\mathcal{L})+$$

$$(1 - \mathcal{L}) (1 + (-1 + r - r S) (1 - k - \mathcal{L}) + (1 + r (-1 + S)) (-k - \mathcal{L}) \alpha_2)) -$$
$$(k z (-1 + (1 + r (-1 + S)) (1 - k - \mathcal{L})) \alpha_2) / (-1 + (1 + r (-1 + S)) (1 - k - \mathcal{L}) +$$
$$(1 - \mathcal{L}) (1 + (-1 + r - r S) (1 - k - \mathcal{L}) + (1 + r (-1 + S)) (-k - \mathcal{L}) \alpha_2)) +$$
$$(k (1 + r (-1 + S)) z (1 - k - \mathcal{L}) \alpha_2) / (-1 + (1 + r (-1 + S)) (1 - k - \mathcal{L}) +$$
$$(1 - \mathcal{L}) (1 + (-1 + r - r S) (1 - k - \mathcal{L}) + (1 + r (-1 + S)) (-k - \mathcal{L}) \alpha_2)) +$$
$$((1 + r (-1 + S)) z (1 - k - \mathcal{L}) \mathcal{L} \alpha_2) / (-1 + (1 + r (-1 + S)) (1 - k - \mathcal{L}) +$$
$$(1 - \mathcal{L}) (1 + (-1 + r - r S) (1 - k - \mathcal{L}) + (1 + r (-1 + S)) (-k - \mathcal{L}) \alpha_2)) +$$
$$((1 - k) k (r (-1 + S) z \alpha_2 + \alpha_1 (-k - r (1 - k - \mathcal{L}) + r S (1 - k - \mathcal{L}) - \mathcal{L} + (1 - \mathcal{L})$$
$$(1 + (-1 + r - r S) (1 - k - \mathcal{L}) + (1 + r (-1 + S)) (-k - \mathcal{L}) \alpha_2))) (-1 + \beta_1)) /$$
$$((-1 + (1 + r (-1 + S)) (1 - k - \mathcal{L}) + (1 - \mathcal{L}) (1 + (-1 + r - r S) (1 - k - \mathcal{L}) + (1 + r (-1 + S))$$
$$(-k - \mathcal{L}) \alpha_2)) (1 + (1 - k) (-1 + \beta_1)))) (-1 + \beta_1) - \lambda_1)) + (1 - k - r + k r - \mathcal{L} + r \mathcal{L} - \lambda_0)$$
$$(-((r - k r) \alpha_2 ((z - (r z (-1 + (1 + r (-1 + S)) (1 - k - \mathcal{L})) (-1 + \mathcal{L})) / (-1 + (1 + r (-1 + S)) (1 - k - \mathcal{L}) +$$
$$(1 - \mathcal{L}) (1 + (-1 + r - r S) (1 - k - \mathcal{L}) + (1 + r (-1 + S)) (-k - \mathcal{L}) \alpha_2)) +$$
$$(r (1 + r (-1 + S)) z (1 - k - \mathcal{L}) (-1 + \mathcal{L}) \alpha_2) / (-1 + (1 + r (-1 + S)) (1 - k - \mathcal{L}) +$$
$$(1 - \mathcal{L}) (1 + (-1 + r - r S) (1 - k - \mathcal{L}) + (1 + r (-1 + S)) (-k - \mathcal{L}) \alpha_2))) (-1 + \beta_1) -$$
$$k (z + (z (-1 + (1 + r (-1 + S)) (1 - k - \mathcal{L}))) / (-1 + (1 + r (-1 + S)) (1 - k - \mathcal{L}) +$$
$$(1 - \mathcal{L}) (1 + (-1 + r - r S) (1 - k - \mathcal{L}) + (1 + r (-1 + S)) (-k - \mathcal{L}) \alpha_2)) -$$
$$(z (-1 + (1 + r (-1 + S)) (1 - k - \mathcal{L})) \mathcal{L}) / (-1 + (1 + r (-1 + S)) (1 - k - \mathcal{L}) +$$
$$(1 - \mathcal{L}) (1 + (-1 + r - r S) (1 - k - \mathcal{L}) + (1 + r (-1 + S)) (-k - \mathcal{L}) \alpha_2)) -$$
$$(z (-1 + (1 + r (-1 + S)) (1 - k - \mathcal{L})) \alpha_2) / (-1 + (1 + r (-1 + S)) (1 - k - \mathcal{L}) +$$
$$(1 - \mathcal{L}) (1 + (-1 + r - r S) (1 - k - \mathcal{L}) + (1 + r (-1 + S)) (-k - \mathcal{L}) \alpha_2)) +$$
$$(r z (-1 + (1 + r (-1 + S)) (1 - k - \mathcal{L})) \alpha_2) / (-1 + (1 + r (-1 + S)) (1 - k - \mathcal{L}) +$$
$$(1 - \mathcal{L}) (1 + (-1 + r - r S) (1 - k - \mathcal{L}) + (1 + r (-1 + S)) (-k - \mathcal{L}) \alpha_2)) -$$
$$(r (1 + r (-1 + S)) z (1 - k - \mathcal{L}) \alpha_2) / (-1 + (1 + r (-1 + S)) (1 - k - \mathcal{L}) +$$
$$(1 - \mathcal{L}) (1 + (-1 + r - r S) (1 - k - \mathcal{L}) + (1 + r (-1 + S)) (-k - \mathcal{L}) \alpha_2)) +$$
$$(z (-1 + (1 + r (-1 + S)) (1 - k - \mathcal{L})) \mathcal{L} \alpha_2) / (-1 + (1 + r (-1 + S)) (1 - k - \mathcal{L}) +$$
$$(1 - \mathcal{L}) (1 + (-1 + r - r S) (1 - k - \mathcal{L}) + (1 + r (-1 + S)) (-k - \mathcal{L}) \alpha_2)) -$$
$$(r z (-1 + (1 + r (-1 + S)) (1 - k - \mathcal{L})) \mathcal{L} \alpha_2) / (-1 + (1 + r (-1 + S)) (1 - k - \mathcal{L}) +$$
$$(1 - \mathcal{L}) (1 + (-1 + r - r S) (1 - k - \mathcal{L}) + (1 + r (-1 + S)) (-k - \mathcal{L}) \alpha_2)) +$$
$$(r (1 + r (-1 + S)) z (1 - k - \mathcal{L}) \mathcal{L} \alpha_2) / (-1 + (1 + r (-1 + S)) (1 - k - \mathcal{L}) +$$
$$(1 - \mathcal{L}) (1 + (-1 + r - r S) (1 - k - \mathcal{L}) + (1 + r (-1 + S)) (-k - \mathcal{L}) \alpha_2))) (-1 + \beta_1)) +$$
$$((-1 + k) (-1 + \beta_1) - \lambda_0) (-y - z + y \mathcal{L} + r (-1 + \mathcal{L}) \alpha_1 + y \alpha_2 - r y \alpha_2 - r z \alpha_2 - y \mathcal{L} \alpha_2 +$$
$$r y \mathcal{L} \alpha_2 - (2 r z (-1 + (1 + r (-1 + S)) (1 - k - \mathcal{L})) \alpha_2) /$$
$$(-1 + (1 + r (-1 + S)) (1 - k - \mathcal{L}) + (1 - \mathcal{L}) (1 + (-1 + r - r S) (1 - k - \mathcal{L}) + (1 + r (-1 + S)) (-k - \mathcal{L}) \alpha_2)) +$$
$$(r (1 + r (-1 + S)) z (1 - k - \mathcal{L}) \alpha_2) / (-1 + (1 + r (-1 + S)) (1 - k - \mathcal{L}) +$$
$$(1 - \mathcal{L}) (1 + (-1 + r - r S) (1 - k - \mathcal{L}) + (1 + r (-1 + S)) (-k - \mathcal{L}) \alpha_2)) +$$
$$(2 r z (-1 + (1 + r (-1 + S)) (1 - k - \mathcal{L})) \mathcal{L} \alpha_2) / (-1 + (1 + r (-1 + S)) (1 - k - \mathcal{L}) +$$
$$(1 - \mathcal{L}) (1 + (-1 + r - r S) (1 - k - \mathcal{L}) + (1 + r (-1 + S)) (-k - \mathcal{L}) \alpha_2)) -$$

$$(r (1 + r (-1 + S)) z (1 - k - \mathcal{L}) \mathcal{L} \alpha_2) / (-1 + (1 + r (-1 + S)) (1 - k - \mathcal{L}) + (1 - \mathcal{L})$$

$$(1 + (-1 + r - r S) (1 - k - \mathcal{L}) + (1 + r (-1 + S)) (-k - \mathcal{L}) \alpha_2)) + (r (1 + r (-1 + S)) z (1 - k - \mathcal{L}) \alpha_2^2) /$$

$$(-1 + (1 + r (-1 + S)) (1 - k - \mathcal{L}) + (1 - \mathcal{L}) (1 + (-1 + r - r S) (1 - k - \mathcal{L}) + (1 + r (-1 + S)) (-k - \mathcal{L}) \alpha_2)) -$$

$$(r (1 + r (-1 + S)) z (1 - k - \mathcal{L}) \mathcal{L} \alpha_2^2) / (-1 + (1 + r (-1 + S)) (1 - k - \mathcal{L}) +$$

$$(1 - \mathcal{L}) (1 + (-1 + r - r S) (1 - k - \mathcal{L}) + (1 + r (-1 + S)) (-k - \mathcal{L}) \alpha_2)) -$$

$$(-\mathcal{L} - k \alpha_2) (z + (z (-1 + (1 + r (-1 + S)) (1 - k - \mathcal{L}))) / (-1 + (1 + r (-1 + S)) (1 - k - \mathcal{L}) +$$

$$(1 - \mathcal{L}) (1 + (-1 + r - r S) (1 - k - \mathcal{L}) + (1 + r (-1 + S)) (-k - \mathcal{L}) \alpha_2)) -$$

$$(z (-1 + (1 + r (-1 + S)) (1 - k - \mathcal{L})) \mathcal{L}) / (-1 + (1 + r (-1 + S)) (1 - k - \mathcal{L}) +$$

$$(1 - \mathcal{L}) (1 + (-1 + r - r S) (1 - k - \mathcal{L}) + (1 + r (-1 + S)) (-k - \mathcal{L}) \alpha_2)) -$$

$$(z (-1 + (1 + r (-1 + S)) (1 - k - \mathcal{L})) \alpha_2) / (-1 + (1 + r (-1 + S)) (1 - k - \mathcal{L}) +$$

$$(1 - \mathcal{L}) (1 + (-1 + r - r S) (1 - k - \mathcal{L}) + (1 + r (-1 + S)) (-k - \mathcal{L}) \alpha_2)) +$$

$$(r z (-1 + (1 + r (-1 + S)) (1 - k - \mathcal{L})) \alpha_2) / (-1 + (1 + r (-1 + S)) (1 - k - \mathcal{L}) +$$

$$(1 - \mathcal{L}) (1 + (-1 + r - r S) (1 - k - \mathcal{L}) + (1 + r (-1 + S)) (-k - \mathcal{L}) \alpha_2)) -$$

$$(r (1 + r (-1 + S)) z (1 - k - \mathcal{L}) \alpha_2) / (-1 + (1 + r (-1 + S)) (1 - k - \mathcal{L}) +$$

$$(1 - \mathcal{L}) (1 + (-1 + r - r S) (1 - k - \mathcal{L}) + (1 + r (-1 + S)) (-k - \mathcal{L}) \alpha_2)) +$$

$$(z (-1 + (1 + r (-1 + S)) (1 - k - \mathcal{L})) \mathcal{L} \alpha_2) / (-1 + (1 + r (-1 + S)) (1 - k - \mathcal{L}) +$$

$$(1 - \mathcal{L}) (1 + (-1 + r - r S) (1 - k - \mathcal{L}) + (1 + r (-1 + S)) (-k - \mathcal{L}) \alpha_2)) -$$

$$(r z (-1 + (1 + r (-1 + S)) (1 - k - \mathcal{L})) \mathcal{L} \alpha_2) / (-1 + (1 + r (-1 + S)) (1 - k - \mathcal{L}) +$$

$$(1 - \mathcal{L}) (1 + (-1 + r - r S) (1 - k - \mathcal{L}) + (1 + r (-1 + S)) (-k - \mathcal{L}) \alpha_2)) +$$

$$(r (1 + r (-1 + S)) z (1 - k - \mathcal{L}) \mathcal{L} \alpha_2) / (-1 + (1 + r (-1 + S)) (1 - k - \mathcal{L}) +$$

$$(1 - \mathcal{L}) (1 + (-1 + r - r S) (1 - k - \mathcal{L}) + (1 + r (-1 + S)) (-k - \mathcal{L}) \alpha_2))) -$$

$$(1 - \mathcal{L} - \alpha_2 + r \alpha_2 + \mathcal{L} \alpha_2 - r \mathcal{L} \alpha_2) (-k \alpha_1 - (z (-1 + (1 + r (-1 + S)) (1 - k - \mathcal{L})) \mathcal{L}) / (-1 +$$

$$(1 + r (-1 + S)) (1 - k - \mathcal{L}) + (1 - \mathcal{L}) (1 + (-1 + r - r S) (1 - k - \mathcal{L}) + (1 + r (-1 + S)) (-k - \mathcal{L}) \alpha_2)) -$$

$$(k z (-1 + (1 + r (-1 + S)) (1 - k - \mathcal{L})) \alpha_2) / (-1 + (1 + r (-1 + S)) (1 - k - \mathcal{L}) +$$

$$(1 - \mathcal{L}) (1 + (-1 + r - r S) (1 - k - \mathcal{L}) + (1 + r (-1 + S)) (-k - \mathcal{L}) \alpha_2)) +$$

$$(k (1 + r (-1 + S)) z (1 - k - \mathcal{L}) \alpha_2) / (-1 + (1 + r (-1 + S)) (1 - k - \mathcal{L}) +$$

$$(1 - \mathcal{L}) (1 + (-1 + r - r S) (1 - k - \mathcal{L}) + (1 + r (-1 + S)) (-k - \mathcal{L}) \alpha_2)) +$$

$$((1 + r (-1 + S)) z (1 - k - \mathcal{L}) \mathcal{L} \alpha_2) / (-1 + (1 + r (-1 + S)) (1 - k - \mathcal{L}) +$$

$$(1 - \mathcal{L}) (1 + (-1 + r - r S) (1 - k - \mathcal{L}) + (1 + r (-1 + S)) (-k - \mathcal{L}) \alpha_2)) +$$

$$((1 - k) k (r (-1 + S) z \alpha_2 + \alpha_1 (-k - r (1 - k - \mathcal{L}) + r S (1 - k - \mathcal{L}) - \mathcal{L} + (1 - \mathcal{L})$$

$$(1 + (-1 + r - r S) (1 - k - \mathcal{L}) + (1 + r (-1 + S)) (-k - \mathcal{L}) \alpha_2)))$$

$$(-1 + \beta_1)) / ((-1 + (1 + r (-1 + S)) (1 - k - \mathcal{L}) + (1 - \mathcal{L}) (1 + (-1 + r - r S) (1 - k - \mathcal{L}) +$$

$$(1 + r (-1 + S)) (-k - \mathcal{L}) \alpha_2)) (1 + (1 - k) (-1 + \beta_1)))) -$$

$$((1 - k) r (-1 + \mathcal{L}) (r (-1 + S) z \alpha_2 + \alpha_1 (-k - r (1 - k - \mathcal{L}) + r S (1 - k - \mathcal{L}) - \mathcal{L} + (1 - \mathcal{L})$$

$$(1 + (-1 + r - r S) (1 - k - \mathcal{L}) + (1 + r (-1 + S)) (-k - \mathcal{L}) \alpha_2))) (-1 + \beta_1)) /$$

$$((-1 + (1 + r (-1 + S)) (1 - k - \mathcal{L}) + (1 - \mathcal{L}) (1 + (-1 + r - r S) (1 - k - \mathcal{L}) + (1 + r (-1 + S)) (-k - \mathcal{L}) \alpha_2))$$

$$(1 + (1 - k) (-1 + \beta_1))) - \lambda_1) + (1 - \mathcal{L} - \alpha_2 + r \alpha_2 + \mathcal{L} \alpha_2 - r \mathcal{L} \alpha_2 - \lambda_0)$$

$$((-1 + k) \alpha_1 + (z - k z + ((1 - k) r z (-1 + (1 + r (-1 + S)) (1 - k - \mathcal{L}))) / (-1 + (1 + r (-1 + S))$$

$$(1-k-\mathcal{L})+(1-\mathcal{L})\left(1+\left(-1+r-r\,S\right)(1-k-\mathcal{L})+\left(1+r\,(-1+S)\right)(-k-\mathcal{L})\,\alpha_2\right)\Big)-$$
$$\Big(\big((1-k)\,r\left(1+r\,(-1+S)\right)z\,(1-k-\mathcal{L})\,\alpha_2\big)\Big/\Big(-1+\left(1+r\,(-1+S)\right)(1-k-\mathcal{L})+$$
$$(1-\mathcal{L})\left(1+\left(-1+r-r\,S\right)(1-k-\mathcal{L})+\left(1+r\,(-1+S)\right)(-k-\mathcal{L})\,\alpha_2\right)\Big)\Big)\,(-1+\beta_1)-$$
$$k\,\Big(-\big((-1+k)\,\alpha_1\big)-\left(-r+k\,r\right)\Big(\Big(z\left(-1+\left(1+r\,(-1+S)\right)(1-k-\mathcal{L})\right)\alpha_2\Big)\Big/\Big(-1+\left(1+r\,(-1+S)\right)$$
$$(1-k-\mathcal{L})+(1-\mathcal{L})\left(1+\left(-1+r-r\,S\right)(1-k-\mathcal{L})+\left(1+r\,(-1+S)\right)(-k-\mathcal{L})\,\alpha_2\right)\Big)-$$
$$\Big(\left(1+r\,(-1+S)\right)z\,(1-k-\mathcal{L})\,\alpha_2\Big)\Big/\Big(-1+\left(1+r\,(-1+S)\right)(1-k-\mathcal{L})+$$
$$(1-\mathcal{L})\left(1+\left(-1+r-r\,S\right)(1-k-\mathcal{L})+\left(1+r\,(-1+S)\right)(-k-\mathcal{L})\,\alpha_2\right)\Big)\Big)+$$
$$\Big((1-k)\,(-1+k)\Big(r\,(-1+S)\,z\,\alpha_2+\alpha_1\Big(-k-r\,(1-k-\mathcal{L})+r\,S\,(1-k-\mathcal{L})-\mathcal{L}+$$
$$(1-\mathcal{L})\left(1+\left(-1+r-r\,S\right)(1-k-\mathcal{L})+\left(1+r\,(-1+S)\right)(-k-\mathcal{L})\,\alpha_2\right)\Big)\Big)$$
$$(-1+\beta_1)\Big)\Big/\Big(\Big(-1+\left(1+r\,(-1+S)\right)(1-k-\mathcal{L})+(1-\mathcal{L})\Big(1+\left(-1+r-r\,S\right)(1-k-\mathcal{L})+$$
$$\left(1+r\,(-1+S)\right)(-k-\mathcal{L})\,\alpha_2\Big)\Big)\left(1+(1-k)\,(-1+\beta_1)\right)\Big)\Big)\,(-1+\beta_1)-$$
$$(-1+k)\,\Big(-k\,\alpha_1-\Big(z\left(-1+\left(1+r\,(-1+S)\right)(1-k-\mathcal{L})\right)\mathcal{L}\Big)\Big/\Big(-1+\left(1+r\,(-1+S)\right)(1-k-\mathcal{L})+$$
$$(1-\mathcal{L})\left(1+\left(-1+r-r\,S\right)(1-k-\mathcal{L})+\left(1+r\,(-1+S)\right)(-k-\mathcal{L})\,\alpha_2\right)\Big)-$$
$$\Big(k\,z\left(-1+\left(1+r\,(-1+S)\right)(1-k-\mathcal{L})\right)\alpha_2\Big)\Big/\Big(-1+\left(1+r\,(-1+S)\right)(1-k-\mathcal{L})+$$
$$(1-\mathcal{L})\left(1+\left(-1+r-r\,S\right)(1-k-\mathcal{L})+\left(1+r\,(-1+S)\right)(-k-\mathcal{L})\,\alpha_2\right)\Big)+$$
$$\Big(k\left(1+r\,(-1+S)\right)z\,(1-k-\mathcal{L})\,\alpha_2\Big)\Big/\Big(-1+\left(1+r\,(-1+S)\right)(1-k-\mathcal{L})+$$
$$(1-\mathcal{L})\left(1+\left(-1+r-r\,S\right)(1-k-\mathcal{L})+\left(1+r\,(-1+S)\right)(-k-\mathcal{L})\,\alpha_2\right)\Big)+$$
$$\Big(\left(1+r\,(-1+S)\right)z\,(1-k-\mathcal{L})\,\mathcal{L}\,\alpha_2\Big)\Big/\Big(-1+\left(1+r\,(-1+S)\right)(1-k-\mathcal{L})+$$
$$(1-\mathcal{L})\left(1+\left(-1+r-r\,S\right)(1-k-\mathcal{L})+\left(1+r\,(-1+S)\right)(-k-\mathcal{L})\,\alpha_2\right)\Big)+$$
$$\Big((1-k)\,k\,\Big(r\,(-1+S)\,z\,\alpha_2+\alpha_1\Big(-k-r\,(1-k-\mathcal{L})+r\,S\,(1-k-\mathcal{L})-\mathcal{L}+(1-\mathcal{L})$$
$$\left(1+\left(-1+r-r\,S\right)(1-k-\mathcal{L})+\left(1+r\,(-1+S)\right)(-k-\mathcal{L})\,\alpha_2\right)\Big)\Big)$$
$$(-1+\beta_1)\Big)\Big/\Big(\Big(-1+\left(1+r\,(-1+S)\right)(1-k-\mathcal{L})+(1-\mathcal{L})\Big(1+\left(-1+r-r\,S\right)(1-k-\mathcal{L})+$$
$$\left(1+r\,(-1+S)\right)(-k-\mathcal{L})\,\alpha_2\Big)\Big)\left(1+(1-k)\,(-1+\beta_1)\right)\Big)\Big)\,(-1+\beta_1)-\lambda_1\Big)\Big)+$$
$$\left(1-\mathcal{L}-\alpha_2+r\,\alpha_2+\mathcal{L}\,\alpha_2-r\,\mathcal{L}\,\alpha_2-\lambda_0\right)\left((-1+k)\,(-1+\beta_1)-\lambda_0\right)\Big(-y+k\,y+r\,y-$$
$$k\,r\,y+$$
$$r\,z-$$
$$k\,r\,z+$$
$$y\,\mathcal{L}-$$
$$r\,y\,\mathcal{L}-$$
$$r\,(-1+k+\mathcal{L})\,\alpha_1+$$
$$\Big(r\,z\left(-1+\left(1+r\,(-1+S)\right)(1-k-\mathcal{L})\right)(1-k-\mathcal{L})\Big)\Big/$$
$$\Big(-1+\left(1+r\,(-1+S)\right)(1-k-\mathcal{L})+$$
$$(1-\mathcal{L})\left(1+\left(-1+r-r\,S\right)(1-k-\mathcal{L})+\left(1+r\,(-1+S)\right)(-k-\mathcal{L})\,\alpha_2\right)\Big)-$$
$$\Big(r\left(1+r\,(-1+S)\right)z\,(1-k-\mathcal{L})^2\,\alpha_2\Big)\Big/\Big(-1+\left(1+r\,(-1+S)\right)(1-k-\mathcal{L})+$$
$$(1-\mathcal{L})\left(1+\left(-1+r-r\,S\right)(1-k-\mathcal{L})+\left(1+r\,(-1+S)\right)(-k-\mathcal{L})\,\alpha_2\right)\Big)+$$
$$r\,(1-k-\mathcal{L})\,\Big(\Big(z\left(-1+\left(1+r\,(-1+S)\right)(1-k-\mathcal{L})\right)\alpha_2\Big)\Big/\Big(-1+\left(1+r\,(-1+S)\right)(1-k-\mathcal{L})+$$
$$(1-\mathcal{L})\left(1+\left(-1+r-r\,S\right)(1-k-\mathcal{L})+\left(1+r\,(-1+S)\right)(-k-\mathcal{L})\,\alpha_2\right)\Big)-\Big(\left(1+r\,(-1+S)\right)z\,(1-k-\mathcal{L})\,\alpha_2\Big)\Big/$$
$$\Big(-1+\left(1+r\,(-1+S)\right)(1-k-\mathcal{L})+(1-\mathcal{L})\left(1+\left(-1+r-r\,S\right)(1-k-\mathcal{L})+\left(1+r\,(-1+S)\right)(-k-\mathcal{L})\,\alpha_2\right)\Big)\Big)+$$
$$(k+\mathcal{L})\left(1-k-r+k\,r-\mathcal{L}+r\,\mathcal{L}\right)\Big(\Big(z\left(-1+\left(1+r\,(-1+S)\right)(1-k-\mathcal{L})\right)\alpha_2\Big)\Big/$$

```
(-1 + (1 + r (-1 + S)) (1 - k - ℒ) + (1 - ℒ) (1 + (-1 + r - r S) (1 - k - ℒ) + (1 + r (-1 + S)) (-k - ℒ) α2)) -
((1 + r (-1 + S)) z (1 - k - ℒ) α2) / (-1 + (1 + r (-1 + S)) (1 - k - ℒ) +
    (1 - ℒ) (1 + (-1 + r - r S) (1 - k - ℒ) + (1 + r (-1 + S)) (-k - ℒ) α2))) -
(-1 + k + r - k r + ℒ - r ℒ) (k α1 + (z (-1 + (1 + r (-1 + S)) (1 - k - ℒ)) ℒ) /
    (-1 + (1 + r (-1 + S)) (1 - k - ℒ) + (1 - ℒ) (1 + (-1 + r - r S) (1 - k - ℒ) + (1 + r (-1 + S)) (-k - ℒ) α2)) +
  (k z (-1 + (1 + r (-1 + S)) (1 - k - ℒ)) α2) / (-1 + (1 + r (-1 + S)) (1 - k - ℒ) +
    (1 - ℒ) (1 + (-1 + r - r S) (1 - k - ℒ) + (1 + r (-1 + S)) (-k - ℒ) α2)) -
  (k (1 + r (-1 + S)) z (1 - k - ℒ) α2) / (-1 + (1 + r (-1 + S)) (1 - k - ℒ) +
    (1 - ℒ) (1 + (-1 + r - r S) (1 - k - ℒ) + (1 + r (-1 + S)) (-k - ℒ) α2)) -
  ((1 + r (-1 + S)) z (1 - k - ℒ) ℒ α2) / (-1 + (1 + r (-1 + S)) (1 - k - ℒ) +
    (1 - ℒ) (1 + (-1 + r - r S) (1 - k - ℒ) + (1 + r (-1 + S)) (-k - ℒ) α2)) -
  ((1 - k) k (r (-1 + S) z α2 + α1 (-k - r (1 - k - ℒ) + r S (1 - k - ℒ) - ℒ + (1 - ℒ)
          (1 + (-1 + r - r S) (1 - k - ℒ) + (1 + r (-1 + S)) (-k - ℒ) α2))) (-1 + β1)) /
   ((-1 + (1 + r (-1 + S)) (1 - k - ℒ) + (1 - ℒ) (1 + (-1 + r - r S) (1 - k - ℒ) + (1 + r (-1 + S)) (-k - ℒ) α2))
    (1 + (1 - k) (-1 + β1)))) -
(((1 - k) r (1 - k - ℒ) (r (-1 + S) z α2 + α1 (-k - r (1 - k - ℒ) + r S (1 - k - ℒ) - ℒ +
        (1 - ℒ) (1 + (-1 + r - r S) (1 - k - ℒ) + (1 + r (-1 + S)) (-k - ℒ) α2))) (-1 + β1)) /
  ((-1 + (1 + r (-1 + S)) (1 - k - ℒ) + (1 - ℒ) (1 + (-1 + r - r S) (1 - k - ℒ) + (1 + r (-1 + S)) (-k - ℒ) α2))
    (1 + (1 - k) (-1 + β1))) -
(1 - k - r + k r - ℒ + r ℒ) β2 - λ1))} /. λ0 → 1/2 (2 - k - r + k
r - 2 ℒ + r ℒ - α2 + r α2 + ℒ
α2 - r
ℒ
α2 +
√(-4 (-1 + r) (-1 + ℒ) (-1 + k + ℒ) (-1 + α2) +
   (k (-1 + r) + (-2 + r) (-1 + ℒ) + (-1 + r + ℒ - r ℒ) α2)^2));
```

```
Solve[% == 0, λ1];
```

```
Simplify[%];
```

Solving for third eigenvalue approximated to first order:

```
{(-((-k (1 - k - r + k r - ℒ + r ℒ) ((z (-1 + (1 + r (-1 + S)) (1 - k - ℒ)) α2) / (-1 + (1 + r (-1 + S)) (1 - k - ℒ) + (1 - ℒ)
          (1 + (-1 + r - r S) (1 - k - ℒ) + (1 + r (-1 + S)) (-k - ℒ) α2)) - ((1 + r (-1 + S)) z (1 - k - ℒ) α2) /
        (-1 + (1 + r (-1 + S)) (1 - k - ℒ) + (1 - ℒ) (1 + (-1 + r - r S) (1 - k - ℒ) + (1 + r (-1 + S)) (-k - ℒ) α2)))
     (-1 + β1) - (z - k z + (r z (-1 + (1 + r (-1 + S)) (1 - k - ℒ)) (1 - k - ℒ)) /
        (-1 + (1 + r (-1 + S)) (1 - k - ℒ) + (1 - ℒ) (1 + (-1 + r - r S) (1 - k - ℒ) + (1 + r (-1 + S)) (-k - ℒ) α2)) -
       (r (1 + r (-1 + S)) z (1 - k - ℒ)^2 α2) / (-1 + (1 + r (-1 + S)) (1 - k - ℒ) +
         (1 - ℒ) (1 + (-1 + r - r S) (1 - k - ℒ) + (1 + r (-1 + S)) (-k - ℒ) α2))) (-1 + β1))
   (-((r - k r) (r - r ℒ) α2) + (r - k r) (1 - ℒ - α2 + r α2 + ℒ α2 - r ℒ α2 - λ0))) - (r - r ℒ)
```

$$(-r (-1 + k + \mathcal{L}) \alpha_1 + r (1 - k - \mathcal{L}) ((z (-1 + (1 + r (-1 + S)) (1 - k - \mathcal{L})) \alpha_2) / (-1 + (1 + r (-1 + S)) (1 - k - \mathcal{L}) +$$
$$(1 - \mathcal{L}) (1 + (-1 + r - r S) (1 - k - \mathcal{L}) + (1 + r (-1 + S)) (-k - \mathcal{L}) \alpha_2)) - ((1 + r (-1 + S)) z (1 - k - \mathcal{L}) \alpha_2) /$$
$$(-1 + (1 + r (-1 + S)) (1 - k - \mathcal{L}) + (1 - \mathcal{L}) (1 + (-1 + r - r S) (1 - k - \mathcal{L}) + (1 + r (-1 + S)) (-k - \mathcal{L}) \alpha_2))) +$$
$$(1 - k - r + k r - \mathcal{L} + r \mathcal{L}) (\mathcal{L} + k \alpha_2) ((z (-1 + (1 + r (-1 + S)) (1 - k - \mathcal{L})) \alpha_2) / (-1 + (1 + r (-1 + S)) (1 - k - \mathcal{L}) +$$
$$(1 - \mathcal{L}) (1 + (-1 + r - r S) (1 - k - \mathcal{L}) + (1 + r (-1 + S)) (-k - \mathcal{L}) \alpha_2)) - ((1 + r (-1 + S)) z (1 - k - \mathcal{L}) \alpha_2) /$$
$$(-1 + (1 + r (-1 + S)) (1 - k - \mathcal{L}) + (1 - \mathcal{L}) (1 + (-1 + r - r S) (1 - k - \mathcal{L}) + (1 + r (-1 + S)) (-k - \mathcal{L}) \alpha_2))) -$$
$$\alpha_2 (y - k y - r y + k r y - r z + k r z - y \mathcal{L} + r y \mathcal{L} - (r z (-1 + (1 + r (-1 + S)) (1 - k - \mathcal{L})) (1 - k - \mathcal{L})) /$$
$$(-1 + (1 + r (-1 + S)) (1 - k - \mathcal{L}) + (1 - \mathcal{L}) (1 + (-1 + r - r S) (1 - k - \mathcal{L}) + (1 + r (-1 + S)) (-k - \mathcal{L}) \alpha_2)) +$$
$$(r (1 + r (-1 + S)) z (1 - k - \mathcal{L})^2 \alpha_2) / (-1 + (1 + r (-1 + S)) (1 - k - \mathcal{L}) +$$
$$(1 - \mathcal{L}) (1 + (-1 + r - r S) (1 - k - \mathcal{L}) + (1 + r (-1 + S)) (-k - \mathcal{L}) \alpha_2))) -$$
$$(-1 + k + r - k r + \mathcal{L} - r \mathcal{L}) \alpha_2 (k \alpha_1 + (z (-1 + (1 + r (-1 + S)) (1 - k - \mathcal{L})) \mathcal{L}) /$$
$$(-1 + (1 + r (-1 + S)) (1 - k - \mathcal{L}) + (1 - \mathcal{L}) (1 + (-1 + r - r S) (1 - k - \mathcal{L}) + (1 + r (-1 + S)) (-k - \mathcal{L}) \alpha_2)) +$$
$$(k z (-1 + (1 + r (-1 + S)) (1 - k - \mathcal{L})) \alpha_2) / (-1 + (1 + r (-1 + S)) (1 - k - \mathcal{L}) + (1 - \mathcal{L})$$
$$(1 + (-1 + r - r S) (1 - k - \mathcal{L}) + (1 + r (-1 + S)) (-k - \mathcal{L}) \alpha_2)) - (k (1 + r (-1 + S)) z (1 - k - \mathcal{L}) \alpha_2) /$$
$$(-1 + (1 + r (-1 + S)) (1 - k - \mathcal{L}) + (1 - \mathcal{L}) (1 + (-1 + r - r S) (1 - k - \mathcal{L}) + (1 + r (-1 + S)) (-k - \mathcal{L}) \alpha_2)) -$$
$$((1 + r (-1 + S)) z (1 - k - \mathcal{L}) \mathcal{L} \alpha_2) / (-1 + (1 + r (-1 + S)) (1 - k - \mathcal{L}) +$$
$$(1 - \mathcal{L}) (1 + (-1 + r - r S) (1 - k - \mathcal{L}) + (1 + r (-1 + S)) (-k - \mathcal{L}) \alpha_2)) -$$
$$((1 - k) k (r (-1 + S) z \alpha_2 + \alpha_1 (-k - r (1 - k - \mathcal{L}) + r S (1 - k - \mathcal{L}) - \mathcal{L} + (1 - \mathcal{L}) (1 + (-1 + r - r S)$$
$$(1 - k - \mathcal{L}) + (1 + r (-1 + S)) (-k - \mathcal{L}) \alpha_2))) (-1 + \beta_1)) / ((-1 + (1 + r (-1 + S)) (1 - k - \mathcal{L}) +$$
$$(1 - \mathcal{L}) (1 + (-1 + r - r S) (1 - k - \mathcal{L}) + (1 + r (-1 + S)) (-k - \mathcal{L}) \alpha_2)) (1 + (1 - k) (-1 + \beta_1)))) -$$
$$((1 - k) r (1 - k - \mathcal{L}) (r (-1 + S) z \alpha_2 + \alpha_1 (-k - r (1 - k - \mathcal{L}) + r S (1 - k - \mathcal{L}) - \mathcal{L} +$$
$$(1 - \mathcal{L}) (1 + (-1 + r - r S) (1 - k - \mathcal{L}) + (1 + r (-1 + S)) (-k - \mathcal{L}) \alpha_2))) (-1 + \beta_1)) /$$
$$((-1 + (1 + r (-1 + S)) (1 - k - \mathcal{L}) + (1 - \mathcal{L}) (1 + (-1 + r - r S) (1 - k - \mathcal{L}) + (1 + r (-1 + S)) (-k - \mathcal{L}) \alpha_2))$$
$$(1 + (1 - k) (-1 + \beta_1)))) ((-1 + k) (-1 + \beta_1) - \lambda_0) -$$
$$(-1 + k + r - k r + \mathcal{L} - r \mathcal{L}) \alpha_2 ((r - k r) ((z - (r z (-1 + (1 + r (-1 + S)) (1 - k - \mathcal{L})) (-1 + \mathcal{L})) / (-1 +$$
$$(1 + r (-1 + S)) (1 - k - \mathcal{L}) + (1 - \mathcal{L}) (1 + (-1 + r - r S) (1 - k - \mathcal{L}) + (1 + r (-1 + S)) (-k - \mathcal{L}) \alpha_2)) +$$
$$(r (1 + r (-1 + S)) z (1 - k - \mathcal{L}) (-1 + \mathcal{L}) \alpha_2) / (-1 + (1 + r (-1 + S)) (1 - k - \mathcal{L}) +$$
$$(1 - \mathcal{L}) (1 + (-1 + r - r S) (1 - k - \mathcal{L}) + (1 + r (-1 + S)) (-k - \mathcal{L}) \alpha_2))) (-1 + \beta_1) -$$
$$k (z + (z (-1 + (1 + r (-1 + S)) (1 - k - \mathcal{L}))) / (-1 + (1 + r (-1 + S)) (1 - k - \mathcal{L}) + (1 - \mathcal{L}) (1 + (-1 + r - r S)$$
$$(1 - k - \mathcal{L}) + (1 + r (-1 + S)) (-k - \mathcal{L}) \alpha_2)) - (z (-1 + (1 + r (-1 + S)) (1 - k - \mathcal{L})) \mathcal{L}) / (-1 +$$
$$(1 + r (-1 + S)) (1 - k - \mathcal{L}) + (1 - \mathcal{L}) (1 + (-1 + r - r S) (1 - k - \mathcal{L}) + (1 + r (-1 + S)) (-k - \mathcal{L}) \alpha_2)) -$$
$$(z (-1 + (1 + r (-1 + S)) (1 - k - \mathcal{L})) \alpha_2) / (-1 + (1 + r (-1 + S)) (1 - k - \mathcal{L}) +$$
$$(1 - \mathcal{L}) (1 + (-1 + r - r S) (1 - k - \mathcal{L}) + (1 + r (-1 + S)) (-k - \mathcal{L}) \alpha_2)) +$$
$$(r z (-1 + (1 + r (-1 + S)) (1 - k - \mathcal{L})) \alpha_2) / (-1 + (1 + r (-1 + S)) (1 - k - \mathcal{L}) +$$
$$(1 - \mathcal{L}) (1 + (-1 + r - r S) (1 - k - \mathcal{L}) + (1 + r (-1 + S)) (-k - \mathcal{L}) \alpha_2)) -$$
$$(r (1 + r (-1 + S)) z (1 - k - \mathcal{L}) \alpha_2) / (-1 + (1 + r (-1 + S)) (1 - k - \mathcal{L}) +$$
$$(1 - \mathcal{L}) (1 + (-1 + r - r S) (1 - k - \mathcal{L}) + (1 + r (-1 + S)) (-k - \mathcal{L}) \alpha_2)) +$$
$$(z (-1 + (1 + r (-1 + S)) (1 - k - \mathcal{L})) \mathcal{L} \alpha_2) / (-1 + (1 + r (-1 + S)) (1 - k - \mathcal{L}) +$$

$$(1 - \mathcal{L}) (1 + (-1 + r - r S) (1 - k - \mathcal{L}) + (1 + r (-1 + S)) (-k - \mathcal{L}) \alpha_2)) -$$
$$(r z (-1 + (1 + r (-1 + S)) (1 - k - \mathcal{L})) \mathcal{L} \alpha_2) / (-1 + (1 + r (-1 + S)) (1 - k - \mathcal{L}) +$$
$$(1 - \mathcal{L}) (1 + (-1 + r - r S) (1 - k - \mathcal{L}) + (1 + r (-1 + S)) (-k - \mathcal{L}) \alpha_2)) +$$
$$(r (1 + r (-1 + S)) z (1 - k - \mathcal{L}) \mathcal{L} \alpha_2) / (-1 + (1 + r (-1 + S)) (1 - k - \mathcal{L}) +$$
$$(1 - \mathcal{L}) (1 + (-1 + r - r S) (1 - k - \mathcal{L}) + (1 + r (-1 + S)) (-k - \mathcal{L}) \alpha_2))) (-1 + \beta_1)) -$$
$$(-r y - z - r z + r y \mathcal{L} + r (-1 + \mathcal{L}) \alpha_1 - (r z (-1 + (1 + r (-1 + S)) (1 - k - \mathcal{L}))) /$$
$$(-1 + (1 + r (-1 + S)) (1 - k - \mathcal{L}) + (1 - \mathcal{L}) (1 + (-1 + r - r S) (1 - k - \mathcal{L}) + (1 + r (-1 + S)) (-k - \mathcal{L}) \alpha_2)) +$$
$$(r z (-1 + (1 + r (-1 + S)) (1 - k - \mathcal{L})) \mathcal{L}) / (-1 + (1 + r (-1 + S)) (1 - k - \mathcal{L}) +$$
$$(1 - \mathcal{L}) (1 + (-1 + r - r S) (1 - k - \mathcal{L}) + (1 + r (-1 + S)) (-k - \mathcal{L}) \alpha_2)) -$$
$$(r z (-1 + (1 + r (-1 + S)) (1 - k - \mathcal{L})) \alpha_2) / (-1 + (1 + r (-1 + S)) (1 - k - \mathcal{L}) + (1 - \mathcal{L})$$
$$(1 + (-1 + r - r S) (1 - k - \mathcal{L}) + (1 + r (-1 + S)) (-k - \mathcal{L}) \alpha_2)) + (2 r (1 + r (-1 + S)) z (1 - k - \mathcal{L}) \alpha_2) /$$
$$(-1 + (1 + r (-1 + S)) (1 - k - \mathcal{L}) + (1 - \mathcal{L}) (1 + (-1 + r - r S) (1 - k - \mathcal{L}) + (1 + r (-1 + S)) (-k - \mathcal{L}) \alpha_2)) +$$
$$(r z (-1 + (1 + r (-1 + S)) (1 - k - \mathcal{L})) \mathcal{L} \alpha_2) / (-1 + (1 + r (-1 + S)) (1 - k - \mathcal{L}) +$$
$$(1 - \mathcal{L}) (1 + (-1 + r - r S) (1 - k - \mathcal{L}) + (1 + r (-1 + S)) (-k - \mathcal{L}) \alpha_2)) -$$
$$(2 r (1 + r (-1 + S)) z (1 - k - \mathcal{L}) \mathcal{L} \alpha_2) / (-1 + (1 + r (-1 + S)) (1 - k - \mathcal{L}) +$$
$$(1 - \mathcal{L}) (1 + (-1 + r - r S) (1 - k - \mathcal{L}) + (1 + r (-1 + S)) (-k - \mathcal{L}) \alpha_2)) -$$
$$(-k - \mathcal{L}) (z + (z (-1 + (1 + r (-1 + S)) (1 - k - \mathcal{L}))) / (-1 + (1 + r (-1 + S)) (1 - k - \mathcal{L}) +$$
$$(1 - \mathcal{L}) (1 + (-1 + r - r S) (1 - k - \mathcal{L}) + (1 + r (-1 + S)) (-k - \mathcal{L}) \alpha_2)) -$$
$$(z (-1 + (1 + r (-1 + S)) (1 - k - \mathcal{L})) \mathcal{L}) / (-1 + (1 + r (-1 + S)) (1 - k - \mathcal{L}) +$$
$$(1 - \mathcal{L}) (1 + (-1 + r - r S) (1 - k - \mathcal{L}) + (1 + r (-1 + S)) (-k - \mathcal{L}) \alpha_2)) -$$
$$(z (-1 + (1 + r (-1 + S)) (1 - k - \mathcal{L})) \alpha_2) / (-1 + (1 + r (-1 + S)) (1 - k - \mathcal{L}) +$$
$$(1 - \mathcal{L}) (1 + (-1 + r - r S) (1 - k - \mathcal{L}) + (1 + r (-1 + S)) (-k - \mathcal{L}) \alpha_2)) +$$
$$(r z (-1 + (1 + r (-1 + S)) (1 - k - \mathcal{L})) \alpha_2) / (-1 + (1 + r (-1 + S)) (1 - k - \mathcal{L}) +$$
$$(1 - \mathcal{L}) (1 + (-1 + r - r S) (1 - k - \mathcal{L}) + (1 + r (-1 + S)) (-k - \mathcal{L}) \alpha_2)) -$$
$$(r (1 + r (-1 + S)) z (1 - k - \mathcal{L}) \alpha_2) / (-1 + (1 + r (-1 + S)) (1 - k - \mathcal{L}) +$$
$$(1 - \mathcal{L}) (1 + (-1 + r - r S) (1 - k - \mathcal{L}) + (1 + r (-1 + S)) (-k - \mathcal{L}) \alpha_2)) +$$
$$(z (-1 + (1 + r (-1 + S)) (1 - k - \mathcal{L})) \mathcal{L} \alpha_2) / (-1 + (1 + r (-1 + S)) (1 - k - \mathcal{L}) +$$
$$(1 - \mathcal{L}) (1 + (-1 + r - r S) (1 - k - \mathcal{L}) + (1 + r (-1 + S)) (-k - \mathcal{L}) \alpha_2)) -$$
$$(r z (-1 + (1 + r (-1 + S)) (1 - k - \mathcal{L})) \mathcal{L} \alpha_2) / (-1 + (1 + r (-1 + S)) (1 - k - \mathcal{L}) +$$
$$(1 - \mathcal{L}) (1 + (-1 + r - r S) (1 - k - \mathcal{L}) + (1 + r (-1 + S)) (-k - \mathcal{L}) \alpha_2)) +$$
$$(r (1 + r (-1 + S)) z (1 - k - \mathcal{L}) \mathcal{L} \alpha_2) / (-1 + (1 + r (-1 + S)) (1 - k - \mathcal{L}) +$$
$$(1 - \mathcal{L}) (1 + (-1 + r - r S) (1 - k - \mathcal{L}) + (1 + r (-1 + S)) (-k - \mathcal{L}) \alpha_2))) -$$
$$(r - r \mathcal{L}) (-k \alpha_1 - (z (-1 + (1 + r (-1 + S)) (1 - k - \mathcal{L})) \mathcal{L}) / (-1 + (1 + r (-1 + S)) (1 - k - \mathcal{L}) +$$
$$(1 - \mathcal{L}) (1 + (-1 + r - r S) (1 - k - \mathcal{L}) + (1 + r (-1 + S)) (-k - \mathcal{L}) \alpha_2)) -$$
$$(k z (-1 + (1 + r (-1 + S)) (1 - k - \mathcal{L})) \alpha_2) / (-1 + (1 + r (-1 + S)) (1 - k - \mathcal{L}) +$$
$$(1 - \mathcal{L}) (1 + (-1 + r - r S) (1 - k - \mathcal{L}) + (1 + r (-1 + S)) (-k - \mathcal{L}) \alpha_2)) +$$
$$(k (1 + r (-1 + S)) z (1 - k - \mathcal{L}) \alpha_2) / (-1 + (1 + r (-1 + S)) (1 - k - \mathcal{L}) +$$
$$(1 - \mathcal{L}) (1 + (-1 + r - r S) (1 - k - \mathcal{L}) + (1 + r (-1 + S)) (-k - \mathcal{L}) \alpha_2)) +$$

$$((1 + r (-1 + S)) z (1 - k - \mathcal{L}) \mathcal{L} \alpha_2) / (-1 + (1 + r (-1 + S)) (1 - k - \mathcal{L}) + (1 - \mathcal{L})$$
$$(1 + (-1 + r - r S) (1 - k - \mathcal{L}) + (1 + r (-1 + S)) (-k - \mathcal{L}) \alpha_2)) + ((1 - k) k (r (-1 + S) z \alpha_2 +$$
$$\alpha_1 (-k - r (1 - k - \mathcal{L}) + r S (1 - k - \mathcal{L}) - \mathcal{L} + (1 - \mathcal{L}) (1 + (-1 + r - r S) (1 - k - \mathcal{L}) +$$
$$(1 + r (-1 + S)) (-k - \mathcal{L}) \alpha_2))) (-1 + \beta_1)) / ((-1 + (1 + r (-1 + S)) (1 - k - \mathcal{L}) + (1 - \mathcal{L})$$
$$(1 + (-1 + r - r S) (1 - k - \mathcal{L}) + (1 + r (-1 + S)) (-k - \mathcal{L}) \alpha_2)) (1 + (1 - k) (-1 + \beta_1)))) -$$
$$((1 - k) r (-1 + \mathcal{L}) (r (-1 + S) z \alpha_2 + \alpha_1 (-k - r (1 - k - \mathcal{L}) + r S (1 - k - \mathcal{L}) - \mathcal{L} + (1 - \mathcal{L})$$
$$(1 + (-1 + r - r S) (1 - k - \mathcal{L}) + (1 + r (-1 + S)) (-k - \mathcal{L}) \alpha_2))) (-1 + \beta_1)) /$$
$$((-1 + (1 + r (-1 + S)) (1 - k - \mathcal{L}) + (1 - \mathcal{L}) (1 + (-1 + r - r S) (1 - k - \mathcal{L}) + (1 + r (-1 + S)) (-k - \mathcal{L}) \alpha_2))$$
$$(1 + (1 - k) (-1 + \beta_1))) + \beta_2 - r \beta_2 - \mathcal{L} \beta_2 + r \mathcal{L} \beta_2) ((-1 + k) (-1 + \beta_1) - \lambda_0) -$$
$$(r - r \mathcal{L}) ((-1 + k) \alpha_1 + (z - k z + ((1 - k) r z (-1 + (1 + r (-1 + S)) (1 - k - \mathcal{L}))) / (-1 +$$
$$(1 + r (-1 + S)) (1 - k - \mathcal{L}) + (1 - \mathcal{L}) (1 + (-1 + r - r S) (1 - k - \mathcal{L}) + (1 + r (-1 + S)) (-k - \mathcal{L}) \alpha_2)) -$$
$$((1 - k) r (1 + r (-1 + S)) z (1 - k - \mathcal{L}) \alpha_2) / (-1 + (1 + r (-1 + S)) (1 - k - \mathcal{L}) +$$
$$(1 - \mathcal{L}) (1 + (-1 + r - r S) (1 - k - \mathcal{L}) + (1 + r (-1 + S)) (-k - \mathcal{L}) \alpha_2))) (-1 + \beta_1) -$$
$$k (-((-1 + k) \alpha_1) - (-r + k r) ((z (-1 + (1 + r (-1 + S)) (1 - k - \mathcal{L})) \alpha_2) / (-1 + (1 + r (-1 + S))$$
$$(1 - k - \mathcal{L}) + (1 - \mathcal{L}) (1 + (-1 + r - r S) (1 - k - \mathcal{L}) + (1 + r (-1 + S)) (-k - \mathcal{L}) \alpha_2)) -$$
$$((1 + r (-1 + S)) z (1 - k - \mathcal{L}) \alpha_2) / (-1 + (1 + r (-1 + S)) (1 - k - \mathcal{L}) +$$
$$(1 - \mathcal{L}) (1 + (-1 + r - r S) (1 - k - \mathcal{L}) + (1 + r (-1 + S)) (-k - \mathcal{L}) \alpha_2))) +$$
$$((1 - k) (-1 + k) (r (-1 + S) z \alpha_2 + \alpha_1 (-k - r (1 - k - \mathcal{L}) + r S (1 - k - \mathcal{L}) - \mathcal{L} +$$
$$(1 - \mathcal{L}) (1 + (-1 + r - r S) (1 - k - \mathcal{L}) + (1 + r (-1 + S)) (-k - \mathcal{L}) \alpha_2)))$$
$$(-1 + \beta_1)) / ((-1 + (1 + r (-1 + S)) (1 - k - \mathcal{L}) + (1 - \mathcal{L}) (1 + (-1 + r - r S) (1 - k - \mathcal{L}) +$$
$$(1 + r (-1 + S)) (-k - \mathcal{L}) \alpha_2)) (1 + (1 - k) (-1 + \beta_1)))) (-1 + \beta_1) -$$
$$(-1 + k) (-k \alpha_1 - (z (-1 + (1 + r (-1 + S)) (1 - k - \mathcal{L})) \mathcal{L}) / (-1 + (1 + r (-1 + S)) (1 - k - \mathcal{L}) +$$
$$(1 - \mathcal{L}) (1 + (-1 + r - r S) (1 - k - \mathcal{L}) + (1 + r (-1 + S)) (-k - \mathcal{L}) \alpha_2)) -$$
$$(k z (-1 + (1 + r (-1 + S)) (1 - k - \mathcal{L})) \alpha_2) / (-1 + (1 + r (-1 + S)) (1 - k - \mathcal{L}) +$$
$$(1 - \mathcal{L}) (1 + (-1 + r - r S) (1 - k - \mathcal{L}) + (1 + r (-1 + S)) (-k - \mathcal{L}) \alpha_2)) +$$
$$(k (1 + r (-1 + S)) z (1 - k - \mathcal{L}) \alpha_2) / (-1 + (1 + r (-1 + S)) (1 - k - \mathcal{L}) +$$
$$(1 - \mathcal{L}) (1 + (-1 + r - r S) (1 - k - \mathcal{L}) + (1 + r (-1 + S)) (-k - \mathcal{L}) \alpha_2)) +$$
$$((1 + r (-1 + S)) z (1 - k - \mathcal{L}) \mathcal{L} \alpha_2) / (-1 + (1 + r (-1 + S)) (1 - k - \mathcal{L}) +$$
$$(1 - \mathcal{L}) (1 + (-1 + r - r S) (1 - k - \mathcal{L}) + (1 + r (-1 + S)) (-k - \mathcal{L}) \alpha_2)) +$$
$$((1 - k) k (r (-1 + S) z \alpha_2 + \alpha_1 (-k - r (1 - k - \mathcal{L}) + r S (1 - k - \mathcal{L}) - \mathcal{L} + (1 - \mathcal{L})$$
$$(1 + (-1 + r - r S) (1 - k - \mathcal{L}) + (1 + r (-1 + S)) (-k - \mathcal{L}) \alpha_2))) (-1 + \beta_1)) /$$
$$((-1 + (1 + r (-1 + S)) (1 - k - \mathcal{L}) + (1 - \mathcal{L}) (1 + (-1 + r - r S) (1 - k - \mathcal{L}) + (1 + r (-1 + S))$$
$$(-k - \mathcal{L}) \alpha_2)) (1 + (1 - k) (-1 + \beta_1)))) (-1 + \beta_1) - \lambda_1)) + (1 - k - r + k r - \mathcal{L} + r \mathcal{L} - \lambda_0)$$
$$(-((r - k r) \alpha_2 ((z - (r z (-1 + (1 + r (-1 + S)) (1 - k - \mathcal{L})) (-1 + \mathcal{L})) / (-1 + (1 + r (-1 + S)) (1 - k - \mathcal{L}) +$$
$$(1 - \mathcal{L}) (1 + (-1 + r - r S) (1 - k - \mathcal{L}) + (1 + r (-1 + S)) (-k - \mathcal{L}) \alpha_2)) +$$
$$(r (1 + r (-1 + S)) z (1 - k - \mathcal{L}) (-1 + \mathcal{L}) \alpha_2) / (-1 + (1 + r (-1 + S)) (1 - k - \mathcal{L}) +$$
$$(1 - \mathcal{L}) (1 + (-1 + r - r S) (1 - k - \mathcal{L}) + (1 + r (-1 + S)) (-k - \mathcal{L}) \alpha_2))) (-1 + \beta_1) -$$
$$k (z + (z (-1 + (1 + r (-1 + S)) (1 - k - \mathcal{L}))) / (-1 + (1 + r (-1 + S)) (1 - k - \mathcal{L}) +$$

$$(1 - \mathcal{L}) (1 + (-1 + r - r S) (1 - k - \mathcal{L}) + (1 + r (-1 + S)) (-k - \mathcal{L}) \alpha_2)) -$$
$$(z (-1 + (1 + r (-1 + S)) (1 - k - \mathcal{L})) \mathcal{L}) / (-1 + (1 + r (-1 + S)) (1 - k - \mathcal{L}) +$$
$$(1 - \mathcal{L}) (1 + (-1 + r - r S) (1 - k - \mathcal{L}) + (1 + r (-1 + S)) (-k - \mathcal{L}) \alpha_2)) -$$
$$(z (-1 + (1 + r (-1 + S)) (1 - k - \mathcal{L})) \alpha_2) / (-1 + (1 + r (-1 + S)) (1 - k - \mathcal{L}) +$$
$$(1 - \mathcal{L}) (1 + (-1 + r - r S) (1 - k - \mathcal{L}) + (1 + r (-1 + S)) (-k - \mathcal{L}) \alpha_2)) +$$
$$(r z (-1 + (1 + r (-1 + S)) (1 - k - \mathcal{L})) \alpha_2) / (-1 + (1 + r (-1 + S)) (1 - k - \mathcal{L}) +$$
$$(1 - \mathcal{L}) (1 + (-1 + r - r S) (1 - k - \mathcal{L}) + (1 + r (-1 + S)) (-k - \mathcal{L}) \alpha_2)) -$$
$$(r (1 + r (-1 + S)) z (1 - k - \mathcal{L}) \alpha_2) / (-1 + (1 + r (-1 + S)) (1 - k - \mathcal{L}) +$$
$$(1 - \mathcal{L}) (1 + (-1 + r - r S) (1 - k - \mathcal{L}) + (1 + r (-1 + S)) (-k - \mathcal{L}) \alpha_2)) +$$
$$(z (-1 + (1 + r (-1 + S)) (1 - k - \mathcal{L})) \mathcal{L} \alpha_2) / (-1 + (1 + r (-1 + S)) (1 - k - \mathcal{L}) +$$
$$(1 - \mathcal{L}) (1 + (-1 + r - r S) (1 - k - \mathcal{L}) + (1 + r (-1 + S)) (-k - \mathcal{L}) \alpha_2)) -$$
$$(r z (-1 + (1 + r (-1 + S)) (1 - k - \mathcal{L})) \mathcal{L} \alpha_2) / (-1 + (1 + r (-1 + S)) (1 - k - \mathcal{L}) +$$
$$(1 - \mathcal{L}) (1 + (-1 + r - r S) (1 - k - \mathcal{L}) + (1 + r (-1 + S)) (-k - \mathcal{L}) \alpha_2)) +$$
$$(r (1 + r (-1 + S)) z (1 - k - \mathcal{L}) \mathcal{L} \alpha_2) / (-1 + (1 + r (-1 + S)) (1 - k - \mathcal{L}) +$$
$$(1 - \mathcal{L}) (1 + (-1 + r - r S) (1 - k - \mathcal{L}) + (1 + r (-1 + S)) (-k - \mathcal{L}) \alpha_2))) (-1 + \beta_1)) +$$
$$((-1 + k) (-1 + \beta_1) - \lambda_0) (-y - z + y \mathcal{L} + r (-1 + \mathcal{L}) \alpha_1 + y \alpha_2 - r y \alpha_2 - r z \alpha_2 - y \mathcal{L} \alpha_2 +$$
$$r y \mathcal{L} \alpha_2 - (2 r z (-1 + (1 + r (-1 + S)) (1 - k - \mathcal{L})) \alpha_2) /$$
$$(-1 + (1 + r (-1 + S)) (1 - k - \mathcal{L}) + (1 - \mathcal{L}) (1 + (-1 + r - r S) (1 - k - \mathcal{L}) + (1 + r (-1 + S)) (-k - \mathcal{L}) \alpha_2)) +$$
$$(r (1 + r (-1 + S)) z (1 - k - \mathcal{L}) \alpha_2) / (-1 + (1 + r (-1 + S)) (1 - k - \mathcal{L}) +$$
$$(1 - \mathcal{L}) (1 + (-1 + r - r S) (1 - k - \mathcal{L}) + (1 + r (-1 + S)) (-k - \mathcal{L}) \alpha_2)) +$$
$$(2 r z (-1 + (1 + r (-1 + S)) (1 - k - \mathcal{L})) \mathcal{L} \alpha_2) / (-1 + (1 + r (-1 + S)) (1 - k - \mathcal{L}) +$$
$$(1 - \mathcal{L}) (1 + (-1 + r - r S) (1 - k - \mathcal{L}) + (1 + r (-1 + S)) (-k - \mathcal{L}) \alpha_2)) -$$
$$(r (1 + r (-1 + S)) z (1 - k - \mathcal{L}) \mathcal{L} \alpha_2) / (-1 + (1 + r (-1 + S)) (1 - k - \mathcal{L}) + (1 - \mathcal{L})$$
$$(1 + (-1 + r - r S) (1 - k - \mathcal{L}) + (1 + r (-1 + S)) (-k - \mathcal{L}) \alpha_2)) + (r (1 + r (-1 + S)) z (1 - k - \mathcal{L}) \alpha_2^2) /$$
$$(-1 + (1 + r (-1 + S)) (1 - k - \mathcal{L}) + (1 - \mathcal{L}) (1 + (-1 + r - r S) (1 - k - \mathcal{L}) + (1 + r (-1 + S)) (-k - \mathcal{L}) \alpha_2)) -$$
$$(r (1 + r (-1 + S)) z (1 - k - \mathcal{L}) \mathcal{L} \alpha_2^2) / (-1 + (1 + r (-1 + S)) (1 - k - \mathcal{L}) +$$
$$(1 - \mathcal{L}) (1 + (-1 + r - r S) (1 - k - \mathcal{L}) + (1 + r (-1 + S)) (-k - \mathcal{L}) \alpha_2)) -$$
$$(-\mathcal{L} - k \alpha_2) (z + (z (-1 + (1 + r (-1 + S)) (1 - k - \mathcal{L}))) / (-1 + (1 + r (-1 + S)) (1 - k - \mathcal{L}) +$$
$$(1 - \mathcal{L}) (1 + (-1 + r - r S) (1 - k - \mathcal{L}) + (1 + r (-1 + S)) (-k - \mathcal{L}) \alpha_2)) -$$
$$(z (-1 + (1 + r (-1 + S)) (1 - k - \mathcal{L})) \mathcal{L}) / (-1 + (1 + r (-1 + S)) (1 - k - \mathcal{L}) +$$
$$(1 - \mathcal{L}) (1 + (-1 + r - r S) (1 - k - \mathcal{L}) + (1 + r (-1 + S)) (-k - \mathcal{L}) \alpha_2)) -$$
$$(z (-1 + (1 + r (-1 + S)) (1 - k - \mathcal{L})) \alpha_2) / (-1 + (1 + r (-1 + S)) (1 - k - \mathcal{L}) +$$
$$(1 - \mathcal{L}) (1 + (-1 + r - r S) (1 - k - \mathcal{L}) + (1 + r (-1 + S)) (-k - \mathcal{L}) \alpha_2)) +$$
$$(r z (-1 + (1 + r (-1 + S)) (1 - k - \mathcal{L})) \alpha_2) / (-1 + (1 + r (-1 + S)) (1 - k - \mathcal{L}) +$$
$$(1 - \mathcal{L}) (1 + (-1 + r - r S) (1 - k - \mathcal{L}) + (1 + r (-1 + S)) (-k - \mathcal{L}) \alpha_2)) -$$
$$(r (1 + r (-1 + S)) z (1 - k - \mathcal{L}) \alpha_2) / (-1 + (1 + r (-1 + S)) (1 - k - \mathcal{L}) +$$
$$(1 - \mathcal{L}) (1 + (-1 + r - r S) (1 - k - \mathcal{L}) + (1 + r (-1 + S)) (-k - \mathcal{L}) \alpha_2)) +$$
$$(z (-1 + (1 + r (-1 + S)) (1 - k - \mathcal{L})) \mathcal{L} \alpha_2) / (-1 + (1 + r (-1 + S)) (1 - k - \mathcal{L}) +$$

$$(1 - \mathcal{L}) (1 + (-1 + r - r S) (1 - k - \mathcal{L}) + (1 + r (-1 + S)) (-k - \mathcal{L}) \alpha_2)) -$$
$$(r z (-1 + (1 + r (-1 + S)) (1 - k - \mathcal{L})) \mathcal{L} \alpha_2) / (-1 + (1 + r (-1 + S)) (1 - k - \mathcal{L}) +$$
$$(1 - \mathcal{L}) (1 + (-1 + r - r S) (1 - k - \mathcal{L}) + (1 + r (-1 + S)) (-k - \mathcal{L}) \alpha_2)) +$$
$$(r (1 + r (-1 + S)) z (1 - k - \mathcal{L}) \mathcal{L} \alpha_2) / (-1 + (1 + r (-1 + S)) (1 - k - \mathcal{L}) +$$
$$(1 - \mathcal{L}) (1 + (-1 + r - r S) (1 - k - \mathcal{L}) + (1 + r (-1 + S)) (-k - \mathcal{L}) \alpha_2))) -$$
$$(1 - \mathcal{L} - \alpha_2 + r \alpha_2 + \mathcal{L} \alpha_2 - r \mathcal{L} \alpha_2) (-k \alpha_1 - (z (-1 + (1 + r (-1 + S)) (1 - k - \mathcal{L})) \mathcal{L}) / (-1 +$$
$$(1 + r (-1 + S)) (1 - k - \mathcal{L}) + (1 - \mathcal{L}) (1 + (-1 + r - r S) (1 - k - \mathcal{L}) + (1 + r (-1 + S)) (-k - \mathcal{L}) \alpha_2)) -$$
$$(k z (-1 + (1 + r (-1 + S)) (1 - k - \mathcal{L})) \alpha_2) / (-1 + (1 + r (-1 + S)) (1 - k - \mathcal{L}) +$$
$$(1 - \mathcal{L}) (1 + (-1 + r - r S) (1 - k - \mathcal{L}) + (1 + r (-1 + S)) (-k - \mathcal{L}) \alpha_2)) +$$
$$(k (1 + r (-1 + S)) z (1 - k - \mathcal{L}) \alpha_2) / (-1 + (1 + r (-1 + S)) (1 - k - \mathcal{L}) +$$
$$(1 - \mathcal{L}) (1 + (-1 + r - r S) (1 - k - \mathcal{L}) + (1 + r (-1 + S)) (-k - \mathcal{L}) \alpha_2)) +$$
$$((1 + r (-1 + S)) z (1 - k - \mathcal{L}) \mathcal{L} \alpha_2) / (-1 + (1 + r (-1 + S)) (1 - k - \mathcal{L}) +$$
$$(1 - \mathcal{L}) (1 + (-1 + r - r S) (1 - k - \mathcal{L}) + (1 + r (-1 + S)) (-k - \mathcal{L}) \alpha_2)) +$$
$$((1 - k) k (r (-1 + S) z \alpha_2 + \alpha_1 (-k - r (1 - k - \mathcal{L}) + r S (1 - k - \mathcal{L}) - \mathcal{L} + (1 - \mathcal{L})$$
$$(1 + (-1 + r - r S) (1 - k - \mathcal{L}) + (1 + r (-1 + S)) (-k - \mathcal{L}) \alpha_2)))$$
$$(-1 + \beta_1)) / ((-1 + (1 + r (-1 + S)) (1 - k - \mathcal{L}) + (1 - \mathcal{L}) (1 + (-1 + r - r S) (1 - k - \mathcal{L}) +$$
$$(1 + r (-1 + S)) (-k - \mathcal{L}) \alpha_2)) (1 + (1 - k) (-1 + \beta_1)))) -$$
$$((1 - k) r (-1 + \mathcal{L}) (r (-1 + S) z \alpha_2 + \alpha_1 (-k - r (1 - k - \mathcal{L}) + r S (1 - k - \mathcal{L}) - \mathcal{L} + (1 - \mathcal{L})$$
$$(1 + (-1 + r - r S) (1 - k - \mathcal{L}) + (1 + r (-1 + S)) (-k - \mathcal{L}) \alpha_2))) (-1 + \beta_1)) /$$
$$((-1 + (1 + r (-1 + S)) (1 - k - \mathcal{L}) + (1 - \mathcal{L}) (1 + (-1 + r - r S) (1 - k - \mathcal{L}) + (1 + r (-1 + S)) (-k - \mathcal{L}) \alpha_2))$$
$$(1 + (1 - k) (-1 + \beta_1))) - \lambda_1) + (1 - \mathcal{L} - \alpha_2 + r \alpha_2 + \mathcal{L} \alpha_2 - r \mathcal{L} \alpha_2 - \lambda_0)$$
$$((-1 + k) \alpha_1 + (z - k z + ((1 - k) r z (-1 + (1 + r (-1 + S)) (1 - k - \mathcal{L}))) / (-1 + (1 + r (-1 + S))$$
$$(1 - k - \mathcal{L}) + (1 - \mathcal{L}) (1 + (-1 + r - r S) (1 - k - \mathcal{L}) + (1 + r (-1 + S)) (-k - \mathcal{L}) \alpha_2)) -$$
$$((1 - k) r (1 + r (-1 + S)) z (1 - k - \mathcal{L}) \alpha_2) / (-1 + (1 + r (-1 + S)) (1 - k - \mathcal{L}) +$$
$$(1 - \mathcal{L}) (1 + (-1 + r - r S) (1 - k - \mathcal{L}) + (1 + r (-1 + S)) (-k - \mathcal{L}) \alpha_2))) (-1 + \beta_1) -$$
$$k (-((-1 + k) \alpha_1) - (-r + k r) ((z (-1 + (1 + r (-1 + S)) (1 - k - \mathcal{L})) \alpha_2) / (-1 + (1 + r (-1 + S))$$
$$(1 - k - \mathcal{L}) + (1 - \mathcal{L}) (1 + (-1 + r - r S) (1 - k - \mathcal{L}) + (1 + r (-1 + S)) (-k - \mathcal{L}) \alpha_2)) -$$
$$((1 + r (-1 + S)) z (1 - k - \mathcal{L}) \alpha_2) / (-1 + (1 + r (-1 + S)) (1 - k - \mathcal{L}) +$$
$$(1 - \mathcal{L}) (1 + (-1 + r - r S) (1 - k - \mathcal{L}) + (1 + r (-1 + S)) (-k - \mathcal{L}) \alpha_2))) +$$
$$((1 - k) (-1 + k) (r (-1 + S) z \alpha_2 + \alpha_1 (-k - r (1 - k - \mathcal{L}) + r S (1 - k - \mathcal{L}) - \mathcal{L} +$$
$$(1 - \mathcal{L}) (1 + (-1 + r - r S) (1 - k - \mathcal{L}) + (1 + r (-1 + S)) (-k - \mathcal{L}) \alpha_2)))$$
$$(-1 + \beta_1)) / ((-1 + (1 + r (-1 + S)) (1 - k - \mathcal{L}) + (1 - \mathcal{L}) (1 + (-1 + r - r S) (1 - k - \mathcal{L}) +$$
$$(1 + r (-1 + S)) (-k - \mathcal{L}) \alpha_2)) (1 + (1 - k) (-1 + \beta_1)))) (-1 + \beta_1) -$$
$$(-1 + k) (-k \alpha_1 - (z (-1 + (1 + r (-1 + S)) (1 - k - \mathcal{L})) \mathcal{L}) / (-1 + (1 + r (-1 + S)) (1 - k - \mathcal{L}) +$$
$$(1 - \mathcal{L}) (1 + (-1 + r - r S) (1 - k - \mathcal{L}) + (1 + r (-1 + S)) (-k - \mathcal{L}) \alpha_2)) -$$
$$(k z (-1 + (1 + r (-1 + S)) (1 - k - \mathcal{L})) \alpha_2) / (-1 + (1 + r (-1 + S)) (1 - k - \mathcal{L}) +$$
$$(1 - \mathcal{L}) (1 + (-1 + r - r S) (1 - k - \mathcal{L}) + (1 + r (-1 + S)) (-k - \mathcal{L}) \alpha_2)) +$$
$$(k (1 + r (-1 + S)) z (1 - k - \mathcal{L}) \alpha_2) / (-1 + (1 + r (-1 + S)) (1 - k - \mathcal{L}) +$$

$$(1 - \mathcal{L}) (1 + (-1 + r - r S) (1 - k - \mathcal{L}) + (1 + r (-1 + S)) (-k - \mathcal{L}) \alpha_2)) +$$
$$((1 + r (-1 + S)) z (1 - k - \mathcal{L}) \mathcal{L} \alpha_2) / (-1 + (1 + r (-1 + S)) (1 - k - \mathcal{L}) +$$
$$(1 - \mathcal{L}) (1 + (-1 + r - r S) (1 - k - \mathcal{L}) + (1 + r (-1 + S)) (-k - \mathcal{L}) \alpha_2)) +$$
$$((1 - k) k (r (-1 + S) z \alpha_2 + \alpha_1 (-k - r (1 - k - \mathcal{L}) + r S (1 - k - \mathcal{L}) - \mathcal{L} + (1 - \mathcal{L})$$
$$(1 + (-1 + r - r S) (1 - k - \mathcal{L}) + (1 + r (-1 + S)) (-k - \mathcal{L}) \alpha_2)))$$
$$(-1 + \beta_1)) / ((-1 + (1 + r (-1 + S)) (1 - k - \mathcal{L}) + (1 - \mathcal{L}) (1 + (-1 + r - r S) (1 - k - \mathcal{L}) +$$
$$(1 + r (-1 + S)) (-k - \mathcal{L}) \alpha_2)) (1 + (1 - k) (-1 + \beta_1)))) (-1 + \beta_1) - \lambda_1)) +$$
$$(1 - \mathcal{L} - \alpha_2 + r \alpha_2 + \mathcal{L} \alpha_2 - r \mathcal{L} \alpha_2 - \lambda_0) ((-1 + k) (-1 + \beta_1) - \lambda_0) (-y + k y + r y -$$
$$k r y +$$
$$r z -$$
$$k r z +$$
$$y \mathcal{L} -$$
$$r y \mathcal{L} -$$
$$r (-1 + k + \mathcal{L}) \alpha_1 +$$
$$(r z (-1 + (1 + r (-1 + S)) (1 - k - \mathcal{L})) (1 - k - \mathcal{L})) /$$
$$(-1 + (1 + r (-1 + S)) (1 - k - \mathcal{L}) +$$
$$(1 - \mathcal{L}) (1 + (-1 + r - r S) (1 - k - \mathcal{L}) + (1 + r (-1 + S)) (-k - \mathcal{L}) \alpha_2)) -$$
$$(r (1 + r (-1 + S)) z (1 - k - \mathcal{L})^2 \alpha_2) / (-1 + (1 + r (-1 + S)) (1 - k - \mathcal{L}) +$$
$$(1 - \mathcal{L}) (1 + (-1 + r - r S) (1 - k - \mathcal{L}) + (1 + r (-1 + S)) (-k - \mathcal{L}) \alpha_2)) +$$
$$r (1 - k - \mathcal{L}) ((z (-1 + (1 + r (-1 + S)) (1 - k - \mathcal{L})) \alpha_2) / (-1 + (1 + r (-1 + S)) (1 - k - \mathcal{L}) +$$
$$(1 - \mathcal{L}) (1 + (-1 + r - r S) (1 - k - \mathcal{L}) + (1 + r (-1 + S)) (-k - \mathcal{L}) \alpha_2)) - ((1 + r (-1 + S)) z (1 - k - \mathcal{L}) \alpha_2) /$$
$$(-1 + (1 + r (-1 + S)) (1 - k - \mathcal{L}) + (1 - \mathcal{L}) (1 + (-1 + r - r S) (1 - k - \mathcal{L}) + (1 + r (-1 + S)) (-k - \mathcal{L}) \alpha_2))) +$$
$$(k + \mathcal{L}) (1 - k - r + k r - \mathcal{L} + r \mathcal{L}) ((z (-1 + (1 + r (-1 + S)) (1 - k - \mathcal{L})) \alpha_2) /$$
$$(-1 + (1 + r (-1 + S)) (1 - k - \mathcal{L}) + (1 - \mathcal{L}) (1 + (-1 + r - r S) (1 - k - \mathcal{L}) + (1 + r (-1 + S)) (-k - \mathcal{L}) \alpha_2)) -$$
$$((1 + r (-1 + S)) z (1 - k - \mathcal{L}) \alpha_2) / (-1 + (1 + r (-1 + S)) (1 - k - \mathcal{L}) +$$
$$(1 - \mathcal{L}) (1 + (-1 + r - r S) (1 - k - \mathcal{L}) + (1 + r (-1 + S)) (-k - \mathcal{L}) \alpha_2))) -$$
$$(-1 + k + r - k r + \mathcal{L} - r \mathcal{L}) (k \alpha_1 + (z (-1 + (1 + r (-1 + S)) (1 - k - \mathcal{L})) \mathcal{L}) /$$
$$(-1 + (1 + r (-1 + S)) (1 - k - \mathcal{L}) + (1 - \mathcal{L}) (1 + (-1 + r - r S) (1 - k - \mathcal{L}) + (1 + r (-1 + S)) (-k - \mathcal{L}) \alpha_2)) +$$
$$(k z (-1 + (1 + r (-1 + S)) (1 - k - \mathcal{L})) \alpha_2) / (-1 + (1 + r (-1 + S)) (1 - k - \mathcal{L}) +$$
$$(1 - \mathcal{L}) (1 + (-1 + r - r S) (1 - k - \mathcal{L}) + (1 + r (-1 + S)) (-k - \mathcal{L}) \alpha_2)) -$$
$$(k (1 + r (-1 + S)) z (1 - k - \mathcal{L}) \alpha_2) / (-1 + (1 + r (-1 + S)) (1 - k - \mathcal{L}) +$$
$$(1 - \mathcal{L}) (1 + (-1 + r - r S) (1 - k - \mathcal{L}) + (1 + r (-1 + S)) (-k - \mathcal{L}) \alpha_2)) -$$
$$((1 + r (-1 + S)) z (1 - k - \mathcal{L}) \mathcal{L} \alpha_2) / (-1 + (1 + r (-1 + S)) (1 - k - \mathcal{L}) +$$
$$(1 - \mathcal{L}) (1 + (-1 + r - r S) (1 - k - \mathcal{L}) + (1 + r (-1 + S)) (-k - \mathcal{L}) \alpha_2)) -$$
$$((1 - k) k (r (-1 + S) z \alpha_2 + \alpha_1 (-k - r (1 - k - \mathcal{L}) + r S (1 - k - \mathcal{L}) - \mathcal{L} + (1 - \mathcal{L})$$
$$(1 + (-1 + r - r S) (1 - k - \mathcal{L}) + (1 + r (-1 + S)) (-k - \mathcal{L}) \alpha_2))) (-1 + \beta_1)) /$$
$$((-1 + (1 + r (-1 + S)) (1 - k - \mathcal{L}) + (1 - \mathcal{L}) (1 + (-1 + r - r S) (1 - k - \mathcal{L}) + (1 + r (-1 + S)) (-k - \mathcal{L}) \alpha_2))$$
$$(1 + (1 - k) (-1 + \beta_1)))) -$$
$$((1 - k) r (1 - k - \mathcal{L}) (r (-1 + S) z \alpha_2 + \alpha_1 (-k - r (1 - k - \mathcal{L}) + r S (1 - k - \mathcal{L}) - \mathcal{L} +$$

```
(1 - ℒ) (1 + (-1 + r - r S) (1 - k - ℒ) + (1 + r (-1 + S)) (-k - ℒ) α2))) (-1 + β1)) /
((-1 + (1 + r (-1 + S)) (1 - k - ℒ) + (1 - ℒ) (1 + (-1 + r - r S) (1 - k - ℒ) + (1 + r (-1 + S)) (-k - ℒ) α2))
    (1 + (1 - k) (-1 + β1))) -
  (1 - k - r + k r - ℒ + r ℒ) β2 - λ1))} /. λ0 → (-1 + k) (-1 + β1);

Solve[% == 0, λ1];

Simplify[%];
```

Combining the zeroth and first order terms for each eigenvalue give the full first order approximation. For numerical parameter values evaluated in the main text and File S1 for case B, the eigenvalues indicated stability of the presented equilibria such that $|\lambda_i| < 1$.

B4 - Numerically evaluating accuracy of equilibria approximations

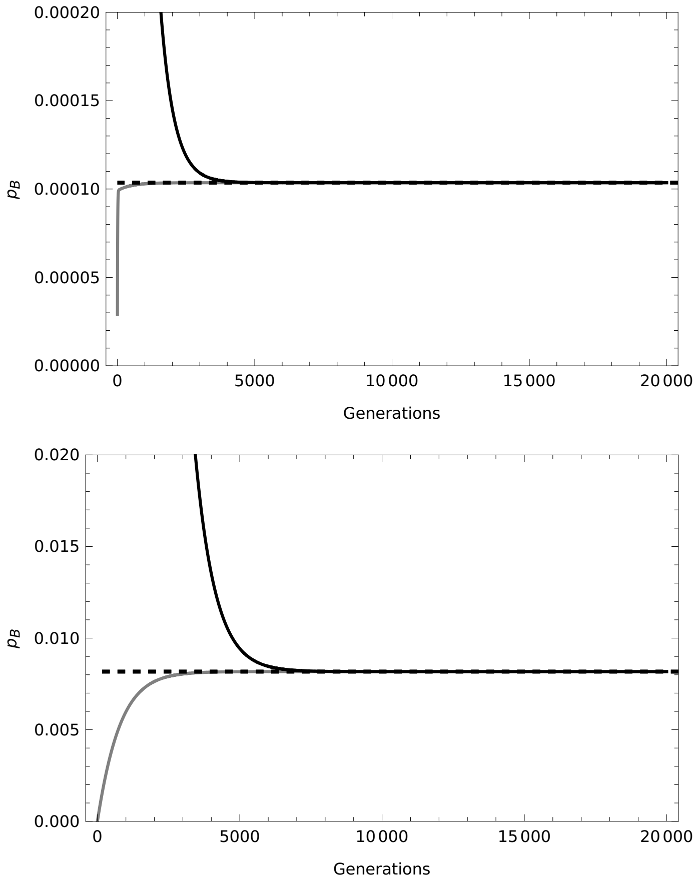

Figure S23: Numerical simulations of the recursion equations to assess equilibrium stability. The dashed line is the equilibrium point, the gray line starts above the equilibrium (p_B= 0.2), and the black line starts below the equilibrium (p_B= 2x10^{-5}). , The top panel is when β_1 = 0.1 > α_2= 0.0005 and bottom panel is when β_1 = 0.0005 < α_2= 0.005. The other parameter values are: α_1= 10^{-5}, β_2 = 10^{-5}, z = 10^{-6}, y = 10^{-5}, S = 0.01, r = 0.1, k = 0.001, $\mathcal{L}$ = 0.002.

Model 2: Three locus haploid mating and selection, full derivation

Derivation of Recursion Equations

Here we show frequencies of transient diploid genotypes that result from pairing of haploid organisms (1).

$$p_{J,t,A,J,t,A}{}^{(1)} = S\left((1-\alpha_1)^2 p_{J,t,A} + \beta_1^2 p_{J,t,B}\right) + (1-S)\left((1-\alpha_1)^2 p_{J,t,A}^2 + 2(1-\alpha_1)\beta_1 p_{J,t,A} p_{J,t,B} + \beta_1^2 p_{J,t,B}^2\right)$$

$$p_{J,t,A,J,t,B}{}^{(1)} = S\left(2(1-\alpha_1)\alpha_1 p_{J,t,A} + 2(1-\beta_1)\beta_1 p_{J,t,B}\right) + (1-S)\left(2(1-\alpha_1)\alpha_1 p_{J,t,A}^2 + 2(1-\alpha_1)(1-\beta_1) p_{J,t,A} p_{J,t,B} + 2\alpha_1\beta_1 p_{J,t,A} p_{J,t,B} + 2(1-\beta_1)\beta_1 p_{J,t,B}^2\right)$$

$$p_{J,t,B,J,t,B}{}^{(1)} = S\left(\alpha_1^2 p_{J,t,A} + (1-\beta_1)^2 p_{J,t,B}\right) + (1-S)\left(\alpha_1^2 p_{J,t,A}^2 + 2\alpha_1(1-\beta_1) p_{J,t,A} p_{J,t,B} + (1-\beta_1)^2 p_{J,t,B}^2\right)$$

$$p_{J,T,A,J,T,A}{}^{(1)} = S\left((1-\alpha_2)^2 p_{J,T,A} + \beta_2^2 p_{J,T,B}\right) + (1-S)\left((1-\alpha_2)^2 p_{J,T,A}^2 + 2(1-\alpha_2)\beta_2 p_{J,T,A} p_{J,T,B} + \beta_2^2 p_{J,T,B}^2\right)$$

$$p_{J,T,A,J,T,B}{}^{(1)} = S\left(2(1-\alpha_2)\alpha_2 p_{J,T,A} + 2(1-\beta_2)\beta_2 p_{J,T,B}\right) + (1-S)\left(2(1-\alpha_2)\alpha_2 p_{J,T,A}^2 + 2(1-\alpha_2)(1-\beta_2) p_{J,T,A} p_{J,T,B} + 2\alpha_2\beta_2 p_{J,T,A} p_{J,T,B} + 2(1-\beta_2)\beta_2 p_{J,T,B}^2\right)$$

$$p_{J,T,B,J,T,B}{}^{(1)} = S\left(\alpha_2^2 p_{J,T,A} + (1-\beta_2)^2 p_{J,T,B}\right) + (1-S)\left(\alpha_2^2 p_{J,T,A}^2 + 2\alpha_2(1-\beta_2) p_{J,T,A} p_{J,T,B} + (1-\beta_2)^2 p_{J,T,B}^2\right)$$

$$p_{J,t,A,J,T,A}{}^{(1)} = 2(1-S)\left((1-\alpha_1)(1-\alpha_2) p_{J,t,A} p_{J,T,A} + (1-\alpha_2)\beta_1 p_{J,t,B} p_{J,T,A} + (1-\alpha_1)\beta_2 p_{J,t,A} p_{J,T,B} + \beta_1\beta_2 p_{J,t,B} p_{J,T,B}\right)$$

$$p_{J,t,A,J,T,B}{}^{(1)} = 2(1-S)\left((1-\alpha_1)\alpha_2 p_{J,t,A} p_{J,T,A} + \alpha_2\beta_1 p_{J,t,B} p_{J,T,A} + (1-\alpha_1)(1-\beta_2) p_{J,t,A} p_{J,T,B} + \beta_1(1-\beta_2) p_{J,t,B} p_{J,T,B}\right)$$

$$p_{J,T,A,J,t,B}{}^{(1)} = 2(1-S)\left(\alpha_1(1-\alpha_2) p_{J,t,A} p_{J,T,A} + (1-\alpha_2)(1-\beta_1) p_{J,t,B} p_{J,T,A} + \alpha_1\beta_2 p_{J,t,A} p_{J,T,B} + (1-\beta_1)\beta_2 p_{J,t,B} p_{J,T,B}\right)$$

$$p_{J,t,B,J,T,B}{}^{(1)} = 2(1-S)\left(\alpha_1\alpha_2 p_{J,t,A} p_{J,T,A} + \alpha_2(1-\beta_1) p_{J,t,B} p_{J,T,A} + \alpha_1(1-\beta_2) p_{J,t,A} p_{J,T,B} + (1-\beta_1)(1-\beta_2) p_{J,t,B} p_{J,T,B}\right)$$

$$p_{j,t,A,j,t,A}{}^{(1)} = S\left((1-\alpha_1)^2 p_{j,t,A} + \beta_1^2 p_{j,t,B}\right) + (1-S)\left((1-\alpha_1)^2 p_{j,t,A}^2 + 2(1-\alpha_1)\beta_1 p_{j,t,A} p_{j,t,B} + \beta_1^2 p_{j,t,B}^2\right)$$

$$p_{j,t,A,j,t,B}{}^{(1)} = S\left(2(1-\alpha_1)\alpha_1 p_{j,t,A} + 2(1-\beta_1)\beta_1 p_{j,t,B}\right) + (1-S)\left(2(1-\alpha_1)\alpha_1 p_{j,t,A}^2 + 2(1-\alpha_1)(1-\beta_1) p_{j,t,A} p_{j,t,B} + 2\alpha_1\beta_1 p_{j,t,A} p_{j,t,B} + 2(1-\beta_1)\beta_1 p_{j,t,B}^2\right)$$

$$p_{j,t,B,j,t,B}{}^{(1)} = S\left(\alpha_1^2 p_{j,t,A} + (1-\beta_1)^2 p_{j,t,B}\right) + (1-S)\left(\alpha_1^2 p_{j,t,A}^2 + 2\alpha_1(1-\beta_1) p_{j,t,A} p_{j,t,B} + (1-\beta_1)^2 p_{j,t,B}^2\right)$$

$$p_{j,T,A,j,T,A}{}^{(1)} = S\left((1-\alpha_2)^2 p_{j,T,A} + \beta_2^2 p_{j,T,B}\right) + (1-S)\left((1-\alpha_2)^2 p_{j,T,A}^2 + 2(1-\alpha_2)\beta_2 p_{j,T,A} p_{j,T,B} + \beta_2^2 p_{j,T,B}^2\right)$$

$$p_{j,T,A,j,T,B}{}^{(1)} = S\left(2(1-\alpha_2)\alpha_2 p_{j,T,A} + 2(1-\beta_2)\beta_2 p_{j,T,B}\right) + (1-S)\left(2(1-\alpha_2)\alpha_2 p_{j,T,A}^2 + 2(1-\alpha_2)(1-\beta_2) p_{j,T,A} p_{j,T,B} + 2\alpha_2\beta_2 p_{j,T,A} p_{j,T,B} + 2(1-\beta_2)\beta_2 p_{j,T,B}^2\right)$$

$$p_{j,T,B,j,T,B}{}^{(1)} = S\left(\alpha_2^2 p_{j,T,A} + (1-\beta_2)^2 p_{j,T,B}\right) + (1-S)\left(\alpha_2^2 p_{j,T,A}^2 + 2\alpha_2(1-\beta_2) p_{j,T,A} p_{j,T,B} + (1-\beta_2)^2 p_{j,T,B}^2\right)$$

$$p_{j,t,A,j,T,A}{}^{(1)} = 2\,(1-S)\left((1-\alpha_1)\,(1-\alpha_2)\,p_{j,t,A}\,p_{j,T,A} + (1-\alpha_2)\,\beta_1\,p_{j,t,B}\,p_{j,T,A} + (1-\alpha_1)\,\beta_2\,p_{j,t,A}\,p_{j,T,B} + \beta_1\,\beta_2\,p_{j,t,B}\,p_{j,T,B}\right)$$

$$p_{j,t,A,j,T,B}{}^{(1)} = 2\,(1-S)\left((1-\alpha_1)\,\alpha_2\,p_{j,t,A}\,p_{j,T,A} + \alpha_2\,\beta_1\,p_{j,t,B}\,p_{j,T,A} + (1-\alpha_1)\,(1-\beta_2)\,p_{j,t,A}\,p_{j,T,B} + \beta_1\,(1-\beta_2)\,p_{j,t,B}\,p_{j,T,B}\right)$$

$$p_{j,T,A,j,t,B}{}^{(1)} = 2\,(1-S)\left(\alpha_1\,(1-\alpha_2)\,p_{j,t,A}\,p_{j,T,A} + (1-\alpha_2)\,(1-\beta_1)\,p_{j,t,B}\,p_{j,T,A} + \alpha_1\,\beta_2\,p_{j,t,A}\,p_{j,T,B} + (1-\beta_1)\,\beta_2\,p_{j,t,B}\,p_{j,T,B}\right)$$

$$p_{j,t,B,j,T,B}{}^{(1)} = 2\,(1-S)\left(\alpha_1\,\alpha_2\,p_{j,t,A}\,p_{j,T,A} + \alpha_2\,(1-\beta_1)\,p_{j,t,B}\,p_{j,T,A} + \alpha_1\,(1-\beta_2)\,p_{j,t,A}\,p_{j,T,B} + (1-\beta_1)\,(1-\beta_2)\,p_{j,t,B}\,p_{j,T,B}\right)$$

$$p_{J,t,A,j,t,A}{}^{(1)} = (1-S)\left((1-\alpha_1)\,(1-\alpha_1)\,2\,p_{J,t,A}\,p_{j,t,A} + (1-\alpha_1)\,\beta_1\,2\,p_{J,t,A}\,p_{j,t,B} + \beta_1\,(1-\alpha_1)\,2\,p_{J,t,B}\,p_{j,t,A} + \beta_1\,\beta_1\,2\,p_{J,t,B}\,p_{j,t,B}\right)$$

$$p_{J,t,A,j,t,B}{}^{(1)} = (1-S)\left((1-\alpha_1)\,\alpha_1\,2\,p_{J,t,A}\,p_{j,t,A} + (1-\alpha_1)\,(1-\beta_1)\,2\,p_{J,t,A}\,p_{j,t,B} + \beta_1\,\alpha_1\,2\,p_{J,t,B}\,p_{j,t,A} + \beta_1\,(1-\beta_1)\,2\,p_{J,t,B}\,p_{j,t,B}\right)$$

$$p_{J,t,B,j,t,A}{}^{(1)} = (1-S)\left(\alpha_1\,(1-\alpha_1)\,2\,p_{J,t,A}\,p_{j,t,A} + \alpha_1\,\beta_1\,2\,p_{J,t,A}\,p_{j,t,B} + (1-\beta_1)\,(1-\alpha_1)\,2\,p_{J,t,B}\,p_{j,t,A} + (1-\beta_1)\,\beta_1\,2\,p_{J,t,B}\,p_{j,t,B}\right)$$

$$p_{J,t,B,j,t,B}{}^{(1)} = (1-S)\left(\alpha_1\,\alpha_1\,2\,p_{J,t,A}\,p_{j,t,A} + \alpha_1\,(1-\beta_1)\,2\,p_{J,t,A}\,p_{j,t,B} + (1-\beta_1)\,\alpha_1\,2\,p_{J,t,B}\,p_{j,t,A} + (1-\beta_1)\,(1-\beta_1)\,2\,p_{J,t,B}\,p_{j,t,B}\right)$$

$$p_{J,t,A,j,T,A}{}^{(1)} = (1-S)\left((1-\alpha_1)\,(1-\alpha_2)\,2\,p_{J,t,A}\,p_{j,T,A} + (1-\alpha_1)\,\beta_2\,2\,p_{J,t,A}\,p_{j,T,B} + \beta_1\,(1-\alpha_2)\,2\,p_{J,t,B}\,p_{j,T,A} + \beta_1\,\beta_2\,2\,p_{J,t,B}\,p_{j,T,B}\right)$$

$$p_{J,t,A,j,T,B}{}^{(1)} = (1-S)\left((1-\alpha_1)\,\alpha_2\,2\,p_{J,t,A}\,p_{j,T,A} + (1-\alpha_1)\,(1-\beta_2)\,2\,p_{J,t,A}\,p_{j,T,B} + \beta_1\,\alpha_2\,2\,p_{J,t,B}\,p_{j,T,A} + \beta_1\,(1-\beta_2)\,2\,p_{J,t,B}\,p_{j,T,B}\right)$$

$$p_{J,t,B,j,T,A}{}^{(1)} = (1-S)\left(\alpha_1\,(1-\alpha_2)\,2\,p_{J,t,A}\,p_{j,T,A} + \alpha_1\,\beta_2\,2\,p_{J,t,A}\,p_{j,T,B} + (1-\beta_1)\,(1-\alpha_2)\,2\,p_{J,t,B}\,p_{j,T,A} + (1-\beta_1)\,\beta_2\,2\,p_{J,t,B}\,p_{j,T,B}\right)$$

$$p_{J,t,B,j,T,B}{}^{(1)} = (1-S)\left(\alpha_1\,\alpha_2\,2\,p_{J,t,A}\,p_{j,T,A} + \alpha_1\,(1-\beta_2)\,2\,p_{J,t,A}\,p_{j,T,B} + (1-\beta_1)\,\alpha_2\,2\,p_{J,t,B}\,p_{j,T,A} + (1-\beta_1)\,(1-\beta_2)\,2\,p_{J,t,B}\,p_{j,T,B}\right)$$

$$p_{J,T,A,j,t,A}{}^{(1)} = (1-S)\left((1-\alpha_2)\,(1-\alpha_1)\,2\,p_{J,T,A}\,p_{j,t,A} + (1-\alpha_2)\,\beta_1\,2\,p_{J,T,A}\,p_{j,t,B} + \beta_2\,(1-\alpha_1)\,2\,p_{J,T,B}\,p_{j,t,A} + \beta_2\,\beta_1\,2\,p_{J,T,B}\,p_{j,t,B}\right)$$

$$p_{J,T,A,j,t,B}{}^{(1)} = (1-S)\left((1-\alpha_2)\,\alpha_1\,2\,p_{J,T,A}\,p_{j,t,A} + (1-\alpha_2)\,(1-\beta_1)\,2\,p_{J,T,A}\,p_{j,t,B} + \beta_2\,\alpha_1\,2\,p_{J,T,B}\,p_{j,t,A} + \beta_2\,(1-\beta_1)\,2\,p_{J,T,B}\,p_{j,t,B}\right)$$

$$p_{J,T,B,j,t,A}{}^{(1)} = (1-S)\left(\alpha_2\,(1-\alpha_1)\,2\,p_{J,T,A}\,p_{j,t,A} + \alpha_2\,\beta_1\,2\,p_{J,T,A}\,p_{j,t,B} + (1-\beta_2)\,(1-\alpha_1)\,2\,p_{J,T,B}\,p_{j,t,A} + (1-\beta_2)\,\beta_1\,2\,p_{J,T,B}\,p_{j,t,B}\right)$$

$$p_{J,T,B,j,t,B}{}^{(1)} = (1-S)\left(\alpha_2\,\alpha_1\,2\,p_{J,T,A}\,p_{j,t,A} + \alpha_2\,(1-\beta_1)\,2\,p_{J,T,A}\,p_{j,t,B} + (1-\beta_2)\,\alpha_1\,2\,p_{J,T,B}\,p_{j,t,A} + (1-\beta_2)\,(1-\beta_1)\,2\,p_{J,T,B}\,p_{j,t,B}\right)$$

$$p_{J,T,A,j,T,A}{}^{(1)} = (1-S)\left((1-\alpha_2)\,(1-\alpha_2)\,2\,p_{J,T,A}\,p_{j,T,A} + (1-\alpha_2)\,\beta_2\,2\,p_{J,T,A}\,p_{j,T,B} + \beta_2\,(1-\alpha_2)\,2\,p_{J,T,B}\,p_{j,T,A} + \beta_2\,\beta_2\,2\,p_{J,T,B}\,p_{j,T,B}\right)$$

$$p_{J,T,A,j,T,B}{}^{(1)} = (1-S)\left((1-\alpha_2)\,\alpha_2\,2\,p_{J,T,A}\,p_{j,T,A} + (1-\alpha_2)\,(1-\beta_2)\,2\,p_{J,T,A}\,p_{j,T,B} + \beta_2\,\alpha_2\,2\,p_{J,T,B}\,p_{j,T,A} + \beta_2\,(1-\beta_2)\,2\,p_{J,T,B}\,p_{j,T,B}\right)$$

$$p_{J,T,B,j,T,A}{}^{(1)} = (1-S)\left(\alpha_2\,(1-\alpha_2)\,2\,p_{J,T,A}\,p_{j,T,A} + \alpha_2\,\beta_2\,2\,p_{J,T,A}\,p_{j,T,B} + (1-\beta_2)\,(1-\alpha_2)\,2\,p_{J,T,B}\,p_{j,T,A} + (1-\beta_2)\,\beta_2\,2\,p_{J,T,B}\,p_{j,T,B}\right)$$

$$p_{J,T,B,j,T,B}{}^{(1)} = (1-S)\left(\alpha_2\,\alpha_2\,2\,p_{J,T,A}\,p_{j,T,A} + \alpha_2\,(1-\beta_2)\,2\,p_{J,T,A}\,p_{j,T,B} + (1-\beta_2)\,\alpha_2\,2\,p_{J,T,B}\,p_{j,T,A} + (1-\beta_2)\,(1-\beta_2)\,2\,p_{J,T,B}\,p_{j,T,B}\right)$$

Recombination occurs generating next generation of haploid organisms (2):

(Note: There are 8 possible haploid organism (epi)genotypes)

$$p_{J,t,A}{}^{(2)} = p_{J,t,A,J,t,A}{}^{(1)} + 1/2\, p_{J,t,A,J,t,B}{}^{(1)} + 1/2\, p_{J,t,A,J,T,A}{}^{(1)} + \left((1-r_1)(1-r_2)\, 1/2 + r_1 (1-r_2)\, 1/2\right) p_{J,t,A,J,T,B}{}^{(1)} + \left((1-r_1)\, r_2\, 1/2 + r_1\, r_2\, 1/2\right) p_{J,T,A,J,t,B}{}^{(1)} + 1/2\, p_{J,t,A,j,t,A}{}^{(1)} + \left((1-r_3)(1-r_4)\, 1/2 + r_3\, r_4\, 1/2\right) p_{J,t,A,j,t,B}{}^{(1)} + \left(r_3 (1-r_4)\, 1/2 + (1-r_3)\, r_4\, 1/2\right) p_{J,t,B,j,t,A}{}^{(1)} + \left((1-r_3)(1-r_4)\, 1/2 + (1-r_3)\, r_4\, 1/2\right) p_{J,t,A,j,T,A}{}^{(1)} + (1-r_3)(1-r_4)\, 1/2\, p_{J,t,A,j,T,B}{}^{(1)} + (1-r_3)\, r_4\, 1/2\, p_{J,t,B,j,T,A}{}^{(1)} + \left(r_3 (1-r_4)\, 1/2 + r_3\, r_4\, 1/2\right) p_{J,T,A,j,t,A}{}^{(1)} + r_3\, r_4\, 1/2\, p_{J,T,A,j,t,B}{}^{(1)} + r_3 (1-r_4)\, 1/2\, p_{J,T,B,j,t,A}{}^{(1)}$$

$$p_{J,t,B}{}^{(2)} = p_{J,t,B,J,t,B}{}^{(1)} + 1/2\, p_{J,t,A,J,t,B}{}^{(1)} + \left((1-r_1)\, r_2\, 1/2 + r_1\, r_2\, 1/2\right) p_{J,t,A,J,T,B}{}^{(1)} + \left((1-r_1)(1-r_2)\, 1/2 + r_1 (1-r_2)\, 1/2\right) p_{J,T,A,J,t,B}{}^{(1)} + 1/2\, p_{J,t,B,J,T,B}{}^{(1)} + \left(r_3 (1-r_4)\, 1/2 + (1-r_3)\, r_4\, 1/2\right) p_{J,t,A,j,t,B}{}^{(1)} + \left((1-r_3)(1-r_4)\, 1/2 + r_3\, r_4\, 1/2\right) p_{J,t,B,j,t,A}{}^{(1)} + 1/2\, p_{J,t,B,j,t,B}{}^{(1)} + (1-r_3)\, r_4\, 1/2\, p_{J,t,A,j,T,B}{}^{(1)} + (1-r_3)(1-r_4)\, 1/2\, p_{J,t,B,j,T,A}{}^{(1)} + r_3 (1-r_4)\, 1/2\, p_{J,T,A,j,t,B}{}^{(1)} + r_3\, r_4\, 1/2\, p_{J,T,B,j,t,A}{}^{(1)} + \left(r_3 (1-r_4)\, 1/2 + r_3\, r_4\, 1/2\right) p_{J,T,B,j,t,B}{}^{(1)} + \left((1-r_3)(1-r_4)\, 1/2 + (1-r_3)\, r_4\, 1/2\right) p_{J,t,B,j,T,B}{}^{(1)}$$

$$p_{J,T,A}{}^{(2)} = p_{J,T,A,J,T,A}{}^{(1)} + 1/2\, p_{J,T,A,J,T,B}{}^{(1)} + 1/2\, p_{J,t,A,J,T,A}{}^{(1)} + \left((1-r_1)\, r_2\, 1/2 + r_1\, r_2\, 1/2\right) p_{J,t,A,J,T,B}{}^{(1)} + \left((1-r_1)(1-r_2)\, 1/2 + r_1 (1-r_2)\, 1/2\right) p_{J,T,A,J,t,B}{}^{(1)} + \left(r_3 (1-r_4)\, 1/2 + r_3\, r_4\, 1/2\right) p_{J,t,A,j,T,A}{}^{(1)} + r_3\, r_4\, 1/2\, p_{J,t,A,j,T,B}{}^{(1)} + r_3 (1-r_4)\, 1/2\, p_{J,t,B,j,T,A}{}^{(1)} + \left((1-r_3)(1-r_4)\, 1/2 + (1-r_3)\, r_4\, 1/2\right) p_{J,T,A,j,t,A}{}^{(1)} + (1-r_3)(1-r_4)\, 1/2\, p_{J,T,A,j,t,B}{}^{(1)} + (1-r_3)\, r_4\, 1/2\, p_{J,T,B,j,t,A}{}^{(1)} + 1/2\, p_{J,T,A,j,T,A}{}^{(1)} + \left((1-r_3)(1-r_4)\, 1/2 + r_3\, r_4\, 1/2\right) p_{J,T,A,j,T,B}{}^{(1)} + \left(r_3 (1-r_4)\, 1/2 + (1-r_3)\, r_4\, 1/2\right) p_{J,T,B,j,T,A}{}^{(1)}$$

$$p_{J,T,B}{}^{(2)} = p_{J,T,B,J,T,B}{}^{(1)} + 1/2\, p_{J,T,A,J,T,B}{}^{(1)} + \left((1-r_1)(1-r_2)\, 1/2 + r_1 (1-r_2)\, 1/2\right) p_{J,t,A,J,T,B}{}^{(1)} + \left((1-r_1)\, r_2\, 1/2 + r_1\, r_2\, 1/2\right) p_{J,T,A,J,t,B}{}^{(1)} + 1/2\, p_{J,t,B,J,T,B}{}^{(1)} + r_3 (1-r_4)\, 1/2\, p_{J,t,A,j,T,B}{}^{(1)} + r_3\, r_4\, 1/2\, p_{J,t,B,j,T,A}{}^{(1)} + \left(r_3 (1-r_4)\, 1/2 + r_3\, r_4\, 1/2\right) p_{J,t,B,j,T,B}{}^{(1)} + (1-r_3)\, r_4\, 1/2\, p_{J,T,A,j,t,B}{}^{(1)} + (1-r_3)(1-r_4)\, 1/2\, p_{J,T,B,j,t,A}{}^{(1)} + \left((1-r_3)(1-r_4)\, 1/2 + (1-r_3)\, r_4\, 1/2\right) p_{J,T,B,j,t,B}{}^{(1)} + \left(r_3 (1-r_4)\, 1/2 + (1-r_3)\, r_4\, 1/2\right) p_{J,T,A,j,T,B}{}^{(1)} + \left((1-r_3)(1-r_4)\, 1/2 + r_3\, r_4\, 1/2\right) p_{J,T,B,j,T,A}{}^{(1)} + 1/2\, p_{J,T,B,j,T,B}{}^{(1)}$$

$$p_{j,t,A}{}^{(2)} = p_{j,t,A,j,t,A}{}^{(1)} + 1/2\, p_{j,t,A,j,t,B}{}^{(1)} + 1/2\, p_{j,t,A,j,T,A}{}^{(1)} + \left((1-r_5)(1-r_6)\, 1/2 + r_5 (1-r_6)\, 1/2\right) p_{j,t,A,j,T,B}{}^{(1)} + \left((1-r_5)\, r_6\, 1/2 + r_5\, r_6\, 1/2\right) p_{j,T,A,j,t,B}{}^{(1)} + 1/2\, p_{J,t,A,j,t,A}{}^{(1)} + \left(r_3 (1-r_4)\, 1/2 + (1-r_3)\, r_4\, 1/2\right) p_{J,t,A,j,t,B}{}^{(1)} + \left((1-r_3)(1-r_4)\, 1/2 + r_3\, r_4\, 1/2\right) p_{J,t,B,j,t,A}{}^{(1)} + \left(r_3 (1-r_4)\, 1/2 + r_3\, r_4\, 1/2\right) p_{J,t,A,j,T,A}{}^{(1)} + r_3 (1-r_4)\, 1/2\, p_{J,t,A,j,T,B}{}^{(1)} + r_3\, r_4\, 1/2\, p_{J,t,B,j,T,A}{}^{(1)} + \left((1-r_3)(1-r_4)\, 1/2 + (1-r_3)\, r_4\, 1/2\right) p_{J,T,A,j,t,A}{}^{(1)} + (1-r_3)\, r_4\, 1/2\, p_{J,T,A,j,t,B}{}^{(1)} + (1-r_3)(1-r_4)\, 1/2\, p_{J,T,B,j,t,A}{}^{(1)}$$

$$p_{j,t,B}{}^{(2)} = p_{j,t,B,j,t,B}{}^{(1)} + 1/2\, p_{j,t,A,j,t,B}{}^{(1)} + \left((1-r_5)\, r_6\, 1/2 + r_5\, r_6\, 1/2\right) p_{j,t,A,j,T,B}{}^{(1)} + \left((1-r_5)(1-r_6)\, 1/2 + r_5 (1-r_6)\, 1/2\right) p_{j,T,A,j,t,B}{}^{(1)} + 1/2\, p_{j,t,B,j,T,B}{}^{(1)} + \left((1-r_3)(1-r_4)\, 1/2 + r_3\, r_4\, 1/2\right) p_{J,t,A,j,t,B}{}^{(1)} + \left(r_3 (1-r_4)\, 1/2 + (1-r_3)\, r_4\, 1/2\right) p_{J,t,B,j,t,A}{}^{(1)} + 1/2\, p_{J,t,B,j,t,B}{}^{(1)} + r_3\, r_4\, 1/2\, p_{J,t,A,j,T,B}{}^{(1)} + r_3 (1-r_4)\, 1/2\, p_{J,t,B,j,T,A}{}^{(1)} + \left(r_3 (1-r_4)\, 1/2 + r_3\, r_4\, 1/2\right) p_{J,t,B,j,T,B}{}^{(1)} + (1-r_3)(1-r_4)\, 1/2\, p_{J,T,A,j,t,B}{}^{(1)} + (1-r_3)\, r_4\, 1/2\, p_{J,T,B,j,t,A}{}^{(1)} + \left((1-r_3)(1-r_4)\, 1/2 + (1-r_3)\, r_4\, 1/2\right) p_{J,T,B,j,t,B}{}^{(1)}$$

$$p_{j,T,A}{}^{(2)} = p_{j,T,A,j,T,A}{}^{(1)} + 1/2\, p_{j,T,A,j,T,B}{}^{(1)} + 1/2\, p_{j,t,A,j,T,A}{}^{(1)} + \left((1-r_5)\, r_6\, 1/2 + r_5\, r_6\, 1/2\right) p_{j,t,A,j,T,B}{}^{(1)} + \left((1-r_5)(1-r_6)\, 1/2 + r_5 (1-r_6)\, 1/2\right) p_{j,T,A,j,t,B}{}^{(1)} + \left((1-r_3)(1-r_4)\, 1/2 + (1-r_3)\, r_4\, 1/2\right) p_{J,t,A,j,T,A}{}^{(1)} + (1-r_3)\, r_4\, 1/2\, p_{J,t,A,j,T,B}{}^{(1)} + (1-r_3)(1-r_4)\, 1/2\, p_{J,t,B,j,T,A}{}^{(1)} + \left(r_3 (1-r_4)\, 1/2 + r_3\, r_4\, 1/2\right) p_{J,T,A,j,t,A}{}^{(1)} + r_3 (1-r_4)\, 1/2\, p_{J,T,A,j,t,B}{}^{(1)} + r_3\, r_4\, 1/2\, p_{J,T,B,j,t,A}{}^{(1)} + 1/2\, p_{J,T,A,j,T,A}{}^{(1)} + \left(r_3 (1-r_4)\, 1/2 + (1-r_3)\, r_4\, 1/2\right) p_{J,T,A,j,T,B}{}^{(1)} + \left((1-r_3)(1-r_4)\, 1/2 + r_3\, r_4\, 1/2\right) p_{J,T,B,j,T,A}{}^{(1)}$$

$$p_{j,T,B}{}^{(2)} = p_{j,T,B,j,T,B}{}^{(1)} + 1/2\, p_{j,T,A,j,T,B}{}^{(1)} + \left((1-r_5)(1-r_6)\, 1/2 + r_5 (1-r_6)\, 1/2\right) p_{j,t,A,j,T,B}{}^{(1)} + \left((1-r_5)\, r_6\, 1/2 + r_5\, r_6\, 1/2\right) p_{j,T,A,j,t,B}{}^{(1)} + 1/2\, p_{j,t,B,j,T,B}{}^{(1)} + (1-r_3)(1-r_4)\, 1/2\, p_{J,t,A,j,T,B}{}^{(1)} + (1-r_3)\, r_4\, 1/2\, p_{J,t,B,j,T,A}{}^{(1)} +$$

$$\left((1-r_3)(1-r_4)\,1/2+(1-r_3)\,r_4\,1/2\right) p_{J,t,B,j,T,B}{}^{(1)}+r_3\,r_4\,1/2\,p_{J,T,A,j,t,B}{}^{(1)}+r_3\,(1-r_4)\,1/2\,p_{J,T,B,j,t,A}{}^{(1)}+\left(r_3\,(1-r_4)\,1/2+r_3\,r_4\,1/2\right) p_{J,T,B,j,t,B}{}^{(1)}+$$
$$\left((1-r_3)(1-r_4)\,1/2+r_3\,r_4\,1/2\right) p_{J,T,A,j,T,B}{}^{(1)}+\left(r_3\,(1-r_4)\,1/2+(1-r_3)\,r_4\,1/2\right) p_{J,T,B,j,T,A}{}^{(1)}+1/2\,p_{J,T,B,j,T,B}{}^{(1)}$$

Natural selection occurs on haploid organisms (3):

$$p_{J,t,A}{}^{(3)} = p_{J,t,A}{}^{(2)}\,w_1\left(\frac{1}{\overline{w}}\right)$$

$$p_{J,t,B}{}^{(3)} = p_{J,t,B}{}^{(2)}\,w_2\left(\frac{1}{\overline{w}}\right)$$

$$p_{J,T,A}{}^{(3)} = p_{J,T,A}{}^{(2)}\,w_3\left(\frac{1}{\overline{w}}\right)$$

$$p_{J,T,B}{}^{(3)} = p_{J,T,B}{}^{(2)}\,w_4\left(\frac{1}{\overline{w}}\right)$$

$$p_{j,t,A}{}^{(3)} = p_{j,t,A}{}^{(2)}\,w_5\left(\frac{1}{\overline{w}}\right)$$

$$p_{j,t,B}{}^{(3)} = p_{j,t,B}{}^{(2)}\,w_6\left(\frac{1}{\overline{w}}\right)$$

$$p_{j,T,A}{}^{(3)} = p_{j,T,A}{}^{(2)}\,w_7\left(\frac{1}{\overline{w}}\right)$$

$$p_{j,T,B}{}^{(3)} = p_{j,T,B}{}^{(2)}\,w_8\left(\frac{1}{\overline{w}}\right)$$

where $\overline{w} = p_{J,t,A}{}^{(2)}\,w_1+p_{J,t,B}{}^{(2)}\,w_2+p_{J,T,A}{}^{(2)}\,w_3+p_{J,T,B}{}^{(2)}\,w_4+p_{j,t,A}{}^{(2)}\,w_5+p_{j,t,B}{}^{(2)}\,w_6+p_{j,T,A}{}^{(2)}\,w_7+p_{j,T,B}{}^{(2)}\,w_8$

Transposition and deletion occurs as the final event (4):

$$p_{J,t,A}{}^{(4)} = (1-z)\,p_{J,t,A}{}^{(3)}+y p_{J,T,A}{}^{(3)}$$

$$p_{J,T,A}{}^{(4)} = (1-y)\,p_{J,T,A}{}^{(3)}+z p_{J,t,A}{}^{(3)}$$

$$p_{J,t,B}{}^{(4)} = (1-z)\,p_{J,t,B}{}^{(3)}+y p_{J,T,B}{}^{(3)}$$

$$p_{J,T,B}{}^{(4)} = (1-y)\,p_{J,T,B}{}^{(3)}+z p_{J,t,B}{}^{(3)}$$

$$p_{j,t,A}{}^{(4)} = (1-z)\,p_{j,t,A}{}^{(3)}+y p_{j,T,A}{}^{(3)}$$

$$p_{j,T,A}{}^{(4)} = (1-y)\,p_{j,T,A}{}^{(3)}+z p_{j,t,A}{}^{(3)}$$

$$p_{j,t,B}{}^{(4)} = (1-z)\, p_{j,t,B}{}^{(3)} + y p_{j,T,B}{}^{(3)}$$

$$p_{j,T,B}{}^{(4)} = (1-y)\, p_{j,T,B}{}^{(3)} + z p_{j,t,B}{}^{(3)}$$

After substituting each event into the subsequent event , the final recursion equations for the haplotype frequencies (used for simulations) are

$$
\begin{aligned}
pJlA[t+1] = \Big(&-\big((-1+S)\,(-1+z)\,(-1+\alpha 1)\; pJlA[t]^2\big) + S\, y\, pJLA[t] - S\, y\, \alpha 2\, pJLA[t] + y\, pJLA[t]^2 - S\, y\, pJLA[t]^2 - y\, \alpha 2\, pJLA[t]^2 + S\, y\, \alpha 2\, pJLA[t]^2 + \\
&r2\, pJLA[t] \times pJlB[t] - r2\, S\, pJLA[t] \times pJlB[t] + y\, pJLA[t] \times pJlB[t] - r2\, y\, pJLA[t] \times pJlB[t] - S\, y\, pJLA[t] \times pJlB[t] + \\
&r2\, S\, y\, pJLA[t] \times pJlB[t] - r2\, z\, pJLA[t] \times pJlB[t] + r2\, S\, z\, pJLA[t] \times pJlB[t] - r2\, \alpha 2\, pJLA[t] \times pJlB[t] + r2\, S\, \alpha 2\, pJLA[t] \times pJlB[t] - \\
&y\, \alpha 2\, pJLA[t] \times pJlB[t] + r2\, y\, \alpha 2\, pJLA[t] \times pJlB[t] + S\, y\, \alpha 2\, pJLA[t] \times pJlB[t] - r2\, S\, y\, \alpha 2\, pJLA[t] \times pJlB[t] + \\
&r2\, z\, \alpha 2\, pJLA[t] \times pJlB[t] - r2\, S\, z\, \alpha 2\, pJLA[t] \times pJlB[t] + S\, y\, \beta 2\, pJLB[t] + y\, pJLA[t] \times pJLB[t] - S\, y\, pJLA[t] \times pJLB[t] - \\
&y\, \alpha 2\, pJLA[t] \times pJLB[t] + S\, y\, \alpha 2\, pJLA[t] \times pJLB[t] + y\, \beta 2\, pJLA[t] \times pJLB[t] - S\, y\, \beta 2\, pJLA[t] \times pJLB[t] + r2\, \beta 2\, pJlB[t] \times pJLB[t] - \\
&r2\, S\, \beta 2\, pJlB[t] \times pJLB[t] + y\, \beta 2\, pJlB[t] \times pJLB[t] - r2\, y\, \beta 2\, pJlB[t] \times pJLB[t] - S\, y\, \beta 2\, pJlB[t] \times pJLB[t] + \\
&r2\, S\, y\, \beta 2\, pJlB[t] \times pJLB[t] - r2\, z\, \beta 2\, pJlB[t] \times pJLB[t] + r2\, S\, z\, \beta 2\, pJlB[t] \times pJLB[t] + y\, \beta 2\, pJLB[t]^2 - S\, y\, \beta 2\, pJLB[t]^2 + \\
&r3\, pJLA[t] \times pQlA[t] - r3\, S\, pJLA[t] \times pQlA[t] + y\, pJLA[t] \times pQlA[t] - r3\, y\, pJLA[t] \times pQlA[t] - S\, y\, pJLA[t] \times pQlA[t] + \\
&r3\, S\, y\, pJLA[t] \times pQlA[t] - r3\, z\, pJLA[t] \times pQlA[t] + r3\, S\, z\, pJLA[t] \times pQlA[t] - r3\, \alpha 1\, pJLA[t] \times pQlA[t] + \\
&r3\, r4\, \alpha 1\, pJLA[t] \times pQlA[t] + r3\, S\, \alpha 1\, pJLA[t] \times pQlA[t] - r3\, r4\, S\, \alpha 1\, pJLA[t] \times pQlA[t] - r4\, y\, \alpha 1\, pJLA[t] \times pQlA[t] + \\
&r3\, r4\, y\, \alpha 1\, pJLA[t] \times pQlA[t] + r4\, S\, y\, \alpha 1\, pJLA[t] \times pQlA[t] - r3\, r4\, S\, y\, \alpha 1\, pJLA[t] \times pQlA[t] + r3\, z\, \alpha 1\, pJLA[t] \times pQlA[t] - \\
&r3\, r4\, z\, \alpha 1\, pJLA[t] \times pQlA[t] - r3\, S\, z\, \alpha 1\, pJLA[t] \times pQlA[t] + r3\, r4\, S\, z\, \alpha 1\, pJLA[t] \times pQlA[t] - r3\, r4\, \alpha 2\, pJLA[t] \times pQlA[t] + \\
&r3\, r4\, S\, \alpha 2\, pJLA[t] \times pQlA[t] - y\, \alpha 2\, pJLA[t] \times pQlA[t] + r3\, y\, \alpha 2\, pJLA[t] \times pQlA[t] + r4\, y\, \alpha 2\, pJLA[t] \times pQlA[t] - \\
&r3\, r4\, y\, \alpha 2\, pJLA[t] \times pQlA[t] + S\, y\, \alpha 2\, pJLA[t] \times pQlA[t] - r3\, S\, y\, \alpha 2\, pJLA[t] \times pQlA[t] - r4\, S\, y\, \alpha 2\, pJLA[t] \times pQlA[t] + \\
&r3\, r4\, S\, y\, \alpha 2\, pJLA[t] \times pQlA[t] + r3\, r4\, z\, \alpha 2\, pJLA[t] \times pQlA[t] - r3\, r4\, S\, z\, \alpha 2\, pJLA[t] \times pQlA[t] + r3\, pJlB[t] \times pQlA[t] + \\
&r4\, pJlB[t] \times pQlA[t] - 2\, r3\, r4\, pJlB[t] \times pQlA[t] - r3\, S\, pJlB[t] \times pQlA[t] - r4\, S\, pJlB[t] \times pQlA[t] + 2\, r3\, r4\, S\, pJlB[t] \times pQlA[t] - \\
&r3\, z\, pJlB[t] \times pQlA[t] - r4\, z\, pJlB[t] \times pQlA[t] + 2\, r3\, r4\, z\, pJlB[t] \times pQlA[t] + r3\, S\, z\, pJlB[t] \times pQlA[t] + \\
&r4\, S\, z\, pJlB[t] \times pQlA[t] - 2\, r3\, r4\, S\, z\, pJlB[t] \times pQlA[t] - r3\, \alpha 1\, pJlB[t] \times pQlA[t] - r4\, \alpha 1\, pJlB[t] \times pQlA[t] + \\
&2\, r3\, r4\, \alpha 1\, pJlB[t] \times pQlA[t] + r3\, S\, \alpha 1\, pJlB[t] \times pQlA[t] + r4\, S\, \alpha 1\, pJlB[t] \times pQlA[t] - 2\, r3\, r4\, S\, \alpha 1\, pJlB[t] \times pQlA[t] + \\
&r3\, z\, \alpha 1\, pJlB[t] \times pQlA[t] + r4\, z\, \alpha 1\, pJlB[t] \times pQlA[t] - 2\, r3\, r4\, z\, \alpha 1\, pJlB[t] \times pQlA[t] - r3\, S\, z\, \alpha 1\, pJlB[t] \times pQlA[t] - \\
&r4\, S\, z\, \alpha 1\, pJlB[t] \times pQlA[t] + 2\, r3\, r4\, S\, z\, \alpha 1\, pJlB[t] \times pQlA[t] + r3\, pJLB[t] \times pQlA[t] - r3\, r4\, pJLB[t] \times pQlA[t] - \\
&r3\, S\, pJLB[t] \times pQlA[t] + r3\, r4\, S\, pJLB[t] \times pQlA[t] + r4\, y\, pJLB[t] \times pQlA[t] - r3\, r4\, y\, pJLB[t] \times pQlA[t] - \\
&r4\, S\, y\, pJLB[t] \times pQlA[t] + r3\, r4\, S\, y\, pJLB[t] \times pQlA[t] - r3\, z\, pJLB[t] \times pQlA[t] + r3\, r4\, z\, pJLB[t] \times pQlA[t] + \\
&r3\, S\, z\, pJLB[t] \times pQlA[t] - r3\, r4\, S\, z\, pJLB[t] \times pQlA[t] - r3\, \alpha 1\, pJLB[t] \times pQlA[t] + r3\, r4\, \alpha 1\, pJLB[t] \times pQlA[t] + \\
&r3\, S\, \alpha 1\, pJLB[t] \times pQlA[t] - r3\, r4\, S\, \alpha 1\, pJLB[t] \times pQlA[t] - r4\, y\, \alpha 1\, pJLB[t] \times pQlA[t] + r3\, r4\, y\, \alpha 1\, pJLB[t] \times pQlA[t] + \\
&r4\, S\, y\, \alpha 1\, pJLB[t] \times pQlA[t] - r3\, r4\, S\, y\, \alpha 1\, pJLB[t] \times pQlA[t] + r3\, z\, \alpha 1\, pJLB[t] \times pQlA[t] - r3\, r4\, z\, \alpha 1\, pJLB[t] \times pQlA[t] - \\
&r3\, S\, z\, \alpha 1\, pJLB[t] \times pQlA[t] + r3\, r4\, S\, z\, \alpha 1\, pJLB[t] \times pQlA[t] + r3\, r4\, \beta 2\, pJLB[t] \times pQlA[t] - r3\, r4\, S\, \beta 2\, pJLB[t] \times pQlA[t] + \\
&y\, \beta 2\, pJLB[t] \times pQlA[t] - r3\, y\, \beta 2\, pJLB[t] \times pQlA[t] - r4\, y\, \beta 2\, pJLB[t] \times pQlA[t] + r3\, r4\, y\, \beta 2\, pJLB[t] \times pQlA[t] - \\
&S\, y\, \beta 2\, pJLB[t] \times pQlA[t] + r3\, S\, y\, \beta 2\, pJLB[t] \times pQlA[t] + r4\, S\, y\, \beta 2\, pJLB[t] \times pQlA[t] - r3\, r4\, S\, y\, \beta 2\, pJLB[t] \times pQlA[t] - \\
&r3\, r4\, z\, \beta 2\, pJLB[t] \times pQlA[t] + r3\, r4\, S\, z\, \beta 2\, pJLB[t] \times pQlA[t] + y\, pJLA[t] \times pQLA[t] - S\, y\, pJLA[t] \times pQLA[t] - \\
&y\, \alpha 2\, pJLA[t] \times pQLA[t] + S\, y\, \alpha 2\, pJLA[t] \times pQLA[t] + r4\, pJlB[t] \times pQLA[t] - r3\, r4\, pJlB[t] \times pQLA[t] - r4\, S\, pJlB[t] \times pQLA[t] + \\
&r3\, r4\, S\, pJlB[t] \times pQLA[t] + r3\, y\, pJlB[t] \times pQLA[t] - r3\, r4\, y\, pJlB[t] \times pQLA[t] - r3\, S\, y\, pJlB[t] \times pQLA[t] + \\
&r3\, r4\, S\, y\, pJlB[t] \times pQLA[t] - r4\, z\, pJlB[t] \times pQLA[t] + r3\, r4\, z\, pJlB[t] \times pQLA[t] + r4\, S\, z\, pJlB[t] \times pQLA[t] - \\
&r3\, r4\, S\, z\, pJlB[t] \times pQLA[t] - r4\, \alpha 2\, pJlB[t] \times pQLA[t] + r3\, r4\, \alpha 2\, pJlB[t] \times pQLA[t] + r4\, S\, \alpha 2\, pJlB[t] \times pQLA[t] - \\
&r3\, r4\, S\, \alpha 2\, pJlB[t] \times pQLA[t] - r3\, y\, \alpha 2\, pJlB[t] \times pQLA[t] + r3\, r4\, y\, \alpha 2\, pJlB[t] \times pQLA[t] + r3\, S\, y\, \alpha 2\, pJlB[t] \times pQLA[t] - \\
&r3\, r4\, S\, y\, \alpha 2\, pJlB[t] \times pQLA[t] + r4\, z\, \alpha 2\, pJlB[t] \times pQLA[t] - r3\, r4\, z\, \alpha 2\, pJlB[t] \times pQLA[t] - r4\, S\, z\, \alpha 2\, pJlB[t] \times pQLA[t] + \\
&r3\, r4\, S\, z\, \alpha 2\, pJlB[t] \times pQLA[t] + r3\, y\, pJLB[t] \times pQLA[t] + r4\, y\, pJLB[t] \times pQLA[t] - 2\, r3\, r4\, y\, pJLB[t] \times pQLA[t] -
\end{aligned}
$$

r3 S y pJLB[t] × pQLA[t] − r4 S y pJLB[t] × pQLA[t] + 2 r3 r4 S y pJLB[t] × pQLA[t] − r3 y α2 pJLB[t] × pQLA[t] −
r4 y α2 pJLB[t] × pQLA[t] + 2 r3 r4 y α2 pJLB[t] × pQLA[t] + r3 S y α2 pJLB[t] × pQLA[t] + r4 S y α2 pJLB[t] × pQLA[t] −
2 r3 r4 S y α2 pJLB[t] × pQLA[t] + y β2 pJLB[t] × pQLA[t] − r3 y β2 pJLB[t] × pQLA[t] − r4 y β2 pJLB[t] × pQLA[t] +
2 r3 r4 y β2 pJLB[t] × pQLA[t] − S y β2 pJLB[t] × pQLA[t] + r3 S y β2 pJLB[t] × pQLA[t] + r4 S y β2 pJLB[t] × pQLA[t] −
2 r3 r4 S y β2 pJLB[t] × pQLA[t] + r3 r4 pJLA[t] × pQlB[t] − r3 r4 S pJLA[t] × pQlB[t] + y pJLA[t] × pQlB[t] −
r3 y pJLA[t] × pQlB[t] − r4 y pJLA[t] × pQlB[t] + r3 r4 y pJLA[t] × pQlB[t] − S y pJLA[t] × pQlB[t] + r3 S y pJLA[t] × pQlB[t] +
r4 S y pJLA[t] × pQlB[t] − r3 r4 S y pJLA[t] × pQlB[t] − r3 r4 z pJLA[t] × pQlB[t] + r3 r4 S z pJLA[t] × pQlB[t] −
r3 r4 α2 pJLA[t] × pQlB[t] + r3 r4 S α2 pJLA[t] × pQlB[t] − y α2 pJLA[t] × pQlB[t] + r3 y α2 pJLA[t] × pQlB[t] +
r4 y α2 pJLA[t] × pQlB[t] − r3 r4 y α2 pJLA[t] × pQlB[t] + S y α2 pJLA[t] × pQlB[t] − r3 S y α2 pJLA[t] × pQlB[t] −
r4 S y α2 pJLA[t] × pQlB[t] + r3 r4 S y α2 pJLA[t] × pQlB[t] + r3 r4 z α2 pJLA[t] × pQlB[t] − r3 r4 S z α2 pJLA[t] × pQlB[t] +
r3 r4 β2 pJLB[t] × pQlB[t] − r3 r4 S β2 pJLB[t] × pQlB[t] + y β2 pJLB[t] × pQlB[t] − r3 y β2 pJLB[t] × pQlB[t] −
r4 y β2 pJLB[t] × pQlB[t] + r3 r4 y β2 pJLB[t] × pQlB[t] − S y β2 pJLB[t] × pQlB[t] + r3 S y β2 pJLB[t] × pQlB[t] +
r4 S y β2 pJLB[t] × pQlB[t] − r3 r4 S y β2 pJLB[t] × pQlB[t] − r3 r4 z β2 pJLB[t] × pQlB[t] + r3 r4 S z β2 pJLB[t] × pQlB[t] +
y pJLA[t] × pQLB[t] − r3 y pJLA[t] × pQLB[t] − r4 y pJLA[t] × pQLB[t] + 2 r3 r4 y pJLA[t] × pQLB[t] −
S y pJLA[t] × pQLB[t] + r3 S y pJLA[t] × pQLB[t] + r4 S y pJLA[t] × pQLB[t] − 2 r3 r4 S y pJLA[t] × pQLB[t] −
y α2 pJLA[t] × pQLB[t] + r3 y α2 pJLA[t] × pQLB[t] + r4 y α2 pJLA[t] × pQLB[t] − 2 r3 r4 y α2 pJLA[t] × pQLB[t] +
S y α2 pJLA[t] × pQLB[t] − r3 S y α2 pJLA[t] × pQLB[t] − r4 S y α2 pJLA[t] × pQLB[t] + 2 r3 r4 S y α2 pJLA[t] × pQLB[t] +
r3 y β2 pJLA[t] × pQLB[t] + r4 y β2 pJLA[t] × pQLB[t] − 2 r3 r4 y β2 pJLA[t] × pQLB[t] − r3 S y β2 pJLA[t] × pQLB[t] −
r4 S y β2 pJLA[t] × pQLB[t] + 2 r3 r4 S y β2 pJLA[t] × pQLB[t] + r4 β2 pJlB[t] × pQLB[t] − r3 r4 β2 pJlB[t] × pQLB[t] −
r4 S β2 pJlB[t] × pQLB[t] + r3 r4 S β2 pJlB[t] × pQLB[t] + r3 y β2 pJlB[t] × pQLB[t] − r3 r4 y β2 pJlB[t] × pQLB[t] −
r3 S y β2 pJlB[t] × pQLB[t] + r3 r4 S y β2 pJlB[t] × pQLB[t] − r4 z β2 pJlB[t] × pQLB[t] + r3 r4 z β2 pJlB[t] × pQLB[t] +
r4 S z β2 pJlB[t] × pQLB[t] − r3 r4 S z β2 pJlB[t] × pQLB[t] + y β2 pJLB[t] × pQLB[t] − S y β2 pJLB[t] × pQLB[t] +
β1 ((−1 + S) (−1 + z) pJlB[t]^2 + (−1 + S) (−r4 y + r3 (−1 + r4 (1 + y − z) + z)) (pJLA[t] + pJLB[t]) pQlB[t] + pJlB[t] (S − S z + pJLA[t] −
r2 pJLA[t] − S pJLA[t] + r2 S pJLA[t] + r2 y pJLA[t] − r2 S y pJLA[t] − z pJLA[t] + r2 z pJLA[t] + S z pJLA[t] −
r2 S z pJLA[t] + pJLB[t] − r2 pJLB[t] − S pJLB[t] + r2 S pJLB[t] + r2 y pJLB[t] − r2 S y pJLB[t] − z pJLB[t] +
r2 z pJLB[t] + S z pJLB[t] − r2 S z pJLB[t] + (1 − r4 + r3 (−1 + 2 r4)) (−1 + S) (−1 + z) pQlA[t] + pQLA[t] − r3 pQLA[t] −
r4 pQLA[t] + r3 r4 pQLA[t] − S pQLA[t] + r3 S pQLA[t] + r4 S pQLA[t] − r3 r4 S pQLA[t] + r3 r4 y pQLA[t] −
r3 r4 S y pQLA[t] − z pQLA[t] + r3 z pQLA[t] + r4 z pQLA[t] − r3 r4 z pQLA[t] + S z pQLA[t] − r3 S z pQLA[t] −
r4 S z pQLA[t] + r3 r4 S z pQLA[t] + (−1 + S) (−1 + z) pQlB[t] + pQLB[t] − r3 pQLB[t] − r4 pQLB[t] + r3 r4 pQLB[t] −
S pQLB[t] + r3 S pQLB[t] + r4 S pQLB[t] − r3 r4 S pQLB[t] + r3 r4 y pQLB[t] − r3 r4 S y pQLB[t] − z pQLB[t] +
r3 z pQLB[t] + r4 z pQLB[t] − r3 r4 z pQLB[t] + S z pQLB[t] − r3 S z pQLB[t] − r4 S z pQLB[t] + r3 r4 S z pQLB[t])) +
pJlA[t] (S − S z + pJLA[t] − S pJLA[t] + y pJLA[t] − S y pJLA[t] − z pJLA[t] + S z pJLA[t] − r2 α2 pJLA[t] +
r2 S α2 pJLA[t] − y α2 pJLA[t] + r2 y α2 pJLA[t] + S y α2 pJLA[t] − r2 S y α2 pJLA[t] + r2 z α2 pJLA[t] − r2 S z α2 pJLA[t] +
pJlB[t] − S pJlB[t] − z pJlB[t] + S z pJlB[t] + β1 pJlB[t] − S β1 pJlB[t] − z β1 pJlB[t] + S z β1 pJlB[t] + pJLB[t] −
r2 pJLB[t] − S pJLB[t] + r2 S pJLB[t] + r2 y pJLB[t] − r2 S y pJLB[t] − z pJLB[t] + r2 z pJLB[t] + S z pJLB[t] −
r2 S z pJLB[t] + r2 β2 pJLB[t] − r2 S β2 pJLB[t] + y β2 pJLB[t] − r2 y β2 pJLB[t] − S y β2 pJLB[t] + r2 S y β2 pJLB[t] −
r2 z β2 pJLB[t] + r2 S z β2 pJLB[t] + pQlA[t] − S pQlA[t] − z pQlA[t] + S z pQlA[t] + pQLA[t] − r3 pQLA[t] − S pQLA[t] +
r3 S pQLA[t] + r3 y pQLA[t] − r3 S y pQLA[t] − z pQLA[t] + r3 z pQLA[t] + S z pQLA[t] − r3 S z pQLA[t] − r4 α2 pQLA[t] +
r3 r4 α2 pQLA[t] + r4 S α2 pQLA[t] − r3 r4 S α2 pQLA[t] − r3 y α2 pQLA[t] + r3 r4 y α2 pQLA[t] + r3 S y α2 pQLA[t] −
r3 r4 S y α2 pQLA[t] + r4 z α2 pQLA[t] − r3 r4 z α2 pQLA[t] − r4 S z α2 pQLA[t] + r3 r4 S z α2 pQLA[t] + pQlB[t] −
r3 pQlB[t] − r4 pQlB[t] + 2 r3 r4 pQlB[t] − S pQlB[t] + r3 S pQlB[t] + r4 S pQlB[t] − 2 r3 r4 S pQlB[t] − z pQlB[t] +
r3 z pQlB[t] + r4 z pQlB[t] − 2 r3 r4 z pQlB[t] + S z pQlB[t] − r3 S z pQlB[t] − r4 S z pQlB[t] + 2 r3 r4 S z pQlB[t] +
r3 β1 pQlB[t] + r4 β1 pQlB[t] − 2 r3 r4 β1 pQlB[t] − r3 S β1 pQlB[t] − r4 S β1 pQlB[t] + 2 r3 r4 S β1 pQlB[t] −
r3 z β1 pQlB[t] − r4 z β1 pQlB[t] + 2 r3 r4 z β1 pQlB[t] + r3 S z β1 pQlB[t] + r4 S z β1 pQlB[t] − 2 r3 r4 S z β1 pQlB[t] +
pQLB[t] − r3 pQLB[t] − r4 pQLB[t] + r3 r4 pQLB[t] − S pQLB[t] + r3 S pQLB[t] + r4 S pQLB[t] − r3 r4 S pQLB[t] +

```
r3 r4 y pQLB[t] - r3 r4 S y pQLB[t] - z pQLB[t] + r3 z pQLB[t] + r4 z pQLB[t] - r3 r4 z pQLB[t] + S z pQLB[t] - r3 S z
  pQLB[t] - r4 S z pQLB[t] + r3 r4 S z pQLB[t] + r4 β2 pQLB[t] - r3 r4 β2 pQLB[t] - r4 S β2 pQLB[t] + r3 r4 S β2 pQLB[t] +
r3 y β2 pQLB[t] - r3 r4 y β2 pQLB[t] - r3 S y β2 pQLB[t] + r3 r4 S y β2 pQLB[t] - r4 z β2 pQLB[t] + r3 r4 z β2 pQLB[t] +
r4 S z β2 pQLB[t] - r3 r4 S z β2 pQLB[t] - α1 (S - S z + pJLA[t] - r2 pJLA[t] - S pJLA[t] + r2 S pJLA[t] + r2 y pJLA[t] -
    r2 S y pJLA[t] - z pJLA[t] + r2 z pJLA[t] + S z pJLA[t] - r2 S z pJLA[t] + pJlB[t] - S pJlB[t] - z pJlB[t] + S z pJlB[t] +
    pJLB[t] - r2 pJLB[t] - S pJLB[t] + r2 S pJLB[t] + r2 y pJLB[t] - r2 S y pJLB[t] - z pJLB[t] + r2 z pJLB[t] +
    S z pJLB[t] - r2 S z pJLB[t] + (-1 + S) (-1 + z) pQlA[t] + pQLA[t] - r3 pQLA[t] - r4 pQLA[t] + r3 r4 pQLA[t] -
    S pQLA[t] + r3 S pQLA[t] + r4 S pQLA[t] - r3 r4 S pQLA[t] + r3 r4 y pQLA[t] - r3 r4 S y pQLA[t] - z pQLA[t] +
    r3 z pQLA[t] + r4 z pQLA[t] - r3 r4 z pQLA[t] + S z pQLA[t] - r3 S z pQLA[t] - r4 S z pQLA[t] + r3 r4 S z pQLA[t] +
    (1 - r3 - r4 + 2 r3 r4) (-1 + S) (-1 + z) pQlB[t] + pQLB[t] - r3 pQLB[t] - r4 pQLB[t] + r3 r4 pQLB[t] - S pQLB[t] +
    r3 S pQLB[t] + r4 S pQLB[t] - r3 r4 S pQLB[t] + r3 r4 y pQLB[t] - r3 r4 S y pQLB[t] - z pQLB[t] + r3 z pQLB[t] +
    r4 z pQLB[t] - r3 r4 z pQLB[t] + S z pQLB[t] - r3 S z pQLB[t] - r4 S z pQLB[t] + r3 r4 S z pQLB[t]))) /
((S + pJlA[t] - S pJlA[t] + pJLA[t] - S pJLA[t] + pJlB[t] - S pJlB[t] + pJLB[t] - S pJLB[t] - (-1 + S) pQlA[t] +
    pQLA[t] - S pQLA[t] - (-1 + S) pQlB[t] + pQLB[t] - S pQLB[t])
  (pJlA[t] - k α1 pJlA[t] + pJLA[t] - k α2 pJLA[t] + pJlB[t] - k pJlB[t] + k β1 pJlB[t] + pJLB[t] - k pJLB[t] + k β2 pJLB[t] +
    (1 - k α1) pQlA[t] + pQLA[t] - k α2 pQLA[t] + (1 - k + k β1) pQlB[t] + pQLB[t] - k pQLB[t] + k β2 pQLB[t]));


pJlB[t + 1] =
  -(((-1 + k) (S y α2 pJLA[t] + r2 α2 pJlA[t] × pJLA[t] - r2 S α2 pJlA[t] × pJLA[t] + y α2 pJlA[t] × pJLA[t] - r2 y α2 pJlA[t] × pJLA[t] - S y
      α2 pJlA[t] × pJLA[t] + r2 S y α2 pJlA[t] × pJLA[t] - r2 z α2 pJlA[t] × pJLA[t] + r2 S z α2 pJlA[t] × pJLA[t] +
    y α2 pJLA[t]^2 - S y α2 pJLA[t]^2 + S pJlB[t] - S z pJlB[t] - S β1 pJlB[t] + S z β1 pJlB[t] + pJlA[t] × pJlB[t] -
    S pJlA[t] × pJlB[t] - z pJlA[t] × pJlB[t] + S z pJlA[t] × pJlB[t] - β1 pJlA[t] × pJlB[t] + S β1 pJlA[t] × pJlB[t] +
    z β1 pJlA[t] × pJlB[t] - S z β1 pJlA[t] × pJlB[t] + pJLA[t] × pJlB[t] - r2 pJLA[t] × pJlB[t] - S pJLA[t] × pJlB[t] +
    r2 S pJLA[t] × pJlB[t] + r2 y pJLA[t] × pJlB[t] - r2 S y pJLA[t] × pJlB[t] - z pJLA[t] × pJlB[t] +
    r2 z pJLA[t] × pJlB[t] + S z pJLA[t] × pJlB[t] - r2 S z pJLA[t] × pJlB[t] + r2 α2 pJLA[t] × pJlB[t] -
    r2 S α2 pJLA[t] × pJlB[t] + y α2 pJLA[t] × pJlB[t] - r2 y α2 pJLA[t] × pJlB[t] - S y α2 pJLA[t] × pJlB[t] +
    r2 S y α2 pJLA[t] × pJlB[t] - r2 z α2 pJLA[t] × pJlB[t] + r2 S z α2 pJLA[t] × pJlB[t] - β1 pJLA[t] × pJlB[t] +
    r2 β1 pJLA[t] × pJlB[t] + S β1 pJLA[t] × pJlB[t] - r2 S β1 pJLA[t] × pJlB[t] - r2 y β1 pJLA[t] × pJlB[t] +
    r2 S y β1 pJLA[t] × pJlB[t] + z β1 pJLA[t] × pJlB[t] - r2 z β1 pJLA[t] × pJlB[t] - S z β1 pJLA[t] × pJlB[t] +
    r2 S z β1 pJLA[t] × pJlB[t] + pJlB[t]^2 - S pJlB[t]^2 - z pJlB[t]^2 + S z pJlB[t]^2 - β1 pJlB[t]^2 + S β1 pJlB[t]^2 +
    z β1 pJlB[t]^2 - S z β1 pJlB[t]^2 + S y pJLB[t] - S y β2 pJLB[t] + r2 pJlA[t] × pJLB[t] - r2 S pJlA[t] × pJLB[t] +
    y pJlA[t] × pJLB[t] - r2 y pJlA[t] × pJLB[t] - S y pJlA[t] × pJLB[t] + r2 S y pJlA[t] × pJLB[t] - r2 z pJlA[t] × pJLB[t] +
    r2 S z pJlA[t] × pJLB[t] - r2 β2 pJlA[t] × pJLB[t] + r2 S β2 pJlA[t] × pJLB[t] - y β2 pJlA[t] × pJLB[t] +
    r2 y β2 pJlA[t] × pJLB[t] + S y β2 pJlA[t] × pJLB[t] - r2 S y β2 pJlA[t] × pJLB[t] + r2 z β2 pJlA[t] × pJLB[t] -
    r2 S z β2 pJlA[t] × pJLB[t] + y pJLA[t] × pJLB[t] - S y pJLA[t] × pJLB[t] + y α2 pJLA[t] × pJLB[t] -
    S y α2 pJLA[t] × pJLB[t] - y β2 pJLA[t] × pJLB[t] + S y β2 pJLA[t] × pJLB[t] + pJlB[t] × pJLB[t] - S pJlB[t] × pJLB[t] +
    y pJlB[t] × pJLB[t] - S y pJlB[t] × pJLB[t] - z pJlB[t] × pJLB[t] + S z pJlB[t] × pJLB[t] - β1 pJlB[t] × pJLB[t] +
    r2 β1 pJlB[t] × pJLB[t] + S β1 pJlB[t] × pJLB[t] - r2 S β1 pJlB[t] × pJLB[t] - r2 y β1 pJlB[t] × pJLB[t] +
    r2 S y β1 pJlB[t] × pJLB[t] + z β1 pJlB[t] × pJLB[t] - r2 z β1 pJlB[t] × pJLB[t] - S z β1 pJlB[t] × pJLB[t] +
    r2 S z β1 pJlB[t] × pJLB[t] - r2 β2 pJlB[t] × pJLB[t] + r2 S β2 pJlB[t] × pJLB[t] - y β2 pJlB[t] × pJLB[t] +
    r2 y β2 pJlB[t] × pJLB[t] + S y β2 pJlB[t] × pJLB[t] - r2 S y β2 pJlB[t] × pJLB[t] + r2 z β2 pJlB[t] × pJLB[t] -
    r2 S z β2 pJlB[t] × pJLB[t] + y pJLB[t]^2 - S y pJLB[t]^2 - y β2 pJLB[t]^2 + S y β2 pJLB[t]^2 + r3 r4 α2 pJLA[t] × pQlA[t] -
    r3 r4 S α2 pJLA[t] × pQlA[t] + y α2 pJLA[t] × pQlA[t] - r3 y α2 pJLA[t] × pQlA[t] - r4 y α2 pJLA[t] × pQlA[t] +
```

r3 r4 y α2 pJLA[t] × pQlA[t] – S y α2 pJLA[t] × pQlA[t] + r3 S y α2 pJLA[t] × pQlA[t] + r4 S y α2 pJLA[t] × pQlA[t] –
r3 r4 S y α2 pJLA[t] × pQlA[t] – r3 r4 z α2 pJLA[t] × pQlA[t] + r3 r4 S z α2 pJLA[t] × pQlA[t] + pJlB[t] × pQlA[t] –
r3 pJlB[t] × pQlA[t] – r4 pJlB[t] × pQlA[t] + 2 r3 r4 pJlB[t] × pQlA[t] – S pJlB[t] × pQlA[t] + r3 S pJlB[t] × pQlA[t] +
r4 S pJlB[t] × pQlA[t] – 2 r3 r4 S pJlB[t] × pQlA[t] – z pJlB[t] × pQlA[t] + r3 z pJlB[t] × pQlA[t] +
r4 z pJlB[t] × pQlA[t] – 2 r3 r4 z pJlB[t] × pQlA[t] + S z pJlB[t] × pQlA[t] – r3 S z pJlB[t] × pQlA[t] –
r4 S z pJlB[t] × pQlA[t] + 2 r3 r4 S z pJlB[t] × pQlA[t] – β1 pJlB[t] × pQlA[t] + r3 β1 pJlB[t] × pQlA[t] +
r4 β1 pJlB[t] × pQlA[t] – 2 r3 r4 β1 pJlB[t] × pQlA[t] + S β1 pJlB[t] × pQlA[t] – r3 S β1 pJlB[t] × pQlA[t] –
r4 S β1 pJlB[t] × pQlA[t] + 2 r3 r4 S β1 pJlB[t] × pQlA[t] + z β1 pJlB[t] × pQlA[t] – r3 z β1 pJlB[t] × pQlA[t] –
r4 z β1 pJlB[t] × pQlA[t] + 2 r3 r4 z β1 pJlB[t] × pQlA[t] – S z β1 pJlB[t] × pQlA[t] + r3 S z β1 pJlB[t] × pQlA[t] +
r4 S z β1 pJlB[t] × pQlA[t] – 2 r3 r4 S z β1 pJlB[t] × pQlA[t] + r3 r4 pJLB[t] × pQlA[t] – r3 r4 S pJLB[t] × pQlA[t] +
y pJLB[t] × pQlA[t] – r3 y pJLB[t] × pQlA[t] – r4 y pJLB[t] × pQlA[t] + r3 r4 y pJLB[t] × pQlA[t] –
S y pJLB[t] × pQlA[t] + r3 S y pJLB[t] × pQlA[t] + r4 S y pJLB[t] × pQlA[t] – r3 r4 S y pJLB[t] × pQlA[t] –
r3 r4 z pJLB[t] × pQlA[t] + r3 r4 S z pJLB[t] × pQlA[t] – r3 r4 β2 pJLB[t] × pQlA[t] + r3 r4 S β2 pJLB[t] × pQlA[t] –
y β2 pJLB[t] × pQlA[t] + r3 y β2 pJLB[t] × pQlA[t] + r4 y β2 pJLB[t] × pQlA[t] – r3 r4 y β2 pJLB[t] × pQlA[t] +
S y β2 pJLB[t] × pQlA[t] – r3 S y β2 pJLB[t] × pQlA[t] – r4 S y β2 pJLB[t] × pQlA[t] + r3 r4 S y β2 pJLB[t] × pQlA[t] +
r3 r4 z β2 pJLB[t] × pQlA[t] – r3 r4 S z β2 pJLB[t] × pQlA[t] + r4 α2 pJlA[t] × pQLA[t] – r3 r4 α2 pJlA[t] × pQLA[t] –
r4 S α2 pJlA[t] × pQLA[t] + r3 r4 S α2 pJlA[t] × pQLA[t] + r3 y α2 pJlA[t] × pQLA[t] – r3 r4 y α2 pJlA[t] × pQLA[t] –
r3 S y α2 pJlA[t] × pQLA[t] + r3 r4 S y α2 pJlA[t] × pQLA[t] – r4 z α2 pJlA[t] × pQLA[t] + r3 r4 z α2 pJlA[t] × pQLA[t] +
r4 S z α2 pJlA[t] × pQLA[t] – r3 r4 S z α2 pJlA[t] × pQLA[t] + y α2 pJLA[t] × pQLA[t] – S y α2 pJLA[t] × pQLA[t] +
pJlB[t] × pQLA[t] – r3 pJlB[t] × pQLA[t] – r4 pJlB[t] × pQLA[t] + r3 r4 pJlB[t] × pQLA[t] – S pJlB[t] × pQLA[t] +
r3 S pJlB[t] × pQLA[t] + r4 S pJlB[t] × pQLA[t] – r3 r4 S pJlB[t] × pQLA[t] + r3 r4 y pJlB[t] × pQLA[t] –
r3 r4 S y pJlB[t] × pQLA[t] – z pJlB[t] × pQLA[t] + r3 z pJlB[t] × pQLA[t] + r4 z pJlB[t] × pQLA[t] –
r3 r4 z pJlB[t] × pQLA[t] + S z pJlB[t] × pQLA[t] – r3 S z pJlB[t] × pQLA[t] – r4 S z pJlB[t] × pQLA[t] +
r3 r4 S z pJlB[t] × pQLA[t] + r4 α2 pJlB[t] × pQLA[t] – r3 r4 α2 pJlB[t] × pQLA[t] – r4 S α2 pJlB[t] × pQLA[t] +
r3 r4 S α2 pJlB[t] × pQLA[t] + r3 y α2 pJlB[t] × pQLA[t] – r3 r4 y α2 pJlB[t] × pQLA[t] – r3 S y α2 pJlB[t] × pQLA[t] +
r3 r4 S y α2 pJlB[t] × pQLA[t] – r4 z α2 pJlB[t] × pQLA[t] + r3 r4 z α2 pJlB[t] × pQLA[t] + r4 S z α2 pJlB[t] × pQLA[t] –
r3 r4 S z α2 pJlB[t] × pQLA[t] – β1 pJlB[t] × pQLA[t] + r3 β1 pJlB[t] × pQLA[t] + r4 β1 pJlB[t] × pQLA[t] –
r3 r4 β1 pJlB[t] × pQLA[t] + S β1 pJlB[t] × pQLA[t] – r3 S β1 pJlB[t] × pQLA[t] – r4 S β1 pJlB[t] × pQLA[t] +
r3 r4 S β1 pJlB[t] × pQLA[t] – r3 r4 y β1 pJlB[t] × pQLA[t] + r3 r4 S y β1 pJlB[t] × pQLA[t] + z β1 pJlB[t] × pQLA[t] –
r3 z β1 pJlB[t] × pQLA[t] – r4 z β1 pJlB[t] × pQLA[t] + r3 r4 z β1 pJlB[t] × pQLA[t] – S z β1 pJlB[t] × pQLA[t] +
r3 S z β1 pJlB[t] × pQLA[t] + r4 S z β1 pJlB[t] × pQLA[t] – r3 r4 S z β1 pJlB[t] × pQLA[t] + y pJLB[t] × pQLA[t] –
r3 y pJLB[t] × pQLA[t] – r4 y pJLB[t] × pQLA[t] + 2 r3 r4 y pJLB[t] × pQLA[t] – S y pJLB[t] × pQLA[t] +
r3 S y pJLB[t] × pQLA[t] + r4 S y pJLB[t] × pQLA[t] – 2 r3 r4 S y pJLB[t] × pQLA[t] + r3 y α2 pJLB[t] × pQLA[t] +
r4 y α2 pJLB[t] × pQLA[t] – 2 r3 r4 y α2 pJLB[t] × pQLA[t] – r3 S y α2 pJLB[t] × pQLA[t] – r4 S y α2 pJLB[t] × pQLA[t] +
2 r3 r4 S y α2 pJLB[t] × pQLA[t] – y β2 pJLB[t] × pQLA[t] + r3 y β2 pJLB[t] × pQLA[t] + r4 y β2 pJLB[t] × pQLA[t] –
2 r3 r4 y β2 pJLB[t] × pQLA[t] + S y β2 pJLB[t] × pQLA[t] – r3 S y β2 pJLB[t] × pQLA[t] – r4 S y β2 pJLB[t] × pQLA[t] +
2 r3 r4 S y β2 pJLB[t] × pQLA[t] + (–1 + S) ((–r4 + r3 (–1 + 2 r4)) (–1 + z) (–1 + β1) pJlA[t] – r3 pJLA[t] + r3 r4 pJLA[t] –
r4 y pJLA[t] + r3 r4 y pJLA[t] + r3 z pJLA[t] – r3 r4 z pJLA[t] – r3 r4 α2 pJLA[t] – y α2 pJLA[t] +
r3 y α2 pJLA[t] + r4 y α2 pJLA[t] – r3 r4 y α2 pJLA[t] + r3 r4 z α2 pJLA[t] + r3 β1 pJLA[t] – r3 r4 β1 pJLA[t] +
r4 y β1 pJLA[t] – r3 r4 y β1 pJLA[t] – r3 z β1 pJLA[t] + r3 r4 z β1 pJLA[t] – (–1 + z) (–1 + β1) pJlB[t] –
r3 pJLB[t] – y pJLB[t] + r3 y pJLB[t] + r3 z pJLB[t] + r3 β1 pJLB[t] – r3 r4 β1 pJLB[t] +
r4 y β1 pJLB[t] – r3 r4 y β1 pJLB[t] – r3 z β1 pJLB[t] + r3 r4 z β1 pJLB[t] + r3 r4 β2 pJLB[t] +
y β2 pJLB[t] – r3 y β2 pJLB[t] – r4 y β2 pJLB[t] + r3 r4 y β2 pJLB[t] – r3 r4 z β2 pJLB[t]) pQlB[t] +
r4 pJlA[t] × pQLB[t] – r3 r4 pJlA[t] × pQLB[t] – r4 S pJlA[t] × pQLB[t] + r3 r4 S pJlA[t] × pQLB[t] +
r3 y pJlA[t] × pQLB[t] – r3 r4 y pJlA[t] × pQLB[t] – r3 S y pJlA[t] × pQLB[t] + r3 r4 S y pJlA[t] × pQLB[t] –

r4 z pJlA[t] × pQLB[t] + r3 r4 z pJlA[t] × pQLB[t] + r4 S z pJlA[t] × pQLB[t] − r3 r4 S z pJlA[t] × pQLB[t] −
r4 β2 pJlA[t] × pQLB[t] + r3 r4 β2 pJlA[t] × pQLB[t] + r4 S β2 pJlA[t] × pQLB[t] − r3 r4 S β2 pJlA[t] × pQLB[t] −
r3 y β2 pJlA[t] × pQLB[t] + r3 r4 y β2 pJlA[t] × pQLB[t] + r3 S y β2 pJlA[t] × pQLB[t] − r3 r4 S y β2 pJlA[t] × pQLB[t] +
r4 z β2 pJlA[t] × pQLB[t] − r3 r4 z β2 pJlA[t] × pQLB[t] − r4 S z β2 pJlA[t] × pQLB[t] + r3 r4 S z β2 pJlA[t] × pQLB[t] +
r3 y pJLA[t] × pQLB[t] + r4 y pJLA[t] × pQLB[t] − 2 r3 r4 y pJLA[t] × pQLB[t] − r3 S y pJLA[t] × pQLB[t] −
r4 S y pJLA[t] × pQLB[t] + 2 r3 r4 S y pJLA[t] × pQLB[t] + y α2 pJLA[t] × pQLB[t] − r3 y α2 pJLA[t] × pQLB[t] −
r4 y α2 pJLA[t] × pQLB[t] + 2 r3 r4 y α2 pJLA[t] × pQLB[t] − S y α2 pJLA[t] × pQLB[t] + r3 S y α2 pJLA[t] × pQLB[t] +
r4 S y α2 pJLA[t] × pQLB[t] − 2 r3 r4 S y α2 pJLA[t] × pQLB[t] − r3 y β2 pJLA[t] × pQLB[t] −
r4 y β2 pJLA[t] × pQLB[t] + 2 r3 r4 y β2 pJLA[t] × pQLB[t] + r3 S y β2 pJLA[t] × pQLB[t] + r4 S y β2 pJLA[t] × pQLB[t] −
2 r3 r4 S y β2 pJLA[t] × pQLB[t] + pJlB[t] × pQLB[t] − r3 pJlB[t] × pQLB[t] − S pJlB[t] × pQLB[t] +
r3 S pJlB[t] × pQLB[t] + r3 y pJlB[t] × pQLB[t] − r3 S y pJlB[t] × pQLB[t] − z pJlB[t] × pQLB[t] + r3 z pJlB[t] × pQLB[t] +
S z pJlB[t] × pQLB[t] − r3 S z pJlB[t] × pQLB[t] − β1 pJlB[t] × pQLB[t] + r3 β1 pJlB[t] × pQLB[t] +
r4 β1 pJlB[t] × pQLB[t] − r3 r4 β1 pJlB[t] × pQLB[t] + S β1 pJlB[t] × pQLB[t] − r3 S β1 pJlB[t] × pQLB[t] −
r4 S β1 pJlB[t] × pQLB[t] + r3 r4 S β1 pJlB[t] × pQLB[t] − r3 r4 y β1 pJlB[t] × pQLB[t] + r3 r4 S y β1 pJlB[t] × pQLB[t] +
z β1 pJlB[t] × pQLB[t] − r3 z β1 pJlB[t] × pQLB[t] − r4 z β1 pJlB[t] × pQLB[t] + r3 r4 z β1 pJlB[t] × pQLB[t] −
S z β1 pJlB[t] × pQLB[t] + r3 S z β1 pJlB[t] × pQLB[t] + r4 S z β1 pJlB[t] × pQLB[t] − r3 r4 S z β1 pJlB[t] × pQLB[t] −
r4 β2 pJlB[t] × pQLB[t] + r3 r4 β2 pJlB[t] × pQLB[t] + r4 S β2 pJlB[t] × pQLB[t] − r3 r4 S β2 pJlB[t] × pQLB[t] −
r3 y β2 pJlB[t] × pQLB[t] + r3 r4 y β2 pJlB[t] × pQLB[t] + r3 S y β2 pJlB[t] × pQLB[t] − r3 r4 S y β2 pJlB[t] × pQLB[t] +
r4 z β2 pJlB[t] × pQLB[t] − r3 r4 z β2 pJlB[t] × pQLB[t] − r4 S z β2 pJlB[t] × pQLB[t] + r3 r4 S z β2 pJlB[t] × pQLB[t] +
y pJLB[t] × pQLB[t] − S y pJLB[t] × pQLB[t] − y β2 pJLB[t] × pQLB[t] + S y β2 pJLB[t] × pQLB[t] +
α1 ((−1 + S) (−1 + z) pJlA[t]^2 − (−1 + S) ((−r4 + r3 (−1 + 2 r4)) (−1 + z) pJlB[t] − (−r4 y + r3 (−1 + r4 (1 + y − z) + z))
(pJLA[t] + pJLB[t])) pQlA[t] + pJlA[t] (S − S z + pJLA[t] − r2 pJLA[t] − S pJLA[t] + r2 S pJLA[t] + r2
y pJLA[t] − r2 S y pJLA[t] − z pJLA[t] + r2 z pJLA[t] + S z pJLA[t] − r2 S z pJLA[t] + pJlB[t] − S
pJlB[t] − z pJlB[t] + S z pJlB[t] + pJLB[t] − r2 pJLB[t] − S pJLB[t] + r2 S pJLB[t] + r2 y pJLB[t] − r2 S y
pJLB[t] − z pJLB[t] + r2 z pJLB[t] + S z pJLB[t] − r2 S z pJLB[t] + (−1 + S) (−1 + z) pQlA[t] + pQLA[t] − r3
pQLA[t] − r4 pQLA[t] + r3 r4 pQLA[t] − S pQLA[t] + r3 S pQLA[t] + r4 S pQLA[t] − r3 r4 S pQLA[t] + r3
r4 y pQLA[t] − r3 r4 S y pQLA[t] − z pQLA[t] + r3 z pQLA[t] + r4 z pQLA[t] − r3 r4 z pQLA[t] + S z
pQLA[t] − r3 S z pQLA[t] − r4 S z pQLA[t] + r3 r4 S z pQLA[t] + (1 − r3 − r4 + 2 r3 r4) (−1 + S) (−1 + z)
pQlB[t] + pQLB[t] − r3 pQLB[t] − r4 pQLB[t] + r3 r4 pQLB[t] − S pQLB[t] + r3 S pQLB[t] + r4 S
pQLB[t] − r3 r4 S pQLB[t] + r3 r4 y pQLB[t] − r3 r4 S y pQLB[t] − z pQLB[t] + r3 z pQLB[t] + r4 z
pQLB[t] − r3 r4 z pQLB[t] + S z pQLB[t] − r3 S z pQLB[t] − r4 S z pQLB[t] + r3 r4 S z pQLB[t])))) /
((S + pJlA[t] − S pJlA[t] + pJLA[t] − S pJLA[t] + pJlB[t] − S pJlB[t] + pJLB[t] − S pJLB[t] − (−1 + S) pQlA[t] +
pQLA[t] − S pQLA[t] − (−1 + S) pQlB[t] + pQLB[t] − S pQLB[t])
(pJlA[t] − k α1 pJlA[t] + pJLA[t] − k α2 pJLA[t] + pJlB[t] − k pJlB[t] + k β1 pJlB[t] + pJLB[t] − k pJLB[t] + k β2 pJLB[t] +
(1 − k α1) pQlA[t] + pQLA[t] − k α2 pQLA[t] + (1 − k + k β1) pQlB[t] + pQLB[t] − k pQLB[t] + k β2 pQLB[t])));

pJLA[t + 1] =
((−1 + S) z (−1 + α1) pJlA[t]^2 + S pJLA[t] − S y pJLA[t] − S α2 pJLA[t] + S y α2 pJLA[t] + pJLA[t]^2 − S pJLA[t]^2 − y pJLA[t]^2 + S y pJLA[t]^2 −
α2 pJLA[t]^2 + S α2 pJLA[t]^2 + y α2 pJLA[t]^2 − S y α2 pJLA[t]^2 + pJLA[t] × pJlB[t] − r2 pJLA[t] × pJlB[t] − S pJLA[t] × pJlB[t] +
r2 S pJLA[t] × pJlB[t] − y pJLA[t] × pJlB[t] + r2 y pJLA[t] × pJlB[t] + S y pJLA[t] × pJlB[t] − r2 S y pJLA[t] × pJlB[t] +
r2 z pJLA[t] × pJlB[t] − r2 S z pJLA[t] × pJlB[t] − α2 pJLA[t] × pJlB[t] + r2 α2 pJLA[t] × pJlB[t] + S α2 pJLA[t] × pJlB[t] −
r2 S α2 pJLA[t] × pJlB[t] + y α2 pJLA[t] × pJlB[t] − r2 y α2 pJLA[t] × pJlB[t] − S y α2 pJLA[t] × pJlB[t] +
r2 S y α2 pJLA[t] × pJlB[t] − r2 z α2 pJLA[t] × pJlB[t] + r2 S z α2 pJLA[t] × pJlB[t] + S β2 pJLB[t] − S y β2 pJLB[t] +

pJLA[t] × pJLB[t] – S pJLA[t] × pJLB[t] – y pJLA[t] × pJLB[t] + S y pJLA[t] × pJLB[t] – α2 pJLA[t] × pJLB[t] +
S α2 pJLA[t] × pJLB[t] + y α2 pJLA[t] × pJLB[t] – S y α2 pJLA[t] × pJLB[t] + β2 pJLA[t] × pJLB[t] – S β2 pJLA[t] × pJLB[t] –
y β2 pJLA[t] × pJLB[t] + S y β2 pJLA[t] × pJLB[t] + β2 pJlB[t] × pJLB[t] – r2 β2 pJlB[t] × pJLB[t] – S β2 pJlB[t] × pJLB[t] +
r2 S β2 pJlB[t] × pJLB[t] – y β2 pJlB[t] × pJLB[t] + r2 y β2 pJlB[t] × pJLB[t] + S y β2 pJlB[t] × pJLB[t] –
r2 S y β2 pJlB[t] × pJLB[t] + r2 z β2 pJlB[t] × pJLB[t] – r2 S z β2 pJlB[t] × pJLB[t] + β2 pJLB[t]^2 – S β2 pJLB[t]^2 –
y β2 pJLB[t]^2 + S y β2 pJLB[t]^2 + pJLA[t] × pQlA[t] – r3 pJLA[t] × pQlA[t] – S pJLA[t] × pQlA[t] + r3 S pJLA[t] × pQlA[t] –
y pJLA[t] × pQlA[t] + r3 y pJLA[t] × pQlA[t] + S y pJLA[t] × pQlA[t] – r3 S y pJLA[t] × pQlA[t] + r3 z pJLA[t] × pQlA[t] –
r3 S z pJLA[t] × pQlA[t] – r4 α1 pJLA[t] × pQlA[t] + r3 r4 α1 pJLA[t] × pQlA[t] + r4 S α1 pJLA[t] × pQlA[t] –
r3 r4 S α1 pJLA[t] × pQlA[t] + r4 y α1 pJLA[t] × pQlA[t] – r3 r4 y α1 pJLA[t] × pQlA[t] – r4 S y α1 pJLA[t] × pQlA[t] +
r3 r4 S y α1 pJLA[t] × pQlA[t] – r3 z α1 pJLA[t] × pQlA[t] + r3 r4 z α1 pJLA[t] × pQlA[t] + r3 S z α1 pJLA[t] × pQlA[t] –
r3 r4 S z α1 pJLA[t] × pQlA[t] – α2 pJLA[t] × pQlA[t] + r3 α2 pJLA[t] × pQlA[t] + r4 α2 pJLA[t] × pQlA[t] –
r3 r4 α2 pJLA[t] × pQlA[t] + S α2 pJLA[t] × pQlA[t] – r3 S α2 pJLA[t] × pQlA[t] – r4 S α2 pJLA[t] × pQlA[t] +
r3 r4 S α2 pJLA[t] × pQlA[t] + y α2 pJLA[t] × pQlA[t] – r3 y α2 pJLA[t] × pQlA[t] – r4 y α2 pJLA[t] × pQlA[t] +
r3 r4 y α2 pJLA[t] × pQlA[t] – S y α2 pJLA[t] × pQlA[t] + r3 S y α2 pJLA[t] × pQlA[t] + r4 S y α2 pJLA[t] × pQlA[t] –
r3 r4 S y α2 pJLA[t] × pQlA[t] – r3 r4 z α2 pJLA[t] × pQlA[t] + r3 r4 S z α2 pJLA[t] × pQlA[t] + r3 z pJlB[t] × pQlA[t] +
r4 z pJlB[t] × pQlA[t] – 2 r3 r4 z pJlB[t] × pQlA[t] – r3 S z pJlB[t] × pQlA[t] – r4 S z pJlB[t] × pQlA[t] +
2 r3 r4 S z pJlB[t] × pQlA[t] – r3 z α1 pJlB[t] × pQlA[t] – r4 z α1 pJlB[t] × pQlA[t] + 2 r3 r4 z α1 pJlB[t] × pQlA[t] +
r3 S z α1 pJlB[t] × pQlA[t] + r4 S z α1 pJlB[t] × pQlA[t] – 2 r3 r4 S z α1 pJlB[t] × pQlA[t] + r4 pJLB[t] × pQlA[t] –
r3 r4 pJLB[t] × pQlA[t] – r4 S pJLB[t] × pQlA[t] + r3 r4 S pJLB[t] × pQlA[t] – r4 y pJLB[t] × pQlA[t] + r3 r4 y pJLB[t] × pQlA[t] +
r4 S y pJLB[t] × pQlA[t] – r3 r4 S y pJLB[t] × pQlA[t] + r3 z pJLB[t] × pQlA[t] – r3 r4 z pJLB[t] × pQlA[t] –
r3 S z pJLB[t] × pQlA[t] + r3 r4 S z pJLB[t] × pQlA[t] – r4 α1 pJLB[t] × pQlA[t] + r3 r4 α1 pJLB[t] × pQlA[t] +
r4 S α1 pJLB[t] × pQlA[t] – r3 r4 S α1 pJLB[t] × pQlA[t] + r4 y α1 pJLB[t] × pQlA[t] – r3 r4 y α1 pJLB[t] × pQlA[t] –
r4 S y α1 pJLB[t] × pQlA[t] + r3 r4 S y α1 pJLB[t] × pQlA[t] – r3 z α1 pJLB[t] × pQlA[t] + r3 r4 z α1 pJLB[t] × pQlA[t] +
r3 S z α1 pJLB[t] × pQlA[t] – r3 r4 S z α1 pJLB[t] × pQlA[t] + β2 pJLB[t] × pQlA[t] – r3 β2 pJLB[t] × pQlA[t] –
r4 β2 pJLB[t] × pQlA[t] + r3 r4 β2 pJLB[t] × pQlA[t] – S β2 pJLB[t] × pQlA[t] + r3 S β2 pJLB[t] × pQlA[t] +
r4 S β2 pJLB[t] × pQlA[t] – r3 r4 S β2 pJLB[t] × pQlA[t] – y β2 pJLB[t] × pQlA[t] + r3 y β2 pJLB[t] × pQlA[t] +
r4 y β2 pJLB[t] × pQlA[t] – r3 r4 y β2 pJLB[t] × pQlA[t] + S y β2 pJLB[t] × pQlA[t] – r3 S y β2 pJLB[t] × pQlA[t] –
r4 S y β2 pJLB[t] × pQlA[t] + r3 r4 S y β2 pJLB[t] × pQlA[t] + r3 r4 z β2 pJLB[t] × pQlA[t] – r3 r4 S z β2 pJLB[t] × pQlA[t] +
pJLA[t] × pQLA[t] – S pJLA[t] × pQLA[t] – y pJLA[t] × pQLA[t] + S y pJLA[t] × pQLA[t] – α2 pJLA[t] × pQLA[t] +
S α2 pJLA[t] × pQLA[t] + y α2 pJLA[t] × pQLA[t] – S y α2 pJLA[t] × pQLA[t] + r3 pJlB[t] × pQLA[t] – r3 r4 pJlB[t] × pQLA[t] –
r3 S pJlB[t] × pQLA[t] + r3 r4 S pJlB[t] × pQLA[t] – r3 y pJlB[t] × pQLA[t] + r3 r4 y pJlB[t] × pQLA[t] + r3 S y pJlB[t] × pQLA[t] –
r3 r4 S y pJlB[t] × pQLA[t] + r4 z pJlB[t] × pQLA[t] – r3 r4 z pJlB[t] × pQLA[t] – r4 S z pJlB[t] × pQLA[t] +
r3 r4 S z pJlB[t] × pQLA[t] – r3 α2 pJlB[t] × pQLA[t] + r3 r4 α2 pJlB[t] × pQLA[t] + r3 S α2 pJlB[t] × pQLA[t] –
r3 r4 S α2 pJlB[t] × pQLA[t] + r3 y α2 pJlB[t] × pQLA[t] – r3 r4 y α2 pJlB[t] × pQLA[t] – r3 S y α2 pJlB[t] × pQLA[t] +
r3 r4 S y α2 pJlB[t] × pQLA[t] – r4 z α2 pJlB[t] × pQLA[t] + r3 r4 z α2 pJlB[t] × pQLA[t] + r4 S z α2 pJlB[t] × pQLA[t] –
r3 r4 S z α2 pJlB[t] × pQLA[t] + r3 pJLB[t] × pQLA[t] + r4 pJLB[t] × pQLA[t] – 2 r3 r4 pJLB[t] × pQLA[t] –
r3 S pJLB[t] × pQLA[t] – r4 S pJLB[t] × pQLA[t] + 2 r3 r4 S pJLB[t] × pQLA[t] – r3 y pJLB[t] × pQLA[t] –
r4 y pJLB[t] × pQLA[t] + 2 r3 r4 y pJLB[t] × pQLA[t] + r3 S y pJLB[t] × pQLA[t] + r4 S y pJLB[t] × pQLA[t] –
2 r3 r4 S y pJLB[t] × pQLA[t] – r3 α2 pJLB[t] × pQLA[t] – r4 α2 pJLB[t] × pQLA[t] + 2 r3 r4 α2 pJLB[t] × pQLA[t] +
r3 S α2 pJLB[t] × pQLA[t] + r4 S α2 pJLB[t] × pQLA[t] – 2 r3 r4 S α2 pJLB[t] × pQLA[t] + r3 y α2 pJLB[t] × pQLA[t] +
r4 y α2 pJLB[t] × pQLA[t] – 2 r3 r4 y α2 pJLB[t] × pQLA[t] – r3 S y α2 pJLB[t] × pQLA[t] – r4 S y α2 pJLB[t] × pQLA[t] +
2 r3 r4 S y α2 pJLB[t] × pQLA[t] + β2 pJLB[t] × pQLA[t] – r3 β2 pJLB[t] × pQLA[t] – r4 β2 pJLB[t] × pQLA[t] +
2 r3 r4 β2 pJLB[t] × pQLA[t] – S β2 pJLB[t] × pQLA[t] + r3 S β2 pJLB[t] × pQLA[t] + r4 S β2 pJLB[t] × pQLA[t] –
2 r3 r4 S β2 pJLB[t] × pQLA[t] – y β2 pJLB[t] × pQLA[t] + r3 y β2 pJLB[t] × pQLA[t] + r4 y β2 pJLB[t] × pQLA[t] –
2 r3 r4 y β2 pJLB[t] × pQLA[t] + S y β2 pJLB[t] × pQLA[t] – r3 S y β2 pJLB[t] × pQLA[t] – r4 S y β2 pJLB[t] × pQLA[t] +

2 r3 r4 S y β2 pJLB[t] × pQLA[t] + pJLA[t] × pQlB[t] − r3 pJLA[t] × pQlB[t] − r4 pJLA[t] × pQlB[t] + r3 r4 pJLA[t] × pQlB[t] −
S pJLA[t] × pQlB[t] + r3 S pJLA[t] × pQlB[t] + r4 S pJLA[t] × pQlB[t] − r3 r4 S pJLA[t] × pQlB[t] − y pJLA[t] × pQlB[t] +
r3 y pJLA[t] × pQlB[t] + r4 y pJLA[t] × pQlB[t] − r3 r4 y pJLA[t] × pQlB[t] + S y pJLA[t] × pQlB[t] − r3 S y pJLA[t] × pQlB[t] −
r4 S y pJLA[t] × pQlB[t] + r3 r4 S y pJLA[t] × pQlB[t] + r3 r4 z pJLA[t] × pQlB[t] − r3 r4 S z pJLA[t] × pQlB[t] −
α2 pJLA[t] × pQlB[t] + r3 α2 pJLA[t] × pQlB[t] + r4 α2 pJLA[t] × pQlB[t] − r3 r4 α2 pJLA[t] × pQlB[t] + S α2 pJLA[t] × pQlB[t] −
r3 S α2 pJLA[t] × pQlB[t] − r4 S α2 pJLA[t] × pQlB[t] + r3 r4 S α2 pJLA[t] × pQlB[t] + y α2 pJLA[t] × pQlB[t] −
r3 y α2 pJLA[t] × pQlB[t] − r4 y α2 pJLA[t] × pQlB[t] + r3 r4 y α2 pJLA[t] × pQlB[t] − S y α2 pJLA[t] × pQlB[t] +
r3 S y α2 pJLA[t] × pQlB[t] + r4 S y α2 pJLA[t] × pQlB[t] − r3 r4 S y α2 pJLA[t] × pQlB[t] − r3 r4 z α2 pJLA[t] × pQlB[t] +
r3 r4 S z α2 pJLA[t] × pQlB[t] + β2 pJLB[t] × pQlB[t] − r3 β2 pJLB[t] × pQlB[t] − r4 β2 pJLB[t] × pQlB[t] +
r3 r4 β2 pJLB[t] × pQlB[t] − S β2 pJLB[t] × pQlB[t] + r3 S β2 pJLB[t] × pQlB[t] + r4 S β2 pJLB[t] × pQlB[t] −
r3 r4 S β2 pJLB[t] × pQlB[t] − y β2 pJLB[t] × pQlB[t] + r3 y β2 pJLB[t] × pQlB[t] + r4 y β2 pJLB[t] × pQlB[t] −
r3 r4 y β2 pJLB[t] × pQlB[t] + S y β2 pJLB[t] × pQlB[t] − r3 S y β2 pJLB[t] × pQlB[t] − r4 S y β2 pJLB[t] × pQlB[t] +
r3 r4 S y β2 pJLB[t] × pQlB[t] + r3 r4 z β2 pJLB[t] × pQlB[t] − r3 r4 S z β2 pJLB[t] × pQlB[t] + pJLA[t] × pQLB[t] −
r3 pJLA[t] × pQLB[t] − r4 pJLA[t] × pQLB[t] + 2 r3 r4 pJLA[t] × pQLB[t] − S pJLA[t] × pQLB[t] + r3 S pJLA[t] × pQLB[t] +
r4 S pJLA[t] × pQLB[t] − 2 r3 r4 S pJLA[t] × pQLB[t] − y pJLA[t] × pQLB[t] + r3 y pJLA[t] × pQLB[t] + r4 y pJLA[t] × pQLB[t] −
2 r3 r4 y pJLA[t] × pQLB[t] + S y pJLA[t] × pQLB[t] − r3 S y pJLA[t] × pQLB[t] − r4 S y pJLA[t] × pQLB[t] +
2 r3 r4 S y pJLA[t] × pQLB[t] − α2 pJLA[t] × pQLB[t] + r3 α2 pJLA[t] × pQLB[t] + r4 α2 pJLA[t] × pQLB[t] −
2 r3 r4 α2 pJLA[t] × pQLB[t] + S α2 pJLA[t] × pQLB[t] − r3 S α2 pJLA[t] × pQLB[t] − r4 S α2 pJLA[t] × pQLB[t] +
2 r3 r4 S α2 pJLA[t] × pQLB[t] + y α2 pJLA[t] × pQLB[t] − r3 y α2 pJLA[t] × pQLB[t] − r4 y α2 pJLA[t] × pQLB[t] +
2 r3 r4 y α2 pJLA[t] × pQLB[t] − S y α2 pJLA[t] × pQLB[t] + r3 S y α2 pJLA[t] × pQLB[t] + r4 S y α2 pJLA[t] × pQLB[t] −
2 r3 r4 S y α2 pJLA[t] × pQLB[t] + r3 β2 pJLA[t] × pQLB[t] + r4 β2 pJLA[t] × pQLB[t] − 2 r3 r4 β2 pJLA[t] × pQLB[t] −
r3 S β2 pJLA[t] × pQLB[t] − r4 S β2 pJLA[t] × pQLB[t] + 2 r3 r4 S β2 pJLA[t] × pQLB[t] − r3 y β2 pJLA[t] × pQLB[t] −
r4 y β2 pJLA[t] × pQLB[t] + 2 r3 r4 y β2 pJLA[t] × pQLB[t] + r3 S y β2 pJLA[t] × pQLB[t] + r4 S y β2 pJLA[t] × pQLB[t] −
2 r3 r4 S y β2 pJLA[t] × pQLB[t] + r3 β2 pJlB[t] × pQLB[t] − r3 r4 β2 pJlB[t] × pQLB[t] − r3 S β2 pJlB[t] × pQLB[t] +
r3 r4 S β2 pJlB[t] × pQLB[t] − r3 y β2 pJlB[t] × pQLB[t] + r3 r4 y β2 pJlB[t] × pQLB[t] + r3 S y β2 pJlB[t] × pQLB[t] −
r3 r4 S y β2 pJlB[t] × pQLB[t] + r4 z β2 pJlB[t] × pQLB[t] − r3 r4 z β2 pJlB[t] × pQLB[t] − r4 S z β2 pJlB[t] × pQLB[t] +
r3 r4 S z β2 pJlB[t] × pQLB[t] + β2 pJLB[t] × pQLB[t] − S β2 pJLB[t] × pQLB[t] − y β2 pJLB[t] × pQLB[t] +
S y β2 pJLB[t] × pQLB[t] + β1 ((z − S z) pJlB[t]^2 − (−1 + S) (r4 − r4 y + r3 r4 (−1 + y − z) + r3 z) (pJLA[t] + pJLB[t]) pQlB[t] + pJlB[t]
(S z + r2 pJLA[t] − r2 S pJLA[t] − r2 y pJLA[t] + r2 S y pJLA[t] + z pJLA[t] − r2 z pJLA[t] − S z pJLA[t] + r2 S z pJLA[t] +
r2 pJLB[t] − r2 S pJLB[t] − r2 y pJLB[t] + r2 S y pJLB[t] + z pJLB[t] − r2 z pJLB[t] − S z pJLB[t] + r2 S z pJLB[t] −
(1 − r4 + r3 (−1 + 2 r4)) (−1 + S) z pQlA[t] + r3 r4 pQLA[t] − r3 r4 S pQLA[t] − r3 r4 y pQLA[t] + r3 r4 S y pQLA[t] +
z pQLA[t] − r3 z pQLA[t] − r4 z pQLA[t] + r3 r4 z pQLA[t] − S z pQLA[t] + r3 S z pQLA[t] + r4 S z pQLA[t] − r3 r4 S z
pQLA[t] + (z − S z) pQlB[t] + r3 r4 pQLB[t] − r3 r4 S pQLB[t] − r3 r4 y pQLB[t] + r3 r4 S y pQLB[t] + z pQLB[t] −
r3 z pQLB[t] − r4 z pQLB[t] + r3 r4 z pQLB[t] − S z pQLB[t] + r3 S z pQLB[t] + r4 S z pQLB[t] − r3 r4 S z pQLB[t])) +
pJlA[t] (S z + pJLA[t] − S pJLA[t] − y pJLA[t] + S y pJLA[t] + z pJLA[t] − S z pJLA[t] − α2 pJLA[t] + r2 α2 pJLA[t] +
S α2 pJLA[t] − r2 S α2 pJLA[t] + y α2 pJLA[t] − r2 y α2 pJLA[t] − S y α2 pJLA[t] + r2 S y α2 pJLA[t] − r2 z α2 pJLA[t] +
r2 S z α2 pJLA[t] + z pJlB[t] − S z pJlB[t] + z β1 pJlB[t] − S z β1 pJlB[t] + r2 pJLB[t] − r2 S pJLB[t] − r2 y pJLB[t] +
r2 S y pJLB[t] + z pJLB[t] − r2 z pJLB[t] − S z pJLB[t] + r2 S z pJLB[t] + β2 pJLB[t] − r2 β2 pJLB[t] − S β2 pJLB[t] +
r2 S β2 pJLB[t] − y β2 pJLB[t] + r2 y β2 pJLB[t] + S y β2 pJLB[t] − r2 S y β2 pJLB[t] + r2 z β2 pJLB[t] −
r2 S z β2 pJLB[t] + z pQlA[t] − S z pQlA[t] + r3 pQLA[t] − r3 S pQLA[t] − r3 y pQLA[t] + r3 S y pQLA[t] + z pQLA[t] −
r3 z pQLA[t] − S z pQLA[t] + r3 S z pQLA[t] − r3 α2 pQLA[t] + r3 r4 α2 pQLA[t] + r3 S α2 pQLA[t] − r3 r4 S α2 pQLA[t] +
r3 y α2 pQLA[t] − r3 r4 y α2 pQLA[t] − r3 S y α2 pQLA[t] + r3 r4 S y α2 pQLA[t] − r4 z α2 pQLA[t] + r3 r4 z α2 pQLA[t] +
r4 S z α2 pQLA[t] − r3 r4 S z α2 pQLA[t] + z pQlB[t] − r3 z pQlB[t] − r4 z pQlB[t] + 2 r3 r4 z pQlB[t] − S z pQlB[t] +
r3 S z pQlB[t] + r4 S z pQlB[t] − 2 r3 r4 S z pQlB[t] + r3 z β1 pQlB[t] + r4 z β1 pQlB[t] − 2 r3 r4 z β1 pQlB[t] −

$$r3\,S\,z\,\beta 1\,pQlB[t]-r4\,S\,z\,\beta 1\,pQlB[t]+2\,r3\,r4\,S\,z\,\beta 1\,pQlB[t]+r3\,r4\,pQLB[t]-r3\,r4\,S\,pQLB[t]-r3\,r4\,y\,pQLB[t]+$$
$$r3\,r4\,S\,y\,pQLB[t]+z\,pQLB[t]-r3\,z\,pQLB[t]-r4\,z\,pQLB[t]+r3\,r4\,z\,pQLB[t]-S\,z\,pQLB[t]+r3\,S\,z\,pQLB[t]+$$
$$r4\,S\,z\,pQLB[t]-r3\,r4\,S\,z\,pQLB[t]+r3\,\beta 2\,pQLB[t]-r3\,r4\,\beta 2\,pQLB[t]-r3\,S\,\beta 2\,pQLB[t]+r3\,r4\,S\,\beta 2\,pQLB[t]-$$
$$r3\,y\,\beta 2\,pQLB[t]+r3\,r4\,y\,\beta 2\,pQLB[t]+r3\,S\,y\,\beta 2\,pQLB[t]-r3\,r4\,S\,y\,\beta 2\,pQLB[t]+r4\,z\,\beta 2\,pQLB[t]-r3\,r4\,z\,\beta 2\,pQLB[t]-$$
$$r4\,S\,z\,\beta 2\,pQLB[t]+r3\,r4\,S\,z\,\beta 2\,pQLB[t]+\alpha 1\Big(-S\,z-r2\,pJLA[t]+r2\,S\,pJLA[t]+r2\,y\,pJLA[t]-r2\,S\,y\,pJLA[t]-z\,pJLA[t]+$$
$$r2\,z\,pJLA[t]+S\,z\,pJLA[t]-r2\,S\,z\,pJLA[t]-z\,pJlB[t]+S\,z\,pJlB[t]-r2\,pJLB[t]+r2\,S\,pJLB[t]+r2\,y\,pJLB[t]-$$
$$r2\,S\,y\,pJLB[t]-z\,pJLB[t]+r2\,z\,pJLB[t]+S\,z\,pJLB[t]-r2\,S\,z\,pJLB[t]+(-1+S)\,z\,pQlA[t]-r3\,r4\,pQLA[t]+$$
$$r3\,r4\,S\,pQLA[t]+r3\,r4\,y\,pQLA[t]-r3\,r4\,S\,y\,pQLA[t]-z\,pQLA[t]+r3\,z\,pQLA[t]+r4\,z\,pQLA[t]-r3\,r4\,z\,pQLA[t]+$$
$$S\,z\,pQLA[t]-r3\,S\,z\,pQLA[t]-r4\,S\,z\,pQLA[t]+r3\,r4\,S\,z\,pQLA[t]+\big(1-r3-r4+2\,r3\,r4\big)\,(-1+S)\,z\,pQlB[t]-$$
$$r3\,r4\,pQLB[t]+r3\,r4\,S\,pQLB[t]+r3\,r4\,y\,pQLB[t]-r3\,r4\,S\,y\,pQLB[t]-z\,pQLB[t]+r3\,z\,pQLB[t]+$$
$$r4\,z\,pQLB[t]-r3\,r4\,z\,pQLB[t]+S\,z\,pQLB[t]-r3\,S\,z\,pQLB[t]-r4\,S\,z\,pQLB[t]+r3\,r4\,S\,z\,pQLB[t]\Big)\Big)\Big)\Big/$$
$$\Big(\big(S+pJlA[t]-S\,pJlA[t]+pJLA[t]-S\,pJLA[t]+pJlB[t]-S\,pJlB[t]+pJLB[t]-S\,pJLB[t]-(-1+S)\,pQlA[t]+$$
$$pQLA[t]-S\,pQLA[t]-(-1+S)\,pQlB[t]+pQLB[t]-S\,pQLB[t]\big)$$
$$\big(pJlA[t]-k\,\alpha 1\,pJlA[t]+pJLA[t]-k\,\alpha 2\,pJLA[t]+pJlB[t]-k\,pJlB[t]+k\,\beta 1\,pJlB[t]+pJLB[t]-k\,pJLB[t]+k\,\beta 2\,pJLB[t]+$$
$$\big(1-k\,\alpha 1\big)\,pQlA[t]+pQLA[t]-k\,\alpha 2\,pQLA[t]+\big(1-k+k\,\beta 1\big)\,pQlB[t]+pQLB[t]-k\,pQLB[t]+k\,\beta 2\,pQLB[t]\big)\Big);$$

$$pJLB[t+1]=$$
$$\Big((-1+k)\,\Big(-S\,\alpha 2\,pJLA[t]+S\,y\,\alpha 2\,pJLA[t]-\alpha 2\,pJlA[t]\times pJLA[t]+r2\,\alpha 2\,pJlA[t]\times pJLA[t]+S\,\alpha 2\,pJlA[t]\times pJLA[t]-r2\,S\,\alpha 2\,pJlA[t]\times$$
$$pJLA[t]+y\,\alpha 2\,pJlA[t]\times pJLA[t]-r2\,y\,\alpha 2\,pJlA[t]\times pJLA[t]-S\,y\,\alpha 2\,pJlA[t]\times pJLA[t]+r2\,S\,y\,\alpha 2\,pJlA[t]\times pJLA[t]-$$
$$r2\,z\,\alpha 2\,pJlA[t]\times pJLA[t]+r2\,S\,z\,\alpha 2\,pJlA[t]\times pJLA[t]-\alpha 2\,pJLA[t]^2+S\,\alpha 2\,pJLA[t]^2+y\,\alpha 2\,pJLA[t]^2-$$
$$S\,y\,\alpha 2\,pJLA[t]^2-S\,z\,pJlB[t]+S\,z\,\beta 1\,pJlB[t]-z\,pJlA[t]\times pJlB[t]+S\,z\,pJlA[t]\times pJlB[t]+z\,\beta 1\,pJlA[t]\times pJlB[t]-$$
$$S\,z\,\beta 1\,pJlA[t]\times pJlB[t]-r2\,pJLA[t]\times pJlB[t]+r2\,S\,pJLA[t]\times pJlB[t]+r2\,y\,pJLA[t]\times pJlB[t]-r2\,S\,y\,pJLA[t]\times pJlB[t]-$$
$$z\,pJLA[t]\times pJlB[t]+r2\,z\,pJLA[t]\times pJlB[t]+S\,z\,pJLA[t]\times pJlB[t]-r2\,S\,z\,pJLA[t]\times pJlB[t]-\alpha 2\,pJLA[t]\times pJlB[t]+$$
$$r2\,\alpha 2\,pJLA[t]\times pJlB[t]+S\,\alpha 2\,pJLA[t]\times pJlB[t]-r2\,S\,\alpha 2\,pJLA[t]\times pJlB[t]+y\,\alpha 2\,pJLA[t]\times pJlB[t]-$$
$$r2\,y\,\alpha 2\,pJLA[t]\times pJlB[t]-S\,y\,\alpha 2\,pJLA[t]\times pJlB[t]+r2\,S\,y\,\alpha 2\,pJLA[t]\times pJlB[t]-r2\,z\,\alpha 2\,pJLA[t]\times pJlB[t]+$$
$$r2\,S\,z\,\alpha 2\,pJLA[t]\times pJlB[t]+r2\,\beta 1\,pJLA[t]\times pJlB[t]-r2\,S\,\beta 1\,pJLA[t]\times pJlB[t]-r2\,y\,\beta 1\,pJLA[t]\times pJlB[t]+$$
$$r2\,S\,y\,\beta 1\,pJLA[t]\times pJlB[t]+z\,\beta 1\,pJLA[t]\times pJlB[t]-r2\,z\,\beta 1\,pJLA[t]\times pJlB[t]-S\,z\,\beta 1\,pJLA[t]\times pJlB[t]+$$
$$r2\,S\,z\,\beta 1\,pJLA[t]\times pJlB[t]-z\,pJlB[t]^2+S\,z\,pJlB[t]^2+z\,\beta 1\,pJlB[t]^2-S\,z\,\beta 1\,pJlB[t]^2-S\,pJLB[t]+S\,y\,pJLB[t]+$$
$$S\,\beta 2\,pJLB[t]-S\,y\,\beta 2\,pJLB[t]-pJlA[t]\times pJLB[t]+r2\,pJlA[t]\times pJLB[t]+S\,pJlA[t]\times pJLB[t]-r2\,S\,pJlA[t]\times pJLB[t]+$$
$$y\,pJlA[t]\times pJLB[t]-r2\,y\,pJlA[t]\times pJLB[t]-S\,y\,pJlA[t]\times pJLB[t]+r2\,S\,y\,pJlA[t]\times pJLB[t]-r2\,z\,pJlA[t]\times pJLB[t]+$$
$$r2\,S\,z\,pJlA[t]\times pJLB[t]+\beta 2\,pJlA[t]\times pJLB[t]-r2\,\beta 2\,pJlA[t]\times pJLB[t]-S\,\beta 2\,pJlA[t]\times pJLB[t]+r2\,S\,\beta 2\,pJlA[t]\times pJLB[t]-$$
$$y\,\beta 2\,pJlA[t]\times pJLB[t]+r2\,y\,\beta 2\,pJlA[t]\times pJLB[t]+S\,y\,\beta 2\,pJlA[t]\times pJLB[t]-r2\,S\,y\,\beta 2\,pJlA[t]\times pJLB[t]+$$
$$r2\,z\,\beta 2\,pJlA[t]\times pJLB[t]-r2\,S\,z\,\beta 2\,pJlA[t]\times pJLB[t]-pJLA[t]\times pJLB[t]+S\,pJLA[t]\times pJLB[t]+y\,pJLA[t]\times pJLB[t]-$$
$$S\,y\,pJLA[t]\times pJLB[t]-\alpha 2\,pJLA[t]\times pJLB[t]+S\,\alpha 2\,pJLA[t]\times pJLB[t]+y\,\alpha 2\,pJLA[t]\times pJLB[t]-S\,y\,\alpha 2\,pJLA[t]\times pJLB[t]+$$
$$\beta 2\,pJLA[t]\times pJLB[t]-S\,\beta 2\,pJLA[t]\times pJLB[t]-y\,\beta 2\,pJLA[t]\times pJLB[t]+S\,y\,\beta 2\,pJLA[t]\times pJLB[t]-pJlB[t]\times pJLB[t]+$$
$$S\,pJlB[t]\times pJLB[t]+y\,pJlB[t]\times pJLB[t]-S\,y\,pJlB[t]\times pJLB[t]-z\,pJlB[t]\times pJLB[t]+S\,z\,pJlB[t]\times pJLB[t]+$$
$$r2\,\beta 1\,pJlB[t]\times pJLB[t]-r2\,S\,\beta 1\,pJlB[t]\times pJLB[t]-r2\,y\,\beta 1\,pJlB[t]\times pJLB[t]+r2\,S\,y\,\beta 1\,pJlB[t]\times pJLB[t]+$$
$$z\,\beta 1\,pJlB[t]\times pJLB[t]-r2\,z\,\beta 1\,pJlB[t]\times pJLB[t]-S\,z\,\beta 1\,pJlB[t]\times pJLB[t]+r2\,S\,z\,\beta 1\,pJlB[t]\times pJLB[t]+$$
$$\beta 2\,pJlB[t]\times pJLB[t]-r2\,\beta 2\,pJlB[t]\times pJLB[t]-S\,\beta 2\,pJlB[t]\times pJLB[t]+r2\,S\,\beta 2\,pJlB[t]\times pJLB[t]-y\,\beta 2\,pJlB[t]\times pJLB[t]+$$
$$r2\,y\,\beta 2\,pJlB[t]\times pJLB[t]+S\,y\,\beta 2\,pJlB[t]\times pJLB[t]-r2\,S\,y\,\beta 2\,pJlB[t]\times pJLB[t]+r2\,z\,\beta 2\,pJlB[t]\times pJLB[t]-$$
$$r2\,S\,z\,\beta 2\,pJlB[t]\times pJLB[t]-pJLB[t]^2+S\,pJLB[t]^2+y\,pJLB[t]^2-S\,y\,pJLB[t]^2+\beta 2\,pJLB[t]^2-S\,\beta 2\,pJLB[t]^2-$$
$$y\,\beta 2\,pJLB[t]^2+S\,y\,\beta 2\,pJLB[t]^2-\alpha 2\,pJLA[t]\times pQlA[t]+r3\,\alpha 2\,pJLA[t]\times pQlA[t]+r4\,\alpha 2\,pJLA[t]\times pQlA[t]-$$
$$r3\,r4\,\alpha 2\,pJLA[t]\times pQlA[t]+S\,\alpha 2\,pJLA[t]\times pQlA[t]-r3\,S\,\alpha 2\,pJLA[t]\times pQlA[t]-r4\,S\,\alpha 2\,pJLA[t]\times pQlA[t]+$$

r3 r4 S α2 pJLA[t] × pQlA[t] + y α2 pJLA[t] × pQlA[t] – r3 y α2 pJLA[t] × pQlA[t] – r4 y α2 pJLA[t] × pQlA[t] +
r3 r4 y α2 pJLA[t] × pQlA[t] – S y α2 pJLA[t] × pQlA[t] + r3 S y α2 pJLA[t] × pQlA[t] + r4 S y α2 pJLA[t] × pQlA[t] –
r3 r4 S y α2 pJLA[t] × pQlA[t] – r3 r4 z α2 pJLA[t] × pQlA[t] + r3 r4 S z α2 pJLA[t] × pQlA[t] – z pJlB[t] × pQlA[t] +
r3 z pJlB[t] × pQlA[t] + r4 z pJlB[t] × pQlA[t] – 2 r3 r4 z pJlB[t] × pQlA[t] + S z pJlB[t] × pQlA[t] – r3 S z pJlB[t] × pQlA[t] –
r4 S z pJlB[t] × pQlA[t] + 2 r3 r4 S z pJlB[t] × pQlA[t] + z β1 pJlB[t] × pQlA[t] – r3 z β1 pJlB[t] × pQlA[t] –
r4 z β1 pJlB[t] × pQlA[t] + 2 r3 r4 z β1 pJlB[t] × pQlA[t] – S z β1 pJlB[t] × pQlA[t] + r3 S z β1 pJlB[t] × pQlA[t] +
r4 S z β1 pJlB[t] × pQlA[t] – 2 r3 r4 S z β1 pJlB[t] × pQlA[t] – pJLB[t] × pQlA[t] + r3 pJLB[t] × pQlA[t] +
r4 pJLB[t] × pQlA[t] – r3 r4 pJLB[t] × pQlA[t] + S pJLB[t] × pQlA[t] – r3 S pJLB[t] × pQlA[t] – r4 S pJLB[t] × pQlA[t] +
r3 r4 S pJLB[t] × pQlA[t] + y pJLB[t] × pQlA[t] – r3 y pJLB[t] × pQlA[t] – r4 y pJLB[t] × pQlA[t] + r3 r4 y pJLB[t] × pQlA[t] –
S y pJLB[t] × pQlA[t] + r3 S y pJLB[t] × pQlA[t] + r4 S y pJLB[t] × pQlA[t] – r3 r4 S y pJLB[t] × pQlA[t] –
r3 r4 z pJLB[t] × pQlA[t] + r3 r4 S z pJLB[t] × pQlA[t] + β2 pJLB[t] × pQlA[t] – r3 β2 pJLB[t] × pQlA[t] –
r4 β2 pJLB[t] × pQlA[t] + r3 r4 β2 pJLB[t] × pQlA[t] – S β2 pJLB[t] × pQlA[t] + r3 S β2 pJLB[t] × pQlA[t] +
r4 S β2 pJLB[t] × pQlA[t] – r3 r4 S β2 pJLB[t] × pQlA[t] – y β2 pJLB[t] × pQlA[t] + r3 y β2 pJLB[t] × pQlA[t] +
r4 y β2 pJLB[t] × pQlA[t] – r3 r4 y β2 pJLB[t] × pQlA[t] + S y β2 pJLB[t] × pQlA[t] – r3 S y β2 pJLB[t] × pQlA[t] –
r4 S y β2 pJLB[t] × pQlA[t] + r3 r4 S y β2 pJLB[t] × pQlA[t] + r3 r4 z β2 pJLB[t] × pQlA[t] – r3 r4 S z β2 pJLB[t] × pQlA[t] –
r3 α2 pJlA[t] × pQLA[t] + r3 r4 α2 pJlA[t] × pQLA[t] + r3 S α2 pJlA[t] × pQLA[t] – r3 r4 S α2 pJlA[t] × pQLA[t] +
r3 y α2 pJlA[t] × pQLA[t] – r3 r4 y α2 pJlA[t] × pQLA[t] – r3 S y α2 pJlA[t] × pQLA[t] + r3 r4 S y α2 pJlA[t] × pQLA[t] –
r4 z α2 pJlA[t] × pQLA[t] + r3 r4 z α2 pJlA[t] × pQLA[t] + r4 S z α2 pJlA[t] × pQLA[t] – r3 r4 S z α2 pJlA[t] × pQLA[t] –
α2 pJLA[t] × pQLA[t] + S α2 pJLA[t] × pQLA[t] + y α2 pJLA[t] × pQLA[t] – S y α2 pJLA[t] × pQLA[t] –
r3 r4 pJlB[t] × pQLA[t] + r3 r4 S pJlB[t] × pQLA[t] + r3 r4 y pJlB[t] × pQLA[t] – r3 r4 S y pJlB[t] × pQLA[t] –
z pJlB[t] × pQLA[t] + r3 z pJlB[t] × pQLA[t] + r4 z pJlB[t] × pQLA[t] – r3 r4 z pJlB[t] × pQLA[t] + S z pJlB[t] × pQLA[t] –
r3 S z pJlB[t] × pQLA[t] – r4 S z pJlB[t] × pQLA[t] + r3 r4 S z pJlB[t] × pQLA[t] – r3 α2 pJlB[t] × pQLA[t] +
r3 r4 α2 pJlB[t] × pQLA[t] + r3 S α2 pJlB[t] × pQLA[t] – r3 r4 S α2 pJlB[t] × pQLA[t] + r3 y α2 pJlB[t] × pQLA[t] –
r3 r4 y α2 pJlB[t] × pQLA[t] – r3 S y α2 pJlB[t] × pQLA[t] + r3 r4 S y α2 pJlB[t] × pQLA[t] – r4 z α2 pJlB[t] × pQLA[t] +
r3 r4 z α2 pJlB[t] × pQLA[t] + r4 S z α2 pJlB[t] × pQLA[t] – r3 r4 S z α2 pJlB[t] × pQLA[t] + r3 r4 β1 pJlB[t] × pQLA[t] –
r3 r4 S β1 pJlB[t] × pQLA[t] – r3 r4 y β1 pJlB[t] × pQLA[t] + r3 r4 S y β1 pJlB[t] × pQLA[t] + z β1 pJlB[t] × pQLA[t] –
r3 z β1 pJlB[t] × pQLA[t] – r4 z β1 pJlB[t] × pQLA[t] + r3 r4 z β1 pJlB[t] × pQLA[t] – S z β1 pJlB[t] × pQLA[t] +
r3 S z β1 pJlB[t] × pQLA[t] + r4 S z β1 pJlB[t] × pQLA[t] – r3 r4 S z β1 pJlB[t] × pQLA[t] – pJLB[t] × pQLA[t] +
r3 pJLB[t] × pQLA[t] + r4 pJLB[t] × pQLA[t] – 2 r3 r4 pJLB[t] × pQLA[t] + S pJLB[t] × pQLA[t] – r3 S pJLB[t] × pQLA[t] –
r4 S pJLB[t] × pQLA[t] + 2 r3 r4 S pJLB[t] × pQLA[t] + y pJLB[t] × pQLA[t] – r3 y pJLB[t] × pQLA[t] – r4 y pJLB[t] × pQLA[t] +
2 r3 r4 y pJLB[t] × pQLA[t] – S y pJLB[t] × pQLA[t] + r3 S y pJLB[t] × pQLA[t] + r4 S y pJLB[t] × pQLA[t] –
2 r3 r4 S y pJLB[t] × pQLA[t] – r3 α2 pJLB[t] × pQLA[t] – r4 α2 pJLB[t] × pQLA[t] + 2 r3 r4 α2 pJLB[t] × pQLA[t] +
r3 S α2 pJLB[t] × pQLA[t] + r4 S α2 pJLB[t] × pQLA[t] – 2 r3 r4 S α2 pJLB[t] × pQLA[t] + r3 y α2 pJLB[t] × pQLA[t] +
r4 y α2 pJLB[t] × pQLA[t] – 2 r3 r4 y α2 pJLB[t] × pQLA[t] – r3 S y α2 pJLB[t] × pQLA[t] – r4 S y α2 pJLB[t] × pQLA[t] +
2 r3 r4 S y α2 pJLB[t] × pQLA[t] + β2 pJLB[t] × pQLA[t] – r3 β2 pJLB[t] × pQLA[t] – r4 β2 pJLB[t] × pQLA[t] +
2 r3 r4 β2 pJLB[t] × pQLA[t] – S β2 pJLB[t] × pQLA[t] + r3 S β2 pJLB[t] × pQLA[t] + r4 S β2 pJLB[t] × pQLA[t] –
2 r3 r4 S β2 pJLB[t] × pQLA[t] – y β2 pJLB[t] × pQLA[t] + r3 y β2 pJLB[t] × pQLA[t] + r4 y β2 pJLB[t] × pQLA[t] –
2 r3 r4 y β2 pJLB[t] × pQLA[t] + S y β2 pJLB[t] × pQLA[t] – r3 S y β2 pJLB[t] × pQLA[t] – r4 S y β2 pJLB[t] × pQLA[t] +
2 r3 r4 S y β2 pJLB[t] × pQLA[t] + (–1 + S) ((–r4 + r3 (–1 + 2 r4)) z (–1 + β1) pJlA[t] + r4 pJLA[t] – r3 r4 pJLA[t] – r4 y pJLA[t] +
r3 r4 y pJLA[t] + r3 z pJLA[t] – r3 r4 z pJLA[t] + α2 pJLA[t] – r3 α2 pJLA[t] – r4 α2 pJLA[t] + r3 r4 α2 pJLA[t] –
y α2 pJLA[t] + r3 y α2 pJLA[t] + r4 y α2 pJLA[t] – r3 r4 y α2 pJLA[t] + r3 r4 z α2 pJLA[t] – r4 β1 pJLA[t] +
r3 r4 β1 pJLA[t] + r4 y β1 pJLA[t] – r3 r4 y β1 pJLA[t] – r3 z β1 pJLA[t] + r3 r4 z β1 pJLA[t] + (z – z β1) pJlB[t] +
pJLB[t] – r3 pJLB[t] – y pJLB[t] + r3 y pJLB[t] + r3 z pJLB[t] – r4 β1 pJLB[t] + r3 r4 β1 pJLB[t] + r4 y β1 pJLB[t] –
r3 r4 y β1 pJLB[t] – r3 z β1 pJLB[t] + r3 r4 z β1 pJLB[t] – β2 pJLB[t] + r3 β2 pJLB[t] + r4 β2 pJLB[t] –
r3 r4 β2 pJLB[t] + y β2 pJLB[t] – r3 y β2 pJLB[t] – r4 y β2 pJLB[t] + r3 r4 y β2 pJLB[t] – r3 r4 z β2 pJLB[t]) pQlB[t] –

r3 pJlA[t] × pQLB[t] + r3 r4 pJlA[t] × pQLB[t] + r3 S pJlA[t] × pQLB[t] − r3 r4 S pJlA[t] × pQLB[t] +
r3 y pJlA[t] × pQLB[t] − r3 r4 y pJlA[t] × pQLB[t] − r3 S y pJlA[t] × pQLB[t] + r3 r4 S y pJlA[t] × pQLB[t] −
r4 z pJlA[t] × pQLB[t] + r3 r4 z pJlA[t] × pQLB[t] + r4 S z pJlA[t] × pQLB[t] − r3 r4 S z pJlA[t] × pQLB[t] +
r3 β2 pJlA[t] × pQLB[t] − r3 r4 β2 pJlA[t] × pQLB[t] − r3 S β2 pJlA[t] × pQLB[t] + r3 r4 S β2 pJlA[t] × pQLB[t] −
r3 y β2 pJlA[t] × pQLB[t] + r3 r4 y β2 pJlA[t] × pQLB[t] + r3 S y β2 pJlA[t] × pQLB[t] − r3 r4 S y β2 pJlA[t] × pQLB[t] +
r4 z β2 pJlA[t] × pQLB[t] − r3 r4 z β2 pJlA[t] × pQLB[t] − r4 S z β2 pJlA[t] × pQLB[t] + r3 r4 S z β2 pJlA[t] × pQLB[t] −
r3 pJLA[t] × pQLB[t] − r4 pJLA[t] × pQLB[t] + 2 r3 r4 pJLA[t] × pQLB[t] + r3 S pJLA[t] × pQLB[t] + r4 S pJLA[t] × pQLB[t] −
2 r3 r4 S pJLA[t] × pQLB[t] + r3 y pJLA[t] × pQLB[t] + r4 y pJLA[t] × pQLB[t] − 2 r3 r4 y pJLA[t] × pQLB[t] −
r3 S y pJLA[t] × pQLB[t] − r4 S y pJLA[t] × pQLB[t] + 2 r3 r4 S y pJLA[t] × pQLB[t] − α2 pJLA[t] × pQLB[t] +
r3 α2 pJLA[t] × pQLB[t] + r4 α2 pJLA[t] × pQLB[t] − 2 r3 r4 α2 pJLA[t] × pQLB[t] + S α2 pJLA[t] × pQLB[t] −
r3 S α2 pJLA[t] × pQLB[t] − r4 S α2 pJLA[t] × pQLB[t] + 2 r3 r4 S α2 pJLA[t] × pQLB[t] + y α2 pJLA[t] × pQLB[t] −
r3 y α2 pJLA[t] × pQLB[t] − r4 y α2 pJLA[t] × pQLB[t] + 2 r3 r4 y α2 pJLA[t] × pQLB[t] − S y α2 pJLA[t] × pQLB[t] +
r3 S y α2 pJLA[t] × pQLB[t] + r4 S y α2 pJLA[t] × pQLB[t] − 2 r3 r4 S y α2 pJLA[t] × pQLB[t] + r3 β2 pJLA[t] × pQLB[t] +
r4 β2 pJLA[t] × pQLB[t] − 2 r3 r4 β2 pJLA[t] × pQLB[t] − r3 S β2 pJLA[t] × pQLB[t] − r4 S β2 pJLA[t] × pQLB[t] +
2 r3 r4 S β2 pJLA[t] × pQLB[t] − r3 y β2 pJLA[t] × pQLB[t] − r4 y β2 pJLA[t] × pQLB[t] + 2 r3 r4 y β2 pJLA[t] × pQLB[t] +
r3 S y β2 pJLA[t] × pQLB[t] + r4 S y β2 pJLA[t] × pQLB[t] − 2 r3 r4 S y β2 pJLA[t] × pQLB[t] − r3 pJlB[t] × pQLB[t] +
r3 S pJlB[t] × pQLB[t] + r3 y pJlB[t] × pQLB[t] − r3 S y pJlB[t] × pQLB[t] − z pJlB[t] × pQLB[t] + r3 z pJlB[t] × pQLB[t] +
S z pJlB[t] × pQLB[t] − r3 S z pJlB[t] × pQLB[t] + r3 r4 β1 pJlB[t] × pQLB[t] − r3 r4 S β1 pJlB[t] × pQLB[t] −
r3 r4 y β1 pJlB[t] × pQLB[t] + r3 r4 S y β1 pJlB[t] × pQLB[t] + z β1 pJlB[t] × pQLB[t] − r3 z β1 pJlB[t] × pQLB[t] −
r4 z β1 pJlB[t] × pQLB[t] + r3 r4 z β1 pJlB[t] × pQLB[t] − S z β1 pJlB[t] × pQLB[t] + r3 S z β1 pJlB[t] × pQLB[t] +
r4 S z β1 pJlB[t] × pQLB[t] − r3 r4 S z β1 pJlB[t] × pQLB[t] + r3 β2 pJlB[t] × pQLB[t] − r3 r4 β2 pJlB[t] × pQLB[t] −
r3 S β2 pJlB[t] × pQLB[t] + r3 r4 S β2 pJlB[t] × pQLB[t] − r3 y β2 pJlB[t] × pQLB[t] + r3 r4 y β2 pJlB[t] × pQLB[t] +
r3 S y β2 pJlB[t] × pQLB[t] − r3 r4 S y β2 pJlB[t] × pQLB[t] + r4 z β2 pJlB[t] × pQLB[t] − r3 r4 z β2 pJlB[t] × pQLB[t] −
r4 S z β2 pJlB[t] × pQLB[t] + r3 r4 S z β2 pJlB[t] × pQLB[t] − pJLB[t] × pQLB[t] + S pJLB[t] × pQLB[t] + y pJLB[t] × pQLB[t] −
S y pJLB[t] × pQLB[t] + β2 pJLB[t] × pQLB[t] − S β2 pJLB[t] × pQLB[t] − y β2 pJLB[t] × pQLB[t] + S y β2 pJLB[t] × pQLB[t] +
α1 ((−1 + S) z pJlA[t]^2 − (−1 + S) ((−r4 + r3 (−1 + 2 r4)) z pJlB[t] − (r4 − r4 y + r3 r4 (−1 + y − z) + r3 z) (pJLA[t] + pJLB[t]))
pQlA[t] + pJlA[t] (−S z − r2 pJLA[t] + r2 S pJLA[t] + r2 y pJLA[t] − r2 S y pJLA[t] − z pJLA[t] + r2 z pJLA[t] +
S z pJLA[t] − r2 S z pJLA[t] − z pJlB[t] + S z pJlB[t] − r2 pJLB[t] + r2 S pJLB[t] + r2 y pJLB[t] − r2 S y pJLB[t] −
z pJLB[t] + r2 z pJLB[t] + S z pJLB[t] − r2 S z pJLB[t] + (−1 + S) z pQlA[t] − r3 r4 pQLA[t] + r3 r4 S pQLA[t] +
r3 r4 y pQLA[t] − r3 r4 S y pQLA[t] − z pQLA[t] + r3 z pQLA[t] + r4 z pQLA[t] − r3 r4 z pQLA[t] +
S z pQLA[t] − r3 S z pQLA[t] − r4 S z pQLA[t] + r3 r4 S z pQLA[t] + (1 − r3 − r4 + 2 r3 r4) (−1 + S) z pQlB[t] −
r3 r4 pQLB[t] + r3 r4 S pQLB[t] + r3 r4 y pQLB[t] − r3 r4 S y pQLB[t] − z pQLB[t] + r3 z pQLB[t] +
r4 z pQLB[t] − r3 r4 z pQLB[t] + S z pQLB[t] − r3 S z pQLB[t] − r4 S z pQLB[t] + r3 r4 S z pQLB[t]))))/
((S + pJlA[t] − S pJlA[t] + pJLA[t] − S pJLA[t] + pJlB[t] − S pJlB[t] + pJLB[t] − S pJLB[t] − (−1 + S) pQlA[t] +
pQLA[t] − S pQLA[t] − (−1 + S) pQlB[t] + pQLB[t] − S pQLB[t])
(pJlA[t] − k α1 pJlA[t] + pJLA[t] − k α2 pJLA[t] + pJlB[t] − k pJlB[t] + k β1 pJlB[t] + pJLB[t] − k pJLB[t] + k β2 pJLB[t] +
(1 − k α1) pQlA[t] + pQLA[t] − k α2 pQLA[t] + (1 − k + k β1) pQlB[t] + pQLB[t] − k pQLB[t] + k β2 pQLB[t]));

pQlA[t + 1] =
(−((−1 + S) (−1 + z) (−1 + α1) pQlA[t]^2) + S y pQLA[t] − S y α2 pQLA[t] + r3 pJlA[t] × pQLA[t] − r3 S pJlA[t] × pQLA[t] + y pJlA[t] × pQLA[t] −
r3 y pJlA[t] × pQLA[t] − S y pJlA[t] × pQLA[t] + r3 S y pJlA[t] × pQLA[t] − r3 z pJlA[t] × pQLA[t] + r3 S z pJlA[t] × pQLA[t] −
r3 α1 pJlA[t] × pQLA[t] + r3 r4 α1 pJlA[t] × pQLA[t] + r3 S α1 pJlA[t] × pQLA[t] − r3 r4 S α1 pJlA[t] × pQLA[t] −
r4 y α1 pJlA[t] × pQLA[t] + r3 r4 y α1 pJlA[t] × pQLA[t] + r4 S y α1 pJlA[t] × pQLA[t] − r3 r4 S y α1 pJlA[t] × pQLA[t] +

r3 z α1 pJlA[t] × pQLA[t] – r3 r4 z α1 pJlA[t] × pQLA[t] – r3 S z α1 pJlA[t] × pQLA[t] + r3 r4 S z α1 pJlA[t] × pQLA[t] –
r3 r4 α2 pJlA[t] × pQLA[t] + r3 r4 S α2 pJlA[t] × pQLA[t] – y α2 pJlA[t] × pQLA[t] + r3 y α2 pJlA[t] × pQLA[t] +
r4 y α2 pJlA[t] × pQLA[t] – r3 r4 y α2 pJlA[t] × pQLA[t] + S y α2 pJlA[t] × pQLA[t] – r3 S y α2 pJlA[t] × pQLA[t] –
r4 S y α2 pJlA[t] × pQLA[t] + r3 r4 S y α2 pJlA[t] × pQLA[t] + r3 r4 z α2 pJlA[t] × pQLA[t] – r3 r4 S z α2 pJlA[t] × pQLA[t] +
y pJLA[t] × pQLA[t] – S y pJLA[t] × pQLA[t] – y α2 pJLA[t] × pQLA[t] + S y α2 pJLA[t] × pQLA[t] + r3 r4 pJlB[t] × pQLA[t] –
r3 r4 S pJlB[t] × pQLA[t] + y pJlB[t] × pQLA[t] – r3 y pJlB[t] × pQLA[t] – r4 y pJlB[t] × pQLA[t] + r3 r4 y pJlB[t] × pQLA[t] –
S y pJlB[t] × pQLA[t] + r3 S y pJlB[t] × pQLA[t] + r4 S y pJlB[t] × pQLA[t] – r3 r4 S y pJlB[t] × pQLA[t] –
r3 r4 z pJlB[t] × pQLA[t] + r3 r4 S z pJlB[t] × pQLA[t] – r3 r4 α2 pJlB[t] × pQLA[t] + r3 r4 S α2 pJlB[t] × pQLA[t] –
y α2 pJlB[t] × pQLA[t] + r3 y α2 pJlB[t] × pQLA[t] + r4 y α2 pJlB[t] × pQLA[t] – r3 r4 y α2 pJlB[t] × pQLA[t] +
S y α2 pJlB[t] × pQLA[t] – r3 S y α2 pJlB[t] × pQLA[t] – r4 S y α2 pJlB[t] × pQLA[t] + r3 r4 S y α2 pJlB[t] × pQLA[t] +
r3 r4 z α2 pJlB[t] × pQLA[t] – r3 r4 S z α2 pJlB[t] × pQLA[t] + y pJLB[t] × pQLA[t] – r3 y pJLB[t] × pQLA[t] –
r4 y pJLB[t] × pQLA[t] + 2 r3 r4 y pJLB[t] × pQLA[t] – S y pJLB[t] × pQLA[t] + r3 S y pJLB[t] × pQLA[t] + r4 S y pJLB[t] × pQLA[t] –
2 r3 r4 S y pJLB[t] × pQLA[t] – y α2 pJLB[t] × pQLA[t] + r3 y α2 pJLB[t] × pQLA[t] + r4 y α2 pJLB[t] × pQLA[t] –
2 r3 r4 y α2 pJLB[t] × pQLA[t] + S y α2 pJLB[t] × pQLA[t] – r3 S y α2 pJLB[t] × pQLA[t] – r4 S y α2 pJLB[t] × pQLA[t] +
2 r3 r4 S y α2 pJLB[t] × pQLA[t] + r3 y β2 pJLB[t] × pQLA[t] + r4 y β2 pJLB[t] × pQLA[t] – 2 r3 r4 y β2 pJLB[t] × pQLA[t] –
r3 S y β2 pJLB[t] × pQLA[t] – r4 S y β2 pJLB[t] × pQLA[t] + 2 r3 r4 S y β2 pJLB[t] × pQLA[t] + y pQLA[t]^2 – S y pQLA[t]^2 –
y α2 pQLA[t]^2 + S y α2 pQLA[t]^2 + r3 pJlA[t] × pQlB[t] + r4 pJlA[t] × pQlB[t] – 2 r3 r4 pJlA[t] × pQlB[t] – r3 S pJlA[t] × pQlB[t] –
r4 S pJlA[t] × pQlB[t] + 2 r3 r4 S pJlA[t] × pQlB[t] – r3 z pJlA[t] × pQlB[t] – r4 z pJlA[t] × pQlB[t] + 2 r3 r4 z pJlA[t] × pQlB[t] +
r3 S z pJlA[t] × pQlB[t] + r4 S z pJlA[t] × pQlB[t] – 2 r3 r4 S z pJlA[t] × pQlB[t] – r3 α1 pJlA[t] × pQlB[t] –
r4 α1 pJlA[t] × pQlB[t] + 2 r3 r4 α1 pJlA[t] × pQlB[t] + r3 S α1 pJlA[t] × pQlB[t] + r4 S α1 pJlA[t] × pQlB[t] –
2 r3 r4 S α1 pJlA[t] × pQlB[t] + r3 z α1 pJlA[t] × pQlB[t] + r4 z α1 pJlA[t] × pQlB[t] – 2 r3 r4 z α1 pJlA[t] × pQlB[t] –
r3 S z α1 pJlA[t] × pQlB[t] – r4 S z α1 pJlA[t] × pQlB[t] + 2 r3 r4 S z α1 pJlA[t] × pQlB[t] + r4 pJLA[t] × pQlB[t] –
r3 r4 pJLA[t] × pQlB[t] – r4 S pJLA[t] × pQlB[t] + r3 r4 S pJLA[t] × pQlB[t] + r3 y pJLA[t] × pQlB[t] – r3 r4 y pJLA[t] × pQlB[t] –
r3 S y pJLA[t] × pQlB[t] + r3 r4 S y pJLA[t] × pQlB[t] – r4 z pJLA[t] × pQlB[t] + r3 r4 z pJLA[t] × pQlB[t] +
r4 S z pJLA[t] × pQlB[t] – r3 r4 S z pJLA[t] × pQlB[t] – r4 α2 pJLA[t] × pQlB[t] + r3 r4 α2 pJLA[t] × pQlB[t] +
r4 S α2 pJLA[t] × pQlB[t] – r3 r4 S α2 pJLA[t] × pQlB[t] – r3 y α2 pJLA[t] × pQlB[t] + r3 r4 y α2 pJLA[t] × pQlB[t] +
r3 S y α2 pJLA[t] × pQlB[t] – r3 r4 S y α2 pJLA[t] × pQlB[t] + r4 z α2 pJLA[t] × pQlB[t] – r3 r4 z α2 pJLA[t] × pQlB[t] –
r4 S z α2 pJLA[t] × pQlB[t] + r3 r4 S z α2 pJLA[t] × pQlB[t] + r4 β2 pJLB[t] × pQlB[t] – r3 r4 β2 pJLB[t] × pQlB[t] –
r4 S β2 pJLB[t] × pQlB[t] + r3 r4 S β2 pJLB[t] × pQlB[t] + r3 y β2 pJLB[t] × pQlB[t] – r3 r4 y β2 pJLB[t] × pQlB[t] –
r3 S y β2 pJLB[t] × pQlB[t] + r3 r4 S y β2 pJLB[t] × pQlB[t] – r4 z β2 pJLB[t] × pQlB[t] + r3 r4 z β2 pJLB[t] × pQlB[t] +
r4 S z β2 pJLB[t] × pQlB[t] – r3 r4 S z β2 pJLB[t] × pQlB[t] + r6 pQLA[t] × pQlB[t] – r6 S pQLA[t] × pQlB[t] + y pQLA[t] × pQlB[t] –
r6 y pQLA[t] × pQlB[t] – S y pQLA[t] × pQlB[t] + r6 S y pQLA[t] × pQlB[t] – r6 z pQLA[t] × pQlB[t] + r6 S z pQLA[t] × pQlB[t] –
r6 α2 pQLA[t] × pQlB[t] + r6 S α2 pQLA[t] × pQlB[t] – y α2 pQLA[t] × pQlB[t] + r6 y α2 pQLA[t] × pQlB[t] +
S y α2 pQLA[t] × pQlB[t] – r6 S y α2 pQLA[t] × pQlB[t] + r6 z α2 pQLA[t] × pQlB[t] – r6 S z α2 pQLA[t] × pQlB[t] +
S y β2 pQLB[t] + r3 pJlA[t] × pQLB[t] – r3 r4 pJlA[t] × pQLB[t] – r3 S pJlA[t] × pQLB[t] + r3 r4 S pJlA[t] × pQLB[t] +
r4 y pJlA[t] × pQLB[t] – r3 r4 y pJlA[t] × pQLB[t] – r4 S y pJlA[t] × pQLB[t] + r3 r4 S y pJlA[t] × pQLB[t] –
r3 z pJlA[t] × pQLB[t] + r3 r4 z pJlA[t] × pQLB[t] + r3 S z pJlA[t] × pQLB[t] – r3 r4 S z pJlA[t] × pQLB[t] –
r3 α1 pJlA[t] × pQLB[t] + r3 r4 α1 pJlA[t] × pQLB[t] + r3 S α1 pJlA[t] × pQLB[t] – r3 r4 S α1 pJlA[t] × pQLB[t] –
r4 y α1 pJlA[t] × pQLB[t] + r3 r4 y α1 pJlA[t] × pQLB[t] + r4 S y α1 pJlA[t] × pQLB[t] – r3 r4 S y α1 pJlA[t] × pQLB[t] +
r3 z α1 pJlA[t] × pQLB[t] – r3 r4 z α1 pJlA[t] × pQLB[t] – r3 S z α1 pJlA[t] × pQLB[t] + r3 r4 S z α1 pJlA[t] × pQLB[t] +
r3 r4 β2 pJlA[t] × pQLB[t] – r3 r4 S β2 pJlA[t] × pQLB[t] + y β2 pJlA[t] × pQLB[t] – r3 y β2 pJlA[t] × pQLB[t] –
r4 y β2 pJlA[t] × pQLB[t] + r3 r4 y β2 pJlA[t] × pQLB[t] – S y β2 pJlA[t] × pQLB[t] + r3 S y β2 pJlA[t] × pQLB[t] +
r4 S y β2 pJlA[t] × pQLB[t] – r3 r4 S y β2 pJlA[t] × pQLB[t] – r3 r4 z β2 pJlA[t] × pQLB[t] + r3 r4 S z β2 pJlA[t] × pQLB[t] +
r3 y pJLA[t] × pQLB[t] + r4 y pJLA[t] × pQLB[t] – 2 r3 r4 y pJLA[t] × pQLB[t] – r3 S y pJLA[t] × pQLB[t] –
r4 S y pJLA[t] × pQLB[t] + 2 r3 r4 S y pJLA[t] × pQLB[t] – r3 y α2 pJLA[t] × pQLB[t] – r4 y α2 pJLA[t] × pQLB[t] +

2 r3 r4 y α2 pJLA[t] × pQLB[t] + r3 S y α2 pJLA[t] × pQLB[t] + r4 S y α2 pJLA[t] × pQLB[t] − 2 r3 r4 S y α2 pJLA[t] × pQLB[t] +
y β2 pJLA[t] × pQLB[t] − r3 y β2 pJLA[t] × pQLB[t] − r4 y β2 pJLA[t] × pQLB[t] + 2 r3 r4 y β2 pJLA[t] × pQLB[t] −
S y β2 pJLA[t] × pQLB[t] + r3 S y β2 pJLA[t] × pQLB[t] + r4 S y β2 pJLA[t] × pQLB[t] − 2 r3 r4 S y β2 pJLA[t] × pQLB[t] +
r3 r4 β2 pJlB[t] × pQLB[t] − r3 r4 S β2 pJlB[t] × pQLB[t] + y β2 pJlB[t] × pQLB[t] − r3 y β2 pJlB[t] × pQLB[t] −
r4 y β2 pJlB[t] × pQLB[t] + r3 r4 y β2 pJlB[t] × pQLB[t] − S y β2 pJlB[t] × pQLB[t] + r3 S y β2 pJlB[t] × pQLB[t] +
r4 S y β2 pJlB[t] × pQLB[t] − r3 r4 S y β2 pJlB[t] × pQLB[t] − r3 r4 z β2 pJlB[t] × pQLB[t] + r3 r4 S z β2 pJlB[t] × pQLB[t] +
y β2 pJLB[t] × pQLB[t] − S y β2 pJLB[t] × pQLB[t] + y pQLA[t] × pQLB[t] − S y pQLA[t] × pQLB[t] − y α2 pQLA[t] × pQLB[t] +
S y α2 pQLA[t] × pQLB[t] + y β2 pQLA[t] × pQLB[t] − S y β2 pQLA[t] × pQLB[t] + r6 β2 pQlB[t] × pQLB[t] −
r6 S β2 pQlB[t] × pQLB[t] + y β2 pQlB[t] × pQLB[t] − r6 y β2 pQlB[t] × pQLB[t] − S y β2 pQlB[t] × pQLB[t] +
r6 S y β2 pQlB[t] × pQLB[t] − r6 z β2 pQlB[t] × pQLB[t] + r6 S z β2 pQlB[t] × pQLB[t] + y β2 pQLB[t]^2 − S y β2 pQLB[t]^2 +
pQlA[t] (S − S z + pJlA[t] − S pJlA[t] − z pJlA[t] + S z pJlA[t] + pJLA[t] − r3 pJLA[t] − S pJLA[t] + r3 S pJLA[t] + r3 y pJLA[t] −
r3 S y pJLA[t] − z pJLA[t] + r3 z pJLA[t] + S z pJLA[t] − r3 S z pJLA[t] − r4 α2 pJLA[t] + r3 r4 α2 pJLA[t] + r4 S α2 pJLA[t] −
r3 r4 S α2 pJLA[t] − r3 y α2 pJLA[t] + r3 r4 y α2 pJLA[t] + r3 S y α2 pJLA[t] − r3 r4 S y α2 pJLA[t] + r4 z α2 pJLA[t] −
r3 r4 z α2 pJLA[t] − r4 S z α2 pJLA[t] + r3 r4 S z α2 pJLA[t] + pJlB[t] − r3 pJlB[t] − r4 pJlB[t] + 2 r3 r4 pJlB[t] −
S pJlB[t] + r3 S pJlB[t] + r4 S pJlB[t] − 2 r3 r4 S pJlB[t] − z pJlB[t] + r3 z pJlB[t] + r4 z pJlB[t] − 2 r3 r4 z pJlB[t] +
S z pJlB[t] − r3 S z pJlB[t] − r4 S z pJlB[t] + 2 r3 r4 S z pJlB[t] + r3 β1 pJlB[t] + r4 β1 pJlB[t] − 2 r3 r4 β1 pJlB[t] −
r3 S β1 pJlB[t] − r4 S β1 pJlB[t] + 2 r3 r4 S β1 pJlB[t] − r3 z β1 pJlB[t] − r4 z β1 pJlB[t] + 2 r3 r4 z β1 pJlB[t] +
r3 S z β1 pJlB[t] + r4 S z β1 pJlB[t] − 2 r3 r4 S z β1 pJlB[t] + pJLB[t] − r3 pJLB[t] − r4 pJLB[t] + r3 r4 pJLB[t] −
S pJLB[t] + r3 S pJLB[t] + r4 S pJLB[t] − r3 r4 S pJLB[t] + r3 r4 y pJLB[t] − r3 r4 S y pJLB[t] − z pJLB[t] + r3 z pJLB[t] +
r4 z pJLB[t] − r3 r4 z pJLB[t] + S z pJLB[t] − r3 S z pJLB[t] − r4 S z pJLB[t] + r3 r4 S z pJLB[t] + r4 β2 pJLB[t] −
r3 r4 β2 pJLB[t] − r4 S β2 pJLB[t] + r3 r4 S β2 pJLB[t] + r3 y β2 pJLB[t] − r3 r4 y β2 pJLB[t] − r3 S y β2 pJLB[t] +
r3 r4 S y β2 pJLB[t] − r4 z β2 pJLB[t] + r3 r4 z β2 pJLB[t] + r4 S z β2 pJLB[t] − r3 r4 S z β2 pJLB[t] + pQLA[t] −
S pQLA[t] + y pQLA[t] − S y pQLA[t] − z pQLA[t] + S z pQLA[t] − r6 α2 pQLA[t] + r6 S α2 pQLA[t] − y α2 pQLA[t] +
r6 y α2 pQLA[t] + S y α2 pQLA[t] − r6 S y α2 pQLA[t] + r6 z α2 pQLA[t] − r6 S z α2 pQLA[t] + pQlB[t] − S pQlB[t] −
z pQlB[t] + S z pQlB[t] + β1 pQlB[t] − S β1 pQlB[t] − z β1 pQlB[t] + S z β1 pQlB[t] + pQLB[t] − r6 pQLB[t] −
S pQLB[t] + r6 S pQLB[t] + r6 y pQLB[t] − r6 S y pQLB[t] − z pQLB[t] + r6 z pQLB[t] + S z pQLB[t] − r6 S z pQLB[t] +
r6 β2 pQLB[t] − r6 S β2 pQLB[t] + y β2 pQLB[t] − r6 y β2 pQLB[t] − S y β2 pQLB[t] + r6 S y β2 pQLB[t] −
r6 z β2 pQLB[t] + r6 S z β2 pQLB[t] − α1 (S − S z + pJlA[t] − S pJlA[t] − z pJlA[t] + S z pJlA[t] + pJLA[t] − r3 pJLA[t] −
r4 pJLA[t] + r3 r4 pJLA[t] − S pJLA[t] + r3 S pJLA[t] + r4 S pJLA[t] − r3 r4 S pJLA[t] + r3 r4 y pJLA[t] −
r3 r4 S y pJLA[t] − z pJLA[t] + r3 z pJLA[t] + r4 z pJLA[t] − r3 r4 z pJLA[t] + S z pJLA[t] − r3 S z pJLA[t] −
r4 S z pJLA[t] + r3 r4 S z pJLA[t] + pJlB[t] − r3 pJlB[t] − r4 pJlB[t] + 2 r3 r4 pJlB[t] − S pJlB[t] + r3 S pJlB[t] +
r4 S pJlB[t] − 2 r3 r4 S pJlB[t] − z pJlB[t] + r3 z pJlB[t] + r4 z pJlB[t] − 2 r3 r4 z pJlB[t] + S z pJlB[t] −
r3 S z pJlB[t] − r4 S z pJlB[t] + 2 r3 r4 S z pJlB[t] + pJLB[t] − r3 pJLB[t] − r4 pJLB[t] + r3 r4 pJLB[t] −
S pJLB[t] + r3 S pJLB[t] + r4 S pJLB[t] − r3 r4 S pJLB[t] + r3 r4 y pJLB[t] − r3 r4 S y pJLB[t] − z pJLB[t] +
r3 z pJLB[t] + r4 z pJLB[t] − r3 r4 z pJLB[t] + S z pJLB[t] − r3 S z pJLB[t] − r4 S z pJLB[t] + r3 r4 S z pJLB[t] −
(−1 + S) (1 − z + r6 (−1 + y + z)) pQLA[t] + (−1 + S) (−1 + z) pQlB[t] + pQLB[t] − r6 pQLB[t] − S pQLB[t] +
r6 S pQLB[t] + r6 y pQLB[t] − r6 S y pQLB[t] − z pQLB[t] + r6 z pQLB[t] + S z pQLB[t] − r6 S z pQLB[t])) +
β1 ((−1 + S) (−1 + z) pQlB[t]^2 + (−1 + S) (−r4 y + r3 (−1 + r4 (1 + y − z) + z)) pJlB[t] (pQLA[t] + pQLB[t]) +
pQlB[t] (S − S z + pJlA[t] − r3 pJlA[t] − r4 pJlA[t] + 2 r3 r4 pJlA[t] − S pJlA[t] + r3 S pJlA[t] + r4 S pJlA[t] −
2 r3 r4 S pJlA[t] − z pJlA[t] + r3 z pJlA[t] + r4 z pJlA[t] − 2 r3 r4 z pJlA[t] + S z pJlA[t] − r3 S z pJlA[t] −
r4 S z pJlA[t] + 2 r3 r4 S z pJlA[t] + pJLA[t] − r3 pJLA[t] − r4 pJLA[t] + r3 r4 pJLA[t] − S pJLA[t] +
r3 S pJLA[t] + r4 S pJLA[t] − r3 r4 S pJLA[t] + r3 r4 y pJLA[t] − r3 r4 S y pJLA[t] − z pJLA[t] + r3 z pJLA[t] +
r4 z pJLA[t] − r3 r4 z pJLA[t] + S z pJLA[t] − r3 S z pJLA[t] − r4 S z pJLA[t] + r3 r4 S z pJLA[t] +
pJlB[t] − S pJlB[t] − z pJlB[t] + S z pJlB[t] + pJLB[t] − r3 pJLB[t] − r4 pJLB[t] + r3 r4 pJLB[t] −

```
S pJLB[t] + r3 S pJLB[t] + r4 S pJLB[t] − r3 r4 S pJLB[t] + r3 r4 y pJLB[t] − r3 r4 S y pJLB[t] −
z pJLB[t] + r3 z pJLB[t] + r4 z pJLB[t] − r3 r4 z pJLB[t] + S z pJLB[t] − r3 S z pJLB[t] − r4 S z pJLB[t] +
r3 r4 S z pJLB[t] − (−1 + S) (1 − z + r6 (−1 + y + z)) pQLA[t] − (−1 + S) (1 − z + r6 (−1 + y + z)) pQLB[t]))) /
((S + pJlA[t] − S pJlA[t] + pJLA[t] − S pJLA[t] + pJlB[t] − S pJlB[t] + pJLB[t] − S pJLB[t] − (−1 + S) pQlA[t] +
pQLA[t] − S pQLA[t] − (−1 + S) pQlB[t] + pQLB[t] − S pQLB[t])
(pJlA[t] − k α1 pJlA[t] + pJLA[t] − k α2 pJLA[t] + pJlB[t] − k pJlB[t] + k β1 pJlB[t] + pJLB[t] − k pJLB[t] + k β2 pJLB[t] +
(1 − k α1) pQlA[t] + pQLA[t] − k α2 pQLA[t] + (1 − k + k β1) pQlB[t] + pQLB[t] − k pQLB[t] + k β2 pQLB[t]));


pQlB[t + 1] =
−(((−1 + k) (r4 α2 pJLA[t] × pQlA[t] − r3 r4 α2 pJLA[t] × pQlA[t] − r4 S α2 pJLA[t] × pQlA[t] + r3 r4 S α2 pJLA[t] × pQlA[t] + r3 y α2
pJLA[t] × pQlA[t] − r3 r4 y α2 pJLA[t] × pQlA[t] − r3 S y α2 pJLA[t] × pQlA[t] + r3 r4 S y α2 pJLA[t] × pQlA[t] −
r4 z α2 pJLA[t] × pQlA[t] + r3 r4 z α2 pJLA[t] × pQlA[t] + r4 S z α2 pJLA[t] × pQlA[t] − r3 r4 S z α2 pJLA[t] × pQlA[t] +
r3 pJlB[t] × pQlA[t] + r4 pJlB[t] × pQlA[t] − 2 r3 r4 pJlB[t] × pQlA[t] − r3 S pJlB[t] × pQlA[t] − r4 S pJlB[t] × pQlA[t] +
2 r3 r4 S pJlB[t] × pQlA[t] − r3 z pJlB[t] × pQlA[t] − r4 z pJlB[t] × pQlA[t] + 2 r3 r4 z pJlB[t] × pQlA[t] +
r3 S z pJlB[t] × pQlA[t] + r4 S z pJlB[t] × pQlA[t] − 2 r3 r4 S z pJlB[t] × pQlA[t] − r3 β1 pJlB[t] × pQlA[t] −
r4 β1 pJlB[t] × pQlA[t] + 2 r3 r4 β1 pJlB[t] × pQlA[t] + r3 S β1 pJlB[t] × pQlA[t] + r4 S β1 pJlB[t] × pQlA[t] −
2 r3 r4 S β1 pJlB[t] × pQlA[t] + r3 z β1 pJlB[t] × pQlA[t] + r4 z β1 pJlB[t] × pQlA[t] − 2 r3 r4 z β1 pJlB[t] × pQlA[t] −
r3 S z β1 pJlB[t] × pQlA[t] − r4 S z β1 pJlB[t] × pQlA[t] + 2 r3 r4 S z β1 pJlB[t] × pQlA[t] + r4 pJLB[t] × pQlA[t] −
r3 r4 pJLB[t] × pQlA[t] − r4 S pJLB[t] × pQlA[t] + r3 r4 S pJLB[t] × pQlA[t] + r3 y pJLB[t] × pQlA[t] −
r3 r4 y pJLB[t] × pQlA[t] − r3 S y pJLB[t] × pQlA[t] + r3 r4 S y pJLB[t] × pQlA[t] − r4 z pJLB[t] × pQlA[t] +
r3 r4 z pJLB[t] × pQlA[t] + r4 S z pJLB[t] × pQlA[t] − r3 r4 S z pJLB[t] × pQlA[t] − r4 β2 pJLB[t] × pQlA[t] +
r3 r4 β2 pJLB[t] × pQlA[t] + r4 S β2 pJLB[t] × pQlA[t] − r3 r4 S β2 pJLB[t] × pQlA[t] − r3 y β2 pJLB[t] × pQlA[t] +
r3 r4 y β2 pJLB[t] × pQlA[t] + r3 S y β2 pJLB[t] × pQlA[t] − r3 r4 S y β2 pJLB[t] × pQlA[t] + r4 z β2 pJLB[t] × pQlA[t] −
r3 r4 z β2 pJLB[t] × pQlA[t] − r4 S z β2 pJLB[t] × pQlA[t] + r3 r4 S z β2 pJLB[t] × pQlA[t] + S y α2 pQLA[t] +
r3 r4 α2 pJlA[t] × pQLA[t] − r3 r4 S α2 pJlA[t] × pQLA[t] + y α2 pJlA[t] × pQLA[t] − r3 y α2 pJlA[t] × pQLA[t] −
r4 y α2 pJlA[t] × pQLA[t] + r3 r4 y α2 pJlA[t] × pQLA[t] − S y α2 pJlA[t] × pQLA[t] + r3 S y α2 pJlA[t] × pQLA[t] +
r4 S y α2 pJlA[t] × pQLA[t] − r3 r4 S y α2 pJlA[t] × pQLA[t] − r3 r4 z α2 pJlA[t] × pQLA[t] +
r3 r4 S z α2 pJlA[t] × pQLA[t] + y α2 pJLA[t] × pQLA[t] − S y α2 pJLA[t] × pQLA[t] + r3 pJlB[t] × pQLA[t] −
r3 r4 pJlB[t] × pQLA[t] − r3 S pJlB[t] × pQLA[t] + r3 r4 S pJlB[t] × pQLA[t] + r4 y pJlB[t] × pQLA[t] −
r3 r4 y pJlB[t] × pQLA[t] − r4 S y pJlB[t] × pQLA[t] + r3 r4 S y pJlB[t] × pQLA[t] − r3 z pJlB[t] × pQLA[t] +
r3 r4 z pJlB[t] × pQLA[t] + r3 S z pJlB[t] × pQLA[t] − r3 r4 S z pJlB[t] × pQLA[t] + r3 r4 α2 pJlB[t] × pQLA[t] −
r3 r4 S α2 pJlB[t] × pQLA[t] + y α2 pJlB[t] × pQLA[t] − r3 y α2 pJlB[t] × pQLA[t] − r4 y α2 pJlB[t] × pQLA[t] +
r3 r4 y α2 pJlB[t] × pQLA[t] − S y α2 pJlB[t] × pQLA[t] + r3 S y α2 pJlB[t] × pQLA[t] + r4 S y α2 pJlB[t] × pQLA[t] −
r3 r4 S y α2 pJlB[t] × pQLA[t] − r3 r4 z α2 pJlB[t] × pQLA[t] + r3 r4 S z α2 pJlB[t] × pQLA[t] −
r3 β1 pJlB[t] × pQLA[t] + r3 r4 β1 pJlB[t] × pQLA[t] + r3 S β1 pJlB[t] × pQLA[t] − r3 r4 S β1 pJlB[t] × pQLA[t] −
r4 y β1 pJlB[t] × pQLA[t] + r3 r4 y β1 pJlB[t] × pQLA[t] + r4 S y β1 pJlB[t] × pQLA[t] − r3 r4 S y β1 pJlB[t] × pQLA[t] +
r3 z β1 pJlB[t] × pQLA[t] − r3 r4 z β1 pJlB[t] × pQLA[t] − r3 S z β1 pJlB[t] × pQLA[t] + r3 r4 S z β1 pJlB[t] × pQLA[t] +
r3 y pJLB[t] × pQLA[t] + r4 y pJLB[t] × pQLA[t] − 2 r3 r4 y pJLB[t] × pQLA[t] − r3 S y pJLB[t] × pQLA[t] −
r4 S y pJLB[t] × pQLA[t] + 2 r3 r4 S y pJLB[t] × pQLA[t] + y α2 pJLB[t] × pQLA[t] − r3 y α2 pJLB[t] × pQLA[t] −
r4 y α2 pJLB[t] × pQLA[t] + 2 r3 r4 y α2 pJLB[t] × pQLA[t] − S y α2 pJLB[t] × pQLA[t] + r3 S y α2 pJLB[t] × pQLA[t] +
r4 S y α2 pJLB[t] × pQLA[t] − 2 r3 r4 S y α2 pJLB[t] × pQLA[t] − r3 y β2 pJLB[t] × pQLA[t] −
r4 y β2 pJLB[t] × pQLA[t] + 2 r3 r4 y β2 pJLB[t] × pQLA[t] + r3 S y β2 pJLB[t] × pQLA[t] + r4 S y β2 pJLB[t] × pQLA[t] −
2 r3 r4 S y β2 pJLB[t] × pQLA[t] + r6 α2 pQlA[t] × pQLA[t] − r6 S α2 pQlA[t] × pQLA[t] + y α2 pQlA[t] × pQLA[t] −
```

r6 y α2 pQlA[t] × pQLA[t] – S y α2 pQlA[t] × pQLA[t] + r6 S y α2 pQlA[t] × pQLA[t] – r6 z α2 pQlA[t] × pQLA[t] +
r6 S z α2 pQlA[t] × pQLA[t] + y α2 pQLA[t]^2 – S y α2 pQLA[t]^2 – (–1 + S) (–1 + z) (–1 + β1) pQlB[t]^2 +
S y pQLB[t] – S y β2 pQLB[t] + r3 r4 pJlA[t] × pQLB[t] – r3 r4 S pJlA[t] × pQLB[t] + y pJlA[t] × pQLB[t] –
r3 y pJlA[t] × pQLB[t] – r4 y pJlA[t] × pQLB[t] + r3 r4 y pJlA[t] × pQLB[t] – S y pJlA[t] × pQLB[t] +
r3 S y pJlA[t] × pQLB[t] + r4 S y pJlA[t] × pQLB[t] – r3 r4 S y pJlA[t] × pQLB[t] – r3 r4 z pJlA[t] × pQLB[t] +
r3 r4 S z pJlA[t] × pQLB[t] – r3 r4 β2 pJlA[t] × pQLB[t] + r3 r4 S β2 pJlA[t] × pQLB[t] – y β2 pJlA[t] × pQLB[t] +
r3 y β2 pJlA[t] × pQLB[t] + r4 y β2 pJlA[t] × pQLB[t] – r3 r4 y β2 pJlA[t] × pQLB[t] + S y β2 pJlA[t] × pQLB[t] –
r3 S y β2 pJlA[t] × pQLB[t] – r4 S y β2 pJlA[t] × pQLB[t] + r3 r4 S y β2 pJlA[t] × pQLB[t] +
r3 r4 z β2 pJlA[t] × pQLB[t] – r3 r4 S z β2 pJlA[t] × pQLB[t] + y pJLA[t] × pQLB[t] – r3 y pJLA[t] × pQLB[t] –
r4 y pJLA[t] × pQLB[t] + 2 r3 r4 y pJLA[t] × pQLB[t] – S y pJLA[t] × pQLB[t] + r3 S y pJLA[t] × pQLB[t] +
r4 S y pJLA[t] × pQLB[t] – 2 r3 r4 S y pJLA[t] × pQLB[t] + r3 y α2 pJLA[t] × pQLB[t] + r4 y α2 pJLA[t] × pQLB[t] –
2 r3 r4 y α2 pJLA[t] × pQLB[t] – r3 S y α2 pJLA[t] × pQLB[t] – r4 S y α2 pJLA[t] × pQLB[t] +
2 r3 r4 S y α2 pJLA[t] × pQLB[t] – y β2 pJLA[t] × pQLB[t] + r3 y β2 pJLA[t] × pQLB[t] + r4 y β2 pJLA[t] × pQLB[t] –
2 r3 r4 y β2 pJLA[t] × pQLB[t] + S y β2 pJLA[t] × pQLB[t] – r3 S y β2 pJLA[t] × pQLB[t] – r4 S y β2 pJLA[t] × pQLB[t] +
2 r3 r4 S y β2 pJLA[t] × pQLB[t] + r3 pJlB[t] × pQLB[t] – r3 S pJlB[t] × pQLB[t] + y pJlB[t] × pQLB[t] –
r3 y pJlB[t] × pQLB[t] – S y pJlB[t] × pQLB[t] + r3 S y pJlB[t] × pQLB[t] – r3 z pJlB[t] × pQLB[t] +
r3 S z pJlB[t] × pQLB[t] – r3 β1 pJlB[t] × pQLB[t] + r3 r4 β1 pJlB[t] × pQLB[t] + r3 S β1 pJlB[t] × pQLB[t] –
r3 r4 S β1 pJlB[t] × pQLB[t] – r4 y β1 pJlB[t] × pQLB[t] + r3 r4 y β1 pJlB[t] × pQLB[t] + r4 S y β1 pJlB[t] × pQLB[t] –
r3 r4 S y β1 pJlB[t] × pQLB[t] + r3 z β1 pJlB[t] × pQLB[t] – r3 r4 z β1 pJlB[t] × pQLB[t] – r3 S z β1 pJlB[t] × pQLB[t] +
r3 r4 S z β1 pJlB[t] × pQLB[t] – r3 r4 β2 pJlB[t] × pQLB[t] + r3 r4 S β2 pJlB[t] × pQLB[t] – y β2 pJlB[t] × pQLB[t] +
r3 y β2 pJlB[t] × pQLB[t] + r4 y β2 pJlB[t] × pQLB[t] – r3 r4 y β2 pJlB[t] × pQLB[t] + S y β2 pJlB[t] × pQLB[t] –
r3 S y β2 pJlB[t] × pQLB[t] – r4 S y β2 pJlB[t] × pQLB[t] + r3 r4 S y β2 pJlB[t] × pQLB[t] + r3 r4 z β2 pJlB[t] × pQLB[t] –
r3 r4 S z β2 pJlB[t] × pQLB[t] + y pJLB[t] × pQLB[t] – S y pJLB[t] × pQLB[t] – y β2 pJLB[t] × pQLB[t] +
S y β2 pJLB[t] × pQLB[t] + r6 pQlA[t] × pQLB[t] – r6 S pQlA[t] × pQLB[t] + y pQlA[t] × pQLB[t] – r6 y pQlA[t] × pQLB[t] –
S y pQlA[t] × pQLB[t] + r6 S y pQlA[t] × pQLB[t] – r6 z pQlA[t] × pQLB[t] + r6 S z pQlA[t] × pQLB[t] –
r6 β2 pQlA[t] × pQLB[t] + r6 S β2 pQlA[t] × pQLB[t] – y β2 pQlA[t] × pQLB[t] + r6 y β2 pQlA[t] × pQLB[t] +
S y β2 pQlA[t] × pQLB[t] – r6 S y β2 pQlA[t] × pQLB[t] + r6 z β2 pQlA[t] × pQLB[t] – r6 S z β2 pQlA[t] × pQLB[t] +
y pQLA[t] × pQLB[t] – S y pQLA[t] × pQLB[t] + y α2 pQLA[t] × pQLB[t] – S y α2 pQLA[t] × pQLB[t] –
y β2 pQLA[t] × pQLB[t] + S y β2 pQLA[t] × pQLB[t] + y pQLB[t]^2 – S y pQLB[t]^2 – y β2 pQLB[t]^2 + S y β2 pQLB[t]^2 +
pQlB[t] (S – S z + pJlA[t] – r3 pJlA[t] – r4 pJlA[t] + 2 r3 r4 pJlA[t] – S pJlA[t] + r3 S pJlA[t] + r4 S pJlA[t] –
2 r3 r4 S pJlA[t] – z pJlA[t] + r3 z pJlA[t] + r4 z pJlA[t] – 2 r3 r4 z pJlA[t] + S z pJlA[t] – r3 S z pJlA[t] –
r4 S z pJlA[t] + 2 r3 r4 S z pJlA[t] + pJLA[t] – r3 pJLA[t] – r4 pJLA[t] + r3 r4 pJLA[t] – S pJLA[t] + r3 S pJLA[t] +
r4 S pJLA[t] – r3 r4 S pJLA[t] + r3 r4 y pJLA[t] – r3 r4 S y pJLA[t] – z pJLA[t] + r3 z pJLA[t] + r4 z pJLA[t] –
r3 r4 z pJLA[t] + S z pJLA[t] – r3 S z pJLA[t] – r4 S z pJLA[t] + r3 r4 S z pJLA[t] + r4 α2 pJLA[t] – r3 r4 α2
pJLA[t] – r4 S α2 pJLA[t] + r3 r4 S α2 pJLA[t] + r3 y α2 pJLA[t] – r3 r4 y α2 pJLA[t] – r3 S y α2 pJLA[t] +
r3 r4 S y α2 pJLA[t] – r4 z α2 pJLA[t] + r3 r4 z α2 pJLA[t] + r4 S z α2 pJLA[t] – r3 r4 S z α2 pJLA[t] +
pJlB[t] – S pJlB[t] – z pJlB[t] + S z pJlB[t] + pJLB[t] – r3 pJLB[t] – S pJLB[t] + r3 S pJLB[t] + r3 y pJLB[t] –
r3 S y pJLB[t] – z pJLB[t] + r3 z pJLB[t] + S z pJLB[t] – r3 S z pJLB[t] – r4 β2 pJLB[t] + r3 r4 β2 pJLB[t] +
r4 S β2 pJLB[t] – r3 r4 S β2 pJLB[t] – r3 y β2 pJLB[t] + r3 r4 y β2 pJLB[t] + r3 S y β2 pJLB[t] –
r3 r4 S y β2 pJLB[t] + r4 z β2 pJLB[t] – r3 r4 z β2 pJLB[t] – r4 S z β2 pJLB[t] + r3 r4 S z β2 pJLB[t] + pQlA[t] –
S pQlA[t] – z pQlA[t] + S z pQlA[t] + pQLA[t] – r6 pQLA[t] – S pQLA[t] + r6 S pQLA[t] + r6 y pQLA[t] –
r6 S y pQLA[t] – z pQLA[t] + r6 z pQLA[t] + S z pQLA[t] – r6 S z pQLA[t] + r6 α2 pQLA[t] – r6 S α2 pQLA[t] +
y α2 pQLA[t] – r6 y α2 pQLA[t] – S y α2 pQLA[t] + r6 S y α2 pQLA[t] – r6 z α2 pQLA[t] + r6 S z α2 pQLA[t] +
pQLB[t] – S pQLB[t] + y pQLB[t] – S y pQLB[t] – z pQLB[t] + S z pQLB[t] – r6 β2 pQLB[t] + r6 S β2 pQLB[t] –
y β2 pQLB[t] + r6 y β2 pQLB[t] + S y β2 pQLB[t] – r6 S y β2 pQLB[t] + r6 z β2 pQLB[t] – r6 S z β2 pQLB[t] –

$$\beta 1 \Big(S - S z + pJlA[t] - r3\, pJlA[t] - r4\, pJlA[t] + 2\, r3\, r4\, pJlA[t] - S\, pJlA[t] + r3\, S\, pJlA[t] + r4\, S\, pJlA[t] - 2\, r3\, r4\, S$$
$$pJlA[t] - z\, pJlA[t] + r3\, z\, pJlA[t] + r4\, z\, pJlA[t] - 2\, r3\, r4\, z\, pJlA[t] + S\, z\, pJlA[t] - r3\, S\, z\, pJlA[t] - r4\, S$$
$$z\, pJlA[t] + 2\, r3\, r4\, S\, z\, pJlA[t] + pJLA[t] - r3\, pJLA[t] - r4\, pJLA[t] + r3\, r4\, pJLA[t] - S\, pJLA[t] + r3\, S$$
$$pJLA[t] + r4\, S\, pJLA[t] - r3\, r4\, S\, pJLA[t] + r3\, r4\, y\, pJLA[t] - r3\, r4\, S\, y\, pJLA[t] - z\, pJLA[t] + r3\, z\, pJLA[t] +$$
$$r4\, z\, pJLA[t] - r3\, r4\, z\, pJLA[t] + S\, z\, pJLA[t] - r3\, S\, z\, pJLA[t] - r4\, S\, z\, pJLA[t] + r3\, r4\, S\, z\, pJLA[t] + pJlB[$$
$$t] - S\, pJlB[t] - z\, pJlB[t] + S\, z\, pJlB[t] + pJLB[t] - r3\, pJLB[t] - r4\, pJLB[t] + r3\, r4\, pJLB[t] - S\, pJLB[t] + r3$$
$$S\, pJLB[t] + r4\, S\, pJLB[t] - r3\, r4\, S\, pJLB[t] + r3\, r4\, y\, pJLB[t] - r3\, r4\, S\, y\, pJLB[t] - z\, pJLB[t] + r3\, z\, pJLB[$$
$$t] + r4\, z\, pJLB[t] - r3\, r4\, z\, pJLB[t] + S\, z\, pJLB[t] - r3\, S\, z\, pJLB[t] - r4\, S\, z\, pJLB[t] + r3\, r4\, S\, z\, pJLB[t] +$$
$$(-1 + S)\, (-1 + z)\, pQlA[t] - (-1 + S)\, \big(1 - z + r6\, (-1 + y + z)\big)\, pQLA[t] + pQLB[t] - r6\, pQLB[t] - S\, pQLB[t] +$$
$$r6\, S\, pQLB[t] + r6\, y\, pQLB[t] - r6\, S\, y\, pQLB[t] - z\, pQLB[t] + r6\, z\, pQLB[t] + S\, z\, pQLB[t] - r6\, S\, z\, pQLB[t]\Big)\Big) +$$
$$\alpha 1 \Big((-1 + S)\, (-1 + z)\, pQlA[t]^2 + pQlA[t]\, \big(S - S z + pJlA[t] - S\, pJlA[t] - z\, pJlA[t] + S\, z\, pJlA[t] + pJLA[t] - r3$$
$$pJLA[t] - r4\, pJLA[t] + r3\, r4\, pJLA[t] - S\, pJLA[t] + r3\, S\, pJLA[t] + r4\, S\, pJLA[t] - r3\, r4\, S\, pJLA[t] + r3$$
$$r4\, y\, pJLA[t] - r3\, r4\, S\, y\, pJLA[t] - z\, pJLA[t] + r3\, z\, pJLA[t] + r4\, z\, pJLA[t] - r3\, r4\, z\, pJLA[t] + S\, z$$
$$pJLA[t] - r3\, S\, z\, pJLA[t] - r4\, S\, z\, pJLA[t] + r3\, r4\, S\, z\, pJLA[t] + pJlB[t] - r3\, pJlB[t] - r4\, pJlB[t] + 2$$
$$r3\, r4\, pJlB[t] - S\, pJlB[t] + r3\, S\, pJlB[t] + r4\, S\, pJlB[t] - 2\, r3\, r4\, S\, pJlB[t] - z\, pJlB[t] + r3\, z$$
$$pJlB[t] + r4\, z\, pJlB[t] - 2\, r3\, r4\, z\, pJlB[t] + S\, z\, pJlB[t] - r3\, S\, z\, pJlB[t] - r4\, S\, z\, pJlB[t] + 2\, r3\, r4\, S$$
$$z\, pJlB[t] + pJLB[t] - r3\, pJLB[t] - r4\, pJLB[t] + r3\, r4\, pJLB[t] - S\, pJLB[t] + r3\, S\, pJLB[t] + r4\, S$$
$$pJLB[t] - r3\, r4\, S\, pJLB[t] + r3\, r4\, y\, pJLB[t] - r3\, r4\, S\, y\, pJLB[t] - z\, pJLB[t] + r3\, z\, pJLB[t] + r4\, z$$
$$pJLB[t] - r3\, r4\, z\, pJLB[t] + S\, z\, pJLB[t] - r3\, S\, z\, pJLB[t] - r4\, S\, z\, pJLB[t] + r3\, r4\, S\, z\, pJLB[t] - (-1 + S)$$
$$\big(1 - z + r6\, (-1 + y + z)\big)\, pQLA[t] + (-1 + S)\, (-1 + z)\, pQlB[t] + pQLB[t] - r6\, pQLB[t] - S\, pQLB[t] + r6\, S$$
$$pQLB[t] + r6\, y\, pQLB[t] - r6\, S\, y\, pQLB[t] - z\, pQLB[t] + r6\, z\, pQLB[t] + S\, z\, pQLB[t] - r6\, S\, z\, pQLB[t]\big) -$$
$$(-1 + S)\, pJlA[t]\, \Big(\big(-r4 + r3\, (-1 + 2\, r4)\big)\, (-1 + z)\, pQlB[t] - \big(-r4\, y + r3\, (-1 + r4\, (1 + y - z) + z)\big)\, (pQLA[t] + pQLB[t])\Big)\Big)\Big)\Big) \Big/$$
$$\Big(\big(S + pJlA[t] - S\, pJlA[t] + pJLA[t] - S\, pJLA[t] + pJlB[t] - S\, pJlB[t] + pJLB[t] - S\, pJLB[t] - (-1 + S)\, pQlA[t] +$$
$$pQLA[t] - S\, pQLA[t] - (-1 + S)\, pQlB[t] + pQLB[t] - S\, pQLB[t]\big)$$
$$\big(pJlA[t] - k\, \alpha 1\, pJlA[t] + pJLA[t] - k\, \alpha 2\, pJLA[t] + pJlB[t] - k\, pJlB[t] + k\, \beta 1\, pJlB[t] + pJLB[t] -$$
$$k\, pJLB[t] + k\, \beta 2\, pJLB[t] + (1 - k\, \alpha 1)\, pQlA[t] + pQLA[t] - k\, \alpha 2\, pQLA[t] +$$
$$(1 - k + k\, \beta 1)\, pQlB[t] + pQLB[t] - k\, pQLB[t] + k\, \beta 2\, pQLB[t]\big)\Big)\Big);$$

$$pQLA[t + 1] =$$
$$\Big((-1 + S)\, z\, (-1 + \alpha 1)\, pQlA[t]^2 + S\, pQLA[t] - S\, y\, pQLA[t] - S\, \alpha 2\, pQLA[t] + S\, y\, \alpha 2\, pQLA[t] + pJlA[t] \times pQLA[t] - r3\, pJlA[t] \times pQLA[t] -$$
$$S\, pJlA[t] \times pQLA[t] + r3\, S\, pJlA[t] \times pQLA[t] - y\, pJlA[t] \times pQLA[t] + r3\, y\, pJlA[t] \times pQLA[t] + S\, y\, pJlA[t] \times pQLA[t] -$$
$$r3\, S\, y\, pJlA[t] \times pQLA[t] + r3\, z\, pJlA[t] \times pQLA[t] - r3\, S\, z\, pJlA[t] \times pQLA[t] - r4\, \alpha 1\, pJlA[t] \times pQLA[t] + r3\, r4\, \alpha 1\, pJlA[t] \times pQLA[t] +$$
$$r4\, S\, \alpha 1\, pJlA[t] \times pQLA[t] - r3\, r4\, S\, \alpha 1\, pJlA[t] \times pQLA[t] + r4\, y\, \alpha 1\, pJlA[t] \times pQLA[t] - r3\, r4\, y\, \alpha 1\, pJlA[t] \times pQLA[t] -$$
$$r4\, S\, y\, \alpha 1\, pJlA[t] \times pQLA[t] + r3\, r4\, S\, y\, \alpha 1\, pJlA[t] \times pQLA[t] - r3\, z\, \alpha 1\, pJlA[t] \times pQLA[t] + r3\, r4\, z\, \alpha 1\, pJlA[t] \times pQLA[t] +$$
$$r3\, S\, z\, \alpha 1\, pJlA[t] \times pQLA[t] - r3\, r4\, S\, z\, \alpha 1\, pJlA[t] \times pQLA[t] - \alpha 2\, pJlA[t] \times pQLA[t] + r3\, \alpha 2\, pJlA[t] \times pQLA[t] +$$
$$r4\, \alpha 2\, pJlA[t] \times pQLA[t] - r3\, r4\, \alpha 2\, pJlA[t] \times pQLA[t] + S\, \alpha 2\, pJlA[t] \times pQLA[t] - r3\, S\, \alpha 2\, pJlA[t] \times pQLA[t] -$$
$$r4\, S\, \alpha 2\, pJlA[t] \times pQLA[t] + r3\, r4\, S\, \alpha 2\, pJlA[t] \times pQLA[t] + y\, \alpha 2\, pJlA[t] \times pQLA[t] - r3\, y\, \alpha 2\, pJlA[t] \times pQLA[t] -$$
$$r4\, y\, \alpha 2\, pJlA[t] \times pQLA[t] + r3\, r4\, y\, \alpha 2\, pJlA[t] \times pQLA[t] - S\, y\, \alpha 2\, pJlA[t] \times pQLA[t] + r3\, S\, y\, \alpha 2\, pJlA[t] \times pQLA[t] +$$
$$r4\, S\, y\, \alpha 2\, pJlA[t] \times pQLA[t] - r3\, r4\, S\, y\, \alpha 2\, pJlA[t] \times pQLA[t] - r3\, r4\, z\, \alpha 2\, pJlA[t] \times pQLA[t] + r3\, r4\, S\, z\, \alpha 2\, pJlA[t] \times pQLA[t] +$$
$$pJLA[t] \times pQLA[t] - S\, pJLA[t] \times pQLA[t] - y\, pJLA[t] \times pQLA[t] + S\, y\, pJLA[t] \times pQLA[t] - \alpha 2\, pJLA[t] \times pQLA[t] +$$
$$S\, \alpha 2\, pJLA[t] \times pQLA[t] + y\, \alpha 2\, pJLA[t] \times pQLA[t] - S\, y\, \alpha 2\, pJLA[t] \times pQLA[t] + pJlB[t] \times pQLA[t] - r3\, pJlB[t] \times pQLA[t] -$$
$$r4\, pJlB[t] \times pQLA[t] + r3\, r4\, pJlB[t] \times pQLA[t] - S\, pJlB[t] \times pQLA[t] + r3\, S\, pJlB[t] \times pQLA[t] + r4\, S\, pJlB[t] \times pQLA[t] -$$

r3 r4 S pJlB[t] × pQLA[t] − y pJlB[t] × pQLA[t] + r3 y pJlB[t] × pQLA[t] + r4 y pJlB[t] × pQLA[t] − r3 r4 y pJlB[t] × pQLA[t] +
S y pJlB[t] × pQLA[t] − r3 S y pJlB[t] × pQLA[t] − r4 S y pJlB[t] × pQLA[t] + r3 r4 S y pJlB[t] × pQLA[t] + r3 r4 z pJlB[t] × pQLA[t] −
r3 r4 S z pJlB[t] × pQLA[t] − α2 pJlB[t] × pQLA[t] + r3 α2 pJlB[t] × pQLA[t] + r4 α2 pJlB[t] × pQLA[t] − r3 r4 α2 pJlB[t] × pQLA[t] +
S α2 pJlB[t] × pQLA[t] − r3 S α2 pJlB[t] × pQLA[t] − r4 S α2 pJlB[t] × pQLA[t] + r3 r4 S α2 pJlB[t] × pQLA[t] +
y α2 pJlB[t] × pQLA[t] − r3 y α2 pJlB[t] × pQLA[t] − r4 y α2 pJlB[t] × pQLA[t] + r3 r4 y α2 pJlB[t] × pQLA[t] −
S y α2 pJlB[t] × pQLA[t] + r3 S y α2 pJlB[t] × pQLA[t] + r4 S y α2 pJlB[t] × pQLA[t] − r3 r4 S y α2 pJlB[t] × pQLA[t] −
r3 r4 z α2 pJlB[t] × pQLA[t] + r3 r4 S z α2 pJlB[t] × pQLA[t] + pJLB[t] × pQLA[t] − r3 pJLB[t] × pQLA[t] − r4 pJLB[t] × pQLA[t] +
2 r3 r4 pJLB[t] × pQLA[t] − S pJLB[t] × pQLA[t] + r3 S pJLB[t] × pQLA[t] + r4 S pJLB[t] × pQLA[t] − 2 r3 r4 S pJLB[t] × pQLA[t] −
y pJLB[t] × pQLA[t] + r3 y pJLB[t] × pQLA[t] + r4 y pJLB[t] × pQLA[t] − 2 r3 r4 y pJLB[t] × pQLA[t] + S y pJLB[t] × pQLA[t] −
r3 S y pJLB[t] × pQLA[t] − r4 S y pJLB[t] × pQLA[t] + 2 r3 r4 S y pJLB[t] × pQLA[t] − α2 pJLB[t] × pQLA[t] +
r3 α2 pJLB[t] × pQLA[t] + r4 α2 pJLB[t] × pQLA[t] − 2 r3 r4 α2 pJLB[t] × pQLA[t] + S α2 pJLB[t] × pQLA[t] −
r3 S α2 pJLB[t] × pQLA[t] − r4 S α2 pJLB[t] × pQLA[t] + 2 r3 r4 S α2 pJLB[t] × pQLA[t] + y α2 pJLB[t] × pQLA[t] −
r3 y α2 pJLB[t] × pQLA[t] − r4 y α2 pJLB[t] × pQLA[t] + 2 r3 r4 y α2 pJLB[t] × pQLA[t] − S y α2 pJLB[t] × pQLA[t] +
r3 S y α2 pJLB[t] × pQLA[t] + r4 S y α2 pJLB[t] × pQLA[t] − 2 r3 r4 S y α2 pJLB[t] × pQLA[t] + r3 β2 pJLB[t] × pQLA[t] +
r4 β2 pJLB[t] × pQLA[t] − 2 r3 r4 β2 pJLB[t] × pQLA[t] − r3 S β2 pJLB[t] × pQLA[t] − r4 S β2 pJLB[t] × pQLA[t] +
2 r3 r4 S β2 pJLB[t] × pQLA[t] − r3 y β2 pJLB[t] × pQLA[t] − r4 y β2 pJLB[t] × pQLA[t] + 2 r3 r4 y β2 pJLB[t] × pQLA[t] +
r3 S y β2 pJLB[t] × pQLA[t] + r4 S y β2 pJLB[t] × pQLA[t] − 2 r3 r4 S y β2 pJLB[t] × pQLA[t] + pQLA[t]^2 − S pQLA[t]^2 −
y pQLA[t]^2 + S y pQLA[t]^2 − α2 pQLA[t]^2 + S α2 pQLA[t]^2 + y α2 pQLA[t]^2 − S y α2 pQLA[t]^2 + r3 z pJlA[t] × pQlB[t] +
r4 z pJlA[t] × pQlB[t] − 2 r3 r4 z pJlA[t] × pQlB[t] − r3 S z pJlA[t] × pQlB[t] − r4 S z pJlA[t] × pQlB[t] +
2 r3 r4 S z pJlA[t] × pQlB[t] − r3 z α1 pJlA[t] × pQlB[t] − r4 z α1 pJlA[t] × pQlB[t] + 2 r3 r4 z α1 pJlA[t] × pQlB[t] +
r3 S z α1 pJlA[t] × pQlB[t] + r4 S z α1 pJlA[t] × pQlB[t] − 2 r3 r4 S z α1 pJlA[t] × pQlB[t] + r3 pJLA[t] × pQlB[t] −
r3 r4 pJLA[t] × pQlB[t] − r3 S pJLA[t] × pQlB[t] + r3 r4 S pJLA[t] × pQlB[t] − r3 y pJLA[t] × pQlB[t] + r3 r4 y pJLA[t] × pQlB[t] +
r3 S y pJLA[t] × pQlB[t] − r3 r4 S y pJLA[t] × pQlB[t] + r4 z pJLA[t] × pQlB[t] − r3 r4 z pJLA[t] × pQlB[t] −
r4 S z pJLA[t] × pQlB[t] + r3 r4 S z pJLA[t] × pQlB[t] − r3 α2 pJLA[t] × pQlB[t] + r3 r4 α2 pJLA[t] × pQlB[t] +
r3 S α2 pJLA[t] × pQlB[t] − r3 r4 S α2 pJLA[t] × pQlB[t] + r3 y α2 pJLA[t] × pQlB[t] − r3 r4 y α2 pJLA[t] × pQlB[t] −
r3 S y α2 pJLA[t] × pQlB[t] + r3 r4 S y α2 pJLA[t] × pQlB[t] − r4 z α2 pJLA[t] × pQlB[t] + r3 r4 z α2 pJLA[t] × pQlB[t] +
r4 S z α2 pJLA[t] × pQlB[t] − r3 r4 S z α2 pJLA[t] × pQlB[t] + r3 β2 pJLB[t] × pQlB[t] − r3 r4 β2 pJLB[t] × pQlB[t] −
r3 S β2 pJLB[t] × pQlB[t] + r3 r4 S β2 pJLB[t] × pQlB[t] − r3 y β2 pJLB[t] × pQlB[t] + r3 r4 y β2 pJLB[t] × pQlB[t] +
r3 S y β2 pJLB[t] × pQlB[t] − r3 r4 S y β2 pJLB[t] × pQlB[t] + r4 z β2 pJLB[t] × pQlB[t] − r3 r4 z β2 pJLB[t] × pQlB[t] −
r4 S z β2 pJLB[t] × pQlB[t] + r3 r4 S z β2 pJLB[t] × pQlB[t] + pQLA[t] × pQlB[t] − r6 pQLA[t] × pQlB[t] − S pQLA[t] × pQlB[t] +
r6 S pQLA[t] × pQlB[t] − y pQLA[t] × pQlB[t] + r6 y pQLA[t] × pQlB[t] + S y pQLA[t] × pQlB[t] − r6 S y pQLA[t] × pQlB[t] +
r6 z pQLA[t] × pQlB[t] − r6 S z pQLA[t] × pQlB[t] − α2 pQLA[t] × pQlB[t] + r6 α2 pQLA[t] × pQlB[t] + S α2 pQLA[t] × pQlB[t] −
r6 S α2 pQLA[t] × pQlB[t] + y α2 pQLA[t] × pQlB[t] − r6 y α2 pQLA[t] × pQlB[t] − S y α2 pQLA[t] × pQlB[t] +
r6 S y α2 pQLA[t] × pQlB[t] − r6 z α2 pQLA[t] × pQlB[t] + r6 S z α2 pQLA[t] × pQlB[t] + S β2 pQLB[t] − S y β2 pQLB[t] +
r4 pJlA[t] × pQLB[t] − r3 r4 pJlA[t] × pQLB[t] − r4 S pJlA[t] × pQLB[t] + r3 r4 S pJlA[t] × pQLB[t] − r4 y pJlA[t] × pQLB[t] +
r3 r4 y pJlA[t] × pQLB[t] + r4 S y pJlA[t] × pQLB[t] − r3 r4 S y pJlA[t] × pQLB[t] + r3 z pJlA[t] × pQLB[t] −
r3 r4 z pJlA[t] × pQLB[t] − r3 S z pJlA[t] × pQLB[t] + r3 r4 S z pJlA[t] × pQLB[t] − r4 α1 pJlA[t] × pQLB[t] +
r3 r4 α1 pJlA[t] × pQLB[t] + r4 S α1 pJlA[t] × pQLB[t] − r3 r4 S α1 pJlA[t] × pQLB[t] + r4 y α1 pJlA[t] × pQLB[t] −
r3 r4 y α1 pJlA[t] × pQLB[t] − r4 S y α1 pJlA[t] × pQLB[t] + r3 r4 S y α1 pJlA[t] × pQLB[t] − r3 z α1 pJlA[t] × pQLB[t] +
r3 r4 z α1 pJlA[t] × pQLB[t] + r3 S z α1 pJlA[t] × pQLB[t] − r3 r4 S z α1 pJlA[t] × pQLB[t] + β2 pJlA[t] × pQLB[t] −
r3 β2 pJlA[t] × pQLB[t] − r4 β2 pJlA[t] × pQLB[t] + r3 r4 β2 pJlA[t] × pQLB[t] − S β2 pJlA[t] × pQLB[t] +
r3 S β2 pJlA[t] × pQLB[t] + r4 S β2 pJlA[t] × pQLB[t] − r3 r4 S β2 pJlA[t] × pQLB[t] − y β2 pJlA[t] × pQLB[t] +
r3 y β2 pJlA[t] × pQLB[t] + r4 y β2 pJlA[t] × pQLB[t] − r3 r4 y β2 pJlA[t] × pQLB[t] + S y β2 pJlA[t] × pQLB[t] −
r3 S y β2 pJlA[t] × pQLB[t] − r4 S y β2 pJlA[t] × pQLB[t] + r3 r4 S y β2 pJlA[t] × pQLB[t] + r3 r4 z β2 pJlA[t] × pQLB[t] −
r3 r4 S z β2 pJlA[t] × pQLB[t] + r3 pJLA[t] × pQLB[t] + r4 pJLA[t] × pQLB[t] − 2 r3 r4 pJLA[t] × pQLB[t] −

r3 S pJLA[t] × pQLB[t] – r4 S pJLA[t] × pQLB[t] + 2 r3 r4 S pJLA[t] × pQLB[t] – r3 y pJLA[t] × pQLB[t] –
r4 y pJLA[t] × pQLB[t] + 2 r3 r4 y pJLA[t] × pQLB[t] + r3 S y pJLA[t] × pQLB[t] + r4 S y pJLA[t] × pQLB[t] –
2 r3 r4 S y pJLA[t] × pQLB[t] – r3 α2 pJLA[t] × pQLB[t] – r4 α2 pJLA[t] × pQLB[t] + 2 r3 r4 α2 pJLA[t] × pQLB[t] +
r3 S α2 pJLA[t] × pQLB[t] + r4 S α2 pJLA[t] × pQLB[t] – 2 r3 r4 S α2 pJLA[t] × pQLB[t] + r3 y α2 pJLA[t] × pQLB[t] +
r4 y α2 pJLA[t] × pQLB[t] – 2 r3 r4 y α2 pJLA[t] × pQLB[t] – r3 S y α2 pJLA[t] × pQLB[t] – r4 S y α2 pJLA[t] × pQLB[t] +
2 r3 r4 S y α2 pJLA[t] × pQLB[t] + β2 pJLA[t] × pQLB[t] – r3 β2 pJLA[t] × pQLB[t] – r4 β2 pJLA[t] × pQLB[t] +
2 r3 r4 β2 pJLA[t] × pQLB[t] – S β2 pJLA[t] × pQLB[t] + r3 S β2 pJLA[t] × pQLB[t] + r4 S β2 pJLA[t] × pQLB[t] –
2 r3 r4 S β2 pJLA[t] × pQLB[t] – y β2 pJLA[t] × pQLB[t] + r3 y β2 pJLA[t] × pQLB[t] + r4 y β2 pJLA[t] × pQLB[t] –
2 r3 r4 y β2 pJLA[t] × pQLB[t] + S y β2 pJLA[t] × pQLB[t] – r3 S y β2 pJLA[t] × pQLB[t] – r4 S y β2 pJLA[t] × pQLB[t] +
2 r3 r4 S y β2 pJLA[t] × pQLB[t] + β2 pJlB[t] × pQLB[t] – r3 β2 pJlB[t] × pQLB[t] – r4 β2 pJlB[t] × pQLB[t] +
r3 r4 β2 pJlB[t] × pQLB[t] – S β2 pJlB[t] × pQLB[t] + r3 S β2 pJlB[t] × pQLB[t] + r4 S β2 pJlB[t] × pQLB[t] –
r3 r4 S β2 pJlB[t] × pQLB[t] – y β2 pJlB[t] × pQLB[t] + r3 y β2 pJlB[t] × pQLB[t] + r4 y β2 pJlB[t] × pQLB[t] –
r3 r4 y β2 pJlB[t] × pQLB[t] + S y β2 pJlB[t] × pQLB[t] – r3 S y β2 pJlB[t] × pQLB[t] – r4 S y β2 pJlB[t] × pQLB[t] +
r3 r4 S y β2 pJlB[t] × pQLB[t] + r3 r4 z β2 pJlB[t] × pQLB[t] – r3 r4 S z β2 pJlB[t] × pQLB[t] + β2 pJLB[t] × pQLB[t] –
S β2 pJLB[t] × pQLB[t] – y β2 pJLB[t] × pQLB[t] + S y β2 pJLB[t] × pQLB[t] + pQLA[t] × pQLB[t] – S pQLA[t] × pQLB[t] –
y pQLA[t] × pQLB[t] + S y pQLA[t] × pQLB[t] – α2 pQLA[t] × pQLB[t] + S α2 pQLA[t] × pQLB[t] + y α2 pQLA[t] × pQLB[t] –
S y α2 pQLA[t] × pQLB[t] + β2 pQLA[t] × pQLB[t] – S β2 pQLA[t] × pQLB[t] – y β2 pQLA[t] × pQLB[t] +
S y β2 pQLA[t] × pQLB[t] + β2 pQlB[t] × pQLB[t] – r6 β2 pQlB[t] × pQLB[t] – S β2 pQlB[t] × pQLB[t] + r6 S β2 pQlB[t] × pQLB[t] –
y β2 pQlB[t] × pQLB[t] + r6 y β2 pQlB[t] × pQLB[t] + S y β2 pQlB[t] × pQLB[t] – r6 S y β2 pQlB[t] × pQLB[t] +
r6 z β2 pQlB[t] × pQLB[t] – r6 S z β2 pQlB[t] × pQLB[t] + β2 pQLB[t]^2 – S β2 pQLB[t]^2 – y β2 pQLB[t]^2 + S y β2 pQLB[t]^2 +
pQlA[t] (S z + z pJlA[t] – S z pJlA[t] + r3 pJLA[t] – r3 S pJLA[t] – r3 y pJLA[t] + r3 S y pJLA[t] + z pJLA[t] – r3 z pJLA[t] –
S z pJLA[t] + r3 S z pJLA[t] – r3 α2 pJLA[t] + r3 r4 α2 pJLA[t] + r3 S α2 pJLA[t] – r3 r4 S α2 pJLA[t] + r3 y α2 pJLA[t] –
r3 r4 y α2 pJLA[t] – r3 S y α2 pJLA[t] + r3 r4 S y α2 pJLA[t] – r4 z α2 pJLA[t] + r3 r4 z α2 pJLA[t] + r4 S z α2 pJLA[t] –
r3 r4 S z α2 pJLA[t] + z pJlB[t] – r3 z pJlB[t] – r4 z pJlB[t] + 2 r3 r4 z pJlB[t] – S z pJlB[t] + r3 S z pJlB[t] + r4 S z pJlB[t] –
2 r3 r4 S z pJlB[t] + r3 z β1 pJlB[t] + r4 z β1 pJlB[t] – 2 r3 r4 z β1 pJlB[t] – r3 S z β1 pJlB[t] – r4 S z β1 pJlB[t] +
2 r3 r4 S z β1 pJlB[t] + r3 r4 pJLB[t] – r3 r4 S pJLB[t] – r3 r4 y pJLB[t] + r3 r4 S y pJLB[t] + z pJLB[t] – r3 z pJLB[t] –
r4 z pJLB[t] + r3 r4 z pJLB[t] – S z pJLB[t] + r3 S z pJLB[t] + r4 S z pJLB[t] – r3 r4 S z pJLB[t] + r3 β2 pJLB[t] –
r3 r4 β2 pJLB[t] – r3 S β2 pJLB[t] + r3 r4 S β2 pJLB[t] – r3 y β2 pJLB[t] + r3 r4 y β2 pJLB[t] + r3 S y β2 pJLB[t] –
r3 r4 S y β2 pJLB[t] + r4 z β2 pJLB[t] – r3 r4 z β2 pJLB[t] – r4 S z β2 pJLB[t] + r3 r4 S z β2 pJLB[t] + pQLA[t] –
S pQLA[t] – y pQLA[t] + S y pQLA[t] + z pQLA[t] – S z pQLA[t] – α2 pQLA[t] + r6 α2 pQLA[t] + S α2 pQLA[t] –
r6 S α2 pQLA[t] + y α2 pQLA[t] – r6 y α2 pQLA[t] – S y α2 pQLA[t] + r6 S y α2 pQLA[t] – r6 z α2 pQLA[t] +
r6 S z α2 pQLA[t] + z pQlB[t] – S z pQlB[t] + z β1 pQlB[t] – S z β1 pQlB[t] + r6 pQLB[t] – r6 S pQLB[t] – r6 y pQLB[t] +
r6 S y pQLB[t] + z pQLB[t] – r6 z pQLB[t] – S z pQLB[t] + r6 S z pQLB[t] + β2 pQLB[t] – r6 β2 pQLB[t] – S β2 pQLB[t] +
r6 S β2 pQLB[t] – y β2 pQLB[t] + r6 y β2 pQLB[t] + S y β2 pQLB[t] – r6 S y β2 pQLB[t] + r6 z β2 pQLB[t] –
r6 S z β2 pQLB[t] + α1 (–S z – z pJlA[t] + S z pJlA[t] – r3 r4 pJLA[t] + r3 r4 S pJLA[t] + r3 r4 y pJLA[t] –
r3 r4 S y pJLA[t] – z pJLA[t] + r3 z pJLA[t] + r4 z pJLA[t] – r3 r4 z pJLA[t] + S z pJLA[t] – r3 S z pJLA[t] –
r4 S z pJLA[t] + r3 r4 S z pJLA[t] – z pJlB[t] + r3 z pJlB[t] + r4 z pJlB[t] – 2 r3 r4 z pJlB[t] + S z pJlB[t] –
r3 S z pJlB[t] – r4 S z pJlB[t] + 2 r3 r4 S z pJlB[t] – r3 r4 pJLB[t] + r3 r4 S pJLB[t] + r3 r4 y pJLB[t] –
r3 r4 S y pJLB[t] – z pJLB[t] + r3 z pJLB[t] + r4 z pJLB[t] – r3 r4 z pJLB[t] + S z pJLB[t] – r3 S z pJLB[t] –
r4 S z pJLB[t] + r3 r4 S z pJLB[t] – (–1 + S) (–z + r6 (–1 + y + z)) pQLA[t] + (–1 + S) z pQlB[t] – r6 pQLB[t] +
r6 S pQLB[t] + r6 y pQLB[t] – r6 S y pQLB[t] – z pQLB[t] + r6 z pQLB[t] + S z pQLB[t] – r6 S z pQLB[t])) +
β1 ((z – S z) pQlB[t]^2 – (–1 + S) (r4 – r4 y + r3 r4 (–1 + y – z) + r3 z) pJlB[t] (pQLA[t] + pQLB[t]) +
pQlB[t] (S z + z pJlA[t] – r3 z pJlA[t] – r4 z pJlA[t] + 2 r3 r4 z pJlA[t] – S z pJlA[t] + r3 S z pJlA[t] + r4 S z pJlA[t] –
2 r3 r4 S z pJlA[t] + r3 r4 pJLA[t] – r3 r4 S pJLA[t] – r3 r4 y pJLA[t] + r3 r4 S y pJLA[t] + z pJLA[t] –

```
r3 z pJLA[t] - r4 z pJLA[t] + r3 r4 z pJLA[t] - S z pJLA[t] + r3 S z pJLA[t] + r4 S z pJLA[t] - r3 r4 S z pJLA[t] +
z pJlB[t] - S z pJlB[t] + r3 r4 pJLB[t] - r3 r4 S pJLB[t] - r3 r4 y pJLB[t] + r3 r4 S y pJLB[t] +
z pJLB[t] - r3 z pJLB[t] - r4 z pJLB[t] + r3 r4 z pJLB[t] - S z pJLB[t] + r3 S z pJLB[t] + r4 S z pJLB[t] -
r3 r4 S z pJLB[t] + (-1 + S) (-z + r6 (-1 + y + z)) pQLA[t] + (-1 + S) (-z + r6 (-1 + y + z)) pQLB[t]))) /
((S + pJlA[t] - S pJlA[t] + pJLA[t] - S pJLA[t] + pJlB[t] - S pJlB[t] + pJLB[t] - S pJLB[t] - (-1 + S) pQlA[t] +
pQLA[t] - S pQLA[t] - (-1 + S) pQlB[t] + pQLB[t] - S pQLB[t])
(pJlA[t] - k α1 pJlA[t] + pJLA[t] - k α2 pJLA[t] + pJlB[t] - k pJlB[t] + k β1 pJlB[t] + pJLB[t] - k pJLB[t] + k β2 pJLB[t] +
(1 - k α1) pQlA[t] + pQLA[t] - k α2 pQLA[t] + (1 - k + k β1) pQlB[t] + pQLB[t] - k pQLB[t] + k β2 pQLB[t]));


pQLB[t + 1] =
((-1 + k) (-r3 α2 pJLA[t] × pQlA[t] + r3 r4 α2 pJLA[t] × pQlA[t] + r3 S α2 pJLA[t] × pQlA[t] - r3 r4 S α2 pJLA[t] × pQlA[t] + r3 y α2
pJLA[t] × pQlA[t] - r3 r4 y α2 pJLA[t] × pQlA[t] - r3 S y α2 pJLA[t] × pQlA[t] + r3 r4 S y α2 pJLA[t] × pQlA[t] -
r4 z α2 pJLA[t] × pQlA[t] + r3 r4 z α2 pJLA[t] × pQlA[t] + r4 S z α2 pJLA[t] × pQlA[t] - r3 r4 S z α2 pJLA[t] × pQlA[t] -
r3 z pJlB[t] × pQlA[t] - r4 z pJlB[t] × pQlA[t] + 2 r3 r4 z pJlB[t] × pQlA[t] + r3 S z pJlB[t] × pQlA[t] + r4 S z pJlB[t] × pQlA[t] -
2 r3 r4 S z pJlB[t] × pQlA[t] + r3 z β1 pJlB[t] × pQlA[t] + r4 z β1 pJlB[t] × pQlA[t] - 2 r3 r4 z β1 pJlB[t] × pQlA[t] -
r3 S z β1 pJlB[t] × pQlA[t] - r4 S z β1 pJlB[t] × pQlA[t] + 2 r3 r4 S z β1 pJlB[t] × pQlA[t] - r3 pJLB[t] × pQlA[t] +
r3 r4 pJLB[t] × pQlA[t] + r3 S pJLB[t] × pQlA[t] - r3 r4 S pJLB[t] × pQlA[t] + r3 y pJLB[t] × pQlA[t] -
r3 r4 y pJLB[t] × pQlA[t] - r3 S y pJLB[t] × pQlA[t] + r3 r4 S y pJLB[t] × pQlA[t] - r4 z pJLB[t] × pQlA[t] +
r3 r4 z pJLB[t] × pQlA[t] + r4 S z pJLB[t] × pQlA[t] - r3 r4 S z pJLB[t] × pQlA[t] + r3 β2 pJLB[t] × pQlA[t] -
r3 r4 β2 pJLB[t] × pQlA[t] - r3 S β2 pJLB[t] × pQlA[t] + r3 r4 S β2 pJLB[t] × pQlA[t] - r3 y β2 pJLB[t] × pQlA[t] +
r3 r4 y β2 pJLB[t] × pQlA[t] + r3 S y β2 pJLB[t] × pQlA[t] - r3 r4 S y β2 pJLB[t] × pQlA[t] + r4 z β2 pJLB[t] × pQlA[t] -
r3 r4 z β2 pJLB[t] × pQlA[t] - r4 S z β2 pJLB[t] × pQlA[t] + r3 r4 S z β2 pJLB[t] × pQlA[t] - S α2 pQLA[t] +
S y α2 pQLA[t] - α2 pJlA[t] × pQLA[t] + r3 α2 pJlA[t] × pQLA[t] + r4 α2 pJlA[t] × pQLA[t] - r3 r4 α2 pJlA[t] × pQLA[t] +
S α2 pJlA[t] × pQLA[t] - r3 S α2 pJlA[t] × pQLA[t] - r4 S α2 pJlA[t] × pQLA[t] + r3 r4 S α2 pJlA[t] × pQLA[t] +
y α2 pJlA[t] × pQLA[t] - r3 y α2 pJlA[t] × pQLA[t] - r4 y α2 pJlA[t] × pQLA[t] + r3 r4 y α2 pJlA[t] × pQLA[t] -
S y α2 pJlA[t] × pQLA[t] + r3 S y α2 pJlA[t] × pQLA[t] + r4 S y α2 pJlA[t] × pQLA[t] - r3 r4 S y α2 pJlA[t] × pQLA[t] -
r3 r4 z α2 pJlA[t] × pQLA[t] + r3 r4 S z α2 pJlA[t] × pQLA[t] - α2 pJLA[t] × pQLA[t] + S α2 pJLA[t] × pQLA[t] +
y α2 pJLA[t] × pQLA[t] - S y α2 pJLA[t] × pQLA[t] - r4 pJlB[t] × pQLA[t] + r3 r4 pJlB[t] × pQLA[t] + r4 S pJlB[t] × pQLA[t] -
r3 r4 S pJlB[t] × pQLA[t] + r4 y pJlB[t] × pQLA[t] - r3 r4 y pJlB[t] × pQLA[t] - r4 S y pJlB[t] × pQLA[t] +
r3 r4 S y pJlB[t] × pQLA[t] - r3 z pJlB[t] × pQLA[t] + r3 r4 z pJlB[t] × pQLA[t] + r3 S z pJlB[t] × pQLA[t] -
r3 r4 S z pJlB[t] × pQLA[t] - α2 pJlB[t] × pQLA[t] + r3 α2 pJlB[t] × pQLA[t] + r4 α2 pJlB[t] × pQLA[t] -
r3 r4 α2 pJlB[t] × pQLA[t] + S α2 pJlB[t] × pQLA[t] - r3 S α2 pJlB[t] × pQLA[t] - r4 S α2 pJlB[t] × pQLA[t] +
r3 r4 S α2 pJlB[t] × pQLA[t] + y α2 pJlB[t] × pQLA[t] - r3 y α2 pJlB[t] × pQLA[t] - r4 y α2 pJlB[t] × pQLA[t] +
r3 r4 y α2 pJlB[t] × pQLA[t] - S y α2 pJlB[t] × pQLA[t] + r3 S y α2 pJlB[t] × pQLA[t] + r4 S y α2 pJlB[t] × pQLA[t] -
r3 r4 S y α2 pJlB[t] × pQLA[t] - r3 r4 z α2 pJlB[t] × pQLA[t] + r3 r4 S z α2 pJlB[t] × pQLA[t] + r4 β1 pJlB[t] × pQLA[t] -
r3 r4 β1 pJlB[t] × pQLA[t] - r4 S β1 pJlB[t] × pQLA[t] + r3 r4 S β1 pJlB[t] × pQLA[t] - r4 y β1 pJlB[t] × pQLA[t] +
r3 r4 y β1 pJlB[t] × pQLA[t] + r4 S y β1 pJlB[t] × pQLA[t] - r3 r4 S y β1 pJlB[t] × pQLA[t] + r3 z β1 pJlB[t] × pQLA[t] -
r3 r4 z β1 pJlB[t] × pQLA[t] - r3 S z β1 pJlB[t] × pQLA[t] + r3 r4 S z β1 pJlB[t] × pQLA[t] - r3 pJLB[t] × pQLA[t] -
r4 pJLB[t] × pQLA[t] + 2 r3 r4 pJLB[t] × pQLA[t] + r3 S pJLB[t] × pQLA[t] + r4 S pJLB[t] × pQLA[t] -
2 r3 r4 S pJLB[t] × pQLA[t] + r3 y pJLB[t] × pQLA[t] + r4 y pJLB[t] × pQLA[t] - 2 r3 r4 y pJLB[t] × pQLA[t] -
r3 S y pJLB[t] × pQLA[t] - r4 S y pJLB[t] × pQLA[t] + 2 r3 r4 S y pJLB[t] × pQLA[t] - α2 pJLB[t] × pQLA[t] +
r3 α2 pJLB[t] × pQLA[t] + r4 α2 pJLB[t] × pQLA[t] - 2 r3 r4 α2 pJLB[t] × pQLA[t] + S α2 pJLB[t] × pQLA[t] -
r3 S α2 pJLB[t] × pQLA[t] - r4 S α2 pJLB[t] × pQLA[t] + 2 r3 r4 S α2 pJLB[t] × pQLA[t] + y α2 pJLB[t] × pQLA[t] -
```

r3 y α2 pJLB[t] × pQLA[t] – r4 y α2 pJLB[t] × pQLA[t] + 2 r3 r4 y α2 pJLB[t] × pQLA[t] – S y α2 pJLB[t] × pQLA[t] +
r3 S y α2 pJLB[t] × pQLA[t] + r4 S y α2 pJLB[t] × pQLA[t] – 2 r3 r4 S y α2 pJLB[t] × pQLA[t] + r3 β2 pJLB[t] × pQLA[t] +
r4 β2 pJLB[t] × pQLA[t] – 2 r3 r4 β2 pJLB[t] × pQLA[t] – r3 S β2 pJLB[t] × pQLA[t] – r4 S β2 pJLB[t] × pQLA[t] +
2 r3 r4 S β2 pJLB[t] × pQLA[t] – r3 y β2 pJLB[t] × pQLA[t] – r4 y β2 pJLB[t] × pQLA[t] + 2 r3 r4 y β2 pJLB[t] × pQLA[t] +
r3 S y β2 pJLB[t] × pQLA[t] + r4 S y β2 pJLB[t] × pQLA[t] – 2 r3 r4 S y β2 pJLB[t] × pQLA[t] – α2 pQlA[t] × pQLA[t] +
r6 α2 pQlA[t] × pQLA[t] + S α2 pQlA[t] × pQLA[t] – r6 S α2 pQlA[t] × pQLA[t] + y α2 pQlA[t] × pQLA[t] –
r6 y α2 pQlA[t] × pQLA[t] – S y α2 pQlA[t] × pQLA[t] + r6 S y α2 pQlA[t] × pQLA[t] – r6 z α2 pQlA[t] × pQLA[t] +
r6 S z α2 pQlA[t] × pQLA[t] – α2 pQLA[t]^2 + S α2 pQLA[t]^2 + y α2 pQLA[t]^2 – S y α2 pQLA[t]^2 – (–1 + S) z (–1 + β1) pQlB[t]^2 –
S pQLB[t] + S y pQLB[t] + S β2 pQLB[t] – S y β2 pQLB[t] – pJlA[t] × pQLB[t] + r3 pJlA[t] × pQLB[t] + r4 pJlA[t] × pQLB[t] –
r3 r4 pJlA[t] × pQLB[t] + S pJlA[t] × pQLB[t] – r3 S pJlA[t] × pQLB[t] – r4 S pJlA[t] × pQLB[t] + r3 r4 S pJlA[t] × pQLB[t] +
y pJlA[t] × pQLB[t] – r3 y pJlA[t] × pQLB[t] – r4 y pJlA[t] × pQLB[t] + r3 r4 y pJlA[t] × pQLB[t] – S y pJlA[t] × pQLB[t] +
r3 S y pJlA[t] × pQLB[t] + r4 S y pJlA[t] × pQLB[t] – r3 r4 S y pJlA[t] × pQLB[t] – r3 r4 z pJlA[t] × pQLB[t] +
r3 r4 S z pJlA[t] × pQLB[t] + β2 pJlA[t] × pQLB[t] – r3 β2 pJlA[t] × pQLB[t] – r4 β2 pJlA[t] × pQLB[t] +
r3 r4 β2 pJlA[t] × pQLB[t] – S β2 pJlA[t] × pQLB[t] + r3 S β2 pJlA[t] × pQLB[t] + r4 S β2 pJlA[t] × pQLB[t] –
r3 r4 S β2 pJlA[t] × pQLB[t] – y β2 pJlA[t] × pQLB[t] + r3 y β2 pJlA[t] × pQLB[t] + r4 y β2 pJlA[t] × pQLB[t] –
r3 r4 y β2 pJlA[t] × pQLB[t] + S y β2 pJlA[t] × pQLB[t] – r3 S y β2 pJlA[t] × pQLB[t] – r4 S y β2 pJlA[t] × pQLB[t] +
r3 r4 S y β2 pJlA[t] × pQLB[t] + r3 r4 z β2 pJlA[t] × pQLB[t] – r3 r4 S z β2 pJlA[t] × pQLB[t] – pJLA[t] × pQLB[t] +
r3 pJLA[t] × pQLB[t] + r4 pJLA[t] × pQLB[t] – 2 r3 r4 pJLA[t] × pQLB[t] + S pJLA[t] × pQLB[t] – r3 S pJLA[t] × pQLB[t] –
r4 S pJLA[t] × pQLB[t] + 2 r3 r4 S pJLA[t] × pQLB[t] + y pJLA[t] × pQLB[t] – r3 y pJLA[t] × pQLB[t] –
r4 y pJLA[t] × pQLB[t] + 2 r3 r4 y pJLA[t] × pQLB[t] – S y pJLA[t] × pQLB[t] + r3 S y pJLA[t] × pQLB[t] +
r4 S y pJLA[t] × pQLB[t] – 2 r3 r4 S y pJLA[t] × pQLB[t] – r3 α2 pJLA[t] × pQLB[t] – r4 α2 pJLA[t] × pQLB[t] +
2 r3 r4 α2 pJLA[t] × pQLB[t] + r3 S α2 pJLA[t] × pQLB[t] + r4 S α2 pJLA[t] × pQLB[t] – 2 r3 r4 S α2 pJLA[t] × pQLB[t] +
r3 y α2 pJLA[t] × pQLB[t] + r4 y α2 pJLA[t] × pQLB[t] – 2 r3 r4 y α2 pJLA[t] × pQLB[t] – r3 S y α2 pJLA[t] × pQLB[t] –
r4 S y α2 pJLA[t] × pQLB[t] + 2 r3 r4 S y α2 pJLA[t] × pQLB[t] + β2 pJLA[t] × pQLB[t] – r3 β2 pJLA[t] × pQLB[t] –
r4 β2 pJLA[t] × pQLB[t] + 2 r3 r4 β2 pJLA[t] × pQLB[t] – S β2 pJLA[t] × pQLB[t] + r3 S β2 pJLA[t] × pQLB[t] +
r4 S β2 pJLA[t] × pQLB[t] – 2 r3 r4 S β2 pJLA[t] × pQLB[t] – y β2 pJLA[t] × pQLB[t] + r3 y β2 pJLA[t] × pQLB[t] +
r4 y β2 pJLA[t] × pQLB[t] – 2 r3 r4 y β2 pJLA[t] × pQLB[t] + S y β2 pJLA[t] × pQLB[t] – r3 S y β2 pJLA[t] × pQLB[t] –
r4 S y β2 pJLA[t] × pQLB[t] + 2 r3 r4 S y β2 pJLA[t] × pQLB[t] – pJlB[t] × pQLB[t] + r3 pJlB[t] × pQLB[t] +
S pJlB[t] × pQLB[t] – r3 S pJlB[t] × pQLB[t] + y pJlB[t] × pQLB[t] – r3 y pJlB[t] × pQLB[t] – S y pJlB[t] × pQLB[t] +
r3 S y pJlB[t] × pQLB[t] – r3 z pJlB[t] × pQLB[t] + r3 S z pJlB[t] × pQLB[t] + r4 β1 pJlB[t] × pQLB[t] –
r3 r4 β1 pJlB[t] × pQLB[t] – r4 S β1 pJlB[t] × pQLB[t] + r3 r4 S β1 pJlB[t] × pQLB[t] – r4 y β1 pJlB[t] × pQLB[t] +
r3 r4 y β1 pJlB[t] × pQLB[t] + r4 S y β1 pJlB[t] × pQLB[t] – r3 r4 S y β1 pJlB[t] × pQLB[t] + r3 z β1 pJlB[t] × pQLB[t] –
r3 r4 z β1 pJlB[t] × pQLB[t] – r3 S z β1 pJlB[t] × pQLB[t] + r3 r4 S z β1 pJlB[t] × pQLB[t] + β2 pJlB[t] × pQLB[t] –
r3 β2 pJlB[t] × pQLB[t] – r4 β2 pJlB[t] × pQLB[t] + r3 r4 β2 pJlB[t] × pQLB[t] – S β2 pJlB[t] × pQLB[t] +
r3 S β2 pJlB[t] × pQLB[t] + r4 S β2 pJlB[t] × pQLB[t] – r3 r4 S β2 pJlB[t] × pQLB[t] – y β2 pJlB[t] × pQLB[t] +
r3 y β2 pJlB[t] × pQLB[t] + r4 y β2 pJlB[t] × pQLB[t] – r3 r4 y β2 pJlB[t] × pQLB[t] + S y β2 pJlB[t] × pQLB[t] –
r3 S y β2 pJlB[t] × pQLB[t] – r4 S y β2 pJlB[t] × pQLB[t] + r3 r4 S y β2 pJlB[t] × pQLB[t] + r3 r4 z β2 pJlB[t] × pQLB[t] –
r3 r4 S z β2 pJlB[t] × pQLB[t] – pJLB[t] × pQLB[t] + S pJLB[t] × pQLB[t] + y pJLB[t] × pQLB[t] – S y pJLB[t] × pQLB[t] +
β2 pJLB[t] × pQLB[t] – S β2 pJLB[t] × pQLB[t] – y β2 pJLB[t] × pQLB[t] + S y β2 pJLB[t] × pQLB[t] – pQlA[t] × pQLB[t] +
r6 pQlA[t] × pQLB[t] + S pQlA[t] × pQLB[t] – r6 S pQlA[t] × pQLB[t] + y pQlA[t] × pQLB[t] – r6 y pQlA[t] × pQLB[t] –
S y pQlA[t] × pQLB[t] + r6 S y pQlA[t] × pQLB[t] – r6 z pQlA[t] × pQLB[t] + r6 S z pQlA[t] × pQLB[t] + β2 pQlA[t] × pQLB[t] –
r6 β2 pQlA[t] × pQLB[t] – S β2 pQlA[t] × pQLB[t] + r6 S β2 pQlA[t] × pQLB[t] – y β2 pQlA[t] × pQLB[t] +
r6 y β2 pQlA[t] × pQLB[t] + S y β2 pQlA[t] × pQLB[t] – r6 S y β2 pQlA[t] × pQLB[t] + r6 z β2 pQlA[t] × pQLB[t] –
r6 S z β2 pQlA[t] × pQLB[t] – pQLA[t] × pQLB[t] + S pQLA[t] × pQLB[t] + y pQLA[t] × pQLB[t] – S y pQLA[t] × pQLB[t] –
α2 pQLA[t] × pQLB[t] + S α2 pQLA[t] × pQLB[t] + y α2 pQLA[t] × pQLB[t] – S y α2 pQLA[t] × pQLB[t] +

```
β2 pQLA[t] × pQLB[t] - S β2 pQLA[t] × pQLB[t] - y β2 pQLA[t] × pQLB[t] + S y β2 pQLA[t] × pQLB[t] - pQLB[t]^2 +
S pQLB[t]^2 + y pQLB[t]^2 - S y pQLB[t]^2 + β2 pQLB[t]^2 - S β2 pQLB[t]^2 - y β2 pQLB[t]^2 + S y β2 pQLB[t]^2 +
pQlB[t] (-S z - z pJlA[t] + r3 z pJlA[t] + r4 z pJlA[t] - 2 r3 r4 z pJlA[t] + S z pJlA[t] - r3 S z pJlA[t] - r4 S z pJlA[t] +
    2 r3 r4 S z pJlA[t] - r3 r4 pJLA[t] + r3 r4 S pJLA[t] + r3 r4 y pJLA[t] - r3 r4 S y pJLA[t] - z pJLA[t] + r3 z pJLA[t] +
    r4 z pJLA[t] - r3 r4 z pJLA[t] + S z pJLA[t] - r3 S z pJLA[t] - r4 S z pJLA[t] + r3 r4 S z pJLA[t] - r3 α2 pJLA[t] +
    r3 r4 α2 pJLA[t] + r3 S α2 pJLA[t] - r3 r4 S α2 pJLA[t] + r3 y α2 pJLA[t] - r3 r4 y α2 pJLA[t] - r3 S y α2 pJLA[t] +
    r3 r4 S y α2 pJLA[t] - r4 z α2 pJLA[t] + r3 r4 z α2 pJLA[t] + r4 S z α2 pJLA[t] - r3 r4 S z α2 pJLA[t] - z pJlB[t] +
    S z pJlB[t] - r3 pJLB[t] + r3 S pJLB[t] + r3 y pJLB[t] - r3 S y pJLB[t] - z pJLB[t] + r3 z pJLB[t] + S z pJLB[t] -
    r3 S z pJLB[t] + r3 β2 pJLB[t] - r3 r4 β2 pJLB[t] - r3 S β2 pJLB[t] + r3 r4 S β2 pJLB[t] - r3 y β2 pJLB[t] + r3 r4 y
      β2 pJLB[t] + r3 S y β2 pJLB[t] - r3 r4 S y β2 pJLB[t] + r4 z β2 pJLB[t] - r3 r4 z β2 pJLB[t] - r4 S z β2 pJLB[t] +
    r3 r4 S z β2 pJLB[t] - z pQlA[t] + S z pQlA[t] - r6 pQLA[t] + r6 S pQLA[t] + r6 y pQLA[t] - r6 S y pQLA[t] - z pQLA[t] +
    r6 z pQLA[t] + S z pQLA[t] - r6 S z pQLA[t] - α2 pQLA[t] + r6 α2 pQLA[t] + S α2 pQLA[t] - r6 S α2 pQLA[t] +
    y α2 pQLA[t] - r6 y α2 pQLA[t] - S y α2 pQLA[t] + r6 S y α2 pQLA[t] - r6 z α2 pQLA[t] + r6 S z α2 pQLA[t] -
    pQLB[t] + S pQLB[t] + y pQLB[t] - S y pQLB[t] - z pQLB[t] + S z pQLB[t] + β2 pQLB[t] - r6 β2 pQLB[t] -
    S β2 pQLB[t] + r6 S β2 pQLB[t] - y β2 pQLB[t] + r6 y β2 pQLB[t] + S y β2 pQLB[t] - r6 S y β2 pQLB[t] +
    r6 z β2 pQLB[t] - r6 S z β2 pQLB[t] + β1 (S z + z pJlA[t] - r3 z pJlA[t] - r4 z pJlA[t] + 2 r3 r4 z pJlA[t] - S z pJlA[t] +
        r3 S z pJlA[t] + r4 S z pJlA[t] - 2 r3 r4 S z pJlA[t] + r3 r4 pJLA[t] - r3 r4 S pJLA[t] - r3 r4 y pJLA[t] +
        r3 r4 S y pJLA[t] + z pJLA[t] - r3 z pJLA[t] - r4 z pJLA[t] + r3 r4 z pJLA[t] - S z pJLA[t] + r3 S z pJLA[t] +
        r4 S z pJLA[t] - r3 r4 S z pJLA[t] + z pJlB[t] - S z pJlB[t] + r3 r4 pJLB[t] - r3 r4 S pJLB[t] - r3 r4 y pJLB[t] +
        r3 r4 S y pJLB[t] + z pJLB[t] - r3 z pJLB[t] - r4 z pJLB[t] + r3 r4 z pJLB[t] - S z pJLB[t] + r3 S z pJLB[t] +
        r4 S z pJLB[t] - r3 r4 S z pJLB[t] + (z - S z) pQlA[t] + (-1 + S) (-z + r6 (-1 + y + z)) pQLA[t] + r6 pQLB[t] -
        r6 S pQLB[t] - r6 y pQLB[t] + r6 S y pQLB[t] + z pQLB[t] - r6 z pQLB[t] - S z pQLB[t] + r6 S z pQLB[t])) +
α1 ((-1 + S) z pQlA[t]^2 + pQlA[t] (-S z - z pJlA[t] + S z pJlA[t] - r3 r4 pJLA[t] + r3 r4 S pJLA[t] + r3 r4 y pJLA[t] -
        r3 r4 S y pJLA[t] - z pJLA[t] + r3 z pJLA[t] + r4 z pJLA[t] - r3 r4 z pJLA[t] + S z pJLA[t] - r3 S z pJLA[t] -
        r4 S z pJLA[t] + r3 r4 S z pJLA[t] - z pJlB[t] + r3 z pJlB[t] + r4 z pJlB[t] - 2 r3 r4 z pJlB[t] + S z pJlB[t] -
        r3 S z pJlB[t] - r4 S z pJlB[t] + 2 r3 r4 S z pJlB[t] - r3 r4 pJLB[t] + r3 r4 S pJLB[t] + r3 r4 y pJLB[t] -
        r3 r4 S y pJLB[t] - z pJLB[t] + r3 z pJLB[t] + r4 z pJLB[t] - r3 r4 z pJLB[t] + S z pJLB[t] - r3 S z pJLB[t] -
        r4 S z pJLB[t] + r3 r4 S z pJLB[t] - (-1 + S) (-z + r6 (-1 + y + z)) pQLA[t] + (-1 + S) z pQlB[t] - r6 pQLB[t] +
        r6 S pQLB[t] + r6 y pQLB[t] - r6 S y pQLB[t] - z pQLB[t] + r6 z pQLB[t] + S z pQLB[t] - r6 S z pQLB[t]) -
    (-1 + S) pJlA[t] ((-r4 + r3 (-1 + 2 r4)) z pQlB[t] - (r4 - r4 y + r3 r4 (-1 + y - z) + r3 z) (pQLA[t] + pQLB[t]))))) /
((S + pJlA[t] - S pJlA[t] + pJLA[t] - S pJLA[t] + pJlB[t] - S pJlB[t] + pJLB[t] - S pJLB[t] - (-1 + S) pQlA[t] +
    pQLA[t] - S pQLA[t] - (-1 + S) pQlB[t] + pQLB[t] - S pQLB[t])
  (pJlA[t] - k α1 pJlA[t] + pJLA[t] - k α2 pJLA[t] + pJlB[t] - k pJlB[t] + k β1 pJlB[t] + pJLB[t] - k pJLB[t] + k β2 pJLB[t] +
    (1 - k α1) pQlA[t] + pQLA[t] - k α2 pQLA[t] + (1 - k + k β1) pQlB[t] + pQLB[t] - k pQLB[t] + k β2 pQLB[t]));
```
