# Supplementary material for: Multi-locus models of deleterious epimutation-selection balance and the evolution of recombination modifiers
Source: G3 (Bethesda). 2026 Jun 11;16(8):jkag147. doi: 10.1093/g3journal/jkag147 (PMC13439953; doi:10.1093/g3journal/jkag147)
Supplement: Supplementary file 1 [file jkag147_supplementary_data.zip › File_S5_G3-2026-406777.pdf]

Model 3: Two locus haploid mating and diploid selection, full derivation and systematic analysis

Derivation of Recursion Equations

There are ten possible pairings of haploid gametes (see below) to generate diploid individuals.

Random Mating
tA X tA
tA X tB
tB X tB
TA X TA
TA X TB
TB X TB
tA X TA
tA X T B
TA X t B
tB X TB

Diploid frequencies after random mating, conditioned on epimutation occurring as the first step:

(Note: Due to the assumption of random mating, diploid parents can be assumed to be composed of haplotypes with given frequencies: $p_{i,j}$).

$$p_{t,A,t,A}{}^{(1)} = \left(p_{t,A}\,(1-\alpha_1)\,p_{t,A}\,(1-\alpha_1) + p_{t,A}\,(1-\alpha_1)\,p_{t,B}\,\beta_1 + p_{t,B}\,\beta_1\,p_{t,A}\,(1-\alpha_1) + p_{t,B}\,\beta_1\,p_{t,B}\,\beta_1\right)$$

$$p_{t,A,t,B}{}^{(1)} = \Big(\big(p_{t,A}\,\alpha_1\,p_{t,A}\,(1-\alpha_1) + p_{t,A}\,\alpha_1\,p_{t,B}\,\beta_1 + p_{t,A}\,(1-\alpha_1)\,p_{t,A}\,\alpha_1 + p_{t,A}\,(1-\alpha_1)\,p_{t,B}\,(1-\beta_1) + p_{t,B}\,\beta_1\,p_{t,A}\,\alpha_1 + p_{t,B}\,\beta_1\,p_{t,B}\,(1-\beta_1) + p_{t,B}\,(1-\beta_1)\,p_{t,B}\,\beta_1 + p_{t,B}\,(1-\beta_1)\,p_{t,A}\,(1-\alpha_1)\big)\Big)$$

$$p_{t,B,t,B}{}^{(1)} = \left(p_{t,A}\,\alpha_1\,p_{t,A}\,\alpha_1 + p_{t,A}\,\alpha_1\,p_{t,B}\,(1-\beta_1) + p_{t,B}\,(1-\beta_1)\,p_{t,B}\,(1-\beta_1) + p_{t,B}\,(1-\beta_1)\,p_{t,A}\,\alpha_1\right)$$

$$p_{T,A,T,A}{}^{(1)} = \Big(\big((1-\alpha_2)^2\,p_{T,A}^2 + 2\,(1-\alpha_2)\,\beta_2\,p_{T,A}\,p_{T,B} + \beta_2^2\,p_{T,B}^2\big)\Big)$$

$$p_{T,A,T,B}{}^{(1)} = \left(2\,(1-\alpha_2)\,\alpha_2\,p_{T,A}^2 + 2\,(1-\alpha_2)\,(1-\beta_2)\,p_{T,A}\,p_{T,B} + 2\,\alpha_2\,\beta_2\,p_{T,A}\,p_{T,B} + 2\,(1-\beta_2)\,\beta_2\,p_{T,B}^2\right)$$

$$p_{T,B,T,B}{}^{(1)} = \Big(\big(\alpha_2^2\,p_{T,A}^2 + 2\,\alpha_2\,(1-\beta_2)\,p_{T,A}\,p_{T,B} + (1-\beta_2)^2\,p_{T,B}^2\big)\Big)$$

$$p_{t,A,T,A}{}^{(1)} = 2\left(p_{t,A}\,(1-\alpha_1)\,p_{T,A}\,(1-\alpha_2) + p_{t,A}\,(1-\alpha_1)\,p_{T,B}\,\beta_2 + p_{t,B}\,\beta_1\,p_{T,A}\,(1-\alpha_2) + p_{t,B}\,\beta_1\,p_{T,B}\,\beta_2\right)$$

$$p_{t,B,T,A}{}^{(1)} = 2\left(p_{t,A}\,\alpha_1\,p_{T,A}\,(1-\alpha_2) + p_{t,A}\,\alpha_1\,p_{T,B}\,\beta_2 + p_{t,B}\,(1-\beta_1)\,p_{T,A}\,(1-\alpha_2) + p_{t,B}\,(1-\beta_1)\,p_{T,B}\,\beta_2\right)$$

$$p_{t,A,T,B}{}^{(1)} = 2\left(p_{t,A}\,(1-\alpha_1)\,p_{T,A}\,\alpha_2 + p_{t,A}\,(1-\alpha_1)\,p_{T,B}\,(1-\beta_2) + p_{t,B}\,\beta_1\,p_{T,A}\,\alpha_2 + p_{t,B}\,\beta_1\,p_{T,B}\,(1-\beta_2)\right)$$

$$p_{t,B,T,B}{}^{(1)} = 2\left(p_{t,A}\,\alpha_1\,p_{T,A}\,\alpha_2 + p_{t,A}\,\alpha_1\,p_{T,B}\,(1-\beta_2) + p_{t,B}\,(1-\beta_1)\,p_{T,A}\,\alpha_2 + p_{t,B}\,(1-\beta_1)\,p_{T,B}\,(1-\beta_2)\right)$$

$$p_{t,A}{}^{(1)} = p_{t,A,t,A}{}^{(1)} + \frac{1}{2}\,p_{t,A,t,B}{}^{(1)} + \frac{1}{2}\,p_{t,A,T,A}{}^{(1)} + \frac{1}{2}\,p_{t,A,T,B}{}^{(1)}$$

$$p_{t,B}{}^{(1)} = p_{t,B,t,B}{}^{(1)} + \frac{1}{2}\,p_{t,A,t,B}{}^{(1)} + \frac{1}{2}\,p_{t,B,T,A}{}^{(1)} + \frac{1}{2}\,p_{t,B,T,B}{}^{(1)}$$

$$p_{T,A}{}^{(1)} = p_{T,A,T,A}{}^{(1)} + \frac{1}{2}\,p_{t,A,T,A}{}^{(1)} + \frac{1}{2}\,p_{t,B,T,A}{}^{(1)} + \frac{1}{2}\,p_{T,A,T,B}{}^{(1)}$$

$$p_{T,B}{}^{(1)} = p_{T,B,T,B}{}^{(1)} + \frac{1}{2}\,p_{t,A,T,B}{}^{(1)} + \frac{1}{2}\,p_{t,B,T,B}{}^{(1)} + \frac{1}{2}\,p_{T,A,T,B}{}^{(1)}$$

Natural selection occurs on diploid organisms as the second event.

$$p_{t,A,t,A}{}^{(2)} = w_1\left(\frac{1}{\overline{w}}\right)p_{t,A,t,A}{}^{(1)}$$

$$p_{t,A,t,B}{}^{(2)} = w_2\left(\frac{1}{\overline{w}}\right)p_{t,A,t,B}{}^{(1)}$$

$$p_{t,B,t,B}{}^{(2)} = w_3\left(\frac{1}{\overline{w}}\right)p_{t,B,t,B}{}^{(1)}$$

$$p_{T,A,T,A}{}^{(2)} = w_4\left(\frac{1}{\overline{w}}\right)p_{T,A,T,A}{}^{(1)}$$

$$p_{T,A,T,B}{}^{(2)} = w_5\left(\frac{1}{\overline{w}}\right)p_{T,A,T,B}{}^{(1)}$$

$$p_{T,B,T,B}{}^{(2)} = w_6\left(\frac{1}{\overline{w}}\right)p_{T,B,T,B}{}^{(1)}$$

$$p_{t,A,T,A}{}^{(2)} = w_7\left(\frac{1}{\overline{w}}\right)p_{t,A,T,A}{}^{(1)}$$

$$p_{t,B,T,A}{}^{(2)} = w_8\left(\frac{1}{\overline{w}}\right)p_{t,B,T,A}{}^{(1)}$$

$$p_{t,A,T,B}{}^{(2)} = w_9 \left(\frac{1}{\overline{w}}\right) p_{t,A,T,B}{}^{(1)}$$

$$p_{t,B,T,B}{}^{(2)} = w_{10} \left(\frac{1}{\overline{w}}\right) p_{t,B,T,B}{}^{(1)}$$

where $\overline{w}$ =

$$w_1\, p_{t,A,t,A}{}^{(1)} + w_2\, p_{t,A,t,B}{}^{(1)} + w_3\, p_{t,B,t,B}{}^{(1)} + w_4\, p_{T,A,T,A}{}^{(1)} + w_5\, p_{T,A,T,B}{}^{(1)} + w_6\, p_{T,B,T,B}{}^{(1)} + w_7\, p_{t,A,T,A}{}^{(1)} + w_8\, p_{t,B,T,A}{}^{(1)} + w_9\, p_{t,A,T,B}{}^{(1)} + w_{10}\, p_{t,B,T,B}{}^{(1)}$$

Recombination occurs generating haploid gametes for the third event.

$$p_{t,A}{}^{(3)} = p_{t,A,t,A}{}^{(2)} + \frac{1}{2}\, p_{t,A,t,B}{}^{(2)} + \frac{1}{2}\, p_{t,A,T,A}{}^{(2)} + \frac{1}{2}\, r\, p_{t,B,T,A}{}^{(2)} + \frac{1}{2}\, (1-r)\, p_{t,A,T,B}{}^{(2)}$$

$$p_{t,B}{}^{(3)} = \frac{1}{2}\, p_{t,A,t,B}{}^{(2)} + p_{t,B,t,B}{}^{(2)} + \frac{1}{2}\, (1-r)\, p_{t,B,T,A}{}^{(2)} + \frac{1}{2}\, r\, p_{t,A,T,B}{}^{(2)} + \frac{1}{2}\, p_{t,B,T,B}{}^{(2)}$$

$$p_{T,A}{}^{(3)} = p_{T,A,T,A}{}^{(2)} + \frac{1}{2}\, p_{T,A,T,B}{}^{(2)} + \frac{1}{2}\, p_{t,A,T,A}{}^{(2)} + \frac{1}{2}\, (1-r)\, p_{t,B,T,A}{}^{(2)} + \frac{1}{2}\, r\, p_{t,A,T,B}{}^{(2)}$$

$$p_{T,B}{}^{(3)} = \frac{1}{2}\, p_{T,A,T,B}{}^{(2)} + p_{T,B,T,B}{}^{(2)} + \frac{1}{2}\, r\, p_{t,B,T,A}{}^{(2)} + \frac{1}{2}\, (1-r)\, p_{t,A,T,B}{}^{(2)} + \frac{1}{2}\, p_{t,B,T,B}{}^{(2)}$$

Gametes undergo transposition/deletion as the final event.

$$p_{t,A}{}^{(4)} = p_{t,A}{}^{(3)}\, (1-z) + p_{T,A}{}^{(3)}\, y$$

$$p_{t,B}{}^{(4)} = p_{t,B}{}^{(3)}\, (1-z) + p_{T,B}{}^{(3)}\, y$$

$$p_{T,A}{}^{(4)} = p_{T,A}{}^{(3)}\, (1-y) + p_{t,A}{}^{(3)}\, z$$

$$p_{T,B}{}^{(4)} = p_{T,B}{}^{(3)}\, (1-y) + p_{t,B}{}^{(3)}\, z$$

After substituting each event into the subsequent event and simplifying, the final recursion equations are derived:

$$\begin{aligned}
p_{t,A}{}' = \Big(&(1-z)\Big(2\, w_1 \left((-1+\alpha_1)\, p_{t,A} - \beta_1\, p_{t,B}\right)^2 + w_2 \left(-2\, \alpha_1^2\, p_{t,A}^2 - 2\, (-1+\beta_1)\, p_{t,B} \left(p_{t,A} + \beta_1\, p_{t,B}\right) + 2\, \alpha_1\, p_{t,A} \left(p_{t,A} + \left(-1+2\,\beta_1\right) p_{t,B}\right)\right) - \\
&\quad 2\, (1-r)\, w_8 \left((-1+\alpha_1)\, p_{t,A} - \beta_1\, p_{t,B}\right) \left(\alpha_2\, p_{T,A} - (-1+\beta_2)\, p_{T,B}\right) - \\
&\quad 2\, r\, w_9 \left(\alpha_1\, p_{t,A} - (-1+\beta_1)\, p_{t,B}\right) \left((-1+\alpha_2)\, p_{T,A} - \beta_2\, p_{T,B}\right) + 2\, w_7 \left((-1+\alpha_1)\, p_{t,A} - \beta_1\, p_{t,B}\right) \left((-1+\alpha_2)\, p_{T,A} - \beta_2\, p_{T,B}\right)\Big) + \\
&y\Big(2\, w_4 \left((-1+\alpha_1)\, p_{T,A} - \beta_1\, p_{T,B}\right)^2 - 2\, r\, w_8 \left((-1+\alpha_1)\, p_{t,A} - \beta_1\, p_{t,B}\right) \left(\alpha_2\, p_{T,A} - (-1+\beta_2)\, p_{T,B}\right) + \\
&\quad 2\, (-1+r)\, w_9 \left(\alpha_1\, p_{t,A} - (-1+\beta_1)\, p_{t,B}\right) \left((-1+\alpha_2)\, p_{T,A} - \beta_2\, p_{T,B}\right) + 2\, w_7 \left((-1+\alpha_1)\, p_{t,A} - \beta_1\, p_{t,B}\right) \left((-1+\alpha_2)\, p_{T,A} - \beta_2\, p_{T,B}\right) + \\
&\quad w_5 \left(-2\, \alpha_2^2\, p_{T,A}^2 - 2\, (-1+\beta_2)\, p_{T,B} \left(p_{T,A} + \beta_2\, p_{T,B}\right) + 2\, \alpha_2\, p_{T,A} \left(p_{T,A} + \left(-1+2\,\beta_2\right) p_{T,B}\right)\right)\Big)\Big) \Big/ \\
\Big(2\Big(&w_3 \left(\alpha_1\, p_{t,A} - (-1+\beta_1)\, p_{t,B}\right)^2 + w_1 \left((-1+\alpha_1)\, p_{t,A} - \beta_1\, p_{t,B}\right)^2 + w_2 \left(-2\, \alpha_1^2\, p_{t,A}^2 - 2\, (-1+\beta_1)\, p_{t,B} \left(p_{t,A} + \beta_1\, p_{t,B}\right) + \right. \\
&\quad \left. 2\, \alpha_1\, p_{t,A} \left(p_{t,A} + \left(-1+2\,\beta_1\right) p_{t,B}\right)\right) + w_4 \left((-1+\alpha_1)\, p_{T,A} - \beta_1\, p_{T,B}\right)^2 + 2\, w_{10} \left(\alpha_1\, p_{t,A} - (-1+\beta_1)\, p_{t,B}\right) \left(\alpha_2\, p_{T,A} + (1-\beta_2)\, p_{T,B}\right) -
\end{aligned}$$

$$2\,w_8\left((-1+\alpha_1)\,p_{t,A}-\beta_1\,p_{t,B}\right)\left(\alpha_2\,p_{T,A}-(-1+\beta_2)\,p_{T,B}\right)+w_6\left(\alpha_2\,p_{T,A}-(-1+\beta_2)\,p_{T,B}\right)^2-$$
$$2\,w_9\left(\alpha_1\,p_{t,A}-(-1+\beta_1)\,p_{t,B}\right)\left((-1+\alpha_2)\,p_{T,A}-\beta_2\,p_{T,B}\right)+2\,w_7\left((-1+\alpha_1)\,p_{t,A}-\beta_1\,p_{t,B}\right)\left((-1+\alpha_2)\,p_{T,A}-\beta_2\,p_{T,B}\right)+$$
$$w_5\left(-2\,\alpha_2^2\,p_{T,A}^2-2\,(-1+\beta_2)\,p_{T,B}\left(p_{T,A}+\beta_2\,p_{T,B}\right)+2\,\alpha_2\,p_{T,A}\left(p_{T,A}+\left(-1+2\,\beta_2\right)p_{T,B}\right)\right)\Big)\Big)$$

$$p_{t,B}{}' = \Big((1-z)\Big(2\,w_3\left(\alpha_1\,p_{t,A}-(-1+\beta_1)\,p_{t,B}\right)^2+w_2\left(-2\,\alpha_1^2\,p_{t,A}^2-2\,(-1+\beta_1)\,p_{t,B}\left(p_{t,A}+\beta_1\,p_{t,B}\right)+2\,\alpha_1\,p_{t,A}\left(p_{t,A}+\left(-1+2\,\beta_1\right)p_{t,B}\right)\right)+$$
$$2\,w_{10}\left(\alpha_1\,p_{t,A}-(-1+\beta_1)\,p_{t,B}\right)\left(\alpha_2\,p_{T,A}+(1-\beta_2)\,p_{T,B}\right)-2\,r\,w_8\left((-1+\alpha_1)\,p_{t,A}-\beta_1\,p_{t,B}\right)\left(\alpha_2\,p_{T,A}-(-1+\beta_2)\,p_{T,B}\right)+$$
$$2\,(-1+r)\,w_9\left(\alpha_1\,p_{t,A}-(-1+\beta_1)\,p_{t,B}\right)\left((-1+\alpha_2)\,p_{T,A}-\beta_2\,p_{T,B}\right)\Big)+$$
$$y\Big(2\,w_{10}\left(\alpha_1\,p_{t,A}-(-1+\beta_1)\,p_{t,B}\right)\left(\alpha_2\,p_{T,A}+(1-\beta_2)\,p_{T,B}\right)-2\,(1-r)\,w_8\left((-1+\alpha_1)\,p_{t,A}-\beta_1\,p_{t,B}\right)\left(\alpha_2\,p_{T,A}-(-1+\beta_2)\,p_{T,B}\right)+$$
$$2\,w_6\left(\alpha_2\,p_{T,A}-(-1+\beta_2)\,p_{T,B}\right)^2-2\,r\,w_9\left(\alpha_1\,p_{t,A}-(-1+\beta_1)\,p_{t,B}\right)\left((-1+\alpha_2)\,p_{T,A}-\beta_2\,p_{T,B}\right)+$$
$$w_5\left(-2\,\alpha_2^2\,p_{T,A}^2-2\,(-1+\beta_2)\,p_{T,B}\left(p_{T,A}+\beta_2\,p_{T,B}\right)+2\,\alpha_2\,p_{T,A}\left(p_{T,A}+\left(-1+2\,\beta_2\right)p_{T,B}\right)\right)\Big)\Big)\Big/$$
$$\Big(2\Big(w_3\left(\alpha_1\,p_{t,A}-(-1+\beta_1)\,p_{t,B}\right)^2+w_1\left((-1+\alpha_1)\,p_{t,A}-\beta_1\,p_{t,B}\right)^2+w_2\Big(-2\,\alpha_1^2\,p_{t,A}^2-2\,(-1+\beta_1)\,p_{t,B}\left(p_{t,A}+\beta_1\,p_{t,B}\right)+$$
$$2\,\alpha_1\,p_{t,A}\left(p_{t,A}+\left(-1+2\,\beta_1\right)p_{t,B}\right)\Big)+w_4\left((-1+\alpha_1)\,p_{T,A}-\beta_1\,p_{T,B}\right)^2+2\,w_{10}\left(\alpha_1\,p_{t,A}-(-1+\beta_1)\,p_{t,B}\right)\left(\alpha_2\,p_{T,A}+(1-\beta_2)\,p_{T,B}\right)-$$
$$2\,w_8\left((-1+\alpha_1)\,p_{t,A}-\beta_1\,p_{t,B}\right)\left(\alpha_2\,p_{T,A}-(-1+\beta_2)\,p_{T,B}\right)+w_6\left(\alpha_2\,p_{T,A}-(-1+\beta_2)\,p_{T,B}\right)^2-$$
$$2\,w_9\left(\alpha_1\,p_{t,A}-(-1+\beta_1)\,p_{t,B}\right)\left((-1+\alpha_2)\,p_{T,A}-\beta_2\,p_{T,B}\right)+2\,w_7\left((-1+\alpha_1)\,p_{t,A}-\beta_1\,p_{t,B}\right)\left((-1+\alpha_2)\,p_{T,A}-\beta_2\,p_{T,B}\right)+$$
$$w_5\left(-2\,\alpha_2^2\,p_{T,A}^2-2\,(-1+\beta_2)\,p_{T,B}\left(p_{T,A}+\beta_2\,p_{T,B}\right)+2\,\alpha_2\,p_{T,A}\left(p_{T,A}+\left(-1+2\,\beta_2\right)p_{T,B}\right)\right)\Big)\Big)$$

$$p_{T,A}{}' = \Big(z\Big(2\,w_1\left((-1+\alpha_1)\,p_{t,A}-\beta_1\,p_{t,B}\right)^2+w_2\left(-2\,\alpha_1^2\,p_{t,A}^2-2\,(-1+\beta_1)\,p_{t,B}\left(p_{t,A}+\beta_1\,p_{t,B}\right)+2\,\alpha_1\,p_{t,A}\left(p_{t,A}+\left(-1+2\,\beta_1\right)p_{t,B}\right)\right)-$$
$$2\,(1-r)\,w_8\left((-1+\alpha_1)\,p_{t,A}-\beta_1\,p_{t,B}\right)\left(\alpha_2\,p_{T,A}-(-1+\beta_2)\,p_{T,B}\right)-$$
$$2\,r\,w_9\left(\alpha_1\,p_{t,A}-(-1+\beta_1)\,p_{t,B}\right)\left((-1+\alpha_2)\,p_{T,A}-\beta_2\,p_{T,B}\right)+2\,w_7\left((-1+\alpha_1)\,p_{t,A}-\beta_1\,p_{t,B}\right)\left((-1+\alpha_2)\,p_{T,A}-\beta_2\,p_{T,B}\right)\Big)+$$
$$(1-y)\Big(2\,w_4\left((-1+\alpha_1)\,p_{T,A}-\beta_1\,p_{T,B}\right)^2-2\,r\,w_8\left((-1+\alpha_1)\,p_{t,A}-\beta_1\,p_{t,B}\right)\left(\alpha_2\,p_{T,A}-(-1+\beta_2)\,p_{T,B}\right)+$$
$$2\,(-1+r)\,w_9\left(\alpha_1\,p_{t,A}-(-1+\beta_1)\,p_{t,B}\right)\left((-1+\alpha_2)\,p_{T,A}-\beta_2\,p_{T,B}\right)+2\,w_7\left((-1+\alpha_1)\,p_{t,A}-\beta_1\,p_{t,B}\right)\left((-1+\alpha_2)\,p_{T,A}-\beta_2\,p_{T,B}\right)+$$
$$w_5\left(-2\,\alpha_2^2\,p_{T,A}^2-2\,(-1+\beta_2)\,p_{T,B}\left(p_{T,A}+\beta_2\,p_{T,B}\right)+2\,\alpha_2\,p_{T,A}\left(p_{T,A}+\left(-1+2\,\beta_2\right)p_{T,B}\right)\right)\Big)\Big)\Big/$$
$$\Big(2\Big(w_3\left(\alpha_1\,p_{t,A}-(-1+\beta_1)\,p_{t,B}\right)^2+w_1\left((-1+\alpha_1)\,p_{t,A}-\beta_1\,p_{t,B}\right)^2+w_2\Big(-2\,\alpha_1^2\,p_{t,A}^2-2\,(-1+\beta_1)\,p_{t,B}\left(p_{t,A}+\beta_1\,p_{t,B}\right)+$$
$$2\,\alpha_1\,p_{t,A}\left(p_{t,A}+\left(-1+2\,\beta_1\right)p_{t,B}\right)\Big)+w_4\left((-1+\alpha_1)\,p_{T,A}-\beta_1\,p_{T,B}\right)^2+2\,w_{10}\left(\alpha_1\,p_{t,A}-(-1+\beta_1)\,p_{t,B}\right)\left(\alpha_2\,p_{T,A}+(1-\beta_2)\,p_{T,B}\right)-$$
$$2\,w_8\left((-1+\alpha_1)\,p_{t,A}-\beta_1\,p_{t,B}\right)\left(\alpha_2\,p_{T,A}-(-1+\beta_2)\,p_{T,B}\right)+w_6\left(\alpha_2\,p_{T,A}-(-1+\beta_2)\,p_{T,B}\right)^2-$$
$$2\,w_9\left(\alpha_1\,p_{t,A}-(-1+\beta_1)\,p_{t,B}\right)\left((-1+\alpha_2)\,p_{T,A}-\beta_2\,p_{T,B}\right)+2\,w_7\left((-1+\alpha_1)\,p_{t,A}-\beta_1\,p_{t,B}\right)\left((-1+\alpha_2)\,p_{T,A}-\beta_2\,p_{T,B}\right)+$$
$$w_5\left(-2\,\alpha_2^2\,p_{T,A}^2-2\,(-1+\beta_2)\,p_{T,B}\left(p_{T,A}+\beta_2\,p_{T,B}\right)+2\,\alpha_2\,p_{T,A}\left(p_{T,A}+\left(-1+2\,\beta_2\right)p_{T,B}\right)\right)\Big)\Big)$$

$$p_{T,B}{}' = \Big(z\Big(2\,w_3\left(\alpha_1\,p_{t,A}-(-1+\beta_1)\,p_{t,B}\right)^2+w_2\left(-2\,\alpha_1^2\,p_{t,A}^2-2\,(-1+\beta_1)\,p_{t,B}\left(p_{t,A}+\beta_1\,p_{t,B}\right)+2\,\alpha_1\,p_{t,A}\left(p_{t,A}+\left(-1+2\,\beta_1\right)p_{t,B}\right)\right)+$$
$$2\,w_{10}\left(\alpha_1\,p_{t,A}-(-1+\beta_1)\,p_{t,B}\right)\left(\alpha_2\,p_{T,A}+(1-\beta_2)\,p_{T,B}\right)-2\,r\,w_8\left((-1+\alpha_1)\,p_{t,A}-\beta_1\,p_{t,B}\right)\left(\alpha_2\,p_{T,A}-(-1+\beta_2)\,p_{T,B}\right)+$$
$$2\,(-1+r)\,w_9\left(\alpha_1\,p_{t,A}-(-1+\beta_1)\,p_{t,B}\right)\left((-1+\alpha_2)\,p_{T,A}-\beta_2\,p_{T,B}\right)\Big)+$$
$$(1-y)\Big(2\,w_{10}\left(\alpha_1\,p_{t,A}-(-1+\beta_1)\,p_{t,B}\right)\left(\alpha_2\,p_{T,A}+(1-\beta_2)\,p_{T,B}\right)-2\,(1-r)\,w_8\left((-1+\alpha_1)\,p_{t,A}-\beta_1\,p_{t,B}\right)\left(\alpha_2\,p_{T,A}-(-1+\beta_2)\,p_{T,B}\right)+$$
$$2\,w_6\left(\alpha_2\,p_{T,A}-(-1+\beta_2)\,p_{T,B}\right)^2-2\,r\,w_9\left(\alpha_1\,p_{t,A}-(-1+\beta_1)\,p_{t,B}\right)\left((-1+\alpha_2)\,p_{T,A}-\beta_2\,p_{T,B}\right)+$$
$$w_5\left(-2\,\alpha_2^2\,p_{T,A}^2-2\,(-1+\beta_2)\,p_{T,B}\left(p_{T,A}+\beta_2\,p_{T,B}\right)+2\,\alpha_2\,p_{T,A}\left(p_{T,A}+\left(-1+2\,\beta_2\right)p_{T,B}\right)\right)\Big)\Big)\Big/$$
$$\Big(2\Big(w_3\left(\alpha_1\,p_{t,A}-(-1+\beta_1)\,p_{t,B}\right)^2+w_1\left((-1+\alpha_1)\,p_{t,A}-\beta_1\,p_{t,B}\right)^2+w_2\Big(-2\,\alpha_1^2\,p_{t,A}^2-2\,(-1+\beta_1)\,p_{t,B}\left(p_{t,A}+\beta_1\,p_{t,B}\right)+$$
$$2\,\alpha_1\,p_{t,A}\left(p_{t,A}+\left(-1+2\,\beta_1\right)p_{t,B}\right)\Big)+w_4\left((-1+\alpha_1)\,p_{T,A}-\beta_1\,p_{T,B}\right)^2+2\,w_{10}\left(\alpha_1\,p_{t,A}-(-1+\beta_1)\,p_{t,B}\right)\left(\alpha_2\,p_{T,A}+(1-\beta_2)\,p_{T,B}\right)-$$
$$2\,w_8\left((-1+\alpha_1)\,p_{t,A}-\beta_1\,p_{t,B}\right)\left(\alpha_2\,p_{T,A}-(-1+\beta_2)\,p_{T,B}\right)+w_6\left(\alpha_2\,p_{T,A}-(-1+\beta_2)\,p_{T,B}\right)^2-$$
$$2\,w_9\left(\alpha_1\,p_{t,A}-(-1+\beta_1)\,p_{t,B}\right)\left((-1+\alpha_2)\,p_{T,A}-\beta_2\,p_{T,B}\right)+2\,w_7\left((-1+\alpha_1)\,p_{t,A}-\beta_1\,p_{t,B}\right)\left((-1+\alpha_2)\,p_{T,A}-\beta_2\,p_{T,B}\right)+$$

$$w_5\left(-2\,\alpha_2^2\,p_{T,A}^2-2\,(-1+\beta_2)\,p_{T,B}\left(p_{T,A}+\beta_2\,p_{T,B}\right)+2\,\alpha_2\,p_{T,A}\left(p_{T,A}+\left(-1+2\,\beta_2\right)p_{T,B}\right)\right)\Big)\Big)$$

Case A: The epiallele is deleterious and the transposon is neutral

A1 - Fitness Assumptions

Genotype	Fitness
tA/tA	$w_1 = 1$
tA/tB	$w_2 = 1 - hk$
tB/tB	$w_3 = 1 - k$
TA/TA	$w_4 = 1$
TA/TB	$w_5 = 1 - hk$
TB/TB	$w_6 = 1 - k$
tA/TA	$w_7 = 1$
tB/TA	$w_8 = 1 - hk$
tA/TB	$w_9 = 1 - hk$
tB/TB	$w_{10} = 1 - k$

A2 - Calculating Equilibria

$$\Big\{0 == \Big(z\Big(2\,w_1\left((-1+\alpha_1)\,p_{t,A}-\beta_1\,p_{t,B}\right)^2+$$
$$w_2\left(-2\,\alpha_1^2\,p_{t,A}^2-2\,(-1+\beta_1)\,p_{t,B}\left(p_{t,A}+\beta_1\,p_{t,B}\right)+2\,\alpha_1\,p_{t,A}\left(p_{t,A}+\left(-1+2\,\beta_1\right)p_{t,B}\right)\right)-$$
$$2\,(1-r)\,w_8\left((-1+\alpha_1)\,p_{t,A}-\beta_1\,p_{t,B}\right)\left(\alpha_2\,p_{T,A}-(-1+\beta_2)\,p_{T,B}\right)-2\,r\,w_9\left(\alpha_1\,p_{t,A}-(-1+\beta_1)\,p_{t,B}\right)$$
$$\left((-1+\alpha_2)\,p_{T,A}-\beta_2\,p_{T,B}\right)+2\,w_7\left((-1+\alpha_1)\,p_{t,A}-\beta_1\,p_{t,B}\right)\left((-1+\alpha_2)\,p_{T,A}-\beta_2\,p_{T,B}\right)\Big)+$$
$$(1-y)\Big(2\,w_4\left((-1+\alpha_1)\,p_{T,A}-\beta_1\,p_{T,B}\right)^2-2\,r\,w_8\left((-1+\alpha_1)\,p_{t,A}-\beta_1\,p_{t,B}\right)\left(\alpha_2\,p_{T,A}-(-1+\beta_2)\,p_{T,B}\right)+$$
$$2\,(-1+r)\,w_9\left(\alpha_1\,p_{t,A}-(-1+\beta_1)\,p_{t,B}\right)\left((-1+\alpha_2)\,p_{T,A}-\beta_2\,p_{T,B}\right)+$$
$$2\,w_7\left((-1+\alpha_1)\,p_{t,A}-\beta_1\,p_{t,B}\right)\left((-1+\alpha_2)\,p_{T,A}-\beta_2\,p_{T,B}\right)+$$
$$w_5\left(-2\,\alpha_2^2\,p_{T,A}^2-2\,(-1+\beta_2)\,p_{T,B}\left(p_{T,A}+\beta_2\,p_{T,B}\right)+2\,\alpha_2\,p_{T,A}\left(p_{T,A}+\left(-1+2\,\beta_2\right)p_{T,B}\right)\right)\Big)\Big)\Big/$$
$$\Big(2\Big(w_3\left(\alpha_1\,p_{t,A}-(-1+\beta_1)\,p_{t,B}\right)^2+w_1\left((-1+\alpha_1)\,p_{t,A}-\beta_1\,p_{t,B}\right)^2+$$
$$w_2\left(-2\,\alpha_1^2\,p_{t,A}^2-2\,(-1+\beta_1)\,p_{t,B}\left(p_{t,A}+\beta_1\,p_{t,B}\right)+2\,\alpha_1\,p_{t,A}\left(p_{t,A}+\left(-1+2\,\beta_1\right)p_{t,B}\right)\right)+$$
$$w_4\left((-1+\alpha_1)\,p_{T,A}-\beta_1\,p_{T,B}\right)^2+2\,w_{10}\left(\alpha_1\,p_{t,A}-(-1+\beta_1)\,p_{t,B}\right)\left(\alpha_2\,p_{T,A}+(1-\beta_2)\,p_{T,B}\right)-$$
$$2\,w_8\left((-1+\alpha_1)\,p_{t,A}-\beta_1\,p_{t,B}\right)\left(\alpha_2\,p_{T,A}-(-1+\beta_2)\,p_{T,B}\right)+$$
$$w_6\left(\alpha_2\,p_{T,A}-(-1+\beta_2)\,p_{T,B}\right)^2-2\,w_9\left(\alpha_1\,p_{t,A}-(-1+\beta_1)\,p_{t,B}\right)\left((-1+\alpha_2)\,p_{T,A}-\beta_2\,p_{T,B}\right)+$$
$$2\,w_7\left((-1+\alpha_1)\,p_{t,A}-\beta_1\,p_{t,B}\right)\left((-1+\alpha_2)\,p_{T,A}-\beta_2\,p_{T,B}\right)+$$
$$w_5\left(-2\,\alpha_2^2\,p_{T,A}^2-2\,(-1+\beta_2)\,p_{T,B}\left(p_{T,A}+\beta_2\,p_{T,B}\right)+2\,\alpha_2\,p_{T,A}\left(p_{T,A}+\left(-1+2\,\beta_2\right)p_{T,B}\right)\right)\Big)\Big)$$
$$-p_{T,A},$$
$$0 == \Big((1-z)\Big(2\,w_3\left(\alpha_1\,p_{t,A}-(-1+\beta_1)\,p_{t,B}\right)^2+w_2\Big(-2\,\alpha_1^2\,p_{t,A}^2-2\,(-1+\beta_1)\,p_{t,B}\left(p_{t,A}+\beta_1\,p_{t,B}\right)+2\,\alpha_1\,p_{t,A}$$
$$\left(p_{t,A}+\left(-1+2\,\beta_1\right)p_{t,B}\right)\Big)+2\,w_{10}\left(\alpha_1\,p_{t,A}-(-1+\beta_1)\,p_{t,B}\right)\left(\alpha_2\,p_{T,A}+(1-\beta_2)\,p_{T,B}\right)-$$
$$2\,r\,w_8\left((-1+\alpha_1)\,p_{t,A}-\beta_1\,p_{t,B}\right)\left(\alpha_2\,p_{T,A}-(-1+\beta_2)\,p_{T,B}\right)+2\,(-1+r)\,w_9\left(\alpha_1\,p_{t,A}-(-1+\beta_1)\,p_{t,B}\right)$$
$$\left((-1+\alpha_2)\,p_{T,A}-\beta_2\,p_{T,B}\right)\Big)+y\Big(2\,w_{10}\left(\alpha_1\,p_{t,A}-(-1+\beta_1)\,p_{t,B}\right)\left(\alpha_2\,p_{T,A}+(1-\beta_2)\,p_{T,B}\right)-$$

$$2\,(1-r)\,w_8\,((-1+\alpha_1)\,p_{t,A}-\beta_1\,p_{t,B})\,(\alpha_2\,p_{T,A}-(-1+\beta_2)\,p_{T,B})+2\,w_6\,(\alpha_2\,p_{T,A}-(-1+\beta_2)\,p_{T,B})^2-$$
$$2\,r\,w_9\,(\alpha_1\,p_{t,A}-(-1+\beta_1)\,p_{t,B})\,((-1+\alpha_2)\,p_{T,A}-\beta_2\,p_{T,B})+$$
$$w_5\,(-2\,\alpha_2^2\,p_{T,A}^2-2\,(-1+\beta_2)\,p_{T,B}\,(p_{T,A}+\beta_2\,p_{T,B})+2\,\alpha_2\,p_{T,A}\,(p_{T,A}+(-1+2\,\beta_2)\,p_{T,B}))))\,/$$
$$(2\,(w_3\,(\alpha_1\,p_{t,A}-(-1+\beta_1)\,p_{t,B})^2+w_1\,((-1+\alpha_1)\,p_{t,A}-\beta_1\,p_{t,B})^2+$$
$$w_2\,(-2\,\alpha_1^2\,p_{t,A}^2-2\,(-1+\beta_1)\,p_{t,B}\,(p_{t,A}+\beta_1\,p_{t,B})+2\,\alpha_1\,p_{t,A}\,(p_{t,A}+(-1+2\,\beta_1)\,p_{t,B}))+$$
$$w_4\,((-1+\alpha_1)\,p_{T,A}-\beta_1\,p_{T,B})^2+2\,w_{10}\,(\alpha_1\,p_{t,A}-(-1+\beta_1)\,p_{t,B})\,(\alpha_2\,p_{T,A}+(1-\beta_2)\,p_{T,B})-$$
$$2\,w_8\,((-1+\alpha_1)\,p_{t,A}-\beta_1\,p_{t,B})\,(\alpha_2\,p_{T,A}-(-1+\beta_2)\,p_{T,B})+$$
$$w_6\,(\alpha_2\,p_{T,A}-(-1+\beta_2)\,p_{T,B})^2-2\,w_9\,(\alpha_1\,p_{t,A}-(-1+\beta_1)\,p_{t,B})\,((-1+\alpha_2)\,p_{T,A}-\beta_2\,p_{T,B})+$$
$$2\,w_7\,((-1+\alpha_1)\,p_{t,A}-\beta_1\,p_{t,B})\,((-1+\alpha_2)\,p_{T,A}-\beta_2\,p_{T,B})+$$
$$w_5\,(-2\,\alpha_2^2\,p_{T,A}^2-2\,(-1+\beta_2)\,p_{T,B}\,(p_{T,A}+\beta_2\,p_{T,B})+2\,\alpha_2\,p_{T,A}\,(p_{T,A}+(-1+2\,\beta_2)\,p_{T,B}))))-p_{t,B},$$

$$0==(z\,(2\,w_3\,(\alpha_1\,p_{t,A}-(-1+\beta_1)\,p_{t,B})^2+$$
$$w_2\,(-2\,\alpha_1^2\,p_{t,A}^2-2\,(-1+\beta_1)\,p_{t,B}\,(p_{t,A}+\beta_1\,p_{t,B})+2\,\alpha_1\,p_{t,A}\,(p_{t,A}+(-1+2\,\beta_1)\,p_{t,B}))+$$
$$2\,w_{10}\,(\alpha_1\,p_{t,A}-(-1+\beta_1)\,p_{t,B})\,(\alpha_2\,p_{T,A}+(1-\beta_2)\,p_{T,B})-2\,r\,w_8\,((-1+\alpha_1)\,p_{t,A}-\beta_1\,p_{t,B})$$
$$(\alpha_2\,p_{T,A}-(-1+\beta_2)\,p_{T,B})+2\,(-1+r)\,w_9\,(\alpha_1\,p_{t,A}-(-1+\beta_1)\,p_{t,B})\,((-1+\alpha_2)\,p_{T,A}-\beta_2\,p_{T,B}))+$$
$$(1-y)\,(2\,w_{10}\,(\alpha_1\,p_{t,A}-(-1+\beta_1)\,p_{t,B})\,(\alpha_2\,p_{T,A}+(1-\beta_2)\,p_{T,B})-$$
$$2\,(1-r)\,w_8\,((-1+\alpha_1)\,p_{t,A}-\beta_1\,p_{t,B})\,(\alpha_2\,p_{T,A}-(-1+\beta_2)\,p_{T,B})+$$
$$2\,w_6\,(\alpha_2\,p_{T,A}-(-1+\beta_2)\,p_{T,B})^2-2\,r\,w_9\,(\alpha_1\,p_{t,A}-(-1+\beta_1)\,p_{t,B})\,((-1+\alpha_2)\,p_{T,A}-\beta_2\,p_{T,B})+$$
$$w_5\,(-2\,\alpha_2^2\,p_{T,A}^2-2\,(-1+\beta_2)\,p_{T,B}\,(p_{T,A}+\beta_2\,p_{T,B})+2\,\alpha_2\,p_{T,A}\,(p_{T,A}+(-1+2\,\beta_2)\,p_{T,B}))))\,/$$
$$(2\,(w_3\,(\alpha_1\,p_{t,A}-(-1+\beta_1)\,p_{t,B})^2+w_1\,((-1+\alpha_1)\,p_{t,A}-\beta_1\,p_{t,B})^2+$$
$$w_2\,(-2\,\alpha_1^2\,p_{t,A}^2-2\,(-1+\beta_1)\,p_{t,B}\,(p_{t,A}+\beta_1\,p_{t,B})+2\,\alpha_1\,p_{t,A}\,(p_{t,A}+(-1+2\,\beta_1)\,p_{t,B}))+$$
$$w_4\,((-1+\alpha_1)\,p_{T,A}-\beta_1\,p_{T,B})^2+2\,w_{10}\,(\alpha_1\,p_{t,A}-(-1+\beta_1)\,p_{t,B})\,(\alpha_2\,p_{T,A}+(1-\beta_2)\,p_{T,B})-$$
$$2\,w_8\,((-1+\alpha_1)\,p_{t,A}-\beta_1\,p_{t,B})\,(\alpha_2\,p_{T,A}-(-1+\beta_2)\,p_{T,B})+$$
$$w_6\,(\alpha_2\,p_{T,A}-(-1+\beta_2)\,p_{T,B})^2-2\,w_9\,(\alpha_1\,p_{t,A}-(-1+\beta_1)\,p_{t,B})\,((-1+\alpha_2)\,p_{T,A}-\beta_2\,p_{T,B})+$$
$$2\,w_7\,((-1+\alpha_1)\,p_{t,A}-\beta_1\,p_{t,B})\,((-1+\alpha_2)\,p_{T,A}-\beta_2\,p_{T,B})+$$
$$w_5\,(-2\,\alpha_2^2\,p_{T,A}^2-2\,(-1+\beta_2)\,p_{T,B}\,(p_{T,A}+\beta_2\,p_{T,B})+2\,\alpha_2\,p_{T,A}\,(p_{T,A}+(-1+2\,\beta_2)\,p_{T,B}))))-$$
$$p_{T,B}\}\;/.\;\{p_{t,A}\to(1-p_{T,A}-p_{t,B}-p_{T,B})\};$$

$$\%\;/.\;\{w_1\to 1,\;w_2\to(1-h\,k),\;w_3\to(1-k),\;w_4\to 1,$$
$$w_5\to(1-h\,k),\;w_6\to(1-k),\;w_7\to 1,\;w_8\to(1-h\,k),\;w_9\to(1-h\,k),\;w_{10}\to(1-k)\};$$

$$\%\;/.\;\{z\to z\,\zeta,\;y\to y\,\zeta,\;\beta_2\to\beta_2\,\zeta,\;\alpha_1\to\alpha_1\,\zeta,$$
$$p_{T,A}\to(p_{T,A,0}+p_{T,A,1}\,\zeta),\;p_{t,B}\to(p_{t,B,0}+p_{t,B,1}\,\zeta),\;p_{T,B}\to(p_{T,B,0}+p_{T,B,1}\,\zeta)\};$$

```
Series[%, {ζ, 0, 1}];
```

$p_{T,A,0}=0$, $p_{t,B,0}=0$, $p_{T,B,0}=0$, is an equilibrium solution for the zeroth order terms. Plugging these into the first order terms and solving.

$$\{(-p_{T,A,1}+\frac{1}{2}\,(-(((2\,(-1+h\,k)\,(-1+r)\,(-1+\alpha_2)\,(-1+\beta_1)\,p_{t,B,0}\,p_{T,A,0}+$$

$$2\,(-1+\alpha_2)\,p_{T,A,0}\,(-1+p_{t,B,0}-\beta_1\,p_{t,B,0}+p_{T,A,0}+p_{T,B,0})-2\,(1-h\,k)\,r$$

$$(-1+p_{t,B,0}-\beta_1\,p_{t,B,0}+p_{T,A,0}+p_{T,B,0})\,(\alpha_2\,p_{T,A,0}+p_{T,B,0})+2\,(p_{T,A,0}+\beta_1\,p_{T,B,0})^2+$$

$$(1-h\,k)\,(-2\,\alpha_2^2\,p_{T,A,0}^2+2\,\alpha_2\,p_{T,A,0}\,(p_{T,A,0}-p_{T,B,0})+2\,p_{T,A,0}\,p_{T,B,0}))$$

$$(-2\,(1-k)\,(-1+\beta_1)\,p_{t,B,0}\,(-((-1+\beta_1)\,p_{t,B,1})-\alpha_1\,(-1+p_{t,B,0}+p_{T,A,0}+p_{T,B,0}))-$$

$$2\,(1-h\,k)\,(-((-1+\beta_1)\,p_{t,B,0}\,((-1+\alpha_2)\,p_{T,A,1}-\beta_2\,p_{T,B,0}))+$$

$$(-1+\alpha_2)\,p_{T,A,0}\,(-((-1+\beta_1)\,p_{t,B,1})-\alpha_1\,(-1+p_{t,B,0}+p_{T,A,0}+p_{T,B,0})))+(1-h\,k)$$

$$(-2\,\alpha_1\,(1-2\,p_{t,B,0}+2\,\beta_1\,p_{t,B,0}-p_{T,A,0}-p_{T,B,0})\,(-1+p_{t,B,0}+p_{T,A,0}+p_{T,B,0})-$$

$$2\,(-1+\beta_1)\,(p_{t,B,1}\,(1-p_{t,B,0}+\beta_1\,p_{t,B,0}-p_{T,A,0}-p_{T,B,0})+$$

$$p_{t,B,0}\,(-p_{t,B,1}+\beta_1\,p_{t,B,1}-p_{T,A,1}-p_{T,B,1})))+(1-h\,k)$$

$$(-4\,\alpha_2^2\,p_{T,A,0}\,p_{T,A,1}-2\,(-p_{T,B,0}\,(p_{T,A,1}+\beta_2\,p_{T,B,0})+p_{T,A,0}\,(\beta_2\,p_{T,B,0}-p_{T,B,1}))+$$

$$2\,\alpha_2\,(p_{T,A,1}\,(p_{T,A,0}-p_{T,B,0})+p_{T,A,0}\,(p_{T,A,1}+2\,\beta_2\,p_{T,B,0}-p_{T,B,1})))+$$

$$2\,(-1+p_{t,B,0}-\beta_1\,p_{t,B,0}+p_{T,A,0}+p_{T,B,0})\,(p_{t,B,1}-\beta_1\,p_{t,B,1}+p_{T,A,1}+$$

$$\alpha_1\,(1-p_{t,B,0}-p_{T,A,0}-p_{T,B,0})+p_{T,B,1})-2\,(-1+k)\,(\alpha_2\,p_{T,A,0}+p_{T,B,0})$$

$$(\alpha_2\,p_{T,A,1}-\beta_2\,p_{T,B,0}+p_{T,B,1})-2\,(p_{T,A,0}+\beta_1\,p_{T,B,0})\,(\alpha_1\,p_{T,A,0}-p_{T,A,1}-\beta_1\,p_{T,B,1})+$$

$$2\,((-1+p_{t,B,0}-\beta_1\,p_{t,B,0}+p_{T,A,0}+p_{T,B,0})\,((-1+\alpha_2)\,p_{T,A,1}-\beta_2\,p_{T,B,0})+(-1+\alpha_2)$$

$$p_{T,A,0}\,(p_{t,B,1}-\beta_1\,p_{t,B,1}+p_{T,A,1}+\alpha_1\,(1-p_{t,B,0}-p_{T,A,0}-p_{T,B,0})+p_{T,B,1}))+$$

$$2\,(1-k)\,((\alpha_2\,p_{T,A,0}+p_{T,B,0})\,(-((-1+\beta_1)\,p_{t,B,1})-\alpha_1\,(-1+p_{t,B,0}+p_{T,A,0}+p_{T,B,0}))-$$

$$(-1+\beta_1)\,p_{t,B,0}\,(\alpha_2\,p_{T,A,1}-\beta_2\,p_{T,B,0}+p_{T,B,1}))-2\,(1-h\,k)\,((\alpha_2\,p_{T,A,0}+p_{T,B,0})$$

$$(p_{t,B,1}-\beta_1\,p_{t,B,1}+p_{T,A,1}+\alpha_1\,(1-p_{t,B,0}-p_{T,A,0}-p_{T,B,0})+p_{T,B,1})+$$

$$(-1+p_{t,B,0}-\beta_1\,p_{t,B,0}+p_{T,A,0}+p_{T,B,0})\,(\alpha_2\,p_{T,A,1}-\beta_2\,p_{T,B,0}+p_{T,B,1}))))\,/$$

$$(-((-1+k)\,(-1+\beta_1)^2\,p_{t,B,0}^2)-2\,(-1+h\,k)\,(-1+\alpha_2)\,(-1+\beta_1)\,p_{t,B,0}\,p_{T,A,0}+$$

$$2\,(-1+h\,k)\,(-1+\beta_1)\,p_{t,B,0}\,(1-p_{t,B,0}+\beta_1\,p_{t,B,0}-p_{T,A,0}-p_{T,B,0})+$$

$$2\,(-1+\alpha_2)\,p_{T,A,0}\,(-1+p_{t,B,0}-\beta_1\,p_{t,B,0}+p_{T,A,0}+p_{T,B,0})+$$

$$(-1+p_{t,B,0}-\beta_1\,p_{t,B,0}+p_{T,A,0}+p_{T,B,0})^2+2\,(-1+k)\,(-1+\beta_1)\,p_{t,B,0}\,(\alpha_2\,p_{T,A,0}+p_{T,B,0})-$$

$$2\,(1-h\,k)\,(-1+p_{t,B,0}-\beta_1\,p_{t,B,0}+p_{T,A,0}+p_{T,B,0})\,(\alpha_2\,p_{T,A,0}+p_{T,B,0})-$$

$$(-1+k)\,(\alpha_2\,p_{T,A,0}+p_{T,B,0})^2+(-p_{T,A,0}-\beta_1\,p_{T,B,0})^2+$$

$$(1-h\,k)\,(-2\,\alpha_2^2\,p_{T,A,0}^2+2\,\alpha_2\,p_{T,A,0}\,(p_{T,A,0}-p_{T,B,0})+2\,p_{T,A,0}\,p_{T,B,0}))^2)+$$

$$(z\,(-2\,(-1+h\,k)\,r\,(-1+\alpha_2)\,(-1+\beta_1)\,p_{t,B,0}\,p_{T,A,0}+2\,(-1+h\,k)\,(-1+\beta_1)\,p_{t,B,0}$$

$$(1-p_{t,B,0}+\beta_1\,p_{t,B,0}-p_{T,A,0}-p_{T,B,0})+2\,(-1+\alpha_2)\,p_{T,A,0}$$

$$(-1+p_{t,B,0}-\beta_1\,p_{t,B,0}+p_{T,A,0}+p_{T,B,0})+2\,(-1+p_{t,B,0}-\beta_1\,p_{t,B,0}+p_{T,A,0}+p_{T,B,0})^2-2$$

$$(1-h\,k)\,(1-r)\,(-1+p_{t,B,0}-\beta_1\,p_{t,B,0}+p_{T,A,0}+p_{T,B,0})\,(\alpha_2\,p_{T,A,0}+p_{T,B,0}))-$$

$$y\,(2\,(-1+h\,k)\,(-1+r)\,(-1+\alpha_2)\,(-1+\beta_1)\,p_{t,B,0}\,p_{T,A,0}+2\,(-1+\alpha_2)\,p_{T,A,0}$$

$$(-1+p_{t,B,0}-\beta_1\,p_{t,B,0}+p_{T,A,0}+p_{T,B,0})-2\,(1-h\,k)\,r$$

$$(-1+p_{t,B,0}-\beta_1\,p_{t,B,0}+p_{T,A,0}+p_{T,B,0})\,(\alpha_2\,p_{T,A,0}+p_{T,B,0})+2\,(p_{T,A,0}+\beta_1\,p_{T,B,0})^2+$$

$$(1-h\,k)\,(-2\,\alpha_2^2\,p_{T,A,0}^2+2\,\alpha_2\,p_{T,A,0}\,(p_{T,A,0}-p_{T,B,0})+2\,p_{T,A,0}\,p_{T,B,0}))+$$

$$2\,(1-h\,k)\,(-1+r)\,(-((-1+\beta_1)\,p_{t,B,0}\,((-1+\alpha_2)\,p_{T,A,1}-\beta_2\,p_{T,B,0}))+(-1+\alpha_2)$$

$$p_{T,A,0}\,(-((-1+\beta_1)\,p_{t,B,1})-\alpha_1\,(-1+p_{t,B,0}+p_{T,A,0}+p_{T,B,0})))+$$

$$(1 - h\,k)\,(-4\,\alpha_2^2\,p_{T,A,0}\,p_{T,A,1} - 2\,(-p_{T,B,0}\,(p_{T,A,1} + \beta_2\,p_{T,B,0}) + p_{T,A,0}\,(\beta_2\,p_{T,B,0} - p_{T,B,1})) +$$

$$2\,\alpha_2\,(p_{T,A,1}\,(p_{T,A,0} - p_{T,B,0}) + p_{T,A,0}\,(p_{T,A,1} + 2\,\beta_2\,p_{T,B,0} - p_{T,B,1}))) -$$

$$4\,(p_{T,A,0} + \beta_1\,p_{T,B,0})\,(\alpha_1\,p_{T,A,0} - p_{T,A,1} - \beta_1\,p_{T,B,1}) +$$

$$2\,((-1 + p_{t,B,0} - \beta_1\,p_{t,B,0} + p_{T,A,0} + p_{T,B,0})\,((-1 + \alpha_2)\,p_{T,A,1} - \beta_2\,p_{T,B,0}) + (-1 + \alpha_2)\,p_{T,A,0}$$

$$(p_{t,B,1} - \beta_1\,p_{t,B,1} + p_{T,A,1} + \alpha_1\,(1 - p_{t,B,0} - p_{T,A,0} - p_{T,B,0}) + p_{T,B,1})) - 2\,(1 - h\,k)\,r$$

$$((\alpha_2\,p_{T,A,0} + p_{T,B,0})\,(p_{t,B,1} - \beta_1\,p_{t,B,1} + p_{T,A,1} + \alpha_1\,(1 - p_{t,B,0} - p_{T,A,0} - p_{T,B,0}) + p_{T,B,1}) +$$

$$(-1 + p_{t,B,0} - \beta_1\,p_{t,B,0} + p_{T,A,0} + p_{T,B,0})\,(\alpha_2\,p_{T,A,1} - \beta_2\,p_{T,B,0} + p_{T,B,1}))) \,/$$

$$(-((-1 + k)\,(-1 + \beta_1)^2\,p_{t,B,0}^2) - 2\,(-1 + h\,k)\,(-1 + \alpha_2)\,(-1 + \beta_1)\,p_{t,B,0}\,p_{T,A,0} +$$

$$2\,(-1 + h\,k)\,(-1 + \beta_1)\,p_{t,B,0}\,(1 - p_{t,B,0} + \beta_1\,p_{t,B,0} - p_{T,A,0} - p_{T,B,0}) +$$

$$2\,(-1 + \alpha_2)\,p_{T,A,0}\,(-1 + p_{t,B,0} - \beta_1\,p_{t,B,0} + p_{T,A,0} + p_{T,B,0}) +$$

$$(-1 + p_{t,B,0} - \beta_1\,p_{t,B,0} + p_{T,A,0} + p_{T,B,0})^2 + 2\,(-1 + k)\,(-1 + \beta_1)\,p_{t,B,0}\,(\alpha_2\,p_{T,A,0} + p_{T,B,0}) -$$

$$2\,(1 - h\,k)\,(-1 + p_{t,B,0} - \beta_1\,p_{t,B,0} + p_{T,A,0} + p_{T,B,0})\,(\alpha_2\,p_{T,A,0} + p_{T,B,0}) -$$

$$(-1 + k)\,(\alpha_2\,p_{T,A,0} + p_{T,B,0})^2 + (-p_{T,A,0} - \beta_1\,p_{T,B,0})^2 +$$

$$(1 - h\,k)\,(-2\,\alpha_2^2\,p_{T,A,0}^2 + 2\,\alpha_2\,p_{T,A,0}\,(p_{T,A,0} - p_{T,B,0}) + 2\,p_{T,A,0}\,p_{T,B,0})))) == 0,$$

$$(-p_{t,B,1} + \frac{1}{2}\,(-((((-2\,(-1 + k)\,(-1 + \beta_1)^2\,p_{t,B,0}^2 + 2\,(-1 + h\,k)\,(-1 + r)\,(-1 + \alpha_2)\,(-1 + \beta_1)\,p_{t,B,0}\,p_{T,A,0} +$$

$$2\,(-1 + h\,k)\,(-1 + \beta_1)\,p_{t,B,0}\,(1 - p_{t,B,0} + \beta_1\,p_{t,B,0} - p_{T,A,0} - p_{T,B,0}) +$$

$$2\,(-1 + k)\,(-1 + \beta_1)\,p_{t,B,0}\,(\alpha_2\,p_{T,A,0} + p_{T,B,0}) -$$

$$2\,(1 - h\,k)\,r\,(-1 + p_{t,B,0} - \beta_1\,p_{t,B,0} + p_{T,A,0} + p_{T,B,0})\,(\alpha_2\,p_{T,A,0} + p_{T,B,0}))$$

$$(-2\,(1 - k)\,(-1 + \beta_1)\,p_{t,B,0}\,(-((-1 + \beta_1)\,p_{t,B,1}) - \alpha_1\,(-1 + p_{t,B,0} + p_{T,A,0} + p_{T,B,0})) -$$

$$2\,(1 - h\,k)\,(-((-1 + \beta_1)\,p_{t,B,0}\,((-1 + \alpha_2)\,p_{T,A,1} - \beta_2\,p_{T,B,0})) +$$

$$(-1 + \alpha_2)\,p_{T,A,0}\,(-((-1 + \beta_1)\,p_{t,B,1}) - \alpha_1\,(-1 + p_{t,B,0} + p_{T,A,0} + p_{T,B,0}))) + (1 - h\,k)$$

$$(-2\,\alpha_1\,(1 - 2\,p_{t,B,0} + 2\,\beta_1\,p_{t,B,0} - p_{T,A,0} - p_{T,B,0})\,(-1 + p_{t,B,0} + p_{T,A,0} + p_{T,B,0}) -$$

$$2\,(-1 + \beta_1)\,(p_{t,B,1}\,(1 - p_{t,B,0} + \beta_1\,p_{t,B,0} - p_{T,A,0} - p_{T,B,0}) +$$

$$p_{t,B,0}\,(-p_{t,B,1} + \beta_1\,p_{t,B,1} - p_{T,A,1} - p_{T,B,1}))) + (1 - h\,k)$$

$$(-4\,\alpha_2^2\,p_{T,A,0}\,p_{T,A,1} - 2\,(-p_{T,B,0}\,(p_{T,A,1} + \beta_2\,p_{T,B,0}) + p_{T,A,0}\,(\beta_2\,p_{T,B,0} - p_{T,B,1})) +$$

$$2\,\alpha_2\,(p_{T,A,1}\,(p_{T,A,0} - p_{T,B,0}) + p_{T,A,0}\,(p_{T,A,1} + 2\,\beta_2\,p_{T,B,0} - p_{T,B,1}))) +$$

$$2\,(-1 + p_{t,B,0} - \beta_1\,p_{t,B,0} + p_{T,A,0} + p_{T,B,0})\,(p_{t,B,1} - \beta_1\,p_{t,B,1} + p_{T,A,1} +$$

$$\alpha_1\,(1 - p_{t,B,0} - p_{T,A,0} - p_{T,B,0}) + p_{T,B,1}) - 2\,(-1 + k)\,(\alpha_2\,p_{T,A,0} + p_{T,B,0})$$

$$(\alpha_2\,p_{T,A,1} - \beta_2\,p_{T,B,0} + p_{T,B,1}) - 2\,(p_{T,A,0} + \beta_1\,p_{T,B,0})\,(\alpha_1\,p_{T,A,0} - p_{T,A,1} - \beta_1\,p_{T,B,1}) +$$

$$2\,((-1 + p_{t,B,0} - \beta_1\,p_{t,B,0} + p_{T,A,0} + p_{T,B,0})\,((-1 + \alpha_2)\,p_{T,A,1} - \beta_2\,p_{T,B,0}) + (-1 + \alpha_2)$$

$$p_{T,A,0}\,(p_{t,B,1} - \beta_1\,p_{t,B,1} + p_{T,A,1} + \alpha_1\,(1 - p_{t,B,0} - p_{T,A,0} - p_{T,B,0}) + p_{T,B,1})) +$$

$$2\,(1 - k)\,((\alpha_2\,p_{T,A,0} + p_{T,B,0})\,(-((-1 + \beta_1)\,p_{t,B,1}) - \alpha_1\,(-1 + p_{t,B,0} + p_{T,A,0} + p_{T,B,0})) -$$

$$(-1 + \beta_1)\,p_{t,B,0}\,(\alpha_2\,p_{T,A,1} - \beta_2\,p_{T,B,0} + p_{T,B,1})) - 2\,(1 - h\,k)\,((\alpha_2\,p_{T,A,0} + p_{T,B,0})$$

$$(p_{t,B,1} - \beta_1\,p_{t,B,1} + p_{T,A,1} + \alpha_1\,(1 - p_{t,B,0} - p_{T,A,0} - p_{T,B,0}) + p_{T,B,1}) +$$

$$(-1 + p_{t,B,0} - \beta_1\,p_{t,B,0} + p_{T,A,0} + p_{T,B,0})\,(\alpha_2\,p_{T,A,1} - \beta_2\,p_{T,B,0} + p_{T,B,1})))) \,/$$

$$(-((-1 + k)\,(-1 + \beta_1)^2\,p_{t,B,0}^2) - 2\,(-1 + h\,k)\,(-1 + \alpha_2)\,(-1 + \beta_1)\,p_{t,B,0}\,p_{T,A,0} +$$

$$2\left(-1+h\,k\right)(-1+\beta_1)\,p_{t,B,0}\left(1-p_{t,B,0}+\beta_1\,p_{t,B,0}-p_{T,A,0}-p_{T,B,0}\right)+$$
$$2\,(-1+\alpha_2)\,p_{T,A,0}\left(-1+p_{t,B,0}-\beta_1\,p_{t,B,0}+p_{T,A,0}+p_{T,B,0}\right)+$$
$$\left(-1+p_{t,B,0}-\beta_1\,p_{t,B,0}+p_{T,A,0}+p_{T,B,0}\right)^2+2\,(-1+k)\,(-1+\beta_1)\,p_{t,B,0}\left(\alpha_2\,p_{T,A,0}+p_{T,B,0}\right)-$$
$$2\left(1-h\,k\right)\left(-1+p_{t,B,0}-\beta_1\,p_{t,B,0}+p_{T,A,0}+p_{T,B,0}\right)\left(\alpha_2\,p_{T,A,0}+p_{T,B,0}\right)-$$
$$(-1+k)\left(\alpha_2\,p_{T,A,0}+p_{T,B,0}\right)^2+\left(-p_{T,A,0}-\beta_1\,p_{T,B,0}\right)^2+$$
$$\left(1-h\,k\right)\left(-2\,\alpha_2^2\,p_{T,A,0}^2+2\,\alpha_2\,p_{T,A,0}\,(p_{T,A,0}-p_{T,B,0})+2\,p_{T,A,0}\,p_{T,B,0}\right)\Big)^2\Big)+$$
$$\Big(-4\,(1-k)\,(-1+\beta_1)\,p_{t,B,0}\left(-\left((-1+\beta_1)\,p_{t,B,1}\right)-\alpha_1\,(-1+p_{t,B,0}+p_{T,A,0}+p_{T,B,0})\right)-$$
$$z\Big(-2\,(-1+k)\,(-1+\beta_1)^2\,p_{t,B,0}^2+2\left(-1+h\,k\right)(-1+r)\,(-1+\alpha_2)\,(-1+\beta_1)\,p_{t,B,0}$$
$$p_{T,A,0}+2\left(-1+h\,k\right)(-1+\beta_1)\,p_{t,B,0}\left(1-p_{t,B,0}+\beta_1\,p_{t,B,0}-p_{T,A,0}-p_{T,B,0}\right)+2$$
$$(-1+k)\,(-1+\beta_1)\,p_{t,B,0}\left(\alpha_2\,p_{T,A,0}+p_{T,B,0}\right)-2\left(1-h\,k\right)r$$
$$\left(-1+p_{t,B,0}-\beta_1\,p_{t,B,0}+p_{T,A,0}+p_{T,B,0}\right)\left(\alpha_2\,p_{T,A,0}+p_{T,B,0}\right)\Big)+$$
$$y\Big(-2\left(-1+h\,k\right)r\,(-1+\alpha_2)\,(-1+\beta_1)\,p_{t,B,0}\,p_{T,A,0}+2\,(-1+k)\,(-1+\beta_1)\,p_{t,B,0}$$
$$\left(\alpha_2\,p_{T,A,0}+p_{T,B,0}\right)-2\left(1-h\,k\right)(1-r)\left(-1+p_{t,B,0}-\beta_1\,p_{t,B,0}+p_{T,A,0}+p_{T,B,0}\right)$$
$$\left(\alpha_2\,p_{T,A,0}+p_{T,B,0}\right)-2\,(-1+k)\left(\alpha_2\,p_{T,A,0}+p_{T,B,0}\right)^2+\left(1-h\,k\right)$$
$$\left(-2\,\alpha_2^2\,p_{T,A,0}^2+2\,\alpha_2\,p_{T,A,0}\,(p_{T,A,0}-p_{T,B,0})+2\,p_{T,A,0}\,p_{T,B,0}\right)\Big)+$$
$$2\left(1-h\,k\right)(-1+r)\Big(-\left((-1+\beta_1)\,p_{t,B,0}\left((-1+\alpha_2)\,p_{T,A,1}-\beta_2\,p_{T,B,0}\right)\right)+(-1+\alpha_2)$$
$$p_{T,A,0}\left(-\left((-1+\beta_1)\,p_{t,B,1}\right)-\alpha_1\,(-1+p_{t,B,0}+p_{T,A,0}+p_{T,B,0})\right)\Big)+$$
$$\left(1-h\,k\right)\Big(-2\,\alpha_1\left(1-2\,p_{t,B,0}+2\,\beta_1\,p_{t,B,0}-p_{T,A,0}-p_{T,B,0}\right)(-1+p_{t,B,0}+p_{T,A,0}+p_{T,B,0})-$$
$$2\,(-1+\beta_1)\Big(p_{t,B,1}\left(1-p_{t,B,0}+\beta_1\,p_{t,B,0}-p_{T,A,0}-p_{T,B,0}\right)+$$
$$p_{t,B,0}\left(-p_{t,B,1}+\beta_1\,p_{t,B,1}-p_{T,A,1}-p_{T,B,1}\right)\Big)\Big)+$$
$$2\,(1-k)\Big(\left(\alpha_2\,p_{T,A,0}+p_{T,B,0}\right)\left(-\left((-1+\beta_1)\,p_{t,B,1}\right)-\alpha_1\,(-1+p_{t,B,0}+p_{T,A,0}+p_{T,B,0})\right)-$$
$$(-1+\beta_1)\,p_{t,B,0}\left(\alpha_2\,p_{T,A,1}-\beta_2\,p_{T,B,0}+p_{T,B,1}\right)\Big)-2\left(1-h\,k\right)r$$
$$\Big(\left(\alpha_2\,p_{T,A,0}+p_{T,B,0}\right)\left(p_{t,B,1}-\beta_1\,p_{t,B,1}+p_{T,A,1}+\alpha_1\,(1-p_{t,B,0}-p_{T,A,0}-p_{T,B,0})+p_{T,B,1}\right)+$$
$$\left(-1+p_{t,B,0}-\beta_1\,p_{t,B,0}+p_{T,A,0}+p_{T,B,0}\right)\left(\alpha_2\,p_{T,A,1}-\beta_2\,p_{T,B,0}+p_{T,B,1}\right)\Big)\Big)\Big/$$
$$\Big(-\left((-1+k)\,(-1+\beta_1)^2\,p_{t,B,0}^2\right)-2\left(-1+h\,k\right)(-1+\alpha_2)\,(-1+\beta_1)\,p_{t,B,0}\,p_{T,A,0}+$$
$$2\left(-1+h\,k\right)(-1+\beta_1)\,p_{t,B,0}\left(1-p_{t,B,0}+\beta_1\,p_{t,B,0}-p_{T,A,0}-p_{T,B,0}\right)+$$
$$2\,(-1+\alpha_2)\,p_{T,A,0}\left(-1+p_{t,B,0}-\beta_1\,p_{t,B,0}+p_{T,A,0}+p_{T,B,0}\right)+$$
$$\left(-1+p_{t,B,0}-\beta_1\,p_{t,B,0}+p_{T,A,0}+p_{T,B,0}\right)^2+2\,(-1+k)\,(-1+\beta_1)\,p_{t,B,0}\left(\alpha_2\,p_{T,A,0}+p_{T,B,0}\right)-$$
$$2\left(1-h\,k\right)\left(-1+p_{t,B,0}-\beta_1\,p_{t,B,0}+p_{T,A,0}+p_{T,B,0}\right)\left(\alpha_2\,p_{T,A,0}+p_{T,B,0}\right)-$$
$$(-1+k)\left(\alpha_2\,p_{T,A,0}+p_{T,B,0}\right)^2+\left(-p_{T,A,0}-\beta_1\,p_{T,B,0}\right)^2+$$
$$\left(1-h\,k\right)\left(-2\,\alpha_2^2\,p_{T,A,0}^2+2\,\alpha_2\,p_{T,A,0}\,(p_{T,A,0}-p_{T,B,0})+2\,p_{T,A,0}\,p_{T,B,0}\right)\Big)\Big)\Big)\ ==\ 0,$$
$$\Big(-p_{T,B,1}+\frac{1}{2}\Big(-\Big(\Big(\Big(-2\left(-1+h\,k\right)r\,(-1+\alpha_2)\,(-1+\beta_1)\,p_{t,B,0}\,p_{T,A,0}+2\,(-1+k)\,(-1+\beta_1)\,p_{t,B,0}$$
$$\left(\alpha_2\,p_{T,A,0}+p_{T,B,0}\right)-2\left(1-h\,k\right)(1-r)\left(-1+p_{t,B,0}-\beta_1\,p_{t,B,0}+p_{T,A,0}+p_{T,B,0}\right)$$
$$\left(\alpha_2\,p_{T,A,0}+p_{T,B,0}\right)-2\,(-1+k)\left(\alpha_2\,p_{T,A,0}+p_{T,B,0}\right)^2+$$
$$\left(1-h\,k\right)\left(-2\,\alpha_2^2\,p_{T,A,0}^2+2\,\alpha_2\,p_{T,A,0}\,(p_{T,A,0}-p_{T,B,0})+2\,p_{T,A,0}\,p_{T,B,0}\right)\Big)$$

$$(-2 (1-k) (-1+\beta_1) p_{t,B,0} (-((-1+\beta_1) p_{t,B,1}) - \alpha_1 (-1+p_{t,B,0}+p_{T,A,0}+p_{T,B,0})) -$$
$$2 (1-h k) (-((-1+\beta_1) p_{t,B,0} ((-1+\alpha_2) p_{T,A,1} - \beta_2 p_{T,B,0})) +$$
$$(-1+\alpha_2) p_{T,A,0} (-((-1+\beta_1) p_{t,B,1}) - \alpha_1 (-1+p_{t,B,0}+p_{T,A,0}+p_{T,B,0}))) + (1-h k)$$
$$(-2 \alpha_1 (1-2 p_{t,B,0}+2 \beta_1 p_{t,B,0}-p_{T,A,0}-p_{T,B,0}) (-1+p_{t,B,0}+p_{T,A,0}+p_{T,B,0}) -$$
$$2 (-1+\beta_1) (p_{t,B,1} (1-p_{t,B,0}+\beta_1 p_{t,B,0}-p_{T,A,0}-p_{T,B,0}) +$$
$$p_{t,B,0} (-p_{t,B,1}+\beta_1 p_{t,B,1}-p_{T,A,1}-p_{T,B,1}))) + (1-h k)$$
$$(-4 \alpha_2^2 p_{T,A,0} p_{T,A,1} - 2 (-p_{T,B,0} (p_{T,A,1}+\beta_2 p_{T,B,0}) + p_{T,A,0} (\beta_2 p_{T,B,0}-p_{T,B,1})) +$$
$$2 \alpha_2 (p_{T,A,1} (p_{T,A,0}-p_{T,B,0}) + p_{T,A,0} (p_{T,A,1}+2 \beta_2 p_{T,B,0}-p_{T,B,1}))) +$$
$$2 (-1+p_{t,B,0}-\beta_1 p_{t,B,0}+p_{T,A,0}+p_{T,B,0}) (p_{t,B,1}-\beta_1 p_{t,B,1}+p_{T,A,1} +$$
$$\alpha_1 (1-p_{t,B,0}-p_{T,A,0}-p_{T,B,0}) + p_{T,B,1}) - 2 (-1+k) (\alpha_2 p_{T,A,0}+p_{T,B,0})$$
$$(\alpha_2 p_{T,A,1}-\beta_2 p_{T,B,0}+p_{T,B,1}) - 2 (p_{T,A,0}+\beta_1 p_{T,B,0}) (\alpha_1 p_{T,A,0}-p_{T,A,1}-\beta_1 p_{T,B,1}) +$$
$$2 ((-1+p_{t,B,0}-\beta_1 p_{t,B,0}+p_{T,A,0}+p_{T,B,0}) ((-1+\alpha_2) p_{T,A,1}-\beta_2 p_{T,B,0}) + (-1+\alpha_2)$$
$$p_{T,A,0} (p_{t,B,1}-\beta_1 p_{t,B,1}+p_{T,A,1}+\alpha_1 (1-p_{t,B,0}-p_{T,A,0}-p_{T,B,0})+p_{T,B,1})) +$$
$$2 (1-k) ((\alpha_2 p_{T,A,0}+p_{T,B,0}) (-((-1+\beta_1) p_{t,B,1}) - \alpha_1 (-1+p_{t,B,0}+p_{T,A,0}+p_{T,B,0})) -$$
$$(-1+\beta_1) p_{t,B,0} (\alpha_2 p_{T,A,1}-\beta_2 p_{T,B,0}+p_{T,B,1})) - 2 (1-h k) ((\alpha_2 p_{T,A,0}+p_{T,B,0})$$
$$(p_{t,B,1}-\beta_1 p_{t,B,1}+p_{T,A,1}+\alpha_1 (1-p_{t,B,0}-p_{T,A,0}-p_{T,B,0})+p_{T,B,1}) +$$
$$(-1+p_{t,B,0}-\beta_1 p_{t,B,0}+p_{T,A,0}+p_{T,B,0}) (\alpha_2 p_{T,A,1}-\beta_2 p_{T,B,0}+p_{T,B,1})))) /$$
$$(-((-1+k) (-1+\beta_1)^2 p_{t,B,0}^2) - 2 (-1+h k) (-1+\alpha_2) (-1+\beta_1) p_{t,B,0} p_{T,A,0} +$$
$$2 (-1+h k) (-1+\beta_1) p_{t,B,0} (1-p_{t,B,0}+\beta_1 p_{t,B,0}-p_{T,A,0}-p_{T,B,0}) +$$
$$2 (-1+\alpha_2) p_{T,A,0} (-1+p_{t,B,0}-\beta_1 p_{t,B,0}+p_{T,A,0}+p_{T,B,0}) +$$
$$(-1+p_{t,B,0}-\beta_1 p_{t,B,0}+p_{T,A,0}+p_{T,B,0})^2 + 2 (-1+k) (-1+\beta_1) p_{t,B,0} (\alpha_2 p_{T,A,0}+p_{T,B,0}) -$$
$$2 (1-h k) (-1+p_{t,B,0}-\beta_1 p_{t,B,0}+p_{T,A,0}+p_{T,B,0}) (\alpha_2 p_{T,A,0}+p_{T,B,0}) -$$
$$(-1+k) (\alpha_2 p_{T,A,0}+p_{T,B,0})^2 + (-p_{T,A,0}-\beta_1 p_{T,B,0})^2 +$$
$$(1-h k) (-2 \alpha_2^2 p_{T,A,0}^2 + 2 \alpha_2 p_{T,A,0} (p_{T,A,0}-p_{T,B,0}) + 2 p_{T,A,0} p_{T,B,0}))^2) +$$
$$(z (-2 (-1+k) (-1+\beta_1)^2 p_{t,B,0}^2 + 2 (-1+h k) (-1+r) (-1+\alpha_2) (-1+\beta_1) p_{t,B,0} p_{T,A,0} + 2$$
$$(-1+h k) (-1+\beta_1) p_{t,B,0} (1-p_{t,B,0}+\beta_1 p_{t,B,0}-p_{T,A,0}-p_{T,B,0}) + 2$$
$$(-1+k) (-1+\beta_1) p_{t,B,0} (\alpha_2 p_{T,A,0}+p_{T,B,0}) - 2 (1-h k) r$$
$$(-1+p_{t,B,0}-\beta_1 p_{t,B,0}+p_{T,A,0}+p_{T,B,0}) (\alpha_2 p_{T,A,0}+p_{T,B,0})) -$$
$$y (-2 (-1+h k) r (-1+\alpha_2) (-1+\beta_1) p_{t,B,0} p_{T,A,0} + 2 (-1+k) (-1+\beta_1) p_{t,B,0}$$
$$(\alpha_2 p_{T,A,0}+p_{T,B,0}) - 2 (1-h k) (1-r) (-1+p_{t,B,0}-\beta_1 p_{t,B,0}+p_{T,A,0}+p_{T,B,0})$$
$$(\alpha_2 p_{T,A,0}+p_{T,B,0}) - 2 (-1+k) (\alpha_2 p_{T,A,0}+p_{T,B,0})^2 + (1-h k)$$
$$(-2 \alpha_2^2 p_{T,A,0}^2 + 2 \alpha_2 p_{T,A,0} (p_{T,A,0}-p_{T,B,0}) + 2 p_{T,A,0} p_{T,B,0})) -$$
$$2 (1-h k) r (-((-1+\beta_1) p_{t,B,0} ((-1+\alpha_2) p_{T,A,1}-\beta_2 p_{T,B,0})) + (-1+\alpha_2) p_{T,A,0}$$
$$(-((-1+\beta_1) p_{t,B,1}) - \alpha_1 (-1+p_{t,B,0}+p_{T,A,0}+p_{T,B,0}))) +$$
$$(1-h k) (-4 \alpha_2^2 p_{T,A,0} p_{T,A,1} - 2 (-p_{T,B,0} (p_{T,A,1}+\beta_2 p_{T,B,0}) + p_{T,A,0} (\beta_2 p_{T,B,0}-p_{T,B,1})) +$$
$$2 \alpha_2 (p_{T,A,1} (p_{T,A,0}-p_{T,B,0}) + p_{T,A,0} (p_{T,A,1}+2 \beta_2 p_{T,B,0}-p_{T,B,1}))) -$$
$$4 (-1+k) (\alpha_2 p_{T,A,0}+p_{T,B,0}) (\alpha_2 p_{T,A,1}-\beta_2 p_{T,B,0}+p_{T,B,1}) +$$

$$2\,(1-k)\,((\alpha_2\,p_{T,A,0}+p_{T,B,0})\,(-((-1+\beta_1)\,p_{t,B,1})-\alpha_1\,(-1+p_{t,B,0}+p_{T,A,0}+p_{T,B,0}))-$$
$$(-1+\beta_1)\,p_{t,B,0}\,(\alpha_2\,p_{T,A,1}-\beta_2\,p_{T,B,0}+p_{T,B,1}))-2\,(1-h\,k)\,(1-r)$$
$$((\alpha_2\,p_{T,A,0}+p_{T,B,0})\,(p_{t,B,1}-\beta_1\,p_{t,B,1}+p_{T,A,1}+\alpha_1\,(1-p_{t,B,0}-p_{T,A,0}-p_{T,B,0})+p_{T,B,1})+$$
$$(-1+p_{t,B,0}-\beta_1\,p_{t,B,0}+p_{T,A,0}+p_{T,B,0})\,(\alpha_2\,p_{T,A,1}-\beta_2\,p_{T,B,0}+p_{T,B,1})))\,/$$
$$(-((-1+k)\,(-1+\beta_1)^2\,p_{t,B,0}^2)-2\,(-1+h\,k)\,(-1+\alpha_2)\,(-1+\beta_1)\,p_{t,B,0}\,p_{T,A,0}+$$
$$2\,(-1+h\,k)\,(-1+\beta_1)\,p_{t,B,0}\,(1-p_{t,B,0}+\beta_1\,p_{t,B,0}-p_{T,A,0}-p_{T,B,0})+$$
$$2\,(-1+\alpha_2)\,p_{T,A,0}\,(-1+p_{t,B,0}-\beta_1\,p_{t,B,0}+p_{T,A,0}+p_{T,B,0})+$$
$$(-1+p_{t,B,0}-\beta_1\,p_{t,B,0}+p_{T,A,0}+p_{T,B,0})^2+2\,(-1+k)\,(-1+\beta_1)\,p_{t,B,0}\,(\alpha_2\,p_{T,A,0}+p_{T,B,0})-$$
$$2\,(1-h\,k)\,(-1+p_{t,B,0}-\beta_1\,p_{t,B,0}+p_{T,A,0}+p_{T,B,0})\,(\alpha_2\,p_{T,A,0}+p_{T,B,0})-$$
$$(-1+k)\,(\alpha_2\,p_{T,A,0}+p_{T,B,0})^2+(-p_{T,A,0}-\beta_1\,p_{T,B,0})^2+$$
$$(1-h\,k)\,(-2\,\alpha_2^2\,p_{T,A,0}^2+2\,\alpha_2\,p_{T,A,0}\,(p_{T,A,0}-p_{T,B,0})+2\,p_{T,A,0}\,p_{T,B,0})))) ==$$
$$0\}\;/.\;\{p_{T,A,0}\to 0,\;p_{t,B,0}\to 0,\;p_{T,B,0}\to 0\};$$

$$\text{Solve}[\%,\,\{p_{T,A,1},\,p_{t,B,1},\,p_{T,B,1}\}]$$

Out[ ]=
$$\left\{\left\{p_{T,A,1}\to-\frac{-h\,k\,z-r\,z+h\,k\,r\,z}{h\,k\,\alpha_2},\;p_{t,B,1}\to-\frac{r\,z-h\,k\,r\,z+h\,k\,\alpha_1-h^2\,k^2\,\alpha_1}{h\,k\,(-h\,k-\beta_1+h\,k\,\beta_1)},\;p_{T,B,1}\to\frac{(-1+h\,k)\,(-z+r\,z)}{h\,k}\right\}\right\}$$

After simplifying and writing in terms of (epi)allele frequencies gives eq. 1 from File S3.

A3 - Calculating Eigenvalues

$$\text{ResourceFunction}[\text{"JacobianMatrix"}][$$
$$\{(z\,(-2\,(1-h\,k)\,(1-r)\,(\alpha_2\,p_{T,A}-(-1+\beta_2)\,p_{T,B})\,(-\beta_1\,p_{t,B}-(-1+\alpha_1)\,(-1+p_{t,B}+p_{T,A}+p_{T,B}))+$$
$$2\,((-1+\alpha_2)\,p_{T,A}-\beta_2\,p_{T,B})\,(-\beta_1\,p_{t,B}-(-1+\alpha_1)\,(-1+p_{t,B}+p_{T,A}+p_{T,B}))+$$
$$2\,(\beta_1\,p_{t,B}+(-1+\alpha_1)\,(-1+p_{t,B}+p_{T,A}+p_{T,B}))^2-$$
$$2\,(1-h\,k)\,r\,((-1+\alpha_2)\,p_{T,A}-\beta_2\,p_{T,B})\,(-((-1+\beta_1)\,p_{t,B})-\alpha_1\,(-1+p_{t,B}+p_{T,A}+p_{T,B}))+$$
$$(1-h\,k)\,(-2\,(-1+\beta_1)\,p_{t,B}\,(1+(-1+\beta_1)\,p_{t,B}-p_{T,A}-p_{T,B})-2\,\alpha_1\,(1+2\,(-1+\beta_1)\,p_{t,B}-p_{T,A}-p_{T,B})$$
$$(-1+p_{t,B}+p_{T,A}+p_{T,B})-2\,\alpha_1^2\,(-1+p_{t,B}+p_{T,A}+p_{T,B})^2))+(1-y)\,(2\,((-1+\alpha_1)\,p_{T,A}-\beta_1\,p_{T,B})^2-$$
$$2\,(1-h\,k)\,r\,(\alpha_2\,p_{T,A}-(-1+\beta_2)\,p_{T,B})\,(-\beta_1\,p_{t,B}-(-1+\alpha_1)\,(-1+p_{t,B}+p_{T,A}+p_{T,B}))+$$
$$2\,((-1+\alpha_2)\,p_{T,A}-\beta_2\,p_{T,B})\,(-\beta_1\,p_{t,B}-(-1+\alpha_1)\,(-1+p_{t,B}+p_{T,A}+p_{T,B}))+$$
$$2\,(1-h\,k)\,(-1+r)\,((-1+\alpha_2)\,p_{T,A}-\beta_2\,p_{T,B})\,(-((-1+\beta_1)\,p_{t,B})-\alpha_1\,(-1+p_{t,B}+p_{T,A}+p_{T,B}))+$$
$$(1-h\,k)\,(-2\,\alpha_2^2\,p_{T,A}^2-2\,(-1+\beta_2)\,p_{T,B}\,(p_{T,A}+\beta_2\,p_{T,B})+2\,\alpha_2\,p_{T,A}\,(p_{T,A}+(-1+2\,\beta_2)\,p_{T,B}))))\,/$$
$$(2\,(((-1+\alpha_1)\,p_{T,A}-\beta_1\,p_{T,B})^2+(1-k)\,(\alpha_2\,p_{T,A}-(-1+\beta_2)\,p_{T,B})^2-2\,(1-h\,k)\,(\alpha_2\,p_{T,A}-(-1+\beta_2)\,p_{T,B})$$
$$(-\beta_1\,p_{t,B}-(-1+\alpha_1)\,(-1+p_{t,B}+p_{T,A}+p_{T,B}))+2\,((-1+\alpha_2)\,p_{T,A}-\beta_2\,p_{T,B})$$
$$(-\beta_1\,p_{t,B}-(-1+\alpha_1)\,(-1+p_{t,B}+p_{T,A}+p_{T,B}))+(\beta_1\,p_{t,B}+(-1+\alpha_1)\,(-1+p_{t,B}+p_{T,A}+p_{T,B}))^2+$$
$$2\,(1-k)\,(\alpha_2\,p_{T,A}+(1-\beta_2)\,p_{T,B})\,(-((-1+\beta_1)\,p_{t,B})-\alpha_1\,(-1+p_{t,B}+p_{T,A}+p_{T,B}))-$$
$$2\,(1-h\,k)\,((-1+\alpha_2)\,p_{T,A}-\beta_2\,p_{T,B})\,(-((-1+\beta_1)\,p_{t,B})-\alpha_1\,(-1+p_{t,B}+p_{T,A}+p_{T,B}))+$$
$$(1-k)\,((-1+\beta_1)\,p_{t,B}+\alpha_1\,(-1+p_{t,B}+p_{T,A}+p_{T,B}))^2+$$

$$(1 - h\,k)\,(-2\,(-1 + \beta_1)\,p_{t,B}\,(1 + (-1 + \beta_1)\,p_{t,B} - p_{T,A} - p_{T,B}) -$$

$$2\,\alpha_1\,(1 + 2\,(-1 + \beta_1)\,p_{t,B} - p_{T,A} - p_{T,B})\,(-1 + p_{t,B} + p_{T,A} + p_{T,B}) - 2\,\alpha_1^2\,(-1 + p_{t,B} + p_{T,A} + p_{T,B})^2) +$$

$$(1 - h\,k)\,(-2\,\alpha_2^2\,p_{T,A}^2 - 2\,(-1 + \beta_2)\,p_{T,B}\,(p_{T,A} + \beta_2\,p_{T,B}) + 2\,\alpha_2\,p_{T,A}\,(p_{T,A} + (-1 + 2\,\beta_2)\,p_{T,B})))),$$

$$((1 - z)\,(-2\,(1 - h\,k)\,r\,(\alpha_2\,p_{T,A} - (-1 + \beta_2)\,p_{T,B})\,(-\beta_1\,p_{t,B} - (-1 + \alpha_1)\,(-1 + p_{t,B} + p_{T,A} + p_{T,B})) +$$

$$2\,(1 - k)\,(\alpha_2\,p_{T,A} + (1 - \beta_2)\,p_{T,B})\,(-((-1 + \beta_1)\,p_{t,B}) - \alpha_1\,(-1 + p_{t,B} + p_{T,A} + p_{T,B})) +$$

$$2\,(1 - h\,k)\,(-1 + r)\,((-1 + \alpha_2)\,p_{T,A} - \beta_2\,p_{T,B})\,(-((-1 + \beta_1)\,p_{t,B}) - \alpha_1\,(-1 + p_{t,B} + p_{T,A} + p_{T,B})) +$$

$$2\,(1 - k)\,((-1 + \beta_1)\,p_{t,B} + \alpha_1\,(-1 + p_{t,B} + p_{T,A} + p_{T,B}))^2 +$$

$$(1 - h\,k)\,(-2\,(-1 + \beta_1)\,p_{t,B}\,(1 + (-1 + \beta_1)\,p_{t,B} - p_{T,A} - p_{T,B}) -$$

$$2\,\alpha_1\,(1 + 2\,(-1 + \beta_1)\,p_{t,B} - p_{T,A} - p_{T,B})\,(-1 + p_{t,B} + p_{T,A} + p_{T,B}) - 2\,\alpha_1^2\,(-1 + p_{t,B} + p_{T,A} + p_{T,B})^2)) +$$

$$y\,(2\,(1 - k)\,(\alpha_2\,p_{T,A} - (-1 + \beta_2)\,p_{T,B})^2 - 2\,(1 - h\,k)\,(1 - r)\,(\alpha_2\,p_{T,A} - (-1 + \beta_2)\,p_{T,B})$$

$$(-\beta_1\,p_{t,B} - (-1 + \alpha_1)\,(-1 + p_{t,B} + p_{T,A} + p_{T,B})) +$$

$$2\,(1 - k)\,(\alpha_2\,p_{T,A} + (1 - \beta_2)\,p_{T,B})\,(-((-1 + \beta_1)\,p_{t,B}) - \alpha_1\,(-1 + p_{t,B} + p_{T,A} + p_{T,B})) -$$

$$2\,(1 - h\,k)\,r\,((-1 + \alpha_2)\,p_{T,A} - \beta_2\,p_{T,B})\,(-((-1 + \beta_1)\,p_{t,B}) - \alpha_1\,(-1 + p_{t,B} + p_{T,A} + p_{T,B})) +$$

$$(1 - h\,k)\,(-2\,\alpha_2^2\,p_{T,A}^2 - 2\,(-1 + \beta_2)\,p_{T,B}\,(p_{T,A} + \beta_2\,p_{T,B}) + 2\,\alpha_2\,p_{T,A}\,(p_{T,A} + (-1 + 2\,\beta_2)\,p_{T,B})))) \,/$$

$$(2\,(((-1 + \alpha_1)\,p_{T,A} - \beta_1\,p_{T,B})^2 + (1 - k)\,(\alpha_2\,p_{T,A} - (-1 + \beta_2)\,p_{T,B})^2 - 2\,(1 - h\,k)\,(\alpha_2\,p_{T,A} - (-1 + \beta_2)\,p_{T,B})$$

$$(-\beta_1\,p_{t,B} - (-1 + \alpha_1)\,(-1 + p_{t,B} + p_{T,A} + p_{T,B})) + 2\,((-1 + \alpha_2)\,p_{T,A} - \beta_2\,p_{T,B})$$

$$(-\beta_1\,p_{t,B} - (-1 + \alpha_1)\,(-1 + p_{t,B} + p_{T,A} + p_{T,B})) + (\beta_1\,p_{t,B} + (-1 + \alpha_1)\,(-1 + p_{t,B} + p_{T,A} + p_{T,B}))^2 +$$

$$2\,(1 - k)\,(\alpha_2\,p_{T,A} + (1 - \beta_2)\,p_{T,B})\,(-((-1 + \beta_1)\,p_{t,B}) - \alpha_1\,(-1 + p_{t,B} + p_{T,A} + p_{T,B})) -$$

$$2\,(1 - h\,k)\,((-1 + \alpha_2)\,p_{T,A} - \beta_2\,p_{T,B})\,(-((-1 + \beta_1)\,p_{t,B}) - \alpha_1\,(-1 + p_{t,B} + p_{T,A} + p_{T,B})) +$$

$$(1 - k)\,((-1 + \beta_1)\,p_{t,B} + \alpha_1\,(-1 + p_{t,B} + p_{T,A} + p_{T,B}))^2 +$$

$$(1 - h\,k)\,(-2\,(-1 + \beta_1)\,p_{t,B}\,(1 + (-1 + \beta_1)\,p_{t,B} - p_{T,A} - p_{T,B}) -$$

$$2\,\alpha_1\,(1 + 2\,(-1 + \beta_1)\,p_{t,B} - p_{T,A} - p_{T,B})\,(-1 + p_{t,B} + p_{T,A} + p_{T,B}) - 2\,\alpha_1^2\,(-1 + p_{t,B} + p_{T,A} + p_{T,B})^2) +$$

$$(1 - h\,k)\,(-2\,\alpha_2^2\,p_{T,A}^2 - 2\,(-1 + \beta_2)\,p_{T,B}\,(p_{T,A} + \beta_2\,p_{T,B}) + 2\,\alpha_2\,p_{T,A}\,(p_{T,A} + (-1 + 2\,\beta_2)\,p_{T,B})))),$$

$$(z\,(-2\,(1 - h\,k)\,r\,(\alpha_2\,p_{T,A} - (-1 + \beta_2)\,p_{T,B})\,(-\beta_1\,p_{t,B} - (-1 + \alpha_1)\,(-1 + p_{t,B} + p_{T,A} + p_{T,B})) +$$

$$2\,(1 - k)\,(\alpha_2\,p_{T,A} + (1 - \beta_2)\,p_{T,B})\,(-((-1 + \beta_1)\,p_{t,B}) - \alpha_1\,(-1 + p_{t,B} + p_{T,A} + p_{T,B})) +$$

$$2\,(1 - h\,k)\,(-1 + r)\,((-1 + \alpha_2)\,p_{T,A} - \beta_2\,p_{T,B})\,(-((-1 + \beta_1)\,p_{t,B}) - \alpha_1\,(-1 + p_{t,B} + p_{T,A} + p_{T,B})) +$$

$$2\,(1 - k)\,((-1 + \beta_1)\,p_{t,B} + \alpha_1\,(-1 + p_{t,B} + p_{T,A} + p_{T,B}))^2 +$$

$$(1 - h\,k)\,(-2\,(-1 + \beta_1)\,p_{t,B}\,(1 + (-1 + \beta_1)\,p_{t,B} - p_{T,A} - p_{T,B}) -$$

$$2\,\alpha_1\,(1 + 2\,(-1 + \beta_1)\,p_{t,B} - p_{T,A} - p_{T,B})\,(-1 + p_{t,B} + p_{T,A} + p_{T,B}) - 2\,\alpha_1^2\,(-1 + p_{t,B} + p_{T,A} + p_{T,B})^2)) +$$

$$(1 - y)\,(2\,(1 - k)\,(\alpha_2\,p_{T,A} - (-1 + \beta_2)\,p_{T,B})^2 - 2\,(1 - h\,k)\,(1 - r)\,(\alpha_2\,p_{T,A} - (-1 + \beta_2)\,p_{T,B})$$

$$(-\beta_1\,p_{t,B} - (-1 + \alpha_1)\,(-1 + p_{t,B} + p_{T,A} + p_{T,B})) +$$

$$2\,(1 - k)\,(\alpha_2\,p_{T,A} + (1 - \beta_2)\,p_{T,B})\,(-((-1 + \beta_1)\,p_{t,B}) - \alpha_1\,(-1 + p_{t,B} + p_{T,A} + p_{T,B})) -$$

$$2\,(1 - h\,k)\,r\,((-1 + \alpha_2)\,p_{T,A} - \beta_2\,p_{T,B})\,(-((-1 + \beta_1)\,p_{t,B}) - \alpha_1\,(-1 + p_{t,B} + p_{T,A} + p_{T,B})) +$$

$$(1 - h\,k)\,(-2\,\alpha_2^2\,p_{T,A}^2 - 2\,(-1 + \beta_2)\,p_{T,B}\,(p_{T,A} + \beta_2\,p_{T,B}) + 2\,\alpha_2\,p_{T,A}\,(p_{T,A} + (-1 + 2\,\beta_2)\,p_{T,B})))) \,/$$

$$(2\,(((-1 + \alpha_1)\,p_{T,A} - \beta_1\,p_{T,B})^2 + (1 - k)\,(\alpha_2\,p_{T,A} - (-1 + \beta_2)\,p_{T,B})^2 - 2\,(1 - h\,k)\,(\alpha_2\,p_{T,A} - (-1 + \beta_2)\,p_{T,B})$$

$$(-\beta_1\,p_{t,B} - (-1 + \alpha_1)\,(-1 + p_{t,B} + p_{T,A} + p_{T,B})) + 2\,((-1 + \alpha_2)\,p_{T,A} - \beta_2\,p_{T,B})$$

$$\left(-\beta_1\, p_{t,B} - (-1+\alpha_1)\,(-1+p_{t,B}+p_{T,A}+p_{T,B})\right) + \left(\beta_1\, p_{t,B} + (-1+\alpha_1)\,(-1+p_{t,B}+p_{T,A}+p_{T,B})\right)^2 +$$
$$2\,(1-k)\left(\alpha_2\, p_{T,A} + (1-\beta_2)\, p_{T,B}\right)\left(-\left((-1+\beta_1)\, p_{t,B}\right) - \alpha_1\,(-1+p_{t,B}+p_{T,A}+p_{T,B})\right) -$$
$$2\left(1-h\,k\right)\left((-1+\alpha_2)\, p_{T,A} - \beta_2\, p_{T,B}\right)\left(-\left((-1+\beta_1)\, p_{t,B}\right) - \alpha_1\,(-1+p_{t,B}+p_{T,A}+p_{T,B})\right) +$$
$$(1-k)\left((-1+\beta_1)\, p_{t,B} + \alpha_1\,(-1+p_{t,B}+p_{T,A}+p_{T,B})\right)^2 +$$
$$\left(1-h\,k\right)\left(-2\,(-1+\beta_1)\, p_{t,B}\left(1+(-1+\beta_1)\, p_{t,B} - p_{T,A} - p_{T,B}\right) -\right.$$
$$\left. 2\,\alpha_1\left(1+2\,(-1+\beta_1)\, p_{t,B} - p_{T,A} - p_{T,B}\right)(-1+p_{t,B}+p_{T,A}+p_{T,B}) - 2\,\alpha_1^2\,(-1+p_{t,B}+p_{T,A}+p_{T,B})^2\right) +$$
$$\left(1-h\,k\right)\left(-2\,\alpha_2^2\, p_{T,A}^2 - 2\,(-1+\beta_2)\, p_{T,B}\left(p_{T,A}+\beta_2\, p_{T,B}\right) + 2\,\alpha_2\, p_{T,A}\left(p_{T,A}+\left(-1+2\,\beta_2\right) p_{T,B}\right)\right)\Big)\Big\},$$
$$\left\{p_{T,A},\ p_{t,B},\ p_{T,B}\right\}\Big]\ \text{// MatrixForm;}$$

$$\text{% /. }\left\{z \to z\,\zeta,\ y \to y\,\zeta,\ \beta_2 \to \beta_2\,\zeta,\ \alpha_1 \to \alpha_1\,\zeta,\ p_{T,A} \to \left(p_{T,A,0} + p_{T,A,1}\,\zeta\right),\right.$$
$$\left. p_{t,B} \to \left(p_{t,B,0} + p_{t,B,1}\,\zeta\right),\ p_{T,B} \to \left(p_{T,B,0} + p_{T,B,1}\,\zeta\right)\right\}\ \text{// MatrixForm;}$$

$$\text{Det}\left[\% - \begin{pmatrix} \lambda_0 + \lambda_1\,\zeta & 0 & 0 \\ 0 & \lambda_0 + \lambda_1\,\zeta & 0 \\ 0 & 0 & \lambda_0 + \lambda_1\,\zeta \end{pmatrix}\right];$$

$$\text{Series}[\% == 0,\ \{\zeta,\ 0,\ 1\}];$$

$$\text{% /. }\left\{p_{T,A,0} \to 0,\ p_{t,B,0} \to 0,\ p_{T,B,0} \to 0,\ p_{T,A,1} \to \frac{\left(r + h\left(k - k\,r\right)\right) z}{h\,k\,\alpha_2},\right.$$
$$\left. p_{t,B,1} \to \frac{\left(-1+h\,k\right)\left(r\,z + h\,k\,\alpha_1\right)}{h\,k\left(-h\,k + \left(-1+h\,k\right)\beta_1\right)},\ p_{T,B,1} \to \frac{\left(-1+h\,k\right)(-1+r)\,z}{h\,k}\right\};$$

In[∘]:=

$$\text{Solve}\Big[\Big(-\left(1-h\,k\right)^2(1-r)\,r\,\alpha_2\left(-\left(\left(1-h\,k\right)(-1+\beta_1)\right) - \lambda_0\right) +$$
$$\left(\left(1-h\,k\right)(1-r) - \lambda_0\right)\left(\frac{1}{2}\left(-2\,(-1+\alpha_2) + 2\left(1-h\,k\right) r\,\alpha_2\right) - \lambda_0\right)\left(-\left(\left(1-h\,k\right)(-1+\beta_1)\right) - \lambda_0\right)\Big) == 0,\ \lambda_0\Big]$$

Out[∘]=

$$\Big\{\Big\{\lambda_0 \to \frac{1}{2}\Big(2 - h\,k - r + h\,k\,r - \alpha_2 + r\,\alpha_2 - h\,k\,r\,\alpha_2 -$$
$$\sqrt{\left(-4\left(1 - h\,k - r + h\,k\,r - \alpha_2 + h\,k\,\alpha_2 + r\,\alpha_2 - h\,k\,r\,\alpha_2\right) + \left(-2 + h\,k + r - h\,k\,r + \alpha_2 - r\,\alpha_2 + h\,k\,r\,\alpha_2\right)^2\right)}\Big)\Big\},$$
$$\Big\{\lambda_0 \to \frac{1}{2}\Big(2 - h\,k - r + h\,k\,r - \alpha_2 + r\,\alpha_2 - h\,k\,r\,\alpha_2 + \sqrt{\left(-4\left(1 - h\,k - r + h\,k\,r - \alpha_2 + h\,k\,\alpha_2 + r\,\alpha_2 - h\,k\,r\,\alpha_2\right) +\right.}$$
$$\left.\left(-2 + h\,k + r - h\,k\,r + \alpha_2 - r\,\alpha_2 + h\,k\,r\,\alpha_2\right)^2\right)\Big)\Big\},\ \left\{\lambda_0 \to \left(-1+h\,k\right)(-1+\beta_1)\right\}\Big\}$$

Calculations of the first order terms of the eigenvalues

A4 - Numerically evaluating accuracy of equilibria approximations

Evaluating the accuracy of the approximated equilibriumin comparison to numerical equilibrium solutions.

Table 1 : Evaluating the accuracy of the equilibrium approximation as the recombination rate increases from a lower to upper limit

r	~ $\hat{p}_B$ Analytical	$\hat{p}_B$ Numerical	Percentage Difference
0.001	0.00195231	0.00194682	0.28126
0.005	0.00198262	0.00197772	0.247414
0.009	0.00201294	0.00200976	0.158207
0.013	0.00204326	0.00204116	0.102371
0.017	0.00207357	0.00207032	0.156585
0.021	0.00210389	0.00209662	0.345592
0.025	0.0021342	0.00212003	0.664316
0.029	0.00216452	0.00214079	1.09637
0.033	0.00219484	0.00215921	1.62293
0.037	0.00222515	0.00217561	2.22627

Parameter values: h = 0.1, k = 0.0025, α_1 = 0.00001, α_2 = 0.001, β_1 = 0.005, β_2 = 0.00001, z = 10 ^ –8, y = 10 ^ –8

r	~ $\hat{p}_T$ Analytical	$\hat{p}_T$ Numerical	Percentage Difference
0.001	0.00008994	0.0000894929	0.49712
0.051	0.00024974	0.000251032	0.517348
0.101	0.00040954	0.00041812	2.09497
0.151	0.00056934	0.000583789	2.5379
0.201	0.00072914	0.000739986	1.48745
0.251	0.00088894	0.00088281	0.689556
0.301	0.00104874	0.00101147	3.55371
0.351	0.00120854	0.00112668	6.77322
0.401	0.00136834	0.00122974	10.1294
0.451	0.00152814	0.00132204	13.4868

Parameter values: h = 0.1, k = 0.0025, α_1 = 0.00001, α_2 = 0.001, β_1 = 0.005, β_2 = 0.00001, z = 10 ^ –8, y = 10 ^ –8

Note: For the parameter conditions assessed in the above tables, the corresponding eigenvalues were always between zero and one in absolute magnitude, suggesting local stability.

Case B: The transposon is deleterious and the epiallele is weakly deleterious

B1 - Fitness Assumptions

Genotype	Fitness
tA/tA	$w_1 = 1$
tA/tB	$w_2 = 1 - h_2\,k$
tB/tB	$w_3 = 1 - k$
TA/TA	$w_4 = 1 - \mathcal{L}$
TA/TB	$w_5 = 1 - \mathcal{L} - h_2\,k$
TB/TB	$w_6 = 1 - \mathcal{L} - k + \gamma$
tA/TA	$w_7 = 1 - h\mathcal{L}$
tB/TA	$w_8 = 1 - h\,\mathcal{L} - h_2\,k$
tA/TB	$w_9 = 1 - h\,\mathcal{L} - h_2\,k$
tB/TB	$w_{10} = 1 - h\,\mathcal{L} - k + \gamma$

B2 - Calculating Equilibria

$$\{0 == -p_{T,A} + \Big(z\,\Big(-2\,(1-r)\,w_8\,\big(\alpha_2\,p_{T,A} - (-1+\beta_2)\,p_{T,B}\big)\,\big(-\beta_1\,p_{t,B} - (-1+\alpha_1)\,(-1+p_{t,B}+p_{T,A}+p_{T,B})\big) +$$
$$2\,w_7\,\big((-1+\alpha_2)\,p_{T,A} - \beta_2\,p_{T,B}\big)\,\big(-\beta_1\,p_{t,B} - (-1+\alpha_1)\,(-1+p_{t,B}+p_{T,A}+p_{T,B})\big) +$$

$$2\, w_1 \left(\beta_1\, p_{t,B} + (-1+\alpha_1)\, (-1 + p_{t,B} + p_{T,A} + p_{T,B})\right)^2 -$$

$$2\, r\, w_9 \left((-1+\alpha_2)\, p_{T,A} - \beta_2\, p_{T,B}\right) \left(-\left((-1+\beta_1)\, p_{t,B}\right) - \alpha_1\, (-1 + p_{t,B} + p_{T,A} + p_{T,B})\right) +$$

$$w_2 \Big(-2\, (-1+\beta_1)\, p_{t,B} \left(1 + (-1+\beta_1)\, p_{t,B} - p_{T,A} - p_{T,B}\right) - 2\, \alpha_1 \left(1 + 2\, (-1+\beta_1)\, p_{t,B} - p_{T,A} - p_{T,B}\right)$$

$$(-1 + p_{t,B} + p_{T,A} + p_{T,B}) - 2\, \alpha_1^2\, (-1 + p_{t,B} + p_{T,A} + p_{T,B})^2\Big)\Big) +$$

$$(1-y) \Big(2\, w_4 \left((-1+\alpha_1)\, p_{T,A} - \beta_1\, p_{T,B}\right)^2 - 2\, r\, w_8 \left(\alpha_2\, p_{T,A} - (-1+\beta_2)\, p_{T,B}\right)$$

$$\left(-\beta_1\, p_{t,B} - (-1+\alpha_1)\, (-1 + p_{t,B} + p_{T,A} + p_{T,B})\right) +$$

$$2\, w_7 \left((-1+\alpha_2)\, p_{T,A} - \beta_2\, p_{T,B}\right) \left(-\beta_1\, p_{t,B} - (-1+\alpha_1)\, (-1 + p_{t,B} + p_{T,A} + p_{T,B})\right) +$$

$$2\, (-1+r)\, w_9 \left((-1+\alpha_2)\, p_{T,A} - \beta_2\, p_{T,B}\right) \left(-\left((-1+\beta_1)\, p_{t,B}\right) - \alpha_1\, (-1 + p_{t,B} + p_{T,A} + p_{T,B})\right) +$$

$$w_5 \left(-2\, \alpha_2^2\, p_{T,A}^2 - 2\, (-1+\beta_2)\, p_{T,B} \left(p_{T,A} + \beta_2\, p_{T,B}\right) + 2\, \alpha_2\, p_{T,A} \left(p_{T,A} + \left(-1 + 2\, \beta_2\right) p_{T,B}\right)\right)\Big)\Big) \Big/$$

$$\Big(2 \Big(w_4 \left((-1+\alpha_1)\, p_{T,A} - \beta_1\, p_{T,B}\right)^2 + w_6 \left(\alpha_2\, p_{T,A} - (-1+\beta_2)\, p_{T,B}\right)^2 - 2\, w_8 \left(\alpha_2\, p_{T,A} - (-1+\beta_2)\, p_{T,B}\right)$$

$$\left(-\beta_1\, p_{t,B} - (-1+\alpha_1)\, (-1 + p_{t,B} + p_{T,A} + p_{T,B})\right) + 2\, w_7 \left((-1+\alpha_2)\, p_{T,A} - \beta_2\, p_{T,B}\right)$$

$$\left(-\beta_1\, p_{t,B} - (-1+\alpha_1)\, (-1 + p_{t,B} + p_{T,A} + p_{T,B})\right) + w_1 \left(\beta_1\, p_{t,B} + (-1+\alpha_1)\, (-1 + p_{t,B} + p_{T,A} + p_{T,B})\right)^2 +$$

$$2\, w_{10} \left(\alpha_2\, p_{T,A} + (1-\beta_2)\, p_{T,B}\right) \left(-\left((-1+\beta_1)\, p_{t,B}\right) - \alpha_1\, (-1 + p_{t,B} + p_{T,A} + p_{T,B})\right) -$$

$$2\, w_9 \left((-1+\alpha_2)\, p_{T,A} - \beta_2\, p_{T,B}\right) \left(-\left((-1+\beta_1)\, p_{t,B}\right) - \alpha_1\, (-1 + p_{t,B} + p_{T,A} + p_{T,B})\right) + w_3$$

$$\left((-1+\beta_1)\, p_{t,B} + \alpha_1\, (-1 + p_{t,B} + p_{T,A} + p_{T,B})\right)^2 + w_2 \Big(-2\, (-1+\beta_1)\, p_{t,B} \left(1 + (-1+\beta_1)\, p_{t,B} - p_{T,A} - p_{T,B}\right) -$$

$$2\, \alpha_1 \left(1 + 2\, (-1+\beta_1)\, p_{t,B} - p_{T,A} - p_{T,B}\right) (-1 + p_{t,B} + p_{T,A} + p_{T,B}) - 2\, \alpha_1^2\, (-1 + p_{t,B} + p_{T,A} + p_{T,B})^2\Big) +$$

$$w_5 \left(-2\, \alpha_2^2\, p_{T,A}^2 - 2\, (-1+\beta_2)\, p_{T,B} \left(p_{T,A} + \beta_2\, p_{T,B}\right) + 2\, \alpha_2\, p_{T,A} \left(p_{T,A} + \left(-1 + 2\, \beta_2\right) p_{T,B}\right)\right)\Big)\Big),$$

$$0 == -p_{t,B} + \Big((1-z) \Big(-2\, r\, w_8 \left(\alpha_2\, p_{T,A} - (-1+\beta_2)\, p_{T,B}\right) \left(-\beta_1\, p_{t,B} - (-1+\alpha_1)\, (-1 + p_{t,B} + p_{T,A} + p_{T,B})\right) +$$

$$2\, w_{10} \left(\alpha_2\, p_{T,A} + (1-\beta_2)\, p_{T,B}\right) \left(-\left((-1+\beta_1)\, p_{t,B}\right) - \alpha_1\, (-1 + p_{t,B} + p_{T,A} + p_{T,B})\right) +$$

$$2\, (-1+r)\, w_9 \left((-1+\alpha_2)\, p_{T,A} - \beta_2\, p_{T,B}\right) \left(-\left((-1+\beta_1)\, p_{t,B}\right) - \alpha_1\, (-1 + p_{t,B} + p_{T,A} + p_{T,B})\right) +$$

$$2\, w_3 \left((-1+\beta_1)\, p_{t,B} + \alpha_1\, (-1 + p_{t,B} + p_{T,A} + p_{T,B})\right)^2 +$$

$$w_2 \Big(-2\, (-1+\beta_1)\, p_{t,B} \left(1 + (-1+\beta_1)\, p_{t,B} - p_{T,A} - p_{T,B}\right) - 2\, \alpha_1 \left(1 + 2\, (-1+\beta_1)\, p_{t,B} - p_{T,A} - p_{T,B}\right)$$

$$(-1 + p_{t,B} + p_{T,A} + p_{T,B}) - 2\, \alpha_1^2\, (-1 + p_{t,B} + p_{T,A} + p_{T,B})^2\Big)\Big) + y \Big(2\, w_6 \left(\alpha_2\, p_{T,A} - (-1+\beta_2)\, p_{T,B}\right)^2 -$$

$$2\, (1-r)\, w_8 \left(\alpha_2\, p_{T,A} - (-1+\beta_2)\, p_{T,B}\right) \left(-\beta_1\, p_{t,B} - (-1+\alpha_1)\, (-1 + p_{t,B} + p_{T,A} + p_{T,B})\right) +$$

$$2\, w_{10} \left(\alpha_2\, p_{T,A} + (1-\beta_2)\, p_{T,B}\right) \left(-\left((-1+\beta_1)\, p_{t,B}\right) - \alpha_1\, (-1 + p_{t,B} + p_{T,A} + p_{T,B})\right) -$$

$$2\, r\, w_9 \left((-1+\alpha_2)\, p_{T,A} - \beta_2\, p_{T,B}\right) \left(-\left((-1+\beta_1)\, p_{t,B}\right) - \alpha_1\, (-1 + p_{t,B} + p_{T,A} + p_{T,B})\right) +$$

$$w_5 \left(-2\, \alpha_2^2\, p_{T,A}^2 - 2\, (-1+\beta_2)\, p_{T,B} \left(p_{T,A} + \beta_2\, p_{T,B}\right) + 2\, \alpha_2\, p_{T,A} \left(p_{T,A} + \left(-1 + 2\, \beta_2\right) p_{T,B}\right)\right)\Big)\Big) \Big/$$

$$\Big(2 \Big(w_4 \left((-1+\alpha_1)\, p_{T,A} - \beta_1\, p_{T,B}\right)^2 + w_6 \left(\alpha_2\, p_{T,A} - (-1+\beta_2)\, p_{T,B}\right)^2 - 2\, w_8 \left(\alpha_2\, p_{T,A} - (-1+\beta_2)\, p_{T,B}\right)$$

$$\left(-\beta_1\, p_{t,B} - (-1+\alpha_1)\, (-1 + p_{t,B} + p_{T,A} + p_{T,B})\right) + 2\, w_7 \left((-1+\alpha_2)\, p_{T,A} - \beta_2\, p_{T,B}\right)$$

$$\left(-\beta_1\, p_{t,B} - (-1+\alpha_1)\, (-1 + p_{t,B} + p_{T,A} + p_{T,B})\right) + w_1 \left(\beta_1\, p_{t,B} + (-1+\alpha_1)\, (-1 + p_{t,B} + p_{T,A} + p_{T,B})\right)^2 +$$

$$2\, w_{10} \left(\alpha_2\, p_{T,A} + (1-\beta_2)\, p_{T,B}\right) \left(-\left((-1+\beta_1)\, p_{t,B}\right) - \alpha_1\, (-1 + p_{t,B} + p_{T,A} + p_{T,B})\right) -$$

$$2\, w_9 \left((-1+\alpha_2)\, p_{T,A} - \beta_2\, p_{T,B}\right) \left(-\left((-1+\beta_1)\, p_{t,B}\right) - \alpha_1\, (-1 + p_{t,B} + p_{T,A} + p_{T,B})\right) + w_3$$

$$\left((-1+\beta_1)\, p_{t,B} + \alpha_1\, (-1 + p_{t,B} + p_{T,A} + p_{T,B})\right)^2 + w_2 \Big(-2\, (-1+\beta_1)\, p_{t,B} \left(1 + (-1+\beta_1)\, p_{t,B} - p_{T,A} - p_{T,B}\right) -$$

$$2\, \alpha_1 \left(1 + 2\, (-1+\beta_1)\, p_{t,B} - p_{T,A} - p_{T,B}\right) (-1 + p_{t,B} + p_{T,A} + p_{T,B}) - 2\, \alpha_1^2\, (-1 + p_{t,B} + p_{T,A} + p_{T,B})^2\Big) +$$

$$w_5 \left(-2\, \alpha_2^2\, p_{T,A}^2 - 2\, (-1+\beta_2)\, p_{T,B} \left(p_{T,A} + \beta_2\, p_{T,B}\right) + 2\, \alpha_2\, p_{T,A} \left(p_{T,A} + \left(-1 + 2\, \beta_2\right) p_{T,B}\right)\right)\Big)\Big),$$

$$0 == -p_{T,B} + \Big(z \Big(-2\, r\, w_8 \left(\alpha_2\, p_{T,A} - (-1+\beta_2)\, p_{T,B}\right) \left(-\beta_1\, p_{t,B} - (-1+\alpha_1)\, (-1 + p_{t,B} + p_{T,A} + p_{T,B})\right) +$$

$$2\, w_{10} \left(\alpha_2\, p_{T,A} + (1-\beta_2)\, p_{T,B}\right) \left(-\left((-1+\beta_1)\, p_{t,B}\right) - \alpha_1 \left(-1 + p_{t,B} + p_{T,A} + p_{T,B}\right)\right) +$$

$$2\, (-1+r)\, w_9 \left((-1+\alpha_2)\, p_{T,A} - \beta_2\, p_{T,B}\right) \left(-\left((-1+\beta_1)\, p_{t,B}\right) - \alpha_1 \left(-1 + p_{t,B} + p_{T,A} + p_{T,B}\right)\right) +$$

$$2\, w_3 \left((-1+\beta_1)\, p_{t,B} + \alpha_1 \left(-1 + p_{t,B} + p_{T,A} + p_{T,B}\right)\right)^2 +$$

$$w_2 \left(-2\, (-1+\beta_1)\, p_{t,B} \left(1 + (-1+\beta_1)\, p_{t,B} - p_{T,A} - p_{T,B}\right) - 2\, \alpha_1 \left(1 + 2\, (-1+\beta_1)\, p_{t,B} - p_{T,A} - p_{T,B}\right)\right.$$

$$\left.\left(-1 + p_{t,B} + p_{T,A} + p_{T,B}\right) - 2\, \alpha_1^2 \left(-1 + p_{t,B} + p_{T,A} + p_{T,B}\right)^2\right)\Big) +$$

$$(1-y) \Big(2\, w_6 \left(\alpha_2\, p_{T,A} - (-1+\beta_2)\, p_{T,B}\right)^2 - 2\, (1-r)\, w_8 \left(\alpha_2\, p_{T,A} - (-1+\beta_2)\, p_{T,B}\right)$$

$$\left(-\beta_1\, p_{t,B} - (-1+\alpha_1) \left(-1 + p_{t,B} + p_{T,A} + p_{T,B}\right)\right) +$$

$$2\, w_{10} \left(\alpha_2\, p_{T,A} + (1-\beta_2)\, p_{T,B}\right) \left(-\left((-1+\beta_1)\, p_{t,B}\right) - \alpha_1 \left(-1 + p_{t,B} + p_{T,A} + p_{T,B}\right)\right) -$$

$$2\, r\, w_9 \left((-1+\alpha_2)\, p_{T,A} - \beta_2\, p_{T,B}\right) \left(-\left((-1+\beta_1)\, p_{t,B}\right) - \alpha_1 \left(-1 + p_{t,B} + p_{T,A} + p_{T,B}\right)\right) +$$

$$w_5 \left(-2\, \alpha_2^2\, p_{T,A}^2 - 2\, (-1+\beta_2)\, p_{T,B} \left(p_{T,A} + \beta_2\, p_{T,B}\right) + 2\, \alpha_2\, p_{T,A} \left(p_{T,A} + \left(-1 + 2\, \beta_2\right) p_{T,B}\right)\right)\Big)\Big) \Big/$$

$$\Big(2 \Big(w_4 \left((-1+\alpha_1)\, p_{T,A} - \beta_1\, p_{T,B}\right)^2 + w_6 \left(\alpha_2\, p_{T,A} - (-1+\beta_2)\, p_{T,B}\right)^2 - 2\, w_8 \left(\alpha_2\, p_{T,A} - (-1+\beta_2)\, p_{T,B}\right)$$

$$\left(-\beta_1\, p_{t,B} - (-1+\alpha_1) \left(-1 + p_{t,B} + p_{T,A} + p_{T,B}\right)\right) + 2\, w_7 \left((-1+\alpha_2)\, p_{T,A} - \beta_2\, p_{T,B}\right)$$

$$\left(-\beta_1\, p_{t,B} - (-1+\alpha_1) \left(-1 + p_{t,B} + p_{T,A} + p_{T,B}\right)\right) + w_1 \left(\beta_1\, p_{t,B} + (-1+\alpha_1) \left(-1 + p_{t,B} + p_{T,A} + p_{T,B}\right)\right)^2 +$$

$$2\, w_{10} \left(\alpha_2\, p_{T,A} + (1-\beta_2)\, p_{T,B}\right) \left(-\left((-1+\beta_1)\, p_{t,B}\right) - \alpha_1 \left(-1 + p_{t,B} + p_{T,A} + p_{T,B}\right)\right) -$$

$$2\, w_9 \left((-1+\alpha_2)\, p_{T,A} - \beta_2\, p_{T,B}\right) \left(-\left((-1+\beta_1)\, p_{t,B}\right) - \alpha_1 \left(-1 + p_{t,B} + p_{T,A} + p_{T,B}\right)\right) + w_3$$

$$\left((-1+\beta_1)\, p_{t,B} + \alpha_1 \left(-1 + p_{t,B} + p_{T,A} + p_{T,B}\right)\right)^2 + w_2 \left(-2\, (-1+\beta_1)\, p_{t,B} \left(1 + (-1+\beta_1)\, p_{t,B} - p_{T,A} - p_{T,B}\right) -\right.$$

$$\left.2\, \alpha_1 \left(1 + 2\, (-1+\beta_1)\, p_{t,B} - p_{T,A} - p_{T,B}\right) \left(-1 + p_{t,B} + p_{T,A} + p_{T,B}\right) - 2\, \alpha_1^2 \left(-1 + p_{t,B} + p_{T,A} + p_{T,B}\right)^2\right) +$$

$$w_5 \left(-2\, \alpha_2^2\, p_{T,A}^2 - 2\, (-1+\beta_2)\, p_{T,B} \left(p_{T,A} + \beta_2\, p_{T,B}\right) + 2\, \alpha_2\, p_{T,A} \left(p_{T,A} + \left(-1 + 2\, \beta_2\right) p_{T,B}\right)\right)\Big)\Big)\Big\} \;/.$$

$$\{w_1 \to 1,\ w_2 \to 1 - h2\, k,\ w_3 \to (1-k),\ w_4 \to (1-\mathcal{L}),$$
$$w_5 \to (1 - \mathcal{L} - h2\, k),$$
$$w_6 \to (1 - \mathcal{L} - k + \gamma),\ w_7 \to (1 - h\, \mathcal{L}),\ w_8 \to (1 - h\, \mathcal{L} - h2\, k),\ w_9 \to (1 - h\, \mathcal{L} - h2\, k),\ w_{10} \to (1 - h\, \mathcal{L} - k + \gamma)\};$$

$$\%\ /.\ \{z \to z\, \zeta,\ y \to y\, \zeta,\ \beta_2 \to \beta_2\, \zeta,\ \alpha_1 \to \alpha_1\, \zeta,\ k \to k\, \zeta,\ \gamma \to \gamma\, \zeta,$$
$$p_{T,A} \to \left(p_{T,A,0} + p_{T,A,1}\, \zeta\right),\ p_{t,B} \to \left(p_{t,B,0} + p_{t,B,1}\, \zeta\right),\ p_{T,B} \to \left(p_{T,B,0} + p_{T,B,1}\, \zeta\right)\};$$

```
Series[%, {ζ, 0, 1}];
```

Substituting $p_{T,A,0} \to 0$, $p_{t,B,0} \to 0$, $p_{T,B,0} \to 0$, into the first order terms and solving...

In[∘]:= $\text{Solve}[\% == 0, \{p_{T,A,1},\ p_{t,B,1},\ p_{T,B,1}\}];$

In[◦]:= `Simplify[%]`

Out[◦]=

$$\left\{\left\{p_{T,A,1} \to \frac{z\left(r + h\mathcal{L} - h\,r\,\mathcal{L}\right)}{h\mathcal{L}\left(r + h\mathcal{L} - h\,r\,\mathcal{L} + (-1 + r)\left(-1 + h\mathcal{L}\right)\alpha_2\right)},\right.\right.$$

$$\left.\left.p_{t,B,1} \to \frac{\alpha_1 + \frac{r\,z\left(1-h\mathcal{L}\right)\alpha_2}{h\mathcal{L}\left(r+h\mathcal{L}-h\,r\,\mathcal{L}+(-1+r)\left(-1+h\mathcal{L}\right)\alpha_2\right)}}{\beta_1},\ p_{T,B,1} \to \frac{(-1 + r)\,z\left(-1 + h\mathcal{L}\right)\alpha_2}{h\mathcal{L}\left(r + h\mathcal{L} - h\,r\,\mathcal{L} + (-1 + r)\left(-1 + h\mathcal{L}\right)\alpha_2\right)}\right\}\right\}$$

After simplifying and writing in terms of (epi)allele frequencies leads to eq. 2 from File S3.

B3 - Calculating Eigenvalues

In[◦]:= `ResourceFunction["JacobianMatrix"][`

$$\begin{aligned}
&\{(z(-2(-1+\beta_1)p_{t,B}(1+(-1+\beta_1)p_{t,B}-p_{T,A}-p_{T,B})-2\alpha_1(1+2(-1+\beta_1)p_{t,B}-p_{T,A}-p_{T,B})\\
&\quad(-1+p_{t,B}+p_{T,A}+p_{T,B})-2\alpha_1^2(-1+p_{t,B}+p_{T,A}+p_{T,B})^2-2(1-r)(1-h\mathcal{L})(\alpha_2 p_{T,A}-(-1+\beta_2)p_{T,B})\\
&\quad(-\beta_1 p_{t,B}-(-1+\alpha_1)(-1+p_{t,B}+p_{T,A}+p_{T,B}))+2(1-h\mathcal{L})((-1+\alpha_2)p_{T,A}-\beta_2 p_{T,B})\\
&\quad(-\beta_1 p_{t,B}-(-1+\alpha_1)(-1+p_{t,B}+p_{T,A}+p_{T,B}))+2(\beta_1 p_{t,B}+(-1+\alpha_1)(-1+p_{t,B}+p_{T,A}+p_{T,B}))^2-\\
&\quad 2r(1-h\mathcal{L})((-1+\alpha_2)p_{T,A}-\beta_2 p_{T,B})(-((-1+\beta_1)p_{t,B})-\alpha_1(-1+p_{t,B}+p_{T,A}+p_{T,B})))+\\
&\quad(1-y)(2(1-\mathcal{L})((-1+\alpha_1)p_{T,A}-\beta_1 p_{T,B})^2-2r(1-h\mathcal{L})(\alpha_2 p_{T,A}-(-1+\beta_2)p_{T,B})\\
&\quad(-\beta_1 p_{t,B}-(-1+\alpha_1)(-1+p_{t,B}+p_{T,A}+p_{T,B}))+\\
&\quad 2(1-h\mathcal{L})((-1+\alpha_2)p_{T,A}-\beta_2 p_{T,B})(-\beta_1 p_{t,B}-(-1+\alpha_1)(-1+p_{t,B}+p_{T,A}+p_{T,B}))+\\
&\quad 2(-1+r)(1-h\mathcal{L})((-1+\alpha_2)p_{T,A}-\beta_2 p_{T,B})(-((-1+\beta_1)p_{t,B})-\alpha_1(-1+p_{t,B}+p_{T,A}+p_{T,B}))+\\
&\quad(1-\mathcal{L})(-2\alpha_2^2 p_{T,A}^2-2(-1+\beta_2)p_{T,B}(p_{T,A}+\beta_2 p_{T,B})+2\alpha_2 p_{T,A}(p_{T,A}+(-1+2\beta_2)p_{T,B}))))/\\
&\quad(2(-2(-1+\beta_1)p_{t,B}(1+(-1+\beta_1)p_{t,B}-p_{T,A}-p_{T,B})-2\alpha_1(1+2(-1+\beta_1)p_{t,B}-p_{T,A}-p_{T,B})\\
&\quad(-1+p_{t,B}+p_{T,A}+p_{T,B})-2\alpha_1^2(-1+p_{t,B}+p_{T,A}+p_{T,B})^2+\\
&\quad(1-\mathcal{L})((-1+\alpha_1)p_{T,A}-\beta_1 p_{T,B})^2+(1-k-\mathcal{L}+\gamma)(\alpha_2 p_{T,A}-(-1+\beta_2)p_{T,B})^2-\\
&\quad 2(1-h\mathcal{L})(\alpha_2 p_{T,A}-(-1+\beta_2)p_{T,B})(-\beta_1 p_{t,B}-(-1+\alpha_1)(-1+p_{t,B}+p_{T,A}+p_{T,B}))+\\
&\quad 2(1-h\mathcal{L})((-1+\alpha_2)p_{T,A}-\beta_2 p_{T,B})(-\beta_1 p_{t,B}-(-1+\alpha_1)(-1+p_{t,B}+p_{T,A}+p_{T,B}))+\\
&\quad(\beta_1 p_{t,B}+(-1+\alpha_1)(-1+p_{t,B}+p_{T,A}+p_{T,B}))^2+\\
&\quad 2(1-k-h\mathcal{L}+\gamma)(\alpha_2 p_{T,A}+(1-\beta_2)p_{T,B})(-((-1+\beta_1)p_{t,B})-\alpha_1(-1+p_{t,B}+p_{T,A}+p_{T,B}))-\\
&\quad 2(1-h\mathcal{L})((-1+\alpha_2)p_{T,A}-\beta_2 p_{T,B})(-((-1+\beta_1)p_{t,B})-\alpha_1(-1+p_{t,B}+p_{T,A}+p_{T,B}))+\\
&\quad(1-k)((-1+\beta_1)p_{t,B}+\alpha_1(-1+p_{t,B}+p_{T,A}+p_{T,B}))^2+\\
&\quad(1-\mathcal{L})(-2\alpha_2^2 p_{T,A}^2-2(-1+\beta_2)p_{T,B}(p_{T,A}+\beta_2 p_{T,B})+2\alpha_2 p_{T,A}(p_{T,A}+(-1+2\beta_2)p_{T,B})))),\\
&((1-z)(-2(-1+\beta_1)p_{t,B}(1+(-1+\beta_1)p_{t,B}-p_{T,A}-p_{T,B})-2\alpha_1(1+2(-1+\beta_1)p_{t,B}-p_{T,A}-p_{T,B})\\
&\quad(-1+p_{t,B}+p_{T,A}+p_{T,B})-2\alpha_1^2(-1+p_{t,B}+p_{T,A}+p_{T,B})^2-\\
&\quad 2r(1-h\mathcal{L})(\alpha_2 p_{T,A}-(-1+\beta_2)p_{T,B})(-\beta_1 p_{t,B}-(-1+\alpha_1)(-1+p_{t,B}+p_{T,A}+p_{T,B}))+\\
&\quad 2(1-k-h\mathcal{L}+\gamma)(\alpha_2 p_{T,A}+(1-\beta_2)p_{T,B})(-((-1+\beta_1)p_{t,B})-\alpha_1(-1+p_{t,B}+p_{T,A}+p_{T,B}))+\\
&\quad 2(-1+r)(1-h\mathcal{L})((-1+\alpha_2)p_{T,A}-\beta_2 p_{T,B})(-((-1+\beta_1)p_{t,B})-\alpha_1(-1+p_{t,B}+p_{T,A}+p_{T,B}))+
\end{aligned}$$

$$
\begin{aligned}
&2\,(1-k)\,((-1+\beta_1)\,p_{t,B}+\alpha_1\,(-1+p_{t,B}+p_{T,A}+p_{T,B}))^2)+y\,(2\,(1-k-\mathcal{L}+\gamma)\,(\alpha_2\,p_{T,A}-(-1+\beta_2)\,p_{T,B})^2-\\
&2\,(1-r)\,(1-h\,\mathcal{L})\,(\alpha_2\,p_{T,A}-(-1+\beta_2)\,p_{T,B})\,(-\beta_1\,p_{t,B}-(-1+\alpha_1)\,(-1+p_{t,B}+p_{T,A}+p_{T,B}))+\\
&2\,(1-k-h\,\mathcal{L}+\gamma)\,(\alpha_2\,p_{T,A}+(1-\beta_2)\,p_{T,B})\,(-((-1+\beta_1)\,p_{t,B})-\alpha_1\,(-1+p_{t,B}+p_{T,A}+p_{T,B}))-\\
&2\,r\,(1-h\,\mathcal{L})\,((-1+\alpha_2)\,p_{T,A}-\beta_2\,p_{T,B})\,(-((-1+\beta_1)\,p_{t,B})-\alpha_1\,(-1+p_{t,B}+p_{T,A}+p_{T,B}))+\\
&(1-\mathcal{L})\,(-2\,\alpha_2^2\,p_{T,A}^2-2\,(-1+\beta_2)\,p_{T,B}\,(p_{T,A}+\beta_2\,p_{T,B})+2\,\alpha_2\,p_{T,A}\,(p_{T,A}+(-1+2\,\beta_2)\,p_{T,B}))))\,/\\
&(2\,(-2\,(-1+\beta_1)\,p_{t,B}\,(1+(-1+\beta_1)\,p_{t,B}-p_{T,A}-p_{T,B})-2\,\alpha_1\,(1+2\,(-1+\beta_1)\,p_{t,B}-p_{T,A}-p_{T,B})\\
&(-1+p_{t,B}+p_{T,A}+p_{T,B})-2\,\alpha_1^2\,(-1+p_{t,B}+p_{T,A}+p_{T,B})^2+\\
&(1-\mathcal{L})\,((-1+\alpha_1)\,p_{T,A}-\beta_1\,p_{T,B})^2+(1-k-\mathcal{L}+\gamma)\,(\alpha_2\,p_{T,A}-(-1+\beta_2)\,p_{T,B})^2-\\
&2\,(1-h\,\mathcal{L})\,(\alpha_2\,p_{T,A}-(-1+\beta_2)\,p_{T,B})\,(-\beta_1\,p_{t,B}-(-1+\alpha_1)\,(-1+p_{t,B}+p_{T,A}+p_{T,B}))+\\
&2\,(1-h\,\mathcal{L})\,((-1+\alpha_2)\,p_{T,A}-\beta_2\,p_{T,B})\,(-\beta_1\,p_{t,B}-(-1+\alpha_1)\,(-1+p_{t,B}+p_{T,A}+p_{T,B}))+\\
&(\beta_1\,p_{t,B}+(-1+\alpha_1)\,(-1+p_{t,B}+p_{T,A}+p_{T,B}))^2+\\
&2\,(1-k-h\,\mathcal{L}+\gamma)\,(\alpha_2\,p_{T,A}+(1-\beta_2)\,p_{T,B})\,(-((-1+\beta_1)\,p_{t,B})-\alpha_1\,(-1+p_{t,B}+p_{T,A}+p_{T,B}))-\\
&2\,(1-h\,\mathcal{L})\,((-1+\alpha_2)\,p_{T,A}-\beta_2\,p_{T,B})\,(-((-1+\beta_1)\,p_{t,B})-\alpha_1\,(-1+p_{t,B}+p_{T,A}+p_{T,B}))+\\
&(1-k)\,((-1+\beta_1)\,p_{t,B}+\alpha_1\,(-1+p_{t,B}+p_{T,A}+p_{T,B}))^2+\\
&(1-\mathcal{L})\,(-2\,\alpha_2^2\,p_{T,A}^2-2\,(-1+\beta_2)\,p_{T,B}\,(p_{T,A}+\beta_2\,p_{T,B})+2\,\alpha_2\,p_{T,A}\,(p_{T,A}+(-1+2\,\beta_2)\,p_{T,B})))),\\
&(z\,(-2\,(-1+\beta_1)\,p_{t,B}\,(1+(-1+\beta_1)\,p_{t,B}-p_{T,A}-p_{T,B})-2\,\alpha_1\,(1+2\,(-1+\beta_1)\,p_{t,B}-p_{T,A}-p_{T,B})\\
&(-1+p_{t,B}+p_{T,A}+p_{T,B})-2\,\alpha_1^2\,(-1+p_{t,B}+p_{T,A}+p_{T,B})^2-\\
&2\,r\,(1-h\,\mathcal{L})\,(\alpha_2\,p_{T,A}-(-1+\beta_2)\,p_{T,B})\,(-\beta_1\,p_{t,B}-(-1+\alpha_1)\,(-1+p_{t,B}+p_{T,A}+p_{T,B}))+\\
&2\,(1-k-h\,\mathcal{L}+\gamma)\,(\alpha_2\,p_{T,A}+(1-\beta_2)\,p_{T,B})\,(-((-1+\beta_1)\,p_{t,B})-\alpha_1\,(-1+p_{t,B}+p_{T,A}+p_{T,B}))+\\
&2\,(-1+r)\,(1-h\,\mathcal{L})\,((-1+\alpha_2)\,p_{T,A}-\beta_2\,p_{T,B})\,(-((-1+\beta_1)\,p_{t,B})-\alpha_1\,(-1+p_{t,B}+p_{T,A}+p_{T,B}))+\\
&2\,(1-k)\,((-1+\beta_1)\,p_{t,B}+\alpha_1\,(-1+p_{t,B}+p_{T,A}+p_{T,B}))^2)+\\
&(1-y)\,(2\,(1-k-\mathcal{L}+\gamma)\,(\alpha_2\,p_{T,A}-(-1+\beta_2)\,p_{T,B})^2-2\,(1-r)\,(1-h\,\mathcal{L})\,(\alpha_2\,p_{T,A}-(-1+\beta_2)\,p_{T,B})\\
&(-\beta_1\,p_{t,B}-(-1+\alpha_1)\,(-1+p_{t,B}+p_{T,A}+p_{T,B}))+\\
&2\,(1-k-h\,\mathcal{L}+\gamma)\,(\alpha_2\,p_{T,A}+(1-\beta_2)\,p_{T,B})\,(-((-1+\beta_1)\,p_{t,B})-\alpha_1\,(-1+p_{t,B}+p_{T,A}+p_{T,B}))-\\
&2\,r\,(1-h\,\mathcal{L})\,((-1+\alpha_2)\,p_{T,A}-\beta_2\,p_{T,B})\,(-((-1+\beta_1)\,p_{t,B})-\alpha_1\,(-1+p_{t,B}+p_{T,A}+p_{T,B}))+\\
&(1-\mathcal{L})\,(-2\,\alpha_2^2\,p_{T,A}^2-2\,(-1+\beta_2)\,p_{T,B}\,(p_{T,A}+\beta_2\,p_{T,B})+2\,\alpha_2\,p_{T,A}\,(p_{T,A}+(-1+2\,\beta_2)\,p_{T,B}))))\,/\\
&(2\,(-2\,(-1+\beta_1)\,p_{t,B}\,(1+(-1+\beta_1)\,p_{t,B}-p_{T,A}-p_{T,B})-2\,\alpha_1\,(1+2\,(-1+\beta_1)\,p_{t,B}-p_{T,A}-p_{T,B})\\
&(-1+p_{t,B}+p_{T,A}+p_{T,B})-2\,\alpha_1^2\,(-1+p_{t,B}+p_{T,A}+p_{T,B})^2+\\
&(1-\mathcal{L})\,((-1+\alpha_1)\,p_{T,A}-\beta_1\,p_{T,B})^2+(1-k-\mathcal{L}+\gamma)\,(\alpha_2\,p_{T,A}-(-1+\beta_2)\,p_{T,B})^2-\\
&2\,(1-h\,\mathcal{L})\,(\alpha_2\,p_{T,A}-(-1+\beta_2)\,p_{T,B})\,(-\beta_1\,p_{t,B}-(-1+\alpha_1)\,(-1+p_{t,B}+p_{T,A}+p_{T,B}))+\\
&2\,(1-h\,\mathcal{L})\,((-1+\alpha_2)\,p_{T,A}-\beta_2\,p_{T,B})\,(-\beta_1\,p_{t,B}-(-1+\alpha_1)\,(-1+p_{t,B}+p_{T,A}+p_{T,B}))+\\
&(\beta_1\,p_{t,B}+(-1+\alpha_1)\,(-1+p_{t,B}+p_{T,A}+p_{T,B}))^2+\\
&2\,(1-k-h\,\mathcal{L}+\gamma)\,(\alpha_2\,p_{T,A}+(1-\beta_2)\,p_{T,B})\,(-((-1+\beta_1)\,p_{t,B})-\alpha_1\,(-1+p_{t,B}+p_{T,A}+p_{T,B}))-\\
&2\,(1-h\,\mathcal{L})\,((-1+\alpha_2)\,p_{T,A}-\beta_2\,p_{T,B})\,(-((-1+\beta_1)\,p_{t,B})-\alpha_1\,(-1+p_{t,B}+p_{T,A}+p_{T,B}))+\\
&(1-k)\,((-1+\beta_1)\,p_{t,B}+\alpha_1\,(-1+p_{t,B}+p_{T,A}+p_{T,B}))^2+(1-\mathcal{L})\,(-2\,\alpha_2^2\,p_{T,A}^2-2\,(-1+\beta_2)\,p_{T,B}\\
&(p_{T,A}+\beta_2\,p_{T,B})+2\,\alpha_2\,p_{T,A}\,(p_{T,A}+(-1+2\,\beta_2)\,p_{T,B}))))\},\ \{p_{T,A},\ p_{t,B},\ p_{T,B}\}]\ //\ \mathtt{MatrixForm};
\end{aligned}
$$

```
% /. {z → z ζ, y → y ζ, β2 → β2 ζ, α1 → α1 ζ, k → k ζ, ɣ → ɣ ζ,
    pT,A → (pT,A,0 + pT,A,1 ζ), pt,B → (pt,B,0 + pt,B,1 ζ), pT,B → (pT,B,0 + pT,B,1 ζ)} // MatrixForm;
```

In[∘]:=

$$\text{Det}\left[\% - \begin{pmatrix} \lambda_0 + \lambda_1\ \zeta & 0 & 0 \\ 0 & \lambda_0 + \lambda_1\ \zeta & 0 \\ 0 & 0 & \lambda_0 + \lambda_1\ \zeta \end{pmatrix}\right];$$

In[∘]:=

```
Series[% == 0, {ζ, 0, 1}];
```

In[∘]:=

$$\% \ /. \ \left\{p_{T,A,0} \to 0,\ p_{t,B,0} \to 0,\ p_{T,B,0} \to 0,\ p_{T,A,1} \to \frac{z\left(r + h\,\mathcal{L} - h\,r\,\mathcal{L}\right)}{h\,\mathcal{L}\left(r + h\,\mathcal{L} - h\,r\,\mathcal{L} + (-1+r)\left(-1 + h\,\mathcal{L}\right)\alpha_2\right)},\right.$$

$$\left. p_{t,B,1} \to \frac{\alpha_1 + \frac{r\,z\left(1 - h\,\mathcal{L}\right)\alpha_2}{h\,\mathcal{L}\left(r + h\,\mathcal{L} - h\,r\,\mathcal{L} + (-1+r)\left(-1 + h\,\mathcal{L}\right)\alpha_2\right)}}{\beta_1},\ p_{T,B,1} \to \frac{(-1+r)\,z\left(-1 + h\,\mathcal{L}\right)\alpha_2}{h\,\mathcal{L}\left(r + h\,\mathcal{L} - h\,r\,\mathcal{L} + (-1+r)\left(-1 + h\,\mathcal{L}\right)\alpha_2\right)}\right\};$$

In[∘]:=

$$\text{Solve}\left[\left(-\left((1-r)\,r\left(1 - h\,\mathcal{L}\right)^2 \alpha_2\,(1 - \beta_1 - \lambda_0)\right) + \left((1-r)\left(1 - h\,\mathcal{L}\right) - \lambda_0\right)\left(\frac{1}{2}\left(-2\left(1 - h\,\mathcal{L}\right)(-1 + \alpha_2) + 2\,r\left(1 - h\,\mathcal{L}\right)\alpha_2\right) - \lambda_0\right)(1 - \beta_1 - \lambda_0)\right) == 0,\ \lambda_0\right]$$

Out[∘]=

$$\left\{\left\{\lambda_0 \to 1 - h\,\mathcal{L}\right\},\ \left\{\lambda_0 \to -\left((-1+r)\left(-1 + h\,\mathcal{L}\right)(-1 + \alpha_2)\right)\right\},\ \{\lambda_0 \to 1 - \beta_1\}\right\}$$

Calculations of the first order terms of the eigenvalues

B4 - Numerically evaluating accuracy of equilibria approximations

Evaluating the accuracy of the approximated equilibrium in comparison to numerical equilibrium solutions.

Table 1 : Evaluating the accuracy of the equilibrium approximation as the dominance coefficient increases from a lower to upper limit

h	~ $\hat{p}_B$ Analytical	$\hat{p}_B$ Numerical	Percentage Difference
0.01	0.0050504	0.0050245	0.512668
0.11	0.00500454	0.00497949	0.500423
0.21	0.00500235	0.00497733	0.5002
0.31	0.00500158	0.00497656	0.500122
0.41	0.00500118	0.00497617	0.500082
0.51	0.00500094	0.00497593	0.500057
0.61	0.00500078	0.00497577	0.500041
0.71	0.00500066	0.00497566	0.50003
0.81	0.00500058	0.00497557	0.500021
0.91	0.00500051	0.0049755	0.500014

Parameter values: r = 0.1, k = 0.00001, $\mathcal{L}$ = 0.01, α_1 = 0.00001, α_2 = 0.001, β_1 = 0.002, β_2 = 0.00001, z = 10 ^ -8, y = 10 ^ -8

h	$\sim \hat{p}_T$ Analytical	$\hat{p}_T$ Numerical	Percentage Difference
0.01	0.0001	0.0000992177	0.782281
0.11	9.09091×10^{-6}	9.09038×10^{-6}	0.00577987
0.21	4.7619×10^{-6}	4.76184×10^{-6}	0.00137006
0.31	3.22581×10^{-6}	3.22579×10^{-6}	0.000529558
0.41	2.43902×10^{-6}	2.43902×10^{-6}	0.000246003
0.51	1.96078×10^{-6}	1.96078×10^{-6}	0.000122292
0.61	1.63934×10^{-6}	1.63934×10^{-6}	0.0000598117
0.71	1.40845×10^{-6}	1.40845×10^{-6}	0.000025187
0.81	1.23457×10^{-6}	1.23457×10^{-6}	4.77183×10^{-6}
0.91	1.0989×10^{-6}	1.0989×10^{-6}	7.77897×10^{-6}

Parameter values: r = 0.1, k = 0.00001, $\mathcal{L}$ = 0.01, α_1 = 0.00001, α_2 = 0.001, β_1 = 0.002, β_2 = 0.00001, z = 10 ^ –8, y = 10 ^ –8

Table 2 : Evaluating the accuracy of the equilibrium approximation as the recombination rate increases from a lower to upper limit

r	$\sim \hat{p}_B$ Analytical	$\hat{p}_B$ Numerical	Percentage Difference
0.001	0.005005	0.00497995	0.500475
0.051	0.005005	0.00497995	0.500471
0.101	0.005005	0.00497995	0.500471
0.151	0.005005	0.00497995	0.500471
0.201	0.005005	0.00497995	0.500471
0.251	0.005005	0.00497995	0.500471
0.301	0.005005	0.00497995	0.500471
0.351	0.005005	0.00497995	0.500471
0.401	0.005005	0.00497995	0.500471
0.451	0.005005	0.00497995	0.500471

Parameter values: h = 0.1, k = 0.00001, $\mathcal{L}$ = 0.01, γ = 0, α_1 = 0.00001, α_2 = 0.001, β_1 = 0.002, β_2 = 0.00001, z = 10 ^ –8, y = 10 ^ –8

r	$\sim \hat{p}_T$ Analytical	$\hat{p}_T$ Numerical	Percentage Difference
0.001	0.00001	9.99911×10^{-6}	0.00890204
0.051	0.00001	9.99929×10^{-6}	0.00713407
0.101	0.00001	9.99929×10^{-6}	0.00708816
0.151	0.00001	9.99929×10^{-6}	0.00707233
0.201	0.00001	9.99929×10^{-6}	0.00706432
0.251	0.00001	9.99929×10^{-6}	0.00705947
0.301	0.00001	9.99929×10^{-6}	0.00705623
0.351	0.00001	9.99929×10^{-6}	0.00705391
0.401	0.00001	9.99929×10^{-6}	0.00705216
0.451	0.00001	9.99929×10^{-6}	0.0070508

Parameter values: h = 0.1, k = 0.00001, $\mathcal{L}$ = 0.01, α_1 = 0.00001, α_2 = 0.001, β_1 = 0.002, β_2 = 0.00001, z = 10 ^ –8, y = 10 ^ –8

Table 3 : Evaluating the accuracy of the equilibrium approximation as the selection coefficient against the transposon increases from a lower to upper limit

$\mathcal{L}$	~ $\hat{p}_B$ Analytical	$\hat{p}_B$ Numerical	Percentage Difference
0.001	0.0050504	0.00502495	0.503869
0.006	0.00500836	0.00498328	0.500816
0.011	0.00500454	0.00497949	0.500424
0.016	0.0050031	0.00497807	0.500277
0.021	0.00500235	0.00497733	0.500201
0.026	0.00500189	0.00497687	0.500154
0.031	0.00500158	0.00497656	0.500122
0.036	0.00500135	0.00497634	0.500099
0.041	0.00500118	0.00497617	0.500082
0.046	0.00500105	0.00497604	0.500069
0.051	0.00500094	0.00497593	0.500058
0.056	0.00500085	0.00497585	0.500049
0.061	0.00500078	0.00497577	0.500041
0.066	0.00500072	0.00497571	0.500035
0.071	0.00500066	0.00497566	0.50003
0.076	0.00500062	0.00497561	0.500025
0.081	0.00500058	0.00497557	0.500021
0.086	0.00500054	0.00497554	0.500017
0.091	0.00500051	0.0049755	0.500014
0.096	0.00500048	0.00497548	0.500011

Parameter values : h = 0.1, k = 0.00001, r = 0.1, α_1 = 0.00001, α_2 = 0.001, β_1 = 0.002, β_2 = 0.00001, z = 10 ^ –8, y = 10 ^ –8

$\mathcal{L}$	~ $\hat{p}_T$ Analytical	$\hat{p}_T$ Numerical	Percentage Difference
0.001	0.0001	0.000100107	0.10744
0.006	0.0000166667	0.0000166651	0.0096158
0.011	9.09091×10^{-6}	9.09031×10^{-6}	0.00660776
0.016	6.25×10^{-6}	6.24969×10^{-6}	0.00489416
0.021	4.7619×10^{-6}	4.76172×10^{-6}	0.00386908
0.026	3.84615×10^{-6}	3.84603×10^{-6}	0.00319468
0.031	3.22581×10^{-6}	3.22572×10^{-6}	0.00271897
0.036	2.77778×10^{-6}	2.77771×10^{-6}	0.00236593
0.041	2.43902×10^{-6}	2.43897×10^{-6}	0.00209371
0.046	2.17391×10^{-6}	2.17387×10^{-6}	0.0018775
0.051	1.96078×10^{-6}	1.96075×10^{-6}	0.00170167
0.056	1.78571×10^{-6}	1.78569×10^{-6}	0.0015559
0.061	1.63934×10^{-6}	1.63932×10^{-6}	0.00143308
0.066	1.51515×10^{-6}	1.51513×10^{-6}	0.00132822
0.071	1.40845×10^{-6}	1.40843×10^{-6}	0.00123763
0.076	1.31579×10^{-6}	1.31577×10^{-6}	0.0011586
0.081	1.23457×10^{-6}	1.23455×10^{-6}	0.00108904
0.086	1.16279×10^{-6}	1.16278×10^{-6}	0.00102736
0.091	1.0989×10^{-6}	1.09889×10^{-6}	0.000972282
0.096	1.04167×10^{-6}	1.04166×10^{-6}	0.000922805

Parameter values : h = 0.1, k = 0.00001, r = 0.1, α_1 = 0.00001, α_2 = 0.001, β_1 = 0.002, β_2 = 0.00001, z = 10 ^ –8, y = 10 ^ –8

Table 4 : Evaluating the accuracy of the equilibrium approximation as the epiallele background reversion rate increases from a lower to upper limit

β_1	~ $\hat{p}_B$ Analytical	$\hat{p}_B$ Numerical	Percentage Difference
0.001	0.0100099	0.00990973	1.0007
0.101	0.0000991954	0.0000991855	0.00993563
0.201	0.0000498884	0.0000498859	0.00501176
0.301	0.0000333435	0.0000333424	0.00336105
0.401	0.0000250504	0.0000250498	0.00253247
0.501	0.000020068	0.0000200676	0.00203189
0.601	0.0000167436	0.0000167433	0.00169405
0.701	0.0000143677	0.0000143674	0.001448
0.801	0.000012585	0.0000125848	0.00125823
0.901	0.000011198	0.0000111979	0.00110497

Parameter values: h = 0.1, k = 0.00001, r = 0.1, α_1 = 0.00001, α_2 = 0.001, $\mathcal{L}$ = 0.01, β_2 = 0.00001, z = 10 ^ –8, y = 10 ^ –8

β_1	~ $\hat{p}_T$ Analytical	$\hat{p}_T$ Numerical	Percentage Difference
0.001	0.00001	9.99928×10^{-6}	0.00717284
0.101	0.00001	9.99947×10^{-6}	0.00530569
0.201	0.00001	9.99964×10^{-6}	0.00356979
0.301	0.00001	9.99982×10^{-6}	0.00183269
0.401	0.00001	9.99999×10^{-6}	0.0000940281
0.501	0.00001	0.0000100002	0.00164625
0.601	0.00001	0.0000100003	0.00338817
0.701	0.00001	0.0000100005	0.00513175
0.801	0.00001	0.0000100007	0.00687698
0.901	0.00001	0.0000100009	0.00862388

Parameter values: h = 0.1, k = 0.00001, r = 0.1, α_1 = 0.00001, α_2 = 0.001, $\mathcal{L}$ = 0.01, β_2 = 0.00001, z = 10 ^ –8, y = 10 ^ –8

Note: For the parameter conditions assessed in the above tables, the corresponding eigenvalues were always between zero and one in absolute magnitude, suggesting local stability.

Model 4: Three locus diploid mating and selection, full derivation

Gamete Probabilities

Derivation of Recursion Equations

B1 - Selfing

B2 - Random mating

First, taking the product of the matrices (discussed in File S2 section C2):

Next substituting in the gamete probabilities from the previous section into the matrix:

B3 - Natural Selection

After mating, diploid individuals undergo natural selection at the final event.

$$p_{J,t,A,J,t,A}'' = \frac{w_1}{\overline{w}}\, p_{J,t,A,J,t,A}'$$

$$p_{J,t,A,J,t,B}'' = \frac{w_2}{\overline{w}}\, p_{J,t,A,J,t,B}'$$

$$p_{J,t,A,J,T,A}'' = \frac{w_3}{\overline{w}}\, p_{J,t,A,J,T,A}'$$

$$p_{J,t,A,J,T,B}'' = \frac{w_4}{\overline{w}}\, p_{J,t,A,J,T,B}'$$

$$p_{J,t,B,J,t,B}'' = \frac{w_5}{\overline{w}}\, p_{J,t,B,J,t,B}'$$

$$p_{J,t,B,J,T,A}'' = \frac{w_6}{\overline{w}}\, p_{J,t,B,J,T,A}'$$

$$p_{J,t,B,J,T,B}'' = \frac{w_7}{\overline{w}}\, p_{J,t,B,J,T,B}'$$

$$p_{J,T,A,J,T,A}'' = \frac{w_8}{\overline{w}}\, p_{J,T,A,J,T,A}'$$

$$p_{J,T,A,J,T,B}'' = \frac{w_9}{\overline{w}}\, p_{J,T,A,J,T,B}'$$

$$p_{J,T,B,J,T,B}'' = \frac{w_{10}}{\overline{w}}\, p_{J,T,B,J,T,B}'$$

$$p_{J,t,A,j,t,A}'' = \frac{w_{11}}{\overline{w}}\, p_{J,t,A,j,t,A}'$$

$$p_{J,t,A,j,t,B}'' = \frac{w_{12}}{\overline{w}}\, p_{J,t,A,j,t,B}'$$

$$p_{j,t,A,J,t,B}'' = \frac{w_{13}}{\overline{w}}\, p_{j,t,A,J,t,B}'$$

$$p_{J,t,A,j,T,A}'' = \frac{w_{14}}{\overline{w}}\, p_{J,t,A,j,T,A}'$$

$$p_{j,t,A,J,T,A}'' = \frac{w_{15}}{\overline{w}}\, p_{j,t,A,J,T,A}'$$

$$p_{J,t,A,j,T,B}'' = \frac{w_{16}}{\overline{w}}\, p_{J,t,A,j,T,B}'$$

$$p_{j,t,A,J,T,B}'' = \frac{w_{17}}{\overline{w}}\, p_{j,t,A,J,T,B}'$$

$$p_{J,t,B,j,t,B}'' = \frac{w_{18}}{\overline{w}} \, p_{J,t,B,j,t,B}'$$

$$p_{J,t,B,j,T,A}'' = \frac{w_{19}}{\overline{w}} \, p_{J,t,B,j,T,A}'$$

$$p_{j,t,B,J,T,A}'' = \frac{w_{20}}{\overline{w}} \, p_{j,t,B,J,T,A}'$$

$$p_{j,t,B,J,T,A}'' = \frac{w_{21}}{\overline{w}} \, p_{j,t,B,J,T,A}'$$

$$p_{J,t,B,j,T,B}'' = \frac{w_{22}}{\overline{w}} \, p_{J,t,B,j,T,B}'$$

$$p_{j,t,B,J,T,B}'' = \frac{w_{23}}{\overline{w}} \, p_{j,t,B,J,T,B}'$$

$$p_{J,T,A,j,T,A}'' = \frac{w_{24}}{\overline{w}} \, p_{J,T,A,j,T,A}'$$

$$p_{J,T,A,j,T,B}'' = \frac{w_{25}}{\overline{w}} \, p_{J,T,A,j,T,B}'$$

$$p_{j,T,A,J,T,B}'' = \frac{w_{26}}{\overline{w}} \, p_{j,T,A,J,T,B}'$$

$$p_{J,T,B,j,T,B}'' = \frac{w_{27}}{\overline{w}} \, p_{J,T,B,j,T,B}'$$

$$p_{j,t,A,j,t,A}'' = \frac{w_{28}}{\overline{w}} \, p_{j,t,A,j,t,A}'$$

$$p_{j,t,A,j,t,B}'' = \frac{w_{29}}{\overline{w}} \, p_{j,t,A,j,t,B}'$$

$$p_{j,t,A,j,T,A}'' = \frac{w_{30}}{\overline{w}} \, p_{j,t,A,j,T,A}'$$

$$p_{j,t,A,j,T,B}'' = \frac{w_{31}}{\overline{w}} \, p_{j,t,A,j,T,B}'$$

$$p_{j,t,B,j,t,B}'' = \frac{w_{32}}{\overline{w}} \, p_{j,t,B,j,t,B}'$$

$$p_{j,t,B,j,T,A}'' = \frac{w_{33}}{\overline{w}} \, p_{j,t,B,j,T,A}'$$

$$p_{j,t,B,j,T,B}'' = \frac{w_{34}}{\overline{w}}\, p_{j,t,B,j,T,B}'$$

$$p_{j,T,A,j,T,A}'' = \frac{w_{35}}{\overline{w}}\, p_{j,T,A,j,T,A}'$$

$$p_{j,T,A,j,T,B}'' = \frac{w_{36}}{\overline{w}}\, p_{j,T,A,j,T,B}'$$

where $p_{b,c,d,w,m,n}' = \left(p_{b,c,d,w,m,n}'\,(\text{from random mating}) + p_{b,c,d,w,m,n}'\,(\text{from selfing})\right)$

B4 - Final Recursion Equations
