# Supplementary material for: Multi-locus models of deleterious epimutation-selection balance and the evolution of recombination modifiers
Source: G3 (Bethesda). 2026 Jun 11;16(8):jkag147. doi: 10.1093/g3journal/jkag147 (PMC13439953; doi:10.1093/g3journal/jkag147)
Supplement: Supplementary file 1 [file jkag147_supplementary_data.zip › File_S7_G3-2026-406777.pdf]

The purpose of this derivation and analysis is to demonstrate the validity of the model and analysis regarding the assumptions on the transposon and regarding it as an individual genetic site, whereby the possibility of multiple transposon insertions is not considered. We calculate the stationary distribution for the number of transposons present at the genetic site. This follows a Markov processs, whereby the probability of an event only depends on the current state.

Let,

i = the number of transposons present

z = probability of a transposition

y = probabilitity of deletion

A stationary distribution $\pi = (\pi_0, \pi_1, \pi_2, ...)$ satisfies:

$\pi = \pi P$

or equivalently

$\pi_j = \sum_i \pi_i P(i \rightarrow j), \forall j.$

Because this represents a birth-death chain, it is reversible and detailed balance can be imposed:

$\pi_i P(i \rightarrow i+1) = \pi_{i+1}(i+1 \rightarrow i)$

When at least one transposon has inserted, i.e. i ≥ 1, there are three possible events. Another transposition event occurs at the same site, a transposon deletion occurs, or neither occur. More formally:

for i ≥ 1,

$P(i \to i-1) = y$, $P(i \to i) = 1 - z - y$, $P(i \to i+1) = z$

Since transposons cannot take on negative values, a reflection boundary at zero is imposed. When there are no transposons present at the genetic site, there are two possible events, transposition or no transpostion:
for $i = 0$ (the reflecting boundary),

$P(0 \to 0) = 1 - z$, $P(0 \to 1) = z$

I.e. deletion is not possible without a transposon.

Then,

For $i \geq 0$,

$$\pi_i z = \pi_{i+1} y$$

$$\pi_{i+1} = \pi_i \frac{z}{y}.$$

Let $x = \frac{z}{y}$,

then through iteration,

$$\pi_i = \pi_0 x^i.$$

$$\sum_{i=0}^{\infty} \pi_i = 1,$$

$$\sum_{i=0}^{\infty} \pi_0 x^i = 1,$$

$$\pi_0 \sum_{i=0}^{\infty} x^i = 1,$$

$$\sum_{i=0}^{\infty} x^i = \frac{1}{1-x},$$

$$\pi_0 \sum_{i=0}^{\infty} x^i = 1,$$

$$\pi_0 \left(\frac{1}{1-x}\right) = 1.$$

Since $\pi_0 = 1 - x$,

for $z < y < 1$,

$$\pi_i = \pi_0 x^i$$

$$\pi_i = (1-x) x^i$$

Back substituting for x, we obtain the stationary distribution for the number of transposons,

$$\pi_i = \left(1 - \frac{z}{y}\right)\left(\frac{z}{y}\right)^i = \left(\frac{y-z}{y}\right)\left(\frac{z}{y}\right)^i$$

Next, simplifying the terms,

$$\pi_i = \left(1 - \frac{z}{y}\right)\left(\frac{z}{y}\right)^i = \left(\frac{y-z}{y}\right)\left(\frac{z}{y}\right)^i$$

For the purposes of the model assumptions whereby only one transposon (or none) is allowed to be present at a site. We first calculate the probability of having more than one transposon present:

$P(i > 1) + P(i \leqslant 1) = 1$

$P(i > 1) = 1 - P(i \leqslant 1)$

$P(i > 1) = 1 - (P(i = 0) + P(i = 1))$

$$P(i > 1) = 1 - \left(\left(\frac{y-z}{y}\right)\left(\frac{z}{y}\right)^0 + \left(\frac{y-z}{y}\right)\left(\frac{z}{y}\right)^1\right)$$

$$P(i > 1) = 1 - \left(\left(\frac{y-z}{y}\right) + \left(\frac{y-z}{y}\right)\left(\frac{z}{y}\right)\right)$$

$$P(i > 1) = 1 - \left(1 - \frac{z^2}{y^2}\right)$$

$$P(i > 1) = \frac{z^2}{y^2}$$

Let, $y = az$, where $a > 1$.

Assume that a probability of less than 0.001 of having more than one transposon is desired:

$$\frac{1}{a^2} < 0.001$$

$$1 < 0.001\, a^2$$

$$1000 < a^2$$

$$\sim 32 < a$$

The deletion rate must be greater than ~32x the transposition rate for a less than 0.001 probability of more than one transposon being present at the site. Generally, y = 50z or higher was used throughout the numerical analysis of the model, such that the analytical approximations are accurate under conditions when more than one transposon insertion is small.
