# Supplementary material for: Multi-locus models of deleterious epimutation-selection balance and the evolution of recombination modifiers
Source: G3 (Bethesda). 2026 Jun 11;16(8):jkag147. doi: 10.1093/g3journal/jkag147 (PMC13439953; doi:10.1093/g3journal/jkag147)
Supplement: Supplementary file 1 [file jkag147_supplementary_data.zip › Supplemental_Material_Legend_G3-2026-406777.pdf]

Supplemental Material

File S1. Overview of the derivation for the haploid models. This file gives an introduction and overview for all model presented in the main text (models 1 and 2).

File S2. Overview of the derivation for the diploid models. This file gives an introduction and overview for the results presented in File S3 (models 3 and 4).

File S3. Summary of diploid models results. This file presents the key analytical and numerical results for the diploid setting.

File S4. Detailed derivation and analysis of haploid models. This file goes through the detailed calculations and analysis for the haploid models (1 and 2) and results corresponding to the main text with additional supporting data.

File S5. Detailed derivation and analysis of diploid models. This file goes through the detailed calculations and analysis for the diploid models (3 and 4) and results corresponding File S3 with additional supporting data.

File S6. Python code for model 3 simulations. This file gives the general code used for python simulations for model 3 in the main text.

File S7. Mathematical justification for transposition/deletion assumptions. This file provides a mathematical justification for the assumptions of the transposition/deletions process at a locus.
